# Transcriptional substrates underlying functional connectivity profiles of subregions within the human sensorimotor cortex

**DOI:** 10.1002/hbm.26031

**Published:** 2022-07-27

**Authors:** Yuhao Shen, Cun Zhang, Shunshun Cui, Rui Wang, Huanhuan Cai, Wenming Zhao, Jiajia Zhu, Yongqiang Yu

**Affiliations:** ^1^ Department of Radiology The First Affiliated Hospital of Anhui Medical University Hefei China; ^2^ Research Center of Clinical Medical Imaging Hefei Anhui Province China; ^3^ Anhui Provincial Institute of Translational Medicine Hefei China

**Keywords:** Allen human brain atlas, functional connectivity, gene expression, resting‐state fMRI, sensorimotor cortex, subregion

## Abstract

The human sensorimotor cortex has multiple subregions showing functional commonalities and differences, likely attributable to their connectivity profiles. However, the molecular substrates underlying such connectivity profiles are unclear. Here, transcriptome‐neuroimaging spatial correlation analyses were performed between transcriptomic data from the Allen human brain atlas and resting‐state functional connectivity (rsFC) of 24 fine‐grained sensorimotor subregions from 793 healthy subjects. Results showed that rsFC of six sensorimotor subregions were associated with expression measures of six gene sets that were specifically expressed in brain tissue. These sensorimotor subregions could be classified into the polygenic‐ and oligogenic‐modulated subregions, whose rsFC were related to gene sets diverging on their numbers (hundreds vs. dozens) and functional characteristics. First, the former were specifically expressed in multiple types of neurons and immune cells, yet the latter were not specifically expressed in any cortical cell types. Second, the former were preferentially expressed during the middle and late stages of cortical development, while the latter showed no preferential expression during any stages. Third, the former were prone to be enriched for general biological functions and pathways, but the latter for specialized biological functions and pathways. Fourth, the former were enriched for neuropsychiatric disorders, whereas this enrichment was absent for the latter. Finally, although the identified genes were commonly associated with sensorimotor behavioral processes, the polygenic‐modulated subregions associated genes were additionally related to vision and dementia. These findings may advance our understanding of the functional homogeneity and heterogeneity of the human sensorimotor cortex from the perspective of underlying genetic architecture.

## Graphical abstract

Our data represent the first report to date investigating the molecular mechanisms underlying rsFC of the sensorimotor cortex at the subregional level. Based on our results, the sensorimotor cortex can be conceptualized as the polygenic‐ and oligogenic‐modulated subregions, whose rsFC were related to gene sets diverging on their numbers and functional characteristics. These findings may advance our understanding of the functional homogeneity and heterogeneity of the human sensorimotor cortex from the perspective of underlying genetic architecture.

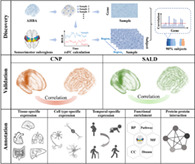

## 1 INTRODUCTION

The human sensorimotor cortex is a complex region that has multiple subregions showing commonalities and differences in their functions. Based on classical Brodmann map, the sensorimotor cortex is divided into several subregions including the primary somatosensory cortex (BA1, 2, and 3), somatosensory association cortex (BA5), primary motor cortex (BA4), and premotor and supplementary motor cortex (BA6) (Zilles & Amunts, 2010). Conventionally, the former two are mainly involved in somatic sensory experiences and processing (Kania, 2021), while the latter two are primarily implicated in aspects of movement including motor execution and planning (Graziano, 2006). Moreover, body parts are also a well‐known organizing principle for the sensorimotor cortex (Penfield & Boldrey, 1937), that is, distinct body parts could be mapped to different sensorimotor subdivisions. This principle is similar to the distinction between sensory and motor processing, complementing and extending the Brodmann model. This important organizing principle is recently confirmed by empirical evidence from neuroimaging (Kuehn et al., 2017).

It is well established that information processing entails complex interactions among widely distributed regions in the brain. Advances in resting‐state functional magnetic resonance imaging (rs‐fMRI) (Logothetis et al., 2001; Rosen & Savoy, 2012), in concert with resting‐state functional connectivity (rsFC) approaches measuring the temporal correlation of blood‐oxygen‐level‐dependent (BOLD) signals between spatially different brain areas (Biswal et al., 1995; Yeo et al., 2011), have facilitated a more thorough characterization of such interactions. Using a combination of rs‐fMRI and rsFC, prior research has revealed that the sensorimotor cortex can be parcellated into distinct subregions with different rsFC profiles (Li et al., 2015; Long et al., 2014), partly in favor of the notion that connectivity variations of sensorimotor subregions might make a significant contribution to their functional differences. Crucially, there is strong evidence that the rsFC profiles of sensorimotor subregions are highly stereotyped between individuals (Mueller et al., 2013), indicating a conservative biological process responsible for their development that is however far from being understood. More generally, an extensive literature has documented selective dysfunctional connectivity of sensorimotor subregions in many brain disorders (e.g., anxiety [Arnold Anteraper et al., 2014; Liao et al., 2010], schizophrenia [Wei, Chang, et al., 2018; Yu et al., 2021], Parkinson's disease [Canu et al., 2015; Horn et al., 2017; Wu et al., 2011], autism [Carper et al., 2015; Linke et al., 2020], and migraine [Qin et al., 2020]), such that clarification of the molecular substrates underlying the rsFC variations across sensorimotor subregions may not only help achieve a deeper understanding of disease neuropathology, but also have important clinical implications for developing new treatment strategies targeted toward disease‐related molecular substrates.

The current availability of comprehensive, brain‐wide transcriptomic atlases (e.g., the Allen human brain atlas [AHBA]; Hawrylycz et al., 2012) has made it increasingly feasible to relate microscale molecular function to macroscale brain organization. The combination of brain transcriptional and imaging data has refueled the formulation of the nascent realm of neuroimaging transcriptomics, the objective of which is to investigate the genetic substrates underlying various neuroimaging phenotypes (Fornito et al., 2019). The most frequently used approach in this realm is transcription‐neuroimaging spatial correlation analysis, that is, relating spatial variations in gene expression to anatomical variations in a given neuroimaging measure (Liu et al., 2022). Taking advantage of this powerful approach, there have been recent efforts to examine the genetic architecture of rsFC, which have enjoyed significant success in the identification of genes with spatial expression patterns tracking spatial distribution of rsFC (Anderson et al., 2018; Chen et al., 2022; Hawrylycz et al., 2015; Krienen et al., 2016; Richiardi et al., 2015; Vertes et al., 2016; Zhang et al., 2022; Zhu et al., 2021). Despite this prior work, there is still a dearth of literature investigating the genetic underpinnings of the rsFC profiles of different sensorimotor subregions. Elucidating such relationship will be contingent on conducting a sophisticated transcriptome‐rsFC spatial association analysis by use of tailored methodologies, including standardized transcriptomic data processing and rsFC measurement based on more fine‐grained parcellation of the sensorimotor cortex.

**FIGURE 1:**
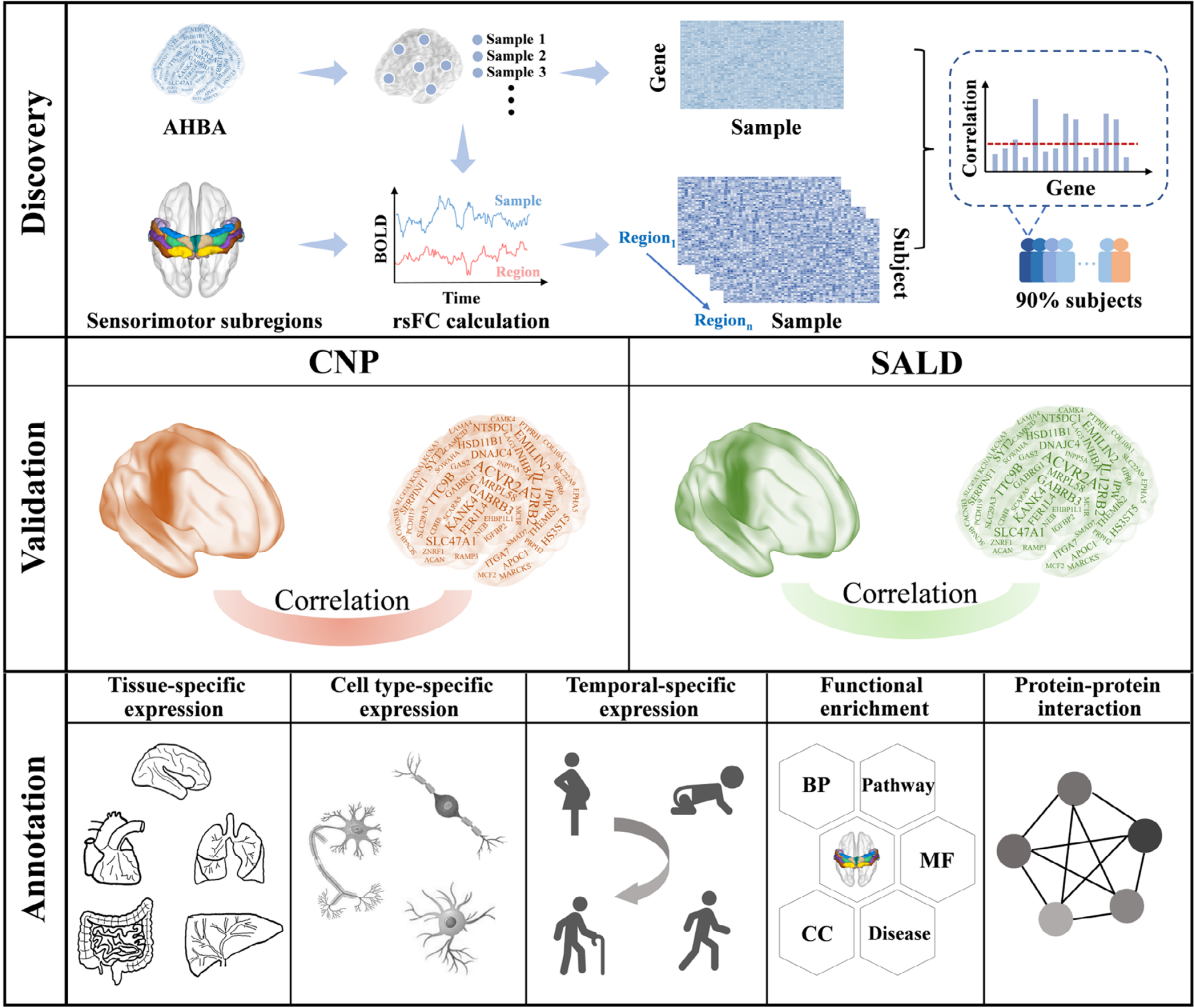
A schematic overview of the study design and analysis pipeline. First, we defined 24 subregions for the bilateral sensorimotor cortex according to the human brainnetome atlas. For a given subregion, we computed its rsFC to each brain tissue sample from the AHBA, resulting in a subregion‐samples rsFC vector. This rsFC calculation was performed for each subject to obtain a sample × subject rsFC matrix for each sensorimotor subregion. Second, we used the newly proposed pipeline to process brain transcriptomic data, resulting in normalized transcriptomic data of 5013 genes for 1280 tissue samples. Third, we conducted transcriptome‐neuroimaging spatial correlation analyses to identify genes associated with rsFC of each sensorimotor subregion. In the discovery experiment, cross‐sample Pearson's correlations between gene expression and rsFC were conducted in a gene‐wise manner, resulting in 5013 correlation coefficients for each subject. Next, genes with significant correlations in more than 90% of subjects were used for further validation analyses. In the validation experiment, the same analysis procedure was performed for the CNP and SALD datasets, respectively. Only genes consistently identified in both discovery and validation experiments were considered rsFC‐related genes. Finally, a set of annotation analyses were performed for the identified rsFC‐related genes including tissue‐specific expression, cell type‐specific expression, temporal‐specific expression, functional enrichment, and protein–protein interaction. AHBA, Allen human brain atlas; BOLD, blood‐oxygen‐level‐dependent; CNP, Consortium for Neuropsychiatric Phenomics; rsFC, resting‐state functional connectivity; SALD, Southwest University Adult Lifespan Dataset

Here, we utilized a newly proposed standardized pipeline (Arnatkeviciute et al., 2019) to process brain transcriptomic data from the AHBA dataset. Seed‐based rsFC analysis was performed using rs‐fMRI data of 793 healthy subjects from a discovery dataset and two independent cross‐scanner cross‐race validation datasets. Critically, 24 fine‐grained sensorimotor subregions were defined as the seeds according to the human brainnetome atlas (Fan et al., 2016), which is based on connectional architecture and thus optimized for connectivity analysis. Then, we conducted transcriptome‐neuroimaging spatial correlation analyses to identify genes associated with rsFC of each sensorimotor subregion, whose functional characteristics were further analyzed by gene functional annotation. A schematic overview of the study design and analysis pipeline is illustrated in Figure 1. We aimed to test the hypothesis of commonalities and differences in the identified genes and their functional characteristics across sensorimotor subregions.

## 2 METHODS

### 2.1 Participants

Our study included a discovery dataset as well as two independent cross‐scanner and cross‐race validation datasets. The discovery participants were healthy adults of Chinese Han and right handedness, enrolled from the local universities and community through poster advertisements. Exclusion criteria included neuropsychiatric or severe somatic disorder, a history of head injury with consciousness loss, pregnancy, MRI contraindications, and a family history of psychiatric illness among first‐degree relatives. Written informed consent was obtained from all participants after they had been given a complete description of the study. This study was approved by the ethics committee of The First Affiliated Hospital of Anhui Medical University. The validation samples were from two publically available datasets: the Consortium for Neuropsychiatric Phenomics (CNP, https://openneuro.org/datasets/ds000030/versions/1.0.0) (Poldrack et al., 2016), and the Southwest University Adult Lifespan Dataset (SALD, https://doi.org/10.15387/fcp_indi.sald) (Wei, Zhuang, et al., 2018). It is noteworthy that we only selected the healthy adults from the cross‐disorder CNP dataset. Full details about the two validation samples (e.g., ethics, informed consent, inclusion and exclusion criteria, among others) have been provided in the data descriptor literature (Poldrack et al., 2016; Wei, Zhuang, et al., 2018). To exclude the potential impact of neurodevelopment and neurodegeneration, all the participants were restricted to an age range of 18–60 years. In addition, we excluded participants with poor image quality or excessive head motion during scanning. This brought the final samples to 361 in the discovery dataset, 103 in the CNP dataset, and 329 in the SALD dataset. Details of the demographic data of the three datasets are described in Table S1.

### 2.2 Image acquisition

MRI data of the discovery sample were acquired using the 3.0‐Tesla General Electric Discovery MR750w scanner, and those of the validation samples were obtained using the 3.0‐Tesla Siemens Trio scanners. Details of the resting‐state fMRI protocols for the three datasets are described in Table S2.

### 2.3 Image processing

Resting‐state BOLD data were preprocessed using Statistical Parametric Mapping software (SPM12, http://www.fil.ion.ucl.ac.uk/spm) and Data Processing & Analysis for Brain Imaging (DPABI, http://rfmri.org/dpabi) (Yan et al., 2016). The first several time points (discovery: 10, CNP: 5, SALD: 10) for each participant were discarded to allow the signal to reach equilibrium and the participants to adapt to the scanning noise. The remaining volumes were corrected for the acquisition time delay between slices. Then, realignment was performed to correct the motion between time points. Head motion parameters were assessed by calculating the translation in each direction and the angular rotation on each axis for each volume. All BOLD data of the final sample were within the defined motion thresholds (i.e., maximum translation or rotation <2 mm or 2°). We also computed frame‐wise displacement (FD), which measures the volume‐to‐volume changes in head position. Several nuisance covariates (the linear drift, the estimated motion parameters based on the Friston‐24 model, the spike volumes with FD >0.5, the white matter signal, and the cerebrospinal fluid signal) were regressed out from the data. The datasets were then band‐pass filtered using a frequency range of 0.01–0.1 Hz. In the normalization step, individual structural images were firstly co‐registered with the average functional images; then the transformed structural images were segmented and normalized to the Montreal Neurological Institute (MNI) space using a high‐level nonlinear warping algorithm, that is, the diffeomorphic anatomical registration through the exponentiated Lie algebra (DARTEL) technique (Ashburner 2007). Finally, each filtered functional volume was spatially normalized to MNI space using the deformation parameters estimated during the above step and resampled into a 3‐mm cubic voxel.

### 2.4 Definition of sensorimotor subregions

**FIGURE 2:**
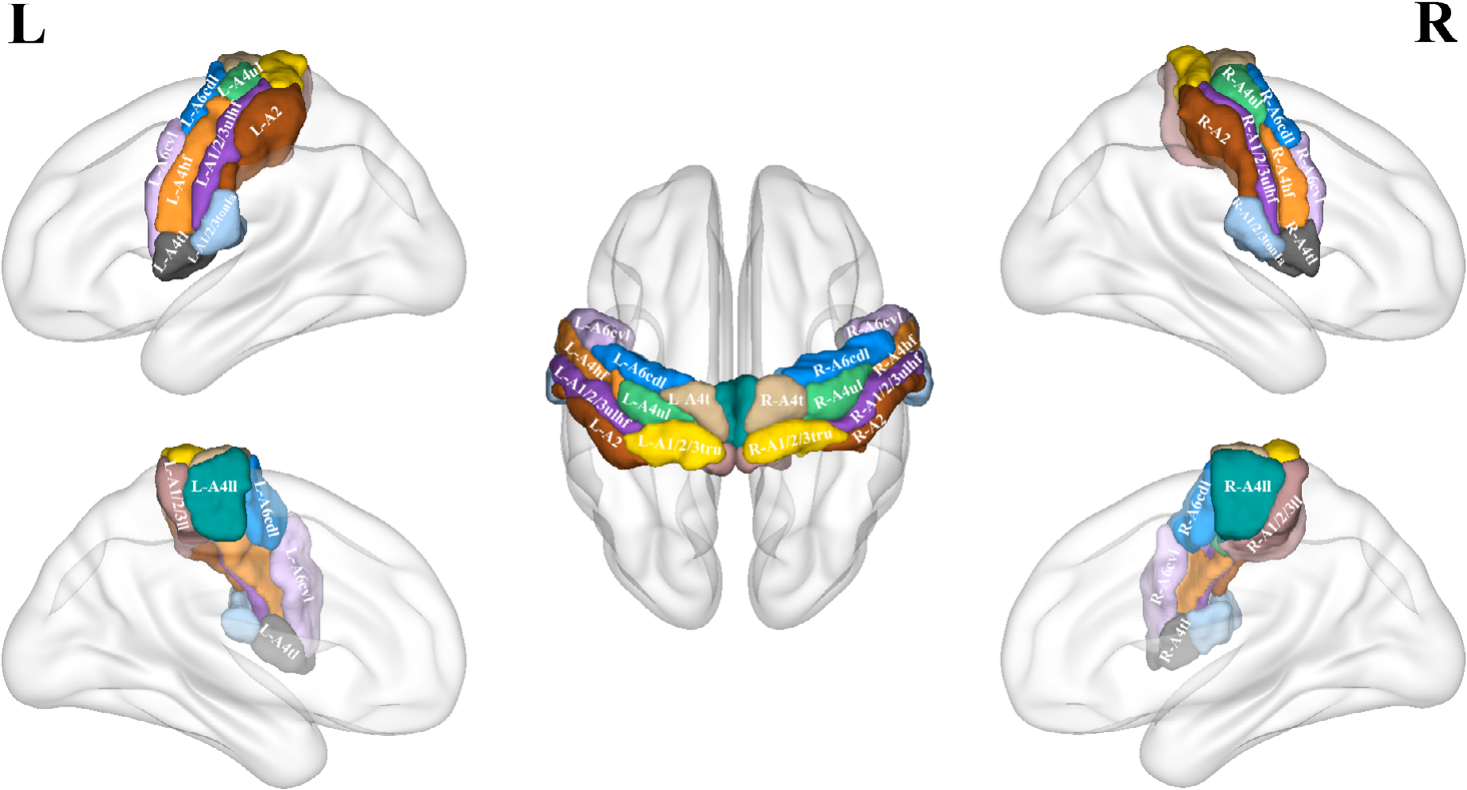
Illustration of sensorimotor subregions. A1/2/3ll, lower limb region of area 1/2/3; A1/2/3tonIa, tongue and larynx region of area 1/2/3; A1/2/3tru, trunk region of area 1/2/3; A1/2/3ulhf, upper limb, head, and face region of area 1/2/3; A2, area 2; A4hf, head and face region of area 4; A4ll, lower limb region of area 4; A4t, trunk region of area 4; A4tl, tongue and larynx region of area 4; A4ul, upper limb region of area 4; A6cdl, caudal dorsolateral area 6; A6cvl, caudal ventrolateral area 6; L, left; R, right

Subregions of the sensorimotor cortex were defined according to a connectivity‐based brain parcellation study (Fan et al., 2016). In that study, these subregions were initially parcellated based on the connectional architecture mapped with probabilistic tractography using diffusion MRI, and were further validated using resting‐state functional connectivity, tractography‐based anatomical connectivity, and meta‐analysis based functional behavioral decoding. In each hemisphere, the precentral gyrus was divided into head and face region of Brodmann area 4 (A4hf), caudal dorsolateral Brodmann area 6 (A6cdl), upper limb region of Brodmann area 4 (A4ul), trunk region of Brodmann area 4 (A4t), tongue and larynx region of Brodmann area 4 (A4tl), and caudal ventrolateral Brodmann area 6 (A6cvl); the paracentral lobule into lower limb region of Brodmann area 1/2/3 (A1/2/3ll) and lower limb region of Brodmann area 4 (A4ll); and the postcentral gyrus into upper limb, head and face region of Brodmann area 1/2/3 (A1/2/3ulhf), tongue and larynx region of Brodmann area 1/2/3 (A1/2/3tonIa), Brodmann area 2 (A2), and trunk region of Brodmann area 1/2/3 (A1/2/3tru). Thus, a total of 24 subregions for the bilateral sensorimotor cortex were defined (Figure 2).

### 2.5 Brain transcriptomic data processing

The brain transcriptome was obtained from the downloadable AHBA dataset (http://www.brain-map.org) (Hawrylycz et al., 2012, 2015), which was derived from six human post‐mortem donors (Table S3). The expression of more than 20 000 genes at 3702 spatially distinct brain tissue samples was measured using Custom 64K Agilent microarrays. The newly proposed pipeline was adopted to process brain transcriptomic data (Arnatkeviciute et al., 2019). First, the probe‐to‐gene annotations were updated based on the latest available information from the National Center for Biotechnology Information (NCBI) using the Re‐Annotator package (Xue et al., 2015). Next, probes that did not exceed the background noise in at least 50% of samples across all donors were excluded using intensity‐based filtering. Since expression level of a single gene was measured using multiple probes, the RNA‐seq data were further used as a reference to select probes. We initially ruled out genes that do not overlap between RNA‐seq and microarray datasets. Then, we calculated the correlations between microarray and RNA‐seq expression measures for the remaining genes. After excluding probes with low correlations (*r* < 0.2), a representative probe with the highest correlation to the RNA‐seq data was selected for a gene. Here, only the tissue samples in the left cerebral cortex were included. For one, all six donors had transcriptomic data in the left hemisphere with only two donors having samples in the right hemisphere. For another, the inclusion of subcortical samples might introduce potential biases considering the substantial divergence in transcriptome between cortical and subcortical regions (Hawrylycz et al., 2012). To adjust for potential inter‐sample differences and donor‐specific effects in transcriptome, we used the scaled robust sigmoid normalization method to conduct both within‐sample cross‐gene and within‐gene cross‐sample normalization. Differential stability (DS) represents an index of consistent regional variation across donor brains. Prior research has demonstrated that genes with higher DS values show more consistent spatial transcriptomic profiles across donors (Hawrylycz et al., 2015). Because gene expression conservation across subjects is a prerequisite for the transcriptome‐neuroimaging spatial correlations, only genes with relatively more conserved expression patterns were selected for analysis. To achieve this goal, we ranked the genes by their DS values and selected the 50% of the highest DS genes for the main analysis. Furthermore, to evaluate the effect of different DS threshold selections, we conducted sensitivity analyses by using two other DS cutoff thresholds (top 40% and 60%). After these processing procedures, we obtained normalized transcriptomic data of 5013 genes for 1280 tissue samples, that is, a sample × gene matrix of 1280 × 5013.

### 2.6 Calculation of rsFC of sensorimotor subregions with tissue samples

rsFC of sensorimotor subregions with tissue samples were calculated using the preprocessed functional MRI data from the discovery and validation datasets based on the MNI coordinates of 1280 tissue samples from the AHBA. First, we drew a sphere (radius = 3 mm) centered at the MNI coordinate of each tissue sample and extracted the average BOLD time course of voxels within each sphere. Then, we calculated Pearson's correlation coefficient between the average time courses of a sensorimotor subregion and each sample, yielding a subregion‐samples rsFC vector for each subject. Finally, the Pearson's correlation coefficients were converted to Fisher's *Z*‐scores to improve normality. This procedure was applied to all participants and we ultimately obtained a sample × subject rsFC matrix for each sensorimotor subregion.

### 2.7 Identification of genes associated with rsFC of sensorimotor subregions

Spatial correlation analyses were used to identify genes related to rsFC of each sensorimotor subregion separately. In the discovery experiment, we performed cross‐sample (1280 samples) Pearson's correlations between gene expression and rsFC in a gene‐wise manner (5013 genes), generating 5013 correlation coefficients for each subject. Then, genes with significant correlations (correction for the number of genes using the Benjamini and Hochberg method for false discovery rate [FDR‐BH]) in more than 90% of subjects were used for further validation analyses. In the validation experiment, we performed identical analysis procedure for the CNP and SALD datasets, respectively. Only genes consistently identified in both discovery and validation experiments were considered rsFC‐related genes. For ease of interpretability, genes associated with the homotopic subregions (e.g., the left and right A4hf) were pooled for subsequent analysis.

To further examine whether the number of the identified rsFC‐related genes was greater than random level, a permutation test was pursued to determine the significance of our results. As both transcriptomic and rsFC data are spatially autocorrelated, the standard non‐parametric null (i.e., randomly shuffling the sample labels) will yield increased family‐wise error rates (Markello & Misic, 2021). Thus, we used a spatially constrained null model (Burt et al., 2020) to carry out the permutation test, because it can simulate volumetric surrogate brain maps that preserve the spatial autocorrelation using three‐dimensional Euclidean distance between regions. This method is implemented in an open‐access, Python‐based software package, BrainSMASH: Brain Surrogate Maps with Autocorrelated Spatial Heterogeneity (https://github.com/murraylab/brainsmash). To correct the spatial autocorrelation in transcriptomic data, this method was utilized to generate spatial autocorrelation‐preserving surrogate maps for each gene. These surrogate maps were used to re‐identify the rsFC‐related genes using exactly the same method as described above. Then, we repeated this procedure 1000 times and recorded the number of genes identified in each test to build a null distribution. Finally, we compared the number of genes identified using the real data with this null distribution to determine whether our results were different from random.

### 2.8 Gene enrichment analysis

For each sensorimotor subregion, a set of enrichment analyses were performed for the identified rsFC‐related genes. First, we used online tissue‐specific expression analysis (TSEA) tool (http://genetics.wustl.edu/jdlab/tsea/) to determine the specific tissues in which the rsFC‐related genes were overrepresented. In case of specific expression in brain tissue, we used online cell type‐specific expression analysis (CSEA) tool (http://genetics.wustl.edu/jdlab/csea-tool-2/) (Dougherty et al., 2010; Xu et al., 2014) to conduct cell type and temporal specific expression analyses. For these analyses, we adopted a novel statistic, the specificity index, which can be used for comparative quantitative analysis to identify genes enriched in specific cell populations across a large number of profiles (Dougherty, et al., 2010). Here, a specificity index probability (*p*_SI_ = .05) was used to assess how likely a gene was to be specifically expressed. Second, these rsFC‐related genes were functionally annotated using the ToppGene portal (https://toppgene.cchmc.org/) (Chen et al., 2009). Gene ontology (GO) was used to determine their biological functions including molecular functions (MFs), biological processes (BPs), and cellular components (CCs). The pathway and disease databases were employed to determine the biological pathways and diseases in which these genes were overrepresented. Finally, we tested the overlap between the rsFC‐related genes identified in this study and those reported in earlier rsFC studies (Anderson et al., 2018; Richiardi et al., 2015). For the above‐described enrichment analyses, Fisher's exact tests were utilized to evaluate their statistical significance. Correction for multiple tests was performed using the FDR‐BH with a corrected *p* value of .05.

### 2.9 Capturing the behavioral relevance of genes related to rsFC of sensorimotor subregions

To further examine the behavioral relevance of the identified rsFC‐related genes, we tested the associations of gene expression with behavioral domains by use of the Neurosynth (https://neurosynth.org/), a well‐validated and publicly available platform for meta‐analysis of functional neuroimaging literature (Yarkoni et al., 2011). The Neurosynth database provides a range of activation maps of behavioral terms capturing conceptually distinct aspects of human behavior. Cross‐sample spatial correlation analyses were performed between expression measures of the identified rsFC‐related genes and activation values in each behavioral term map. For each behavioral term, we obtained a set of correlation coefficients corresponding to the number of the rsFC‐related genes. A positive correlation coefficient indicates that a brain region with higher gene expression tends to show greater neural activation, while a negative correlation coefficient means that a brain region with lower gene expression tends to show greater neural activation. Thus, both positive and negative correlation coefficients indicate that a gene contributes to a behavioral term. To avoid biases due to offset, we averaged the absolute values of these correlation coefficients (|*r*|_mean_) to index the extent to which this set of genes were linked to each behavioral term. Consequently, the behavioral terms were ordered based on their |*r*|_mean_ and those with higher |*r*|_mean_ were selected to capture the behavioral relevance of genes associated with rsFC of each sensorimotor subregion. Here, a threshold of |*r*|_mean_ >0.2 was used for visualization and interpretation.

### 2.10 Protein–protein interaction analysis

STRING v11.0 (https://string-db.org/) (Szklarczyk et al., 2019) was utilized to conduct protein–protein interaction (PPI) analysis, that is, constructing PPI networks and identifying hub genes from the rsFC‐related genes. We defined hub genes with the top 10% of the node degree in the PPI networks using a highest confidence interaction score of 0.9. In addition, we used the Human Brain Transcriptome database (http://hbatlas.org/) to characterize the spatial–temporal expression trajectory of hub genes with the highest node degree.

### 2.11 Sensitivity analysis

Considering that global signal regression (GSR) is a controversial topic in rs‐fMRI analyses (Murphy & Fox, 2017), we re‐computed rsFC of each sensorimotor subregion based on BOLD data with GSR and then repeated the entire analyses.

## 3 RESULTS

### 3.1 rsFC patterns of sensorimotor subregions

**FIGURE 3:**
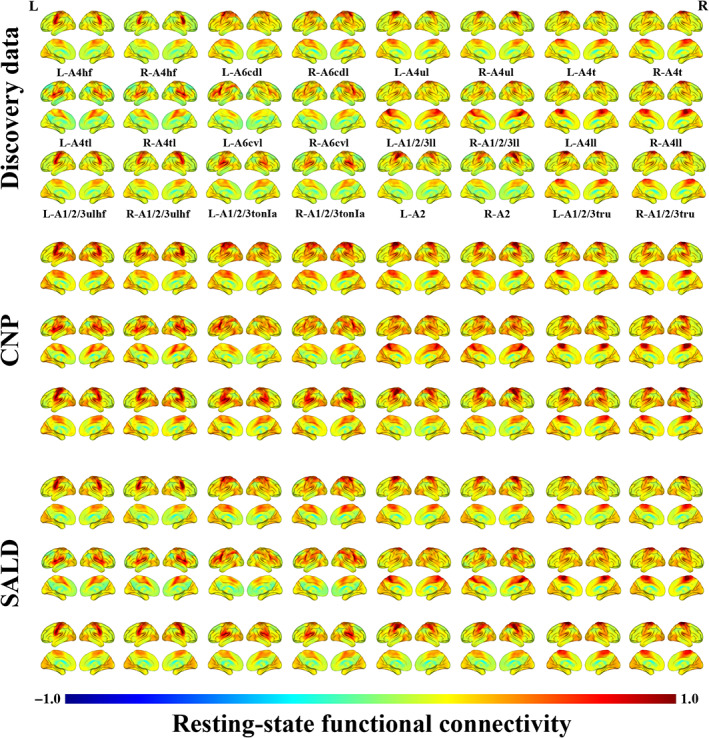
rsFC maps of sensorimotor subregions in three datasets. The maps reflect rsFC of a given sensorimotor subregion (seed region) to voxels across the entire brain. A1/2/3ll, lower limb region of area 1/2/3; A1/2/3tonIa, tongue and larynx region of area 1/2/3; A1/2/3tru, trunk region of area 1/2/3; A1/2/3ulhf, upper limb, head, and face region of area 1/2/3; A2, area 2; A4hf, head and face region of area 4; A4ll, lower limb region of area 4; A4t, trunk region of area 4; A4tl, tongue and larynx region of area 4; A4ul, upper limb region of area 4; A6cdl, caudal dorsolateral area 6; A6cvl, caudal ventrolateral area 6; CNP, the Consortium for Neuropsychiatric Phenomics; L, left; R, right; rsFC, resting‐state functional connectivity; SALD, the Southwest University Adult Lifespan Dataset

The rsFC maps of sensorimotor subregions in the discovery and validation datasets are illustrated in Figure 3. Overall, each subregion exhibited similar rsFC patterns across the three datasets. Although different sensorimotor subregions had consistent strong connections with lower‐order, primary, and unimodal cortex subserving sensory and motor functions, there was some variability in their rsFC patterns, which were largely consistent with the previous findings (Fan et al., 2016).

### 3.2 Genes related to rsFC of sensorimotor subregions

**FIGURE 4:**
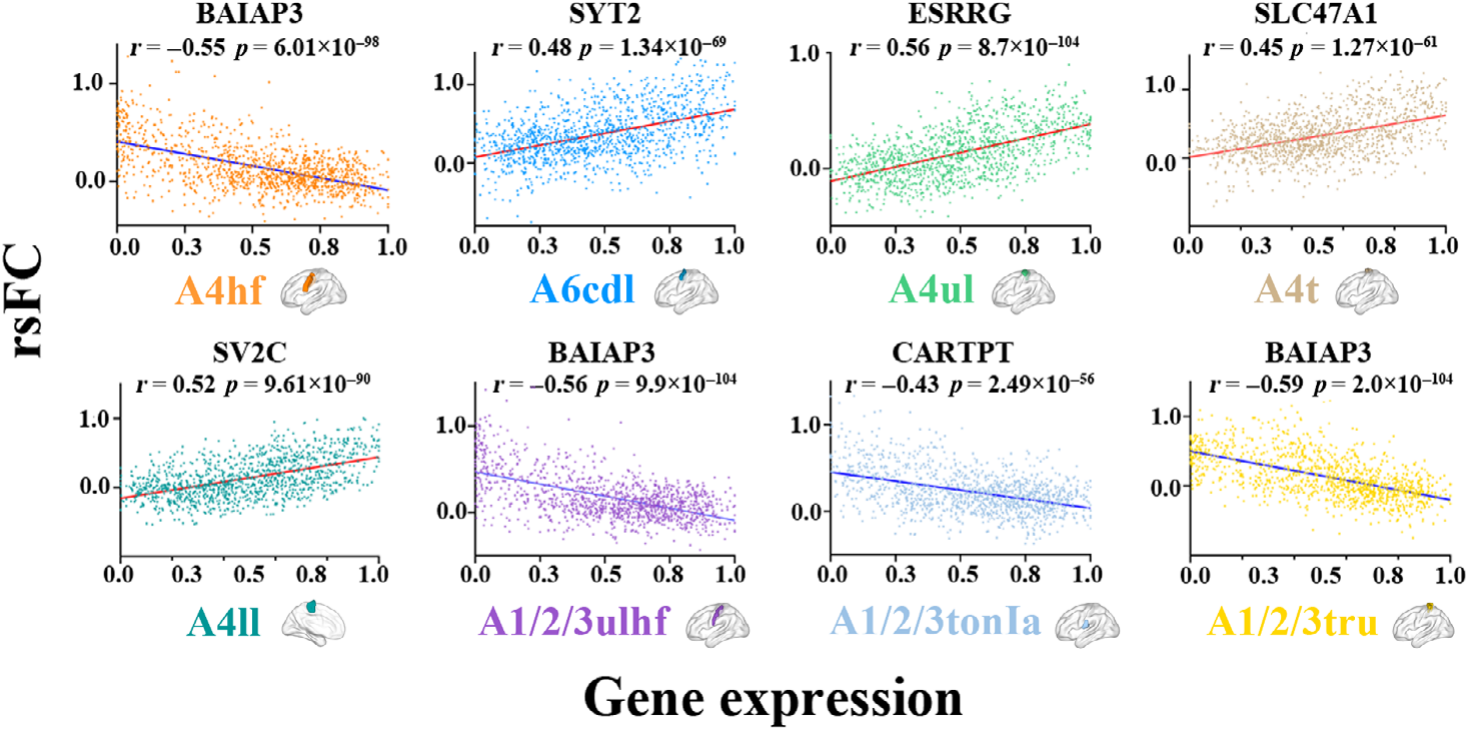
Scatter plots of the representative correlations between gene expression and rsFC of each sensorimotor subregion. The *p* value was corrected using the FDR‐BH. A1/2/3tonIa, tongue and larynx region of area 1/2/3; A1/2/3tru, trunk region of area 1/2/3; A1/2/3ulhf, upper limb, head, and face region of area 1/2/3; A4hf, head and face region of area 4; A4ll, lower limb region of area 4; A4t, trunk region of area 4; A4ul, upper limb region of area 4; A6cdl, caudal dorsolateral area 6; FDR‐BH, Benjamini and Hochberg method for false discovery rate; rsFC, resting‐state functional connectivity

For each sensorimotor subregion, gene expression‐rsFC spatial correlations were performed at the individual level in the discovery and validation datasets. Only genes with significant correlations (*p* < .05, FDR‐BH corrected) in more than 90% of subjects in all the three datasets were considered rsFC‐related genes. Consequently, we found 11 sets of genes associated with rsFC of 11 sensorimotor subregions (see Supplementary file 1 for the pooled genes and Supplementary file 2 for genes related to rsFC of left and right sensorimotor subregions separately). Specifically, expression measures of 470 genes were correlated with rsFC of the A4hf, 69 with the A6cdl, 821 with the A4ul, 11 with the A4t, 5 with the A4tl, 2 with the A1/2/3ll, 35 with the A4ll, 734 with the A1/2/3ulhf, 15 with the A1/2/3tonIa, 50 with A2, and 362 with the A1/2/3tru. Moreover, with exception of the A4tl, A1/2/3ll, and A2, spatially constrained permutation tests demonstrated that our results were different from random for the other eight subregions (*p*_perm_ <.05) (Table S4), which were used for further analysis. The overlapping genes between different sensorimotor subregions are shown in Figure S1. Scatter plots of the representative correlations between gene expression and rsFC of each sensorimotor subregion in the discovery dataset are shown in Figure 4.

### 3.3 Tissue, cell type, and temporal specific expression

**FIGURE 5:**
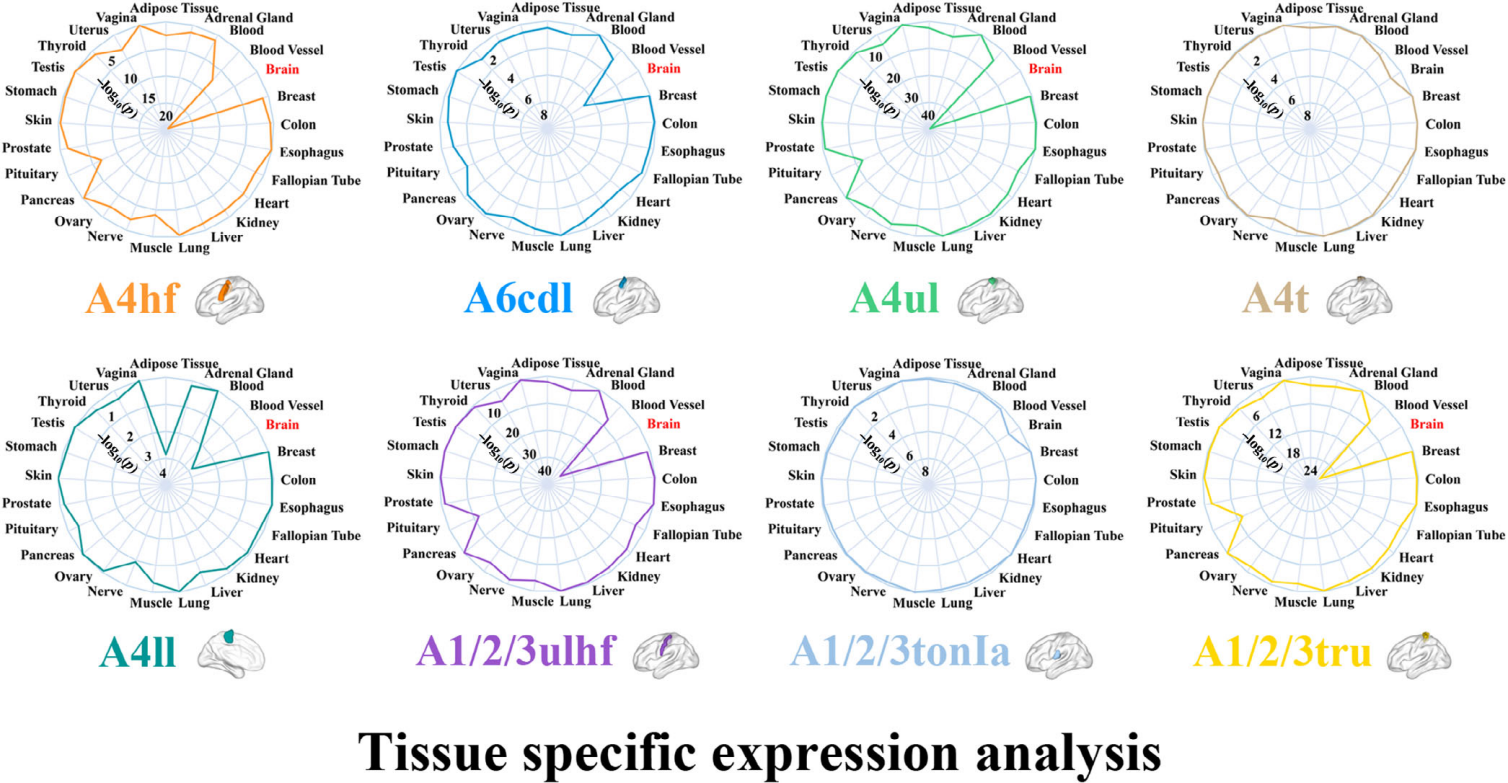
Tissue specific expression of the genes related to rsFC of sensorimotor subregions. The inner location indicates a greater −log_10_(*p*) (FDR‐BH corrected) which represents a higher statistical significance. Red fonts represent that genes were significantly specifically expressed in brain tissue. A1/2/3tonIa, tongue and larynx region of area 1/2/3; A1/2/3tru, trunk region of area 1/2/3; A1/2/3ulhf, upper limb, head, and face region of area 1/2/3; A4hf, head and face region of area 4; A4ll, lower limb region of area 4; A4t, trunk region of area 4; A4ul, upper limb region of area 4; A6cdl, caudal dorsolateral area 6; FDR‐BH, Benjamini and Hochberg method for false discovery rate; rsFC, resting‐state functional connectivity

**FIGURE 6:**
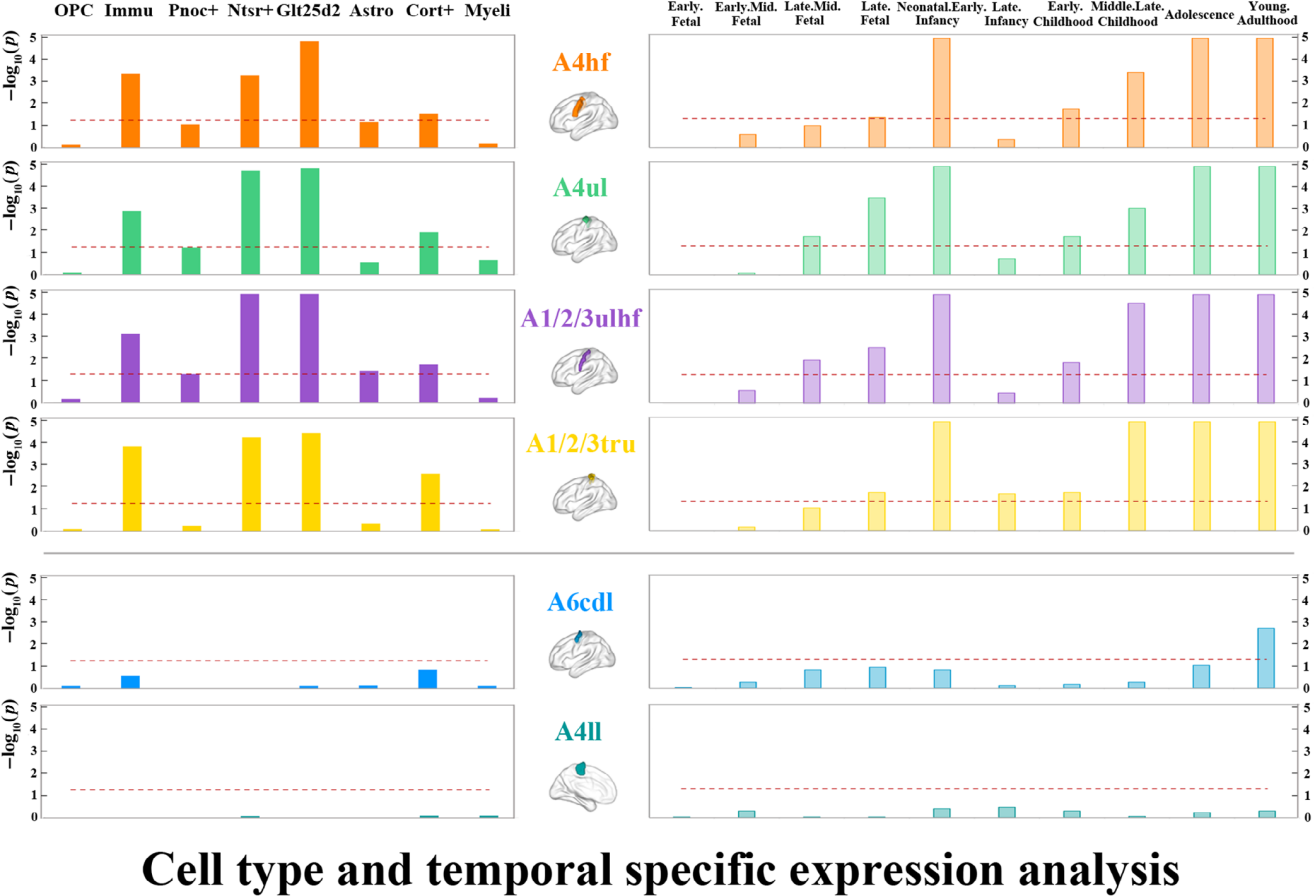
Cell type and temporal specific expression of the genes related to rsFC of sensorimotor subregions. *Y* axis is −log_10_(*p*); if −log_10_(*p*) > 5, then −log_10_(*p*) = 5. The red dashed line represents the statistical significance threshold of *p* < .05 (FDR‐BH corrected). Four subregions above the gray line were the polygenic‐modulated subregions and two subregions below the gray line were the oligogenic‐modulated subregions. A1/2/3tru, trunk region of area 1/2/3; A1/2/3ulhf, upper limb, head, and face region of area 1/2/3; A4hf, head and face region of area 4; A4ll, lower limb region of area 4; A4ul, upper limb region of area 4; A6cdl, caudal dorsolateral area 6; Astro, astrocytes; Cort+, cortistatin‐expressing interneurons; FDR‐BH, Benjamini and Hochberg method for false discovery rate; Glt25d2, corticopontine neurons; Immu, immune cells; Myeli, myelinating oligodendrocytes; Nstr+, corticothalamic neurons; OPC, oligodendrocyte progenitor cells; Pnoc+, prepronociceptin‐expressing neurons; rsFC, resting‐state functional connectivity

The genes associated with rsFC of the A4hf, A6cdl, A4ul, A4ll, A1/2/3ulhf, and A1/2/3tru presented specific expression in brain tissue (Figure 5 and Supplementary file 3, sheet “Tissues”). For gene sets that were specifically expressed in brain tissue, we further examined their specific expression in cortical cell types and developmental stages. As a result, we found that the genes related to rsFC of the A4hf, A4ul, A1/2/3ulhf, and A1/2/3tru were specifically expressed in multiple types of neurons (Ntsr+, Glt25d2, and Cort+ neurons) and immune cells, while those of the A6cdl and A4ll were not specifically expressed in any cortical cell types (Figure 6 and Supplementary file 3, sheet “Cell types”). The temporal‐specific expression analyses demonstrated that the genes related to rsFC of the A4hf, A4ul, A1/2/3ulhf, and A1/2/3tru were preferentially expressed during late fetal, neonatal and early infancy, early childhood, middle and late childhood, adolescence, and young adulthood; those of the A6cdl were preferentially expressed during young adulthood; and those of the A4ll were not preferentially expressed during any developmental stages (Figure 6 and Supplementary file 3, sheet “Developmental stages”). Based on these observations, we classified these sensorimotor subregions into two categories. Specifically, we defined two primary motor subregions (the A4hf and A4ul) and two primary somatosensory subregions (the A1/2/3ulhf and A1/2/3tru) as polygenic‐modulated subregions, whose rsFC were related to hundreds of genes with similar specific expression. The other two motor subregions (the A6cdl and A4ll) were defined as oligogenic‐modulated subregions, whose rsFC were related to dozens of genes with different specific expression from that of the polygenic‐modulated subregions.

### 3.4 Gene functional enrichment

**FIGURE 7:**
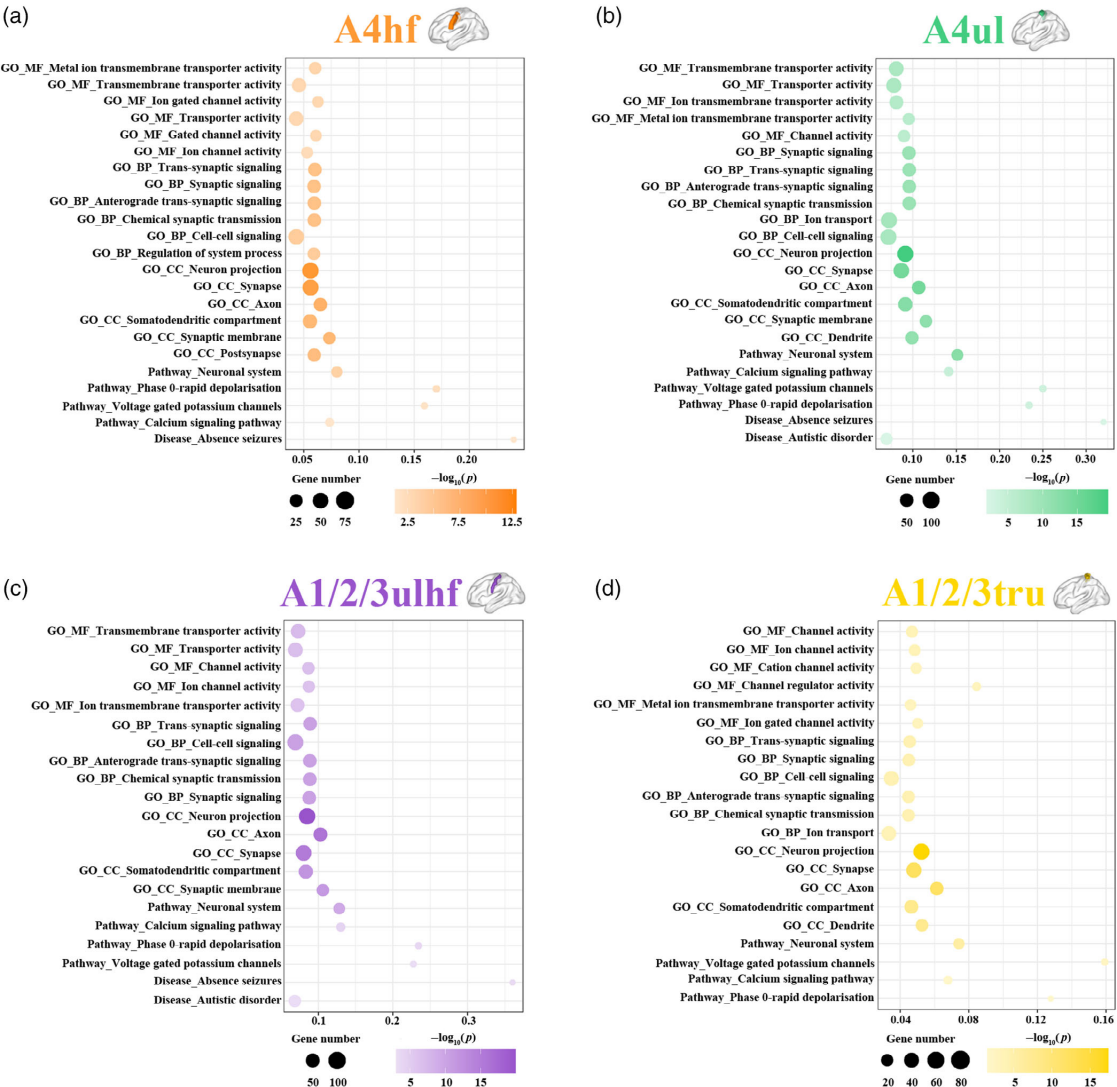
Functional enrichment of the genes related to rsFC of the polygenic‐modulated sensorimotor subregions. For each bubble chart corresponding to a sensorimotor subregion, the *x*‐axis denotes the rich factor and the *y*‐axis denotes items from the GO, pathway and disease databases. The bubble size represents the number of genes overlapping with those belonging to each item, and the bubble color represents the −log_10_(*p*) with the *p* value corrected by the FDR‐BH. The rich factor refers to the ratio of the number of rsFC‐related genes annotated to the item to the number of all genes annotated to the item. A1/2/3tru, trunk region of area 1/2/3; A4hf, head and face region of area 4; A4ul, upper limb region of area 4; A1/2/3ulhf, upper limb, head, and face region of area 1/2/3; FDR‐BH, Benjamini and Hochberg method for false discovery rate; GO, gene ontology; rsFC, resting‐state functional connectivity

We carried out functional enrichment analyses with the ToppGene portal to characterize the biological functions, pathways, and diseases of the rsFC‐related gene sets (Figure 7 and Supplementary file 4). Overall, the genes related to rsFC of the polygenic‐modulated subregions (the A4hf, A4ul, A1/2/3ulhf, and A1/2/3tru) showed similar enrichment results, which were different from those of the oligogenic‐modulated subregions (the A6cdl and A4ll). Specifically, the genes related to rsFC of the A4hf, A4ul, A1/2/3ulhf, and A1/2/3tru were enriched for MFs including transporter activity and channel activity, BPs including synaptic signaling and cell–cell signaling, and CCs including neuron projection, synapse and axon; for pathways including neuronal system, phase 0‐rapid depolarization, voltage gated potassium channels, and calcium signaling pathway; and for diseases including neuropsychiatric disorders such as absence seizures (Figure 7). The genes related to rsFC of the A6cdl were enriched for MFs including voltage‐gated calcium channel activity, BPs including neurofilament bundle assembly and neurofilament cytoskeleton organization, and CCs including neurofilament and postsynaptic intermediate filament cytoskeleton (Supplementary file 4, sheet “A6cdl”). The genes related to rsFC of the A4ll were enriched for diseases including epilepsy (Supplementary file 4, sheet “A4ll”).

### 3.5 Overlap with rsFC‐related genes in earlier studies

Fisher's exact tests revealed that rsFC‐related genes identified in the present study significantly overlapped with those reported in earlier rsFC studies (*p* < .05, FDR‐BH corrected; Tables S5 and S6), demonstrating a certain degree of rsFC specificity of our results.

### 3.6 Behavioral relevance

**FIGURE 8:**
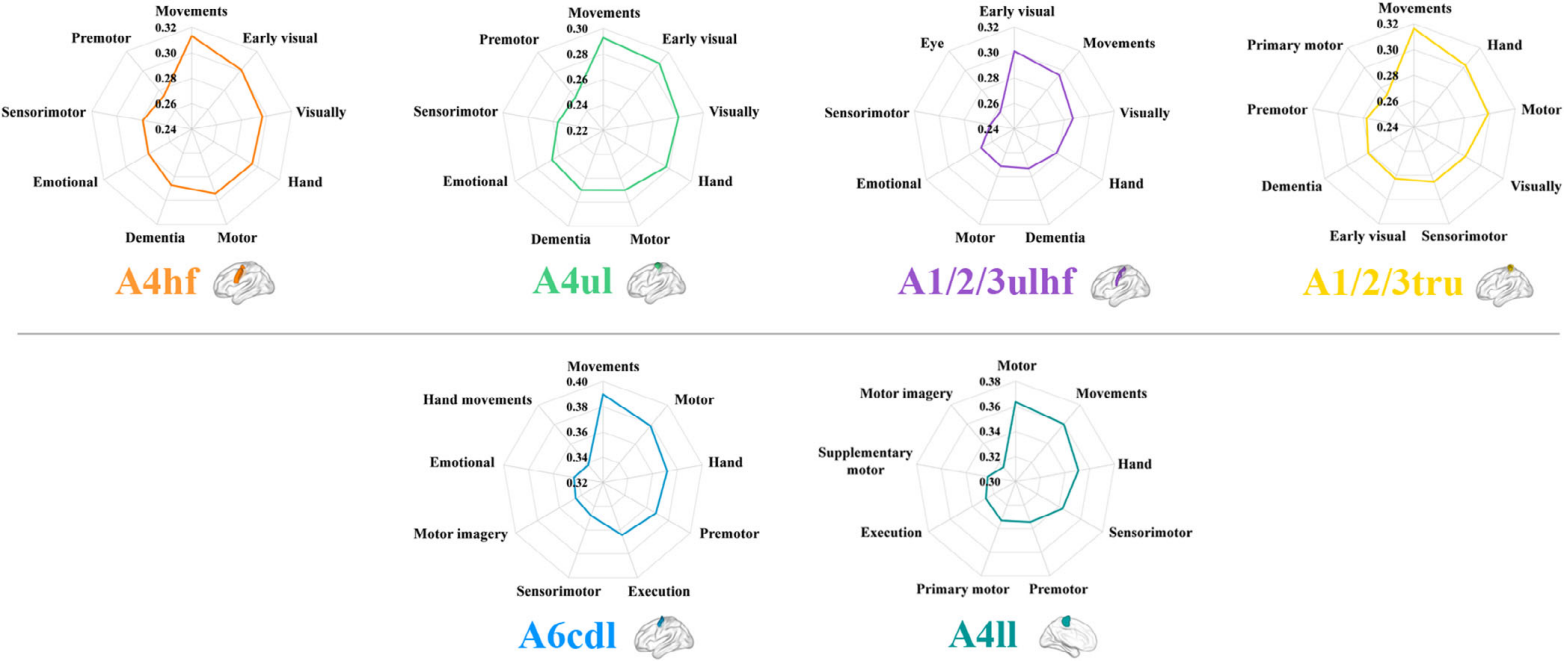
Correlations between rsFC‐related genes and behavioral terms in the Neurosynth. The coordinate values represent the mean absolute correlations between expression measures of the identified rsFC‐related genes and activation values in each behavioral term map. Four subregions above the gray line were the polygenic‐modulated subregions and two subregions below the gray line were the oligogenic‐modulated subregions. A1/2/3tru, trunk region of area 1/2/3; A1/2/3ulhf, upper limb, head and face region of area 1/2/3; A4hf, head and face region of area 4; A4ll, lower limb region of area 4; A4ul, upper limb region of area 4; A6cdl, caudal dorsolateral area 6; rsFC, resting‐state functional connectivity

By correlating gene expression with behavioral domains in the Neurosynth, we found that genes related to rsFC of sensorimotor subregions were associated with common behavioral terms including sensorimotor, movements and motor (Figure 8). Moreover, the genes related to rsFC of the polygenic‐modulated subregions (the A4hf, A4ul, A1/2/3ulhf, and A1/2/3tru) were consistently associated with additional behavioral terms including vision and dementia (Figure 8).

### 3.7 PPI networks and hub genes

**FIGURE 9:**
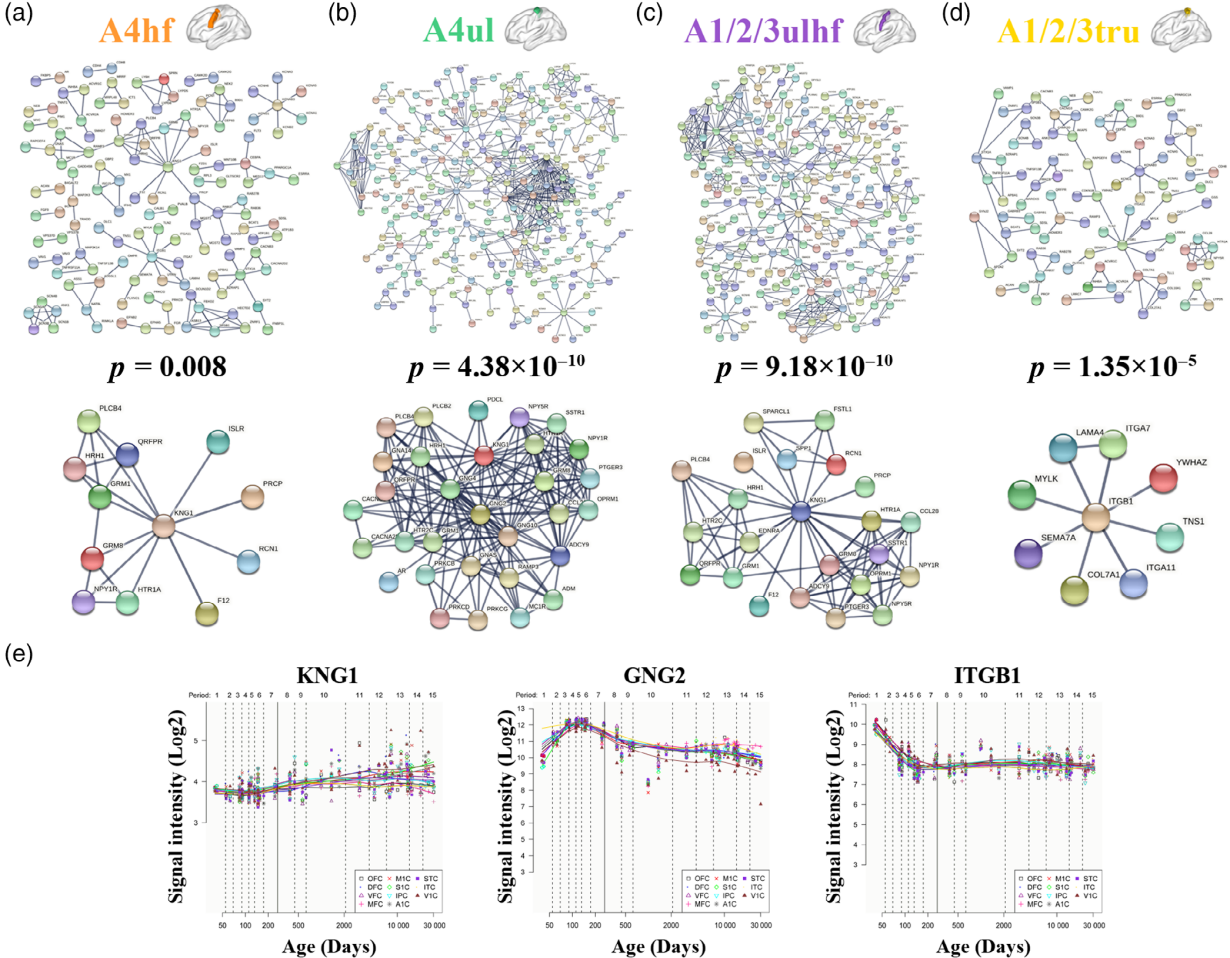
PPI networks. PPI networks with statistical significance were constructed by the genes related to rsFC of four polygenic‐modulated sensorimotor subregions including the A4hf (a), A4ul (b), A1/2/3ulhf (c), and A1/2/3tru (d). Top panel: Whole PPI networks; bottom panel: PPI subnetworks centered at the hub genes with the highest node degree. The *p* value denotes the statistical significance of how likely the proteins encoded by the input genes are connected to construct a network. Spatial–temporal specific expression curves of the hub genes with the highest node degree are shown in (e). A1/2/3tru, trunk region of area 1/2/3; A1/2/3ulhf, upper limb, head, and face region of area 1/2/3; A4hf, head and face region of area 4; A4ul, upper limb region of area 4; PPI, protein–protein interaction; rsFC, resting‐state functional connectivity

**FIGURE 10:**
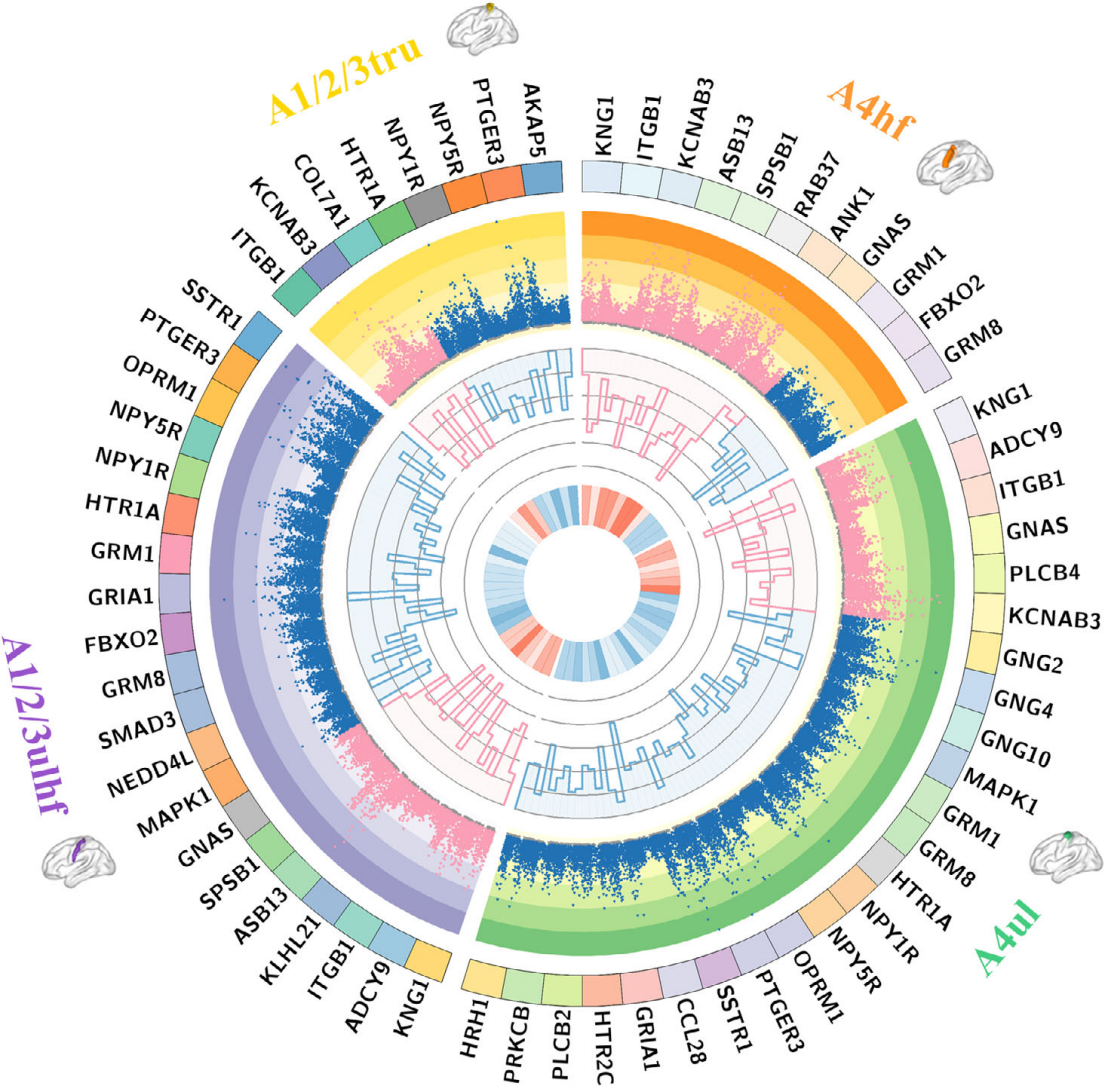
Circos map showing hub genes in the PPI networks. The orange, green, purple and yellow tracks represent 11, 24, 20, and 8 hub genes in the PPI networks constructed by the gene sets associated with four polygenic‐modulated sensorimotor subregions including the A4hf, A4ul, A1/2/3ulhf and A1/2/3tru, respectively. For each track, the outermost ring represents the symbols of the hub genes. The second circle represents −log_10_(*p*) (FDR‐BH corrected) of the transcriptome‐neuroimaging spatial correlations for each subject (a dot or triangle). The significant *p* value is shown as pink dot (positive correlation) or blue triangle (negative correlation), and the non‐significant *p* value is shown as gray. The third circle represents the percentage of subjects with consistently significant correlations in each dataset sorted clockwise as the discovery, CNP, and SALD datasets. The gray lines from the outside to inside represent the percentages of 90%, 92%, 94%, 96%, 98%, and 100%, respectively. The innermost ring shows the average correlation coefficients between gene expression and rsFC of all subjects from the three datasets, with a darker red (blue) color indicating a greater positive (negative) correlation coefficient. A1/2/3tru, trunk region of area 1/2/3; A1/2/3ulhf, upper limb, head and face region of area 1/2/3; A4hf, head and face region of area 4; A4ul, upper limb region of area 4; CNP, the Consortium for Neuropsychiatric Phenomics; PPI, protein–protein interaction; rsFC, resting‐state functional connectivity; SALD, the Southwest University Adult Lifespan Dataset

PPI analyses revealed that genes related to rsFC of the A4hf, A4ul, A1/2/3ulhf, and A1/2/3tru could construct PPI networks with statistical significance (Figure 9a–d). Genes with top 10% of the node degree in each PPI network were identified as the hub genes. There were 11, 24, 20, and 8 hub genes in the PPI networks constructed by the gene sets associated with the A4hf, A4ul, A1/2/3ulhf, and A1/2/3tru, respectively (Figure 10). We also delineated the spatial–temporal expression trajectory of three hub genes with the highest node degree (KNG1 for the A4hf and A1/2/3ulhf, GNG2 for the A4ul, and ITGB1 for the A1/2/3tru) (Figure 9e).

### 3.8 Sensitivity analysis

To evaluate the effect of different DS threshold selections, two other DS cutoff thresholds (top 40% and 60%) were used during the brain transcriptomic data processing to obtain normalized expression measures of 4010 and 6016 genes, respectively. As a result, we found considerable overlaps between the identified genes in the main analyses and those in the sensitivity analyses based on the thresholds of 40% (overlap ratio: 86.08%–97.49%) and 60% (overlap ratio: 97.42%–100%) (Tables S7 and S8 and Supplementary file 5). In addition, using BOLD data with GSR resulted in a marked reduction in the identified rsFC‐related genes, but most of the identified genes (73.33%–96.15%) were included in those found in our main analyses (Table S9 and Supplementary file 6).

## 4 DISCUSSION

By applying transcriptome‐rsFC spatial correlation analyses to brain transcriptomic and rs‐fMRI data, our design represents the first study to examine the underlying molecular substrates of rsFC profiles of sensorimotor subregions. rsFC of six sensorimotor subregions were found to associate with expression measures of six gene sets that were specifically expressed in brain tissue. Furthermore, we identified two primary motor subregions (the A4hf and A4ul) and two primary somatosensory subregions (the A1/2/3ulhf and A1/2/3tru) as polygenic‐modulated subregions, whose rsFC were related to hundreds of genes with similar functional characteristics. The other two motor subregions (the A6cdl and A4ll) were defined as oligogenic‐modulated subregions, whose rsFC were related to dozens of genes with different functional characteristics from those of the polygenic‐modulated subregions. Divergence between the genes associated with the polygenic‐ and oligogenic‐modulated subregions was present in multiple facets of the functional characteristics. First, the former were specifically expressed in multiple types of neurons and immune cells, yet the latter were not specifically expressed in any cortical cell types. Second, the former were preferentially expressed during the middle and late stages of cortical development, while the latter showed no preferential expression during any stages. Third, the former were prone to be enriched for general biological functions and pathways, but the latter for specialized biological functions and pathways. Fourth, the former were enriched for neuropsychiatric disorders, whereas this enrichment was absent for the latter. Finally, although the identified genes were commonly associated with sensorimotor behavioral processes, the polygenic‐modulated subregions associated genes were additionally related to vision and dementia. Combined, these observations corroborate our hypothesis of commonalities and differences in the identified rsFC‐related genes and their functional characteristics across sensorimotor subregions.

Prior research has demonstrated a strong contribution of the synchronized gene expression to the development of brain functional connectivity (Richiardi et al., 2015; Zhu et al., 2021). In agreement with this view, our data showed a set of genes whose transcriptional patterns were linked with the rsFC profiles of sensorimotor subregions. Furthermore, the rsFC‐related genes identified in the present study exhibited specific expression in brain tissue and significantly overlapped with those reported in earlier rsFC studies, suggesting specificity of these genes to brain functional connectivity and thereby enhancing our confidence in interpreting our results.

The somatosensory and motor cortices are traditionally considered distinct entities that are predominantly engaged by neural processes involving aspects of somatosensation and movement, respectively (Graziano, 2006; Kania, 2021). Nevertheless, we found that rsFC of two primary motor subregions (the A4hf and A4ul) and two primary somatosensory subregions (the A1/2/3ulhf and A1/2/3tru) showed similar polygenic modulation. Several factors might explain the seemingly counter‐intuitive findings. First, these polygenic‐modulated subregions are located closely around the central sulcus, increasing the difficulty in spatially disentangling these motor and somatosensory subregions with rs‐fMRI. Second, the primary motor cortex functions like a somatosensory area before it develops its motor functions, suggesting a common neurodevelopmental origin. This idea has been supported by previous findings that the primary motor cortex initially establishes a sensory framework upon which its later‐emerging role in motor control is built (Dooley & Blumberg, 2018) and that the primary motor cortex neurons receive somatosensory information before the motor map develops (Chakrabarty & Martin, 2005). Finally, intense research has provided empirical evidence for a complementary or synergistic nature of the functions of sensory and motor cortices. For example, Matyas et al. found a strong and direct role of sensory cortex in motor control (Matyas et al., 2010). Brovelli and colleagues revealed that primary somatosensory cortex and primary motor cortex were bound together in a synchronized large‐scale cortical network subserving premovement maintenance behavior (Brovelli et al., 2004). In addition, previous data offer compelling demonstrations that the somatosensory cortex receives oscillatory efference copy information from the primary motor cortex about the motor command (Witham et al., 2010) and neuroplastic changes in the coherence between motor and somatosensory areas emerge during sensorimotor learning (Arce‐McShane et al., 2016). Notably, we found that rsFC of the higher‐order motor subregions were oligogenic‐modulated (the A6cdl) or showed no significant spatial correlations with gene expression measures (the A6cvl). Given emerging evidence of higher individual variability in rsFC in higher‐order cortex and lower variability in primary cortex (Mueller et al., 2013), one possible explanation may be that highly variable rsFC of the higher‐order motor subregions might give rise to the muted gene expression‐rsFC correlations.

Our annotation analysis brought forward an important observation of the similar functional characteristics of the genes related to rsFC of the polygenic‐modulated subregions. Specifically, cell type‐specific expression analysis showed that these genes were specifically expressed in multiple types of neurons and immune cells. These findings add important context to existing literature highlighting the pivotal role of neurons in mediating the genetic effects on rsFC (Anderson et al., 2018; Richiardi et al., 2015) as well as the potential of immune cells (e.g., microglial cells) to regulate neuronal networks (Garaschuk & Verkhratsky 2019). Concurrently, temporal‐specific expression analysis revealed that the polygenic‐modulated subregions associated genes were preferentially expressed during the middle and late stages of cortical development. It is noteworthy that the brain undergoes dramatic remodeling and neuropsychiatric disorders often emerge during this period (Paus et al., 2008), coincident with the finding that these genes were enriched for neuropsychiatric disorders. These results complement and extend previous rs‐fMRI studies demonstrating an association between neuropsychiatric disorders and sensorimotor cortical rsFC (Evangelisti et al., 2018; Vollmar et al., 2011; Wang et al., 2017). In addition, the current observation of temporal spike of enrichment in early life, then lower enrichment in late infancy/early childhood followed by spike in late childhood suggests potential environmental influences. For instance, more participation in sports and extra‐curriculars during late childhood may contribute to the maturation of sensorimotor circuitry. Indeed, there is empirical evidence that epigenetic mechanisms, including DNA and histone modifications as well as expression of specific microRNAs, can be modulated by environmental factors or external stimuli (e.g., exercise) and eventually induce specific and fine‐tuned changes to the transcriptional response (Tümer et al., 2001; Widmann et al., 2019). It is generally accepted that cellular processes are associated with changes in the expression patterns of groups of genes that share common biological functions or attributes rather than single genes (Maleki et al., 2020). This is also the case for neural processes. Our functional enrichment analysis showed that the gene sets associated with rsFC of the polygenic‐modulated subregions were consistently prone to be enriched for general biological functions and pathways. For example, with respect to MFs, these genes were enriched for ion channel activity such as voltage‐gated potassium channel, sodium channel, and calcium channel. It is quite apparent that these ion channels are key mediators of neuronal excitability and are critically involved in cellular and molecular signaling pathways (Eijkelkamp et al., 2012; Kumar et al., 2016; Shah & Aizenman, 2014). In regard to BPs, these genes were enriched for synaptic signaling via which information exchanges between neurons (Biederer et al., 2017), laying the basis for rsFC. In terms of CCs, these genes were enriched for basic components of the central nervous system including neuron projection, synapse, and axon.

By contrast, the genes related to rsFC of the oligogenic‐modulated subregions (the A6cdl and A4ll) demonstrated a non‐specific pattern of functional characteristics. First, these genes were not specifically expressed in any cortical cell types and showed no preferential expression during any neurodevelopmental stages. This is likely due to the fewer genes identified. Second, these genes tended to be enriched for specialized biological functions and pathways. For instance, the genes related to rsFC of the A6cdl were enriched for voltage‐gated calcium channel activity and neurofilament. Voltage‐gated calcium channel channels are key transducers of membrane potential changes into intracellular calcium transients that initiate many physiological events (Catterall, 2011). Neurofilaments belong to the family of cytoskeletal intermediate filament proteins that give cells their shape; they determine axonal caliber, which controls signal conduction; and they regulate the transport of synaptic vesicles and modulate synaptic plasticity by binding to neurotransmitter receptors. There is mounting evidence for the role of neurofilament aggregation in neurodegeneration (Didonna & Opal, 2019). Moreover, recent reports that subunits of neurofilaments exist within postsynaptic terminal boutons and influence neurotransmission might explain how neurofilament might contribute to normal synaptic function and neuropsychiatric conditions (Yuan et al., 2017).

By correlating gene expression with behavioral domains in the Neurosynth, we found that genes related to rsFC of sensorimotor subregions were associated with common behavioral terms including sensorimotor, movements and motor, confirming the prominent sensorimotor involvement of the identified genes. Moreover, the genes related to rsFC of the polygenic‐modulated subregions were consistently associated with additional behavioral terms including vision and dementia. Although the relationship of the sensorimotor cortex with vision (Eisenberg et al., 2011; Glickstein, 2000; Hayhoe, 2017) and dementia (Boa Sorte Silva et al., 2020; Soman et al., 2020) is well established, our data may generate novel insights into such relation from the genetic perspective.

The GSR is a topic of great debate in rs‐fMRI analyses (Murphy & Fox, 2017). In the present study, we investigated genes associated with rsFC of sensorimotor subregions based on BOLD data without and with GSR, respectively. Results showed a significant reduction in the identified rsFC‐related genes based on data with GSR relative to those without GSR. Since the global signal contains both non‐neuronal confounds (e.g., physiological artifacts and head motion) and neuronal component, the application of GSR may remove not only global confounds but also physiologically relevant neuronal signals. This has led to an assumption that the loss of neuronal information may impede the identification of genes related to these neuronal activities, leading to a reduction of the rsFC‐related genes.

Our results should be interpreted in view of some limitations. First, the transcriptomic and imaging data were derived from six post‐mortem donor brains and 793 participants' living brains, respectively. This disparity may have introduced potential biases. Considering prior reports of conservative gene expression across individuals (Hawrylycz et al., 2015; Zeng et al., 2012), we set a DS threshold to focus our transcriptome‐rsFC spatial correlation analysis on genes with more conserved expression profiles to minimize the biases. However, this would miss some genes with higher transcriptional variability across individuals. Second, although the AHBA offers investigators a comprehensive, brain‐wide transcriptomic atlas, one may argue that the dataset is limited in light of the scarce donor brains. To verify and interpret our preliminary findings, animal experiments will be part of our future investigations. Third, only tissue samples in the left cerebral cortex were included in our analysis due to the fact that transcriptomic data were less in the right hemisphere and transcriptional profile differed substantially between cortical and subcortical areas. Nevertheless, the reduced tissue samples as well as hemisphere and region constrains may have influenced our results. Finally, these subregions that were not found to be significantly associated with any genes could still be oligogenic or even polygenic. More tissue samples or larger imaging samples might be required to detect these associations in the future.

In conclusion, our data represent the first report to date investigating the molecular substrates underlying rsFC of the sensorimotor cortex at the subregional level. Based on our results, the sensorimotor cortex can be conceptualized as the polygenic‐ and oligogenic‐modulated subregions, whose rsFC were related to gene sets diverging on their numbers and functional characteristics. These findings may advance our understanding of the functional homogeneity and heterogeneity of the human sensorimotor cortex from the perspective of underlying genetic architecture.

## CONFLICT OF INTEREST

The authors declare no conflict of interests.

## Supporting information


**Appendix S1** Supplementary Information

## Data Availability

The data that support the findings of this study are available on request from the corresponding author. The data are not publicly available due to privacy or ethical restrictions.
