# Supplementary material for: Transcriptional substrates underlying functional connectivity profiles of subregions within the human sensorimotor cortex
Source: Hum Brain Mapp. 2022 Jul 27;43(18):5562–78. doi: 10.1002/hbm.26031 (PMC9704778; doi:10.1002/hbm.26031)
Supplement: Supplementary file 1 — Appendix S1 Supplementary Information [file HBM-43-5562-s001.zip › HBM_26031_Supplementary file 2.pdf]

Genes related to rsFC of the L-A4hf in both the discovery and validation experiments

GeneID	Genesymbol	Discovery dataset						CNP dataset						SALD dataset					
		r					Percentage	*r*					Percentage	*r*					Percentage
		Min	Med	Max	Mean	SD		Min	Med	Max	Mean	SD		Min	Med	Max	Mean	SD	
92	ACVR2A	-0.31	-0.15	0.01	-0.15	0.06	0.93	-0.32	-0.15	-0.06	-0.16	0.05	0.99	-0.30	-0.17	0.01	-0.17	0.05	0.97
133	ADM	-0.08	0.16	0.38	0.17	0.07	0.94	0.03	0.16	0.30	0.16	0.06	0.95	-0.01	0.17	0.34	0.17	0.07	0.92
176	ACAN	-0.04	0.23	0.48	0.24	0.09	0.97	0.08	0.24	0.45	0.25	0.08	1.00	0.01	0.26	0.47	0.26	0.09	0.99
204	AK2	-0.04	0.14	0.29	0.14	0.05	0.91	0.04	0.14	0.25	0.14	0.05	0.95	0.00	0.16	0.27	0.16	0.05	0.95
286	ANK1	-0.07	0.21	0.47	0.21	0.09	0.96	0.07	0.22	0.45	0.23	0.08	0.99	0.00	0.22	0.45	0.23	0.08	0.98
320	APBA1	-0.22	-0.13	0.03	-0.13	0.04	0.92	-0.28	-0.14	-0.05	-0.15	0.04	0.99	-0.24	-0.14	-0.02	-0.15	0.04	0.97
367	AR	-0.03	0.19	0.43	0.19	0.07	0.96	0.08	0.19	0.39	0.20	0.06	1.00	0.05	0.21	0.39	0.21	0.07	0.98
430	ASCL2	-0.44	-0.20	0.07	-0.20	0.08	0.95	-0.42	-0.21	-0.06	-0.22	0.08	0.99	-0.41	-0.22	0.01	-0.22	0.08	0.96
445	ASS1	-0.36	-0.19	0.09	-0.19	0.07	0.96	-0.34	-0.21	-0.03	-0.20	0.06	0.98	-0.39	-0.21	-0.03	-0.21	0.07	0.98
460	ASTN1	-0.32	-0.14	0.04	-0.14	0.06	0.90	-0.32	-0.15	-0.06	-0.16	0.05	0.98	-0.33	-0.16	0.03	-0.16	0.06	0.95
481	ATP1B1	-0.03	0.16	0.37	0.16	0.07	0.92	0.05	0.17	0.32	0.18	0.05	0.98	0.03	0.18	0.35	0.18	0.06	0.96
483	ATP1B3	-0.03	0.14	0.32	0.14	0.06	0.91	0.05	0.15	0.30	0.15	0.05	0.95	-0.02	0.15	0.30	0.15	0.06	0.91
493	ATP2B4	-0.42	-0.17	0.13	-0.18	0.09	0.90	-0.41	-0.19	-0.02	-0.19	0.08	0.94	-0.41	-0.20	0.00	-0.20	0.08	0.94
563	AZGP1	-0.09	0.14	0.26	0.14	0.05	0.93	0.04	0.15	0.25	0.15	0.05	0.97	0.04	0.16	0.28	0.16	0.05	0.98
586	BCAT1	-0.05	0.15	0.34	0.15	0.06	0.92	0.04	0.14	0.32	0.15	0.05	0.95	-0.04	0.16	0.32	0.16	0.06	0.93
613	BCR	-0.34	-0.15	0.09	-0.15	0.07	0.92	-0.31	-0.17	-0.04	-0.17	0.06	0.98	-0.32	-0.18	-0.03	-0.17	0.06	0.95
784	CACNB3	-0.34	-0.15	0.02	-0.14	0.05	0.92	-0.26	-0.14	-0.01	-0.14	0.05	0.94	-0.29	-0.15	0.02	-0.15	0.06	0.93
793	CALB1	-0.42	-0.20	0.04	-0.20	0.07	0.95	-0.35	-0.19	0.01	-0.20	0.07	0.99	-0.38	-0.21	0.03	-0.21	0.08	0.97
814	CAMK4	-0.31	-0.17	0.07	-0.17	0.06	0.95	-0.29	-0.17	-0.05	-0.16	0.05	0.96	-0.31	-0.17	0.01	-0.17	0.06	0.95
817	CAMK2D	-0.42	-0.21	0.06	-0.21	0.08	0.96	-0.42	-0.21	-0.08	-0.22	0.07	1.00	-0.42	-0.24	-0.01	-0.23	0.08	0.98
818	CAMK2G	-0.05	0.17	0.40	0.18	0.08	0.93	0.06	0.19	0.36	0.19	0.07	0.96	-0.01	0.19	0.38	0.19	0.07	0.96
869	CBLN1	-0.28	-0.14	0.05	-0.14	0.05	0.91	-0.23	-0.14	-0.01	-0.14	0.05	0.90	-0.28	-0.15	0.02	-0.15	0.06	0.92
1002	CDH4	-0.33	-0.15	0.06	-0.15	0.07	0.91	-0.30	-0.17	-0.06	-0.17	0.06	0.97	-0.32	-0.17	0.00	-0.17	0.06	0.95
1006	CDH8	-0.39	-0.21	0.06	-0.21	0.07	0.97	-0.36	-0.22	-0.09	-0.23	0.06	1.00	-0.38	-0.23	-0.02	-0.22	0.07	0.99
1050	CEBPA	-0.38	-0.17	0.10	-0.17	0.08	0.92	-0.36	-0.19	-0.07	-0.20	0.07	0.98	-0.39	-0.20	-0.01	-0.20	0.07	0.97
1272	CNTN1	-0.36	-0.16	0.09	-0.16	0.07	0.92	-0.33	-0.17	-0.04	-0.17	0.06	0.97	-0.36	-0.18	-0.02	-0.18	0.07	0.94
1300	COL10A1	-0.28	-0.16	0.06	-0.15	0.06	0.92	-0.27	-0.16	-0.03	-0.16	0.05	0.99	-0.28	-0.16	0.03	-0.16	0.05	0.95
1381	CRABP1	-0.07	0.19	0.40	0.19	0.07	0.96	0.05	0.18	0.33	0.18	0.07	0.95	0.03	0.20	0.39	0.20	0.08	0.96
1501	CTNND2	-0.36	-0.17	0.07	-0.17	0.07	0.94	-0.29	-0.17	-0.04	-0.17	0.05	0.98	-0.33	-0.18	-0.02	-0.18	0.07	0.95
1522	CTSZ	-0.05	0.18	0.36	0.18	0.07	0.94	0.05	0.17	0.36	0.17	0.06	0.97	-0.03	0.19	0.38	0.19	0.07	0.97
1607	DGKB	-0.35	-0.18	0.08	-0.18	0.07	0.96	-0.31	-0.17	-0.03	-0.17	0.06	0.93	-0.36	-0.20	0.03	-0.19	0.07	0.96
1730	DIAPH2	-0.31	-0.15	0.03	-0.15	0.06	0.92	-0.27	-0.15	-0.06	-0.15	0.05	0.95	-0.31	-0.16	-0.03	-0.16	0.05	0.96
1809	DPYSL3	-0.48	-0.18	0.08	-0.18	0.09	0.92	-0.38	-0.19	-0.03	-0.19	0.08	0.94	-0.41	-0.20	-0.01	-0.20	0.09	0.95
1893	ECM1	-0.04	0.18	0.40	0.18	0.08	0.94	0.06	0.19	0.39	0.20	0.06	0.99	-0.05	0.19	0.36	0.19	0.07	0.96
1948	EFNB2	-0.34	-0.16	0.08	-0.16	0.07	0.91	-0.32	-0.17	-0.04	-0.17	0.07	0.93	-0.36	-0.17	0.02	-0.18	0.07	0.94
2027	ENO3	-0.01	0.13	0.24	0.13	0.04	0.90	0.03	0.13	0.24	0.13	0.04	0.94	-0.01	0.13	0.25	0.13	0.05	0.93
2044	EPHA5	-0.30	-0.13	0.06	-0.13	0.05	0.91	-0.30	-0.14	-0.04	-0.14	0.05	0.96	-0.28	-0.15	0.00	-0.14	0.05	0.93
2070	EYA4	-0.07	0.17	0.40	0.17	0.07	0.93	0.07	0.19	0.36	0.19	0.06	0.97	-0.01	0.19	0.35	0.19	0.07	0.96
2101	ESRRA	-0.04	0.20	0.44	0.20	0.08	0.96	0.07	0.21	0.42	0.21	0.07	0.99	0.00	0.22	0.42	0.22	0.08	0.98
2104	ESRRG	-0.04	0.22	0.47	0.22	0.09	0.96	0.09	0.23	0.46	0.24	0.08	1.00	0.01	0.24	0.46	0.24	0.09	0.98
2155	F7	-0.05	0.13	0.29	0.13	0.05	0.91	0.03	0.12	0.22	0.12	0.04	0.95	-0.02	0.12	0.24	0.13	0.05	0.90
2161	F12	-0.43	-0.19	0.06	-0.19	0.08	0.95	-0.38	-0.21	-0.08	-0.21	0.07	1.00	-0.41	-0.22	-0.05	-0.22	0.07	0.99
2173	FABP7	-0.45	-0.18	0.06	-0.19	0.08	0.93	-0.37	-0.19	-0.06	-0.20	0.07	0.97	-0.41	-0.20	0.03	-0.20	0.08	0.95
2254	FGF9	-0.06	0.18	0.43	0.18	0.08	0.93	0.05	0.19	0.40	0.20	0.07	0.98	-0.02	0.19	0.40	0.20	0.08	0.94
2268	FGR	-0.06	0.12	0.25	0.12	0.05	0.90	0.05	0.13	0.26	0.14	0.04	0.98	0.01	0.15	0.25	0.15	0.04	0.96
2289	FKBP5	-0.08	0.16	0.38	0.16	0.07	0.91	0.00	0.16	0.31	0.16	0.07	0.93	-0.02	0.19	0.34	0.18	0.07	0.94
2322	FLT3	-0.04	0.16	0.34	0.16	0.07	0.94	0.04	0.16	0.35	0.17	0.06	0.98	0.00	0.17	0.33	0.17	0.06	0.95
2560	GABRB1	-0.43	-0.19	0.08	-0.19	0.08	0.96	-0.36	-0.20	-0.06	-0.20	0.07	0.99	-0.40	-0.21	-0.04	-0.21	0.08	0.97
2565	GABRG1	-0.35	-0.17	0.03	-0.17	0.06	0.94	-0.27	-0.17	-0.07	-0.17	0.05	1.00	-0.35	-0.19	-0.04	-0.18	0.06	0.98
2620	GAS2	-0.08	0.15	0.36	0.15	0.07	0.91	0.04	0.15	0.32	0.16	0.06	0.95	-0.02	0.17	0.33	0.17	0.07	0.93
2632	GBE1	-0.03	0.14	0.33	0.14	0.06	0.91	0.03	0.14	0.28	0.14	0.05	0.96	0.00	0.16	0.31	0.16	0.05	0.95
2634	GBP2	-0.05	0.15	0.25	0.14	0.05	0.94	0.06	0.15	0.23	0.15	0.04	0.98	0.00	0.15	0.25	0.15	0.04	0.98
2742	GLRA2	-0.41	-0.18	0.07	-0.18	0.08	0.93	-0.39	-0.19	-0.04	-0.20	0.07	0.97	-0.40	-0.21	0.02	-0.21	0.08	0.96
2766	GMPR	-0.04	0.16	0.33	0.16	0.06	0.94	0.03	0.15	0.27	0.15	0.05	0.95	-0.01	0.17	0.29	0.16	0.06	0.96
2778	GNAS	-0.03	0.14	0.29	0.14	0.06	0.91	0.04	0.14	0.29	0.14	0.05	0.91	-0.02	0.15	0.28	0.15	0.06	0.91
2823	GPM6A	-0.33	-0.16	0.04	-0.16	0.06	0.94	-0.29	-0.16	-0.06	-0.16	0.05	0.99	-0.32	-0.19	0.00	-0.18	0.06	0.97
2830	GPR6	-0.33	-0.18	0.00	-0.17	0.06	0.95	-0.33	-0.19	-0.06	-0.19	0.05	0.99	-0.34	-0.20	0.00	-0.20	0.06	0.98
2888	GRB14	-0.33	-0.17	0.08	-0.17	0.07	0.92	-0.31	-0.19	0.00	-0.18	0.07	0.96	-0.34	-0.17	0.01	-0.18	0.07	0.93
2898	GRIK2	-0.36	-0.15	0.06	-0.16	0.07	0.92	-0.26	-0.14	-0.01	-0.15	0.06	0.92	-0.32	-0.17	0.03	-0.17	0.07	0.94
2911	GRM1	-0.35	-0.16	0.09	-0.16	0.07	0.93	-0.31	-0.16	-0.04	-0.17	0.06	0.93	-0.34	-0.19	-0.01	-0.18	0.07	0.95
2918	GRM8	-0.32	-0.17	0.05	-0.16	0.06	0.96	-0.30	-0.17	-0.06	-0.16	0.05	0.97	-0.34	-0.19	-0.03	-0.19	0.05	0.98
2952	GSTT1	-0.04	0.21	0.44	0.21	0.08	0.96	0.08	0.21	0.42	0.22	0.07	1.00	-0.04	0.23	0.42	0.23	0.08	0.98
3067	HDC	-0.37	-0.18	0.05	-0.18	0.07	0.94	-0.30	-0.17	-0.04	-0.18	0.06	0.97	-0.33	-0.18	0.01	-0.19	0.07	0.95
3231	HOXD1	-0.01	0.11	0.21	0.11	0.04	0.91	0.03	0.13	0.20	0.13	0.04	0.97	0.03	0.14	0.24	0.13	0.04	0.95
3269	HRH1	-0.36	-0.17	0.07	-0.17	0.07	0.94	-0.31	-0.17	-0.05	-0.17	0.07	0.92	-0.37	-0.18	-0.02	-0.19	0.07	0.95
3290	HSD11B1	-0.05	0.16	0.33	0.17	0.06	0.94	0.08	0.20	0.31	0.19	0.06	1.00	-0.03	0.20	0.35	0.20	0.06	0.98
3316	HSPB2	-0.04	0.17	0.35	0.17	0.06	0.94	0.06	0.16	0.31	0.16	0.05	0.99	0.04	0.19	0.34	0.18	0.06	0.97
3350	HTR1A	-0.37	-0.17	0.08	-0.16	0.07	0.92	-0.36	-0.17	0.01	-0.17	0.07	0.98	-0.37	-0.18	-0.01	-0.18	0.07	0.93
3396	MRPL58	-0.08	0.15	0.34	0.15	0.06	0.93	0.03	0.14	0.28	0.14	0.05	0.94	0.00	0.16	0.32	0.16	0.06	0.94
3485	IGFBP2	-0.06	0.15	0.41	0.16	0.07	0.90	0.04	0.17	0.32	0.17	0.06	0.96	0.01	0.18	0.36	0.18	0.07	0.94
3595	IL12RB2	-0.26	-0.13	0.04	-0.13	0.05	0.90	-0.23	-0.13	-0.03	-0.13	0.05	0.92	-0.26	-0.15	-0.02	-0.15	0.05	0.96
3624	INHBA	-0.01	0.16	0.31	0.16	0.05	0.96	0.03	0.18	0.30	0.17	0.05	0.98	0.06	0.19	0.33	0.19	0.05	0.98
3632	INPP5A	-0.04	0.17	0.33	0.16	0.06	0.95	0.03	0.16	0.31	0.17	0.05	0.98	0.00	0.18	0.33	0.17	0.06	0.96
3653	IPW	-0.08	0.16	0.38	0.17	0.08	0.91	0.03	0.17	0.39	0.18	0.07	0.96	-0.02	0.18	0.37	0.19	0.08	0.94
3671	ISLR	-0.05	0.15	0.32	0.15	0.06	0.91	0.02	0.17	0.29	0.17	0.05	0.96	-0.03	0.15	0.28	0.15	0.06	0.92
3679	ITGA7	-0.01	0.14	0.26	0.14	0.05	0.92	0.06	0.15	0.25	0.15	0.04	0.98	0.05	0.16	0.27	0.16	0.05	0.98
3688	ITGB1	-0.03	0.13	0.25	0.13	0.04	0.93	0.02	0.15	0.24	0.14	0.04	0.95	0.04	0.15	0.26	0.15	0.04	0.96
3736	KCNA1	-0.05	0.19	0.44	0.19	0.08	0.93	0.06	0.19	0.41	0.20	0.07	0.99	-0.05	0.22	0.41	0.21	0.08	0.97
3738	KCNA3	-0.43	-0.18	0.08	-0.18	0.08	0.93	-0.35	-0.18	-0.02	-0.19	0.08	0.95	-0.42	-0.19	0.04	-0.20	0.09	0.95
3741	KCNA5	-0.43	-0.17	0.09	-0.18	0.08	0.93	-0.36	-0.20	0.00	-0.19	0.08	0.94	-0.40	-0.19	0.05	-0.19	0.09	0.93
3755	KCNG1	-0.45	-0.21	0.03	-0.21	0.09	0.95	-0.45	-0.22	-0.07	-0.23	0.08	0.99	-0.42	-0.22	0.02	-0.23	0.09	0.98
3782	KCNN3	-0.37	-0.16	0.06	-0.16	0.07	0.92	-0.32	-0.18	-0.05	-0.18	0.06	0.95	-0.33	-0.18	-0.03	-0.18	0.07	0.95
3783	KCNN4	-0.06	0.18	0.32	0.18	0.06	0.96	0.04	0.17	0.28	0.17	0.05	0.98	-0.03	0.17	0.32	0.17	0.06	0.96
3827	KNG1	-0.05	0.16	0.37	0.16	0.07	0.92	0.04	0.17	0.32	0.17	0.06	0.97	0.00	0.19	0.38	0.19	0.07	0.96
3902	LAG3	-0.11	0.20	0.48	0.20	0.09	0.94	0.06	0.22	0.41	0.22	0.08	0.98	0.02	0.22	0.43	0.22	0.09	0.97
3910	LAMA4	-0.05	0.19	0.37	0.19	0.07	0.96	0.07	0.19	0.34	0.20	0.06	0.99	0.00	0.20	0.35	0.20	0.07	0.97
4062	LY6H	-0.45	-0.20	0.10	-0.21	0.09	0.96	-0.42	-0.21	-0.07	-0.22	0.08	0.99	-0.44	-0.23	-0.03	-0.23	0.09	0.98
4082	MARCKS	-0.38	-0.16	0.05	-0.16	0.07	0.91	-0.35	-0.18	-0.05	-0.18	0.06	0.97	-0.36	-0.20	0.02	-0.20	0.07	0.96
4092	SMAD7	-0.12	0.14	0.31	0.14	0.06	0.91	0.03	0.17	0.29	0.17	0.05	0.96	-0.01	0.17	0.34	0.17	0.06	0.95
4157	MC1R	-0.11	0.16	0.39	0.17	0.07	0.92	0.04	0.18	0.34	0.18	0.07	0.95	0.01	0.18	0.36	0.18	0.07	0.95
4257	MGST1	-0.38	-0.16	0.11	-0.17	0.08	0.92	-0.35	-0.18	-0.05	-0.18	0.07	0.96	-0.36	-0.18	0.02	-0.18	0.08	0.93
4258	MGST2	-0.04	0.22	0.42	0.22	0.08	0.96	0.07	0.22	0.40	0.23	0.07	0.99	0.02	0.24	0.42	0.24	0.07	0.99
4286	MITF	-0.04	0.17	0.32	0.17	0.06	0.96	0.07	0.17	0.28	0.18	0.05	1.00	0.03	0.18	0.32	0.19	0.06	0.97
4324	MMP15	-0.06	0.16	0.32	0.16	0.06	0.93	0.05	0.17	0.30	0.17	0.06	0.98	0.04	0.18	0.36	0.18	0.06	0.97
4354	MPP1	-0.03	0.18	0.42	0.18	0.08	0.94	0.07	0.19	0.38	0.20	0.06	0.99	0.00	0.20	0.39	0.21	0.08	0.96
4599	MX1	-0.03	0.17	0.40	0.18	0.07	0.92	0.03	0.19	0.39	0.19	0.07	0.99	0.00	0.20	0.38	0.20	0.07	0.97
4609	MYC	-0.03	0.19	0.37	0.19	0.07	0.96	0.07	0.19	0.37	0.20	0.06	0.99	-0.01	0.20	0.35	0.20	0.07	0.96
4616	GADD45B	-0.03	0.15	0.32	0.15	0.05	0.93	0.06	0.15	0.31	0.15	0.04	0.98	0.03	0.17	0.29	0.16	0.05	0.95
4638	MYLK	-0.01	0.15	0.25	0.15	0.04	0.94	0.05	0.15	0.25	0.15	0.04	0.98	0.05	0.17	0.27	0.17	0.04	0.98
4703	NEB	-0.06	0.22	0.41	0.22	0.08	0.98	0.08	0.23	0.40	0.23	0.07	1.00	0.03	0.23	0.43	0.23	0.08	0.98
4715	NDUFB9	-0.05	0.16	0.32	0.17	0.06	0.94	0.01	0.15	0.29	0.15	0.06	0.92	-0.01	0.16	0.32	0.16	0.06	0.92
4744	NEFH	-0.05	0.18	0.42	0.18	0.08	0.93	0.05	0.19	0.39	0.19	0.06	0.99	-0.05	0.20	0.40	0.20	0.08	0.96
4751	NEK2	-0.43	-0.20	0.11	-0.20	0.08	0.96	-0.36	-0.21	-0.05	-0.21	0.07	0.99	-0.40	-0.21	-0.02	-0.22	0.08	0.97
4856	NOV	-0.45	-0.19	0.09	-0.20	0.08	0.94	-0.39	-0.21	-0.05	-0.21	0.08	0.95	-0.43	-0.23	-0.03	-0.23	0.08	0.98
4885	NPTX2	-0.31	-0.15	0.09	-0.15	0.06	0.92	-0.31	-0.16	-0.01	-0.16	0.06	0.93	-0.34	-0.17	0.05	-0.16	0.06	0.93
4886	NPY1R	-0.40	-0.18	0.07	-0.17	0.08	0.92	-0.35	-0.17	-0.03	-0.17	0.07	0.91	-0.35	-0.18	0.06	-0.18	0.08	0.93
5090	PBX3	-0.03	0.14	0.27	0.13	0.05	0.92	0.04	0.13	0.25	0.13	0.05	0.90	-0.02	0.14	0.27	0.14	0.05	0.94
5116	PCNT	-0.07	0.17	0.39	0.18	0.07	0.93	0.05	0.20	0.36	0.20	0.07	0.99	0.03	0.20	0.39	0.20	0.07	0.95
5121	PCP4	-0.05	0.20	0.42	0.20	0.07	0.97	0.09	0.20	0.36	0.20	0.06	1.00	0.04	0.22	0.40	0.22	0.07	0.99
5141	PDE4A	-0.08	0.20	0.43	0.20	0.08	0.96	0.06	0.21	0.40	0.21	0.07	0.99	-0.01	0.21	0.43	0.21	0.08	0.95
5176	SERPINF1	-0.33	-0.19	0.04	-0.19	0.06	0.96	-0.30	-0.20	-0.02	-0.20	0.06	0.99	-0.35	-0.21	0.02	-0.20	0.06	0.98
5184	PEPD	-0.05	0.14	0.35	0.14	0.06	0.92	0.06	0.15	0.28	0.15	0.05	0.97	0.00	0.17	0.29	0.17	0.05	0.96
5272	SERPINB9	-0.07	0.16	0.39	0.17	0.07	0.91	0.04	0.16	0.35	0.17	0.06	0.96	-0.01	0.18	0.35	0.18	0.07	0.94
5292	PIM1	-0.06	0.16	0.37	0.17	0.07	0.93	0.04	0.18	0.34	0.18	0.06	0.98	0.01	0.18	0.35	0.18	0.07	0.95
5332	PLCB4	-0.07	0.16	0.39	0.16	0.07	0.91	0.05	0.17	0.32	0.17	0.06	0.97	0.01	0.19	0.36	0.19	0.06	0.97
5409	PNMT	-0.44	-0.19	0.03	-0.20	0.08	0.95	-0.42	-0.20	-0.07	-0.21	0.07	0.98	-0.42	-0.22	0.04	-0.22	0.08	0.96
5412	UBL3	-0.04	0.17	0.36	0.17	0.06	0.95	0.04	0.17	0.31	0.17	0.06	0.97	0.03	0.18	0.34	0.18	0.06	0.96
5467	PPARD	-0.09	0.15	0.35	0.16	0.07	0.91	0.03	0.16	0.31	0.16	0.07	0.93	0.02	0.18	0.38	0.18	0.07	0.96
5547	PRCP	-0.06	0.14	0.29	0.14	0.05	0.91	0.02	0.13	0.28	0.14	0.05	0.95	-0.03	0.15	0.28	0.15	0.05	0.95
5569	PKIA	-0.45	-0.19	0.11	-0.19	0.09	0.94	-0.40	-0.21	-0.07	-0.21	0.08	0.99	-0.44	-0.22	-0.02	-0.23	0.08	0.97
5580	PRKCD	-0.47	-0.20	0.13	-0.20	0.09	0.95	-0.44	-0.22	-0.07	-0.22	0.08	1.00	-0.43	-0.23	-0.02	-0.23	0.09	0.97
5582	PRKCG	-0.42	-0.18	0.12	-0.18	0.08	0.92	-0.39	-0.19	-0.06	-0.20	0.08	0.97	-0.38	-0.20	-0.02	-0.20	0.08	0.95
5606	MAP2K3	-0.06	0.15	0.35	0.15	0.06	0.91	0.02	0.18	0.30	0.17	0.06	0.96	0.02	0.18	0.33	0.17	0.06	0.94
5774	PTPN3	-0.29	-0.15	0.04	-0.15	0.05	0.92	-0.25	-0.14	-0.02	-0.15	0.05	0.92	-0.28	-0.15	-0.02	-0.15	0.05	0.95
5792	PTPRF	-0.39	-0.17	0.07	-0.17	0.08	0.93	-0.30	-0.16	-0.03	-0.17	0.07	0.94	-0.36	-0.17	-0.01	-0.18	0.08	0.93
5794	PTPRH	-0.01	0.13	0.23	0.13	0.04	0.92	0.05	0.15	0.23	0.15	0.04	0.97	0.02	0.15	0.25	0.15	0.04	0.96
5800	PTPRO	-0.23	-0.12	0.02	-0.12	0.04	0.91	-0.21	-0.13	-0.05	-0.13	0.04	0.96	-0.23	-0.14	-0.05	-0.14	0.04	0.97
5816	PVALB	-0.04	0.18	0.43	0.18	0.08	0.90	0.04	0.19	0.39	0.19	0.07	0.97	-0.01	0.20	0.39	0.20	0.08	0.95
5860	QDPR	-0.01	0.12	0.21	0.12	0.04	0.92	0.03	0.13	0.23	0.13	0.04	0.96	0.03	0.14	0.24	0.14	0.04	0.95
5874	RAB27B	-0.41	-0.19	0.10	-0.19	0.08	0.94	-0.39	-0.21	-0.07	-0.21	0.07	1.00	-0.41	-0.22	-0.04	-0.22	0.08	0.98
5912	RAP2B	-0.40	-0.16	0.11	-0.16	0.08	0.90	-0.36	-0.18	-0.04	-0.18	0.07	0.96	-0.38	-0.19	0.00	-0.19	0.08	0.94
5937	RBMS1	-0.05	0.17	0.40	0.17	0.08	0.93	0.02	0.20	0.36	0.20	0.07	0.99	0.02	0.20	0.39	0.20	0.07	0.96
5950	RBP4	-0.48	-0.19	0.12	-0.20	0.09	0.93	-0.41	-0.22	-0.06	-0.21	0.08	0.96	-0.43	-0.21	0.01	-0.22	0.09	0.95
5954	RCN1	-0.33	-0.16	0.07	-0.16	0.07	0.93	-0.28	-0.17	0.01	-0.17	0.06	0.92	-0.33	-0.17	0.01	-0.17	0.07	0.92
5979	RET	-0.05	0.17	0.40	0.17	0.08	0.93	0.04	0.18	0.38	0.18	0.07	0.98	0.00	0.18	0.39	0.19	0.07	0.96
5993	RFX5	-0.07	0.17	0.38	0.17	0.07	0.93	0.04	0.17	0.36	0.18	0.06	0.98	-0.01	0.19	0.37	0.19	0.07	0.95
6017	RLBP1	-0.31	-0.16	0.03	-0.16	0.05	0.95	-0.23	-0.16	-0.06	-0.16	0.04	0.99	-0.29	-0.16	-0.04	-0.16	0.05	0.95
6122	RPL3	-0.05	0.14	0.28	0.14	0.06	0.90	0.05	0.14	0.24	0.14	0.05	0.91	-0.01	0.15	0.27	0.15	0.06	0.92

6272	SORT1	-0.05	0.14	0.29	0.14	0.06	0.92	0.06	0.16	0.28	0.17	0.05	0.99	0.03	0.17	0.30	0.17	0.05	0.96
6323	SCN1A	-0.09	0.17	0.44	0.17	0.09	0.91	0.04	0.19	0.39	0.19	0.08	0.96	-0.02	0.20	0.42	0.20	0.08	0.95
6324	SCN1B	-0.06	0.19	0.46	0.19	0.09	0.92	0.04	0.21	0.41	0.21	0.08	0.98	-0.04	0.21	0.42	0.22	0.09	0.96
6330	SCN4B	-0.05	0.24	0.48	0.24	0.08	0.97	0.11	0.23	0.46	0.24	0.07	1.00	0.00	0.26	0.46	0.26	0.08	0.99
6451	SH3BGRL	-0.05	0.15	0.36	0.15	0.06	0.91	0.03	0.14	0.31	0.15	0.06	0.92	0.00	0.17	0.34	0.17	0.06	0.95
6533	SLC6A6	-0.06	0.16	0.34	0.16	0.06	0.92	0.07	0.17	0.30	0.17	0.05	1.00	0.03	0.18	0.32	0.18	0.06	0.96
6543	SLC8A2	-0.28	-0.15	0.04	-0.14	0.06	0.90	-0.28	-0.13	-0.02	-0.14	0.05	0.90	-0.29	-0.15	0.02	-0.15	0.06	0.94
6646	SOAT1	-0.07	0.16	0.36	0.16	0.07	0.92	0.02	0.17	0.34	0.18	0.06	0.99	0.00	0.18	0.34	0.18	0.06	0.95
6804	STX1A	-0.35	-0.19	0.04	-0.18	0.07	0.95	-0.35	-0.17	-0.06	-0.18	0.06	0.98	-0.34	-0.20	-0.01	-0.20	0.07	0.98
6843	VAMP1	-0.08	0.19	0.45	0.19	0.09	0.93	0.06	0.21	0.42	0.21	0.08	0.95	-0.03	0.22	0.42	0.22	0.08	0.95
6890	TAP1	-0.08	0.16	0.33	0.16	0.06	0.94	0.06	0.18	0.31	0.18	0.06	0.99	0.02	0.19	0.31	0.19	0.06	0.97
6905	TBCE	-0.06	0.17	0.40	0.18	0.08	0.94	0.05	0.17	0.36	0.18	0.07	0.96	-0.03	0.18	0.38	0.19	0.08	0.94
7062	TCHH	-0.03	0.16	0.35	0.16	0.06	0.94	0.04	0.15	0.29	0.16	0.05	0.96	-0.01	0.16	0.31	0.16	0.07	0.93
7068	THRB	-0.32	-0.17	0.04	-0.17	0.06	0.95	-0.28	-0.18	-0.08	-0.18	0.05	1.00	-0.34	-0.19	-0.06	-0.19	0.06	0.99
7089	TLE2	-0.09	0.16	0.38	0.16	0.07	0.93	0.05	0.18	0.36	0.18	0.06	0.97	0.00	0.18	0.37	0.18	0.07	0.95
7092	TLL1	-0.32	-0.15	0.04	-0.15	0.06	0.92	-0.33	-0.15	0.02	-0.15	0.06	0.96	-0.31	-0.17	-0.01	-0.17	0.06	0.96
7103	TSPAN8	-0.02	0.15	0.26	0.15	0.05	0.95	0.06	0.15	0.26	0.15	0.04	0.97	0.01	0.16	0.28	0.16	0.05	0.97
7138	TNNT1	-0.37	-0.16	0.10	-0.16	0.08	0.91	-0.35	-0.19	-0.03	-0.18	0.07	0.98	-0.37	-0.19	0.02	-0.19	0.07	0.95
7145	TNS1	-0.04	0.13	0.24	0.12	0.04	0.90	0.04	0.13	0.23	0.13	0.04	0.91	0.02	0.15	0.27	0.14	0.04	0.95
7222	TRPC3	-0.10	0.19	0.45	0.19	0.09	0.93	0.06	0.20	0.41	0.21	0.08	0.97	0.01	0.21	0.43	0.21	0.08	0.96
7378	UPP1	-0.09	0.17	0.43	0.17	0.08	0.92	0.04	0.18	0.36	0.19	0.07	0.96	-0.02	0.18	0.37	0.18	0.08	0.91
7402	UTRN	-0.07	0.17	0.33	0.16	0.06	0.93	0.04	0.16	0.27	0.16	0.06	0.93	-0.02	0.16	0.30	0.16	0.06	0.94
7409	VAV1	-0.38	-0.16	0.08	-0.15	0.07	0.90	-0.37	-0.18	-0.03	-0.18	0.07	0.96	-0.33	-0.18	0.00	-0.18	0.07	0.94
7480	WNT10B	-0.40	-0.17	0.13	-0.17	0.08	0.92	-0.35	-0.18	-0.06	-0.19	0.07	0.95	-0.38	-0.19	0.01	-0.20	0.08	0.95
7781	SLC30A3	-0.31	-0.17	0.08	-0.17	0.06	0.96	-0.30	-0.16	-0.06	-0.17	0.05	0.98	-0.33	-0.17	0.00	-0.17	0.06	0.96
8001	GLRA3	-0.42	-0.18	0.06	-0.19	0.08	0.94	-0.33	-0.18	-0.03	-0.18	0.07	0.95	-0.37	-0.19	0.00	-0.20	0.08	0.95
8174	MADCAM1	-0.05	0.16	0.37	0.16	0.07	0.91	0.03	0.18	0.36	0.18	0.06	0.97	0.02	0.19	0.39	0.19	0.07	0.96
8209	C21orf33	-0.03	0.13	0.26	0.13	0.05	0.90	0.02	0.14	0.25	0.14	0.05	0.92	-0.02	0.14	0.28	0.14	0.05	0.91
8321	FZD1	-0.40	-0.16	0.08	-0.16	0.08	0.91	-0.34	-0.17	-0.04	-0.18	0.06	0.96	-0.37	-0.17	0.01	-0.17	0.08	0.92
8437	RASAL1	-0.40	-0.17	0.06	-0.17	0.08	0.91	-0.36	-0.17	-0.02	-0.17	0.07	0.93	-0.36	-0.18	-0.01	-0.18	0.07	0.93
8445	DYRK2	-0.34	-0.15	0.08	-0.15	0.06	0.91	-0.27	-0.15	0.00	-0.15	0.06	0.90	-0.31	-0.16	0.01	-0.16	0.07	0.93
8482	SEMA7A	-0.08	0.19	0.45	0.20	0.09	0.95	0.06	0.21	0.40	0.21	0.08	0.99	-0.02	0.22	0.43	0.22	0.09	0.97
8604	SLC25A12	-0.03	0.16	0.37	0.17	0.07	0.92	0.06	0.18	0.34	0.18	0.06	0.96	0.01	0.18	0.34	0.18	0.07	0.93
8611	PLPP1	-0.06	0.14	0.28	0.14	0.05	0.92	0.07	0.15	0.24	0.15	0.04	0.99	0.02	0.16	0.27	0.16	0.05	0.96
8704	B4GALT2	-0.42	-0.19	0.07	-0.19	0.08	0.95	-0.34	-0.19	-0.03	-0.19	0.07	0.95	-0.38	-0.20	-0.01	-0.20	0.08	0.95
8715	NOL4	-0.38	-0.18	0.05	-0.18	0.07	0.95	-0.34	-0.19	-0.04	-0.19	0.07	0.96	-0.36	-0.20	0.02	-0.20	0.07	0.96
8717	TRADD	-0.05	0.14	0.25	0.13	0.05	0.93	0.02	0.14	0.24	0.14	0.04	0.96	-0.02	0.14	0.27	0.14	0.05	0.93
8792	TNFRSF11A	-0.36	-0.17	0.10	-0.18	0.07	0.94	-0.36	-0.18	-0.01	-0.18	0.07	0.96	-0.38	-0.21	-0.01	-0.20	0.07	0.97
8884	SLC5A6	-0.05	0.16	0.37	0.17	0.07	0.93	0.07	0.18	0.35	0.19	0.06	0.99	-0.02	0.19	0.36	0.19	0.07	0.96
8938	BAIAP3	-0.53	-0.22	0.08	-0.23	0.10	0.96	-0.44	-0.24	-0.07	-0.24	0.09	0.99	-0.47	-0.25	-0.02	-0.26	0.10	0.98
9020	MAP3K14	-0.09	0.15	0.31	0.15	0.06	0.92	0.06	0.17	0.29	0.17	0.05	0.99	0.00	0.16	0.29	0.16	0.06	0.93
9033	PKD2L1	-0.36	-0.21	0.06	-0.21	0.07	0.98	-0.31	-0.21	-0.07	-0.20	0.06	0.99	-0.38	-0.22	0.01	-0.21	0.07	0.98
9120	SLC16A6	-0.04	0.16	0.41	0.16	0.08	0.91	0.04	0.17	0.35	0.17	0.07	0.95	-0.02	0.18	0.37	0.18	0.08	0.94
9127	P2RX6	-0.08	0.19	0.45	0.19	0.08	0.95	0.02	0.21	0.43	0.21	0.07	0.99	0.01	0.22	0.43	0.22	0.08	0.96
9168	TMSB10	-0.36	-0.16	0.13	-0.16	0.08	0.91	-0.36	-0.18	-0.01	-0.18	0.07	0.96	-0.36	-0.19	0.00	-0.19	0.07	0.94
9196	KCNAB3	-0.09	0.19	0.44	0.19	0.08	0.94	0.07	0.20	0.40	0.20	0.07	0.98	0.00	0.20	0.42	0.20	0.08	0.95
9254	CACNA2D2	-0.03	0.15	0.34	0.15	0.06	0.91	0.05	0.15	0.31	0.16	0.06	0.97	-0.01	0.16	0.34	0.16	0.06	0.94
9256	TSPOAP1	-0.34	-0.16	0.02	-0.16	0.06	0.93	-0.31	-0.17	-0.05	-0.17	0.05	0.97	-0.35	-0.19	0.00	-0.19	0.06	0.97
9312	KCNB2	-0.34	-0.19	0.02	-0.18	0.06	0.96	-0.30	-0.18	-0.04	-0.18	0.06	0.98	-0.37	-0.20	-0.04	-0.20	0.06	0.98
9315	NREP	-0.04	0.16	0.36	0.16	0.07	0.91	0.06	0.17	0.35	0.18	0.06	0.99	-0.04	0.17	0.35	0.17	0.07	0.95
9362	CPNE6	-0.44	-0.17	0.08	-0.18	0.09	0.90	-0.42	-0.18	-0.04	-0.19	0.08	0.95	-0.41	-0.20	0.03	-0.20	0.08	0.95
9452	ITM2A	-0.09	0.18	0.39	0.18	0.08	0.93	0.06	0.20	0.35	0.20	0.07	0.99	0.02	0.21	0.38	0.21	0.07	0.96
9454	HOMER3	-0.04	0.17	0.32	0.17	0.06	0.96	0.06	0.18	0.31	0.18	0.05	0.99	0.05	0.20	0.35	0.20	0.05	0.98
9592	IER2	-0.08	0.16	0.37	0.16	0.07	0.93	0.05	0.18	0.34	0.18	0.06	0.99	0.02	0.19	0.34	0.18	0.07	0.95
9607	CARTPT	-0.45	-0.27	0.07	-0.25	0.09	0.97	-0.41	-0.24	-0.02	-0.25	0.08	0.99	-0.44	-0.26	-0.01	-0.25	0.08	0.98
9609	RAB36	-0.39	-0.17	0.06	-0.17	0.08	0.92	-0.34	-0.17	-0.06	-0.18	0.07	0.96	-0.36	-0.18	0.00	-0.19	0.07	0.95
9636	ISG15	-0.33	-0.16	0.04	-0.16	0.06	0.92	-0.35	-0.17	-0.04	-0.18	0.05	0.99	-0.36	-0.19	0.01	-0.18	0.06	0.96
9828	ARHGEF17	-0.03	0.15	0.33	0.15	0.06	0.91	0.07	0.16	0.32	0.17	0.05	1.00	0.02	0.17	0.31	0.17	0.06	0.96
9911	TMCC2	-0.04	0.15	0.35	0.16	0.07	0.92	0.05	0.16	0.33	0.17	0.06	0.97	-0.02	0.18	0.33	0.18	0.07	0.95
10083	USH1C	-0.01	0.14	0.25	0.14	0.05	0.94	0.04	0.15	0.25	0.15	0.04	0.96	0.04	0.16	0.26	0.15	0.05	0.95
10154	PLXNC1	-0.38	-0.18	0.03	-0.18	0.07	0.94	-0.35	-0.18	-0.08	-0.19	0.06	1.00	-0.36	-0.20	-0.04	-0.20	0.07	0.98
10160	FARP1	-0.43	-0.18	0.04	-0.18	0.08	0.93	-0.36	-0.19	-0.05	-0.20	0.07	0.99	-0.40	-0.21	0.01	-0.21	0.08	0.97
10171	RCL1	-0.05	0.14	0.29	0.14	0.06	0.91	0.03	0.15	0.27	0.15	0.05	0.95	-0.01	0.16	0.28	0.16	0.06	0.95
10231	RCAN2	-0.01	0.18	0.41	0.18	0.07	0.94	0.07	0.20	0.41	0.20	0.06	1.00	0.00	0.20	0.38	0.20	0.07	0.97
10268	RAMP3	-0.03	0.16	0.33	0.16	0.06	0.94	0.06	0.17	0.31	0.17	0.05	0.99	0.02	0.18	0.34	0.18	0.06	0.97
10332	CLEC4M	-0.02	0.14	0.30	0.14	0.05	0.93	0.04	0.14	0.26	0.14	0.05	0.95	0.02	0.15	0.29	0.15	0.05	0.95
10384	BTN3A3	-0.07	0.14	0.28	0.14	0.05	0.92	0.05	0.14	0.25	0.14	0.05	0.94	0.01	0.16	0.28	0.15	0.05	0.95
10395	DLC1	-0.04	0.16	0.31	0.16	0.05	0.93	0.02	0.14	0.26	0.16	0.05	0.97	-0.01	0.18	0.30	0.17	0.06	0.95
10451	VAV3	-0.06	0.22	0.46	0.23	0.09	0.96	0.06	0.23	0.40	0.23	0.08	0.99	0.01	0.22	0.45	0.23	0.09	0.96
10505	SEMA4F	-0.37	-0.18	0.04	-0.18	0.07	0.95	-0.32	-0.17	-0.05	-0.18	0.06	0.95	-0.36	-0.19	-0.01	-0.19	0.07	0.96
10613	ERLIN1	-0.29	-0.15	0.05	-0.15	0.06	0.91	-0.26	-0.13	-0.04	-0.13	0.05	0.91	-0.30	-0.15	0.02	-0.15	0.06	0.91
10673	TNFSF13B	-0.29	-0.14	0.06	-0.14	0.05	0.94	-0.23	-0.12	-0.01	-0.13	0.05	0.91	-0.26	-0.14	0.02	-0.14	0.05	0.92
10891	PPARGC1A	-0.04	0.19	0.40	0.19	0.08	0.94	0.07	0.20	0.43	0.20	0.07	0.98	-0.02	0.20	0.39	0.20	0.08	0.96
11069	RAPGEF4	-0.36	-0.17	0.10	-0.17	0.07	0.93	-0.33	-0.19	-0.08	-0.19	0.06	1.00	-0.35	-0.20	-0.03	-0.19	0.07	0.96
11138	TBC1D8	-0.04	0.17	0.33	0.17	0.06	0.95	0.04	0.17	0.31	0.17	0.06	0.99	0.02	0.18	0.32	0.17	0.06	0.94
11151	CORO1A	-0.31	-0.14	0.09	-0.14	0.06	0.90	-0.29	-0.15	-0.01	-0.16	0.06	0.96	-0.31	-0.17	0.00	-0.17	0.06	0.94
11164	NUDT5	-0.06	0.14	0.32	0.14	0.06	0.90	0.05	0.15	0.30	0.16	0.05	0.98	-0.02	0.17	0.30	0.16	0.06	0.94
11259	FILIP1L	-0.30	-0.15	0.05	-0.15	0.06	0.93	-0.29	-0.16	-0.05	-0.16	0.06	0.96	-0.29	-0.17	-0.01	-0.16	0.06	0.94
11279	KLF8	-0.34	-0.15	0.06	-0.15	0.06	0.92	-0.28	-0.15	-0.03	-0.16	0.05	0.95	-0.30	-0.16	-0.01	-0.17	0.06	0.94
22801	ITGA11	-0.05	0.21	0.38	0.21	0.07	0.96	0.04	0.23	0.36	0.22	0.07	0.99	0.03	0.21	0.38	0.21	0.07	0.98
22881	ANKRD6	-0.47	-0.18	0.09	-0.19	0.09	0.92	-0.40	-0.19	-0.05	-0.20	0.08	0.96	-0.42	-0.22	0.03	-0.22	0.09	0.97
22987	SV2C	-0.04	0.20	0.42	0.20	0.08	0.95	0.08	0.20	0.45	0.21	0.07	1.00	-0.06	0.22	0.41	0.22	0.08	0.97
22996	TTC39A	-0.06	0.17	0.36	0.17	0.06	0.94	0.06	0.16	0.31	0.17	0.05	0.97	-0.01	0.18	0.37	0.18	0.07	0.96
23046	KIF21B	-0.35	-0.16	0.03	-0.16	0.06	0.94	-0.31	-0.16	-0.06	-0.17	0.05	0.99	-0.32	-0.17	-0.02	-0.17	0.06	0.95
23109	DDN	-0.33	-0.16	0.07	-0.16	0.06	0.94	-0.29	-0.16	-0.04	-0.16	0.05	0.95	-0.31	-0.17	-0.04	-0.17	0.06	0.94
23180	RFTN1	-0.30	-0.15	0.06	-0.15	0.06	0.91	-0.25	-0.14	0.00	-0.14	0.05	0.90	-0.29	-0.15	0.05	-0.15	0.06	0.90
23245	ASTN2	-0.06	0.16	0.34	0.16	0.07	0.92	0.06	0.17	0.31	0.17	0.06	0.95	0.02	0.18	0.35	0.18	0.07	0.96
23274	CLEC16A	-0.08	0.16	0.39	0.16	0.07	0.92	0.03	0.17	0.35	0.18	0.07	0.98	0.03	0.18	0.35	0.18	0.07	0.96
23305	ACSL6	-0.05	0.15	0.34	0.15	0.06	0.90	0.03	0.16	0.30	0.16	0.06	0.91	-0.01	0.17	0.32	0.17	0.06	0.94
23406	COTL1	-0.02	0.15	0.28	0.15	0.05	0.95	0.04	0.13	0.26	0.14	0.05	0.95	-0.02	0.15	0.29	0.15	0.05	0.95
23460	ABCA6	-0.06	0.15	0.36	0.15	0.07	0.90	0.05	0.16	0.30	0.16	0.06	0.95	-0.01	0.17	0.33	0.17	0.06	0.93
23484	LEPROTL1	-0.02	0.18	0.40	0.18	0.07	0.95	0.07	0.18	0.38	0.19	0.06	1.00	0.00	0.20	0.36	0.20	0.07	0.97
23504	RIMBP2	-0.33	-0.16	0.08	-0.16	0.06	0.93	-0.28	-0.16	-0.05	-0.16	0.05	0.95	-0.32	-0.18	-0.01	-0.18	0.06	0.96
23642	SNHG1	-0.09	0.18	0.38	0.18	0.07	0.94	0.06	0.17	0.33	0.18	0.06	0.94	-0.01	0.18	0.36	0.19	0.07	0.94
25825	BACE2	-0.04	0.14	0.27	0.14	0.06	0.90	0.00	0.15	0.25	0.15	0.05	0.96	0.00	0.17	0.34	0.16	0.06	0.96
25841	ABTB2	-0.06	0.20	0.37	0.20	0.07	0.96	0.09	0.21	0.35	0.22	0.06	1.00	0.05	0.23	0.40	0.23	0.07	0.99
25900	IFFO1	-0.06	0.18	0.42	0.18	0.08	0.92	0.06	0.20	0.37	0.19	0.07	0.99	-0.02	0.19	0.38	0.20	0.08	0.94
25907	TMEM158	-0.41	-0.18	0.11	-0.18	0.08	0.93	-0.38	-0.20	-0.05	-0.20	0.07	0.98	-0.40	-0.20	-0.01	-0.20	0.08	0.96
25946	ZNF385A	-0.07	0.16	0.35	0.16	0.07	0.92	0.07	0.17	0.33	0.17	0.06	0.98	0.04	0.19	0.36	0.19	0.06	0.97
25953	PNKD	-0.04	0.15	0.37	0.15	0.07	0.91	0.04	0.15	0.36	0.16	0.06	0.94	-0.04	0.16	0.34	0.17	0.07	0.91
25989	ULK3	-0.05	0.19	0.39	0.19	0.07	0.95	0.08	0.20	0.37	0.21	0.06	1.00	0.04	0.21	0.40	0.21	0.07	0.98
26031	OSBPL3	-0.03	0.15	0.35	0.15	0.06	0.91	0.01	0.15	0.33	0.16	0.06	0.98	-0.01	0.17	0.33	0.17	0.06	0.95
26232	FBXO2	-0.32	-0.17	0.10	-0.16	0.07	0.92	-0.32	-0.19	-0.05	-0.18	0.06	0.98	-0.34	-0.18	0.01	-0.18	0.07	0.95
27077	B9D1	-0.39	-0.17	0.08	-0.17	0.08	0.91	-0.37	-0.17	-0.03	-0.18	0.07	0.94	-0.38	-0.19	0.00	-0.19	0.08	0.94
27132	CPNE7	-0.42	-0.18	0.07	-0.18	0.08	0.93	-0.38	-0.18	-0.06	-0.19	0.07	0.97	-0.40	-0.20	-0.03	-0.21	0.08	0.97
27163	NAAA	-0.06	0.18	0.37	0.17	0.07	0.94	0.04	0.18	0.33	0.19	0.06	0.97	0.02	0.19	0.37	0.19	0.07	0.95
28955	DEXI	-0.04	0.17	0.40	0.17	0.07	0.94	0.07	0.17	0.38	0.18	0.06	0.98	-0.01	0.19	0.35	0.19	0.07	0.95
29799	YPEL1	-0.40	-0.17	0.06	-0.17	0.08	0.91	-0.36	-0.18	-0.05	-0.18	0.06	0.98	-0.38	-0.21	0.02	-0.20	0.08	0.96
29803	REPIN1	-0.04	0.17	0.35	0.16	0.07	0.93	0.07	0.17	0.34	0.18	0.06	0.99	0.02	0.19	0.34	0.19	0.06	0.96
29844	TFPT	-0.38	-0.17	0.07	-0.17	0.07	0.91	-0.33	-0.19	-0.04	-0.19	0.06	0.98	-0.34	-0.19	0.01	-0.18	0.07	0.95
29997	NOP53	-0.11	0.15	0.35	0.15	0.07	0.91	0.05	0.16	0.31	0.16	0.06	0.91	-0.01	0.16	0.36	0.17	0.07	0.93
30850	CDR2L	-0.09	0.18	0.41	0.19	0.08	0.95	0.08	0.21	0.37	0.20	0.07	1.00	0.03	0.21	0.39	0.22	0.07	0.98
50486	G0S2	-0.01	0.16	0.33	0.16	0.06	0.94	0.07	0.17	0.34	0.17	0.05	1.00	0.00	0.18	0.34	0.18	0.06	0.98
50853	VILL	-0.05	0.15	0.35	0.15	0.06	0.90	0.00	0.16	0.34	0.16	0.06	0.96	-0.05	0.17	0.32	0.17	0.07	0.94
51059	FAM135B	-0.03	0.14	0.27	0.14	0.06	0.90	0.06	0.13	0.32	0.14	0.05	0.98	-0.04	0.14	0.30	0.14	0.06	0.90
51074	APIP	-0.06	0.14	0.27	0.14	0.06	0.91	0.04	0.14	0.26	0.14	0.05	0.95	0.02	0.15	0.28	0.15	0.05	0.94
51105	PHF20L1	-0.26	-0.14	0.08	-0.14	0.05	0.93	-0.24	-0.14	-0.01	-0.14	0.05	0.94	-0.26	-0.15	0.01	-0.15	0.05	0.95
51123	ZNF706	-0.36	-0.16	0.05	-0.16	0.07	0.90	-0.30	-0.16	-0.04	-0.17	0.06	0.94	-0.34	-0.18	0.03	-0.18	0.07	0.95
51134	CEP83	-0.29	-0.15	0.06	-0.15	0.06	0.93	-0.29	-0.17	-0.02	-0.17	0.05	0.98	-0.29	-0.16	0.02	-0.16	0.06	0.95
51312	SLC25A37	-0.10	0.18	0.44	0.18	0.08	0.92	0.04	0.19	0.41	0.20	0.08	0.98	0.00	0.20	0.39	0.20	0.08	0.94
51319	RSRC1	-0.05	0.15	0.38	0.15	0.07	0.91	0.03	0.16	0.32	0.16	0.06	0.93	-0.02	0.16	0.33	0.16	0.07	0.91
51334	PRR16	-0.04	0.16	0.33	0.16	0.07	0.92	0.04	0.16	0.30	0.17	0.06	0.94	-0.01	0.17	0.33	0.17	0.06	0.95
51375	SNX7	-0.43	-0.20	0.10	-0.19	0.09	0.95	-0.41	-0.23	-0.06	-0.22	0.07	0.99	-0.41	-0.23	-0.02	-0.23	0.08	0.97
51440	HPCAL4	-0.38	-0.16	0.09	-0.16	0.08	0.92	-0.34	-0.18	-0.05	-0.18	0.07	0.95	-0.37	-0.19	0.00	-0.19	0.07	0.95
51454	GULP1	-0.39	-0.20	0.06	-0.20	0.08	0.96	-0.34	-0.20	-0.01	-0.20	0.07	0.96	-0.42	-0.21	0.01	-0.21	0.08	0.96
51522	TMEM14C	-0.04	0.16	0.36	0.17	0.07	0.94	0.02	0.15	0.33	0.16	0.06	0.93	-0.04	0.18	0.34	0.18	0.07	0.95
51642	MRPL48	-0.08	0.13	0.29	0.14	0.06	0.91	0.02	0.14	0.25	0.14	0.05	0.94	-0.01	0.15	0.30	0.15	0.06	0.94
51660	MPC1	-0.04	0.18	0.37	0.18	0.07	0.95	0.06	0.18	0.35	0.18	0.06	0.99	0.01	0.19	0.34	0.19	0.07	0.96
51705	EMCN	-0.03	0.15	0.33	0.15	0.05	0.93	0.07	0.16	0.29	0.17	0.05	1.00	0.02	0.18	0.28	0.17	0.05	0.97
54112	GPR88	-0.37	-0.18	0.06	-0.18	0.07	0.94	-0.36	-0.19	-0.04	-0.20	0.06	0.99	-0.38	-0.20	0.01	-0.20	0.07	0.97
54536	EXOC6	-0.37	-0.17	0.07	-0.17	0.07	0.93	-0.35	-0.17	-0.03	-0.17	0.06	0.96	-0.36	-0.18	-0.01	-0.18	0.07	0.94
54550	NECAB2	-0.43	-0.19	0.07	-0.19	0.09	0.94	-0.39	-0.20	-0.05	-0.20	0.08	0.95	-0.43	-0.22	0.01	-0.22	0.09	0.95
54551	MAGEL2	-0.04	0.16	0.36	0.17	0.07	0.93	0.04	0.17	0.33	0.17	0.06	0.98	0.00	0.18	0.34	0.18	0.07	0.96
54566	EPB41L4B	-0.32	-0.15	0.07	-0.15	0.06	0.93	-0.29	-0.16	-0.02	-0.17	0.05	0.95	-0.31	-0.18	0.02	-0.17	0.06	0.95
54793	KCTD9	-0.02	0.18	0.42	0.18	0.08	0.93	0.05	0.18	0.41	0.19	0.07	0.98	-0.03	0.19	0.37	0.20	0.08	0.95
54843	SYTL2	-0.33	-0.17	0.05	-0.16	0.06	0.94	-0.30	-0.17	-0.05	-0.17	0.05	0.98	-0.33	-0.19	0.01	-0.18	0.06	0.97
54847	SIDT1	-0.32	-0.16	0.08	-0.16	0.06	0.93	-0.29	-0.15	-0.06	-0.15	0.06	0.91	-0.30	-0.17	0.00	-0.17	0.06	0.95
54874	FNBP1L	-0.41	-0.17	0.09	-0.17	0.08	0.93	-0.36	-0.20	-0.06	-0.19	0.06	0.98	-0.39	-0.20	-0.01	-0.21	0.07	0.97
55040	EPN3	-0.02	0.19	0.42	0.19	0.08	0.95	0.06	0.19	0.42	0.20	0.06	0.99	-0.01	0.21	0.38	0.21	0.08	0.97
55086	CXorf57	-0.44	-0.18	0.06	-0.19	0.09	0.93	-0.39	-0.19	-0.03	-0.20	0.07	0.95	-0.41	-0.20	0.03	-0.21	0.09	0.95
55118	CRTAC1	-0.03	0.16	0.34	0.16	0.06	0.94	0.07	0.17	0.30	0.17	0.05	1.00	0.03	0.18	0.32	0.18	0.06	0.97
55122	AKIRIN2	-0.34	-0.15	0.10	-0.15	0.07	0.90	-0.31	-0.16	-0.02	-0.17	0.06	0.96	-0.30	-0.17	0.00	-0.17	0.06	0.93
55160	ARHGEF10L	-0.07	0.16	0.33	0.16	0.07	0.91	0.02	0.16	0.32	0.16	0.06	0.92	-0.02	0.16	0.31	0.16	0.07	0.92
55170	PRMT6	-0.31	-0.14	0.05	-0.14	0.05	0.91	-0.26	-0.15	-0.05	-0.16	0.05	0.98	-0.28	-0.16	-0.03	-0.16	0.05	0.95
55208	DCUN1D2	-0.05	0.16	0.39	0.16	0.07	0.92	0.05	0.17	0.33	0.17	0.06	0.96	0.01	0.17	0.34	0.17	0.07	0.92

55244	SLC47A1	-0.03	0.24	0.44	0.24	0.08	0.98	0.12	0.24	0.45	0.25	0.07	1.00	0.03	0.26	0.46	0.25	0.08	0.99
55315	SLC29A3	-0.32	-0.17	0.02	-0.17	0.06	0.96	-0.30	-0.17	-0.07	-0.18	0.05	0.97	-0.32	-0.19	-0.02	-0.19	0.06	0.98
55353	LAPTM4B	-0.06	0.16	0.39	0.17	0.08	0.90	0.05	0.17	0.37	0.18	0.06	0.98	-0.02	0.18	0.36	0.19	0.07	0.96
55509	BATF3	-0.38	-0.18	0.10	-0.17	0.08	0.94	-0.37	-0.19	-0.04	-0.19	0.07	0.95	-0.38	-0.20	0.02	-0.20	0.07	0.96
55591	VEZT	-0.35	-0.15	0.08	-0.15	0.06	0.91	-0.30	-0.15	-0.03	-0.15	0.06	0.93	-0.31	-0.16	0.03	-0.16	0.07	0.92
55686	MREG	-0.02	0.16	0.34	0.16	0.06	0.93	0.07	0.16	0.33	0.16	0.05	1.00	-0.03	0.17	0.34	0.17	0.06	0.95
55800	SCN3B	-0.44	-0.20	0.06	-0.20	0.08	0.95	-0.43	-0.21	-0.07	-0.21	0.07	0.98	-0.42	-0.22	-0.01	-0.22	0.08	0.97
55853	IDI2-AS1	-0.05	0.17	0.38	0.18	0.07	0.94	0.06	0.17	0.36	0.18	0.06	0.98	-0.04	0.19	0.36	0.19	0.07	0.96
55897	MESP1	-0.41	-0.18	0.05	-0.18	0.08	0.94	-0.35	-0.19	-0.05	-0.20	0.07	0.99	-0.40	-0.21	-0.03	-0.21	0.07	0.97
56172	ANKH	-0.04	0.18	0.43	0.19	0.08	0.94	0.07	0.20	0.39	0.21	0.07	0.98	0.02	0.20	0.39	0.21	0.08	0.97
56648	EIF5A2	-0.07	0.17	0.42	0.18	0.08	0.92	0.05	0.18	0.39	0.19	0.07	0.97	0.00	0.20	0.40	0.20	0.08	0.96
56934	CA10	-0.40	-0.18	0.12	-0.18	0.08	0.94	-0.32	-0.18	-0.01	-0.18	0.07	0.95	-0.36	-0.18	0.05	-0.18	0.08	0.92
56937	PMEPA1	-0.04	0.20	0.39	0.20	0.07	0.97	0.07	0.22	0.37	0.22	0.06	0.99	0.03	0.22	0.40	0.22	0.07	0.99
56967	C14orf132	-0.35	-0.17	0.02	-0.17	0.06	0.96	-0.31	-0.16	-0.05	-0.16	0.05	0.96	-0.31	-0.17	-0.01	-0.17	0.06	0.95
56971	CEACAM19	-0.11	0.14	0.31	0.14	0.06	0.91	0.04	0.17	0.31	0.17	0.06	0.96	-0.01	0.16	0.30	0.16	0.06	0.91
57194	ATP10A	-0.05	0.16	0.31	0.16	0.06	0.95	0.07	0.17	0.32	0.19	0.05	0.99	0.06	0.19	0.33	0.19	0.05	0.99
57406	ABHD6	-0.04	0.15	0.31	0.15	0.06	0.93	0.05	0.15	0.26	0.15	0.05	0.99	0.01	0.16	0.30	0.16	0.06	0.95
57453	DSCAML1	-0.06	0.15	0.31	0.15	0.06	0.93	0.06	0.17	0.29	0.17	0.05	0.98	0.00	0.17	0.32	0.17	0.06	0.96
57465	TBC1D24	-0.34	-0.15	0.05	-0.16	0.07	0.91	-0.27	-0.15	-0.03	-0.16	0.06	0.94	-0.33	-0.17	-0.01	-0.17	0.06	0.95
57495	NWD2	-0.35	-0.20	0.03	-0.20	0.07	0.97	-0.36	-0.19	-0.06	-0.20	0.06	0.98	-0.37	-0.21	0.00	-0.21	0.07	0.98
57496	MKL2	-0.37	-0.18	0.05	-0.18	0.07	0.95	-0.33	-0.17	-0.05	-0.17	0.06	0.94	-0.35	-0.19	0.02	-0.19	0.07	0.95
57519	STARD9	-0.12	0.15	0.33	0.15	0.07	0.90	0.06	0.18	0.34	0.18	0.06	0.97	0.01	0.17	0.33	0.17	0.06	0.95
57526	PCDH19	-0.40	-0.21	0.05	-0.21	0.07	0.96	-0.37	-0.22	-0.08	-0.22	0.07	1.00	-0.40	-0.23	-0.04	-0.23	0.07	0.98
57596	BEGAIN	-0.26	-0.13	0.04	-0.13	0.05	0.90	-0.24	-0.14	-0.03	-0.14	0.05	0.94	-0.27	-0.15	-0.02	-0.15	0.05	0.94
57718	PPP4R4	-0.44	-0.18	0.13	-0.18	0.09	0.93	-0.39	-0.20	-0.05	-0.20	0.07	0.97	-0.42	-0.21	-0.01	-0.21	0.08	0.96
63974	NEUROD6	-0.32	-0.16	0.04	-0.17	0.06	0.93	-0.30	-0.18	-0.03	-0.18	0.06	0.98	-0.36	-0.19	-0.01	-0.19	0.06	0.98
63982	ANO3	-0.39	-0.17	0.12	-0.17	0.08	0.92	-0.36	-0.18	-0.03	-0.18	0.07	0.93	-0.36	-0.18	0.07	-0.18	0.08	0.93
64131	XYLT1	-0.42	-0.18	0.12	-0.18	0.08	0.93	-0.33	-0.19	-0.03	-0.19	0.07	0.96	-0.39	-0.20	-0.02	-0.20	0.08	0.97
64132	XYLT2	-0.09	0.17	0.36	0.17	0.07	0.94	0.03	0.17	0.35	0.18	0.06	0.98	0.02	0.19	0.37	0.19	0.07	0.95
64135	IFIH1	-0.03	0.15	0.28	0.15	0.05	0.93	0.02	0.16	0.26	0.16	0.05	0.96	0.00	0.17	0.30	0.17	0.05	0.98
64137	ABCG4	-0.09	0.15	0.36	0.15	0.07	0.90	0.05	0.15	0.32	0.16	0.06	0.96	0.00	0.17	0.36	0.17	0.07	0.94
64149	C17orf75	-0.04	0.15	0.33	0.15	0.06	0.92	0.03	0.14	0.33	0.15	0.06	0.96	-0.02	0.16	0.32	0.16	0.06	0.91
64333	ARHGAP9	-0.06	0.17	0.45	0.18	0.08	0.91	0.04	0.18	0.38	0.18	0.07	0.98	-0.03	0.20	0.39	0.20	0.08	0.96
64850	ETNPPL	-0.36	-0.15	0.07	-0.15	0.07	0.91	-0.30	-0.17	-0.05	-0.17	0.06	0.95	-0.34	-0.16	-0.01	-0.17	0.07	0.93
65997	RASL11B	-0.37	-0.19	0.07	-0.19	0.07	0.96	-0.34	-0.18	-0.04	-0.19	0.07	0.95	-0.37	-0.21	-0.01	-0.21	0.07	0.98
66000	TMEM108	-0.37	-0.17	0.04	-0.17	0.07	0.93	-0.31	-0.17	-0.05	-0.18	0.06	0.93	-0.35	-0.19	0.00	-0.19	0.07	0.96
66008	TRAK2	-0.06	0.16	0.33	0.16	0.06	0.94	0.05	0.15	0.28	0.16	0.06	0.98	0.01	0.17	0.32	0.17	0.06	0.95
79012	CAMKV	-0.35	-0.16	0.06	-0.16	0.07	0.91	-0.35	-0.16	-0.03	-0.17	0.06	0.94	-0.36	-0.18	0.00	-0.18	0.07	0.95
79017	GGCT	-0.42	-0.17	0.13	-0.17	0.08	0.93	-0.35	-0.18	-0.04	-0.18	0.07	0.97	-0.37	-0.19	-0.02	-0.19	0.07	0.94
79183	TTPAL	-0.31	-0.14	0.09	-0.14	0.06	0.91	-0.29	-0.16	0.00	-0.16	0.06	0.96	-0.30	-0.17	0.02	-0.16	0.06	0.94
79585	CORO7	-0.07	0.15	0.34	0.15	0.07	0.92	0.02	0.16	0.30	0.16	0.06	0.97	0.01	0.16	0.32	0.17	0.07	0.95
79645	EFCAB1	-0.46	-0.18	0.08	-0.19	0.09	0.93	-0.39	-0.19	-0.04	-0.20	0.08	0.95	-0.42	-0.21	-0.01	-0.21	0.09	0.95
79660	PPP1R3B	-0.01	0.16	0.31	0.16	0.05	0.95	0.08	0.16	0.28	0.16	0.04	1.00	0.02	0.17	0.28	0.17	0.05	0.98
79720	VPS37B	-0.27	-0.13	0.03	-0.13	0.05	0.91	-0.29	-0.13	-0.06	-0.13	0.05	0.97	-0.27	-0.13	0.02	-0.13	0.05	0.91
79745	CLIP4	-0.03	0.15	0.30	0.15	0.05	0.93	0.03	0.15	0.27	0.15	0.05	0.97	0.03	0.16	0.28	0.16	0.06	0.93
79750	ZNF385D	-0.04	0.16	0.33	0.16	0.06	0.94	0.04	0.16	0.33	0.16	0.06	0.95	0.00	0.17	0.33	0.17	0.06	0.95
79754	ASB13	-0.07	0.19	0.44	0.19	0.08	0.93	0.06	0.20	0.39	0.20	0.07	0.99	0.01	0.20	0.40	0.21	0.08	0.97
79822	ARHGAP28	-0.37	-0.18	0.06	-0.18	0.07	0.95	-0.33	-0.18	-0.04	-0.18	0.06	0.97	-0.36	-0.19	0.01	-0.19	0.07	0.96
79874	RABEP2	-0.06	0.16	0.32	0.16	0.06	0.95	0.07	0.18	0.29	0.17	0.05	1.00	0.03	0.18	0.33	0.18	0.06	0.96
79930	DOK3	-0.05	0.15	0.37	0.16	0.07	0.91	0.03	0.15	0.33	0.16	0.06	0.97	-0.03	0.18	0.37	0.18	0.07	0.94
79993	ELOVL7	-0.03	0.14	0.29	0.14	0.05	0.92	0.06	0.15	0.27	0.15	0.05	0.98	0.03	0.16	0.27	0.16	0.05	0.96
80020	FOXRED2	-0.36	-0.20	0.05	-0.20	0.07	0.96	-0.36	-0.20	-0.07	-0.20	0.06	0.99	-0.37	-0.22	-0.02	-0.22	0.07	0.99
80036	TRPM3	-0.07	0.18	0.40	0.19	0.08	0.95	0.05	0.19	0.36	0.20	0.07	0.97	0.01	0.20	0.42	0.20	0.07	0.96
80119	PIF1	-0.04	0.20	0.45	0.21	0.08	0.96	0.09	0.22	0.43	0.23	0.07	1.00	0.01	0.23	0.44	0.24	0.08	0.98
80176	SPSB1	-0.06	0.15	0.28	0.14	0.06	0.92	0.06	0.16	0.31	0.16	0.05	0.98	0.04	0.17	0.32	0.17	0.05	0.97
80179	MYO19	-0.12	0.16	0.41	0.16	0.08	0.91	0.06	0.18	0.38	0.18	0.07	0.94	-0.03	0.18	0.36	0.18	0.08	0.91
80307	FER1L4	-0.04	0.22	0.44	0.23	0.08	0.97	0.09	0.24	0.43	0.24	0.07	1.00	0.03	0.25	0.46	0.25	0.08	0.99
80323	CCDC68	-0.37	-0.17	0.09	-0.16	0.07	0.93	-0.31	-0.17	-0.02	-0.17	0.06	0.98	-0.33	-0.18	0.01	-0.18	0.07	0.95
80774	LIMD2	-0.24	-0.12	0.01	-0.12	0.04	0.91	-0.25	-0.13	-0.03	-0.13	0.05	0.92	-0.26	-0.13	0.00	-0.13	0.05	0.91
80820	EEPD1	-0.12	0.19	0.46	0.19	0.09	0.95	0.08	0.22	0.42	0.22	0.08	1.00	0.01	0.22	0.41	0.22	0.09	0.96
80854	SETD7	-0.05	0.17	0.34	0.17	0.07	0.93	0.04	0.16	0.31	0.17	0.06	0.98	0.02	0.17	0.33	0.18	0.07	0.94
81033	KCNH6	-0.05	0.18	0.41	0.19	0.08	0.94	0.07	0.19	0.38	0.20	0.06	0.99	-0.01	0.20	0.39	0.20	0.07	0.98
81539	SLC38A1	-0.06	0.14	0.35	0.14	0.06	0.90	0.02	0.15	0.31	0.15	0.06	0.95	0.01	0.16	0.31	0.16	0.06	0.94
81552	VOPP1	-0.30	-0.15	0.04	-0.15	0.06	0.92	-0.27	-0.16	-0.03	-0.16	0.05	0.98	-0.31	-0.16	-0.01	-0.16	0.06	0.95
81849	ST6GALNAC5	-0.40	-0.22	0.05	-0.22	0.08	0.97	-0.37	-0.23	-0.09	-0.23	0.07	1.00	-0.41	-0.23	-0.04	-0.24	0.07	0.99
83445	GSG1	-0.43	-0.18	0.07	-0.18	0.08	0.91	-0.35	-0.17	-0.01	-0.18	0.07	0.93	-0.37	-0.19	0.00	-0.19	0.08	0.95
83468	GLT8D2	-0.31	-0.15	0.06	-0.15	0.06	0.92	-0.34	-0.16	-0.04	-0.17	0.06	0.98	-0.31	-0.19	-0.01	-0.18	0.06	0.96
83482	SCRT1	-0.06	0.18	0.43	0.18	0.09	0.92	0.06	0.20	0.41	0.20	0.07	0.97	-0.02	0.20	0.42	0.21	0.08	0.97
83547	RILP	-0.04	0.17	0.40	0.17	0.07	0.91	0.03	0.17	0.38	0.17	0.06	0.97	-0.02	0.19	0.37	0.19	0.07	0.95
83660	TLN2	-0.35	-0.19	0.04	-0.19	0.07	0.96	-0.32	-0.19	-0.08	-0.19	0.06	1.00	-0.34	-0.21	-0.04	-0.21	0.06	0.98
83690	CRISPLD1	-0.08	0.15	0.30	0.15	0.06	0.94	-0.03	0.15	0.27	0.15	0.05	0.94	-0.02	0.17	0.31	0.16	0.06	0.95
83692	CD99L2	-0.03	0.16	0.40	0.17	0.08	0.92	0.07	0.19	0.34	0.19	0.06	0.99	0.00	0.19	0.39	0.19	0.07	0.95
83707	TRPT1	-0.07	0.14	0.33	0.14	0.06	0.91	0.03	0.13	0.28	0.14	0.05	0.93	0.00	0.16	0.32	0.16	0.06	0.93
83714	NRIP2	-0.04	0.15	0.30	0.15	0.05	0.94	0.04	0.15	0.28	0.15	0.05	0.95	0.02	0.17	0.30	0.17	0.05	0.96
84034	EMILIN2	-0.03	0.21	0.33	0.20	0.06	0.99	0.09	0.21	0.33	0.21	0.05	1.00	0.06	0.22	0.36	0.22	0.06	0.99
84109	QRFPR	-0.04	0.18	0.38	0.18	0.07	0.95	0.02	0.19	0.34	0.19	0.07	0.97	0.01	0.19	0.39	0.19	0.08	0.95
84187	TMEM164	-0.32	-0.15	0.06	-0.15	0.06	0.91	-0.29	-0.17	-0.02	-0.17	0.06	0.96	-0.31	-0.17	0.00	-0.17	0.06	0.93
84221	SPATC1L	-0.32	-0.16	0.06	-0.16	0.06	0.94	-0.29	-0.16	-0.06	-0.17	0.05	0.96	-0.31	-0.18	-0.03	-0.18	0.06	0.97
84439	HHIPL1	-0.37	-0.17	0.04	-0.17	0.07	0.93	-0.32	-0.18	-0.02	-0.18	0.06	0.97	-0.34	-0.17	0.01	-0.18	0.07	0.93
84524	ZC3H8	-0.21	-0.12	0.00	-0.12	0.04	0.91	-0.22	-0.12	-0.01	-0.12	0.04	0.94	-0.23	-0.13	-0.02	-0.12	0.04	0.93
84525	HOPX	-0.29	-0.15	0.05	-0.15	0.05	0.93	-0.26	-0.16	-0.04	-0.16	0.05	0.98	-0.31	-0.17	-0.05	-0.17	0.05	0.98
84623	KIRREL3	-0.36	-0.18	0.05	-0.18	0.06	0.95	-0.30	-0.18	-0.04	-0.17	0.06	0.98	-0.37	-0.20	-0.03	-0.20	0.06	0.98
84803	GPAT3	-0.06	0.18	0.45	0.18	0.09	0.91	0.05	0.19	0.39	0.19	0.07	0.96	-0.02	0.20	0.41	0.21	0.09	0.95
84812	PLCD4	-0.06	0.18	0.41	0.19	0.08	0.95	0.06	0.19	0.36	0.19	0.07	0.98	0.01	0.20	0.40	0.20	0.07	0.97
84936	ZFYVE19	-0.05	0.17	0.39	0.17	0.07	0.92	0.05	0.17	0.35	0.17	0.06	0.97	-0.01	0.18	0.34	0.18	0.07	0.93
84937	ZNRF1	-0.31	-0.14	0.04	-0.14	0.06	0.91	-0.30	-0.16	-0.06	-0.17	0.05	0.99	-0.29	-0.17	-0.05	-0.17	0.05	0.97
85461	TANC1	-0.04	0.14	0.27	0.14	0.05	0.91	0.04	0.14	0.26	0.14	0.05	0.94	0.03	0.16	0.26	0.15	0.05	0.95
89958	SAPCD2	-0.31	-0.14	0.05	-0.14	0.06	0.91	-0.26	-0.14	-0.03	-0.14	0.05	0.96	-0.28	-0.14	0.02	-0.14	0.05	0.91
90102	PHLDB2	-0.03	0.14	0.29	0.14	0.06	0.91	0.02	0.16	0.30	0.16	0.05	0.99	0.01	0.16	0.28	0.16	0.05	0.95
90523	MLIP	-0.32	-0.18	0.02	-0.17	0.05	0.97	-0.28	-0.17	-0.03	-0.17	0.05	0.97	-0.31	-0.19	-0.02	-0.19	0.05	0.99
90861	JPT2	0.00	0.13	0.24	0.13	0.04	0.94	0.03	0.15	0.23	0.14	0.04	0.97	0.04	0.15	0.25	0.15	0.04	0.97
90865	IL33	-0.38	-0.16	0.08	-0.16	0.07	0.91	-0.33	-0.18	-0.05	-0.18	0.06	0.94	-0.36	-0.18	-0.01	-0.18	0.07	0.93
91133	L3MBTL4	-0.30	-0.17	0.03	-0.16	0.06	0.95	-0.27	-0.18	-0.07	-0.18	0.05	1.00	-0.30	-0.17	-0.04	-0.17	0.05	0.96
91252	SLC39A13	-0.08	0.20	0.41	0.20	0.08	0.96	0.08	0.21	0.39	0.21	0.07	1.00	0.01	0.22	0.43	0.22	0.08	0.98
91624	NEXN	-0.04	0.18	0.38	0.18	0.07	0.96	0.05	0.18	0.33	0.19	0.06	0.97	-0.03	0.20	0.36	0.20	0.07	0.97
91683	SYT12	-0.07	0.16	0.38	0.16	0.07	0.90	0.07	0.18	0.34	0.18	0.06	0.98	-0.03	0.18	0.37	0.18	0.07	0.93
92335	STRADA	-0.03	0.14	0.31	0.14	0.06	0.91	0.04	0.15	0.28	0.16	0.05	0.96	0.00	0.16	0.30	0.16	0.06	0.93
92399	MRRF	-0.07	0.15	0.33	0.15	0.07	0.90	0.03	0.15	0.28	0.15	0.06	0.94	0.00	0.15	0.33	0.16	0.07	0.92
92597	MOB1B	-0.38	-0.16	0.11	-0.16	0.07	0.93	-0.34	-0.18	-0.04	-0.18	0.06	0.96	-0.37	-0.19	-0.01	-0.19	0.07	0.95
92610	TIFA	-0.07	0.16	0.40	0.17	0.08	0.91	0.03	0.16	0.32	0.17	0.07	0.93	-0.02	0.17	0.36	0.17	0.08	0.92
94160	ABCC12	-0.28	-0.15	0.09	-0.15	0.06	0.90	-0.27	-0.15	-0.03	-0.15	0.05	0.92	-0.28	-0.15	0.07	-0.16	0.06	0.94
113452	TMEM54	-0.34	-0.17	0.03	-0.17	0.06	0.94	-0.29	-0.18	-0.06	-0.18	0.05	0.97	-0.33	-0.18	0.02	-0.18	0.06	0.98
113675	SDSL	-0.04	0.16	0.36	0.16	0.07	0.92	0.04	0.15	0.35	0.16	0.06	0.95	-0.03	0.18	0.34	0.18	0.07	0.96
114571	SLC22A9	-0.30	-0.15	0.02	-0.14	0.06	0.92	-0.29	-0.14	-0.03	-0.14	0.05	0.96	-0.36	-0.16	0.02	-0.15	0.06	0.95
114787	GPRIN1	-0.35	-0.16	0.03	-0.16	0.06	0.93	-0.32	-0.16	-0.04	-0.17	0.06	0.98	-0.33	-0.18	0.01	-0.18	0.06	0.96
114990	VASN	-0.26	-0.15	0.01	-0.14	0.05	0.92	-0.26	-0.14	-0.03	-0.14	0.05	0.94	-0.27	-0.15	-0.01	-0.15	0.05	0.94
116135	LRRC3B	-0.44	-0.19	0.08	-0.19	0.08	0.94	-0.38	-0.20	-0.07	-0.21	0.07	1.00	-0.40	-0.21	-0.01	-0.21	0.08	0.97
117154	DACH2	-0.40	-0.17	0.15	-0.17	0.08	0.91	-0.36	-0.18	-0.03	-0.18	0.07	0.94	-0.39	-0.19	0.02	-0.19	0.08	0.93
117245	HRASLS5	-0.28	-0.13	0.01	-0.13	0.05	0.90	-0.22	-0.15	-0.05	-0.14	0.04	0.95	-0.28	-0.16	-0.03	-0.16	0.05	0.98
118427	OLFM3	-0.32	-0.17	0.10	-0.17	0.07	0.93	-0.27	-0.16	-0.03	-0.16	0.06	0.92	-0.33	-0.18	0.01	-0.17	0.07	0.94
118429	ANTXR2	-0.05	0.17	0.31	0.17	0.06	0.96	0.08	0.17	0.30	0.17	0.05	1.00	-0.01	0.17	0.30	0.18	0.05	0.98
119587	CPXM2	0.00	0.14	0.29	0.14	0.05	0.90	0.04	0.15	0.29	0.15	0.04	0.99	0.03	0.16	0.30	0.16	0.05	0.95
122622	ADSSL1	-0.06	0.14	0.28	0.14	0.05	0.92	0.04	0.15	0.26	0.15	0.05	0.97	0.01	0.16	0.29	0.16	0.05	0.94
122953	JDP2	-0.07	0.18	0.44	0.19	0.08	0.94	0.05	0.20	0.39	0.20	0.07	0.98	0.00	0.21	0.41	0.21	0.08	0.95
126755	LRRC38	-0.04	0.17	0.36	0.17	0.07	0.93	0.05	0.16	0.33	0.17	0.05	0.97	0.00	0.18	0.36	0.18	0.07	0.96
127833	SYT2	-0.04	0.23	0.48	0.23	0.09	0.96	0.10	0.23	0.48	0.24	0.07	1.00	-0.01	0.25	0.46	0.25	0.09	0.99
130399	ACVR1C	-0.07	0.18	0.39	0.18	0.07	0.95	0.05	0.19	0.42	0.20	0.06	0.99	0.04	0.20	0.36	0.20	0.07	0.97
132160	PPM1M	-0.42	-0.17	0.08	-0.17	0.08	0.90	-0.35	-0.18	-0.06	-0.19	0.07	0.98	-0.38	-0.20	-0.01	-0.20	0.08	0.96
132321	C4orf33	-0.10	0.18	0.39	0.18	0.08	0.93	0.04	0.19	0.36	0.19	0.07	0.97	0.00	0.20	0.38	0.20	0.07	0.97
133418	EMB	-0.06	0.15	0.34	0.16	0.07	0.91	0.03	0.15	0.29	0.15	0.06	0.92	-0.04	0.16	0.34	0.17	0.07	0.92
134548	SOWAHA	-0.38	-0.17	0.04	-0.17	0.07	0.93	-0.34	-0.18	-0.04	-0.18	0.06	0.96	-0.38	-0.19	0.02	-0.19	0.07	0.95
139221	MUM1L1	-0.40	-0.19	0.09	-0.19	0.08	0.94	-0.35	-0.20	-0.02	-0.20	0.08	0.94	-0.40	-0.20	0.05	-0.20	0.08	0.94
139411	PTCHD1	-0.40	-0.16	0.06	-0.16	0.07	0.91	-0.35	-0.16	-0.02	-0.16	0.07	0.92	-0.36	-0.19	0.00	-0.19	0.07	0.95
143279	HECTD2	-0.29	-0.14	0.06	-0.14	0.06	0.91	-0.26	-0.15	-0.04	-0.15	0.05	0.96	-0.30	-0.18	0.00	-0.18	0.05	0.97
144402	CPNE8	-0.32	-0.18	0.08	-0.17	0.06	0.96	-0.29	-0.17	-0.02	-0.17	0.05	0.99	-0.30	-0.18	0.00	-0.17	0.06	0.96
147463	ANKRD29	-0.05	0.18	0.41	0.18	0.08	0.94	0.06	0.18	0.37	0.19	0.07	0.97	-0.02	0.19	0.38	0.20	0.07	0.97
147968	CAPN12	-0.32	-0.17	0.09	-0.17	0.06	0.94	-0.30	-0.17	-0.04	-0.17	0.06	0.95	-0.33	-0.18	-0.01	-0.18	0.06	0.95
148281	SYT6	-0.03	0.20	0.39	0.20	0.08	0.96	0.03	0.18	0.32	0.18	0.07	0.98	-0.04	0.20	0.38	0.20	0.07	0.97
152189	CMTM8	-0.35	-0.15	0.04	-0.15	0.06	0.92	-0.31	-0.16	-0.06	-0.17	0.05	0.97	-0.34	-0.19	0.02	-0.19	0.06	0.97
152273	FGD5	-0.05	0.16	0.32	0.16	0.06	0.94	0.06	0.16	0.30	0.17	0.05	0.98	0.02	0.17	0.31	0.17	0.05	0.97
152940	C4orf45	-0.30	-0.16	0.06	-0.16	0.06	0.94	-0.27	-0.17	-0.05	-0.17	0.05	0.98	-0.34	-0.19	-0.03	-0.19	0.06	0.98
155382	VPS37D	-0.32	-0.14	0.05	-0.14	0.06	0.91	-0.28	-0.15	-0.03	-0.16	0.06	0.95	-0.30	-0.16	-0.01	-0.16	0.06	0.94
160760	PPTC7	-0.05	0.17	0.39	0.17	0.07	0.93	0.04	0.17	0.38	0.17	0.06	0.98	-0.04	0.19	0.36	0.19	0.08	0.94
162494	RHBDL3	-0.08	0.19	0.45	0.19	0.09	0.94	0.06	0.20	0.43	0.20	0.08	0.98	-0.04	0.21	0.43	0.21	0.09	0.96
163183	SYNE4	-0.08	0.18	0.42	0.19	0.08	0.94	0.06	0.19	0.38	0.20	0.07	0.98	0.02	0.20	0.41	0.20	0.08	0.96
163782	KANK4	-0.01	0.20	0.36	0.20	0.06	0.97	0.10	0.21	0.34	0.22	0.05	1.00	0.05	0.23	0.39	0.23	0.06	0.99
196383	RILPL2	-0.43	-0.18	0.08	-0.18	0.08	0.93	-0.38	-0.19	-0.04	-0.20	0.07	0.96	-0.41	-0.20	0.02	-0.21	0.08	0.96
200058	FLJ23867	-0.08	0.16	0.37	0.17	0.07	0.93	0.03	0.18	0.36	0.18	0.07	0.95	-0.01	0.18	0.39	0.19	0.07	0.95
200942	KLHDC8B	-0.41	-0.17	0.07	-0.17	0.08	0.91	-0.34	-0.18	-0.04	-0.18	0.07	0.95	-0.36	-0.19	0.00	-0.19	0.08	0.95
201191	SAMD14	-0.28	-0.16	0.03	-0.15	0.05	0.92	-0.25	-0.17	-0.05	-0.17	0.05	0.99	-0.29	-0.17	0.00	-0.17	0.06	0.97
202333	CMYA5	-0.09	0.18	0.38	0.18	0.08	0.93	0.03	0.19	0.37	0.19	0.07	0.97	0.01	0.21	0.40	0.21	0.07	0.97
220202	ATOH7	-0.41	-0.18	0.10	-0.18	0.08	0.93	-0.39	-0.19	-0.04	-0.20	0.07	0.98	-0.40	-0.21	-0.03	-0.21	0.08	0.96
221294	NT5DC1	-0.04	0.17	0.33	0.17	0.06	0.94	0.08	0.18	0.30	0.18	0.05	1.00	0.05	0.20	0.33	0.19	0.06	0.98
221336	BEND6	-0.05	0.16	0.40	0.17	0.08	0.90	0.03	0.17	0.35	0.17	0.07	0.93	-0.04	0.19	0.36	0.19	0.08	0.94
221421	RSPH9	-0.40	-0.17	0.04	-0.17	0.08	0.90	-0.37	-0.17	-0.05	-0.18	0.07	0.96	-0.37	-0.19	0.01	-0.19	0.08	0.94

222537	HS3ST5	-0.04	0.19	0.40	0.19	0.07	0.95	0.08	0.21	0.38	0.22	0.06	1.00	0.02	0.22	0.40	0.22	0.07	0.98
253832	ZDHHC20	-0.04	0.13	0.25	0.13	0.05	0.92	0.04	0.15	0.26	0.15	0.04	0.97	0.02	0.16	0.26	0.15	0.05	0.95
254102	EHBP1L1	-0.34	-0.19	0.04	-0.19	0.06	0.96	-0.33	-0.20	-0.06	-0.20	0.05	0.99	-0.35	-0.21	-0.07	-0.20	0.06	1.00
260434	PYDC1	-0.39	-0.17	0.06	-0.17	0.07	0.93	-0.36	-0.19	-0.06	-0.19	0.06	0.99	-0.39	-0.20	0.00	-0.20	0.07	0.98
283209	PGM2L1	-0.35	-0.15	0.09	-0.16	0.07	0.91	-0.32	-0.16	-0.03	-0.16	0.06	0.93	-0.33	-0.18	-0.03	-0.18	0.07	0.95
283284	IGSF22	-0.32	-0.15	0.03	-0.15	0.06	0.91	-0.31	-0.17	-0.05	-0.17	0.05	0.98	-0.32	-0.18	-0.03	-0.18	0.06	0.98
283316	CD163L1	-0.05	0.14	0.30	0.14	0.05	0.93	0.04	0.13	0.25	0.13	0.05	0.92	-0.03	0.14	0.27	0.14	0.06	0.91
284119	CAVIN1	-0.04	0.11	0.21	0.11	0.04	0.91	0.04	0.13	0.21	0.13	0.04	0.98	0.04	0.14	0.23	0.14	0.04	0.97
284339	TMEM145	-0.12	0.16	0.38	0.16	0.08	0.90	0.05	0.18	0.36	0.18	0.07	0.96	0.00	0.18	0.36	0.18	0.08	0.91
284348	LYPD5	-0.06	0.17	0.43	0.18	0.08	0.92	0.05	0.19	0.41	0.19	0.07	0.97	-0.04	0.20	0.41	0.20	0.08	0.95
284415	VSTM1	-0.04	0.17	0.37	0.17	0.06	0.94	0.02	0.15	0.28	0.15	0.06	0.91	-0.03	0.17	0.33	0.16	0.06	0.94
284454	LOC284454	-0.05	0.15	0.29	0.15	0.05	0.94	0.04	0.16	0.34	0.16	0.05	0.98	0.00	0.18	0.31	0.18	0.05	0.96
284611	FAM102B	-0.35	-0.15	0.08	-0.15	0.07	0.90	-0.29	-0.15	-0.01	-0.16	0.06	0.92	-0.34	-0.16	-0.01	-0.17	0.07	0.94
284716	RIMKLA	-0.07	0.17	0.41	0.17	0.08	0.92	0.05	0.17	0.39	0.18	0.07	0.98	-0.02	0.19	0.37	0.19	0.08	0.96
285755	PPIL6	-0.29	-0.14	0.05	-0.14	0.05	0.93	-0.25	-0.16	-0.02	-0.15	0.05	0.97	-0.29	-0.16	-0.04	-0.17	0.05	0.97
285780	LY86-AS1	-0.38	-0.17	0.10	-0.17	0.07	0.95	-0.31	-0.18	-0.02	-0.18	0.07	0.94	-0.35	-0.19	0.03	-0.19	0.07	0.95
286133	SCARA5	-0.46	-0.20	0.04	-0.21	0.09	0.96	-0.42	-0.22	-0.08	-0.23	0.07	1.00	-0.43	-0.23	0.04	-0.24	0.09	0.97
326624	RAB37	-0.04	0.20	0.45	0.20	0.08	0.96	0.08	0.21	0.43	0.22	0.07	1.00	0.02	0.23	0.44	0.23	0.08	0.98
339983	NAT8L	-0.05	0.16	0.42	0.16	0.08	0.90	0.04	0.17	0.36	0.17	0.07	0.96	-0.04	0.18	0.37	0.19	0.07	0.95
340348	TSPAN33	-0.39	-0.17	0.04	-0.17	0.08	0.92	-0.32	-0.16	0.00	-0.17	0.07	0.92	-0.36	-0.18	0.03	-0.18	0.07	0.94
340719	NANOS1	-0.38	-0.16	0.11	-0.16	0.08	0.90	-0.33	-0.18	-0.05	-0.18	0.07	0.97	-0.37	-0.19	-0.01	-0.19	0.08	0.95
342667	STAC2	-0.06	0.20	0.47	0.21	0.09	0.95	0.06	0.22	0.46	0.22	0.07	0.99	-0.02	0.23	0.45	0.23	0.08	0.98
348013	TMEM255B	-0.04	0.18	0.38	0.18	0.07	0.96	0.05	0.18	0.32	0.19	0.06	0.99	0.00	0.19	0.36	0.19	0.07	0.97
349136	WDR86	-0.49	-0.22	0.08	-0.22	0.09	0.96	-0.43	-0.22	-0.04	-0.24	0.08	0.98	-0.45	-0.23	-0.01	-0.24	0.09	0.97
373156	GSTK1	-0.04	0.14	0.27	0.14	0.05	0.90	0.02	0.13	0.25	0.14	0.05	0.96	0.00	0.15	0.26	0.15	0.05	0.96
373863	DND1	-0.08	0.17	0.38	0.16	0.07	0.92	0.05	0.17	0.34	0.17	0.06	0.95	0.00	0.18	0.36	0.18	0.07	0.92
374378	GALNT18	-0.27	-0.13	0.03	-0.13	0.05	0.91	-0.24	-0.12	-0.03	-0.13	0.04	0.91	-0.26	-0.14	-0.01	-0.14	0.05	0.93
386618	KCTD4	-0.43	-0.19	0.04	-0.19	0.08	0.94	-0.43	-0.20	-0.02	-0.21	0.07	0.99	-0.43	-0.21	0.01	-0.22	0.08	0.97
387357	THEMIS	-0.03	0.15	0.30	0.15	0.06	0.92	0.01	0.15	0.30	0.15	0.05	0.95	-0.05	0.15	0.30	0.15	0.06	0.92
389073	C2orf80	-0.33	-0.17	0.08	-0.16	0.06	0.93	-0.30	-0.18	-0.06	-0.18	0.06	0.98	-0.36	-0.19	-0.05	-0.19	0.06	0.97
400569	MED11	-0.07	0.15	0.33	0.15	0.07	0.90	0.04	0.15	0.30	0.16	0.06	0.91	-0.03	0.17	0.33	0.17	0.06	0.94
400745	SH2D5	-0.35	-0.18	0.07	-0.17	0.07	0.94	-0.29	-0.18	-0.03	-0.17	0.06	0.94	-0.38	-0.19	0.01	-0.19	0.07	0.95
404217	CTXN1	-0.41	-0.19	0.10	-0.19	0.08	0.94	-0.39	-0.20	-0.05	-0.21	0.07	0.99	-0.38	-0.22	-0.02	-0.22	0.08	0.97
415116	PIM3	-0.06	0.16	0.38	0.16	0.08	0.90	0.05	0.18	0.34	0.18	0.06	0.97	-0.02	0.19	0.36	0.19	0.07	0.95
503542	SPRN	-0.37	-0.18	0.10	-0.18	0.07	0.95	-0.32	-0.18	-0.03	-0.18	0.07	0.95	-0.36	-0.19	0.00	-0.19	0.07	0.95
574036	SERTAD4-AS1	-0.07	0.19	0.45	0.19	0.08	0.93	0.07	0.20	0.39	0.21	0.07	0.99	0.00	0.21	0.39	0.21	0.08	0.97
642273	FAM110C	-0.50	-0.23	0.06	-0.23	0.09	0.96	-0.41	-0.23	-0.06	-0.24	0.08	0.99	-0.45	-0.25	-0.01	-0.25	0.09	0.99
646424	SPINK8	-0.05	0.15	0.27	0.15	0.06	0.92	0.02	0.15	0.25	0.15	0.05	0.94	0.00	0.16	0.31	0.16	0.06	0.93
646627	LYPD8	-0.42	-0.18	0.12	-0.18	0.08	0.92	-0.37	-0.19	-0.02	-0.19	0.07	0.97	-0.39	-0.19	0.04	-0.19	0.09	0.92
654502	IQCJ	-0.34	-0.15	0.02	-0.16	0.06	0.94	-0.26	-0.14	-0.02	-0.15	0.05	0.94	-0.31	-0.17	-0.01	-0.17	0.06	0.95
654790	PCP4L1	-0.05	0.16	0.38	0.16	0.07	0.94	0.04	0.17	0.33	0.18	0.06	0.99	0.01	0.18	0.35	0.18	0.06	0.97
100507436	MICA	-0.04	0.16	0.30	0.16	0.06	0.94	0.05	0.16	0.28	0.16	0.05	0.96	0.01	0.18	0.30	0.17	0.06	0.96

Abbreviations: rsFC, resting-state functional connectivity; L, left; A4hf, head and face region of area 4; CNP, the Consortium for Neuropsychiatric Phenomics; SALD, the Southwest University Adult Lifespan Dataset; Min, minimum; Med, median; Max, maximum; SD, standard deviation.

Genes related to rsFC of the R-A4hf in both the discovery and validation experiments

GeneID	Genesymbol	Discovery dataset						CNP dataset						SALD dataset					
		r					Percentage	*r*					Percentage	*r*					Percentage
		Min	Med	Max	Mean	SD		Min	Med	Max	Mean	SD		Min	Med	Max	Mean	SD	
1006	CDH8	-0.39	-0.18	0.10	-0.17	0.08	0.92	-0.35	-0.20	0.03	-0.20	0.07	0.94	-0.40	-0.20	0.04	-0.20	0.07	0.95
4258	MGST2	-0.14	0.18	0.44	0.18	0.09	0.91	-0.06	0.19	0.36	0.19	0.08	0.90	-0.07	0.22	0.42	0.21	0.08	0.95
4703	NEB	-0.16	0.18	0.40	0.18	0.09	0.92	-0.09	0.19	0.36	0.19	0.09	0.92	-0.04	0.21	0.42	0.21	0.08	0.95
6330	SCN4B	-0.18	0.19	0.48	0.19	0.09	0.91	-0.04	0.20	0.42	0.20	0.09	0.91	-0.06	0.23	0.45	0.23	0.09	0.96
9033	PKD2L1	-0.34	-0.18	0.06	-0.17	0.08	0.91	-0.32	-0.19	0.07	-0.17	0.07	0.91	-0.37	-0.20	0.09	-0.19	0.07	0.95
9607	CARTPT	-0.40	-0.23	0.14	-0.22	0.09	0.96	-0.43	-0.23	0.09	-0.22	0.10	0.91	-0.43	-0.24	0.04	-0.23	0.09	0.96
22801	ITGA11	-0.11	0.18	0.39	0.17	0.08	0.90	-0.03	0.20	0.35	0.19	0.08	0.93	-0.06	0.20	0.40	0.20	0.08	0.94
51454	GULP1	-0.38	-0.17	0.11	-0.17	0.08	0.92	-0.34	-0.19	0.07	-0.18	0.08	0.91	-0.39	-0.20	0.08	-0.20	0.08	0.95
55244	SLC47A1	-0.15	0.20	0.45	0.20	0.09	0.93	-0.03	0.21	0.41	0.21	0.08	0.93	-0.05	0.23	0.45	0.22	0.09	0.96
57526	PCDH19	-0.41	-0.17	0.12	-0.17	0.08	0.90	-0.33	-0.18	0.05	-0.18	0.08	0.91	-0.41	-0.20	0.05	-0.20	0.08	0.92
81849	ST6GALNAC5	-0.45	-0.18	0.13	-0.18	0.09	0.93	-0.34	-0.20	0.04	-0.20	0.08	0.92	-0.43	-0.21	0.02	-0.22	0.08	0.96
84034	EMILIN2	-0.07	0.17	0.32	0.17	0.06	0.93	-0.04	0.18	0.29	0.18	0.07	0.93	-0.02	0.20	0.35	0.20	0.06	0.97
163782	KANK4	-0.07	0.16	0.32	0.16	0.07	0.91	-0.08	0.19	0.31	0.18	0.07	0.93	-0.04	0.20	0.37	0.20	0.07	0.96

Abbreviations: rsFC, resting-state functional connectivity; R, right; A4hf, head and face region of area 4; CNP, the Consortium for Neuropsychiatric Phenomics; SALD, the Southwest University Adult Lifespan Dataset; Min, minimum; Med, median; Max, maximum; SD, standard deviation.

Genes related to rsFC of the L-A6cdl in both the discovery and validation experiments

GeneID	Genesymbol	Discovery dataset						CNP dataset						SALD dataset					
		r					Percentage	*r*					Percentage	*r*					Percentage
		Min	Med	Max	Mean	SD		Min	Med	Max	Mean	SD		Min	Med	Max	Mean	SD	
92	ACVR2A	-0.31	-0.15	0.02	-0.15	0.05	0.93	-0.32	-0.17	-0.05	-0.17	0.05	0.99	-0.33	-0.17	-0.01	-0.17	0.05	0.96
176	ACAN	-0.10	0.21	0.42	0.20	0.09	0.93	0.08	0.23	0.45	0.23	0.08	0.98	0.01	0.24	0.45	0.23	0.09	0.96
326	AIRE	-0.04	0.15	0.32	0.15	0.06	0.93	0.01	0.16	0.33	0.16	0.06	0.98	-0.01	0.17	0.31	0.17	0.06	0.95
784	CACNB3	-0.30	-0.14	0.04	-0.14	0.06	0.90	-0.28	-0.17	-0.03	-0.16	0.05	0.99	-0.29	-0.17	0.01	-0.16	0.05	0.95
793	CALB1	-0.38	-0.17	0.09	-0.17	0.08	0.93	-0.35	-0.20	-0.07	-0.19	0.07	0.97	-0.40	-0.20	0.03	-0.20	0.08	0.94
817	CAMK2D	-0.37	-0.18	0.14	-0.18	0.08	0.91	-0.42	-0.21	-0.05	-0.21	0.07	0.97	-0.41	-0.21	0.01	-0.21	0.08	0.94
1893	ECM1	-0.10	0.17	0.38	0.17	0.08	0.91	0.04	0.19	0.39	0.19	0.06	0.97	-0.04	0.18	0.33	0.18	0.07	0.92
2044	EPHA5	-0.28	-0.13	0.03	-0.14	0.05	0.91	-0.31	-0.16	-0.03	-0.16	0.05	0.98	-0.29	-0.17	0.00	-0.16	0.05	0.95
2830	GPR6	-0.34	-0.16	0.04	-0.16	0.06	0.94	-0.32	-0.19	-0.05	-0.19	0.06	0.98	-0.33	-0.18	0.01	-0.18	0.06	0.96
2952	GSTT1	-0.09	0.19	0.41	0.19	0.08	0.94	0.07	0.21	0.42	0.21	0.07	0.97	-0.03	0.21	0.39	0.21	0.08	0.95
3290	HSD11B1	-0.09	0.16	0.31	0.16	0.07	0.90	0.09	0.18	0.32	0.19	0.05	0.99	0.02	0.20	0.35	0.20	0.06	0.97
3736	KCNA1	-0.08	0.18	0.40	0.19	0.08	0.93	0.08	0.20	0.41	0.20	0.07	0.99	-0.04	0.21	0.39	0.21	0.08	0.95
3755	KCNG1	-0.41	-0.18	0.11	-0.18	0.09	0.92	-0.44	-0.21	-0.07	-0.21	0.07	0.97	-0.41	-0.20	0.01	-0.20	0.08	0.94
3787	KCNS1	-0.05	0.17	0.41	0.17	0.08	0.91	0.03	0.18	0.38	0.18	0.07	0.95	-0.07	0.19	0.36	0.18	0.08	0.93
4082	MARCKS	-0.39	-0.17	0.06	-0.18	0.07	0.93	-0.39	-0.19	-0.06	-0.19	0.06	0.96	-0.37	-0.21	0.00	-0.21	0.07	0.95
4599	MX1	-0.07	0.17	0.36	0.17	0.08	0.92	0.04	0.20	0.39	0.20	0.06	0.97	0.02	0.21	0.37	0.20	0.07	0.95
4741	NEFM	-0.02	0.14	0.33	0.15	0.06	0.91	0.05	0.15	0.30	0.15	0.06	0.92	-0.03	0.16	0.32	0.16	0.06	0.92
4744	NEFH	-0.03	0.18	0.41	0.19	0.08	0.95	0.07	0.19	0.41	0.20	0.06	0.99	-0.02	0.20	0.38	0.20	0.08	0.96
5121	PCP4	-0.11	0.16	0.35	0.16	0.07	0.90	0.03	0.19	0.34	0.18	0.06	0.97	-0.01	0.19	0.38	0.19	0.07	0.94
5409	PNMT	-0.43	-0.19	0.10	-0.20	0.08	0.94	-0.42	-0.22	-0.06	-0.21	0.07	0.98	-0.42	-0.22	0.02	-0.22	0.08	0.96
6330	SCN4B	-0.09	0.21	0.42	0.21	0.09	0.96	0.09	0.24	0.46	0.24	0.07	0.99	0.03	0.24	0.42	0.24	0.08	0.98
6482	ST3GAL1	0.01	0.14	0.25	0.14	0.05	0.91	0.02	0.14	0.21	0.14	0.04	0.95	0.00	0.15	0.27	0.15	0.05	0.95
8557	TCAP	-0.27	-0.14	0.01	-0.14	0.05	0.91	-0.23	-0.12	0.03	-0.13	0.05	0.90	-0.31	-0.15	0.01	-0.15	0.05	0.92
9256	TSPOAP1	-0.32	-0.15	0.07	-0.16	0.06	0.91	-0.31	-0.17	-0.04	-0.17	0.05	0.96	-0.34	-0.18	0.00	-0.18	0.06	0.94
9473	THEMIS2	-0.04	0.15	0.30	0.16	0.05	0.95	0.05	0.16	0.28	0.16	0.04	0.97	0.01	0.18	0.29	0.18	0.05	0.96
9651	PLCH2	-0.27	-0.13	0.03	-0.14	0.05	0.90	-0.28	-0.15	-0.04	-0.14	0.05	0.97	-0.27	-0.15	0.03	-0.15	0.05	0.95
10160	FARP1	-0.40	-0.17	0.08	-0.18	0.08	0.92	-0.40	-0.19	-0.05	-0.19	0.07	0.97	-0.38	-0.20	0.02	-0.20	0.08	0.95
10231	RCAN2	-0.07	0.17	0.39	0.17	0.08	0.92	0.08	0.20	0.41	0.20	0.06	0.99	-0.02	0.19	0.37	0.19	0.07	0.93
10268	RAMP3	-0.11	0.15	0.32	0.15	0.06	0.92	0.03	0.16	0.32	0.17	0.05	0.98	-0.01	0.17	0.32	0.17	0.06	0.92
10683	DLL3	-0.33	-0.15	0.06	-0.16	0.07	0.91	-0.33	-0.17	-0.06	-0.17	0.05	0.98	-0.35	-0.19	0.00	-0.18	0.06	0.94
22987	SV2C	-0.04	0.19	0.44	0.20	0.08	0.95	0.07	0.21	0.45	0.21	0.07	0.99	0.01	0.21	0.40	0.21	0.08	0.96
22996	TTC39A	-0.05	0.15	0.34	0.16	0.07	0.91	0.05	0.17	0.32	0.17	0.06	0.95	-0.01	0.17	0.33	0.17	0.07	0.94
23406	COTL1	-0.03	0.14	0.28	0.14	0.05	0.91	0.04	0.15	0.28	0.15	0.05	0.95	-0.01	0.15	0.30	0.15	0.05	0.94
25953	PNKD	-0.03	0.16	0.36	0.16	0.07	0.91	0.05	0.16	0.37	0.16	0.06	0.93	-0.02	0.17	0.34	0.17	0.07	0.93
26010	SPATS2L	-0.22	-0.12	0.00	-0.12	0.04	0.90	-0.20	-0.13	-0.02	-0.12	0.04	0.95	-0.22	-0.13	-0.02	-0.13	0.04	0.94
29799	YPEL1	-0.40	-0.18	0.05	-0.18	0.07	0.94	-0.38	-0.19	-0.05	-0.19	0.07	0.96	-0.39	-0.21	0.01	-0.21	0.08	0.96
50486	G0S2	-0.03	0.16	0.32	0.17	0.06	0.94	0.09	0.18	0.36	0.19	0.05	0.99	0.02	0.19	0.32	0.19	0.06	0.96
50853	VILL	0.01	0.16	0.35	0.16	0.06	0.90	0.02	0.16	0.34	0.17	0.06	0.92	-0.07	0.17	0.33	0.16	0.07	0.92
51059	FAM135B	-0.02	0.14	0.29	0.14	0.06	0.91	0.06	0.16	0.33	0.16	0.05	0.96	0.01	0.15	0.31	0.15	0.06	0.92
54492	NEURL1B	-0.37	-0.18	0.00	-0.18	0.07	0.95	-0.36	-0.19	-0.03	-0.18	0.06	0.98	-0.36	-0.19	-0.02	-0.20	0.07	0.96
54551	MAGEL2	-0.07	0.15	0.34	0.16	0.07	0.90	0.05	0.17	0.33	0.17	0.06	0.94	-0.02	0.17	0.31	0.17	0.07	0.92
54793	KCTD9	-0.06	0.18	0.41	0.18	0.08	0.94	0.06	0.20	0.42	0.19	0.07	0.97	-0.04	0.19	0.36	0.19	0.08	0.95
55040	EPN3	-0.06	0.18	0.40	0.18	0.08	0.93	0.07	0.19	0.43	0.20	0.07	0.99	-0.03	0.20	0.38	0.20	0.08	0.95
55244	SLC47A1	-0.11	0.21	0.41	0.21	0.08	0.96	0.06	0.24	0.45	0.24	0.07	0.98	0.03	0.22	0.41	0.22	0.08	0.96
55686	MREG	-0.05	0.17	0.33	0.17	0.06	0.94	0.08	0.17	0.34	0.18	0.05	0.99	-0.01	0.18	0.34	0.18	0.06	0.97
56967	C14orf132	-0.30	-0.16	0.05	-0.16	0.06	0.94	-0.28	-0.16	-0.05	-0.17	0.05	0.98	-0.35	-0.17	0.01	-0.17	0.05	0.96
79660	PPP1R3B	-0.04	0.15	0.28	0.14	0.05	0.92	0.06	0.15	0.28	0.16	0.04	0.97	0.02	0.16	0.27	0.16	0.05	0.93
79767	ELMO3	-0.01	0.16	0.36	0.16	0.06	0.93	0.01	0.14	0.31	0.15	0.06	0.92	-0.07	0.16	0.31	0.16	0.07	0.91
80212	CCDC92	0.00	0.13	0.23	0.13	0.04	0.92	0.03	0.13	0.22	0.12	0.04	0.92	0.02	0.14	0.25	0.14	0.04	0.94
80307	FER1L4	-0.15	0.19	0.39	0.19	0.09	0.92	0.05	0.22	0.42	0.22	0.07	0.98	-0.01	0.21	0.40	0.21	0.08	0.94
83547	RILP	-0.03	0.16	0.39	0.17	0.07	0.92	0.04	0.17	0.38	0.17	0.06	0.93	-0.05	0.18	0.36	0.18	0.08	0.92
84034	EMILIN2	-0.12	0.16	0.33	0.15	0.06	0.91	0.08	0.19	0.32	0.19	0.05	0.99	0.04	0.19	0.34	0.19	0.06	0.98
90523	MLIP	-0.31	-0.16	0.04	-0.15	0.06	0.93	-0.27	-0.18	-0.05	-0.17	0.05	0.96	-0.32	-0.19	-0.02	-0.19	0.05	0.98
91624	NEXN	-0.07	0.16	0.36	0.16	0.07	0.90	0.05	0.18	0.32	0.18	0.06	0.96	-0.03	0.18	0.35	0.18	0.07	0.94
113675	SDSL	-0.05	0.16	0.37	0.16	0.07	0.91	0.04	0.16	0.34	0.17	0.06	0.92	-0.03	0.17	0.34	0.18	0.07	0.94
114787	GPRIN1	-0.33	-0.15	0.07	-0.16	0.07	0.91	-0.32	-0.18	-0.06	-0.18	0.06	0.94	-0.33	-0.18	-0.01	-0.18	0.06	0.96
114990	VASN	-0.27	-0.14	0.03	-0.13	0.05	0.90	-0.26	-0.15	-0.04	-0.15	0.05	0.94	-0.26	-0.15	0.01	-0.15	0.05	0.94
127833	SYT2	-0.11	0.20	0.42	0.20	0.09	0.94	0.08	0.23	0.48	0.23	0.07	0.99	-0.01	0.23	0.43	0.23	0.09	0.95
134548	SOWAHA	-0.36	-0.16	0.09	-0.17	0.07	0.91	-0.35	-0.18	-0.04	-0.18	0.06	0.98	-0.35	-0.18	0.02	-0.18	0.07	0.92
160760	PPTC7	-0.06	0.17	0.38	0.17	0.07	0.91	0.07	0.18	0.39	0.18	0.06	0.96	0.00	0.19	0.35	0.19	0.07	0.95
163782	KANK4	-0.11	0.18	0.36	0.18	0.07	0.95	0.08	0.21	0.32	0.21	0.06	1.00	0.04	0.21	0.38	0.22	0.06	0.98
192668	CYS1	-0.27	-0.15	0.02	-0.15	0.05	0.93	-0.25	-0.16	-0.04	-0.16	0.04	0.98	-0.29	-0.17	-0.01	-0.17	0.05	0.97
222537	HS3ST5	-0.12	0.17	0.35	0.17	0.07	0.91	0.05	0.20	0.36	0.20	0.06	0.98	0.00	0.20	0.36	0.19	0.07	0.94
286133	SCARA5	-0.42	-0.18	0.15	-0.18	0.09	0.90	-0.41	-0.22	-0.09	-0.21	0.07	1.00	-0.44	-0.22	0.05	-0.22	0.08	0.94
326624	RAB37	-0.10	0.18	0.42	0.18	0.08	0.92	0.06	0.21	0.42	0.20	0.07	0.96	-0.03	0.20	0.39	0.20	0.08	0.93
342667	STAC2	-0.10	0.19	0.43	0.19	0.09	0.93	0.07	0.22	0.45	0.22	0.07	0.98	-0.03	0.21	0.41	0.21	0.09	0.94
415116	PIM3	-0.08	0.17	0.36	0.17	0.07	0.91	0.05	0.18	0.36	0.18	0.06	0.96	-0.02	0.19	0.35	0.19	0.07	0.93
642273	FAM110C	-0.43	-0.18	0.14	-0.18	0.09	0.91	-0.40	-0.21	-0.05	-0.21	0.08	0.97	-0.44	-0.21	0.01	-0.21	0.09	0.94
654502	IQCJ	-0.30	-0.15	0.06	-0.15	0.06	0.93	-0.26	-0.15	0.01	-0.15	0.05	0.94	-0.35	-0.16	-0.01	-0.16	0.06	0.93

Abbreviations: rsFC, resting-state functional connectivity; L, left; A6cdl, caudal dorsolateral area 6; CNP, the Consortium for Neuropsychiatric Phenomics; SALD, the Southwest University Adult Lifespan Dataset; Min, minimum; Med, median; Max, maximum; SD, standard deviation.

Genes related to rsFC of the R-A6cdl in both the discovery and validation experiments

GeneID	Genesymbol	Discovery dataset						CNP dataset						SALD dataset					
		r					Percentage	*r*					Percentage	*r*					Percentage
		Min	Med	Max	Mean	SD		Min	Med	Max	Mean	SD		Min	Med	Max	Mean	SD	
55244	SLC47A1	-0.11	0.19	0.40	0.19	0.09	0.91	0.01	0.21	0.43	0.21	0.08	0.94	-0.06	0.20	0.40	0.20	0.09	0.91
163782	KANK4	-0.11	0.16	0.33	0.16	0.07	0.90	-0.03	0.18	0.31	0.18	0.07	0.91	-0.02	0.20	0.36	0.19	0.07	0.95

Abbreviations: rsFC, resting-state functional connectivity; R, right; A6cdl, caudal dorsolateral area 6; CNP, the Consortium for Neuropsychiatric Phenomics; SALD, the Southwest University Adult Lifespan Dataset; Min, minimum; Med, median; Max, maximum; SD, standard deviation.

Genes related to rsFC of the L-A4ul in both the discovery and validation experiments

GeneID	Genesymbol	Discovery dataset						CNP dataset						SALD dataset					
		r					Percentage	*r*					Percentage	*r*					Percentage
		Min	Med	Max	Mean	SD		Min	Med	Max	Mean	SD		Min	Med	Max	Mean	SD	
92	ACVR2A	-0.30	-0.16	-0.02	-0.16	0.05	0.95	-0.29	-0.18	-0.08	-0.18	0.05	1.00	-0.32	-0.19	-0.03	-0.19	0.05	0.99
98	ACYP2	-0.01	0.16	0.38	0.16	0.06	0.94	0.05	0.16	0.31	0.16	0.06	0.95	0.02	0.17	0.33	0.17	0.06	0.96
115	ADCY9	-0.05	0.17	0.45	0.17	0.08	0.91	0.00	0.17	0.33	0.17	0.07	0.94	0.01	0.19	0.38	0.19	0.07	0.97
133	ADM	-0.06	0.17	0.39	0.17	0.07	0.94	0.05	0.17	0.29	0.17	0.06	0.97	0.00	0.17	0.36	0.18	0.07	0.96
161	AP2A2	-0.34	-0.15	0.04	-0.15	0.06	0.93	-0.26	-0.14	-0.03	-0.14	0.05	0.94	-0.31	-0.16	0.01	-0.16	0.05	0.97
176	ACAN	-0.03	0.25	0.51	0.25	0.09	0.97	0.10	0.26	0.44	0.26	0.08	1.00	0.09	0.28	0.51	0.29	0.08	1.00
204	AK2	0.02	0.16	0.30	0.16	0.05	0.96	0.05	0.16	0.27	0.16	0.05	0.96	0.00	0.18	0.30	0.18	0.05	0.98
220	ALDH1A3	-0.04	0.17	0.47	0.17	0.08	0.91	0.01	0.16	0.37	0.17	0.07	0.92	-0.03	0.18	0.39	0.18	0.08	0.95
249	ALPL	-0.30	-0.14	0.03	-0.14	0.05	0.90	-0.25	-0.14	-0.03	-0.15	0.05	0.96	-0.26	-0.16	-0.03	-0.16	0.05	0.96
272	AMPD3	0.00	0.12	0.25	0.12	0.04	0.93	0.03	0.13	0.22	0.13	0.04	0.96	0.01	0.14	0.23	0.14	0.04	0.98
286	ANK1	-0.06	0.22	0.49	0.22	0.09	0.95	0.05	0.23	0.42	0.23	0.08	0.98	0.02	0.25	0.46	0.25	0.08	0.99
292	SLC25A5	0.01	0.15	0.30	0.15	0.06	0.92	0.05	0.16	0.31	0.16	0.05	0.99	0.02	0.16	0.29	0.16	0.06	0.96
308	ANXA5	0.00	0.14	0.25	0.14	0.04	0.96	0.04	0.15	0.23	0.15	0.04	0.98	0.03	0.16	0.28	0.16	0.04	0.98
320	APBA1	-0.25	-0.13	0.00	-0.14	0.04	0.95	-0.25	-0.16	-0.05	-0.16	0.04	0.98	-0.25	-0.16	-0.04	-0.16	0.04	0.98
341	APOC1	-0.34	-0.15	-0.01	-0.15	0.06	0.92	-0.29	-0.17	-0.04	-0.17	0.05	0.96	-0.33	-0.18	0.01	-0.17	0.06	0.95
367	AR	0.01	0.20	0.43	0.21	0.07	0.97	0.07	0.22	0.38	0.21	0.06	0.99	0.04	0.22	0.39	0.23	0.07	0.99
403	ARL3	-0.06	0.14	0.32	0.14	0.06	0.90	0.01	0.14	0.28	0.14	0.05	0.92	-0.01	0.15	0.34	0.15	0.06	0.94
430	ASCL2	-0.48	-0.21	0.06	-0.20	0.09	0.94	-0.40	-0.22	-0.05	-0.22	0.08	0.98	-0.43	-0.23	0.00	-0.24	0.08	0.99
445	ASS1	-0.41	-0.19	0.04	-0.19	0.07	0.95	-0.34	-0.20	-0.04	-0.20	0.06	0.99	-0.41	-0.22	-0.05	-0.22	0.07	0.99
460	ASTN1	-0.33	-0.17	-0.03	-0.17	0.06	0.96	-0.31	-0.19	-0.07	-0.19	0.05	0.99	-0.32	-0.20	-0.01	-0.20	0.06	0.99
481	ATP1B1	-0.03	0.17	0.36	0.17	0.07	0.93	0.05	0.18	0.31	0.18	0.06	0.96	0.03	0.19	0.34	0.19	0.06	0.99
483	ATP1B3	-0.02	0.15	0.31	0.15	0.06	0.92	0.01	0.16	0.26	0.16	0.05	0.96	0.02	0.16	0.30	0.16	0.06	0.96
492	ATP2B3	-0.05	0.16	0.37	0.16	0.07	0.91	0.03	0.16	0.30	0.17	0.06	0.97	0.01	0.17	0.36	0.17	0.07	0.96
493	ATP2B4	-0.48	-0.20	0.08	-0.20	0.09	0.95	-0.39	-0.20	0.00	-0.20	0.08	0.97	-0.43	-0.22	-0.01	-0.23	0.08	0.98
563	AZGP1	-0.02	0.13	0.29	0.13	0.05	0.93	0.02	0.14	0.25	0.14	0.04	0.97	0.01	0.15	0.30	0.15	0.05	0.97
586	BCAT1	0.03	0.17	0.33	0.17	0.06	0.96	0.05	0.18	0.32	0.18	0.05	0.98	0.05	0.19	0.31	0.19	0.06	0.98
613	BCR	-0.36	-0.15	0.05	-0.15	0.07	0.90	-0.30	-0.17	-0.03	-0.16	0.06	0.94	-0.35	-0.18	0.01	-0.18	0.06	0.97
631	BFSP1	-0.29	-0.14	0.03	-0.14	0.05	0.93	-0.25	-0.15	0.01	-0.15	0.05	0.96	-0.30	-0.18	-0.01	-0.18	0.05	0.99
715	C1R	-0.35	-0.16	0.05	-0.16	0.07	0.93	-0.33	-0.17	0.01	-0.17	0.06	0.95	-0.36	-0.20	-0.02	-0.20	0.06	0.98
716	C1S	-0.42	-0.16	0.13	-0.16	0.08	0.91	-0.32	-0.16	0.01	-0.16	0.07	0.90	-0.42	-0.17	0.03	-0.18	0.08	0.92
784	CACNB3	-0.34	-0.18	0.00	-0.17	0.05	0.98	-0.30	-0.19	-0.08	-0.18	0.05	1.00	-0.31	-0.19	-0.05	-0.19	0.05	0.99
793	CALB1	-0.42	-0.21	0.01	-0.21	0.07	0.97	-0.37	-0.22	-0.08	-0.22	0.07	1.00	-0.43	-0.23	-0.07	-0.24	0.07	0.99
808	CALM3	-0.45	-0.17	0.08	-0.17	0.08	0.92	-0.35	-0.18	-0.04	-0.18	0.07	0.96	-0.37	-0.20	0.02	-0.20	0.07	0.98
814	CAMK4	-0.35	-0.19	-0.03	-0.19	0.06	0.99	-0.31	-0.20	-0.06	-0.20	0.05	0.99	-0.35	-0.21	-0.04	-0.21	0.05	1.00
817	CAMK2D	-0.43	-0.22	0.01	-0.22	0.07	0.97	-0.40	-0.23	-0.06	-0.24	0.07	0.99	-0.43	-0.26	-0.02	-0.26	0.07	1.00
818	CAMK2G	-0.06	0.19	0.42	0.19	0.08	0.94	0.04	0.20	0.36	0.19	0.07	0.96	0.03	0.21	0.41	0.21	0.07	0.98
831	CAST	-0.01	0.14	0.32	0.14	0.06	0.91	0.00	0.15	0.28	0.15	0.05	0.91	-0.01	0.17	0.30	0.17	0.05	0.97
955	ENTPD6	-0.27	-0.14	0.00	-0.13	0.05	0.93	-0.24	-0.14	0.01	-0.13	0.04	0.98	-0.25	-0.15	0.01	-0.15	0.04	0.98
1002	CDH4	-0.36	-0.17	0.02	-0.17	0.06	0.95	-0.31	-0.18	-0.03	-0.18	0.06	0.95	-0.34	-0.19	0.00	-0.19	0.06	0.98
1006	CDH8	-0.40	-0.21	0.05	-0.21	0.07	0.97	-0.36	-0.23	-0.06	-0.22	0.06	0.99	-0.41	-0.23	-0.02	-0.23	0.06	0.99
1007	CDH9	-0.39	-0.17	0.05	-0.17	0.07	0.94	-0.32	-0.16	-0.03	-0.16	0.06	0.93	-0.39	-0.19	-0.01	-0.19	0.07	0.98
1027	CDKN1B	-0.02	0.15	0.30	0.15	0.05	0.94	0.03	0.16	0.29	0.15	0.05	0.96	-0.02	0.16	0.30	0.17	0.05	0.98
1031	CDKN2C	-0.02	0.15	0.34	0.15	0.06	0.93	0.04	0.14	0.25	0.14	0.05	0.93	0.04	0.17	0.30	0.17	0.05	0.98
1032	CDKN2D	-0.35	-0.15	0.05	-0.15	0.06	0.93	-0.28	-0.15	-0.03	-0.15	0.05	0.95	-0.34	-0.17	0.00	-0.18	0.06	0.97
1040	CDS1	-0.05	0.17	0.40	0.17	0.08	0.92	0.03	0.17	0.34	0.18	0.07	0.94	0.00	0.19	0.37	0.19	0.07	0.97
1050	CEBPA	-0.40	-0.18	0.07	-0.18	0.08	0.93	-0.36	-0.20	-0.04	-0.20	0.07	0.96	-0.39	-0.22	0.01	-0.22	0.07	0.99
1073	CFL2	0.01	0.16	0.30	0.16	0.05	0.95	0.03	0.17	0.28	0.16	0.05	0.99	-0.02	0.18	0.31	0.18	0.05	0.98
1176	AP3S1	-0.36	-0.14	0.09	-0.15	0.07	0.91	-0.31	-0.15	-0.04	-0.16	0.06	0.94	-0.34	-0.17	0.03	-0.17	0.07	0.96
1272	CNTN1	-0.41	-0.17	0.08	-0.18	0.07	0.94	-0.34	-0.17	-0.02	-0.18	0.07	0.96	-0.37	-0.20	0.01	-0.20	0.07	0.98
1294	COL7A1	-0.04	0.16	0.37	0.16	0.06	0.93	0.05	0.16	0.30	0.17	0.05	0.95	-0.02	0.18	0.33	0.18	0.06	0.98
1299	COL9A3	0.00	0.11	0.21	0.11	0.04	0.91	0.03	0.12	0.21	0.12	0.04	0.93	0.00	0.13	0.22	0.13	0.04	0.95
1300	COL10A1	-0.33	-0.15	0.00	-0.15	0.06	0.94	-0.29	-0.18	-0.05	-0.18	0.05	0.99	-0.31	-0.18	-0.01	-0.18	0.05	0.98
1381	CRABP1	-0.07	0.17	0.42	0.18	0.08	0.93	0.04	0.18	0.33	0.18	0.06	0.95	0.02	0.19	0.41	0.19	0.07	0.97
1397	CRIP2	-0.41	-0.16	0.11	-0.16	0.08	0.91	-0.33	-0.16	-0.01	-0.16	0.07	0.95	-0.37	-0.17	0.00	-0.18	0.07	0.95
1501	CTNND2	-0.36	-0.17	0.05	-0.17	0.07	0.94	-0.30	-0.18	-0.03	-0.17	0.05	0.96	-0.37	-0.19	-0.02	-0.19	0.06	0.97
1522	CTSZ	0.02	0.19	0.36	0.19	0.07	0.98	0.03	0.19	0.35	0.19	0.06	0.99	0.04	0.22	0.37	0.22	0.06	0.99
1607	DGKB	-0.37	-0.17	0.04	-0.18	0.07	0.95	-0.30	-0.18	-0.03	-0.18	0.06	0.96	-0.40	-0.20	-0.03	-0.20	0.07	0.98
1730	DIAPH2	-0.33	-0.16	0.02	-0.16	0.06	0.94	-0.27	-0.17	-0.03	-0.17	0.05	0.98	-0.29	-0.18	-0.04	-0.18	0.05	0.99
1755	DMBT1	-0.01	0.13	0.22	0.13	0.04	0.94	0.05	0.14	0.21	0.14	0.03	0.98	0.01	0.15	0.23	0.15	0.04	0.99
1809	DPYSL3	-0.48	-0.19	0.08	-0.19	0.09	0.93	-0.38	-0.19	-0.01	-0.19	0.08	0.94	-0.43	-0.21	0.00	-0.22	0.09	0.96
1893	ECM1	-0.02	0.20	0.43	0.20	0.07	0.95	0.07	0.21	0.38	0.21	0.06	0.99	0.02	0.22	0.38	0.22	0.07	0.98
1949	EFNB3	-0.46	-0.18	0.08	-0.18	0.08	0.92	-0.37	-0.18	-0.03	-0.18	0.08	0.93	-0.43	-0.20	0.01	-0.21	0.08	0.97
2027	ENO3	-0.02	0.13	0.24	0.12	0.04	0.92	0.06	0.14	0.23	0.14	0.04	0.99	0.02	0.14	0.27	0.14	0.04	0.98
2044	EPHA5	-0.32	-0.17	-0.05	-0.17	0.05	0.98	-0.30	-0.18	-0.07	-0.18	0.04	1.00	-0.31	-0.19	-0.04	-0.19	0.05	0.99
2070	EYA4	-0.04	0.20	0.43	0.20	0.07	0.96	0.05	0.20	0.37	0.20	0.06	0.97	0.01	0.21	0.41	0.22	0.07	0.99
2101	ESRRA	-0.04	0.21	0.47	0.21	0.08	0.96	0.05	0.22	0.39	0.22	0.07	0.97	0.04	0.24	0.43	0.24	0.08	0.99
2104	ESRRG	-0.06	0.23	0.51	0.23	0.09	0.96	0.06	0.24	0.44	0.24	0.08	0.98	0.02	0.25	0.48	0.26	0.08	0.99
2161	F12	-0.46	-0.21	0.06	-0.21	0.08	0.96	-0.39	-0.22	-0.06	-0.22	0.07	0.99	-0.41	-0.23	0.00	-0.24	0.07	1.00
2170	FABP3	-0.29	-0.14	0.05	-0.14	0.06	0.91	-0.26	-0.16	-0.04	-0.16	0.05	0.98	-0.29	-0.16	-0.01	-0.16	0.05	0.98
2173	FABP7	-0.45	-0.19	0.05	-0.19	0.09	0.93	-0.36	-0.19	-0.04	-0.20	0.07	0.99	-0.42	-0.20	0.00	-0.21	0.08	0.97
2182	ACSL4	-0.36	-0.15	0.04	-0.15	0.06	0.93	-0.29	-0.15	-0.02	-0.15	0.06	0.91	-0.33	-0.17	-0.01	-0.17	0.06	0.96
2230	FDX1	0.00	0.12	0.24	0.12	0.05	0.91	0.02	0.12	0.25	0.12	0.04	0.90	0.01	0.13	0.24	0.13	0.05	0.91
2254	FGF9	-0.06	0.19	0.44	0.19	0.08	0.93	0.03	0.20	0.38	0.20	0.07	0.96	0.00	0.21	0.41	0.21	0.08	0.98
2289	FKBP5	-0.07	0.16	0.39	0.16	0.07	0.92	0.02	0.17	0.32	0.16	0.07	0.90	0.01	0.20	0.39	0.19	0.07	0.97
2322	FLT3	0.02	0.19	0.37	0.19	0.06	0.96	0.04	0.19	0.35	0.19	0.06	0.98	0.04	0.20	0.33	0.20	0.06	0.99
2558	GABRA5	-0.52	-0.19	0.08	-0.19	0.09	0.93	-0.41	-0.19	-0.02	-0.19	0.08	0.93	-0.43	-0.21	0.01	-0.22	0.09	0.98
2560	GABRB1	-0.46	-0.19	0.06	-0.20	0.08	0.95	-0.36	-0.20	-0.03	-0.20	0.07	0.96	-0.42	-0.21	0.02	-0.22	0.08	0.99
2562	GABRB3	-0.31	-0.15	0.03	-0.15	0.05	0.96	-0.26	-0.15	-0.04	-0.15	0.05	0.93	-0.30	-0.17	0.00	-0.17	0.05	0.98
2565	GABRG1	-0.35	-0.16	0.00	-0.17	0.06	0.94	-0.26	-0.17	-0.05	-0.17	0.05	0.96	-0.34	-0.19	-0.04	-0.19	0.06	0.99
2620	GAS2	-0.01	0.17	0.38	0.18	0.07	0.94	0.05	0.17	0.32	0.18	0.06	0.97	0.04	0.19	0.37	0.19	0.07	0.97
2632	GBE1	-0.05	0.15	0.31	0.15	0.06	0.93	0.01	0.15	0.28	0.15	0.05	0.97	0.04	0.17	0.31	0.17	0.05	0.98
2634	GBP2	-0.04	0.13	0.26	0.13	0.05	0.90	0.00	0.15	0.23	0.14	0.04	0.95	-0.04	0.14	0.24	0.14	0.04	0.97
2690	GHR	-0.29	-0.14	0.01	-0.14	0.05	0.92	-0.27	-0.15	-0.07	-0.16	0.05	0.99	-0.30	-0.17	-0.01	-0.17	0.05	0.98
2742	GLRA2	-0.43	-0.20	0.02	-0.20	0.08	0.94	-0.39	-0.21	-0.03	-0.22	0.07	0.98	-0.42	-0.23	0.00	-0.24	0.08	0.99
2764	GMFB	-0.41	-0.17	0.11	-0.17	0.08	0.93	-0.35	-0.16	0.00	-0.17	0.07	0.94	-0.40	-0.20	0.02	-0.20	0.07	0.97
2766	GMPR	0.01	0.17	0.35	0.17	0.06	0.97	0.08	0.17	0.28	0.17	0.05	1.00	0.02	0.19	0.33	0.18	0.05	0.99
2778	GNAS	0.00	0.14	0.32	0.15	0.06	0.92	0.06	0.16	0.29	0.16	0.05	0.97	0.03	0.16	0.30	0.16	0.05	0.96
2786	GNG4	-0.48	-0.17	0.08	-0.17	0.09	0.92	-0.35	-0.17	-0.01	-0.17	0.08	0.92	-0.40	-0.20	0.00	-0.21	0.08	0.97
2790	GNG10	-0.32	-0.14	0.01	-0.14	0.06	0.90	-0.28	-0.14	0.00	-0.15	0.06	0.90	-0.32	-0.16	0.00	-0.16	0.06	0.96
2820	GPD2	-0.31	-0.14	0.01	-0.14	0.06	0.93	-0.28	-0.15	0.00	-0.15	0.05	0.94	-0.34	-0.16	0.02	-0.16	0.06	0.96
2823	GPM6A	-0.35	-0.18	-0.02	-0.18	0.06	0.98	-0.29	-0.18	-0.06	-0.18	0.05	0.98	-0.33	-0.21	-0.06	-0.21	0.06	0.99
2830	GPR6	-0.35	-0.20	-0.02	-0.20	0.06	0.98	-0.34	-0.21	-0.12	-0.21	0.05	1.00	-0.35	-0.22	-0.06	-0.22	0.06	0.99
2888	GRB14	-0.34	-0.15	0.07	-0.15	0.07	0.90	-0.30	-0.18	0.02	-0.17	0.06	0.90	-0.35	-0.17	0.02	-0.17	0.07	0.92
2890	GRIA1	-0.42	-0.16	0.06	-0.17	0.08	0.91	-0.33	-0.17	-0.03	-0.17	0.07	0.92	-0.37	-0.18	0.02	-0.19	0.08	0.96
2898	GRIK2	-0.38	-0.17	0.01	-0.17	0.07	0.96	-0.29	-0.17	-0.03	-0.16	0.06	0.94	-0.35	-0.20	-0.05	-0.20	0.06	0.98
2911	GRM1	-0.38	-0.18	0.05	-0.18	0.07	0.94	-0.32	-0.17	-0.02	-0.18	0.06	0.97	-0.36	-0.20	-0.03	-0.20	0.06	0.99
2918	GRM8	-0.32	-0.16	0.01	-0.16	0.06	0.94	-0.29	-0.17	-0.03	-0.17	0.05	0.97	-0.32	-0.19	-0.02	-0.19	0.06	0.98
2937	GSS	-0.34	-0.15	0.07	-0.15	0.06	0.93	-0.28	-0.16	-0.03	-0.15	0.06	0.92	-0.30	-0.17	-0.01	-0.17	0.06	0.98
2947	GSTM3	-0.36	-0.15	0.05	-0.15	0.07	0.91	-0.28	-0.15	0.00	-0.15	0.05	0.93	-0.33	-0.16	0.00	-0.16	0.06	0.95
2952	GSTT1	-0.01	0.22	0.47	0.22	0.08	0.96	0.08	0.23	0.41	0.23	0.07	1.00	0.06	0.25	0.44	0.25	0.08	0.99
3067	HDC	-0.40	-0.18	0.05	-0.18	0.07	0.96	-0.32	-0.20	-0.05	-0.19	0.06	0.99	-0.37	-0.21	-0.04	-0.20	0.06	0.99
3145	HMBS	-0.04	0.16	0.36	0.16	0.06	0.93	0.05	0.16	0.29	0.16	0.05	0.97	0.01	0.17	0.34	0.18	0.06	0.97
3156	HMGCR	-0.31	-0.14	0.06	-0.15	0.06	0.92	-0.27	-0.14	-0.04	-0.14	0.05	0.91	-0.32	-0.16	0.00	-0.16	0.06	0.96
3231	HOXD1	-0.02	0.13	0.23	0.13	0.04	0.93	0.05	0.14	0.21	0.14	0.03	0.98	0.01	0.15	0.26	0.15	0.04	0.99
3269	HRH1	-0.40	-0.16	0.10	-0.16	0.07	0.92	-0.30	-0.17	-0.02	-0.17	0.06	0.94	-0.39	-0.17	-0.01	-0.18	0.07	0.96
3290	HSD11B1	0.00	0.18	0.34	0.18	0.06	0.96	0.09	0.21	0.32	0.20	0.05	1.00	0.03	0.22	0.37	0.22	0.06	0.99
3295	HSD17B4	-0.01	0.12	0.22	0.12	0.04	0.92	0.05	0.12	0.20	0.12	0.03	0.97	0.00	0.14	0.23	0.13	0.04	0.96
3316	HSPB2	-0.02	0.16	0.33	0.16	0.06	0.94	0.05	0.16	0.29	0.16	0.05	0.95	0.04	0.19	0.34	0.19	0.06	0.98
3338	DNAJC4	-0.04	0.16	0.40	0.16	0.07	0.92	0.01	0.15	0.30	0.16	0.06	0.92	0.01	0.18	0.36	0.18	0.07	0.97
3350	HTR1A	-0.39	-0.18	0.04	-0.18	0.07	0.94	-0.34	-0.19	0.02	-0.18	0.07	0.97	-0.38	-0.20	0.02	-0.20	0.07	0.99
3358	HTR2C	-0.39	-0.16	0.03	-0.17	0.07	0.93	-0.35	-0.17	0.07	-0.17	0.07	0.94	-0.39	-0.21	-0.01	-0.21	0.07	0.98
3396	MRPL58	-0.01	0.15	0.32	0.15	0.06	0.91	0.03	0.15	0.29	0.15	0.05	0.91	0.02	0.16	0.32	0.16	0.06	0.95
3423	IDS	-0.28	-0.12	0.00	-0.12	0.04	0.90	-0.22	-0.13	-0.04	-0.13	0.04	0.92	-0.24	-0.14	0.03	-0.14	0.04	0.97
3551	IKBKB	-0.06	0.14	0.31	0.13	0.06	0.90	0.02	0.14	0.27	0.14	0.05	0.94	-0.03	0.15	0.31	0.16	0.05	0.96
3598	IL13RA2	-0.46	-0.18	0.03	-0.19	0.08	0.93	-0.38	-0.19	-0.01	-0.19	0.07	0.95	-0.41	-0.22	0.00	-0.22	0.08	0.98
3624	INHBA	0.00	0.17	0.32	0.17	0.05	0.97	0.01	0.18	0.30	0.18	0.05	0.98	0.05	0.20	0.33	0.20	0.05	0.99
3632	INPP5A	-0.02	0.16	0.34	0.16	0.06	0.93	0.05	0.18	0.30	0.17	0.05	0.97	0.04	0.18	0.33	0.18	0.06	0.99
3653	IPW	-0.05	0.18	0.47	0.19	0.08	0.93	0.01	0.19	0.37	0.19	0.07	0.96	0.02	0.21	0.39	0.21	0.07	0.98
3679	ITGA7	0.01	0.15	0.26	0.15	0.05	0.96	0.07	0.17	0.29	0.17	0.04	1.00	0.04	0.18	0.30	0.18	0.04	0.99
3688	ITGB1	0.00	0.14	0.25	0.14	0.04	0.96	0.06	0.16	0.24	0.16	0.04	0.99	0.00	0.17	0.26	0.17	0.04	0.99
3706	ITPKA	-0.29	-0.14	0.03	-0.14	0.05	0.93	-0.24	-0.14	-0.01	-0.13	0.05	0.93	-0.27	-0.15	0.00	-0.14	0.05	0.95
3736	KCNA1	-0.01	0.20	0.44	0.20	0.08	0.94	0.07	0.21	0.39	0.21	0.07	0.99	0.03	0.23	0.42	0.23	0.08	0.98
3738	KCNA3	-0.46	-0.20	0.10	-0.21	0.08	0.96	-0.36	-0.20	-0.02	-0.20	0.07	0.96	-0.48	-0.23	-0.04	-0.23	0.08	0.98
3739	KCNA4	-0.21	-0.12	-0.01	-0.12	0.04	0.94	-0.20	-0.12	-0.02	-0.12	0.04	0.92	-0.24	-0.13	-0.01	-0.13	0.04	0.96
3741	KCNA5	-0.45	-0.18	0.10	-0.18	0.09	0.93	-0.35	-0.19	-0.03	-0.19	0.07	0.96	-0.42	-0.19	0.02	-0.20	0.09	0.95
3746	KCNC1	0.00	0.16	0.37	0.16	0.07	0.92	0.04	0.17	0.33	0.17	0.06	0.96	0.02	0.17	0.33	0.18	0.06	0.97
3755	KCNG1	-0.48	-0.21	0.02	-0.21	0.09	0.94	-0.42	-0.23	-0.06	-0.23	0.08	0.99	-0.44	-0.23	-0.02	-0.24	0.08	0.98
3783	KCNN4	0.00	0.17	0.33	0.17	0.06	0.97	0.06	0.18	0.29	0.18	0.05	0.99	0.05	0.18	0.33	0.18	0.06	0.99
3787	KCNS1	-0.03	0.17	0.42	0.18	0.08	0.91	0.02	0.19	0.36	0.19	0.07	0.95	0.00	0.20	0.38	0.20	0.08	0.96
3827	KNG1	-0.04	0.17	0.38	0.17	0.07	0.92	0.01	0.17	0.31	0.17	0.06	0.96	0.03	0.19	0.35	0.20	0.06	0.99
3902	LAG3	-0.10	0.20	0.49	0.21	0.09	0.93	0.02	0.22	0.41	0.21	0.08	0.98	0.02	0.22	0.48	0.23	0.09	0.97
3910	LAMA4	-0.07	0.19	0.39	0.19	0.07	0.96	0.07	0.19	0.31	0.19	0.06	0.99	0.03	0.21	0.39	0.21	0.06	0.98
4062	LY6H	-0.52	-0.22	0.07	-0.22	0.09	0.96	-0.42	-0.22	-0.04	-0.23	0.08	0.99	-0.48	-0.24	-0.04	-0.26	0.08	0.99
4082	MARCKS	-0.42	-0.20	-0.01	-0.20	0.07	0.96	-0.39	-0.21	-0.06	-0.21	0.07	0.98	-0.41	-0.24	-0.04	-0.24	0.07	0.99
4092	SMAD7	-0.04	0.15	0.36	0.15	0.06	0.91	0.04	0.17	0.29	0.17	0.05	0.97	0.02	0.19	0.38	0.19	0.06	0.98
4157	MC1R	-0.11	0.16	0.40	0.16	0.08	0.90	0.01	0.16	0.33	0.16	0.07	0.93	-0.01	0.18	0.42	0.18	0.07	0.95
4168	MCF2	-0.04	0.17	0.37	0.17	0.07	0.91	0.03	0.17	0.32	0.17	0.06	0.93	0.01	0.18	0.34	0.18	0.07	0.96
4257	MGST1	-0.40	-0.17	0.10	-0.17	0.08	0.91	-0.33	-0.17	-0.02	-0.17	0.06	0.95	-0.39	-0.18	0.04	-0.19	0.08	0.95

4258	MGST2	-0.04	0.20	0.44	0.20	0.08	0.96	0.03	0.22	0.39	0.22	0.07	0.99	0.05	0.24	0.43	0.24	0.07	0.99
4286	MITF	0.03	0.19	0.34	0.19	0.06	0.98	0.07	0.20	0.29	0.19	0.05	1.00	0.02	0.20	0.35	0.21	0.06	0.99
4324	MMP15	-0.05	0.16	0.32	0.16	0.06	0.96	0.03	0.17	0.29	0.17	0.05	0.96	0.04	0.19	0.35	0.19	0.05	0.99
4337	MOCS1	-0.03	0.13	0.27	0.13	0.05	0.91	0.04	0.13	0.24	0.13	0.04	0.93	0.00	0.13	0.24	0.14	0.05	0.95
4345	CD200	-0.25	-0.14	-0.04	-0.14	0.04	0.96	-0.25	-0.14	-0.06	-0.15	0.04	0.99	-0.29	-0.16	-0.05	-0.16	0.04	0.98
4354	MPP1	-0.02	0.19	0.42	0.19	0.08	0.94	0.06	0.20	0.36	0.20	0.07	0.98	0.04	0.22	0.38	0.22	0.07	0.99
4599	MX1	0.00	0.20	0.41	0.20	0.07	0.96	0.03	0.20	0.36	0.21	0.06	0.99	0.05	0.24	0.39	0.24	0.07	0.99
4602	MYB	-0.36	-0.17	0.07	-0.17	0.07	0.92	-0.31	-0.16	0.03	-0.16	0.06	0.92	-0.36	-0.18	-0.01	-0.19	0.07	0.98
4609	MYC	0.01	0.20	0.39	0.20	0.07	0.98	0.08	0.21	0.37	0.21	0.06	1.00	0.05	0.22	0.36	0.22	0.06	0.99
4616	GADD45B	-0.02	0.14	0.30	0.14	0.06	0.91	0.06	0.15	0.29	0.15	0.05	0.96	-0.01	0.17	0.29	0.16	0.05	0.97
4625	MYH7	-0.41	-0.17	0.06	-0.17	0.07	0.93	-0.34	-0.18	-0.03	-0.18	0.07	0.94	-0.38	-0.20	-0.03	-0.21	0.07	0.98
4638	MYLK	-0.01	0.16	0.27	0.16	0.05	0.97	0.06	0.17	0.26	0.17	0.04	0.99	0.06	0.18	0.28	0.18	0.04	0.99
4703	NEB	-0.01	0.21	0.46	0.22	0.08	0.98	0.10	0.24	0.40	0.24	0.07	1.00	0.08	0.25	0.46	0.25	0.07	1.00
4715	NDUFB9	-0.03	0.15	0.32	0.15	0.06	0.93	0.03	0.15	0.30	0.15	0.05	0.95	-0.01	0.16	0.34	0.16	0.06	0.92
4744	NEFH	0.00	0.20	0.42	0.20	0.08	0.94	0.08	0.21	0.38	0.21	0.06	1.00	0.03	0.22	0.41	0.22	0.07	0.98
4751	NEK2	-0.44	-0.20	0.08	-0.20	0.08	0.96	-0.37	-0.21	-0.06	-0.21	0.07	0.98	-0.45	-0.23	-0.05	-0.23	0.08	0.99
4753	NELL2	-0.29	-0.14	0.00	-0.14	0.05	0.94	-0.25	-0.15	-0.03	-0.14	0.05	0.93	-0.28	-0.16	-0.01	-0.16	0.05	0.98
4782	NFIC	-0.01	0.15	0.35	0.15	0.06	0.91	0.02	0.15	0.30	0.15	0.05	0.95	0.03	0.17	0.32	0.17	0.06	0.98
4824	NKX3-1	-0.07	0.15	0.36	0.15	0.07	0.91	0.01	0.15	0.29	0.15	0.06	0.90	-0.01	0.17	0.35	0.17	0.06	0.96
4856	NOV	-0.48	-0.19	0.11	-0.20	0.09	0.93	-0.38	-0.21	-0.04	-0.21	0.07	0.97	-0.47	-0.22	-0.03	-0.23	0.09	0.99
4878	NPPA	-0.35	-0.18	0.01	-0.18	0.06	0.95	-0.32	-0.16	0.05	-0.17	0.06	0.96	-0.37	-0.20	-0.03	-0.21	0.06	0.98
4885	NPTX2	-0.30	-0.15	0.04	-0.15	0.06	0.91	-0.28	-0.16	0.03	-0.16	0.06	0.94	-0.32	-0.17	0.00	-0.17	0.06	0.95
4886	NPY1R	-0.46	-0.19	0.06	-0.20	0.08	0.95	-0.34	-0.18	-0.02	-0.19	0.07	0.96	-0.41	-0.21	-0.01	-0.21	0.07	0.98
4889	NPY5R	-0.28	-0.15	0.00	-0.15	0.05	0.96	-0.24	-0.16	-0.02	-0.15	0.05	0.96	-0.29	-0.17	-0.02	-0.16	0.05	0.98
4968	OGG1	-0.28	-0.14	-0.01	-0.14	0.05	0.92	-0.28	-0.16	-0.04	-0.15	0.05	0.97	-0.29	-0.17	0.01	-0.17	0.05	0.98
4988	OPRM1	-0.44	-0.18	0.12	-0.18	0.08	0.93	-0.35	-0.18	-0.04	-0.19	0.07	0.94	-0.43	-0.20	0.00	-0.20	0.08	0.98
5064	PALM	-0.27	-0.14	0.00	-0.13	0.05	0.91	-0.24	-0.14	-0.03	-0.14	0.04	0.93	-0.27	-0.14	-0.03	-0.15	0.05	0.95
5082	PDCL	0.01	0.13	0.26	0.13	0.04	0.93	0.02	0.13	0.23	0.13	0.04	0.94	0.04	0.15	0.25	0.15	0.04	0.99
5090	PBX3	-0.02	0.14	0.30	0.14	0.05	0.94	0.05	0.15	0.25	0.15	0.04	0.99	0.01	0.15	0.27	0.15	0.05	0.95
5091	PC	0.01	0.16	0.31	0.15	0.05	0.94	0.04	0.15	0.28	0.16	0.05	0.97	0.04	0.17	0.32	0.17	0.05	0.98
5116	PCNT	-0.08	0.18	0.41	0.18	0.07	0.94	0.05	0.20	0.36	0.20	0.06	0.99	0.03	0.21	0.43	0.21	0.07	0.98
5119	CHMP1A	-0.39	-0.15	0.06	-0.15	0.07	0.91	-0.29	-0.16	-0.03	-0.15	0.06	0.92	-0.32	-0.16	-0.01	-0.16	0.07	0.95
5121	PCP4	-0.01	0.18	0.37	0.19	0.07	0.95	0.06	0.20	0.34	0.20	0.06	0.99	0.04	0.21	0.37	0.21	0.06	0.99
5138	PDE2A	-0.36	-0.17	0.03	-0.17	0.07	0.94	-0.33	-0.17	-0.03	-0.17	0.06	0.94	-0.35	-0.19	-0.01	-0.19	0.06	0.98
5141	PDE4A	-0.04	0.21	0.45	0.21	0.08	0.94	0.04	0.21	0.38	0.21	0.07	0.99	0.02	0.23	0.42	0.23	0.07	0.99
5176	SERPINF1	-0.36	-0.21	0.01	-0.20	0.06	0.97	-0.32	-0.21	-0.01	-0.21	0.06	0.99	-0.36	-0.23	-0.04	-0.23	0.06	0.99
5184	PEPD	0.01	0.17	0.33	0.17	0.06	0.96	0.04	0.17	0.28	0.17	0.05	0.98	0.02	0.19	0.31	0.19	0.05	0.99
5256	PHKA2	-0.10	0.16	0.37	0.15	0.07	0.90	0.00	0.17	0.33	0.16	0.06	0.95	-0.04	0.18	0.36	0.18	0.06	0.97
5272	SERPINB9	-0.01	0.18	0.40	0.18	0.07	0.94	0.04	0.18	0.34	0.18	0.07	0.96	0.02	0.20	0.36	0.20	0.07	0.97
5292	PIM1	-0.07	0.16	0.38	0.16	0.08	0.91	0.02	0.17	0.33	0.17	0.06	0.93	-0.02	0.19	0.36	0.18	0.07	0.96
5310	PKD1	-0.06	0.14	0.33	0.14	0.06	0.91	0.04	0.15	0.27	0.16	0.05	0.95	0.01	0.16	0.31	0.16	0.06	0.97
5330	PLCB2	-0.38	-0.16	0.06	-0.15	0.07	0.93	-0.32	-0.17	-0.02	-0.17	0.06	0.94	-0.32	-0.18	0.03	-0.18	0.06	0.98
5332	PLCB4	-0.02	0.17	0.37	0.17	0.07	0.92	0.02	0.17	0.32	0.17	0.06	0.97	0.04	0.19	0.34	0.20	0.06	0.98
5409	PNMT	-0.46	-0.22	0.02	-0.23	0.08	0.96	-0.42	-0.23	-0.06	-0.23	0.08	0.99	-0.44	-0.26	-0.05	-0.26	0.08	0.99
5412	UBL3	0.00	0.18	0.34	0.18	0.06	0.96	0.03	0.19	0.31	0.19	0.06	0.97	0.04	0.20	0.34	0.20	0.06	0.98
5453	POU3F1	-0.02	0.14	0.30	0.14	0.06	0.91	-0.05	0.15	0.26	0.14	0.05	0.92	0.00	0.17	0.31	0.17	0.06	0.97
5467	PPARD	-0.12	0.15	0.41	0.15	0.07	0.91	-0.01	0.15	0.31	0.16	0.07	0.91	0.00	0.18	0.38	0.18	0.07	0.97
5480	PPIC	-0.01	0.12	0.27	0.12	0.05	0.92	0.02	0.13	0.22	0.13	0.04	0.95	0.02	0.15	0.25	0.15	0.04	0.97
5547	PRCP	-0.01	0.16	0.30	0.16	0.05	0.96	0.05	0.16	0.30	0.16	0.05	0.97	0.04	0.18	0.30	0.18	0.05	0.98
5557	PRIM1	-0.05	0.15	0.34	0.15	0.06	0.91	0.02	0.15	0.28	0.15	0.06	0.90	-0.01	0.15	0.34	0.16	0.06	0.94
5569	PKIA	-0.49	-0.21	0.08	-0.21	0.09	0.95	-0.40	-0.22	-0.04	-0.22	0.08	0.98	-0.45	-0.24	0.00	-0.25	0.08	0.99
5570	PKIB	-0.32	-0.14	0.03	-0.14	0.06	0.92	-0.25	-0.15	0.02	-0.15	0.05	0.95	-0.30	-0.18	-0.02	-0.18	0.05	0.99
5579	PRKCB	-0.35	-0.16	0.07	-0.16	0.06	0.93	-0.28	-0.14	-0.01	-0.15	0.05	0.92	-0.33	-0.17	-0.01	-0.18	0.06	0.96
5580	PRKCD	-0.49	-0.21	0.10	-0.22	0.09	0.95	-0.44	-0.22	-0.04	-0.23	0.08	0.98	-0.47	-0.24	0.01	-0.25	0.09	0.99
5582	PRKCG	-0.44	-0.19	0.06	-0.20	0.08	0.94	-0.39	-0.20	-0.04	-0.21	0.07	0.98	-0.41	-0.22	0.00	-0.22	0.08	0.98
5594	MAPK1	-0.36	-0.15	0.01	-0.16	0.06	0.94	-0.30	-0.16	-0.03	-0.17	0.06	0.95	-0.34	-0.18	0.04	-0.18	0.06	0.98
5606	MAP2K3	-0.03	0.16	0.36	0.16	0.06	0.93	0.02	0.18	0.30	0.17	0.06	0.96	0.02	0.19	0.34	0.19	0.06	0.97
5733	PTGER3	-0.52	-0.20	0.06	-0.20	0.09	0.94	-0.41	-0.19	-0.01	-0.20	0.08	0.97	-0.43	-0.22	0.00	-0.22	0.08	0.98
5774	PTPN3	-0.29	-0.15	-0.02	-0.15	0.05	0.96	-0.29	-0.17	-0.04	-0.17	0.04	0.99	-0.31	-0.18	-0.07	-0.18	0.04	1.00
5786	PTPRA	-0.41	-0.18	0.03	-0.18	0.08	0.92	-0.33	-0.18	-0.01	-0.18	0.07	0.93	-0.38	-0.20	0.02	-0.20	0.07	0.97
5792	PTPRF	-0.41	-0.16	0.09	-0.17	0.08	0.91	-0.31	-0.16	0.01	-0.16	0.07	0.93	-0.39	-0.17	0.00	-0.18	0.07	0.95
5794	PTPRH	0.01	0.15	0.27	0.15	0.04	0.97	0.05	0.17	0.24	0.16	0.04	0.99	-0.01	0.17	0.27	0.17	0.04	0.99
5800	PTPRO	-0.24	-0.13	0.01	-0.13	0.04	0.94	-0.21	-0.14	-0.05	-0.14	0.04	0.95	-0.24	-0.15	-0.03	-0.15	0.04	0.98
5801	PTPRR	-0.36	-0.17	0.00	-0.17	0.07	0.93	-0.34	-0.18	-0.06	-0.19	0.06	0.97	-0.40	-0.21	-0.02	-0.21	0.07	0.98
5806	PTX3	-0.03	0.15	0.35	0.15	0.06	0.92	0.03	0.15	0.28	0.15	0.05	0.93	-0.01	0.16	0.33	0.16	0.06	0.95
5816	PVALB	-0.02	0.18	0.44	0.18	0.08	0.92	0.02	0.19	0.36	0.19	0.07	0.95	0.01	0.20	0.39	0.21	0.08	0.96
5860	QDPR	0.00	0.13	0.24	0.13	0.04	0.96	0.01	0.15	0.25	0.15	0.04	0.99	0.02	0.16	0.25	0.16	0.04	0.99
5874	RAB27B	-0.47	-0.21	0.05	-0.21	0.08	0.96	-0.40	-0.22	-0.06	-0.22	0.07	0.99	-0.43	-0.24	0.00	-0.24	0.07	0.99
5912	RAP2B	-0.43	-0.18	0.06	-0.19	0.08	0.94	-0.38	-0.19	-0.02	-0.19	0.07	0.98	-0.42	-0.21	0.01	-0.22	0.07	0.99
5937	RBMS1	-0.04	0.18	0.39	0.18	0.08	0.93	0.00	0.20	0.34	0.20	0.07	0.98	0.01	0.22	0.38	0.22	0.07	0.99
5950	RBP4	-0.51	-0.20	0.13	-0.20	0.09	0.94	-0.42	-0.20	-0.04	-0.21	0.08	0.97	-0.49	-0.22	-0.02	-0.24	0.09	0.98
5954	RCN1	-0.37	-0.16	0.06	-0.16	0.07	0.92	-0.29	-0.17	-0.01	-0.17	0.05	0.95	-0.35	-0.18	-0.02	-0.18	0.06	0.96
5961	PRPH2	-0.32	-0.17	0.02	-0.17	0.06	0.96	-0.30	-0.18	0.04	-0.18	0.06	0.99	-0.35	-0.20	-0.02	-0.20	0.06	0.99
5979	RET	-0.02	0.18	0.42	0.19	0.08	0.94	0.03	0.19	0.36	0.19	0.07	0.96	0.02	0.20	0.37	0.20	0.07	0.98
5991	RFX3	-0.24	-0.13	0.00	-0.13	0.04	0.92	-0.23	-0.13	-0.04	-0.13	0.04	0.94	-0.23	-0.14	-0.04	-0.13	0.04	0.96
5993	RFX5	-0.03	0.18	0.42	0.18	0.07	0.94	0.04	0.19	0.35	0.19	0.07	0.95	0.02	0.20	0.37	0.20	0.07	0.98
6017	RLBP1	-0.32	-0.16	0.01	-0.16	0.06	0.95	-0.23	-0.16	-0.06	-0.16	0.04	0.99	-0.30	-0.17	-0.03	-0.17	0.05	0.98
6118	RPA2	-0.29	-0.13	0.02	-0.13	0.05	0.91	-0.27	-0.13	0.01	-0.13	0.05	0.90	-0.29	-0.15	0.01	-0.15	0.05	0.95
6122	RPL3	-0.05	0.14	0.32	0.14	0.06	0.91	0.01	0.14	0.26	0.14	0.05	0.94	0.00	0.15	0.29	0.15	0.06	0.94
6272	SORT1	0.00	0.16	0.31	0.16	0.06	0.95	0.06	0.18	0.28	0.17	0.05	0.98	0.02	0.19	0.33	0.19	0.05	0.99
6320	CLEC11A	0.00	0.12	0.24	0.12	0.04	0.91	0.02	0.13	0.23	0.13	0.04	0.96	0.02	0.14	0.25	0.14	0.04	0.95
6323	SCN1A	-0.09	0.18	0.45	0.18	0.09	0.91	0.01	0.19	0.38	0.19	0.08	0.93	-0.02	0.21	0.42	0.21	0.08	0.98
6324	SCN1B	-0.06	0.20	0.49	0.20	0.09	0.93	0.02	0.21	0.39	0.20	0.08	0.95	0.00	0.22	0.44	0.23	0.09	0.98
6326	SCN2A	-0.27	-0.15	-0.03	-0.15	0.05	0.94	-0.25	-0.15	-0.05	-0.15	0.04	0.97	-0.30	-0.17	-0.04	-0.16	0.05	0.98
6330	SCN4B	0.04	0.24	0.48	0.24	0.08	0.98	0.10	0.25	0.44	0.25	0.07	1.00	0.08	0.27	0.45	0.27	0.08	1.00
6451	SH3BGRL	-0.05	0.16	0.36	0.16	0.07	0.91	0.01	0.16	0.31	0.16	0.06	0.94	0.01	0.17	0.33	0.18	0.06	0.97
6509	SLC1A4	-0.30	-0.13	0.03	-0.14	0.05	0.90	-0.23	-0.14	-0.01	-0.14	0.05	0.93	-0.28	-0.14	0.02	-0.15	0.06	0.93
6533	SLC6A6	-0.03	0.16	0.35	0.16	0.06	0.91	0.06	0.17	0.29	0.16	0.05	0.97	0.00	0.18	0.32	0.18	0.06	0.96
6541	SLC7A1	-0.01	0.12	0.23	0.12	0.04	0.93	0.05	0.12	0.20	0.12	0.04	0.95	0.01	0.13	0.23	0.13	0.04	0.95
6543	SLC8A2	-0.34	-0.17	-0.02	-0.17	0.06	0.96	-0.29	-0.17	-0.05	-0.17	0.05	0.97	-0.31	-0.19	-0.03	-0.19	0.05	0.98
6545	SLC7A4	-0.43	-0.16	0.08	-0.16	0.07	0.91	-0.31	-0.16	-0.01	-0.16	0.07	0.92	-0.36	-0.18	0.02	-0.19	0.07	0.98
6567	SLC16A2	-0.41	-0.17	0.05	-0.17	0.08	0.91	-0.35	-0.17	0.00	-0.17	0.07	0.97	-0.38	-0.19	0.02	-0.19	0.08	0.95
6585	SLIT1	-0.50	-0.19	0.09	-0.19	0.09	0.94	-0.38	-0.19	0.00	-0.19	0.08	0.96	-0.42	-0.21	0.02	-0.22	0.08	0.98
6603	SMARCD2	-0.04	0.14	0.32	0.14	0.06	0.91	0.01	0.13	0.29	0.14	0.06	0.92	-0.02	0.16	0.30	0.16	0.06	0.96
6646	SOAT1	-0.06	0.18	0.37	0.18	0.07	0.94	-0.01	0.18	0.34	0.18	0.06	0.97	0.01	0.21	0.36	0.20	0.06	0.98
6676	SPAG4	-0.09	0.16	0.39	0.16	0.07	0.92	0.02	0.17	0.33	0.17	0.06	0.96	-0.02	0.19	0.35	0.19	0.07	0.98
6696	SPP1	-0.01	0.13	0.24	0.13	0.04	0.93	0.04	0.14	0.22	0.14	0.04	0.99	0.02	0.15	0.25	0.15	0.04	0.98
6751	SSTR1	-0.50	-0.19	0.12	-0.19	0.09	0.91	-0.39	-0.19	0.00	-0.19	0.08	0.95	-0.44	-0.21	0.04	-0.22	0.09	0.97
6770	STAR	-0.03	0.14	0.29	0.14	0.06	0.90	0.06	0.15	0.30	0.15	0.05	0.98	0.00	0.15	0.30	0.15	0.06	0.94
6804	STX1A	-0.38	-0.21	0.00	-0.21	0.07	0.98	-0.36	-0.22	-0.06	-0.21	0.06	0.99	-0.39	-0.24	-0.02	-0.24	0.06	0.99
6843	VAMP1	-0.10	0.20	0.49	0.21	0.09	0.94	0.03	0.22	0.41	0.21	0.08	0.96	0.01	0.23	0.44	0.24	0.08	0.98
6854	SYN2	-0.35	-0.16	0.00	-0.16	0.06	0.94	-0.30	-0.16	-0.05	-0.16	0.05	0.96	-0.32	-0.18	0.02	-0.18	0.06	0.98
6890	TAP1	0.00	0.18	0.34	0.18	0.06	0.96	0.06	0.19	0.32	0.19	0.05	0.99	0.01	0.22	0.36	0.21	0.06	0.99
6905	TBCE	-0.02	0.20	0.43	0.20	0.08	0.95	0.04	0.20	0.35	0.19	0.07	0.97	0.02	0.21	0.39	0.21	0.07	0.99
6920	TCEA3	-0.44	-0.18	0.07	-0.18	0.08	0.93	-0.36	-0.19	-0.04	-0.19	0.07	0.95	-0.42	-0.21	-0.02	-0.21	0.08	0.98
7007	TECTA	-0.02	0.12	0.24	0.12	0.04	0.90	0.00	0.13	0.21	0.12	0.04	0.95	0.03	0.14	0.25	0.14	0.04	0.96
7062	TCHH	-0.01	0.16	0.37	0.16	0.06	0.92	0.04	0.15	0.29	0.16	0.05	0.96	0.03	0.17	0.32	0.17	0.06	0.95
7068	THRB	-0.35	-0.17	-0.01	-0.18	0.06	0.97	-0.28	-0.19	-0.07	-0.19	0.05	1.00	-0.34	-0.20	-0.03	-0.20	0.06	0.99
7087	ICAM5	-0.33	-0.14	0.03	-0.15	0.06	0.93	-0.29	-0.14	0.02	-0.14	0.06	0.90	-0.30	-0.16	0.01	-0.16	0.05	0.98
7089	TLE2	-0.08	0.17	0.40	0.17	0.07	0.92	0.03	0.18	0.34	0.18	0.06	0.96	-0.02	0.19	0.40	0.19	0.07	0.98
7092	TLL1	-0.32	-0.17	-0.01	-0.17	0.06	0.96	-0.32	-0.17	0.03	-0.17	0.06	0.99	-0.33	-0.20	-0.04	-0.20	0.06	0.99
7103	TSPAN8	-0.04	0.15	0.27	0.15	0.05	0.96	0.04	0.16	0.26	0.16	0.04	0.99	0.02	0.17	0.28	0.17	0.05	0.99
7138	TNNT1	-0.40	-0.19	0.07	-0.19	0.07	0.95	-0.36	-0.20	0.00	-0.19	0.06	0.99	-0.38	-0.21	0.01	-0.21	0.07	0.98
7145	TNS1	-0.02	0.13	0.26	0.13	0.04	0.94	0.03	0.14	0.23	0.14	0.04	0.97	0.02	0.15	0.28	0.15	0.04	0.98
7222	TRPC3	-0.09	0.19	0.49	0.20	0.09	0.94	0.02	0.20	0.40	0.20	0.08	0.96	0.00	0.22	0.44	0.22	0.08	0.98
7260	EIPR1	-0.25	-0.13	-0.01	-0.13	0.04	0.91	-0.24	-0.13	-0.01	-0.13	0.05	0.91	-0.26	-0.15	0.01	-0.15	0.05	0.96
7301	TYRO3	-0.32	-0.16	0.02	-0.16	0.06	0.94	-0.29	-0.15	0.01	-0.16	0.05	0.95	-0.30	-0.17	-0.02	-0.17	0.05	0.97
7347	UCHL3	-0.40	-0.15	0.06	-0.15	0.07	0.91	-0.33	-0.16	-0.02	-0.16	0.07	0.92	-0.38	-0.18	0.03	-0.19	0.07	0.97
7378	UPP1	-0.09	0.19	0.49	0.19	0.08	0.93	0.02	0.19	0.37	0.19	0.07	0.95	-0.01	0.20	0.42	0.20	0.08	0.96
7402	UTRN	-0.08	0.14	0.34	0.15	0.07	0.90	0.00	0.16	0.26	0.15	0.06	0.92	-0.01	0.16	0.32	0.16	0.06	0.95
7409	VAV1	-0.38	-0.16	0.09	-0.16	0.07	0.91	-0.36	-0.18	-0.03	-0.18	0.07	0.96	-0.35	-0.19	0.03	-0.19	0.07	0.98
7480	WNT10B	-0.45	-0.18	0.08	-0.19	0.08	0.94	-0.36	-0.19	-0.04	-0.19	0.07	0.96	-0.44	-0.21	0.01	-0.21	0.08	0.98
7533	YWHAH	-0.32	-0.15	0.05	-0.15	0.06	0.93	-0.27	-0.15	-0.03	-0.15	0.05	0.93	-0.32	-0.16	-0.04	-0.17	0.06	0.97
7534	YWHAZ	-0.30	-0.16	-0.01	-0.16	0.05	0.95	-0.28	-0.16	-0.04	-0.16	0.05	0.98	-0.33	-0.18	0.00	-0.18	0.06	0.98
7726	TRIM26	0.00	0.14	0.30	0.14	0.05	0.92	0.05	0.15	0.25	0.15	0.05	0.95	0.02	0.16	0.31	0.16	0.05	0.97
7781	SLC30A3	-0.33	-0.19	0.01	-0.19	0.06	0.97	-0.31	-0.19	-0.05	-0.19	0.05	0.98	-0.34	-0.21	-0.03	-0.21	0.05	0.99
7791	ZYX	-0.06	0.17	0.41	0.17	0.08	0.91	0.02	0.17	0.33	0.18	0.07	0.94	0.00	0.19	0.38	0.19	0.07	0.97
7798	LUZP1	-0.02	0.15	0.34	0.15	0.06	0.91	0.04	0.15	0.31	0.15	0.05	0.94	0.03	0.16	0.31	0.16	0.06	0.95
8001	GLRA3	-0.42	-0.19	0.06	-0.19	0.08	0.96	-0.34	-0.19	-0.04	-0.19	0.07	0.97	-0.42	-0.21	-0.03	-0.21	0.08	0.98
8099	CDK2AP1	0.00	0.15	0.30	0.15	0.05	0.95	0.00	0.14	0.26	0.15	0.05	0.97	0.03	0.17	0.30	0.17	0.05	0.98
8174	MADCAM1	-0.02	0.17	0.37	0.17	0.07	0.93	0.01	0.18	0.34	0.18	0.07	0.93	0.01	0.20	0.38	0.20	0.07	0.98
8193	DPF1	-0.31	-0.14	0.06	-0.14	0.06	0.91	-0.25	-0.15	-0.01	-0.14	0.05	0.94	-0.29	-0.15	-0.02	-0.15	0.05	0.95
8209	C21orf33	0.03	0.15	0.28	0.15	0.05	0.95	0.06	0.16	0.25	0.16	0.05	0.98	0.02	0.17	0.30	0.17	0.05	0.98
8321	FZD1	-0.38	-0.16	0.05	-0.16	0.08	0.91	-0.33	-0.17	-0.01	-0.17	0.06	0.97	-0.36	-0.17	0.03	-0.18	0.07	0.95
8404	SPARCL1	0.00	0.15	0.28	0.15	0.05	0.93	0.02	0.16	0.28	0.16	0.05	0.97	0.04	0.17	0.29	0.17	0.05	0.98
8425	LTBP4	-0.33	-0.15	0.01	-0.15	0.06	0.92	-0.29	-0.15	-0.03	-0.15	0.05	0.96	-0.31	-0.17	0.01	-0.17	0.06	0.96
8437	RASAL1	-0.43	-0.17	0.10	-0.18	0.08	0.93	-0.35	-0.17	0.01	-0.18	0.07	0.94	-0.38	-0.20	0.00	-0.20	0.07	0.97
8445	DYRK2	-0.36	-0.15	0.08	-0.15	0.06	0.91	-0.27	-0.15	-0.02	-0.15	0.06	0.95	-0.34	-0.16	-0.02	-0.17	0.07	0.94
8470	SORBS2	-0.30	-0.14	0.02	-0.15	0.06	0.91	-0.28	-0.14	0.00	-0.14	0.06	0.90	-0.32	-0.16	-0.01	-0.16	0.06	0.97
8482	SEMA7A	-0.08	0.20	0.48	0.20	0.09	0.94	0.03	0.21	0.40	0.20	0.08	0.96	0.01	0.22	0.45	0.23	0.09	0.98
8507	ENC1	-0.28	-0.14	0.04	-0.14	0.06	0.90	-0.24	-0.13	0.07	-0.13	0.05	0.92	-0.27	-0.16	0.00	-0.15	0.05	0.95
8604	SLC25A12	-0.07	0.17	0.38	0.17	0.07	0.91	0.03	0.18	0.33	0.18	0.06	0.94	0.00	0.18	0.35	0.18	0.07	0.97
8611	PLPP1	0.02	0.16	0.30	0.16	0.05	0.98	0.08	0.17	0.27	0.17	0.04	1.00	0.00	0.18	0.30	0.18	0.05	1.00
8704	B4GALT2	-0.41	-0.19	0.07	-0.19	0.08	0.96	-0.34	-0.20	-0.05	-0.20	0.07	0.98	-0.42	-0.21	-0.02	-0.22	0.08	0.98
8715	NOL4	-0.40	-0.20	0.05	-0.20	0.07	0.96	-0.37	-0.20	-0.06	-0.20	0.07	0.99	-0.40	-0.22	-0.06	-0.22	0.07	0.99

8717	TRADD	0.01	0.16	0.28	0.15	0.05	0.96	0.07	0.16	0.23	0.16	0.04	1.00	0.04	0.16	0.27	0.16	0.05	0.98
8792	TNFRSF11A	-0.39	-0.19	0.07	-0.19	0.07	0.95	-0.35	-0.20	0.01	-0.19	0.06	0.98	-0.40	-0.23	-0.05	-0.23	0.07	0.99
8805	TRIM24	-0.31	-0.13	0.03	-0.13	0.05	0.91	-0.28	-0.15	-0.03	-0.15	0.05	0.95	-0.26	-0.15	-0.03	-0.16	0.05	0.98
8853	ASAP2	-0.03	0.13	0.28	0.13	0.05	0.92	0.05	0.15	0.27	0.14	0.04	0.96	0.02	0.14	0.25	0.14	0.05	0.95
8871	SYNJ2	-0.02	0.13	0.24	0.13	0.04	0.93	0.02	0.15	0.24	0.15	0.04	0.98	0.04	0.15	0.25	0.15	0.04	0.97
8884	SLC5A6	-0.03	0.18	0.39	0.18	0.07	0.95	0.06	0.19	0.34	0.20	0.06	0.97	0.00	0.21	0.38	0.21	0.07	0.99
8938	BAIAP3	-0.55	-0.24	0.08	-0.24	0.10	0.97	-0.45	-0.24	-0.07	-0.25	0.09	0.98	-0.54	-0.26	-0.04	-0.27	0.09	0.99
9020	MAP3K14	-0.01	0.16	0.31	0.15	0.06	0.93	0.05	0.17	0.28	0.17	0.05	0.98	-0.01	0.18	0.33	0.18	0.05	0.98
9033	PKD2L1	-0.39	-0.19	0.01	-0.19	0.07	0.97	-0.32	-0.21	-0.06	-0.20	0.06	0.99	-0.39	-0.21	0.01	-0.21	0.07	0.98
9120	SLC16A6	-0.03	0.18	0.43	0.18	0.08	0.93	0.04	0.19	0.37	0.19	0.07	0.96	0.03	0.21	0.40	0.21	0.07	0.98
9127	P2RX6	-0.01	0.20	0.44	0.21	0.08	0.95	0.00	0.22	0.40	0.22	0.07	0.99	0.02	0.24	0.43	0.25	0.07	0.99
9131	AIFM1	0.01	0.13	0.27	0.13	0.04	0.94	0.02	0.12	0.26	0.13	0.04	0.94	0.02	0.14	0.25	0.14	0.04	0.95
9168	TMSB10	-0.43	-0.18	0.07	-0.18	0.08	0.94	-0.37	-0.19	0.02	-0.19	0.07	0.97	-0.39	-0.21	0.01	-0.22	0.07	0.99
9196	KCNAB3	-0.08	0.21	0.47	0.20	0.09	0.94	0.03	0.20	0.40	0.21	0.07	0.96	0.01	0.22	0.43	0.23	0.08	0.98
9253	NUMBL	-0.29	-0.15	-0.03	-0.15	0.05	0.95	-0.24	-0.16	-0.07	-0.17	0.04	1.00	-0.29	-0.17	-0.01	-0.17	0.05	0.98
9254	CACNA2D2	-0.02	0.16	0.34	0.16	0.06	0.93	0.03	0.17	0.30	0.16	0.06	0.93	0.04	0.18	0.33	0.18	0.06	0.98
9256	TSPOAP1	-0.34	-0.18	-0.02	-0.18	0.06	0.96	-0.31	-0.19	-0.08	-0.19	0.05	1.00	-0.37	-0.21	-0.04	-0.21	0.06	0.99
9267	CYTH1	0.00	0.14	0.29	0.14	0.05	0.92	0.03	0.15	0.26	0.15	0.04	0.98	-0.05	0.17	0.27	0.16	0.05	0.98
9312	KCNB2	-0.35	-0.17	0.00	-0.17	0.06	0.96	-0.31	-0.19	-0.06	-0.19	0.06	1.00	-0.37	-0.19	-0.05	-0.19	0.06	0.99
9315	NREP	-0.03	0.16	0.35	0.16	0.07	0.91	0.06	0.18	0.31	0.18	0.06	0.95	0.02	0.18	0.33	0.18	0.06	0.98
9362	CPNE6	-0.48	-0.20	0.04	-0.21	0.09	0.94	-0.40	-0.20	-0.03	-0.21	0.08	0.98	-0.43	-0.23	-0.01	-0.23	0.08	0.99
9379	NRXN2	-0.24	-0.13	-0.01	-0.13	0.04	0.93	-0.26	-0.15	-0.05	-0.14	0.04	0.98	-0.29	-0.15	-0.03	-0.15	0.04	0.98
9382	COG1	-0.32	-0.14	0.03	-0.14	0.06	0.92	-0.27	-0.15	-0.01	-0.15	0.05	0.93	-0.30	-0.16	0.02	-0.16	0.05	0.97
9399	STOML1	-0.31	-0.15	0.05	-0.15	0.06	0.94	-0.28	-0.15	-0.01	-0.15	0.05	0.95	-0.29	-0.17	0.00	-0.17	0.05	0.98
9452	ITM2A	-0.08	0.17	0.40	0.17	0.08	0.92	0.05	0.19	0.35	0.19	0.07	0.96	-0.01	0.20	0.42	0.21	0.07	0.97
9454	HOMER3	-0.04	0.17	0.33	0.17	0.06	0.96	0.03	0.17	0.29	0.17	0.05	0.97	0.03	0.20	0.34	0.20	0.05	0.99
9473	THEMIS2	0.03	0.17	0.30	0.17	0.05	0.98	0.07	0.18	0.27	0.17	0.04	1.00	0.03	0.19	0.33	0.19	0.05	1.00
9495	AKAP5	-0.29	-0.14	-0.01	-0.15	0.05	0.95	-0.26	-0.16	-0.04	-0.16	0.04	0.98	-0.28	-0.17	0.02	-0.17	0.04	0.98
9508	ADAMTS3	-0.43	-0.16	0.14	-0.16	0.08	0.90	-0.29	-0.16	-0.01	-0.16	0.07	0.91	-0.45	-0.17	0.03	-0.18	0.08	0.93
9537	TP53I11	-0.32	-0.15	0.02	-0.15	0.06	0.91	-0.30	-0.16	-0.03	-0.16	0.06	0.96	-0.32	-0.17	-0.03	-0.18	0.06	0.97
9570	GOSR2	-0.28	-0.13	0.03	-0.13	0.05	0.91	-0.27	-0.15	-0.05	-0.15	0.05	0.94	-0.29	-0.16	-0.02	-0.16	0.05	0.98
9592	IER2	-0.08	0.15	0.35	0.16	0.07	0.92	0.02	0.17	0.33	0.17	0.06	0.94	-0.09	0.18	0.35	0.18	0.07	0.96
9607	CARTPT	-0.44	-0.21	0.05	-0.21	0.09	0.94	-0.39	-0.24	-0.02	-0.24	0.08	0.96	-0.45	-0.25	-0.04	-0.24	0.08	0.98
9609	RAB36	-0.42	-0.18	0.06	-0.18	0.08	0.93	-0.33	-0.19	-0.06	-0.19	0.07	0.96	-0.39	-0.20	-0.01	-0.21	0.07	0.98
9630	GNA14	-0.01	0.14	0.31	0.14	0.05	0.93	0.08	0.14	0.26	0.15	0.04	1.00	0.04	0.16	0.29	0.16	0.05	0.96
9636	ISG15	-0.34	-0.17	0.02	-0.17	0.07	0.94	-0.34	-0.19	-0.03	-0.19	0.05	0.98	-0.35	-0.21	-0.03	-0.21	0.06	0.99
9651	PLCH2	-0.28	-0.16	0.01	-0.15	0.05	0.94	-0.29	-0.15	-0.04	-0.15	0.05	0.97	-0.29	-0.16	0.02	-0.16	0.05	0.96
9653	HS2ST1	-0.23	-0.13	0.01	-0.13	0.04	0.94	-0.23	-0.14	-0.03	-0.14	0.04	0.95	-0.24	-0.16	-0.01	-0.15	0.04	0.99
9717	SEC14L5	0.01	0.15	0.30	0.15	0.05	0.93	0.06	0.16	0.28	0.16	0.05	0.97	-0.02	0.17	0.29	0.17	0.05	0.98
9731	CEP104	-0.02	0.13	0.25	0.13	0.04	0.93	0.00	0.13	0.23	0.13	0.04	0.95	-0.01	0.14	0.25	0.14	0.04	0.96
9750	RIPOR2	-0.49	-0.19	0.10	-0.19	0.09	0.93	-0.40	-0.20	-0.03	-0.20	0.08	0.97	-0.45	-0.21	0.01	-0.22	0.09	0.97
9770	RASSF2	0.00	0.12	0.20	0.12	0.04	0.92	0.03	0.13	0.21	0.13	0.04	0.97	0.02	0.14	0.23	0.13	0.04	0.97
9790	BMS1	-0.01	0.14	0.32	0.14	0.05	0.91	0.01	0.13	0.27	0.13	0.05	0.94	0.01	0.15	0.28	0.15	0.05	0.93
9823	ARMCX2	-0.32	-0.14	0.07	-0.14	0.06	0.91	-0.26	-0.14	-0.01	-0.14	0.05	0.93	-0.29	-0.16	0.03	-0.16	0.06	0.96
9854	C2CD2L	-0.28	-0.15	0.00	-0.15	0.05	0.96	-0.26	-0.15	-0.03	-0.15	0.04	0.98	-0.27	-0.17	-0.03	-0.17	0.04	0.98
9882	TBC1D4	-0.06	0.16	0.40	0.17	0.07	0.93	0.03	0.17	0.34	0.17	0.06	0.95	0.00	0.18	0.35	0.19	0.06	0.97
9903	KLHL21	-0.01	0.16	0.31	0.15	0.06	0.93	0.06	0.16	0.29	0.17	0.05	0.98	0.02	0.17	0.31	0.18	0.05	0.98
9909	DENND4B	-0.03	0.13	0.32	0.13	0.05	0.90	0.02	0.13	0.25	0.13	0.05	0.92	0.03	0.14	0.26	0.14	0.05	0.93
9911	TMCC2	0.01	0.18	0.38	0.19	0.06	0.96	0.06	0.19	0.32	0.19	0.06	0.99	0.04	0.21	0.35	0.21	0.06	0.99
10023	FRAT1	-0.08	0.16	0.45	0.16	0.08	0.90	-0.01	0.16	0.34	0.16	0.07	0.91	-0.01	0.17	0.38	0.18	0.08	0.97
10025	MED16	-0.30	-0.13	0.03	-0.14	0.05	0.91	-0.25	-0.13	-0.01	-0.13	0.05	0.90	-0.27	-0.14	0.00	-0.14	0.05	0.96
10026	PIGK	-0.01	0.14	0.28	0.14	0.05	0.92	0.03	0.14	0.23	0.14	0.04	0.96	0.01	0.14	0.26	0.14	0.05	0.94
10039	PARP3	-0.02	0.15	0.36	0.15	0.06	0.92	0.02	0.15	0.27	0.15	0.05	0.94	0.02	0.16	0.30	0.16	0.05	0.97
10040	TOM1L1	-0.38	-0.16	0.08	-0.16	0.07	0.93	-0.31	-0.16	-0.02	-0.16	0.06	0.97	-0.37	-0.18	0.01	-0.18	0.07	0.96
10083	USH1C	0.00	0.15	0.28	0.15	0.05	0.96	0.07	0.16	0.26	0.16	0.04	1.00	0.04	0.17	0.27	0.17	0.05	0.99
10154	PLXNC1	-0.37	-0.18	-0.01	-0.19	0.07	0.96	-0.34	-0.21	-0.08	-0.20	0.06	1.00	-0.36	-0.21	-0.02	-0.21	0.07	0.99
10160	FARP1	-0.44	-0.20	0.02	-0.20	0.08	0.94	-0.40	-0.21	-0.03	-0.21	0.07	0.99	-0.42	-0.22	-0.03	-0.23	0.08	0.99
10171	RCL1	-0.03	0.15	0.31	0.15	0.06	0.95	0.05	0.15	0.27	0.15	0.05	0.95	-0.03	0.16	0.30	0.17	0.06	0.98
10231	RCAN2	0.01	0.19	0.41	0.20	0.07	0.96	0.08	0.22	0.38	0.21	0.06	1.00	0.04	0.22	0.38	0.22	0.07	0.99
10268	RAMP3	0.03	0.18	0.33	0.18	0.06	0.98	0.07	0.19	0.33	0.19	0.05	0.99	0.06	0.21	0.35	0.21	0.05	0.99
10318	TNIP1	-0.05	0.18	0.40	0.18	0.07	0.94	0.02	0.17	0.34	0.18	0.07	0.95	0.03	0.20	0.39	0.20	0.07	0.98
10332	CLEC4M	-0.02	0.14	0.29	0.15	0.05	0.94	0.03	0.15	0.25	0.15	0.05	0.96	0.04	0.16	0.29	0.16	0.05	0.98
10368	CACNG3	-0.35	-0.16	0.08	-0.15	0.07	0.91	-0.30	-0.16	-0.01	-0.16	0.06	0.93	-0.34	-0.18	0.01	-0.18	0.06	0.97
10384	BTN3A3	-0.02	0.15	0.27	0.15	0.05	0.96	0.06	0.16	0.26	0.16	0.04	0.98	0.04	0.18	0.28	0.18	0.05	0.99
10395	DLC1	-0.01	0.16	0.30	0.16	0.05	0.96	0.07	0.17	0.28	0.17	0.05	0.99	0.04	0.18	0.31	0.18	0.05	0.98
10402	ST3GAL6	-0.12	0.17	0.46	0.18	0.09	0.91	0.02	0.17	0.37	0.18	0.08	0.91	-0.03	0.19	0.45	0.19	0.08	0.96
10425	ARIH2	-0.05	0.16	0.33	0.16	0.07	0.93	0.04	0.17	0.30	0.17	0.05	0.96	-0.03	0.18	0.33	0.18	0.06	0.97
10451	VAV3	-0.08	0.21	0.51	0.22	0.09	0.96	0.07	0.22	0.40	0.22	0.08	1.00	0.04	0.23	0.51	0.23	0.09	0.98
10478	SLC25A17	-0.03	0.14	0.33	0.15	0.06	0.91	0.01	0.14	0.27	0.14	0.05	0.91	-0.01	0.15	0.32	0.15	0.06	0.94
10505	SEMA4F	-0.38	-0.18	0.04	-0.18	0.07	0.94	-0.31	-0.18	-0.06	-0.19	0.06	0.97	-0.37	-0.20	-0.03	-0.20	0.07	0.98
10560	SLC19A2	-0.04	0.15	0.36	0.15	0.07	0.90	-0.03	0.16	0.29	0.16	0.06	0.93	-0.02	0.17	0.33	0.17	0.06	0.96
10613	ERLIN1	-0.30	-0.15	0.02	-0.15	0.06	0.93	-0.27	-0.15	-0.03	-0.15	0.05	0.98	-0.27	-0.16	0.03	-0.16	0.05	0.96
10636	RGS14	-0.31	-0.14	0.05	-0.14	0.06	0.91	-0.29	-0.15	0.01	-0.15	0.05	0.95	-0.32	-0.17	0.06	-0.17	0.05	0.97
10669	CGREF1	-0.38	-0.18	0.01	-0.18	0.06	0.97	-0.34	-0.19	-0.02	-0.19	0.06	0.99	-0.35	-0.22	0.00	-0.21	0.06	0.99
10673	TNFSF13B	-0.28	-0.14	0.00	-0.14	0.05	0.94	-0.23	-0.15	-0.03	-0.15	0.05	0.96	-0.27	-0.15	-0.01	-0.15	0.05	0.97
10683	DLL3	-0.35	-0.16	-0.01	-0.17	0.06	0.94	-0.31	-0.18	-0.02	-0.18	0.06	0.98	-0.33	-0.21	-0.01	-0.20	0.06	0.99
10776	ARPP19	-0.38	-0.16	0.09	-0.17	0.06	0.94	-0.28	-0.15	-0.03	-0.16	0.06	0.93	-0.32	-0.18	-0.04	-0.18	0.06	0.98
10783	NEK6	-0.26	-0.14	-0.02	-0.14	0.04	0.96	-0.23	-0.15	-0.06	-0.15	0.04	0.99	-0.26	-0.15	-0.02	-0.15	0.04	0.98
10797	MTHFD2	0.00	0.13	0.26	0.14	0.05	0.93	0.02	0.13	0.24	0.13	0.04	0.93	0.03	0.14	0.25	0.14	0.04	0.95
10815	CPLX1	-0.08	0.19	0.50	0.19	0.09	0.93	0.02	0.19	0.39	0.19	0.08	0.94	-0.01	0.21	0.41	0.21	0.08	0.97
10857	PGRMC1	-0.33	-0.13	0.02	-0.13	0.06	0.90	-0.25	-0.14	0.00	-0.13	0.05	0.91	-0.28	-0.14	0.01	-0.15	0.05	0.94
10867	TSPAN9	-0.02	0.15	0.35	0.16	0.06	0.92	0.02	0.16	0.30	0.16	0.06	0.94	0.02	0.17	0.36	0.18	0.06	0.98
10891	PPARGC1A	-0.01	0.20	0.44	0.20	0.08	0.94	0.06	0.22	0.41	0.21	0.07	0.98	0.04	0.23	0.39	0.23	0.07	0.99
10988	METAP2	-0.03	0.14	0.32	0.14	0.06	0.91	0.02	0.15	0.27	0.15	0.05	0.95	-0.02	0.15	0.31	0.16	0.06	0.96
11069	RAPGEF4	-0.40	-0.19	0.01	-0.19	0.07	0.96	-0.35	-0.20	-0.07	-0.20	0.06	0.99	-0.37	-0.22	-0.01	-0.22	0.06	0.99
11118	BTN3A2	0.00	0.12	0.23	0.12	0.04	0.90	0.00	0.12	0.20	0.12	0.04	0.92	0.01	0.13	0.23	0.13	0.04	0.95
11138	TBC1D8	-0.02	0.17	0.35	0.18	0.07	0.95	0.04	0.18	0.30	0.18	0.05	0.98	0.03	0.19	0.34	0.19	0.06	0.98
11151	CORO1A	-0.35	-0.16	0.04	-0.16	0.06	0.94	-0.30	-0.16	0.01	-0.16	0.06	0.96	-0.32	-0.18	0.00	-0.19	0.05	0.99
11164	NUDT5	0.01	0.18	0.35	0.18	0.06	0.96	0.07	0.18	0.29	0.18	0.05	0.99	0.04	0.20	0.34	0.20	0.06	0.99
11167	FSTL1	-0.10	0.17	0.45	0.17	0.08	0.93	0.01	0.17	0.34	0.18	0.07	0.95	-0.01	0.20	0.40	0.20	0.07	0.97
11211	FZD10	0.00	0.12	0.23	0.12	0.04	0.93	0.03	0.12	0.21	0.12	0.03	0.96	-0.01	0.13	0.22	0.13	0.03	0.97
11228	RASSF8	-0.03	0.15	0.32	0.15	0.06	0.92	0.00	0.15	0.26	0.15	0.05	0.93	0.03	0.17	0.29	0.17	0.05	0.98
11259	FILIP1L	-0.34	-0.17	0.02	-0.16	0.06	0.96	-0.28	-0.18	-0.03	-0.17	0.05	0.96	-0.31	-0.19	-0.01	-0.19	0.05	0.98
11279	KLF8	-0.34	-0.16	0.04	-0.16	0.06	0.94	-0.29	-0.16	-0.05	-0.17	0.05	0.97	-0.33	-0.17	-0.01	-0.18	0.06	0.98
11342	RNF13	-0.02	0.13	0.23	0.12	0.04	0.93	0.05	0.14	0.22	0.14	0.04	0.97	0.02	0.15	0.24	0.15	0.04	0.98
22801	ITGA11	-0.03	0.20	0.39	0.20	0.07	0.96	0.07	0.22	0.35	0.22	0.06	1.00	0.05	0.22	0.42	0.22	0.07	0.99
22824	HSPA4L	-0.05	0.16	0.36	0.16	0.07	0.91	0.01	0.15	0.29	0.15	0.06	0.92	0.00	0.17	0.32	0.17	0.06	0.96
22881	ANKRD6	-0.49	-0.21	0.07	-0.21	0.09	0.94	-0.42	-0.21	-0.01	-0.21	0.08	0.98	-0.46	-0.23	-0.02	-0.24	0.09	0.98
22987	SV2C	0.01	0.22	0.49	0.22	0.08	0.95	0.06	0.23	0.43	0.23	0.07	0.99	0.04	0.24	0.43	0.24	0.08	0.99
22996	TTC39A	0.01	0.19	0.36	0.19	0.07	0.96	0.08	0.18	0.32	0.19	0.05	1.00	0.07	0.20	0.35	0.20	0.06	1.00
23046	KIF21B	-0.34	-0.17	0.00	-0.17	0.06	0.96	-0.30	-0.18	-0.03	-0.18	0.05	0.98	-0.33	-0.18	-0.02	-0.19	0.06	0.98
23094	SIPA1L3	-0.01	0.13	0.27	0.13	0.05	0.93	0.02	0.14	0.24	0.14	0.04	0.96	0.02	0.15	0.26	0.15	0.04	0.98
23109	DDN	-0.33	-0.16	0.01	-0.17	0.06	0.96	-0.30	-0.17	-0.03	-0.17	0.05	0.97	-0.34	-0.18	-0.01	-0.19	0.06	0.98
23111	SPART	-0.02	0.15	0.35	0.15	0.06	0.93	0.03	0.15	0.30	0.15	0.05	0.94	-0.01	0.16	0.31	0.17	0.06	0.96
23180	RFTN1	-0.32	-0.15	-0.01	-0.15	0.06	0.92	-0.28	-0.17	-0.01	-0.16	0.05	0.96	-0.32	-0.18	0.00	-0.17	0.06	0.98
23199	GSE1	-0.01	0.15	0.31	0.15	0.06	0.94	0.03	0.15	0.29	0.16	0.05	0.97	0.03	0.16	0.30	0.16	0.05	0.96
23212	RRS1	0.00	0.14	0.29	0.14	0.05	0.92	0.02	0.14	0.24	0.14	0.05	0.93	0.03	0.15	0.26	0.15	0.05	0.96
23259	DDHD2	0.00	0.14	0.31	0.15	0.06	0.91	0.02	0.15	0.25	0.15	0.05	0.95	-0.01	0.15	0.29	0.16	0.06	0.95
23263	MCF2L	-0.05	0.15	0.36	0.15	0.07	0.91	0.01	0.16	0.31	0.16	0.06	0.94	-0.01	0.17	0.34	0.17	0.06	0.98
23274	CLEC16A	-0.06	0.17	0.42	0.18	0.07	0.93	0.01	0.18	0.35	0.18	0.07	0.95	0.01	0.20	0.38	0.20	0.07	0.99
23284	ADGRL3	-0.25	-0.13	-0.01	-0.13	0.04	0.92	-0.25	-0.13	-0.05	-0.13	0.04	0.92	-0.26	-0.13	0.00	-0.14	0.04	0.97
23325	WASHC4	-0.02	0.13	0.28	0.13	0.05	0.90	0.02	0.14	0.25	0.14	0.05	0.93	-0.02	0.16	0.28	0.16	0.05	0.97
23406	COTL1	-0.02	0.16	0.29	0.16	0.05	0.95	0.08	0.16	0.29	0.16	0.05	1.00	0.03	0.17	0.29	0.17	0.05	0.98
23428	SLC7A8	0.01	0.14	0.29	0.15	0.05	0.93	0.03	0.15	0.25	0.15	0.05	0.93	0.04	0.16	0.28	0.16	0.05	0.97
23460	ABCA6	-0.07	0.16	0.38	0.16	0.07	0.92	0.03	0.16	0.31	0.16	0.06	0.93	-0.01	0.18	0.38	0.18	0.06	0.98
23467	NPTXR	-0.30	-0.15	0.00	-0.15	0.05	0.93	-0.28	-0.15	-0.01	-0.16	0.05	0.98	-0.30	-0.18	-0.03	-0.18	0.05	0.98
23479	ISCU	-0.01	0.13	0.25	0.13	0.05	0.90	0.03	0.13	0.24	0.14	0.04	0.97	0.00	0.13	0.25	0.13	0.05	0.94
23484	LEPROTL1	0.01	0.19	0.39	0.19	0.07	0.94	0.08	0.20	0.36	0.20	0.06	1.00	0.04	0.21	0.39	0.21	0.07	0.98
23504	RIMBP2	-0.33	-0.16	0.02	-0.16	0.06	0.94	-0.27	-0.16	-0.05	-0.16	0.05	0.98	-0.33	-0.18	-0.02	-0.18	0.06	0.98
23516	SLC39A14	-0.02	0.15	0.35	0.15	0.06	0.92	0.02	0.16	0.29	0.15	0.06	0.92	0.01	0.16	0.30	0.16	0.06	0.97
23642	SNHG1	-0.10	0.19	0.41	0.19	0.07	0.96	0.04	0.19	0.33	0.18	0.06	0.97	0.02	0.20	0.41	0.20	0.07	0.98
23780	APOL2	-0.04	0.17	0.41	0.17	0.08	0.92	0.03	0.19	0.34	0.18	0.07	0.95	0.01	0.20	0.37	0.20	0.07	0.98
24139	EML2	0.01	0.13	0.26	0.13	0.05	0.92	0.03	0.13	0.23	0.13	0.04	0.94	0.03	0.14	0.26	0.15	0.05	0.97
25759	SHC2	-0.32	-0.14	0.04	-0.15	0.06	0.92	-0.26	-0.14	-0.02	-0.14	0.05	0.91	-0.33	-0.15	0.02	-0.16	0.06	0.94
25769	SLC24A2	-0.03	0.18	0.41	0.18	0.08	0.93	0.04	0.19	0.35	0.19	0.07	0.95	-0.01	0.20	0.38	0.21	0.08	0.98
25818	KLK5	-0.40	-0.16	0.08	-0.16	0.07	0.92	-0.31	-0.16	-0.03	-0.16	0.06	0.94	-0.36	-0.18	-0.01	-0.18	0.06	0.97
25841	ABTB2	0.00	0.21	0.42	0.21	0.07	0.98	0.10	0.23	0.37	0.23	0.06	1.00	0.05	0.25	0.44	0.25	0.07	0.99
25854	FAM149A	-0.39	-0.17	0.03	-0.18	0.07	0.94	-0.33	-0.19	-0.06	-0.19	0.06	0.98	-0.37	-0.20	0.01	-0.21	0.07	0.99
25871	NEPRO	-0.33	-0.15	0.05	-0.15	0.06	0.93	-0.28	-0.15	-0.01	-0.15	0.06	0.94	-0.28	-0.17	-0.01	-0.17	0.05	0.97
25900	IFFO1	-0.05	0.19	0.44	0.20	0.08	0.94	0.04	0.20	0.36	0.20	0.07	0.96	0.02	0.21	0.41	0.22	0.08	0.98
25907	TMEM158	-0.42	-0.18	0.08	-0.18	0.08	0.92	-0.38	-0.19	-0.01	-0.19	0.07	0.97	-0.43	-0.21	0.01	-0.22	0.08	0.98
25924	MYRIP	-0.34	-0.16	0.01	-0.16	0.06	0.95	-0.32	-0.18	-0.06	-0.17	0.06	0.97	-0.35	-0.20	-0.02	-0.20	0.06	1.00
25946	ZNF385A	-0.03	0.17	0.40	0.18	0.07	0.95	0.04	0.18	0.34	0.18	0.06	0.97	0.04	0.20	0.37	0.21	0.06	0.99
25953	PNKD	-0.01	0.18	0.38	0.18	0.07	0.94	0.04	0.18	0.34	0.18	0.06	0.96	0.03	0.19	0.35	0.19	0.07	0.98
25989	ULK3	-0.04	0.20	0.41	0.20	0.07	0.96	0.07	0.22	0.37	0.22	0.06	0.98	0.02	0.23	0.41	0.23	0.07	0.99
26010	SPATS2L	-0.23	-0.13	0.00	-0.13	0.04	0.94	-0.20	-0.13	-0.04	-0.13	0.04	0.95	-0.22	-0.13	-0.03	-0.13	0.04	0.97
26031	OSBPL3	0.01	0.17	0.34	0.17	0.06	0.95	0.01	0.17	0.33	0.18	0.06	0.99	0.05	0.19	0.33	0.19	0.05	0.99
26059	ERC2	-0.36	-0.16	0.03	-0.17	0.06	0.95	-0.33	-0.18	-0.02	-0.18	0.06	0.95	-0.35	-0.20	0.02	-0.20	0.06	0.98
26232	FBXO2	-0.35	-0.16	0.05	-0.16	0.07	0.92	-0.30	-0.17	-0.04	-0.17	0.06	0.94	-0.33	-0.18	0.03	-0.18	0.07	0.95
26996	GPR160	-0.48	-0.18	0.06	-0.18	0.08	0.92	-0.38	-0.19	-0.04	-0.19	0.08	0.96	-0.40	-0.20	0.00	-0.20	0.08	0.97
27077	B9D1	-0.47	-0.19	0.06	-0.19	0.08	0.95	-0.37	-0.20	-0.02	-0.20	0.07	0.99	-0.39	-0.21	0.00	-0.22	0.07	0.98
27132	CPNE7	-0.45	-0.20	0.03	-0.20	0.08	0.95	-0.37	-0.20	-0.06	-0.20	0.07	0.98	-0.43	-0.22	-0.01	-0.23	0.08	0.98
27163	NAAA	-0.06	0.18	0.38	0.18	0.07	0.95	0.02	0.19	0.33	0.19	0.06	0.98	0.03	0.20	0.37	0.20	0.06	0.98
27252	KLHL20	-0.02	0.15	0.33	0.15	0.06	0.94	0.02	0.16	0.29	0.16	0.05	0.94	0.02	0.17	0.32	0.17	0.06	0.98
27254	CSDC2	-0.06	0.17	0.37	0.17	0.07	0.94	0.02	0.17	0.33	0.17	0.06	0.95	0.04	0.19	0.37	0.19	0.06	0.98

27294	DHDH	-0.35	-0.17	-0.02	-0.17	0.06	0.95	-0.35	-0.19	-0.07	-0.18	0.06	0.99	-0.36	-0.20	-0.03	-0.21	0.06	0.99
27341	RRP7A	-0.45	-0.16	0.06	-0.16	0.08	0.91	-0.33	-0.16	-0.02	-0.17	0.07	0.93	-0.36	-0.17	0.00	-0.18	0.08	0.95
27345	KCNMB4	-0.44	-0.16	0.12	-0.17	0.08	0.91	-0.36	-0.17	-0.02	-0.17	0.07	0.94	-0.40	-0.19	0.01	-0.19	0.08	0.96
28231	SLCO4A1	-0.03	0.16	0.39	0.16	0.07	0.92	-0.01	0.16	0.33	0.17	0.07	0.91	-0.04	0.19	0.35	0.19	0.07	0.96
28232	SLCO3A1	0.00	0.12	0.23	0.12	0.04	0.91	0.02	0.13	0.22	0.13	0.04	0.96	0.02	0.14	0.23	0.14	0.04	0.95
28955	DEXI	0.01	0.19	0.40	0.19	0.07	0.95	0.05	0.20	0.37	0.20	0.06	0.98	0.04	0.21	0.36	0.22	0.06	0.99
28966	SNX24	-0.06	0.13	0.25	0.13	0.04	0.92	0.04	0.12	0.22	0.13	0.04	0.98	0.02	0.13	0.23	0.13	0.04	0.94
29115	SAP30BP	-0.24	-0.13	0.00	-0.13	0.05	0.90	-0.24	-0.14	-0.02	-0.14	0.04	0.93	-0.24	-0.14	-0.03	-0.14	0.04	0.97
29126	CD274	0.01	0.15	0.31	0.15	0.06	0.93	0.05	0.15	0.28	0.15	0.05	0.95	0.02	0.16	0.31	0.16	0.06	0.96
29799	YPEL1	-0.39	-0.19	-0.02	-0.20	0.08	0.96	-0.37	-0.21	-0.05	-0.20	0.07	0.98	-0.39	-0.23	-0.04	-0.23	0.07	0.99
29803	REPIN1	0.00	0.18	0.37	0.18	0.06	0.95	0.06	0.19	0.34	0.19	0.06	0.99	0.00	0.21	0.36	0.21	0.06	0.99
29844	TFPT	-0.40	-0.17	0.09	-0.17	0.08	0.92	-0.32	-0.19	-0.03	-0.19	0.06	0.96	-0.38	-0.20	0.01	-0.20	0.07	0.97
29902	FAM216A	-0.04	0.17	0.39	0.17	0.07	0.91	0.03	0.16	0.33	0.16	0.06	0.94	-0.01	0.17	0.35	0.18	0.07	0.97
29904	EEF2K	-0.03	0.14	0.34	0.14	0.06	0.92	0.05	0.15	0.26	0.15	0.05	0.95	0.01	0.16	0.30	0.16	0.05	0.98
30850	CDR2L	-0.02	0.20	0.43	0.21	0.07	0.96	0.07	0.22	0.38	0.21	0.06	0.99	0.00	0.23	0.43	0.24	0.07	0.99
50486	G0S2	0.03	0.19	0.32	0.18	0.06	0.98	0.07	0.20	0.34	0.20	0.05	1.00	0.08	0.22	0.34	0.22	0.05	1.00
50853	VILL	-0.01	0.17	0.33	0.17	0.06	0.94	0.06	0.18	0.33	0.19	0.05	0.99	0.01	0.20	0.35	0.19	0.06	0.98
51022	GLRX2	0.00	0.14	0.27	0.14	0.06	0.90	0.03	0.13	0.27	0.14	0.05	0.93	-0.01	0.14	0.27	0.14	0.05	0.94
51043	ZBTB7B	-0.01	0.12	0.24	0.12	0.04	0.90	0.02	0.12	0.19	0.12	0.04	0.92	0.01	0.13	0.25	0.13	0.04	0.94
51059	FAM135B	0.00	0.16	0.29	0.16	0.06	0.93	0.07	0.17	0.31	0.17	0.05	1.00	0.03	0.17	0.31	0.17	0.05	0.97
51074	APIP	-0.01	0.16	0.30	0.16	0.05	0.95	0.04	0.16	0.27	0.16	0.05	0.95	0.03	0.17	0.31	0.17	0.05	0.98
51105	PHF20L1	-0.26	-0.13	0.03	-0.13	0.05	0.90	-0.23	-0.13	-0.01	-0.13	0.05	0.91	-0.26	-0.15	-0.02	-0.15	0.05	0.95
51123	ZNF706	-0.36	-0.16	0.04	-0.16	0.07	0.92	-0.31	-0.17	-0.02	-0.16	0.06	0.94	-0.37	-0.18	-0.01	-0.19	0.07	0.97
51134	CEP83	-0.30	-0.14	0.02	-0.14	0.06	0.90	-0.26	-0.18	-0.03	-0.17	0.05	0.98	-0.30	-0.17	0.00	-0.17	0.06	0.96
51155	JPT1	-0.37	-0.14	0.09	-0.15	0.07	0.90	-0.31	-0.16	0.00	-0.16	0.06	0.91	-0.34	-0.17	0.03	-0.18	0.06	0.98
51312	SLC25A37	-0.09	0.19	0.50	0.20	0.09	0.93	0.03	0.20	0.40	0.20	0.08	0.96	-0.01	0.22	0.43	0.22	0.08	0.98
51319	RSRC1	-0.04	0.16	0.38	0.16	0.07	0.92	0.04	0.16	0.31	0.16	0.06	0.92	0.02	0.17	0.35	0.17	0.07	0.95
51330	TNFRSF12A	-0.01	0.13	0.25	0.13	0.05	0.94	0.02	0.12	0.20	0.12	0.04	0.90	-0.02	0.14	0.25	0.14	0.04	0.94
51334	PRR16	-0.06	0.15	0.37	0.16	0.07	0.92	-0.01	0.15	0.29	0.16	0.06	0.96	0.01	0.17	0.37	0.17	0.06	0.97
51375	SNX7	-0.47	-0.21	0.07	-0.21	0.08	0.96	-0.41	-0.23	-0.06	-0.22	0.07	0.99	-0.45	-0.25	0.00	-0.25	0.08	0.99
51393	TRPV2	-0.31	-0.15	0.04	-0.15	0.06	0.92	-0.26	-0.15	0.01	-0.15	0.05	0.95	-0.30	-0.17	-0.01	-0.17	0.05	0.97
51440	HPCAL4	-0.43	-0.18	0.04	-0.19	0.07	0.95	-0.36	-0.20	-0.04	-0.19	0.06	0.98	-0.39	-0.21	-0.02	-0.22	0.07	0.98
51454	GULP1	-0.44	-0.17	0.07	-0.18	0.08	0.94	-0.34	-0.19	0.01	-0.18	0.07	0.97	-0.41	-0.19	0.00	-0.20	0.08	0.95
51522	TMEM14C	-0.02	0.17	0.40	0.17	0.07	0.94	0.04	0.17	0.34	0.17	0.06	0.97	0.04	0.19	0.36	0.19	0.07	0.96
51538	ZCCHC17	-0.42	-0.17	0.09	-0.17	0.08	0.92	-0.35	-0.18	-0.01	-0.18	0.07	0.93	-0.42	-0.19	0.00	-0.20	0.07	0.97
51635	DHRS7	-0.03	0.14	0.25	0.14	0.05	0.93	0.05	0.13	0.25	0.13	0.04	0.99	-0.02	0.14	0.22	0.13	0.04	0.95
51642	MRPL48	-0.03	0.14	0.31	0.14	0.06	0.92	0.00	0.15	0.25	0.15	0.05	0.95	0.03	0.16	0.31	0.16	0.06	0.97
51660	MPC1	-0.03	0.19	0.38	0.19	0.07	0.97	0.06	0.20	0.35	0.20	0.06	0.98	0.04	0.21	0.37	0.21	0.07	0.99
51667	NUB1	-0.07	0.16	0.37	0.16	0.07	0.94	0.03	0.17	0.30	0.16	0.06	0.96	0.01	0.18	0.34	0.18	0.06	0.98
51705	EMCN	0.00	0.15	0.30	0.15	0.05	0.94	0.07	0.18	0.28	0.17	0.05	1.00	-0.02	0.18	0.31	0.18	0.05	0.99
53826	FXYD6	-0.44	-0.16	0.11	-0.16	0.08	0.91	-0.35	-0.16	0.01	-0.16	0.07	0.90	-0.39	-0.18	0.00	-0.19	0.07	0.96
54112	GPR88	-0.39	-0.19	0.03	-0.20	0.07	0.95	-0.36	-0.21	-0.07	-0.21	0.06	0.99	-0.37	-0.22	-0.01	-0.22	0.07	0.99
54206	ERRFI1	0.01	0.15	0.32	0.15	0.06	0.91	0.04	0.15	0.29	0.15	0.05	0.95	0.01	0.17	0.30	0.17	0.06	0.95
54331	GNG2	-0.38	-0.15	0.06	-0.15	0.07	0.92	-0.31	-0.16	0.01	-0.16	0.06	0.93	-0.35	-0.17	0.05	-0.18	0.06	0.97
54407	SLC38A2	-0.07	0.17	0.38	0.16	0.08	0.91	0.01	0.18	0.36	0.18	0.07	0.94	-0.02	0.20	0.37	0.20	0.07	0.97
54492	NEURL1B	-0.38	-0.18	0.02	-0.18	0.07	0.93	-0.33	-0.20	-0.06	-0.19	0.06	0.98	-0.37	-0.20	0.00	-0.20	0.07	0.96
54536	EXOC6	-0.41	-0.19	0.04	-0.19	0.07	0.97	-0.34	-0.19	-0.02	-0.20	0.06	0.99	-0.37	-0.22	-0.02	-0.22	0.06	0.99
54550	NECAB2	-0.46	-0.21	0.05	-0.21	0.09	0.95	-0.38	-0.22	-0.02	-0.21	0.07	0.98	-0.44	-0.24	-0.03	-0.24	0.08	0.99
54551	MAGEL2	0.00	0.18	0.39	0.18	0.07	0.94	0.04	0.18	0.32	0.18	0.06	0.97	0.03	0.19	0.34	0.19	0.06	0.98
54566	EPB41L4B	-0.31	-0.16	0.03	-0.16	0.06	0.94	-0.29	-0.16	0.01	-0.17	0.05	0.97	-0.33	-0.19	-0.01	-0.19	0.05	0.98
54793	KCTD9	0.02	0.20	0.42	0.20	0.08	0.96	0.06	0.20	0.39	0.21	0.07	0.97	0.05	0.22	0.39	0.23	0.07	0.99
54843	SYTL2	-0.33	-0.17	0.01	-0.17	0.06	0.95	-0.30	-0.18	-0.02	-0.18	0.05	0.98	-0.34	-0.20	-0.04	-0.20	0.06	0.99
54847	SIDT1	-0.35	-0.16	0.04	-0.16	0.06	0.94	-0.27	-0.16	-0.02	-0.16	0.05	0.96	-0.34	-0.19	-0.01	-0.19	0.06	0.98
54874	FNBP1L	-0.42	-0.18	0.06	-0.18	0.07	0.94	-0.35	-0.19	-0.03	-0.19	0.07	0.94	-0.42	-0.21	-0.01	-0.22	0.07	0.99
54972	TMEM132A	-0.29	-0.15	0.00	-0.15	0.05	0.95	-0.25	-0.15	-0.02	-0.15	0.05	0.95	-0.28	-0.16	-0.01	-0.16	0.05	0.96
55006	TRMT61B	-0.08	0.15	0.40	0.16	0.07	0.91	0.01	0.15	0.30	0.15	0.06	0.92	-0.03	0.16	0.34	0.17	0.07	0.95
55022	PID1	-0.44	-0.17	0.11	-0.17	0.08	0.90	-0.36	-0.18	0.02	-0.18	0.08	0.90	-0.42	-0.20	0.04	-0.21	0.08	0.97
55040	EPN3	0.01	0.21	0.44	0.21	0.08	0.94	0.08	0.22	0.41	0.21	0.07	1.00	0.05	0.23	0.41	0.23	0.08	0.99
55062	WIPI1	-0.02	0.13	0.29	0.13	0.05	0.90	0.04	0.13	0.24	0.13	0.04	0.91	0.00	0.15	0.26	0.15	0.04	0.96
55086	CXorf57	-0.46	-0.19	0.08	-0.19	0.09	0.93	-0.39	-0.20	-0.02	-0.20	0.08	0.95	-0.44	-0.21	-0.01	-0.22	0.08	0.97
55118	CRTAC1	0.00	0.16	0.34	0.16	0.06	0.93	0.05	0.18	0.31	0.17	0.05	0.98	0.04	0.18	0.31	0.18	0.05	0.99
55122	AKIRIN2	-0.37	-0.16	0.06	-0.16	0.07	0.93	-0.30	-0.17	0.00	-0.17	0.06	0.98	-0.35	-0.19	0.00	-0.19	0.06	0.98
55160	ARHGEF10L	-0.05	0.17	0.37	0.17	0.06	0.94	0.00	0.17	0.31	0.17	0.06	0.96	0.01	0.19	0.38	0.19	0.06	0.98
55170	PRMT6	-0.28	-0.14	0.04	-0.14	0.05	0.91	-0.27	-0.17	-0.04	-0.16	0.05	0.97	-0.28	-0.16	-0.01	-0.16	0.05	0.99
55203	LGI2	0.00	0.14	0.27	0.14	0.05	0.92	-0.02	0.14	0.26	0.14	0.05	0.91	0.03	0.15	0.26	0.15	0.04	0.96
55208	DCUN1D2	-0.05	0.17	0.39	0.17	0.07	0.93	0.03	0.17	0.33	0.17	0.06	0.95	0.00	0.18	0.36	0.19	0.07	0.97
55244	SLC47A1	0.03	0.25	0.46	0.25	0.08	0.99	0.12	0.27	0.44	0.27	0.07	1.00	0.09	0.28	0.45	0.28	0.07	1.00
55315	SLC29A3	-0.34	-0.19	-0.02	-0.19	0.06	0.98	-0.30	-0.20	-0.05	-0.20	0.05	0.99	-0.33	-0.21	-0.07	-0.21	0.05	1.00
55353	LAPTM4B	0.00	0.18	0.40	0.19	0.07	0.93	0.06	0.19	0.35	0.19	0.06	0.99	0.02	0.20	0.40	0.21	0.07	0.98
55359	STYK1	-0.31	-0.14	0.05	-0.14	0.06	0.90	-0.25	-0.14	0.05	-0.14	0.05	0.92	-0.29	-0.16	0.02	-0.16	0.05	0.96
55502	HES6	-0.04	0.17	0.41	0.16	0.07	0.93	0.05	0.17	0.33	0.17	0.06	0.96	0.01	0.18	0.34	0.19	0.07	0.96
55509	BATF3	-0.43	-0.20	0.07	-0.20	0.08	0.96	-0.38	-0.21	-0.02	-0.21	0.07	0.99	-0.42	-0.23	-0.03	-0.23	0.07	0.99
55591	VEZT	-0.34	-0.16	0.04	-0.17	0.06	0.94	-0.30	-0.17	-0.04	-0.17	0.05	0.96	-0.34	-0.18	-0.01	-0.18	0.06	0.98
55686	MREG	-0.01	0.18	0.36	0.18	0.06	0.97	0.08	0.19	0.33	0.18	0.05	1.00	0.04	0.19	0.32	0.19	0.06	0.98
55714	TENM3	-0.34	-0.15	0.00	-0.15	0.06	0.92	-0.31	-0.15	-0.05	-0.15	0.06	0.92	-0.35	-0.17	0.01	-0.17	0.06	0.96
55790	CSGALNACT1	-0.28	-0.14	-0.02	-0.14	0.05	0.92	-0.23	-0.13	0.00	-0.13	0.04	0.93	-0.28	-0.16	-0.01	-0.16	0.05	0.97
55800	SCN3B	-0.48	-0.22	0.03	-0.23	0.08	0.96	-0.41	-0.24	-0.07	-0.23	0.07	0.99	-0.45	-0.25	-0.02	-0.26	0.08	0.99
55853	IDI2-AS1	0.01	0.19	0.39	0.19	0.07	0.95	0.08	0.19	0.35	0.19	0.06	1.00	0.03	0.21	0.38	0.21	0.07	0.99
55884	WSB2	-0.32	-0.14	0.09	-0.14	0.06	0.92	-0.25	-0.14	-0.01	-0.14	0.05	0.94	-0.31	-0.17	-0.02	-0.16	0.06	0.96
55897	MESP1	-0.44	-0.20	0.04	-0.21	0.07	0.96	-0.36	-0.21	-0.03	-0.22	0.07	0.99	-0.40	-0.23	-0.02	-0.24	0.07	0.99
55902	ACSS2	-0.05	0.12	0.24	0.12	0.04	0.90	0.05	0.13	0.22	0.13	0.04	0.94	-0.03	0.14	0.24	0.14	0.04	0.97
56172	ANKH	-0.06	0.19	0.42	0.19	0.08	0.93	0.04	0.20	0.37	0.20	0.07	0.97	0.01	0.21	0.40	0.21	0.07	0.98
56477	CCL28	-0.25	-0.14	0.00	-0.14	0.05	0.94	-0.25	-0.15	-0.04	-0.15	0.04	0.96	-0.26	-0.16	-0.02	-0.16	0.05	0.98
56616	DIABLO	-0.03	0.13	0.24	0.13	0.05	0.93	0.06	0.13	0.23	0.13	0.04	0.96	0.01	0.15	0.24	0.15	0.04	0.97
56648	EIF5A2	-0.06	0.18	0.44	0.19	0.08	0.93	0.03	0.19	0.37	0.19	0.07	0.95	0.00	0.21	0.41	0.21	0.08	0.98
56666	PANX2	-0.09	0.17	0.44	0.17	0.08	0.91	0.00	0.17	0.34	0.17	0.07	0.93	0.00	0.19	0.39	0.19	0.08	0.96
56848	SPHK2	-0.02	0.15	0.35	0.16	0.07	0.93	0.01	0.16	0.32	0.16	0.06	0.92	0.01	0.18	0.33	0.18	0.06	0.96
56884	FSTL5	-0.41	-0.15	0.08	-0.15	0.07	0.90	-0.28	-0.15	-0.04	-0.16	0.06	0.94	-0.32	-0.15	0.01	-0.15	0.07	0.92
56888	KCMF1	-0.01	0.15	0.31	0.15	0.06	0.92	0.04	0.16	0.29	0.15	0.05	0.95	0.03	0.16	0.28	0.16	0.05	0.96
56906	THAP10	-0.07	0.18	0.43	0.18	0.08	0.93	0.02	0.18	0.36	0.18	0.07	0.94	0.00	0.19	0.39	0.20	0.08	0.97
56934	CA10	-0.43	-0.18	0.11	-0.18	0.08	0.95	-0.33	-0.18	-0.01	-0.18	0.07	0.95	-0.41	-0.20	0.02	-0.20	0.08	0.96
56937	PMEPA1	-0.03	0.20	0.38	0.20	0.07	0.97	0.05	0.22	0.36	0.22	0.06	0.99	0.03	0.24	0.37	0.23	0.06	0.99
56967	C14orf132	-0.31	-0.17	0.01	-0.17	0.06	0.97	-0.29	-0.17	-0.05	-0.18	0.04	0.99	-0.32	-0.19	-0.05	-0.19	0.05	0.99
57110	HRASLS	-0.35	-0.16	0.07	-0.16	0.06	0.91	-0.31	-0.18	-0.07	-0.18	0.06	1.00	-0.34	-0.20	-0.01	-0.19	0.06	0.98
57185	NIPAL3	-0.03	0.13	0.26	0.13	0.04	0.94	0.02	0.14	0.24	0.14	0.04	0.97	0.02	0.15	0.26	0.15	0.04	0.98
57194	ATP10A	-0.03	0.14	0.31	0.15	0.06	0.93	0.06	0.18	0.29	0.18	0.05	0.98	0.00	0.19	0.33	0.19	0.05	0.98
57406	ABHD6	0.00	0.16	0.31	0.16	0.06	0.95	0.06	0.17	0.28	0.16	0.05	0.98	0.03	0.18	0.33	0.18	0.05	0.98
57453	DSCAML1	-0.01	0.17	0.33	0.17	0.06	0.95	0.07	0.18	0.29	0.18	0.05	0.99	0.03	0.19	0.34	0.19	0.05	0.99
57465	TBC1D24	-0.36	-0.16	0.02	-0.17	0.06	0.95	-0.30	-0.16	-0.04	-0.17	0.06	0.97	-0.36	-0.18	-0.02	-0.18	0.06	0.99
57484	RNF150	-0.41	-0.17	0.09	-0.17	0.08	0.93	-0.33	-0.17	0.00	-0.17	0.07	0.93	-0.38	-0.19	0.02	-0.19	0.07	0.97
57495	NWD2	-0.37	-0.21	0.00	-0.21	0.06	0.98	-0.36	-0.21	-0.09	-0.22	0.06	1.00	-0.38	-0.24	-0.06	-0.23	0.06	0.99
57496	MKL2	-0.37	-0.20	0.02	-0.20	0.07	0.97	-0.34	-0.20	-0.05	-0.20	0.06	0.98	-0.38	-0.22	-0.04	-0.22	0.06	0.99
57519	STARD9	-0.03	0.17	0.37	0.17	0.06	0.93	0.06	0.18	0.33	0.18	0.06	0.99	-0.04	0.19	0.36	0.20	0.06	0.99
57526	PCDH19	-0.42	-0.22	0.02	-0.22	0.07	0.98	-0.37	-0.23	-0.05	-0.23	0.06	0.99	-0.42	-0.25	-0.05	-0.25	0.07	0.99
57554	LRRC7	-0.30	-0.16	-0.01	-0.15	0.05	0.95	-0.28	-0.16	-0.07	-0.16	0.05	1.00	-0.30	-0.17	-0.03	-0.17	0.05	0.98
57596	BEGAIN	-0.27	-0.13	0.01	-0.13	0.05	0.91	-0.24	-0.14	-0.04	-0.14	0.04	0.91	-0.28	-0.15	-0.02	-0.15	0.05	0.96
57644	MYH7B	-0.02	0.17	0.41	0.18	0.07	0.94	0.03	0.18	0.35	0.18	0.06	0.96	0.01	0.19	0.38	0.19	0.07	0.96
57699	CPNE5	-0.31	-0.15	0.04	-0.15	0.06	0.92	-0.29	-0.16	-0.01	-0.16	0.05	0.94	-0.31	-0.17	-0.01	-0.17	0.06	0.97
57718	PPP4R4	-0.46	-0.20	0.09	-0.20	0.09	0.94	-0.39	-0.20	-0.04	-0.20	0.07	0.98	-0.45	-0.22	0.01	-0.23	0.08	0.99
57761	TRIB3	-0.28	-0.14	-0.02	-0.15	0.05	0.95	-0.26	-0.15	-0.04	-0.15	0.05	0.96	-0.29	-0.17	-0.05	-0.17	0.05	0.98
60625	DHX35	-0.06	0.16	0.41	0.16	0.07	0.92	0.03	0.17	0.34	0.17	0.06	0.94	0.01	0.18	0.36	0.19	0.07	0.98
63974	NEUROD6	-0.39	-0.16	0.06	-0.16	0.07	0.93	-0.28	-0.17	-0.02	-0.17	0.06	0.97	-0.37	-0.18	-0.03	-0.19	0.07	0.98
63982	ANO3	-0.41	-0.17	0.13	-0.17	0.08	0.91	-0.35	-0.17	-0.02	-0.17	0.07	0.95	-0.42	-0.18	0.03	-0.19	0.08	0.94
64131	XYLT1	-0.44	-0.16	0.08	-0.17	0.08	0.91	-0.33	-0.18	-0.04	-0.18	0.07	0.96	-0.41	-0.19	0.00	-0.20	0.08	0.95
64132	XYLT2	-0.07	0.17	0.40	0.18	0.07	0.94	0.01	0.18	0.35	0.18	0.06	0.96	0.03	0.20	0.38	0.20	0.07	0.98
64135	IFIH1	0.02	0.16	0.29	0.16	0.05	0.97	0.07	0.17	0.27	0.17	0.05	1.00	0.04	0.18	0.31	0.19	0.05	0.99
64137	ABCG4	-0.02	0.16	0.37	0.16	0.07	0.92	0.03	0.16	0.31	0.17	0.06	0.95	0.03	0.18	0.33	0.18	0.06	0.97
64149	C17orf75	0.00	0.17	0.36	0.18	0.06	0.95	0.04	0.17	0.32	0.17	0.06	0.97	0.04	0.19	0.32	0.19	0.06	0.98
64150	DIO3OS	0.00	0.13	0.26	0.13	0.05	0.94	0.04	0.14	0.30	0.15	0.05	0.98	0.04	0.17	0.28	0.16	0.04	0.99
64332	NFKBIZ	-0.06	0.13	0.29	0.13	0.05	0.90	0.01	0.14	0.23	0.13	0.04	0.91	0.01	0.15	0.30	0.15	0.05	0.97
64333	ARHGAP9	-0.03	0.18	0.43	0.18	0.08	0.92	0.02	0.18	0.35	0.18	0.07	0.95	0.01	0.20	0.39	0.21	0.08	0.98
64420	SUSD1	-0.35	-0.16	0.08	-0.16	0.07	0.91	-0.31	-0.16	-0.01	-0.16	0.06	0.94	-0.34	-0.18	0.01	-0.18	0.06	0.98
64781	CERK	-0.01	0.14	0.29	0.14	0.06	0.90	0.03	0.13	0.29	0.13	0.05	0.91	-0.01	0.14	0.28	0.14	0.06	0.92
64792	IFT22	-0.42	-0.17	0.09	-0.17	0.08	0.92	-0.36	-0.18	-0.04	-0.18	0.07	0.97	-0.40	-0.20	0.02	-0.20	0.07	0.98
64881	PCDH20	-0.47	-0.17	0.09	-0.17	0.08	0.91	-0.34	-0.16	-0.02	-0.17	0.07	0.92	-0.41	-0.19	-0.01	-0.19	0.08	0.95
65078	RTN4R	-0.30	-0.16	-0.04	-0.16	0.04	0.99	-0.24	-0.15	-0.06	-0.15	0.04	0.98	-0.26	-0.17	-0.04	-0.17	0.04	0.99
65110	UPF3A	-0.04	0.15	0.32	0.15	0.06	0.92	0.04	0.16	0.31	0.16	0.05	0.97	-0.05	0.17	0.32	0.17	0.06	0.97
65263	PYCR3	-0.32	-0.15	0.06	-0.15	0.06	0.91	-0.28	-0.15	-0.02	-0.15	0.06	0.94	-0.31	-0.17	0.01	-0.17	0.06	0.97
65992	DDRGK1	-0.35	-0.15	0.07	-0.16	0.07	0.91	-0.29	-0.15	0.02	-0.15	0.06	0.91	-0.33	-0.17	0.00	-0.17	0.06	0.95
65997	RASL11B	-0.41	-0.20	0.04	-0.20	0.07	0.97	-0.34	-0.21	-0.03	-0.21	0.07	0.99	-0.39	-0.23	-0.07	-0.24	0.07	1.00
66000	TMEM108	-0.36	-0.17	0.02	-0.18	0.07	0.94	-0.30	-0.18	-0.04	-0.18	0.06	0.99	-0.38	-0.20	-0.03	-0.20	0.07	0.98
66008	TRAK2	0.00	0.17	0.34	0.18	0.06	0.96	0.04	0.18	0.29	0.18	0.05	0.98	0.04	0.19	0.34	0.19	0.06	0.99
78990	OTUB2	-0.27	-0.13	-0.01	-0.13	0.05	0.92	-0.24	-0.13	-0.02	-0.13	0.04	0.95	-0.26	-0.15	0.00	-0.14	0.05	0.95
79012	CAMKV	-0.37	-0.19	0.01	-0.19	0.07	0.96	-0.34	-0.19	-0.01	-0.19	0.06	0.99	-0.36	-0.21	-0.05	-0.22	0.06	0.99
79016	DDA1	-0.36	-0.14	0.04	-0.15	0.07	0.90	-0.30	-0.16	-0.04	-0.16	0.06	0.97	-0.34	-0.17	0.00	-0.17	0.06	0.96
79017	GGCT	-0.42	-0.17	0.09	-0.17	0.08	0.94	-0.35	-0.18	-0.02	-0.18	0.07	0.96	-0.40	-0.19	-0.01	-0.20	0.07	0.97
79075	DSCC1	-0.07	0.14	0.33	0.15	0.06	0.90	-0.04	0.14	0.29	0.14	0.06	0.93	-0.05	0.16	0.32	0.16	0.06	0.96
79085	SLC25A23	-0.32	-0.16	0.01	-0.16	0.06	0.93	-0.25	-0.15	-0.02	-0.15	0.05	0.95	-0.31	-0.16	-0.02	-0.17	0.06	0.97
79158	GNPTAB	-0.28	-0.13	0.02	-0.13	0.05	0.91	-0.27	-0.14	-0.03	-0.14	0.04	0.93	-0.28	-0.14	0.00	-0.14	0.05	0.96
79183	TTPAL	-0.31	-0.14	0.07	-0.14	0.06	0.92	-0.27	-0.15	-0.03	-0.16	0.05	0.94	-0.30	-0.17	0.00	-0.17	0.06	0.97
79442	LRRC2	-0.40	-0.17	0.08	-0.17	0.08	0.94	-0.31	-0.16	-0.01	-0.16	0.07	0.91	-0.41	-0.18	-0.02	-0.19	0.07	0.97
79585	CORO7	-0.03	0.16	0.36	0.16	0.06	0.94	0.04	0.17	0.31	0.16	0.06	0.96	0.01	0.17	0.36	0.18	0.07	0.97
79645	EFCAB1	-0.48	-0.20	0.08	-0.21	0.09	0.95	-0.40	-0.20	-0.03	-0.21	0.08	0.98	-0.44	-0.22	-0.02	-0.23	0.08	0.98
79660	PPP1R3B	0.04	0.17	0.33	0.18	0.05	0.99	0.07	0.18	0.28	0.18	0.04	1.00	0.01	0.19	0.29	0.19	0.05	1.00
79720	VPS37B	-0.27	-0.16	-0.02	-0.15	0.05	0.95	-0.30	-0.15	-0.04	-0.16	0.05	0.98	-0.28	-0.16	-0.02	-0.16	0.05	0.98

79745	CLIP4	0.00	0.16	0.30	0.16	0.06	0.94	0.00	0.17	0.27	0.16	0.05	0.98	0.01	0.18	0.30	0.18	0.05	0.98
79750	ZNF385D	-0.01	0.16	0.34	0.16	0.06	0.92	0.02	0.16	0.32	0.17	0.05	0.96	0.03	0.17	0.31	0.17	0.05	0.97
79754	ASB13	-0.06	0.19	0.44	0.19	0.09	0.92	0.02	0.20	0.38	0.19	0.07	0.97	0.02	0.20	0.42	0.21	0.08	0.98
79762	C1orf115	-0.24	-0.13	0.00	-0.13	0.04	0.94	-0.22	-0.13	-0.04	-0.13	0.04	0.97	-0.25	-0.13	-0.01	-0.13	0.04	0.95
79767	ELMO3	-0.01	0.16	0.35	0.16	0.07	0.92	0.02	0.15	0.30	0.15	0.06	0.91	-0.01	0.17	0.34	0.17	0.07	0.94
79822	ARHGAP28	-0.39	-0.18	0.07	-0.18	0.07	0.95	-0.34	-0.19	-0.04	-0.18	0.06	0.99	-0.39	-0.20	-0.03	-0.20	0.07	0.98
79874	RABEP2	-0.05	0.16	0.35	0.16	0.06	0.93	0.06	0.17	0.28	0.17	0.05	0.98	0.04	0.19	0.36	0.18	0.06	0.98
79884	MAP9	-0.02	0.16	0.36	0.16	0.07	0.90	0.03	0.16	0.32	0.16	0.06	0.90	0.00	0.17	0.34	0.18	0.07	0.96
79887	PLBD1	0.00	0.12	0.27	0.12	0.04	0.91	0.02	0.12	0.22	0.11	0.04	0.93	0.01	0.12	0.23	0.12	0.04	0.94
79930	DOK3	-0.02	0.17	0.38	0.18	0.07	0.94	0.02	0.17	0.34	0.17	0.06	0.95	0.03	0.20	0.35	0.20	0.07	0.98
79956	ERMP1	-0.01	0.13	0.27	0.13	0.05	0.92	0.03	0.13	0.24	0.13	0.04	0.95	-0.01	0.14	0.28	0.15	0.05	0.97
79957	PAQR6	0.01	0.12	0.21	0.11	0.04	0.91	0.01	0.13	0.21	0.12	0.04	0.94	0.02	0.12	0.23	0.12	0.04	0.93
79962	DNAJC22	-0.01	0.13	0.26	0.13	0.05	0.91	0.04	0.14	0.21	0.13	0.04	0.92	-0.01	0.15	0.26	0.15	0.05	0.95
79990	PLEKHH3	-0.09	0.17	0.40	0.17	0.08	0.91	-0.03	0.17	0.36	0.17	0.07	0.94	-0.04	0.19	0.38	0.20	0.07	0.96
79993	ELOVL7	-0.01	0.16	0.30	0.16	0.05	0.96	0.05	0.17	0.28	0.17	0.04	0.98	0.00	0.18	0.29	0.18	0.05	0.99
80020	FOXRED2	-0.38	-0.21	0.01	-0.21	0.07	0.98	-0.36	-0.22	-0.05	-0.22	0.06	0.99	-0.39	-0.24	-0.05	-0.24	0.06	0.99
80036	TRPM3	-0.06	0.19	0.42	0.19	0.08	0.94	0.03	0.20	0.37	0.20	0.07	0.99	0.02	0.21	0.41	0.21	0.07	0.99
80119	PIF1	-0.04	0.22	0.47	0.22	0.08	0.96	0.08	0.24	0.42	0.24	0.07	1.00	0.03	0.25	0.44	0.26	0.08	0.99
80176	SPSB1	0.00	0.17	0.33	0.17	0.05	0.97	0.04	0.18	0.28	0.18	0.05	0.98	0.00	0.20	0.32	0.20	0.05	0.99
80179	MYO19	-0.12	0.18	0.44	0.18	0.08	0.92	0.04	0.18	0.37	0.19	0.07	0.97	-0.04	0.19	0.42	0.20	0.08	0.97
80307	FER1L4	-0.03	0.23	0.47	0.23	0.08	0.98	0.07	0.25	0.42	0.25	0.07	0.99	0.03	0.26	0.46	0.27	0.07	1.00
80323	CCDC68	-0.38	-0.18	0.07	-0.18	0.07	0.95	-0.31	-0.18	-0.04	-0.19	0.06	0.99	-0.37	-0.20	-0.04	-0.21	0.06	0.99
80774	LIMD2	-0.27	-0.15	0.01	-0.14	0.05	0.96	-0.26	-0.17	-0.04	-0.16	0.04	0.98	-0.28	-0.16	-0.02	-0.16	0.05	0.99
80818	ZNF436	-0.32	-0.15	0.05	-0.15	0.06	0.94	-0.30	-0.16	0.01	-0.16	0.06	0.95	-0.33	-0.18	-0.01	-0.18	0.06	0.97
80820	EEPD1	-0.11	0.21	0.48	0.21	0.09	0.95	0.06	0.22	0.42	0.22	0.08	0.97	0.00	0.23	0.47	0.24	0.08	0.98
80852	GRIP2	0.01	0.12	0.28	0.13	0.05	0.92	0.02	0.13	0.25	0.13	0.04	0.92	0.02	0.13	0.24	0.13	0.04	0.94
80853	KDM7A	-0.04	0.15	0.35	0.15	0.06	0.91	0.01	0.14	0.27	0.15	0.06	0.91	-0.02	0.16	0.32	0.16	0.06	0.95
80854	SETD7	-0.02	0.19	0.39	0.19	0.07	0.97	0.04	0.19	0.32	0.19	0.06	0.98	0.05	0.20	0.38	0.20	0.06	0.98
81033	KCNH6	-0.04	0.18	0.42	0.18	0.08	0.93	0.04	0.19	0.36	0.19	0.06	0.98	0.03	0.20	0.38	0.21	0.07	0.98
81539	SLC38A1	-0.02	0.16	0.34	0.16	0.06	0.92	0.02	0.17	0.30	0.17	0.06	0.91	0.03	0.18	0.30	0.18	0.06	0.98
81552	VOPP1	-0.33	-0.16	0.03	-0.16	0.06	0.93	-0.27	-0.17	-0.05	-0.17	0.05	0.95	-0.31	-0.18	0.01	-0.18	0.06	0.98
81602	CDADC1	-0.08	0.15	0.37	0.15	0.07	0.90	0.02	0.15	0.32	0.15	0.06	0.91	0.00	0.17	0.34	0.17	0.06	0.97
81605	URM1	-0.10	0.18	0.47	0.18	0.08	0.92	0.00	0.18	0.39	0.19	0.07	0.95	-0.03	0.20	0.43	0.21	0.08	0.98
81619	TSPAN14	-0.31	-0.14	0.01	-0.14	0.05	0.90	-0.27	-0.15	-0.03	-0.14	0.05	0.96	-0.28	-0.15	0.04	-0.15	0.05	0.95
81849	ST6GALNAC5	-0.44	-0.21	0.04	-0.21	0.08	0.96	-0.36	-0.23	-0.04	-0.23	0.07	0.98	-0.45	-0.24	-0.06	-0.24	0.07	0.99
83445	GSG1	-0.44	-0.17	0.10	-0.18	0.08	0.93	-0.34	-0.18	-0.04	-0.19	0.07	0.94	-0.43	-0.20	-0.02	-0.21	0.08	0.98
83468	GLT8D2	-0.32	-0.17	-0.01	-0.18	0.06	0.97	-0.33	-0.19	-0.07	-0.19	0.05	1.00	-0.35	-0.21	-0.06	-0.21	0.06	0.99
83482	SCRT1	-0.07	0.20	0.46	0.20	0.09	0.94	0.02	0.20	0.38	0.20	0.08	0.95	0.01	0.22	0.43	0.22	0.08	0.98
83547	RILP	-0.01	0.17	0.39	0.18	0.07	0.93	0.05	0.18	0.35	0.18	0.06	0.97	0.03	0.20	0.36	0.20	0.07	0.96
83660	TLN2	-0.36	-0.19	0.00	-0.19	0.06	0.98	-0.34	-0.20	-0.07	-0.20	0.05	0.99	-0.35	-0.22	-0.02	-0.22	0.06	0.99
83690	CRISPLD1	-0.04	0.15	0.31	0.16	0.06	0.93	-0.04	0.15	0.26	0.15	0.05	0.94	0.03	0.18	0.34	0.18	0.05	0.98
83692	CD99L2	-0.05	0.17	0.40	0.17	0.08	0.91	0.03	0.18	0.33	0.18	0.07	0.94	0.01	0.19	0.36	0.19	0.07	0.97
83707	TRPT1	0.00	0.14	0.31	0.15	0.06	0.91	0.04	0.14	0.28	0.14	0.05	0.92	0.02	0.16	0.30	0.16	0.06	0.96
83714	NRIP2	-0.04	0.15	0.29	0.15	0.06	0.94	0.05	0.16	0.25	0.15	0.05	0.98	0.03	0.17	0.31	0.17	0.05	0.98
83723	FAM57B	0.01	0.15	0.31	0.15	0.06	0.93	0.05	0.15	0.31	0.15	0.05	0.98	0.03	0.16	0.33	0.16	0.06	0.95
83787	ARMC10	-0.34	-0.16	0.02	-0.16	0.06	0.93	-0.31	-0.16	-0.01	-0.16	0.06	0.95	-0.32	-0.19	0.04	-0.19	0.06	0.98
83875	BCO2	-0.06	0.17	0.37	0.18	0.06	0.95	0.05	0.17	0.30	0.17	0.05	0.99	0.02	0.19	0.36	0.19	0.06	0.98
83937	RASSF4	-0.11	0.16	0.42	0.17	0.08	0.91	0.01	0.16	0.34	0.17	0.07	0.92	-0.03	0.18	0.39	0.19	0.07	0.97
83986	FAM234A	-0.03	0.12	0.23	0.12	0.04	0.91	0.04	0.13	0.20	0.13	0.03	0.96	-0.03	0.14	0.23	0.14	0.04	0.97
83992	CTTNBP2	-0.05	0.16	0.34	0.15	0.06	0.92	0.02	0.15	0.28	0.15	0.06	0.93	-0.03	0.17	0.32	0.17	0.06	0.96
84034	EMILIN2	0.02	0.20	0.36	0.20	0.06	0.99	0.12	0.23	0.33	0.22	0.05	1.00	0.08	0.23	0.36	0.23	0.05	1.00
84063	KIRREL2	-0.46	-0.18	0.11	-0.18	0.09	0.93	-0.38	-0.19	0.03	-0.19	0.08	0.96	-0.43	-0.20	0.01	-0.21	0.08	0.97
84109	QRFPR	-0.08	0.18	0.42	0.18	0.07	0.95	0.04	0.19	0.35	0.19	0.06	0.98	0.01	0.20	0.41	0.20	0.07	0.98
84187	TMEM164	-0.35	-0.16	0.06	-0.16	0.07	0.92	-0.29	-0.17	-0.03	-0.16	0.05	0.94	-0.34	-0.17	-0.01	-0.18	0.06	0.97
84221	SPATC1L	-0.34	-0.16	0.02	-0.16	0.06	0.96	-0.27	-0.17	-0.04	-0.17	0.05	0.95	-0.32	-0.19	-0.04	-0.19	0.05	0.99
84314	TMEM107	-0.26	-0.14	-0.02	-0.14	0.05	0.94	-0.25	-0.15	-0.05	-0.15	0.04	0.97	-0.26	-0.15	-0.02	-0.16	0.05	0.97
84332	DYDC2	-0.43	-0.17	0.09	-0.17	0.08	0.91	-0.38	-0.19	0.00	-0.19	0.08	0.96	-0.42	-0.20	0.03	-0.21	0.08	0.98
84439	HHIPL1	-0.39	-0.18	0.04	-0.18	0.07	0.94	-0.32	-0.19	-0.06	-0.19	0.06	0.98	-0.37	-0.19	-0.02	-0.19	0.07	0.98
84525	HOPX	-0.31	-0.16	0.04	-0.16	0.05	0.96	-0.26	-0.17	-0.06	-0.17	0.05	0.98	-0.31	-0.19	-0.03	-0.19	0.05	0.99
84542	KIAA1841	-0.06	0.16	0.36	0.16	0.06	0.93	0.01	0.16	0.31	0.16	0.06	0.94	0.00	0.19	0.36	0.19	0.06	0.98
84572	GNPTG	-0.03	0.13	0.31	0.14	0.05	0.91	0.04	0.14	0.26	0.14	0.05	0.95	0.02	0.15	0.31	0.16	0.05	0.96
84623	KIRREL3	-0.36	-0.19	0.00	-0.19	0.06	0.96	-0.33	-0.19	-0.03	-0.19	0.06	0.97	-0.36	-0.21	-0.07	-0.22	0.06	1.00
84691	FAM71F1	-0.44	-0.17	0.08	-0.17	0.08	0.91	-0.35	-0.17	-0.01	-0.17	0.07	0.91	-0.37	-0.19	0.01	-0.20	0.07	0.98
84735	CNDP1	0.01	0.13	0.25	0.12	0.04	0.92	0.05	0.14	0.22	0.14	0.04	0.96	-0.01	0.15	0.24	0.15	0.04	0.98
84769	MPV17L2	-0.37	-0.17	0.03	-0.17	0.06	0.94	-0.32	-0.18	-0.04	-0.17	0.06	0.99	-0.32	-0.18	-0.01	-0.19	0.06	0.98
84803	GPAT3	-0.05	0.20	0.47	0.20	0.09	0.94	0.03	0.21	0.40	0.21	0.08	0.96	0.04	0.23	0.45	0.23	0.08	0.98
84812	PLCD4	-0.06	0.19	0.44	0.19	0.08	0.94	0.04	0.19	0.35	0.19	0.07	0.97	0.04	0.21	0.43	0.21	0.07	0.98
84864	RIOX2	-0.38	-0.15	0.07	-0.15	0.07	0.90	-0.31	-0.15	-0.02	-0.15	0.06	0.92	-0.32	-0.16	0.01	-0.16	0.07	0.95
84936	ZFYVE19	-0.03	0.18	0.40	0.18	0.07	0.94	0.02	0.18	0.34	0.18	0.06	0.97	0.03	0.19	0.38	0.20	0.07	0.97
84937	ZNRF1	-0.32	-0.17	0.01	-0.17	0.05	0.97	-0.29	-0.19	-0.08	-0.19	0.05	1.00	-0.33	-0.20	-0.02	-0.20	0.05	0.99
84957	RELT	-0.03	0.15	0.33	0.15	0.06	0.92	0.03	0.15	0.26	0.15	0.05	0.94	0.01	0.16	0.34	0.16	0.06	0.95
85352	SHISAL1	-0.36	-0.17	0.01	-0.18	0.07	0.96	-0.32	-0.19	-0.07	-0.19	0.06	1.00	-0.35	-0.20	-0.04	-0.21	0.06	0.99
85461	TANC1	0.01	0.15	0.31	0.15	0.05	0.95	0.04	0.16	0.27	0.15	0.04	0.97	-0.01	0.17	0.29	0.17	0.05	0.98
87178	PNPT1	-0.01	0.13	0.25	0.13	0.05	0.91	0.05	0.13	0.24	0.13	0.04	0.92	-0.03	0.15	0.26	0.14	0.05	0.97
88455	ANKRD13A	-0.02	0.13	0.27	0.13	0.05	0.90	0.05	0.14	0.22	0.13	0.04	0.95	-0.02	0.15	0.25	0.15	0.04	0.98
89782	LMLN	-0.04	0.16	0.36	0.16	0.07	0.92	0.03	0.16	0.31	0.16	0.06	0.93	0.01	0.18	0.37	0.18	0.06	0.98
89846	FGD3	-0.34	-0.15	0.06	-0.15	0.06	0.92	-0.30	-0.17	-0.05	-0.17	0.06	0.97	-0.31	-0.18	0.00	-0.18	0.06	0.98
90102	PHLDB2	-0.02	0.15	0.29	0.15	0.05	0.94	-0.01	0.17	0.29	0.17	0.05	0.98	0.01	0.18	0.30	0.18	0.05	0.99
90488	TMEM263	-0.45	-0.18	0.11	-0.17	0.08	0.92	-0.39	-0.18	-0.01	-0.18	0.08	0.93	-0.42	-0.20	0.01	-0.21	0.08	0.97
90523	MLIP	-0.32	-0.18	-0.03	-0.18	0.05	0.98	-0.28	-0.19	-0.06	-0.19	0.05	0.99	-0.32	-0.21	-0.07	-0.21	0.05	1.00
90850	ZNF598	0.01	0.15	0.32	0.15	0.05	0.94	0.05	0.16	0.27	0.16	0.05	0.97	-0.03	0.17	0.27	0.17	0.05	0.98
90861	JPT2	0.00	0.15	0.24	0.15	0.04	0.98	0.05	0.16	0.24	0.16	0.04	0.99	0.02	0.17	0.26	0.17	0.04	1.00
91133	L3MBTL4	-0.32	-0.16	0.02	-0.16	0.06	0.94	-0.27	-0.18	-0.06	-0.17	0.05	0.98	-0.30	-0.18	-0.02	-0.18	0.05	0.98
91252	SLC39A13	-0.04	0.20	0.44	0.20	0.08	0.95	0.05	0.21	0.38	0.21	0.07	0.99	-0.01	0.23	0.41	0.23	0.07	0.99
91624	NEXN	-0.03	0.20	0.40	0.20	0.07	0.96	0.07	0.21	0.35	0.20	0.06	1.00	0.05	0.23	0.41	0.23	0.07	0.99
91683	SYT12	-0.06	0.17	0.38	0.17	0.07	0.93	0.02	0.18	0.34	0.18	0.06	0.94	-0.01	0.19	0.35	0.19	0.07	0.96
92293	TMEM132C	-0.02	0.15	0.34	0.15	0.06	0.92	0.05	0.15	0.30	0.15	0.05	0.92	0.02	0.16	0.28	0.16	0.05	0.96
92335	STRADA	-0.01	0.16	0.34	0.16	0.06	0.94	0.04	0.17	0.29	0.16	0.05	0.97	0.00	0.18	0.32	0.18	0.05	0.98
92399	MRRF	-0.03	0.15	0.34	0.16	0.07	0.91	0.02	0.16	0.28	0.16	0.05	0.94	0.02	0.17	0.37	0.17	0.07	0.97
92597	MOB1B	-0.39	-0.17	0.09	-0.17	0.07	0.93	-0.34	-0.18	-0.03	-0.18	0.06	0.97	-0.39	-0.19	-0.03	-0.20	0.07	0.99
92610	TIFA	-0.05	0.18	0.40	0.18	0.08	0.93	0.02	0.18	0.34	0.17	0.07	0.93	0.00	0.19	0.40	0.19	0.07	0.96
93587	TRMT10A	0.00	0.14	0.32	0.14	0.06	0.92	0.04	0.14	0.26	0.14	0.05	0.93	0.00	0.16	0.30	0.16	0.05	0.98
113263	GLCCI1	-0.08	0.18	0.46	0.18	0.09	0.91	0.02	0.19	0.37	0.19	0.07	0.96	-0.01	0.19	0.40	0.20	0.08	0.96
113452	TMEM54	-0.36	-0.15	0.05	-0.16	0.07	0.93	-0.29	-0.17	-0.02	-0.16	0.06	0.94	-0.37	-0.17	0.00	-0.18	0.06	0.97
113675	SDSL	0.03	0.18	0.37	0.19	0.07	0.96	0.07	0.18	0.33	0.19	0.06	0.99	0.05	0.21	0.36	0.21	0.06	0.99
113829	SLC35A4	-0.04	0.15	0.37	0.16	0.07	0.91	-0.01	0.15	0.31	0.16	0.06	0.92	0.00	0.18	0.34	0.18	0.06	0.97
114571	SLC22A9	-0.36	-0.16	0.00	-0.16	0.06	0.94	-0.31	-0.17	-0.07	-0.18	0.05	0.99	-0.37	-0.18	-0.02	-0.18	0.06	0.99
114787	GPRIN1	-0.35	-0.19	-0.05	-0.19	0.06	0.96	-0.32	-0.19	-0.08	-0.20	0.06	1.00	-0.36	-0.21	-0.05	-0.21	0.06	1.00
114800	CCDC85A	-0.29	-0.13	-0.02	-0.14	0.05	0.94	-0.22	-0.13	-0.04	-0.13	0.04	0.95	-0.24	-0.14	0.02	-0.13	0.04	0.95
114801	TMEM200A	-0.40	-0.16	0.09	-0.16	0.08	0.90	-0.34	-0.17	-0.01	-0.17	0.07	0.94	-0.39	-0.17	0.05	-0.18	0.07	0.95
114804	RNF157	-0.04	0.15	0.40	0.16	0.07	0.91	-0.02	0.16	0.32	0.16	0.07	0.91	0.01	0.17	0.36	0.18	0.07	0.96
114880	OSBPL6	-0.07	0.17	0.45	0.18	0.08	0.92	0.01	0.18	0.36	0.18	0.07	0.92	-0.03	0.19	0.38	0.20	0.08	0.97
114990	VASN	-0.27	-0.16	-0.01	-0.15	0.05	0.96	-0.26	-0.16	-0.08	-0.16	0.04	1.00	-0.28	-0.17	-0.03	-0.17	0.05	0.99
115584	SLC5A11	0.00	0.12	0.23	0.12	0.04	0.90	0.04	0.14	0.20	0.13	0.04	0.97	-0.01	0.14	0.24	0.14	0.04	0.98
116028	RMI2	-0.31	-0.15	0.04	-0.15	0.06	0.95	-0.28	-0.16	0.00	-0.16	0.05	0.98	-0.30	-0.19	0.00	-0.18	0.05	0.99
116135	LRRC3B	-0.45	-0.20	0.07	-0.20	0.08	0.94	-0.37	-0.21	-0.04	-0.21	0.07	0.98	-0.43	-0.23	0.01	-0.23	0.08	0.98
116535	MRGPRF	-0.06	0.16	0.35	0.16	0.07	0.93	0.01	0.18	0.32	0.17	0.06	0.93	-0.01	0.19	0.34	0.19	0.06	0.98
116832	RPL39L	-0.28	-0.13	0.00	-0.14	0.05	0.92	-0.28	-0.15	0.00	-0.15	0.05	0.95	-0.30	-0.18	-0.01	-0.17	0.05	0.98
116966	WDR17	-0.26	-0.13	0.02	-0.13	0.05	0.91	-0.23	-0.13	-0.05	-0.14	0.04	0.96	-0.26	-0.14	-0.01	-0.14	0.05	0.96
117154	DACH2	-0.43	-0.18	0.12	-0.18	0.08	0.94	-0.37	-0.18	-0.01	-0.19	0.07	0.96	-0.42	-0.21	0.02	-0.21	0.08	0.98
117245	HRASLS5	-0.28	-0.13	0.01	-0.14	0.05	0.93	-0.23	-0.15	-0.05	-0.15	0.04	0.98	-0.27	-0.16	-0.03	-0.16	0.04	0.98
118427	OLFM3	-0.35	-0.16	0.09	-0.16	0.07	0.93	-0.28	-0.17	-0.02	-0.17	0.06	0.97	-0.36	-0.19	0.01	-0.18	0.07	0.96
119587	CPXM2	0.01	0.16	0.30	0.16	0.05	0.97	0.08	0.18	0.30	0.18	0.04	1.00	0.05	0.18	0.31	0.18	0.05	0.99
122622	ADSSL1	0.00	0.15	0.32	0.15	0.05	0.96	0.05	0.17	0.26	0.16	0.05	0.98	-0.03	0.17	0.30	0.18	0.05	0.99
122953	JDP2	-0.08	0.18	0.45	0.19	0.09	0.93	0.02	0.20	0.38	0.20	0.08	0.95	-0.01	0.21	0.42	0.22	0.08	0.97
124976	SPNS2	-0.04	0.15	0.35	0.15	0.06	0.90	0.03	0.15	0.28	0.14	0.05	0.93	-0.04	0.16	0.30	0.17	0.06	0.95
126755	LRRC38	0.00	0.16	0.37	0.17	0.07	0.93	0.04	0.17	0.32	0.17	0.05	0.97	0.04	0.19	0.36	0.19	0.06	0.97
127003	C1orf194	-0.27	-0.13	-0.01	-0.13	0.05	0.90	-0.24	-0.15	-0.06	-0.15	0.04	0.96	-0.27	-0.16	-0.05	-0.16	0.05	0.98
127833	SYT2	-0.01	0.24	0.50	0.24	0.09	0.96	0.08	0.25	0.45	0.25	0.08	1.00	0.07	0.27	0.47	0.27	0.08	0.99
128434	VSTM2L	-0.41	-0.17	0.12	-0.17	0.08	0.91	-0.29	-0.18	-0.03	-0.17	0.06	0.95	-0.36	-0.19	0.02	-0.18	0.07	0.94
130399	ACVR1C	0.00	0.20	0.41	0.20	0.07	0.96	0.04	0.22	0.40	0.21	0.06	0.99	0.06	0.22	0.39	0.23	0.07	0.99
132160	PPM1M	-0.45	-0.19	0.01	-0.19	0.08	0.93	-0.38	-0.20	-0.05	-0.20	0.07	0.97	-0.41	-0.22	-0.01	-0.23	0.08	0.98
132321	C4orf33	-0.09	0.19	0.42	0.19	0.08	0.94	0.02	0.19	0.37	0.19	0.07	0.98	0.02	0.22	0.42	0.22	0.07	0.98
133418	EMB	0.00	0.17	0.36	0.17	0.06	0.96	0.02	0.17	0.30	0.17	0.05	0.97	0.04	0.19	0.34	0.19	0.06	0.98
134548	SOWAHA	-0.39	-0.20	-0.02	-0.20	0.07	0.97	-0.35	-0.20	-0.03	-0.20	0.06	0.99	-0.37	-0.22	-0.06	-0.23	0.06	0.99
138428	PTRH1	-0.31	-0.14	0.03	-0.14	0.05	0.92	-0.26	-0.15	-0.06	-0.15	0.05	0.99	-0.27	-0.15	-0.02	-0.16	0.05	0.98
139221	MUM1L1	-0.46	-0.19	0.11	-0.19	0.08	0.94	-0.34	-0.18	-0.02	-0.19	0.07	0.96	-0.45	-0.21	-0.01	-0.21	0.08	0.98
139411	PTCHD1	-0.42	-0.18	0.01	-0.19	0.07	0.95	-0.35	-0.19	-0.03	-0.19	0.07	0.98	-0.38	-0.21	-0.03	-0.21	0.07	0.99
139728	PNCK	-0.41	-0.17	0.09	-0.17	0.07	0.93	-0.33	-0.17	-0.02	-0.17	0.07	0.95	-0.35	-0.18	0.00	-0.19	0.07	0.98
140733	MACROD2	-0.27	-0.14	-0.01	-0.14	0.05	0.96	-0.26	-0.16	0.01	-0.16	0.05	0.98	-0.31	-0.18	-0.01	-0.18	0.05	0.99
143279	HECTD2	-0.31	-0.15	0.01	-0.15	0.06	0.94	-0.28	-0.16	-0.05	-0.16	0.05	0.98	-0.32	-0.18	-0.05	-0.18	0.05	0.98
144348	ZNF664	0.01	0.14	0.24	0.14	0.04	0.96	0.01	0.14	0.23	0.14	0.04	0.98	0.00	0.14	0.25	0.14	0.04	0.97
144402	CPNE8	-0.35	-0.18	-0.01	-0.18	0.06	0.98	-0.32	-0.18	-0.06	-0.19	0.06	0.99	-0.33	-0.20	-0.02	-0.19	0.06	0.99
146760	RTN4RL1	-0.27	-0.13	0.02	-0.13	0.05	0.93	-0.22	-0.13	0.01	-0.13	0.05	0.93	-0.25	-0.15	-0.02	-0.15	0.04	0.97
147463	ANKRD29	-0.01	0.19	0.40	0.19	0.07	0.95	0.04	0.20	0.36	0.19	0.07	0.95	0.03	0.21	0.38	0.21	0.07	0.99
147968	CAPN12	-0.32	-0.16	0.02	-0.16	0.06	0.93	-0.28	-0.17	-0.01	-0.17	0.05	0.97	-0.30	-0.18	0.00	-0.18	0.06	0.98
148014	TTC9B	-0.25	-0.13	0.01	-0.13	0.04	0.92	-0.27	-0.14	-0.03	-0.15	0.04	0.97	-0.26	-0.15	-0.03	-0.15	0.05	0.98
148281	SYT6	-0.04	0.18	0.41	0.17	0.08	0.91	-0.01	0.19	0.32	0.18	0.06	0.94	-0.01	0.20	0.36	0.20	0.07	0.98
149473	CCDC24	-0.29	-0.14	0.00	-0.14	0.05	0.93	-0.25	-0.14	-0.01	-0.14	0.04	0.96	-0.28	-0.15	-0.01	-0.15	0.05	0.95
150209	AIFM3	-0.06	0.15	0.43	0.15	0.07	0.90	0.01	0.16	0.31	0.16	0.06	0.90	0.01	0.18	0.36	0.18	0.06	0.97
151516	ASPRV1	0.01	0.12	0.23	0.12	0.04	0.91	0.04	0.13	0.21	0.13	0.04	0.97	-0.02	0.14	0.22	0.14	0.04	0.98
152189	CMTM8	-0.33	-0.15	0.01	-0.15	0.06	0.91	-0.31	-0.17	-0.07	-0.17	0.05	0.99	-0.33	-0.18	-0.01	-0.19	0.06	0.98
152273	FGD5	-0.01	0.15	0.33	0.16	0.06	0.93	0.04	0.17	0.29	0.16	0.05	0.97	-0.02	0.18	0.30	0.17	0.05	0.98
152940	C4orf45	-0.32	-0.17	0.03	-0.17	0.06	0.96	-0.28	-0.19	-0.05	-0.19	0.05	0.98	-0.33	-0.21	-0.07	-0.21	0.05	0.99
154141	MBOAT1	-0.02	0.12	0.22	0.12	0.04	0.91	0.04	0.14	0.23	0.14	0.03	0.99	0.02	0.15	0.28	0.15	0.04	0.98

154790	CLEC2L	0.00	0.15	0.32	0.15	0.06	0.92	0.04	0.15	0.29	0.15	0.05	0.95	0.02	0.16	0.30	0.16	0.05	0.95
160760	PPTC7	0.01	0.19	0.42	0.19	0.07	0.95	0.06	0.20	0.36	0.20	0.06	0.98	0.05	0.22	0.38	0.22	0.07	0.99
162494	RHBDL3	-0.08	0.21	0.51	0.21	0.09	0.94	0.03	0.21	0.40	0.21	0.08	0.97	0.02	0.23	0.46	0.24	0.08	0.98
163183	SYNE4	-0.10	0.19	0.46	0.20	0.08	0.94	0.04	0.19	0.38	0.20	0.07	0.95	0.00	0.21	0.45	0.22	0.08	0.99
163732	CITED4	-0.28	-0.14	0.02	-0.13	0.05	0.92	-0.22	-0.14	-0.02	-0.14	0.04	0.95	-0.27	-0.15	0.00	-0.14	0.05	0.95
163782	KANK4	0.00	0.21	0.38	0.21	0.06	0.99	0.07	0.23	0.34	0.23	0.05	1.00	0.08	0.25	0.38	0.24	0.06	1.00
165215	FAM171B	-0.38	-0.17	0.05	-0.17	0.07	0.93	-0.32	-0.17	-0.02	-0.17	0.06	0.97	-0.36	-0.19	0.02	-0.19	0.07	0.98
166752	FREM3	-0.47	-0.17	0.18	-0.17	0.09	0.90	-0.32	-0.17	0.01	-0.17	0.07	0.93	-0.44	-0.18	0.03	-0.19	0.08	0.93
167681	PRSS35	-0.41	-0.16	0.08	-0.17	0.08	0.90	-0.34	-0.17	0.01	-0.17	0.07	0.94	-0.39	-0.18	0.02	-0.19	0.08	0.95
167691	LCA5	-0.08	0.17	0.40	0.17	0.07	0.92	0.02	0.17	0.35	0.17	0.07	0.95	0.02	0.19	0.38	0.20	0.07	0.97
170261	ZCCHC12	-0.46	-0.18	0.04	-0.18	0.08	0.93	-0.39	-0.18	0.01	-0.18	0.08	0.92	-0.41	-0.21	0.00	-0.21	0.08	0.98
170850	KCNG3	-0.37	-0.16	0.05	-0.16	0.07	0.94	-0.27	-0.16	-0.04	-0.16	0.06	0.95	-0.37	-0.18	-0.03	-0.18	0.06	0.97
192668	CYS1	-0.26	-0.15	-0.03	-0.15	0.05	0.97	-0.26	-0.16	-0.06	-0.16	0.04	0.99	-0.29	-0.18	-0.05	-0.18	0.05	0.99
196383	RILPL2	-0.46	-0.20	0.05	-0.20	0.08	0.94	-0.39	-0.21	-0.05	-0.21	0.07	0.99	-0.43	-0.22	-0.03	-0.23	0.08	0.98
196527	ANO6	0.01	0.12	0.22	0.12	0.04	0.93	0.03	0.11	0.19	0.12	0.04	0.92	0.01	0.13	0.22	0.13	0.03	0.97
200058	FLJ23867	-0.06	0.18	0.40	0.18	0.07	0.94	0.03	0.19	0.35	0.18	0.07	0.96	0.03	0.20	0.38	0.20	0.07	0.98
200942	KLHDC8B	-0.43	-0.19	0.07	-0.19	0.08	0.94	-0.36	-0.18	-0.03	-0.18	0.07	0.98	-0.41	-0.20	-0.01	-0.21	0.08	0.98
201191	SAMD14	-0.31	-0.14	0.02	-0.14	0.06	0.92	-0.25	-0.17	-0.03	-0.16	0.05	0.94	-0.30	-0.16	0.00	-0.16	0.05	0.96
202333	CMYA5	-0.07	0.19	0.42	0.19	0.08	0.94	0.01	0.21	0.37	0.20	0.07	0.97	0.01	0.22	0.40	0.23	0.07	0.99
203286	ANKS6	-0.01	0.17	0.38	0.17	0.07	0.94	0.05	0.18	0.32	0.18	0.06	0.97	-0.03	0.19	0.33	0.19	0.06	0.99
219348	PLAC9	-0.35	-0.15	0.04	-0.15	0.07	0.91	-0.27	-0.16	-0.04	-0.15	0.06	0.94	-0.34	-0.16	-0.02	-0.17	0.07	0.95
220164	DOK6	-0.44	-0.16	0.10	-0.17	0.08	0.91	-0.36	-0.17	-0.01	-0.17	0.07	0.95	-0.42	-0.18	0.01	-0.19	0.08	0.96
220202	ATOH7	-0.44	-0.19	0.09	-0.19	0.08	0.94	-0.39	-0.20	-0.01	-0.20	0.08	0.96	-0.42	-0.22	0.02	-0.23	0.08	0.99
221294	NT5DC1	-0.02	0.19	0.36	0.19	0.06	0.98	0.08	0.20	0.31	0.20	0.05	1.00	0.00	0.22	0.33	0.21	0.05	0.99
221336	BEND6	-0.08	0.17	0.44	0.17	0.08	0.92	0.02	0.18	0.35	0.17	0.07	0.94	-0.01	0.19	0.39	0.20	0.08	0.97
221421	RSPH9	-0.45	-0.19	0.03	-0.19	0.08	0.94	-0.38	-0.19	-0.04	-0.19	0.07	0.97	-0.39	-0.21	-0.02	-0.21	0.07	0.98
221662	RBM24	-0.21	-0.12	0.00	-0.12	0.04	0.93	-0.19	-0.12	-0.04	-0.12	0.03	0.93	-0.24	-0.13	-0.03	-0.13	0.04	0.95
222537	HS3ST5	-0.01	0.20	0.38	0.20	0.07	0.97	0.08	0.22	0.36	0.22	0.06	1.00	0.04	0.23	0.38	0.23	0.06	0.99
253832	ZDHHC20	0.01	0.15	0.28	0.15	0.05	0.96	0.05	0.16	0.27	0.16	0.04	0.99	-0.02	0.18	0.28	0.17	0.05	0.99
254102	EHBP1L1	-0.35	-0.21	-0.01	-0.21	0.06	0.99	-0.34	-0.23	-0.08	-0.22	0.05	1.00	-0.37	-0.24	-0.07	-0.24	0.05	1.00
254170	FBXO33	-0.02	0.16	0.38	0.16	0.07	0.91	0.02	0.17	0.34	0.17	0.06	0.95	0.00	0.18	0.35	0.19	0.07	0.97
254263	CNIH2	-0.37	-0.15	0.06	-0.15	0.07	0.91	-0.30	-0.17	-0.04	-0.17	0.06	0.96	-0.34	-0.18	0.04	-0.18	0.07	0.97
254552	NUDT8	-0.02	0.12	0.28	0.13	0.05	0.90	0.02	0.12	0.25	0.12	0.05	0.91	0.00	0.14	0.27	0.14	0.05	0.95
256281	NUDT14	-0.42	-0.16	0.11	-0.16	0.08	0.90	-0.32	-0.17	-0.03	-0.17	0.07	0.93	-0.39	-0.18	0.00	-0.19	0.08	0.96
260434	PYDC1	-0.39	-0.18	0.02	-0.18	0.07	0.94	-0.34	-0.20	-0.04	-0.20	0.06	0.99	-0.38	-0.21	-0.01	-0.21	0.07	0.99
282969	FUOM	-0.31	-0.16	0.02	-0.16	0.06	0.94	-0.28	-0.18	-0.05	-0.18	0.05	0.99	-0.30	-0.18	-0.05	-0.18	0.05	0.99
282973	JAKMIP3	0.00	0.15	0.30	0.15	0.05	0.92	0.05	0.17	0.29	0.17	0.05	0.98	-0.01	0.17	0.32	0.18	0.05	0.99
283131	NEAT1	-0.01	0.12	0.26	0.12	0.04	0.93	0.04	0.13	0.23	0.13	0.04	0.96	-0.02	0.15	0.23	0.15	0.04	0.98
283143	LINC00900	-0.31	-0.15	0.02	-0.15	0.06	0.93	-0.27	-0.15	-0.02	-0.15	0.05	0.97	-0.30	-0.17	-0.02	-0.17	0.05	0.98
283209	PGM2L1	-0.40	-0.18	0.05	-0.18	0.07	0.94	-0.33	-0.18	-0.02	-0.18	0.06	0.97	-0.35	-0.20	0.02	-0.20	0.06	0.99
283284	IGSF22	-0.33	-0.17	-0.01	-0.17	0.06	0.96	-0.34	-0.19	-0.07	-0.19	0.06	0.99	-0.34	-0.20	-0.01	-0.20	0.06	0.99
283316	CD163L1	-0.03	0.15	0.29	0.15	0.05	0.94	0.07	0.15	0.27	0.15	0.05	1.00	0.02	0.16	0.30	0.16	0.05	0.96
284069	FAM171A2	-0.25	-0.12	0.00	-0.12	0.04	0.91	-0.23	-0.14	-0.02	-0.13	0.04	0.97	-0.26	-0.15	0.00	-0.15	0.04	0.98
284119	CAVIN1	0.02	0.12	0.21	0.12	0.04	0.93	0.05	0.13	0.20	0.13	0.03	0.98	-0.03	0.15	0.24	0.15	0.04	0.99
284339	TMEM145	-0.14	0.16	0.41	0.16	0.08	0.90	0.02	0.16	0.35	0.17	0.07	0.94	-0.02	0.18	0.41	0.18	0.08	0.95
284348	LYPD5	-0.06	0.20	0.47	0.20	0.08	0.94	0.03	0.21	0.40	0.20	0.08	0.96	0.00	0.22	0.40	0.22	0.08	0.98
284415	VSTM1	-0.01	0.18	0.34	0.17	0.06	0.96	0.01	0.17	0.30	0.17	0.05	0.97	0.06	0.19	0.33	0.19	0.05	1.00
284454	LOC284454	-0.02	0.15	0.28	0.15	0.05	0.95	0.01	0.16	0.30	0.16	0.05	0.98	-0.01	0.19	0.30	0.18	0.05	0.97
284485	RIIAD1	-0.39	-0.17	0.06	-0.17	0.07	0.93	-0.33	-0.18	-0.01	-0.18	0.06	0.98	-0.36	-0.19	0.01	-0.20	0.07	0.99
284611	FAM102B	-0.39	-0.16	0.08	-0.16	0.07	0.93	-0.31	-0.17	-0.04	-0.17	0.06	0.95	-0.36	-0.19	-0.04	-0.19	0.07	0.97
284716	RIMKLA	-0.01	0.18	0.41	0.18	0.08	0.93	0.04	0.19	0.36	0.19	0.07	0.95	0.03	0.21	0.38	0.21	0.07	0.98
285220	EPHA6	-0.24	-0.12	0.02	-0.12	0.04	0.91	-0.24	-0.13	-0.04	-0.13	0.04	0.95	-0.25	-0.13	-0.01	-0.13	0.04	0.94
285598	ARL10	-0.28	-0.14	0.01	-0.14	0.05	0.92	-0.27	-0.14	-0.04	-0.14	0.05	0.94	-0.28	-0.15	0.01	-0.15	0.05	0.95
285613	RELL2	-0.03	0.16	0.39	0.16	0.08	0.91	0.00	0.16	0.32	0.16	0.07	0.92	0.00	0.17	0.35	0.18	0.07	0.95
285755	PPIL6	-0.33	-0.15	0.03	-0.15	0.05	0.96	-0.27	-0.16	-0.03	-0.16	0.05	0.96	-0.31	-0.18	-0.02	-0.18	0.05	0.99
285780	LY86-AS1	-0.40	-0.19	0.07	-0.19	0.07	0.96	-0.32	-0.19	0.01	-0.19	0.06	0.97	-0.38	-0.21	-0.03	-0.21	0.07	0.98
286133	SCARA5	-0.49	-0.22	0.07	-0.22	0.09	0.96	-0.41	-0.24	-0.07	-0.24	0.08	1.00	-0.48	-0.26	-0.04	-0.26	0.08	0.99
286336	FAM78A	-0.03	0.16	0.40	0.17	0.07	0.91	0.01	0.17	0.35	0.17	0.07	0.90	0.02	0.19	0.34	0.19	0.07	0.96
326624	RAB37	-0.01	0.21	0.47	0.22	0.08	0.96	0.06	0.22	0.40	0.22	0.07	0.98	0.03	0.24	0.41	0.24	0.08	0.99
337876	CHSY3	-0.24	-0.11	0.00	-0.11	0.04	0.90	-0.18	-0.11	-0.04	-0.11	0.03	0.95	-0.21	-0.12	-0.03	-0.12	0.03	0.95
339829	CCDC39	-0.06	0.16	0.43	0.16	0.08	0.91	0.02	0.16	0.32	0.16	0.07	0.90	-0.01	0.18	0.35	0.18	0.07	0.95
339983	NAT8L	-0.03	0.17	0.41	0.18	0.08	0.91	0.02	0.18	0.35	0.18	0.07	0.95	0.01	0.21	0.38	0.20	0.07	0.98
340719	NANOS1	-0.41	-0.17	0.06	-0.17	0.08	0.92	-0.35	-0.19	-0.03	-0.18	0.07	0.96	-0.41	-0.20	0.01	-0.21	0.08	0.98
342667	STAC2	-0.03	0.21	0.48	0.22	0.09	0.95	0.04	0.23	0.42	0.23	0.07	0.99	0.02	0.25	0.43	0.25	0.08	0.99
347730	LRRTM1	-0.28	-0.14	0.03	-0.14	0.06	0.90	-0.29	-0.15	-0.03	-0.16	0.05	0.97	-0.32	-0.15	0.02	-0.15	0.06	0.92
347902	AMIGO2	-0.51	-0.17	0.14	-0.18	0.09	0.91	-0.37	-0.17	-0.01	-0.17	0.08	0.90	-0.44	-0.19	0.02	-0.20	0.09	0.94
348013	TMEM255B	-0.02	0.20	0.39	0.20	0.07	0.96	0.08	0.21	0.34	0.20	0.06	1.00	0.06	0.22	0.39	0.22	0.06	0.99
349136	WDR86	-0.50	-0.22	0.06	-0.23	0.09	0.95	-0.43	-0.25	-0.08	-0.24	0.08	1.00	-0.49	-0.25	-0.03	-0.26	0.09	0.99
353149	TBC1D26	-0.33	-0.15	0.02	-0.15	0.07	0.90	-0.31	-0.15	-0.03	-0.15	0.06	0.94	-0.33	-0.17	0.01	-0.17	0.06	0.96
373156	GSTK1	-0.04	0.14	0.30	0.15	0.05	0.93	0.04	0.15	0.26	0.15	0.05	0.96	0.03	0.17	0.28	0.17	0.05	0.99
373863	DND1	-0.07	0.16	0.39	0.16	0.07	0.92	0.02	0.17	0.32	0.17	0.06	0.93	-0.02	0.18	0.36	0.19	0.07	0.96
374378	GALNT18	-0.30	-0.15	0.01	-0.15	0.05	0.95	-0.24	-0.14	-0.03	-0.15	0.04	0.96	-0.27	-0.16	-0.01	-0.16	0.04	0.99
374875	HSD11B1L	-0.34	-0.16	0.09	-0.16	0.07	0.93	-0.29	-0.16	-0.02	-0.16	0.06	0.97	-0.37	-0.18	-0.01	-0.18	0.06	0.97
375057	STUM	-0.50	-0.20	0.07	-0.21	0.09	0.96	-0.40	-0.20	-0.02	-0.21	0.08	0.98	-0.41	-0.23	-0.02	-0.23	0.08	0.98
375449	MAST4	-0.04	0.16	0.39	0.17	0.07	0.93	0.00	0.18	0.33	0.18	0.07	0.94	0.01	0.19	0.37	0.20	0.07	0.98
386618	KCTD4	-0.43	-0.20	0.02	-0.21	0.08	0.96	-0.41	-0.22	-0.01	-0.22	0.07	0.98	-0.41	-0.23	-0.04	-0.24	0.07	0.99
387775	SLC22A10	-0.30	-0.13	0.02	-0.13	0.05	0.92	-0.27	-0.15	-0.05	-0.15	0.04	0.99	-0.33	-0.15	-0.02	-0.15	0.05	0.97
389073	C2orf80	-0.36	-0.17	0.05	-0.17	0.06	0.94	-0.32	-0.18	-0.06	-0.18	0.06	0.95	-0.37	-0.19	0.01	-0.20	0.06	0.99
389941	C1QL3	-0.44	-0.17	0.11	-0.17	0.08	0.91	-0.35	-0.16	0.00	-0.17	0.07	0.92	-0.39	-0.18	0.01	-0.19	0.08	0.95
400120	SERTM1	-0.37	-0.15	0.04	-0.15	0.06	0.90	-0.34	-0.15	-0.05	-0.15	0.06	0.91	-0.34	-0.18	-0.01	-0.18	0.06	0.98
400569	MED11	-0.04	0.17	0.36	0.17	0.07	0.93	0.03	0.17	0.30	0.16	0.06	0.96	0.03	0.19	0.36	0.19	0.06	0.98
400745	SH2D5	-0.42	-0.17	0.08	-0.18	0.08	0.95	-0.31	-0.18	0.00	-0.18	0.06	0.97	-0.41	-0.21	-0.01	-0.21	0.07	0.97
401647	GOLGA7B	-0.03	0.14	0.32	0.14	0.06	0.91	0.01	0.14	0.26	0.14	0.05	0.92	0.02	0.16	0.29	0.15	0.05	0.95
404037	HAPLN4	-0.02	0.16	0.40	0.17	0.08	0.93	0.03	0.18	0.35	0.18	0.07	0.94	-0.01	0.20	0.38	0.20	0.07	0.96
404217	CTXN1	-0.46	-0.21	0.07	-0.21	0.08	0.96	-0.40	-0.22	-0.02	-0.22	0.07	0.99	-0.42	-0.24	-0.01	-0.24	0.07	0.99
407738	FAM19A1	-0.31	-0.15	-0.01	-0.15	0.05	0.94	-0.28	-0.15	-0.04	-0.15	0.05	0.96	-0.29	-0.17	-0.05	-0.17	0.05	0.98
408263	FNDC9	-0.26	-0.13	0.05	-0.13	0.05	0.92	-0.23	-0.13	-0.04	-0.13	0.04	0.98	-0.27	-0.14	-0.02	-0.14	0.04	0.96
415116	PIM3	-0.04	0.18	0.39	0.18	0.08	0.93	0.04	0.19	0.34	0.18	0.07	0.96	-0.01	0.21	0.37	0.21	0.07	0.98
441108	C5orf56	-0.03	0.14	0.32	0.14	0.05	0.94	0.03	0.15	0.24	0.15	0.04	0.95	0.01	0.16	0.28	0.16	0.05	0.97
442319	ZNF727	-0.29	-0.13	0.02	-0.13	0.05	0.91	-0.26	-0.14	-0.02	-0.14	0.05	0.95	-0.29	-0.14	0.02	-0.14	0.05	0.95
494470	RNF165	-0.26	-0.15	-0.03	-0.15	0.04	0.96	-0.27	-0.16	-0.07	-0.15	0.04	0.99	-0.27	-0.17	-0.06	-0.17	0.04	0.99
503542	SPRN	-0.40	-0.19	0.04	-0.20	0.07	0.96	-0.33	-0.20	-0.02	-0.20	0.07	0.99	-0.39	-0.21	0.01	-0.22	0.07	0.99
574036	SERTAD4-AS1	-0.04	0.20	0.45	0.21	0.08	0.94	0.05	0.21	0.39	0.21	0.07	0.98	-0.02	0.23	0.43	0.23	0.08	0.99
642273	FAM110C	-0.50	-0.23	0.03	-0.24	0.09	0.98	-0.42	-0.24	-0.08	-0.25	0.08	1.00	-0.50	-0.26	-0.07	-0.27	0.08	1.00
642852	LOC642852	-0.34	-0.15	0.04	-0.15	0.06	0.92	-0.27	-0.16	-0.04	-0.16	0.05	0.95	-0.32	-0.17	0.00	-0.17	0.06	0.97
643037	C11orf97	-0.41	-0.17	0.09	-0.17	0.08	0.91	-0.36	-0.19	-0.04	-0.19	0.07	0.97	-0.41	-0.20	0.03	-0.21	0.08	0.98
646627	LYPD8	-0.44	-0.18	0.12	-0.18	0.09	0.92	-0.37	-0.19	-0.02	-0.19	0.07	0.98	-0.45	-0.21	-0.02	-0.21	0.08	0.95
654502	IQCJ	-0.30	-0.16	0.00	-0.16	0.05	0.96	-0.26	-0.16	-0.03	-0.16	0.05	0.99	-0.33	-0.18	-0.03	-0.18	0.05	0.99
654790	PCP4L1	0.00	0.16	0.36	0.16	0.07	0.91	0.05	0.17	0.32	0.17	0.06	0.95	0.04	0.18	0.31	0.18	0.06	0.98
100093630	SNHG8	-0.39	-0.15	0.05	-0.15	0.07	0.91	-0.33	-0.17	-0.04	-0.16	0.06	0.92	-0.36	-0.18	-0.03	-0.19	0.06	0.98
100170841	EPOP	-0.39	-0.17	0.07	-0.17	0.07	0.92	-0.31	-0.16	0.00	-0.16	0.06	0.96	-0.40	-0.19	-0.01	-0.19	0.07	0.97
100288911	LOC100288911	-0.38	-0.16	0.08	-0.16	0.07	0.91	-0.33	-0.17	-0.03	-0.17	0.07	0.93	-0.40	-0.18	-0.01	-0.19	0.07	0.97
100507436	MICA	-0.02	0.17	0.34	0.17	0.06	0.96	0.04	0.17	0.29	0.18	0.05	0.98	0.06	0.20	0.35	0.20	0.05	0.98

Abbreviations: rsFC, resting-state functional connectivity; L, left; A4ul, upper limb region of area 4; CNP, the Consortium for Neuropsychiatric Phenomics; SALD, the Southwest University Adult Lifespan Dataset; Min, minimum; Med, median; Max, maximum; SD, standard deviation.

Genes related to rsFC of the R-A4ul in both the discovery and validation experiments

GeneID	Genesymbol	Discovery dataset						CNP dataset						SALD dataset					
		r					Percentage	*r*					Percentage	*r*					Percentage
		Min	Med	Max	Mean	SD		Min	Med	Max	Mean	SD		Min	Med	Max	Mean	SD	
176	ACAN	-0.10	0.21	0.52	0.22	0.10	0.92	0.00	0.22	0.44	0.22	0.09	0.95	-0.06	0.26	0.49	0.26	0.10	0.96
286	ANK1	-0.13	0.19	0.53	0.19	0.10	0.91	-0.05	0.20	0.43	0.21	0.09	0.94	-0.11	0.24	0.45	0.23	0.10	0.93
367	AR	-0.06	0.17	0.43	0.18	0.08	0.91	-0.01	0.18	0.36	0.18	0.07	0.94	-0.08	0.21	0.40	0.20	0.08	0.95
445	ASS1	-0.38	-0.17	0.07	-0.17	0.08	0.90	-0.35	-0.18	-0.01	-0.18	0.07	0.90	-0.43	-0.21	0.05	-0.20	0.08	0.95
793	CALB1	-0.43	-0.18	0.05	-0.19	0.08	0.92	-0.38	-0.19	-0.04	-0.19	0.08	0.94	-0.40	-0.22	0.03	-0.22	0.09	0.94
814	CAMK4	-0.32	-0.17	0.07	-0.16	0.06	0.90	-0.31	-0.16	0.02	-0.16	0.06	0.95	-0.33	-0.19	0.02	-0.18	0.06	0.95
817	CAMK2D	-0.45	-0.19	0.13	-0.19	0.09	0.92	-0.39	-0.20	0.04	-0.21	0.08	0.95	-0.42	-0.24	0.04	-0.23	0.09	0.95
1006	CDH8	-0.41	-0.18	0.11	-0.19	0.08	0.91	-0.37	-0.20	0.00	-0.20	0.07	0.95	-0.41	-0.23	0.05	-0.22	0.08	0.96
2161	F12	-0.45	-0.17	0.14	-0.18	0.09	0.91	-0.38	-0.20	0.02	-0.20	0.08	0.95	-0.41	-0.22	0.08	-0.22	0.09	0.96
2322	FLT3	-0.10	0.16	0.39	0.16	0.07	0.90	-0.06	0.16	0.33	0.16	0.07	0.92	-0.06	0.18	0.33	0.18	0.07	0.92
2830	GPR6	-0.33	-0.16	0.06	-0.16	0.06	0.93	-0.34	-0.17	-0.01	-0.17	0.06	0.96	-0.34	-0.19	0.04	-0.18	0.07	0.93
2952	GSTT1	-0.07	0.18	0.46	0.19	0.09	0.92	0.01	0.19	0.40	0.20	0.08	0.94	-0.08	0.23	0.42	0.22	0.10	0.93
3624	INHBA	-0.07	0.15	0.31	0.14	0.06	0.91	-0.05	0.16	0.28	0.15	0.06	0.94	-0.04	0.18	0.32	0.18	0.06	0.94
4258	MGST2	-0.11	0.18	0.43	0.18	0.09	0.90	-0.06	0.20	0.38	0.19	0.08	0.94	-0.06	0.23	0.44	0.22	0.09	0.93
4286	MITF	-0.04	0.16	0.34	0.16	0.07	0.91	0.04	0.17	0.29	0.17	0.06	0.94	-0.03	0.19	0.35	0.19	0.07	0.95
4599	MX1	-0.18	0.17	0.42	0.17	0.08	0.91	-0.08	0.17	0.35	0.18	0.07	0.92	-0.02	0.22	0.39	0.21	0.08	0.94
4609	MYC	-0.05	0.17	0.37	0.17	0.08	0.91	-0.02	0.18	0.35	0.18	0.07	0.95	-0.04	0.20	0.34	0.19	0.08	0.93
4703	NEB	-0.07	0.20	0.43	0.19	0.09	0.92	0.01	0.21	0.39	0.21	0.08	0.95	-0.04	0.23	0.46	0.22	0.09	0.95
5176	SERPINF1	-0.35	-0.18	0.12	-0.17	0.07	0.92	-0.32	-0.18	0.07	-0.18	0.07	0.94	-0.36	-0.22	0.04	-0.21	0.07	0.96
5409	PNMT	-0.51	-0.18	0.15	-0.19	0.10	0.91	-0.43	-0.19	0.05	-0.19	0.09	0.92	-0.42	-0.23	0.06	-0.23	0.10	0.93
6330	SCN4B	-0.07	0.20	0.47	0.21	0.10	0.92	0.03	0.21	0.44	0.21	0.08	0.94	-0.05	0.25	0.46	0.24	0.10	0.95
6804	STX1A	-0.39	-0.18	0.06	-0.18	0.08	0.91	-0.35	-0.17	0.00	-0.17	0.07	0.94	-0.37	-0.21	0.07	-0.20	0.08	0.94
7068	THRB	-0.34	-0.15	0.07	-0.15	0.07	0.90	-0.27	-0.16	-0.03	-0.16	0.06	0.93	-0.35	-0.18	0.04	-0.18	0.07	0.94
7781	SLC30A3	-0.34	-0.16	0.11	-0.16	0.07	0.92	-0.30	-0.16	0.03	-0.16	0.06	0.94	-0.34	-0.19	0.03	-0.18	0.07	0.93
8938	BAIAP3	-0.56	-0.20	0.10	-0.21	0.11	0.90	-0.46	-0.21	-0.03	-0.22	0.10	0.93	-0.54	-0.26	0.09	-0.25	0.11	0.94
9033	PKD2L1	-0.38	-0.17	0.07	-0.17	0.08	0.91	-0.34	-0.18	0.00	-0.17	0.07	0.90	-0.40	-0.20	0.07	-0.19	0.08	0.92
10154	PLXNC1	-0.38	-0.16	0.06	-0.17	0.08	0.90	-0.36	-0.17	-0.01	-0.18	0.07	0.94	-0.36	-0.19	0.06	-0.19	0.08	0.92
10268	RAMP3	-0.11	0.15	0.33	0.15	0.07	0.91	-0.03	0.15	0.30	0.15	0.06	0.92	-0.03	0.18	0.34	0.17	0.07	0.93
22801	ITGA11	-0.07	0.18	0.39	0.18	0.08	0.90	0.01	0.19	0.35	0.19	0.07	0.93	-0.09	0.21	0.43	0.20	0.08	0.94
22987	SV2C	-0.09	0.18	0.50	0.18	0.09	0.90	0.01	0.19	0.43	0.20	0.08	0.93	-0.09	0.22	0.43	0.21	0.10	0.93
25841	ABTB2	-0.10	0.18	0.39	0.19	0.08	0.92	0.04	0.19	0.37	0.20	0.07	0.96	-0.02	0.24	0.43	0.23	0.08	0.97
25989	ULK3	-0.15	0.18	0.43	0.18	0.08	0.90	-0.01	0.19	0.38	0.19	0.08	0.94	-0.04	0.22	0.41	0.21	0.08	0.95
30850	CDR2L	-0.13	0.17	0.43	0.18	0.09	0.90	0.03	0.18	0.37	0.19	0.07	0.94	-0.06	0.22	0.42	0.22	0.09	0.94
51660	MPC1	-0.09	0.16	0.41	0.17	0.08	0.90	0.00	0.16	0.35	0.17	0.07	0.92	-0.07	0.19	0.36	0.19	0.08	0.91
54112	GPR88	-0.44	-0.16	0.09	-0.17	0.08	0.90	-0.37	-0.17	-0.01	-0.18	0.07	0.95	-0.38	-0.20	0.02	-0.20	0.08	0.93
55244	SLC47A1	-0.07	0.22	0.46	0.22	0.09	0.95	0.02	0.23	0.44	0.23	0.08	0.95	-0.04	0.25	0.44	0.25	0.09	0.96
55315	SLC29A3	-0.35	-0.16	0.09	-0.16	0.07	0.91	-0.30	-0.16	0.04	-0.16	0.06	0.94	-0.32	-0.20	-0.01	-0.19	0.06	0.94
55800	SCN3B	-0.52	-0.19	0.13	-0.19	0.10	0.91	-0.42	-0.20	0.03	-0.20	0.09	0.95	-0.44	-0.23	0.11	-0.23	0.10	0.95
55897	MESP1	-0.46	-0.18	0.17	-0.18	0.09	0.90	-0.37	-0.18	0.06	-0.19	0.08	0.94	-0.39	-0.21	0.05	-0.21	0.08	0.93
56937	PMEPA1	-0.12	0.17	0.39	0.17	0.08	0.90	-0.05	0.19	0.35	0.19	0.07	0.95	-0.03	0.22	0.38	0.21	0.08	0.94
57495	NWD2	-0.38	-0.18	0.08	-0.18	0.08	0.94	-0.35	-0.18	0.00	-0.18	0.07	0.95	-0.36	-0.21	0.04	-0.20	0.08	0.94
57496	MKL2	-0.39	-0.17	0.11	-0.17	0.08	0.90	-0.32	-0.16	0.03	-0.16	0.07	0.91	-0.37	-0.19	0.08	-0.19	0.08	0.92
57526	PCDH19	-0.44	-0.19	0.16	-0.19	0.09	0.93	-0.37	-0.19	0.05	-0.19	0.08	0.94	-0.41	-0.23	0.03	-0.22	0.08	0.95
79660	PPP1R3B	-0.04	0.14	0.31	0.15	0.06	0.91	0.01	0.15	0.27	0.14	0.05	0.92	-0.03	0.17	0.30	0.16	0.06	0.92
80020	FOXRED2	-0.37	-0.18	0.11	-0.18	0.08	0.93	-0.34	-0.18	0.04	-0.18	0.07	0.95	-0.37	-0.22	0.03	-0.21	0.08	0.94
80307	FER1L4	-0.13	0.19	0.48	0.20	0.10	0.92	-0.03	0.21	0.41	0.22	0.08	0.95	-0.04	0.25	0.44	0.24	0.09	0.95
81849	ST6GALNAC5	-0.43	-0.19	0.11	-0.19	0.09	0.91	-0.37	-0.21	-0.02	-0.20	0.08	0.94	-0.46	-0.24	0.05	-0.23	0.09	0.96
83660	TLN2	-0.37	-0.17	0.06	-0.17	0.07	0.91	-0.31	-0.17	0.02	-0.17	0.06	0.95	-0.35	-0.21	0.04	-0.20	0.07	0.94
84034	EMILIN2	-0.04	0.17	0.32	0.17	0.06	0.94	0.04	0.19	0.32	0.19	0.06	0.97	-0.04	0.21	0.35	0.20	0.07	0.96
127833	SYT2	-0.08	0.20	0.49	0.21	0.10	0.92	-0.01	0.21	0.45	0.22	0.09	0.94	-0.09	0.25	0.46	0.24	0.10	0.95
130399	ACVR1C	-0.13	0.17	0.41	0.17	0.08	0.90	-0.03	0.19	0.39	0.19	0.07	0.95	-0.06	0.21	0.38	0.20	0.08	0.94
144402	CPNE8	-0.31	-0.16	0.06	-0.16	0.07	0.91	-0.32	-0.16	-0.03	-0.16	0.06	0.90	-0.32	-0.17	0.07	-0.17	0.07	0.92
152940	C4orf45	-0.34	-0.15	0.14	-0.15	0.07	0.91	-0.29	-0.16	0.02	-0.16	0.06	0.90	-0.34	-0.19	0.03	-0.18	0.06	0.95
163782	KANK4	-0.05	0.17	0.33	0.17	0.07	0.93	0.03	0.18	0.30	0.18	0.06	0.96	-0.06	0.21	0.36	0.21	0.07	0.96
221294	NT5DC1	-0.10	0.16	0.32	0.16	0.07	0.93	0.04	0.17	0.30	0.17	0.06	0.94	-0.02	0.19	0.34	0.19	0.07	0.95
222537	HS3ST5	-0.10	0.17	0.40	0.17	0.08	0.91	0.02	0.19	0.36	0.19	0.07	0.93	-0.02	0.21	0.39	0.21	0.08	0.94
254102	EHBP1L1	-0.34	-0.18	0.07	-0.18	0.07	0.94	-0.34	-0.19	-0.01	-0.19	0.06	0.95	-0.35	-0.21	0.02	-0.21	0.06	0.97
286133	SCARA5	-0.50	-0.19	0.06	-0.19	0.10	0.91	-0.42	-0.21	-0.03	-0.21	0.09	0.94	-0.44	-0.24	0.09	-0.24	0.10	0.95
348013	TMEM255B	-0.08	0.17	0.38	0.17	0.08	0.91	-0.02	0.18	0.33	0.17	0.07	0.92	-0.04	0.20	0.37	0.20	0.08	0.94
404217	CTXN1	-0.44	-0.18	0.15	-0.19	0.09	0.90	-0.40	-0.19	0.06	-0.19	0.08	0.95	-0.43	-0.23	0.09	-0.23	0.09	0.95
642273	FAM110C	-0.51	-0.20	0.06	-0.21	0.10	0.92	-0.44	-0.21	-0.04	-0.22	0.09	0.94	-0.48	-0.25	0.07	-0.25	0.10	0.95

Abbreviations: rsFC, resting-state functional connectivity; R, right; A4ul, upper limb region of area 4; CNP, the Consortium for Neuropsychiatric Phenomics; SALD, the Southwest University Adult Lifespan Dataset; Min, minimum; Med, median; Max, maximum; SD, standard deviation.

Genes related to rsFC of the L-A4t in both the discovery and validation experiments

GeneID	Genesymbol	Discovery dataset						CNP dataset						SALD dataset					
		r					Percentage	*r*					Percentage	*r*					Percentage
		Min	Med	Max	Mean	SD		Min	Med	Max	Mean	SD		Min	Med	Max	Mean	SD	
784	CACNB3	-0.34	-0.16	0.02	-0.16	0.06	0.94	-0.30	-0.15	-0.03	-0.15	0.05	0.93	-0.31	-0.18	0.01	-0.17	0.06	0.95
814	CAMK4	-0.32	-0.17	0.06	-0.17	0.06	0.94	-0.30	-0.16	-0.01	-0.16	0.06	0.93	-0.34	-0.19	0.04	-0.18	0.06	0.95
2044	EPHA5	-0.29	-0.15	0.03	-0.15	0.05	0.91	-0.32	-0.14	-0.03	-0.14	0.05	0.93	-0.29	-0.17	0.04	-0.16	0.06	0.94
2830	GPR6	-0.34	-0.17	0.02	-0.17	0.07	0.90	-0.34	-0.19	-0.06	-0.18	0.06	0.96	-0.35	-0.19	0.06	-0.19	0.07	0.94
3679	ITGA7	-0.05	0.13	0.25	0.14	0.05	0.91	0.02	0.15	0.29	0.15	0.05	0.95	-0.05	0.17	0.28	0.16	0.05	0.97
5176	SERPINF1	-0.33	-0.17	0.09	-0.16	0.07	0.92	-0.33	-0.18	0.00	-0.18	0.07	0.92	-0.35	-0.20	0.04	-0.19	0.07	0.96
55244	SLC47A1	-0.04	0.21	0.44	0.21	0.09	0.93	0.06	0.22	0.44	0.22	0.08	0.93	-0.07	0.23	0.42	0.23	0.09	0.95
56967	C14orf132	-0.34	-0.16	0.01	-0.16	0.06	0.91	-0.29	-0.14	-0.03	-0.15	0.05	0.93	-0.33	-0.17	0.00	-0.17	0.06	0.95
90523	MLIP	-0.31	-0.16	0.04	-0.15	0.06	0.91	-0.27	-0.16	-0.01	-0.15	0.06	0.92	-0.34	-0.18	0.05	-0.18	0.06	0.96
163782	KANK4	-0.08	0.18	0.34	0.18	0.07	0.92	0.02	0.20	0.34	0.19	0.07	0.95	0.00	0.22	0.39	0.21	0.07	0.96
254102	EHBP1L1	-0.33	-0.18	0.06	-0.17	0.07	0.91	-0.30	-0.19	-0.03	-0.19	0.06	0.96	-0.35	-0.21	0.01	-0.20	0.06	0.96

Abbreviations: rsFC, resting-state functional connectivity; L, left; A4t, trunk region of area 4; CNP, the Consortium for Neuropsychiatric Phenomics; SALD, the Southwest University Adult Lifespan Dataset; Min, minimum; Med, median; Max, maximum; SD, standard deviation.

Genes related to rsFC of the R-A4t in both the discovery and validation experiments

GeneID	Genesymbol	Discovery dataset						CNP dataset						SALD dataset					
		r					Percentage	*r*					Percentage	*r*					Percentage
		Min	Med	Max	Mean	SD		Min	Med	Max	Mean	SD		Min	Med	Max	Mean	SD	
55244	SLC47A1	-0.06	0.20	0.44	0.20	0.09	0.92	0.04	0.21	0.45	0.22	0.09	0.94	0.00	0.22	0.45	0.23	0.09	0.95
254102	EHBP1L1	-0.34	-0.17	0.06	-0.17	0.07	0.90	-0.33	-0.17	-0.05	-0.18	0.06	0.94	-0.35	-0.20	-0.02	-0.20	0.06	0.95

Abbreviations: rsFC, resting-state functional connectivity; R, right; A4t, trunk region of area 4; CNP, the Consortium for Neuropsychiatric Phenomics; SALD, the Southwest University Adult Lifespan Dataset; Min, minimum; Med, median; Max, maximum; SD, standard deviation.

Genes related to rsFC of the L-A4tl in both the discovery and validation experiments

GeneID	Genesymbol	Discovery dataset						CNP dataset						SALD dataset					
		r					Percentage	*r*					Percentage	*r*					Percentage
		Min	Med	Max	Mean	SD		Min	Med	Max	Mean	SD		Min	Med	Max	Mean	SD	
1300	COL10A1	-0.27	-0.17	0.03	-0.16	0.05	0.94	-0.27	-0.16	-0.06	-0.16	0.05	0.95	-0.27	-0.16	0.02	-0.16	0.05	0.94
2634	GBP2	-0.02	0.15	0.26	0.15	0.04	0.93	0.04	0.15	0.22	0.14	0.04	0.94	0.02	0.15	0.24	0.15	0.04	0.94
5587	PRKD1	-0.25	-0.14	-0.01	-0.14	0.04	0.93	-0.23	-0.13	0.01	-0.12	0.04	0.91	-0.23	-0.13	0.02	-0.13	0.04	0.90
9607	CARTPT	-0.43	-0.25	0.02	-0.25	0.08	0.98	-0.38	-0.23	0.03	-0.23	0.08	0.96	-0.45	-0.25	0.00	-0.25	0.08	0.99
118429	ANTXR2	-0.04	0.16	0.28	0.16	0.05	0.94	0.01	0.16	0.28	0.16	0.05	0.93	0.03	0.16	0.27	0.16	0.05	0.94

Abbreviations: rsFC, resting-state functional connectivity; L, left; A4tl, tongue and larynx region of area 4; CNP, the Consortium for Neuropsychiatric Phenomics; SALD, the Southwest University Adult Lifespan Dataset; Min, minimum; Med, median; Max, maximum; SD, standard deviation.

Genes related to rsFC of the R-A4tl in both the discovery and validation experiments

GeneID	Genesymbol	Discovery dataset						CNP dataset						SALD dataset					
		r					Percentage	*r*					Percentage	*r*					Percentage
		Min	Med	Max	Mean	SD		Min	Med	Max	Mean	SD		Min	Med	Max	Mean	SD	
9607	CARTPT	-0.41	-0.24	0.14	-0.23	0.08	0.97	-0.36	-0.21	0.03	-0.21	0.08	0.94	-0.45	-0.23	0.08	-0.23	0.08	0.98

Abbreviations: rsFC, resting-state functional connectivity; R, right; A4tl, tongue and larynx region of area 4; CNP, the Consortium for Neuropsychiatric Phenomics; SALD, the Southwest University Adult Lifespan Dataset; Min, minimum; Med, median; Max, maximum; SD, standard deviation.

Genes related to rsFC of the L-A1/2/3ll in both the discovery and validation experiments

GeneID	Genesymbol	Discovery dataset						CNP dataset						SALD dataset					
		r					Percentage	*r*					Percentage	*r*					Percentage
		Min	Med	Max	Mean	SD		Min	Med	Max	Mean	SD		Min	Med	Max	Mean	SD	
814	CAMK4	-0.33	-0.16	0.10	-0.16	0.06	0.91	-0.28	-0.16	0.03	-0.16	0.06	0.90	-0.33	-0.17	0.00	-0.16	0.06	0.93
1300	COL10A1	-0.32	-0.15	0.07	-0.15	0.06	0.90	-0.27	-0.17	0.00	-0.16	0.06	0.93	-0.30	-0.17	0.01	-0.17	0.05	0.95

Abbreviations: rsFC, resting-state functional connectivity; L, left; A1/2/3ll, lower limb region of area1/2/3; CNP, the Consortium for Neuropsychiatric Phenomics; SALD, the Southwest University Adult Lifespan Dataset; Min, minimum; Med, median; Max, maximum; SD, standard deviation.

Genes related to rsFC of the R-A1/2/3ll in both the discovery and validation experiments																				
GeneID	Genesymbol	Discovery dataset						CNP dataset						SALD dataset						
		r					Percentage	*r*					Percentage	*r*					Percentage
		Min	Med	Max	Mean	SD		Min	Med	Max	Mean	SD		Min	Med	Max	Mean	SD	
1300	COL10A1	-0.30	-0.17	0.00	-0.17	0.05	0.95	-0.29	-0.17	0.02	-0.16	0.06	0.92	-0.32	-0.18	-0.04	-0.17	0.05	0.97

Abbreviations: rsFC, resting-state functional connectivity; R, right; A1/2/3ll, lower limb region of area1/2/3; CNP, the Consortium for Neuropsychiatric Phenomics; SALD, the Southwest University Adult Lifespan Dataset; Min, minimum; Med, median; Max, maximum; SD, standard deviation.

Genes related to rsFC of the L-A4ll in both the discovery and validation experiments

GeneID	Genesymbol	Discovery dataset						CNP dataset						SALD dataset					
		r					Percentage	*r*					Percentage	*r*					Percentage
		Min	Med	Max	Mean	SD		Min	Med	Max	Mean	SD		Min	Med	Max	Mean	SD	
176	ACAN	-0.08	0.20	0.52	0.20	0.10	0.91	0.01	0.20	0.42	0.21	0.09	0.93	-0.01	0.21	0.47	0.23	0.10	0.95
341	APOC1	-0.35	-0.16	0.09	-0.16	0.06	0.93	-0.30	-0.16	-0.01	-0.16	0.06	0.92	-0.36	-0.17	0.01	-0.17	0.07	0.93
784	CACNB3	-0.34	-0.18	-0.02	-0.18	0.05	0.97	-0.28	-0.16	-0.01	-0.16	0.06	0.90	-0.31	-0.18	-0.03	-0.18	0.05	0.97
793	CALB1	-0.45	-0.18	0.08	-0.19	0.08	0.93	-0.36	-0.19	-0.03	-0.18	0.08	0.91	-0.39	-0.20	0.05	-0.20	0.09	0.94
814	CAMK4	-0.35	-0.19	-0.02	-0.19	0.06	0.98	-0.30	-0.16	-0.02	-0.16	0.06	0.91	-0.34	-0.19	0.01	-0.19	0.06	0.97
817	CAMK2D	-0.44	-0.19	0.10	-0.19	0.08	0.93	-0.39	-0.19	-0.01	-0.19	0.08	0.93	-0.41	-0.20	0.03	-0.21	0.09	0.95
2044	EPHA5	-0.32	-0.16	0.05	-0.16	0.05	0.96	-0.31	-0.15	-0.04	-0.15	0.06	0.90	-0.29	-0.17	-0.01	-0.17	0.05	0.96
2830	GPR6	-0.35	-0.17	0.03	-0.17	0.06	0.96	-0.33	-0.18	-0.02	-0.18	0.07	0.95	-0.35	-0.18	0.01	-0.18	0.07	0.95
3679	ITGA7	0.02	0.15	0.27	0.15	0.05	0.94	0.00	0.15	0.27	0.15	0.05	0.93	0.04	0.17	0.28	0.17	0.05	0.98
4703	NEB	-0.07	0.19	0.39	0.18	0.08	0.91	-0.03	0.18	0.38	0.19	0.09	0.91	-0.03	0.18	0.42	0.20	0.09	0.93
5176	SERPINF1	-0.38	-0.19	-0.02	-0.19	0.06	0.96	-0.30	-0.18	0.02	-0.17	0.07	0.90	-0.36	-0.21	-0.03	-0.21	0.07	0.97
5794	PTPRH	-0.06	0.13	0.23	0.13	0.05	0.91	0.01	0.14	0.25	0.14	0.05	0.91	0.01	0.15	0.26	0.15	0.05	0.94
5961	PRPH2	-0.34	-0.15	0.05	-0.15	0.06	0.91	-0.32	-0.15	0.06	-0.15	0.06	0.93	-0.33	-0.17	0.04	-0.17	0.07	0.93
6330	SCN4B	-0.08	0.20	0.48	0.20	0.09	0.93	0.00	0.22	0.44	0.21	0.09	0.94	-0.03	0.22	0.43	0.22	0.10	0.95
9473	THEMIS2	-0.08	0.15	0.30	0.15	0.06	0.92	0.01	0.15	0.27	0.15	0.05	0.92	0.01	0.17	0.31	0.17	0.06	0.94
10268	RAMP3	-0.05	0.17	0.34	0.17	0.06	0.93	0.03	0.16	0.30	0.17	0.06	0.93	0.02	0.18	0.35	0.18	0.07	0.95
22987	SV2C	-0.12	0.19	0.53	0.19	0.09	0.90	-0.02	0.18	0.44	0.19	0.09	0.93	-0.03	0.19	0.39	0.20	0.09	0.91
50486	G0S2	-0.04	0.17	0.32	0.17	0.06	0.95	0.04	0.17	0.34	0.17	0.06	0.96	0.02	0.19	0.34	0.19	0.06	0.96
55244	SLC47A1	-0.05	0.23	0.47	0.23	0.08	0.96	-0.02	0.23	0.45	0.23	0.09	0.98	0.00	0.22	0.44	0.23	0.09	0.97
55315	SLC29A3	-0.32	-0.17	0.06	-0.17	0.06	0.94	-0.30	-0.16	0.03	-0.16	0.07	0.90	-0.32	-0.18	-0.03	-0.18	0.06	0.96
57526	PCDH19	-0.42	-0.18	0.05	-0.18	0.08	0.93	-0.35	-0.17	0.01	-0.18	0.08	0.91	-0.38	-0.20	0.02	-0.20	0.08	0.94
80307	FER1L4	-0.09	0.20	0.48	0.20	0.09	0.92	0.01	0.21	0.42	0.20	0.09	0.93	0.01	0.21	0.44	0.22	0.09	0.94
84034	EMILIN2	0.00	0.19	0.34	0.19	0.06	0.98	0.01	0.20	0.31	0.19	0.07	0.94	0.03	0.20	0.37	0.21	0.07	0.97
84937	ZNRF1	-0.32	-0.14	0.04	-0.14	0.06	0.90	-0.28	-0.15	-0.01	-0.15	0.06	0.94	-0.29	-0.16	-0.01	-0.17	0.06	0.94
114571	SLC22A9	-0.38	-0.16	0.02	-0.16	0.06	0.94	-0.27	-0.16	0.02	-0.15	0.06	0.91	-0.34	-0.17	0.01	-0.17	0.06	0.95
127833	SYT2	-0.10	0.19	0.50	0.20	0.10	0.91	0.01	0.21	0.45	0.20	0.09	0.93	-0.02	0.21	0.45	0.22	0.10	0.94
134548	SOWAHA	-0.38	-0.17	0.07	-0.17	0.07	0.91	-0.35	-0.16	0.01	-0.17	0.07	0.91	-0.39	-0.18	0.03	-0.18	0.08	0.93
148014	TTC9B	-0.25	-0.13	0.05	-0.13	0.05	0.92	-0.26	-0.13	-0.03	-0.13	0.05	0.91	-0.26	-0.15	0.00	-0.15	0.05	0.96
163782	KANK4	0.00	0.19	0.35	0.20	0.07	0.98	-0.03	0.20	0.32	0.19	0.07	0.91	0.01	0.22	0.40	0.22	0.07	0.97
221294	NT5DC1	-0.02	0.16	0.33	0.16	0.06	0.91	-0.01	0.17	0.29	0.16	0.06	0.92	0.03	0.18	0.34	0.18	0.07	0.95
222537	HS3ST5	-0.07	0.16	0.38	0.17	0.08	0.90	-0.02	0.18	0.33	0.19	0.07	0.93	0.00	0.18	0.38	0.19	0.08	0.94
254102	EHBP1L1	-0.34	-0.20	0.01	-0.20	0.06	0.98	-0.31	-0.18	-0.01	-0.19	0.06	0.95	-0.37	-0.21	-0.06	-0.21	0.06	0.99
286133	SCARA5	-0.53	-0.18	0.10	-0.18	0.10	0.90	-0.39	-0.20	0.04	-0.20	0.09	0.90	-0.44	-0.20	0.06	-0.21	0.10	0.92

Abbreviations: rsFC, resting-state functional connectivity; L, left; A4ll, lower limb region of area 4; CNP, the Consortium for Neuropsychiatric Phenomics; SALD, the Southwest University Adult Lifespan Dataset; Min, minimum; Med, median; Max, maximum; SD, standard deviation.

Genes related to rsFC of the R-A4ll in both the discovery and validation experiments

GeneID	Genesymbol	Discovery dataset						CNP dataset						SALD dataset					
		r					Percentage	*r*					Percentage	*r*					Percentage
		Min	Med	Max	Mean	SD		Min	Med	Max	Mean	SD		Min	Med	Max	Mean	SD	
784	CACNB3	-0.34	-0.17	0.08	-0.17	0.05	0.96	-0.29	-0.14	-0.03	-0.15	0.06	0.90	-0.31	-0.18	0.02	-0.17	0.06	0.96
814	CAMK4	-0.32	-0.18	0.09	-0.18	0.06	0.97	-0.31	-0.15	0.02	-0.15	0.06	0.90	-0.34	-0.19	0.04	-0.19	0.06	0.97
2044	EPHA5	-0.28	-0.15	0.09	-0.15	0.05	0.96	-0.31	-0.14	-0.03	-0.14	0.05	0.91	-0.29	-0.17	0.03	-0.16	0.06	0.95
2830	GPR6	-0.34	-0.16	0.10	-0.16	0.06	0.94	-0.34	-0.17	-0.03	-0.17	0.06	0.95	-0.35	-0.17	0.10	-0.17	0.07	0.91
3679	ITGA7	0.00	0.14	0.26	0.14	0.05	0.94	0.02	0.14	0.24	0.14	0.05	0.94	0.02	0.17	0.29	0.17	0.05	0.97
5176	SERPINF1	-0.35	-0.19	0.04	-0.18	0.07	0.95	-0.29	-0.18	0.03	-0.17	0.07	0.91	-0.36	-0.20	0.04	-0.21	0.07	0.97
6330	SCN4B	-0.17	0.18	0.48	0.19	0.09	0.91	0.03	0.20	0.45	0.20	0.09	0.92	-0.04	0.21	0.41	0.21	0.10	0.91
10268	RAMP3	-0.11	0.15	0.32	0.16	0.06	0.91	0.02	0.15	0.31	0.16	0.06	0.90	-0.05	0.17	0.34	0.17	0.07	0.91
50486	G0S2	-0.07	0.16	0.30	0.16	0.06	0.94	0.03	0.16	0.34	0.16	0.06	0.97	-0.07	0.18	0.33	0.18	0.06	0.94
55244	SLC47A1	-0.09	0.21	0.46	0.22	0.08	0.96	0.05	0.22	0.45	0.22	0.08	0.93	-0.04	0.22	0.43	0.22	0.09	0.95
79660	PPP1R3B	-0.11	0.14	0.31	0.14	0.05	0.90	0.03	0.14	0.27	0.14	0.05	0.90	-0.01	0.15	0.30	0.15	0.06	0.91
84034	EMILIN2	-0.11	0.18	0.34	0.18	0.06	0.96	0.04	0.19	0.33	0.18	0.07	0.92	-0.02	0.20	0.35	0.20	0.07	0.96
114990	VASN	-0.27	-0.15	0.06	-0.14	0.05	0.90	-0.28	-0.14	0.01	-0.13	0.06	0.90	-0.29	-0.15	0.03	-0.15	0.05	0.92
254102	EHBP1L1	-0.33	-0.19	0.09	-0.19	0.06	0.96	-0.32	-0.18	-0.04	-0.18	0.06	0.94	-0.37	-0.21	-0.02	-0.20	0.07	0.98

Abbreviations: rsFC, resting-state functional connectivity; R, right; A4ll, lower limb region of area 4; CNP, the Consortium for Neuropsychiatric Phenomics; SALD, the Southwest University Adult Lifespan Dataset; Min, minimum; Med, median; Max, maximum; SD, standard deviation.

Genes related to rsFC of the L-A1/2/3ulhf in both the discovery and validation experiments

GeneID	Genesymbol	Discovery dataset *r* Min	Med	Max	Mean	SD	Percentage	CNP dataset *r* Min	Med	Max	Mean	SD	Percentage	SALD dataset *r* Min	Med	Max	Mean	SD	Percentage
92	ACVR2A	-0.32	-0.15	0.03	-0.16	0.05	0.95	-0.32	-0.17	-0.08	-0.17	0.05	1.00	-0.30	-0.17	-0.01	-0.17	0.05	0.98
98	ACYP2	-0.12	0.16	0.35	0.16	0.06	0.93	0.04	0.14	0.32	0.16	0.06	0.97	-0.03	0.16	0.32	0.16	0.07	0.91
115	ADCY9	-0.13	0.16	0.38	0.16	0.08	0.92	0.03	0.17	0.34	0.17	0.07	0.94	-0.03	0.17	0.35	0.18	0.08	0.92
133	ADM	-0.12	0.18	0.41	0.18	0.07	0.94	0.04	0.17	0.32	0.17	0.06	0.95	0.00	0.17	0.36	0.18	0.08	0.93
161	AP2A2	-0.36	-0.15	0.07	-0.15	0.06	0.92	-0.28	-0.14	-0.04	-0.14	0.05	0.94	-0.28	-0.15	-0.02	-0.16	0.06	0.94
176	ACAN	-0.15	0.24	0.51	0.25	0.09	0.98	0.09	0.25	0.44	0.26	0.08	1.00	0.03	0.27	0.47	0.27	0.09	0.99
204	AK2	-0.05	0.15	0.30	0.15	0.05	0.94	0.04	0.15	0.26	0.15	0.05	0.95	0.02	0.17	0.28	0.17	0.05	0.98
249	ALPL	-0.28	-0.14	0.06	-0.14	0.05	0.95	-0.27	-0.15	-0.04	-0.15	0.05	0.97	-0.26	-0.15	-0.02	-0.15	0.05	0.93
272	AMPD3	-0.03	0.11	0.21	0.11	0.04	0.91	0.04	0.13	0.21	0.13	0.04	0.93	0.02	0.13	0.23	0.13	0.04	0.95
286	ANK1	-0.13	0.22	0.51	0.22	0.09	0.96	0.04	0.23	0.44	0.23	0.08	0.98	0.02	0.24	0.42	0.24	0.09	0.98
292	SLC25A5	-0.09	0.14	0.31	0.15	0.06	0.93	0.05	0.14	0.31	0.15	0.05	0.99	-0.02	0.15	0.30	0.15	0.06	0.91
308	ANXA5	-0.02	0.14	0.23	0.14	0.04	0.95	0.04	0.14	0.22	0.15	0.04	0.99	0.04	0.15	0.25	0.15	0.04	0.98
320	APBA1	-0.23	-0.14	0.02	-0.14	0.04	0.94	-0.27	-0.16	-0.07	-0.16	0.04	1.00	-0.24	-0.15	-0.02	-0.15	0.04	0.97
366	AQP9	-0.31	-0.14	0.11	-0.14	0.07	0.90	-0.26	-0.15	0.02	-0.15	0.06	0.91	-0.34	-0.16	0.08	-0.16	0.07	0.90
367	AR	-0.07	0.21	0.46	0.21	0.07	0.98	0.08	0.21	0.40	0.22	0.06	1.00	0.05	0.23	0.39	0.23	0.07	1.00
396	ARHGDIA	-0.41	-0.16	0.09	-0.16	0.08	0.91	-0.35	-0.16	-0.01	-0.16	0.07	0.93	-0.35	-0.18	0.00	-0.18	0.08	0.91
430	ASCL2	-0.46	-0.21	0.13	-0.21	0.09	0.96	-0.42	-0.22	-0.05	-0.23	0.08	0.99	-0.40	-0.22	-0.03	-0.23	0.09	0.97
445	ASS1	-0.39	-0.20	0.08	-0.20	0.07	0.96	-0.36	-0.21	-0.05	-0.22	0.06	0.98	-0.41	-0.22	0.00	-0.22	0.07	0.98
460	ASTN1	-0.35	-0.16	0.08	-0.16	0.06	0.94	-0.32	-0.18	-0.06	-0.17	0.05	0.98	-0.34	-0.18	-0.01	-0.18	0.06	0.98
481	ATP1B1	-0.12	0.16	0.37	0.16	0.07	0.94	0.02	0.17	0.31	0.18	0.06	0.97	0.01	0.17	0.33	0.17	0.07	0.94
492	ATP2B3	-0.09	0.16	0.38	0.16	0.07	0.93	0.04	0.17	0.32	0.17	0.06	0.98	-0.01	0.17	0.34	0.17	0.07	0.93
493	ATP2B4	-0.49	-0.20	0.12	-0.20	0.09	0.94	-0.40	-0.19	0.00	-0.20	0.08	0.96	-0.42	-0.21	0.01	-0.22	0.09	0.97
528	ATP6V1C1	-0.34	-0.16	0.06	-0.15	0.06	0.91	-0.30	-0.15	-0.03	-0.15	0.06	0.95	-0.32	-0.16	-0.02	-0.17	0.06	0.96
563	AZGP1	-0.04	0.15	0.28	0.15	0.05	0.94	0.04	0.16	0.25	0.16	0.04	0.98	0.04	0.16	0.29	0.16	0.05	0.96
586	BCAT1	-0.12	0.16	0.33	0.16	0.06	0.94	0.05	0.16	0.31	0.16	0.05	0.98	-0.02	0.16	0.32	0.16	0.06	0.94
613	BCR	-0.37	-0.15	0.09	-0.15	0.07	0.91	-0.32	-0.16	-0.05	-0.17	0.05	0.97	-0.32	-0.17	-0.03	-0.17	0.07	0.94
715	C1R	-0.37	-0.15	0.13	-0.15	0.07	0.90	-0.33	-0.15	0.02	-0.16	0.06	0.94	-0.34	-0.18	0.00	-0.18	0.07	0.95
784	CACNB3	-0.31	-0.15	0.00	-0.15	0.05	0.94	-0.27	-0.16	-0.04	-0.16	0.05	0.98	-0.29	-0.15	0.04	-0.15	0.05	0.95
793	CALB1	-0.42	-0.21	0.10	-0.21	0.07	0.98	-0.36	-0.21	-0.03	-0.22	0.07	0.99	-0.42	-0.22	0.00	-0.22	0.08	0.98
808	CALM3	-0.43	-0.17	0.08	-0.17	0.08	0.90	-0.36	-0.19	-0.03	-0.19	0.07	0.96	-0.36	-0.19	0.01	-0.19	0.08	0.94
814	CAMK4	-0.32	-0.18	0.09	-0.18	0.06	0.97	-0.29	-0.18	-0.03	-0.18	0.05	0.99	-0.33	-0.18	-0.01	-0.18	0.05	0.97
817	CAMK2D	-0.46	-0.22	0.11	-0.22	0.08	0.98	-0.41	-0.23	-0.06	-0.23	0.07	0.99	-0.42	-0.24	-0.07	-0.24	0.08	1.00
818	CAMK2G	-0.11	0.19	0.42	0.19	0.08	0.94	0.03	0.20	0.35	0.19	0.07	0.96	0.00	0.20	0.37	0.20	0.08	0.97
862	RUNX1T1	-0.26	-0.12	0.04	-0.12	0.04	0.93	-0.26	-0.12	-0.03	-0.12	0.04	0.92	-0.26	-0.14	0.00	-0.14	0.05	0.94
869	CBLN1	-0.31	-0.14	0.07	-0.14	0.06	0.93	-0.24	-0.14	-0.02	-0.14	0.05	0.92	-0.29	-0.16	-0.02	-0.15	0.06	0.91
955	ENTPD6	-0.26	-0.13	0.02	-0.12	0.05	0.90	-0.22	-0.12	0.00	-0.12	0.04	0.96	-0.23	-0.14	-0.02	-0.14	0.04	0.93
1002	CDH4	-0.35	-0.16	0.10	-0.16	0.07	0.93	-0.31	-0.17	-0.04	-0.17	0.05	0.97	-0.32	-0.17	-0.01	-0.17	0.07	0.95
1005	CDH7	-0.10	0.15	0.37	0.16	0.07	0.93	0.03	0.16	0.32	0.17	0.06	0.97	-0.01	0.17	0.35	0.17	0.07	0.90
1006	CDH8	-0.40	-0.21	0.10	-0.22	0.07	0.97	-0.37	-0.23	-0.07	-0.23	0.06	1.00	-0.39	-0.23	-0.05	-0.23	0.07	0.99
1007	CDH9	-0.42	-0.17	0.10	-0.17	0.07	0.92	-0.29	-0.16	-0.03	-0.16	0.06	0.94	-0.37	-0.19	0.00	-0.19	0.07	0.96
1027	CDKN1B	-0.06	0.15	0.33	0.15	0.06	0.93	0.06	0.14	0.30	0.15	0.05	0.98	0.03	0.16	0.29	0.16	0.06	0.94
1031	CDKN2C	-0.07	0.14	0.34	0.15	0.06	0.93	0.04	0.14	0.25	0.14	0.05	0.93	0.02	0.16	0.28	0.16	0.06	0.94
1032	CDKN2D	-0.35	-0.16	0.06	-0.16	0.07	0.93	-0.28	-0.16	-0.04	-0.16	0.05	0.97	-0.33	-0.18	-0.03	-0.18	0.07	0.96
1040	CDS1	-0.14	0.17	0.42	0.17	0.08	0.92	0.02	0.17	0.35	0.18	0.07	0.97	-0.02	0.18	0.36	0.18	0.08	0.93
1050	CEBPA	-0.41	-0.18	0.10	-0.18	0.08	0.94	-0.38	-0.20	-0.04	-0.20	0.07	0.98	-0.37	-0.20	0.00	-0.21	0.08	0.98
1073	CFL2	-0.07	0.15	0.30	0.14	0.05	0.93	0.05	0.15	0.28	0.15	0.05	0.96	0.03	0.15	0.29	0.16	0.06	0.96
1176	AP3S1	-0.37	-0.16	0.07	-0.16	0.07	0.90	-0.32	-0.17	-0.05	-0.17	0.06	0.97	-0.36	-0.18	-0.03	-0.18	0.07	0.95
1272	CNTN1	-0.40	-0.18	0.07	-0.18	0.07	0.93	-0.35	-0.18	-0.03	-0.18	0.07	0.98	-0.38	-0.19	-0.02	-0.19	0.07	0.96
1294	COL7A1	-0.06	0.16	0.36	0.16	0.06	0.92	0.05	0.17	0.30	0.17	0.06	0.96	0.00	0.17	0.30	0.17	0.07	0.95
1300	COL10A1	-0.28	-0.15	0.08	-0.15	0.06	0.93	-0.27	-0.18	-0.04	-0.17	0.04	0.98	-0.25	-0.16	0.01	-0.15	0.05	0.95
1381	CRABP1	-0.14	0.20	0.45	0.20	0.08	0.98	0.02	0.19	0.35	0.20	0.07	0.98	-0.01	0.21	0.41	0.20	0.08	0.97
1501	CTNND2	-0.38	-0.18	0.09	-0.18	0.07	0.95	-0.31	-0.18	-0.07	-0.18	0.05	1.00	-0.37	-0.19	-0.01	-0.19	0.07	0.97
1522	CTSZ	-0.13	0.18	0.38	0.18	0.07	0.96	0.03	0.16	0.34	0.18	0.06	0.98	-0.04	0.19	0.38	0.20	0.07	0.96
1523	CUX1	-0.08	0.14	0.33	0.15	0.06	0.92	0.01	0.15	0.27	0.15	0.06	0.93	-0.05	0.16	0.32	0.16	0.07	0.93
1607	DGKB	-0.39	-0.19	0.11	-0.19	0.07	0.96	-0.32	-0.18	-0.05	-0.18	0.06	0.96	-0.38	-0.20	-0.01	-0.20	0.07	0.97
1730	DIAPH2	-0.32	-0.16	0.05	-0.16	0.06	0.94	-0.27	-0.16	-0.07	-0.16	0.05	0.99	-0.30	-0.17	-0.03	-0.17	0.05	0.97
1755	DMBT1	-0.02	0.12	0.19	0.11	0.04	0.90	0.04	0.13	0.20	0.13	0.04	0.98	0.02	0.13	0.23	0.13	0.04	0.96
1768	DNAH6	-0.35	-0.14	0.06	-0.14	0.06	0.91	-0.26	-0.15	-0.03	-0.15	0.05	0.97	-0.30	-0.16	-0.01	-0.16	0.06	0.95
1809	DPYSL3	-0.51	-0.19	0.12	-0.20	0.09	0.92	-0.40	-0.20	-0.04	-0.20	0.08	0.96	-0.41	-0.21	0.00	-0.21	0.09	0.95
1871	E2F3	-0.32	-0.13	0.08	-0.14	0.06	0.90	-0.26	-0.15	-0.02	-0.15	0.06	0.93	-0.31	-0.16	0.03	-0.15	0.07	0.91
1875	E2F5	-0.36	-0.14	0.07	-0.14	0.06	0.92	-0.28	-0.16	-0.04	-0.16	0.05	0.96	-0.30	-0.16	-0.01	-0.16	0.06	0.94
1893	ECM1	-0.13	0.18	0.42	0.19	0.08	0.95	0.04	0.21	0.38	0.20	0.06	0.98	0.01	0.19	0.36	0.20	0.07	0.95
1909	EDNRA	-0.10	0.14	0.32	0.14	0.06	0.91	0.01	0.15	0.28	0.15	0.05	0.94	0.01	0.15	0.30	0.15	0.06	0.91
1948	EFNB2	-0.38	-0.17	0.11	-0.17	0.07	0.94	-0.33	-0.17	-0.06	-0.18	0.06	0.96	-0.39	-0.19	0.01	-0.19	0.08	0.94
1949	EFNB3	-0.48	-0.18	0.10	-0.18	0.08	0.92	-0.38	-0.18	-0.04	-0.19	0.08	0.95	-0.43	-0.21	0.00	-0.21	0.08	0.96
1960	EGR3	-0.24	-0.13	0.04	-0.12	0.04	0.90	-0.20	-0.13	-0.03	-0.13	0.04	0.94	-0.25	-0.13	-0.01	-0.13	0.05	0.91
2027	ENO3	-0.02	0.12	0.23	0.12	0.04	0.92	0.04	0.13	0.21	0.13	0.04	0.97	0.00	0.13	0.25	0.13	0.04	0.94
2044	EPHA5	-0.33	-0.15	0.02	-0.15	0.05	0.97	-0.29	-0.16	-0.06	-0.16	0.04	0.99	-0.28	-0.16	-0.01	-0.16	0.05	0.96
2070	EYA4	-0.10	0.19	0.43	0.19	0.07	0.95	0.05	0.19	0.38	0.20	0.07	0.98	0.04	0.20	0.37	0.20	0.08	0.97
2101	ESRRA	-0.13	0.21	0.47	0.21	0.08	0.96	0.04	0.22	0.41	0.22	0.07	0.99	0.02	0.22	0.41	0.23	0.08	0.98
2104	ESRRG	-0.16	0.23	0.51	0.24	0.09	0.97	0.06	0.24	0.45	0.25	0.08	0.99	0.01	0.24	0.44	0.25	0.09	0.99
2161	F12	-0.49	-0.21	0.08	-0.21	0.08	0.97	-0.39	-0.22	-0.08	-0.23	0.06	1.00	-0.40	-0.23	-0.04	-0.24	0.08	0.99
2170	FABP3	-0.31	-0.15	0.04	-0.15	0.05	0.93	-0.30	-0.16	-0.06	-0.17	0.05	0.99	-0.29	-0.17	-0.01	-0.17	0.06	0.95
2173	FABP7	-0.49	-0.20	0.11	-0.20	0.09	0.95	-0.37	-0.21	-0.06	-0.21	0.07	0.99	-0.40	-0.21	-0.02	-0.22	0.09	0.96
2182	ACSL4	-0.36	-0.15	0.08	-0.16	0.07	0.92	-0.28	-0.15	-0.03	-0.15	0.06	0.92	-0.33	-0.17	0.00	-0.17	0.07	0.93
2254	FGF9	-0.12	0.19	0.46	0.19	0.09	0.93	0.05	0.20	0.40	0.20	0.07	0.98	0.00	0.20	0.38	0.21	0.08	0.95
2322	FLT3	-0.11	0.17	0.36	0.17	0.07	0.95	0.02	0.17	0.34	0.18	0.06	0.98	0.00	0.18	0.34	0.18	0.06	0.97
2558	GABRA5	-0.50	-0.19	0.10	-0.19	0.09	0.91	-0.40	-0.19	-0.02	-0.19	0.09	0.94	-0.45	-0.21	0.01	-0.21	0.09	0.95
2560	GABRB1	-0.50	-0.21	0.12	-0.21	0.08	0.96	-0.38	-0.21	-0.06	-0.21	0.07	0.97	-0.40	-0.22	-0.04	-0.23	0.08	0.98
2562	GABRB3	-0.33	-0.15	0.04	-0.15	0.06	0.91	-0.26	-0.13	-0.04	-0.14	0.05	0.91	-0.30	-0.16	-0.04	-0.16	0.06	0.95
2565	GABRG1	-0.34	-0.16	0.12	-0.16	0.06	0.94	-0.26	-0.17	-0.04	-0.17	0.05	0.96	-0.33	-0.17	0.00	-0.17	0.06	0.95
2620	GAS2	-0.12	0.17	0.41	0.17	0.07	0.94	0.06	0.17	0.33	0.17	0.06	0.97	0.01	0.18	0.35	0.18	0.07	0.94
2634	GBP2	-0.05	0.14	0.25	0.13	0.05	0.93	0.03	0.15	0.23	0.15	0.04	0.98	-0.02	0.14	0.24	0.14	0.04	0.94
2690	GHR	-0.32	-0.14	0.06	-0.14	0.06	0.92	-0.28	-0.15	-0.06	-0.15	0.05	0.98	-0.31	-0.16	-0.01	-0.16	0.06	0.96
2742	GLRA2	-0.46	-0.20	0.09	-0.20	0.08	0.94	-0.39	-0.20	-0.04	-0.21	0.07	0.98	-0.41	-0.22	-0.02	-0.23	0.08	0.98
2764	GMFB	-0.40	-0.17	0.08	-0.17	0.08	0.90	-0.36	-0.18	-0.01	-0.18	0.07	0.97	-0.37	-0.19	0.01	-0.19	0.07	0.95
2766	GMPR	-0.10	0.17	0.35	0.17	0.06	0.96	0.04	0.16	0.28	0.16	0.05	0.99	0.01	0.17	0.31	0.17	0.06	0.96
2778	GNAS	-0.09	0.15	0.31	0.15	0.06	0.94	0.04	0.15	0.29	0.16	0.05	0.96	0.02	0.16	0.29	0.16	0.06	0.92
2823	GPM6A	-0.36	-0.17	0.08	-0.18	0.06	0.98	-0.29	-0.17	-0.06	-0.17	0.05	0.99	-0.33	-0.19	-0.02	-0.19	0.06	0.98
2830	GPR6	-0.35	-0.19	0.02	-0.19	0.06	0.97	-0.34	-0.20	-0.10	-0.20	0.05	1.00	-0.35	-0.21	-0.03	-0.21	0.06	0.99
2888	GRB14	-0.34	-0.18	0.08	-0.18	0.07	0.96	-0.32	-0.20	-0.04	-0.20	0.06	0.97	-0.39	-0.19	0.06	-0.19	0.07	0.94
2890	GRIA1	-0.47	-0.17	0.11	-0.17	0.08	0.90	-0.32	-0.17	-0.04	-0.18	0.07	0.96	-0.39	-0.19	-0.01	-0.19	0.08	0.94
2898	GRIK2	-0.39	-0.17	0.11	-0.17	0.07	0.95	-0.27	-0.16	-0.03	-0.16	0.05	0.96	-0.32	-0.17	0.02	-0.18	0.07	0.94
2911	GRM1	-0.39	-0.18	0.11	-0.18	0.07	0.94	-0.33	-0.17	-0.03	-0.18	0.06	0.97	-0.34	-0.19	-0.03	-0.19	0.07	0.96
2918	GRM8	-0.35	-0.17	0.06	-0.17	0.06	0.94	-0.29	-0.17	-0.06	-0.17	0.05	0.99	-0.33	-0.20	-0.05	-0.20	0.06	0.99
2937	GSS	-0.34	-0.15	0.08	-0.16	0.06	0.95	-0.29	-0.16	-0.04	-0.16	0.06	0.95	-0.30	-0.17	-0.03	-0.17	0.06	0.95
2947	GSTM3	-0.38	-0.15	0.09	-0.15	0.07	0.91	-0.28	-0.15	-0.03	-0.16	0.05	0.96	-0.33	-0.16	-0.01	-0.16	0.07	0.93
2952	GSTT1	-0.14	0.22	0.46	0.22	0.08	0.97	0.08	0.22	0.41	0.23	0.07	1.00	0.01	0.23	0.42	0.24	0.08	0.98
3067	HDC	-0.38	-0.19	0.08	-0.19	0.07	0.97	-0.32	-0.19	-0.07	-0.20	0.06	1.00	-0.35	-0.21	-0.03	-0.20	0.07	0.98
3145	HMBS	-0.07	0.16	0.37	0.16	0.07	0.91	0.05	0.16	0.28	0.16	0.06	0.97	0.03	0.18	0.33	0.18	0.06	0.96
3156	HMGCR	-0.31	-0.15	0.09	-0.15	0.06	0.93	-0.28	-0.14	-0.03	-0.15	0.05	0.95	-0.32	-0.16	-0.01	-0.16	0.06	0.93
3231	HOXD1	-0.03	0.12	0.20	0.11	0.04	0.91	0.03	0.14	0.21	0.13	0.03	0.98	0.01	0.13	0.25	0.13	0.04	0.95
3269	HRH1	-0.43	-0.18	0.12	-0.18	0.07	0.95	-0.33	-0.18	-0.05	-0.18	0.06	0.95	-0.39	-0.19	-0.01	-0.20	0.07	0.97
3290	HSD11B1	-0.09	0.16	0.33	0.17	0.07	0.95	0.05	0.21	0.31	0.19	0.06	0.99	-0.02	0.19	0.35	0.20	0.06	0.98
3316	HSPB2	-0.10	0.17	0.37	0.17	0.06	0.96	0.06	0.16	0.30	0.16	0.05	0.98	0.04	0.19	0.32	0.18	0.06	0.96
3338	DNAJC4	-0.13	0.16	0.39	0.16	0.07	0.91	0.00	0.15	0.31	0.15	0.06	0.94	-0.03	0.17	0.33	0.17	0.07	0.93
3350	HTR1A	-0.42	-0.18	0.09	-0.18	0.08	0.93	-0.35	-0.17	0.02	-0.18	0.07	0.98	-0.35	-0.19	0.00	-0.19	0.07	0.96
3358	HTR2C	-0.40	-0.16	0.10	-0.16	0.08	0.90	-0.33	-0.15	0.07	-0.16	0.07	0.92	-0.37	-0.19	0.00	-0.19	0.07	0.96
3396	MRPL58	-0.13	0.16	0.36	0.16	0.06	0.94	0.03	0.15	0.28	0.15	0.05	0.94	0.01	0.16	0.32	0.16	0.06	0.92
3485	IGFBP2	-0.08	0.16	0.43	0.16	0.07	0.91	0.04	0.17	0.32	0.17	0.06	0.98	0.00	0.18	0.37	0.18	0.07	0.94
3595	IL12RB2	-0.29	-0.14	0.06	-0.13	0.05	0.91	-0.24	-0.14	-0.04	-0.14	0.05	0.94	-0.26	-0.16	-0.03	-0.16	0.05	0.96
3598	IL13RA2	-0.43	-0.18	0.11	-0.18	0.08	0.93	-0.37	-0.17	0.00	-0.19	0.08	0.95	-0.40	-0.20	-0.01	-0.20	0.08	0.96
3624	INHBA	-0.04	0.17	0.32	0.17	0.05	0.96	0.03	0.18	0.30	0.18	0.05	0.99	0.06	0.19	0.32	0.19	0.05	1.00
3632	INPP5A	-0.13	0.16	0.32	0.16	0.06	0.96	0.03	0.17	0.30	0.17	0.05	0.96	0.01	0.17	0.31	0.17	0.06	0.94
3653	IPW	-0.15	0.18	0.42	0.18	0.08	0.94	0.00	0.19	0.38	0.19	0.07	0.98	0.01	0.20	0.37	0.20	0.08	0.95
3671	ISLR	-0.08	0.14	0.31	0.14	0.07	0.90	0.04	0.17	0.29	0.17	0.05	0.96	-0.02	0.14	0.29	0.15	0.06	0.90
3679	ITGA7	-0.03	0.14	0.27	0.14	0.05	0.92	0.06	0.15	0.25	0.16	0.04	0.99	0.02	0.16	0.28	0.16	0.04	0.98
3688	ITGB1	-0.03	0.14	0.25	0.14	0.04	0.95	0.06	0.16	0.25	0.15	0.04	0.98	0.05	0.16	0.25	0.16	0.04	0.98
3736	KCNA1	-0.14	0.19	0.45	0.20	0.08	0.95	0.06	0.21	0.40	0.21	0.07	0.99	-0.03	0.21	0.41	0.22	0.08	0.96
3738	KCNA3	-0.45	-0.20	0.14	-0.21	0.09	0.96	-0.37	-0.20	-0.05	-0.21	0.07	0.98	-0.45	-0.22	0.01	-0.22	0.09	0.96
3741	KCNA5	-0.44	-0.19	0.13	-0.19	0.09	0.95	-0.37	-0.21	-0.06	-0.21	0.08	0.99	-0.42	-0.20	0.01	-0.21	0.09	0.92
3746	KCNC1	-0.08	0.15	0.39	0.16	0.07	0.92	0.05	0.16	0.34	0.16	0.06	0.96	-0.01	0.17	0.34	0.17	0.07	0.93
3755	KCNG1	-0.47	-0.22	0.12	-0.23	0.09	0.97	-0.45	-0.25	-0.08	-0.24	0.08	1.00	-0.43	-0.24	-0.01	-0.25	0.09	0.98
3782	KCNN3	-0.39	-0.16	0.09	-0.16	0.07	0.91	-0.32	-0.17	-0.02	-0.18	0.06	0.96	-0.32	-0.17	0.02	-0.17	0.07	0.93
3783	KCNN4	-0.13	0.18	0.32	0.18	0.06	0.97	0.05	0.18	0.28	0.18	0.05	0.96	0.03	0.18	0.32	0.17	0.06	0.95
3787	KCNS1	-0.11	0.16	0.41	0.17	0.08	0.91	0.02	0.17	0.37	0.18	0.07	0.95	-0.02	0.18	0.38	0.19	0.08	0.94
3827	KNG1	-0.08	0.17	0.39	0.17	0.07	0.93	0.02	0.17	0.32	0.17	0.06	0.97	-0.01	0.18	0.35	0.19	0.07	0.96
3902	LAG3	-0.12	0.22	0.51	0.22	0.09	0.95	0.06	0.24	0.43	0.23	0.08	0.98	0.01	0.24	0.46	0.24	0.10	0.98
3910	LAMA4	-0.10	0.19	0.41	0.20	0.07	0.96	0.00	0.20	0.33	0.20	0.06	0.97	0.02	0.21	0.36	0.20	0.07	0.97
4062	LY6H	-0.53	-0.22	0.13	-0.23	0.09	0.96	-0.44	-0.23	-0.05	-0.23	0.08	0.99	-0.44	-0.25	-0.04	-0.25	0.09	0.99
4082	MARCKS	-0.40	-0.18	0.09	-0.18	0.07	0.94	-0.35	-0.19	-0.04	-0.19	0.07	0.95	-0.39	-0.21	0.01	-0.21	0.07	0.98
4088	SMAD3	-0.37	-0.15	0.09	-0.15	0.07	0.91	-0.31	-0.14	-0.02	-0.15	0.06	0.95	-0.32	-0.16	-0.01	-0.16	0.07	0.93
4092	SMAD7	-0.07	0.15	0.33	0.15	0.06	0.92	0.04	0.18	0.30	0.18	0.05	0.96	0.00	0.18	0.36	0.18	0.06	0.97
4157	MC1R	-0.10	0.17	0.41	0.18	0.08	0.94	0.04	0.17	0.35	0.18	0.07	0.94	0.00	0.19	0.39	0.19	0.08	0.95
4168	MCF2	-0.10	0.16	0.39	0.16	0.07	0.91	0.04	0.16	0.33	0.16	0.06	0.94	-0.02	0.17	0.32	0.17	0.07	0.93
4257	MGST1	-0.42	-0.17	0.12	-0.17	0.08	0.91	-0.35	-0.18	-0.03	-0.19	0.07	0.97	-0.38	-0.18	0.02	-0.19	0.08	0.93
4258	MGST2	-0.13	0.22	0.45	0.22	0.08	0.98	0.05	0.22	0.39	0.23	0.07	0.99	0.04	0.23	0.41	0.23	0.08	0.99
4286	MITF	-0.06	0.19	0.35	0.18	0.06	0.97	0.07	0.19	0.30	0.19	0.05	1.00	0.03	0.20	0.33	0.20	0.06	0.98

4324	MMP15	-0.12	0.16	0.34	0.16	0.06	0.93	0.05	0.16	0.30	0.17	0.05	0.97	0.02	0.17	0.33	0.17	0.06	0.97
4345	CD200	-0.24	-0.12	0.03	-0.12	0.04	0.91	-0.24	-0.12	-0.03	-0.13	0.04	0.96	-0.24	-0.13	0.01	-0.13	0.04	0.94
4354	MPP1	-0.12	0.18	0.44	0.19	0.08	0.93	0.07	0.20	0.37	0.20	0.07	0.98	0.02	0.20	0.39	0.20	0.08	0.95
4599	MX1	-0.11	0.18	0.40	0.18	0.07	0.95	0.01	0.19	0.36	0.19	0.06	0.99	0.02	0.21	0.37	0.21	0.07	0.98
4602	MYB	-0.39	-0.17	0.13	-0.17	0.08	0.91	-0.32	-0.16	0.01	-0.17	0.06	0.96	-0.35	-0.18	0.02	-0.18	0.08	0.94
4609	MYC	-0.09	0.20	0.38	0.20	0.07	0.97	0.06	0.20	0.37	0.20	0.06	0.99	0.00	0.21	0.36	0.21	0.07	0.97
4616	GADD45B	-0.10	0.14	0.31	0.14	0.06	0.92	0.05	0.15	0.31	0.15	0.05	0.96	0.02	0.15	0.28	0.15	0.06	0.94
4625	MYH7	-0.43	-0.16	0.12	-0.16	0.08	0.91	-0.31	-0.16	-0.03	-0.17	0.06	0.94	-0.35	-0.18	0.02	-0.19	0.07	0.95
4638	MYLK	-0.02	0.16	0.26	0.15	0.04	0.98	0.08	0.17	0.25	0.17	0.04	1.00	0.01	0.17	0.26	0.17	0.04	0.99
4703	NEB	-0.14	0.23	0.41	0.23	0.08	0.98	0.12	0.23	0.40	0.25	0.07	1.00	0.05	0.25	0.44	0.24	0.08	0.99
4715	NDUFB9	-0.16	0.17	0.34	0.17	0.06	0.94	0.03	0.16	0.29	0.16	0.05	0.98	-0.01	0.17	0.33	0.16	0.07	0.92
4744	NEFH	-0.12	0.19	0.41	0.19	0.08	0.95	0.07	0.19	0.39	0.20	0.06	1.00	-0.04	0.20	0.41	0.20	0.08	0.96
4751	NEK2	-0.44	-0.22	0.12	-0.22	0.08	0.97	-0.39	-0.22	-0.07	-0.23	0.07	0.99	-0.42	-0.23	-0.01	-0.23	0.08	0.98
4753	NELL2	-0.29	-0.14	0.04	-0.14	0.05	0.92	-0.25	-0.13	-0.02	-0.14	0.05	0.92	-0.29	-0.15	-0.03	-0.15	0.05	0.96
4782	NFIC	-0.08	0.15	0.34	0.15	0.06	0.90	0.02	0.15	0.30	0.15	0.06	0.94	-0.02	0.17	0.31	0.17	0.06	0.95
4824	NKX3-1	-0.15	0.15	0.38	0.16	0.07	0.92	0.03	0.15	0.30	0.15	0.06	0.95	-0.01	0.16	0.32	0.17	0.07	0.93
4856	NOV	-0.50	-0.21	0.11	-0.21	0.09	0.95	-0.41	-0.22	-0.06	-0.22	0.08	0.98	-0.47	-0.24	0.00	-0.24	0.09	0.98
4885	NPTX2	-0.31	-0.16	0.09	-0.16	0.06	0.93	-0.31	-0.17	-0.02	-0.17	0.05	0.96	-0.33	-0.17	0.00	-0.17	0.06	0.96
4886	NPY1R	-0.41	-0.19	0.14	-0.19	0.08	0.96	-0.34	-0.19	-0.03	-0.19	0.07	0.97	-0.38	-0.20	0.03	-0.20	0.08	0.96
4889	NPY5R	-0.27	-0.15	0.06	-0.15	0.05	0.95	-0.23	-0.14	-0.04	-0.14	0.04	0.97	-0.27	-0.15	-0.02	-0.15	0.05	0.96
4988	OPRM1	-0.44	-0.18	0.12	-0.18	0.08	0.94	-0.37	-0.20	-0.04	-0.19	0.08	0.96	-0.40	-0.20	0.03	-0.20	0.09	0.94
5090	PBX3	-0.08	0.14	0.28	0.14	0.05	0.95	0.05	0.15	0.25	0.15	0.05	0.95	0.00	0.16	0.26	0.15	0.05	0.95
5091	PC	-0.05	0.15	0.33	0.14	0.05	0.92	0.04	0.14	0.28	0.14	0.05	0.96	-0.01	0.15	0.29	0.15	0.05	0.95
5116	PCNT	-0.12	0.19	0.41	0.19	0.07	0.96	0.06	0.20	0.38	0.21	0.07	0.98	0.02	0.20	0.40	0.20	0.08	0.97
5121	PCP4	-0.13	0.19	0.39	0.19	0.07	0.96	0.07	0.19	0.34	0.20	0.06	0.99	0.03	0.21	0.38	0.20	0.07	0.98
5138	PDE2A	-0.37	-0.16	0.07	-0.16	0.07	0.93	-0.32	-0.16	-0.03	-0.16	0.06	0.94	-0.34	-0.18	-0.01	-0.18	0.07	0.96
5141	PDE4A	-0.15	0.21	0.45	0.21	0.08	0.96	0.03	0.21	0.40	0.22	0.07	0.99	0.00	0.21	0.40	0.21	0.08	0.95
5176	SERPINF1	-0.32	-0.19	0.09	-0.19	0.06	0.97	-0.30	-0.20	-0.01	-0.20	0.05	0.99	-0.33	-0.20	-0.02	-0.20	0.06	0.98
5184	PEPD	-0.07	0.15	0.34	0.15	0.06	0.95	0.05	0.16	0.28	0.16	0.05	0.97	0.03	0.17	0.30	0.17	0.05	0.98
5272	SERPINB9	-0.12	0.17	0.43	0.18	0.08	0.93	0.02	0.17	0.36	0.18	0.07	0.98	0.01	0.18	0.36	0.19	0.07	0.94
5292	PIM1	-0.11	0.17	0.39	0.17	0.07	0.92	0.01	0.18	0.36	0.18	0.07	0.96	-0.02	0.18	0.35	0.18	0.07	0.93
5310	PKD1	-0.08	0.15	0.34	0.15	0.06	0.92	0.03	0.16	0.29	0.16	0.05	0.95	0.01	0.16	0.29	0.16	0.06	0.95
5325	PLAGL1	-0.15	0.17	0.43	0.17	0.08	0.91	-0.01	0.17	0.37	0.18	0.08	0.94	-0.06	0.18	0.39	0.18	0.08	0.93
5332	PLCB4	-0.07	0.16	0.40	0.16	0.07	0.93	0.04	0.17	0.32	0.17	0.06	0.95	0.02	0.19	0.34	0.19	0.07	0.95
5361	PLXNA1	-0.34	-0.15	0.11	-0.16	0.07	0.93	-0.26	-0.15	-0.01	-0.15	0.05	0.94	-0.35	-0.16	0.04	-0.16	0.07	0.93
5409	PNMT	-0.46	-0.20	0.13	-0.21	0.08	0.95	-0.40	-0.21	-0.04	-0.21	0.08	0.98	-0.42	-0.23	0.02	-0.23	0.08	0.98
5412	UBL3	-0.11	0.18	0.36	0.18	0.06	0.96	0.06	0.18	0.31	0.18	0.05	0.98	0.03	0.19	0.34	0.19	0.06	0.97
5467	PPARD	-0.13	0.16	0.40	0.17	0.07	0.92	0.01	0.17	0.33	0.17	0.07	0.95	0.00	0.19	0.37	0.19	0.07	0.97
5475	PPEF1	-0.44	-0.18	0.11	-0.18	0.08	0.93	-0.36	-0.19	-0.03	-0.19	0.08	0.96	-0.38	-0.20	0.04	-0.20	0.09	0.95
5547	PRCP	-0.07	0.15	0.30	0.15	0.05	0.96	0.04	0.15	0.28	0.15	0.05	0.98	-0.03	0.16	0.29	0.16	0.05	0.97
5569	PKIA	-0.52	-0.21	0.12	-0.21	0.09	0.94	-0.41	-0.22	-0.05	-0.22	0.08	0.98	-0.44	-0.23	-0.02	-0.24	0.09	0.98
5579	PRKCB	-0.32	-0.15	0.04	-0.15	0.06	0.92	-0.27	-0.14	-0.02	-0.14	0.05	0.91	-0.31	-0.16	0.02	-0.16	0.07	0.91
5580	PRKCD	-0.51	-0.23	0.12	-0.22	0.09	0.96	-0.46	-0.24	-0.05	-0.24	0.08	0.98	-0.44	-0.24	-0.01	-0.25	0.09	0.98
5582	PRKCG	-0.47	-0.21	0.09	-0.20	0.08	0.94	-0.41	-0.21	-0.05	-0.21	0.08	0.97	-0.43	-0.22	-0.02	-0.22	0.08	0.98
5594	MAPK1	-0.38	-0.15	0.06	-0.15	0.06	0.90	-0.31	-0.15	-0.01	-0.16	0.06	0.95	-0.31	-0.17	-0.02	-0.17	0.06	0.95
5600	MAPK11	-0.24	-0.12	0.06	-0.13	0.05	0.92	-0.23	-0.11	-0.02	-0.12	0.04	0.91	-0.24	-0.13	0.02	-0.13	0.05	0.91
5606	MAP2K3	-0.08	0.16	0.36	0.16	0.07	0.92	0.01	0.17	0.30	0.17	0.06	0.96	0.02	0.18	0.32	0.18	0.06	0.95
5655	KLK10	-0.42	-0.18	0.12	-0.17	0.08	0.90	-0.37	-0.18	-0.01	-0.19	0.07	0.97	-0.37	-0.20	0.03	-0.19	0.08	0.94
5733	PTGER3	-0.45	-0.19	0.10	-0.19	0.09	0.92	-0.39	-0.19	0.00	-0.20	0.08	0.96	-0.40	-0.20	0.00	-0.21	0.09	0.94
5774	PTPN3	-0.27	-0.15	0.05	-0.15	0.05	0.96	-0.26	-0.16	-0.04	-0.16	0.04	0.99	-0.27	-0.16	0.01	-0.15	0.05	0.97
5786	PTPRA	-0.44	-0.16	0.09	-0.17	0.08	0.90	-0.35	-0.17	-0.01	-0.17	0.07	0.93	-0.35	-0.18	0.00	-0.18	0.08	0.93
5792	PTPRF	-0.44	-0.18	0.14	-0.18	0.08	0.94	-0.31	-0.17	-0.03	-0.18	0.07	0.97	-0.40	-0.19	0.01	-0.19	0.08	0.94
5794	PTPRH	-0.01	0.14	0.23	0.14	0.04	0.93	0.04	0.16	0.24	0.16	0.04	0.99	0.03	0.16	0.28	0.15	0.04	0.96
5800	PTPRO	-0.23	-0.13	0.01	-0.13	0.04	0.95	-0.21	-0.12	-0.06	-0.13	0.03	0.97	-0.24	-0.14	-0.02	-0.14	0.04	0.97
5801	PTPRR	-0.38	-0.16	0.07	-0.16	0.07	0.92	-0.35	-0.17	-0.05	-0.17	0.06	0.93	-0.36	-0.20	0.00	-0.20	0.07	0.98
5816	PVALB	-0.11	0.18	0.44	0.19	0.08	0.94	0.04	0.19	0.38	0.20	0.07	0.96	0.01	0.20	0.38	0.21	0.08	0.95
5860	QDPR	-0.04	0.12	0.20	0.12	0.04	0.93	0.06	0.14	0.23	0.14	0.04	0.98	0.01	0.14	0.24	0.14	0.04	0.95
5874	RAB27B	-0.49	-0.21	0.09	-0.21	0.08	0.96	-0.41	-0.22	-0.06	-0.22	0.07	0.99	-0.41	-0.23	-0.06	-0.23	0.08	0.99
5912	RAP2B	-0.44	-0.18	0.10	-0.18	0.08	0.93	-0.37	-0.19	-0.03	-0.19	0.07	0.98	-0.39	-0.21	-0.02	-0.21	0.08	0.97
5937	RBMS1	-0.12	0.17	0.41	0.18	0.08	0.93	-0.01	0.19	0.37	0.19	0.07	0.96	0.02	0.20	0.35	0.20	0.07	0.95
5950	RBP4	-0.51	-0.21	0.14	-0.21	0.10	0.94	-0.45	-0.23	-0.05	-0.23	0.08	0.98	-0.46	-0.23	0.01	-0.24	0.10	0.96
5954	RCN1	-0.33	-0.17	0.12	-0.17	0.07	0.96	-0.30	-0.17	0.00	-0.18	0.06	0.96	-0.34	-0.17	0.01	-0.17	0.07	0.93
5961	PRPH2	-0.32	-0.16	0.05	-0.15	0.06	0.91	-0.31	-0.16	0.03	-0.16	0.05	0.97	-0.33	-0.18	-0.02	-0.18	0.06	0.97
5979	RET	-0.12	0.19	0.45	0.19	0.08	0.94	0.01	0.18	0.37	0.19	0.07	0.97	0.00	0.19	0.37	0.20	0.07	0.96
5991	RFX3	-0.23	-0.13	0.04	-0.13	0.04	0.93	-0.21	-0.12	-0.04	-0.12	0.04	0.93	-0.21	-0.13	0.00	-0.12	0.04	0.91
5993	RFX5	-0.14	0.18	0.41	0.19	0.07	0.96	0.05	0.19	0.36	0.19	0.06	0.98	0.03	0.19	0.37	0.20	0.07	0.97
6017	RLBP1	-0.33	-0.16	0.09	-0.16	0.06	0.95	-0.24	-0.16	-0.04	-0.16	0.04	0.98	-0.30	-0.16	-0.01	-0.16	0.06	0.96
6272	SORT1	-0.07	0.15	0.30	0.15	0.06	0.94	0.06	0.18	0.28	0.17	0.05	0.99	0.04	0.17	0.29	0.17	0.06	0.96
6323	SCN1A	-0.13	0.18	0.46	0.18	0.09	0.91	0.02	0.19	0.39	0.20	0.08	0.97	-0.04	0.21	0.39	0.21	0.09	0.94
6324	SCN1B	-0.13	0.20	0.49	0.20	0.09	0.94	0.05	0.21	0.41	0.21	0.08	0.97	-0.02	0.22	0.41	0.22	0.09	0.96
6326	SCN2A	-0.26	-0.14	0.03	-0.14	0.05	0.94	-0.23	-0.13	-0.05	-0.14	0.04	0.98	-0.29	-0.15	-0.03	-0.15	0.05	0.96
6330	SCN4B	-0.14	0.24	0.48	0.24	0.08	0.98	0.11	0.25	0.45	0.25	0.07	1.00	0.01	0.25	0.45	0.26	0.08	1.00
6451	SH3BGRL	-0.11	0.16	0.39	0.16	0.07	0.93	0.02	0.16	0.31	0.16	0.06	0.94	0.00	0.17	0.32	0.17	0.06	0.97
6509	SLC1A4	-0.33	-0.14	0.05	-0.14	0.06	0.93	-0.28	-0.15	-0.06	-0.15	0.05	0.98	-0.29	-0.14	-0.01	-0.15	0.06	0.91
6513	SLC2A1	-0.04	0.12	0.24	0.12	0.04	0.90	0.05	0.13	0.23	0.14	0.04	0.97	0.03	0.14	0.23	0.13	0.04	0.93
6533	SLC6A6	-0.09	0.16	0.35	0.16	0.06	0.93	0.04	0.17	0.30	0.17	0.06	0.97	0.03	0.17	0.34	0.17	0.06	0.95
6543	SLC8A2	-0.30	-0.16	0.10	-0.16	0.06	0.95	-0.27	-0.14	-0.04	-0.15	0.05	0.97	-0.29	-0.17	0.01	-0.17	0.06	0.96
6567	SLC16A2	-0.43	-0.17	0.09	-0.17	0.08	0.90	-0.34	-0.16	-0.02	-0.17	0.07	0.93	-0.36	-0.18	0.00	-0.19	0.08	0.92
6585	SLIT1	-0.52	-0.19	0.11	-0.19	0.09	0.92	-0.38	-0.19	0.00	-0.19	0.08	0.97	-0.40	-0.21	0.02	-0.21	0.09	0.96
6623	SNCG	-0.30	-0.15	0.04	-0.15	0.06	0.90	-0.29	-0.16	-0.01	-0.16	0.05	0.97	-0.30	-0.17	-0.01	-0.17	0.06	0.95
6646	SOAT1	-0.10	0.17	0.37	0.17	0.07	0.92	-0.01	0.17	0.34	0.18	0.06	0.98	0.01	0.19	0.33	0.18	0.07	0.97
6696	SPP1	-0.02	0.12	0.22	0.12	0.04	0.92	0.04	0.14	0.22	0.14	0.04	0.95	-0.01	0.14	0.25	0.14	0.04	0.95
6751	SSTR1	-0.48	-0.19	0.14	-0.19	0.09	0.91	-0.40	-0.19	-0.01	-0.19	0.08	0.95	-0.41	-0.20	0.04	-0.21	0.09	0.93
6804	STX1A	-0.36	-0.20	0.07	-0.20	0.07	0.98	-0.35	-0.20	-0.06	-0.20	0.06	0.99	-0.36	-0.22	-0.05	-0.22	0.07	0.99
6843	VAMP1	-0.14	0.20	0.47	0.20	0.09	0.94	0.03	0.21	0.41	0.21	0.08	0.98	-0.01	0.22	0.41	0.22	0.09	0.97
6854	SYN2	-0.40	-0.16	0.07	-0.16	0.06	0.93	-0.30	-0.15	-0.04	-0.16	0.05	0.97	-0.31	-0.17	-0.03	-0.17	0.06	0.96
6890	TAP1	-0.04	0.18	0.34	0.17	0.06	0.94	0.06	0.19	0.32	0.19	0.05	0.99	0.02	0.20	0.33	0.20	0.06	0.99
6905	TBCE	-0.12	0.19	0.44	0.19	0.08	0.95	0.03	0.18	0.35	0.18	0.07	0.95	0.00	0.19	0.36	0.19	0.08	0.95
6920	TCEA3	-0.44	-0.18	0.11	-0.18	0.08	0.93	-0.39	-0.20	-0.05	-0.20	0.07	0.98	-0.38	-0.20	-0.01	-0.21	0.08	0.95
7062	TCHH	-0.10	0.17	0.36	0.17	0.06	0.95	0.06	0.17	0.30	0.17	0.05	0.98	-0.01	0.17	0.34	0.18	0.07	0.95
7068	THRB	-0.37	-0.18	0.07	-0.18	0.06	0.96	-0.29	-0.18	-0.08	-0.19	0.05	1.00	-0.35	-0.19	-0.05	-0.19	0.06	0.98
7087	ICAM5	-0.36	-0.14	0.06	-0.14	0.06	0.91	-0.27	-0.13	0.01	-0.14	0.05	0.95	-0.27	-0.15	-0.03	-0.15	0.06	0.94
7089	TLE2	-0.09	0.18	0.41	0.18	0.07	0.94	0.03	0.18	0.36	0.18	0.07	0.97	0.02	0.19	0.37	0.19	0.07	0.96
7092	TLL1	-0.34	-0.16	0.07	-0.16	0.06	0.93	-0.31	-0.16	0.04	-0.16	0.06	0.97	-0.32	-0.18	-0.02	-0.18	0.06	0.97
7103	TSPAN8	-0.03	0.15	0.26	0.15	0.05	0.95	0.05	0.15	0.25	0.16	0.04	0.97	0.00	0.16	0.28	0.15	0.05	0.95
7138	TNNT1	-0.41	-0.18	0.13	-0.18	0.08	0.94	-0.37	-0.19	-0.01	-0.19	0.06	0.97	-0.36	-0.19	0.00	-0.19	0.08	0.96
7145	TNS1	-0.03	0.14	0.23	0.13	0.04	0.94	0.04	0.14	0.24	0.14	0.04	0.98	0.04	0.15	0.27	0.15	0.05	0.95
7222	TRPC3	-0.13	0.21	0.52	0.21	0.09	0.94	0.04	0.21	0.43	0.22	0.08	0.97	-0.02	0.22	0.42	0.22	0.09	0.98
7301	TYRO3	-0.33	-0.16	0.11	-0.16	0.06	0.93	-0.28	-0.15	-0.01	-0.15	0.05	0.97	-0.30	-0.17	0.00	-0.17	0.06	0.95
7378	UPP1	-0.11	0.18	0.46	0.19	0.08	0.93	0.02	0.19	0.39	0.19	0.08	0.96	-0.01	0.19	0.38	0.19	0.09	0.93
7402	UTRN	-0.16	0.16	0.33	0.16	0.07	0.94	0.01	0.16	0.27	0.16	0.06	0.94	-0.01	0.16	0.31	0.16	0.06	0.92
7480	WNT10B	-0.46	-0.19	0.08	-0.19	0.08	0.94	-0.37	-0.19	-0.04	-0.20	0.07	0.97	-0.40	-0.21	-0.01	-0.21	0.08	0.96
7533	YWHAH	-0.33	-0.16	0.07	-0.16	0.06	0.93	-0.27	-0.15	-0.03	-0.16	0.05	0.95	-0.32	-0.18	-0.02	-0.17	0.06	0.97
7534	YWHAZ	-0.32	-0.16	0.04	-0.16	0.06	0.95	-0.30	-0.15	-0.03	-0.16	0.05	0.98	-0.30	-0.17	-0.02	-0.17	0.06	0.96
7726	TRIM26	-0.09	0.15	0.33	0.15	0.06	0.93	0.06	0.15	0.28	0.15	0.05	0.98	0.04	0.16	0.30	0.17	0.06	0.96
7781	SLC30A3	-0.32	-0.18	0.10	-0.18	0.06	0.98	-0.30	-0.18	-0.04	-0.18	0.05	0.99	-0.34	-0.19	-0.01	-0.18	0.06	0.97
7791	ZYX	-0.11	0.17	0.43	0.17	0.08	0.92	0.03	0.17	0.35	0.18	0.07	0.95	0.00	0.18	0.35	0.19	0.08	0.93
8001	GLRA3	-0.45	-0.20	0.14	-0.20	0.08	0.97	-0.34	-0.19	-0.05	-0.20	0.07	0.98	-0.40	-0.21	0.00	-0.21	0.08	0.96
8013	NR4A3	-0.39	-0.15	0.07	-0.16	0.08	0.90	-0.32	-0.17	-0.05	-0.16	0.07	0.92	-0.36	-0.17	0.02	-0.18	0.08	0.93
8174	MADCAM1	-0.09	0.17	0.40	0.17	0.07	0.93	0.01	0.17	0.34	0.18	0.07	0.97	0.00	0.19	0.36	0.19	0.07	0.97
8209	C21orf33	-0.06	0.14	0.25	0.14	0.05	0.95	0.05	0.16	0.26	0.16	0.05	0.98	0.00	0.16	0.29	0.16	0.05	0.95
8321	FZD1	-0.41	-0.17	0.10	-0.17	0.08	0.93	-0.35	-0.18	-0.05	-0.18	0.07	0.99	-0.38	-0.18	0.00	-0.18	0.08	0.92
8404	SPARCL1	-0.09	0.14	0.30	0.14	0.05	0.94	0.04	0.15	0.27	0.15	0.05	0.99	-0.01	0.15	0.29	0.15	0.05	0.94
8437	RASAL1	-0.45	-0.18	0.14	-0.18	0.08	0.93	-0.35	-0.17	0.01	-0.18	0.07	0.96	-0.36	-0.19	0.02	-0.19	0.08	0.94
8445	DYRK2	-0.34	-0.16	0.09	-0.16	0.06	0.94	-0.30	-0.17	-0.04	-0.17	0.06	0.95	-0.33	-0.18	-0.01	-0.17	0.07	0.93
8482	SEMA7A	-0.15	0.20	0.50	0.21	0.09	0.94	0.05	0.21	0.42	0.22	0.08	0.98	0.00	0.22	0.42	0.23	0.09	0.97
8604	SLC25A12	-0.12	0.17	0.39	0.17	0.07	0.94	0.04	0.18	0.35	0.19	0.06	0.96	0.00	0.17	0.33	0.18	0.07	0.93
8611	PLPP1	-0.04	0.15	0.29	0.15	0.05	0.93	0.06	0.15	0.25	0.16	0.04	0.98	0.01	0.16	0.29	0.16	0.05	0.98
8704	B4GALT2	-0.40	-0.20	0.14	-0.20	0.08	0.97	-0.34	-0.21	-0.06	-0.21	0.07	0.99	-0.38	-0.21	-0.01	-0.22	0.08	0.97
8706	B3GALNT1	-0.33	-0.14	0.06	-0.14	0.06	0.91	-0.27	-0.15	-0.04	-0.15	0.06	0.94	-0.32	-0.16	-0.02	-0.17	0.06	0.95
8715	NOL4	-0.41	-0.20	0.12	-0.20	0.07	0.97	-0.35	-0.20	-0.08	-0.20	0.06	1.00	-0.37	-0.21	-0.01	-0.21	0.08	0.98
8717	TRADD	-0.06	0.14	0.26	0.14	0.05	0.95	0.06	0.14	0.24	0.15	0.04	0.99	0.00	0.15	0.27	0.15	0.05	0.96
8792	TNFRSF11A	-0.40	-0.19	0.11	-0.19	0.08	0.95	-0.35	-0.19	0.01	-0.19	0.06	0.96	-0.39	-0.22	-0.02	-0.21	0.07	0.97
8805	TRIM24	-0.28	-0.14	0.08	-0.14	0.05	0.93	-0.27	-0.14	-0.02	-0.15	0.05	0.98	-0.28	-0.15	0.01	-0.15	0.05	0.94
8871	SYNJ2	0.00	0.12	0.21	0.12	0.04	0.93	0.04	0.15	0.22	0.14	0.04	0.95	0.00	0.14	0.24	0.13	0.05	0.92
8877	SPHK1	-0.10	0.14	0.34	0.15	0.06	0.91	-0.01	0.14	0.26	0.14	0.06	0.92	-0.03	0.16	0.29	0.16	0.06	0.92
8884	SLC5A6	-0.09	0.17	0.40	0.18	0.07	0.94	0.05	0.19	0.34	0.19	0.06	0.98	0.03	0.19	0.35	0.20	0.07	0.95
8938	BAIAP3	-0.56	-0.24	0.16	-0.25	0.10	0.98	-0.47	-0.25	-0.10	-0.26	0.09	1.00	-0.49	-0.27	-0.02	-0.27	0.10	0.98
9020	MAP3K14	-0.09	0.16	0.32	0.16	0.06	0.94	0.06	0.17	0.28	0.17	0.05	0.97	0.03	0.17	0.31	0.17	0.06	0.96
9033	PKD2L1	-0.37	-0.21	0.12	-0.20	0.07	0.98	-0.32	-0.20	-0.05	-0.21	0.06	0.99	-0.39	-0.21	-0.02	-0.21	0.07	0.97
9120	SLC16A6	-0.12	0.18	0.45	0.18	0.08	0.94	0.05	0.18	0.34	0.18	0.07	0.97	0.00	0.19	0.37	0.19	0.08	0.94
9127	P2RX6	-0.10	0.20	0.45	0.20	0.08	0.96	-0.01	0.21	0.41	0.22	0.07	0.98	0.03	0.22	0.42	0.22	0.08	0.98
9168	TMSB10	-0.44	-0.19	0.08	-0.18	0.08	0.94	-0.38	-0.19	0.00	-0.20	0.07	0.97	-0.38	-0.21	0.01	-0.21	0.08	0.98
9196	KCNAB3	-0.12	0.20	0.49	0.21	0.09	0.95	0.05	0.21	0.41	0.21	0.08	0.98	-0.01	0.21	0.41	0.22	0.09	0.96
9215	LARGE1	-0.42	-0.17	0.08	-0.17	0.08	0.90	-0.36	-0.17	-0.01	-0.18	0.07	0.95	-0.38	-0.19	0.02	-0.19	0.08	0.95
9253	NUMBL	-0.28	-0.14	0.00	-0.14	0.05	0.93	-0.27	-0.15	-0.06	-0.15	0.04	0.97	-0.28	-0.16	-0.01	-0.16	0.05	0.96
9254	CACNA2D2	-0.08	0.16	0.36	0.16	0.06	0.94	0.03	0.16	0.32	0.16	0.06	0.96	0.02	0.17	0.31	0.17	0.06	0.96
9256	TSPOAP1	-0.34	-0.17	0.06	-0.17	0.06	0.93	-0.30	-0.18	-0.07	-0.18	0.05	0.99	-0.35	-0.19	-0.02	-0.19	0.06	0.97
9312	KCNB2	-0.34	-0.19	0.08	-0.19	0.06	0.97	-0.30	-0.20	-0.06	-0.20	0.05	0.99	-0.38	-0.21	-0.02	-0.21	0.06	0.98
9315	NREP	-0.10	0.16	0.37	0.16	0.07	0.93	0.06	0.18	0.34	0.18	0.06	0.98	0.00	0.17	0.32	0.18	0.07	0.96
9362	CPNE6	-0.49	-0.20	0.12	-0.20	0.09	0.94	-0.40	-0.19	-0.01	-0.20	0.08	0.98	-0.42	-0.21	0.00	-0.22	0.09	0.96
9399	STOML1	-0.32	-0.15	0.05	-0.15	0.06	0.94	-0.28	-0.15	-0.02	-0.15	0.05	0.98	-0.29	-0.17	-0.03	-0.17	0.06	0.94
9452	ITM2A	-0.11	0.18	0.43	0.18	0.08	0.93	0.03	0.20	0.37	0.20	0.07	0.96	-0.02	0.20	0.38	0.20	0.08	0.96
9454	HOMER3	-0.08	0.18	0.35	0.18	0.06	0.96	0.07	0.18	0.30	0.18	0.05	0.99	0.06	0.20	0.34	0.20	0.06	0.99
9473	THEMIS2	-0.03	0.15	0.30	0.14	0.05	0.91	0.04	0.15	0.26	0.15	0.05	0.98	0.00	0.16	0.32	0.16	0.05	0.96

9495	AKAP5	-0.30	-0.14	0.04	-0.14	0.05	0.93	-0.27	-0.15	-0.05	-0.15	0.04	0.97	-0.27	-0.16	-0.04	-0.16	0.05	0.97
9592	IER2	-0.10	0.16	0.39	0.16	0.07	0.92	0.03	0.18	0.34	0.17	0.06	0.98	0.02	0.18	0.35	0.18	0.07	0.94
9607	CARTPT	-0.43	-0.25	0.12	-0.24	0.09	0.98	-0.42	-0.26	-0.05	-0.26	0.08	0.99	-0.43	-0.25	-0.01	-0.24	0.08	0.97
9609	RAB36	-0.43	-0.18	0.12	-0.18	0.08	0.95	-0.35	-0.19	-0.05	-0.19	0.07	0.98	-0.36	-0.20	-0.03	-0.20	0.08	0.96
9636	ISG15	-0.37	-0.16	0.13	-0.16	0.07	0.94	-0.34	-0.17	-0.01	-0.18	0.05	0.99	-0.36	-0.18	-0.02	-0.18	0.06	0.98
9644	SH3PXD2A	-0.01	0.12	0.25	0.12	0.04	0.90	0.04	0.13	0.23	0.13	0.04	0.94	0.02	0.13	0.24	0.13	0.05	0.92
9651	PLCH2	-0.27	-0.15	0.05	-0.15	0.05	0.94	-0.27	-0.13	-0.03	-0.14	0.05	0.96	-0.27	-0.14	0.02	-0.14	0.05	0.92
9653	HS2ST1	-0.25	-0.12	0.02	-0.12	0.04	0.91	-0.21	-0.14	-0.03	-0.13	0.04	0.91	-0.25	-0.14	-0.03	-0.14	0.04	0.96
9731	CEP104	-0.03	0.12	0.22	0.12	0.04	0.92	0.04	0.13	0.23	0.13	0.04	0.94	0.03	0.12	0.23	0.12	0.04	0.91
9750	RIPOR2	-0.50	-0.20	0.12	-0.20	0.09	0.93	-0.41	-0.20	-0.04	-0.21	0.08	0.98	-0.43	-0.22	0.01	-0.22	0.09	0.96
9770	RASSF2	-0.02	0.11	0.19	0.11	0.03	0.92	0.03	0.12	0.20	0.12	0.04	0.93	0.01	0.12	0.21	0.12	0.04	0.93
9823	ARMCX2	-0.35	-0.15	0.08	-0.15	0.06	0.92	-0.28	-0.15	-0.03	-0.16	0.05	0.95	-0.31	-0.17	-0.03	-0.17	0.06	0.95
9828	ARHGEF17	-0.08	0.14	0.33	0.15	0.06	0.93	0.05	0.17	0.32	0.16	0.06	0.97	0.02	0.16	0.31	0.17	0.06	0.97
9854	C2CD2L	-0.28	-0.14	0.07	-0.14	0.05	0.94	-0.25	-0.13	-0.03	-0.14	0.04	0.98	-0.27	-0.15	-0.02	-0.15	0.05	0.96
9882	TBC1D4	-0.10	0.16	0.40	0.16	0.07	0.91	0.02	0.16	0.35	0.17	0.06	0.94	-0.02	0.17	0.33	0.17	0.07	0.95
9903	KLHL21	-0.08	0.15	0.30	0.15	0.06	0.93	0.07	0.16	0.32	0.17	0.05	0.99	0.03	0.16	0.30	0.16	0.06	0.94
9911	TMCC2	-0.07	0.17	0.37	0.18	0.07	0.94	0.04	0.18	0.33	0.18	0.06	0.98	0.02	0.19	0.37	0.19	0.07	0.96
9957	HS3ST1	-0.12	0.18	0.46	0.19	0.09	0.93	0.01	0.20	0.39	0.20	0.08	0.96	-0.04	0.19	0.42	0.19	0.09	0.91
10023	FRAT1	-0.10	0.17	0.46	0.17	0.08	0.90	0.04	0.17	0.36	0.17	0.07	0.96	-0.02	0.18	0.37	0.18	0.08	0.92
10025	MED16	-0.30	-0.13	0.06	-0.13	0.05	0.91	-0.24	-0.13	-0.01	-0.13	0.05	0.92	-0.25	-0.13	0.00	-0.13	0.05	0.90
10026	PIGK	-0.07	0.14	0.29	0.14	0.05	0.93	0.03	0.15	0.25	0.15	0.04	0.98	-0.01	0.14	0.27	0.14	0.06	0.90
10039	PARP3	-0.12	0.14	0.33	0.14	0.06	0.92	0.02	0.14	0.28	0.14	0.05	0.94	0.03	0.15	0.28	0.15	0.06	0.94
10040	TOM1L1	-0.39	-0.17	0.08	-0.17	0.07	0.92	-0.32	-0.17	-0.04	-0.17	0.06	0.95	-0.37	-0.18	-0.01	-0.19	0.07	0.94
10083	USH1C	-0.03	0.15	0.26	0.15	0.04	0.95	0.07	0.16	0.26	0.16	0.04	1.00	0.01	0.16	0.27	0.16	0.05	0.98
10154	PLXNC1	-0.42	-0.19	0.09	-0.19	0.07	0.97	-0.37	-0.20	-0.09	-0.20	0.06	1.00	-0.36	-0.20	-0.05	-0.21	0.07	0.98
10160	FARP1	-0.46	-0.20	0.07	-0.20	0.08	0.95	-0.36	-0.21	-0.06	-0.21	0.07	0.99	-0.42	-0.21	-0.01	-0.22	0.08	0.96
10171	RCL1	-0.08	0.15	0.34	0.15	0.06	0.94	0.02	0.15	0.29	0.16	0.05	0.96	0.02	0.16	0.29	0.16	0.06	0.95
10174	SORBS3	-0.09	0.15	0.38	0.15	0.07	0.90	0.00	0.15	0.32	0.16	0.07	0.92	-0.01	0.18	0.36	0.18	0.07	0.94
10231	RCAN2	-0.08	0.19	0.40	0.19	0.07	0.95	0.08	0.20	0.40	0.21	0.06	1.00	0.03	0.20	0.38	0.21	0.07	0.98
10268	RAMP3	-0.10	0.17	0.33	0.17	0.06	0.95	0.06	0.17	0.30	0.17	0.05	0.98	0.00	0.18	0.35	0.18	0.06	0.96
10318	TNIP1	-0.12	0.17	0.40	0.17	0.07	0.92	0.03	0.17	0.33	0.17	0.07	0.94	-0.02	0.19	0.36	0.19	0.07	0.96
10332	CLEC4M	-0.07	0.15	0.30	0.15	0.05	0.96	0.03	0.15	0.27	0.15	0.05	0.98	0.02	0.16	0.29	0.16	0.06	0.95
10368	CACNG3	-0.37	-0.17	0.07	-0.17	0.07	0.93	-0.33	-0.17	-0.04	-0.18	0.06	0.97	-0.34	-0.19	-0.01	-0.18	0.07	0.95
10384	BTN3A3	-0.11	0.15	0.28	0.14	0.05	0.94	0.05	0.14	0.25	0.15	0.04	0.99	0.02	0.16	0.27	0.16	0.05	0.96
10395	DLC1	-0.10	0.15	0.29	0.15	0.05	0.96	0.06	0.15	0.26	0.16	0.04	0.98	0.00	0.16	0.30	0.16	0.06	0.94
10402	ST3GAL6	-0.13	0.18	0.45	0.18	0.09	0.93	0.02	0.19	0.40	0.19	0.08	0.95	-0.05	0.19	0.42	0.19	0.09	0.91
10425	ARIH2	-0.11	0.16	0.33	0.16	0.06	0.93	0.03	0.17	0.32	0.17	0.06	0.95	0.02	0.16	0.30	0.17	0.07	0.92
10451	VAV3	-0.17	0.24	0.51	0.24	0.09	0.98	0.07	0.24	0.43	0.25	0.08	1.00	0.00	0.24	0.48	0.24	0.10	0.97
10478	SLC25A17	-0.11	0.15	0.37	0.15	0.06	0.91	0.02	0.14	0.29	0.15	0.05	0.95	0.00	0.15	0.30	0.15	0.06	0.92
10505	SEMA4F	-0.41	-0.19	0.13	-0.20	0.07	0.96	-0.32	-0.19	-0.07	-0.19	0.06	0.99	-0.35	-0.21	-0.01	-0.20	0.07	0.98
10555	AGPAT2	-0.27	-0.12	0.08	-0.12	0.05	0.91	-0.24	-0.14	-0.01	-0.13	0.05	0.91	-0.26	-0.14	-0.02	-0.14	0.05	0.91
10655	DMRT2	0.02	0.11	0.19	0.11	0.03	0.92	0.03	0.13	0.23	0.13	0.04	0.97	0.01	0.13	0.23	0.13	0.04	0.96
10669	CGREF1	-0.32	-0.16	0.05	-0.16	0.06	0.92	-0.33	-0.18	-0.01	-0.18	0.06	0.98	-0.32	-0.19	-0.04	-0.19	0.06	0.97
10673	TNFSF13B	-0.26	-0.14	0.06	-0.14	0.05	0.93	-0.23	-0.14	-0.05	-0.14	0.04	0.98	-0.25	-0.13	0.02	-0.13	0.05	0.92
10752	CHL1	-0.30	-0.15	0.07	-0.15	0.06	0.93	-0.25	-0.14	-0.02	-0.14	0.05	0.92	-0.31	-0.16	0.02	-0.15	0.06	0.93
10776	ARPP19	-0.33	-0.16	0.07	-0.16	0.06	0.94	-0.27	-0.15	-0.02	-0.15	0.06	0.94	-0.30	-0.17	-0.02	-0.16	0.06	0.91
10783	NEK6	-0.25	-0.13	0.06	-0.13	0.05	0.92	-0.22	-0.14	-0.05	-0.14	0.04	0.97	-0.26	-0.14	-0.02	-0.14	0.05	0.94
10797	MTHFD2	-0.09	0.13	0.28	0.13	0.05	0.93	0.02	0.11	0.23	0.12	0.04	0.95	-0.02	0.13	0.25	0.13	0.05	0.91
10815	CPLX1	-0.15	0.18	0.44	0.19	0.09	0.92	0.01	0.18	0.38	0.19	0.08	0.96	-0.05	0.20	0.38	0.20	0.09	0.94
10867	TSPAN9	-0.09	0.16	0.40	0.16	0.07	0.93	0.04	0.16	0.31	0.16	0.06	0.96	0.02	0.17	0.34	0.17	0.07	0.96
10891	PPARGC1A	-0.13	0.20	0.44	0.20	0.08	0.96	0.04	0.21	0.42	0.21	0.07	0.99	0.02	0.21	0.37	0.21	0.08	0.96
11069	RAPGEF4	-0.40	-0.18	0.06	-0.18	0.07	0.93	-0.35	-0.19	-0.05	-0.20	0.06	0.98	-0.36	-0.19	-0.04	-0.20	0.07	0.97
11118	BTN3A2	-0.04	0.12	0.23	0.12	0.04	0.90	0.04	0.11	0.21	0.11	0.03	0.96	0.01	0.12	0.22	0.12	0.04	0.93
11138	TBC1D8	-0.14	0.17	0.34	0.17	0.07	0.96	0.03	0.17	0.31	0.18	0.06	0.96	0.01	0.16	0.32	0.17	0.06	0.92
11151	CORO1A	-0.35	-0.16	0.10	-0.16	0.06	0.94	-0.30	-0.16	0.01	-0.17	0.06	0.98	-0.30	-0.17	-0.04	-0.17	0.06	0.97
11164	NUDT5	-0.06	0.16	0.35	0.16	0.06	0.94	0.06	0.16	0.29	0.17	0.05	0.97	0.01	0.18	0.31	0.18	0.06	0.97
11167	FSTL1	-0.11	0.18	0.44	0.18	0.08	0.92	0.01	0.19	0.36	0.19	0.07	0.95	0.00	0.20	0.39	0.20	0.08	0.95
11211	FZD10	-0.02	0.12	0.22	0.12	0.03	0.95	0.04	0.11	0.21	0.12	0.03	0.98	0.03	0.13	0.21	0.13	0.04	0.95
11228	RASSF8	-0.11	0.14	0.33	0.15	0.06	0.90	0.00	0.14	0.26	0.14	0.05	0.95	0.02	0.16	0.29	0.16	0.05	0.95
11259	FILIP1L	-0.28	-0.16	0.07	-0.16	0.06	0.96	-0.28	-0.17	-0.03	-0.18	0.05	0.99	-0.31	-0.18	-0.02	-0.17	0.06	0.97
11279	KLF8	-0.38	-0.16	0.08	-0.17	0.06	0.94	-0.29	-0.17	-0.07	-0.17	0.05	0.99	-0.31	-0.18	-0.02	-0.18	0.06	0.96
22801	ITGA11	-0.09	0.22	0.38	0.21	0.07	0.97	0.06	0.23	0.37	0.23	0.06	0.98	0.02	0.22	0.40	0.22	0.07	0.98
22881	ANKRD6	-0.52	-0.20	0.12	-0.20	0.09	0.94	-0.39	-0.21	-0.04	-0.21	0.08	0.98	-0.44	-0.23	0.01	-0.23	0.09	0.97
22987	SV2C	-0.12	0.21	0.44	0.22	0.08	0.97	0.07	0.22	0.44	0.23	0.07	1.00	-0.04	0.23	0.42	0.23	0.08	0.98
22996	TTC39A	-0.11	0.18	0.35	0.18	0.06	0.97	0.09	0.17	0.32	0.18	0.05	1.00	0.00	0.19	0.36	0.19	0.07	0.97
23046	KIF21B	-0.37	-0.17	0.09	-0.17	0.06	0.97	-0.32	-0.18	-0.08	-0.18	0.05	1.00	-0.30	-0.18	-0.03	-0.18	0.06	0.98
23109	DDN	-0.34	-0.17	0.08	-0.16	0.06	0.94	-0.29	-0.17	-0.05	-0.17	0.05	0.98	-0.31	-0.18	-0.03	-0.18	0.06	0.96
23180	RFTN1	-0.31	-0.15	0.09	-0.15	0.06	0.93	-0.26	-0.15	0.00	-0.15	0.05	0.97	-0.28	-0.15	0.02	-0.15	0.06	0.93
23212	RRS1	-0.04	0.14	0.32	0.14	0.05	0.91	0.03	0.13	0.25	0.13	0.05	0.94	0.01	0.14	0.26	0.14	0.05	0.93
23245	ASTN2	-0.13	0.16	0.37	0.16	0.07	0.92	0.04	0.17	0.32	0.17	0.06	0.96	0.00	0.17	0.36	0.18	0.07	0.94
23259	DDHD2	-0.10	0.14	0.35	0.14	0.06	0.91	0.03	0.15	0.29	0.15	0.05	0.96	-0.03	0.15	0.28	0.15	0.06	0.91
23263	MCF2L	-0.10	0.16	0.38	0.15	0.07	0.92	0.01	0.16	0.32	0.16	0.06	0.93	-0.01	0.17	0.32	0.17	0.07	0.94
23274	CLEC16A	-0.11	0.18	0.43	0.18	0.08	0.92	0.01	0.18	0.37	0.18	0.07	0.96	0.00	0.19	0.37	0.19	0.07	0.97
23305	ACSL6	-0.12	0.15	0.37	0.15	0.07	0.92	0.02	0.16	0.32	0.17	0.06	0.97	0.00	0.17	0.33	0.17	0.07	0.96
23327	NEDD4L	-0.29	-0.13	0.04	-0.13	0.05	0.90	-0.25	-0.13	-0.03	-0.14	0.05	0.93	-0.29	-0.15	-0.04	-0.15	0.05	0.95
23370	ARHGEF18	-0.07	0.15	0.35	0.15	0.06	0.90	0.05	0.16	0.32	0.16	0.06	0.96	0.03	0.16	0.31	0.17	0.06	0.94
23406	COTL1	-0.07	0.16	0.28	0.16	0.05	0.96	0.07	0.14	0.27	0.15	0.05	0.99	-0.02	0.16	0.28	0.16	0.05	0.96
23428	SLC7A8	-0.07	0.13	0.30	0.14	0.05	0.93	0.02	0.13	0.26	0.13	0.05	0.93	0.00	0.13	0.26	0.13	0.05	0.91
23460	ABCA6	-0.06	0.16	0.39	0.16	0.07	0.91	0.03	0.16	0.31	0.17	0.06	0.96	0.01	0.17	0.34	0.17	0.07	0.95
23467	NPTXR	-0.29	-0.13	0.13	-0.13	0.06	0.91	-0.26	-0.13	0.01	-0.14	0.05	0.98	-0.29	-0.15	0.02	-0.15	0.05	0.92
23484	LEPROTL1	-0.10	0.18	0.41	0.19	0.07	0.97	0.07	0.19	0.37	0.20	0.06	0.99	0.02	0.19	0.37	0.20	0.07	0.96
23504	RIMBP2	-0.35	-0.17	0.07	-0.17	0.06	0.96	-0.28	-0.16	-0.06	-0.17	0.05	0.99	-0.31	-0.18	-0.03	-0.18	0.06	0.98
23642	SNHG1	-0.13	0.19	0.42	0.19	0.08	0.96	0.03	0.19	0.34	0.19	0.07	0.96	-0.01	0.20	0.39	0.20	0.08	0.95
23780	APOL2	-0.11	0.16	0.41	0.17	0.08	0.91	0.03	0.17	0.34	0.17	0.07	0.94	-0.02	0.18	0.36	0.19	0.08	0.93
24139	EML2	-0.09	0.13	0.29	0.13	0.05	0.93	0.03	0.13	0.24	0.13	0.04	0.94	0.00	0.14	0.25	0.14	0.05	0.93
25769	SLC24A2	-0.11	0.18	0.44	0.18	0.08	0.92	0.06	0.18	0.37	0.19	0.07	0.96	-0.01	0.19	0.37	0.19	0.08	0.94
25818	KLK5	-0.36	-0.16	0.10	-0.16	0.07	0.92	-0.33	-0.16	-0.04	-0.17	0.06	0.96	-0.33	-0.17	-0.01	-0.17	0.07	0.92
25841	ABTB2	-0.11	0.21	0.41	0.21	0.07	0.97	0.09	0.23	0.37	0.22	0.06	1.00	0.03	0.23	0.41	0.23	0.07	1.00
25854	FAM149A	-0.41	-0.17	0.06	-0.17	0.07	0.93	-0.35	-0.19	-0.06	-0.19	0.06	0.99	-0.37	-0.19	-0.03	-0.20	0.07	0.97
25871	NEPRO	-0.34	-0.16	0.09	-0.16	0.06	0.94	-0.29	-0.15	0.00	-0.16	0.06	0.97	-0.29	-0.16	-0.01	-0.16	0.06	0.94
25900	IFFO1	-0.11	0.19	0.46	0.19	0.08	0.94	0.06	0.20	0.38	0.20	0.07	0.98	0.02	0.20	0.38	0.21	0.08	0.95
25907	TMEM158	-0.44	-0.19	0.12	-0.19	0.08	0.94	-0.39	-0.19	-0.03	-0.20	0.07	0.98	-0.39	-0.21	-0.01	-0.22	0.08	0.97
25924	MYRIP	-0.36	-0.15	0.04	-0.15	0.06	0.93	-0.30	-0.17	-0.05	-0.17	0.06	0.95	-0.34	-0.18	-0.06	-0.19	0.06	0.99
25946	ZNF385A	-0.10	0.18	0.40	0.18	0.07	0.93	0.05	0.18	0.35	0.18	0.06	0.97	0.03	0.19	0.35	0.20	0.07	0.98
25953	PNKD	-0.10	0.17	0.38	0.17	0.07	0.95	0.05	0.17	0.35	0.17	0.06	0.95	-0.03	0.18	0.35	0.18	0.07	0.93
25989	ULK3	-0.14	0.20	0.42	0.20	0.07	0.96	0.06	0.21	0.39	0.22	0.07	0.98	0.04	0.21	0.38	0.21	0.07	0.98
26010	SPATS2L	-0.24	-0.13	0.01	-0.13	0.04	0.94	-0.18	-0.12	-0.04	-0.12	0.03	0.93	-0.21	-0.12	0.00	-0.12	0.04	0.91
26031	OSBPL3	-0.09	0.15	0.35	0.16	0.06	0.93	-0.01	0.16	0.33	0.16	0.06	0.99	-0.02	0.17	0.32	0.17	0.06	0.96
26059	ERC2	-0.33	-0.15	0.05	-0.15	0.06	0.92	-0.33	-0.17	-0.02	-0.17	0.06	0.93	-0.35	-0.18	-0.02	-0.18	0.06	0.96
26232	FBXO2	-0.37	-0.18	0.08	-0.18	0.07	0.95	-0.33	-0.19	-0.05	-0.19	0.06	0.98	-0.38	-0.19	0.00	-0.19	0.07	0.95
26996	GPR160	-0.42	-0.18	0.07	-0.18	0.08	0.92	-0.40	-0.20	-0.03	-0.20	0.08	0.97	-0.39	-0.20	0.00	-0.20	0.08	0.96
27077	B9D1	-0.44	-0.18	0.13	-0.19	0.08	0.94	-0.36	-0.18	0.00	-0.19	0.07	0.98	-0.37	-0.19	-0.02	-0.20	0.08	0.95
27132	CPNE7	-0.48	-0.20	0.10	-0.20	0.08	0.96	-0.38	-0.19	-0.07	-0.20	0.07	0.98	-0.40	-0.22	-0.05	-0.22	0.08	0.97
27163	NAAA	-0.15	0.18	0.38	0.18	0.07	0.96	0.02	0.19	0.35	0.19	0.06	0.98	0.01	0.18	0.35	0.19	0.07	0.96
27254	CSDC2	-0.12	0.16	0.39	0.17	0.07	0.92	0.02	0.16	0.33	0.17	0.06	0.95	0.00	0.18	0.34	0.18	0.07	0.95
27294	DHDH	-0.35	-0.15	0.06	-0.16	0.06	0.93	-0.32	-0.17	-0.04	-0.17	0.06	0.99	-0.34	-0.18	0.00	-0.18	0.06	0.96
27345	KCNMB4	-0.45	-0.18	0.09	-0.18	0.08	0.92	-0.38	-0.19	-0.04	-0.19	0.07	0.96	-0.40	-0.20	0.02	-0.20	0.08	0.95
28955	DEXI	-0.10	0.18	0.42	0.18	0.07	0.95	0.04	0.18	0.38	0.19	0.06	0.98	0.00	0.19	0.36	0.19	0.07	0.98
29115	SAP30BP	-0.25	-0.13	0.06	-0.13	0.05	0.91	-0.25	-0.13	-0.03	-0.13	0.04	0.99	-0.23	-0.14	0.00	-0.13	0.04	0.94
29126	CD274	-0.08	0.14	0.34	0.14	0.06	0.91	0.04	0.14	0.28	0.14	0.05	0.96	0.00	0.15	0.29	0.15	0.06	0.90
29799	YPEL1	-0.42	-0.18	0.09	-0.19	0.08	0.94	-0.37	-0.18	-0.04	-0.19	0.07	0.96	-0.40	-0.21	-0.02	-0.21	0.08	0.97
29803	REPIN1	-0.09	0.18	0.39	0.18	0.07	0.96	0.05	0.18	0.35	0.19	0.06	0.99	0.06	0.19	0.34	0.20	0.06	0.98
29844	TFPT	-0.41	-0.18	0.10	-0.18	0.08	0.94	-0.35	-0.20	-0.01	-0.20	0.07	0.96	-0.36	-0.19	0.01	-0.19	0.08	0.92
29902	FAM216A	-0.12	0.16	0.40	0.16	0.07	0.92	0.03	0.15	0.34	0.16	0.06	0.95	-0.02	0.17	0.34	0.17	0.07	0.91
29997	NOP53	-0.13	0.17	0.38	0.16	0.07	0.92	0.03	0.16	0.32	0.17	0.06	0.96	-0.01	0.18	0.37	0.18	0.07	0.92
30850	CDR2L	-0.10	0.20	0.44	0.20	0.08	0.96	0.07	0.22	0.38	0.21	0.07	0.99	0.05	0.22	0.40	0.23	0.08	0.99
49855	SCAPER	-0.29	-0.13	0.06	-0.13	0.06	0.90	-0.28	-0.14	-0.04	-0.15	0.05	0.95	-0.28	-0.15	0.00	-0.15	0.06	0.93
50486	G0S2	-0.06	0.16	0.31	0.17	0.06	0.96	0.05	0.17	0.33	0.17	0.05	0.99	0.02	0.18	0.33	0.18	0.06	0.99
50853	VILL	-0.08	0.16	0.34	0.16	0.06	0.92	0.05	0.17	0.33	0.17	0.06	0.99	0.00	0.18	0.32	0.18	0.06	0.97
51059	FAM135B	-0.09	0.15	0.30	0.15	0.06	0.94	0.08	0.14	0.31	0.15	0.05	1.00	-0.02	0.14	0.30	0.14	0.06	0.91
51074	APIP	-0.08	0.15	0.29	0.15	0.06	0.93	0.05	0.15	0.27	0.15	0.05	0.93	0.02	0.15	0.28	0.15	0.06	0.94
51105	PHF20L1	-0.27	-0.14	0.06	-0.14	0.05	0.96	-0.25	-0.15	-0.02	-0.15	0.04	0.97	-0.26	-0.16	0.00	-0.16	0.05	0.95
51123	ZNF706	-0.40	-0.16	0.11	-0.17	0.07	0.93	-0.32	-0.16	-0.02	-0.17	0.06	0.98	-0.35	-0.18	0.02	-0.18	0.07	0.96
51134	CEP83	-0.29	-0.16	0.05	-0.16	0.06	0.96	-0.30	-0.19	-0.04	-0.18	0.05	0.98	-0.30	-0.17	-0.01	-0.17	0.06	0.96
51155	JPT1	-0.38	-0.16	0.07	-0.16	0.07	0.90	-0.33	-0.16	-0.02	-0.17	0.06	0.97	-0.35	-0.18	-0.03	-0.18	0.07	0.96
51161	C3orf18	-0.12	0.14	0.37	0.15	0.07	0.90	0.02	0.15	0.31	0.16	0.06	0.95	0.00	0.16	0.32	0.16	0.07	0.92
51312	SLC25A37	-0.13	0.20	0.48	0.20	0.09	0.94	0.03	0.21	0.42	0.21	0.08	0.97	-0.02	0.21	0.40	0.22	0.09	0.96
51319	RSRC1	-0.11	0.16	0.42	0.17	0.07	0.93	0.03	0.16	0.32	0.17	0.06	0.96	0.00	0.17	0.33	0.17	0.07	0.91
51334	PRR16	-0.12	0.17	0.35	0.17	0.07	0.95	0.02	0.17	0.31	0.18	0.06	0.95	0.00	0.17	0.36	0.17	0.07	0.94
51375	SNX7	-0.46	-0.21	0.13	-0.20	0.09	0.94	-0.42	-0.22	-0.06	-0.23	0.08	0.98	-0.42	-0.22	-0.03	-0.23	0.08	0.98
51393	TRPV2	-0.31	-0.15	0.05	-0.15	0.06	0.93	-0.28	-0.15	0.01	-0.15	0.05	0.97	-0.31	-0.16	-0.01	-0.16	0.05	0.95
51440	HPCAL4	-0.44	-0.18	0.10	-0.18	0.08	0.93	-0.34	-0.18	-0.03	-0.19	0.07	0.99	-0.37	-0.20	-0.02	-0.20	0.08	0.97
51454	GULP1	-0.39	-0.19	0.12	-0.20	0.08	0.96	-0.35	-0.21	-0.04	-0.21	0.07	0.96	-0.41	-0.21	0.03	-0.21	0.08	0.96
51522	TMEM14C	-0.12	0.17	0.39	0.18	0.07	0.95	0.04	0.16	0.34	0.17	0.06	0.97	-0.02	0.19	0.34	0.19	0.07	0.96
51538	ZCCHC17	-0.44	-0.17	0.08	-0.17	0.08	0.91	-0.34	-0.18	-0.01	-0.18	0.07	0.95	-0.38	-0.19	0.00	-0.20	0.08	0.95
51642	MRPL48	-0.10	0.14	0.31	0.14	0.06	0.93	0.03	0.14	0.27	0.15	0.05	0.97	0.01	0.15	0.30	0.15	0.06	0.91
51660	MPC1	-0.09	0.19	0.41	0.19	0.07	0.96	0.06	0.20	0.36	0.20	0.06	0.99	0.03	0.21	0.36	0.21	0.07	0.98
51667	NUB1	-0.09	0.15	0.34	0.15	0.07	0.93	0.01	0.16	0.31	0.16	0.06	0.93	0.01	0.16	0.31	0.16	0.07	0.93
51705	EMCN	-0.06	0.14	0.33	0.15	0.06	0.93	0.05	0.17	0.29	0.17	0.05	0.96	0.03	0.17	0.29	0.17	0.05	0.95
53616	ADAM22	-0.10	0.15	0.41	0.16	0.07	0.90	0.04	0.17	0.33	0.17	0.06	0.95	-0.01	0.17	0.33	0.17	0.07	0.91
53826	FXYD6	-0.44	-0.17	0.09	-0.17	0.08	0.91	-0.35	-0.16	0.00	-0.17	0.07	0.93	-0.38	-0.18	0.03	-0.19	0.08	0.95
54112	GPR88	-0.39	-0.19	0.11	-0.20	0.07	0.96	-0.37	-0.21	-0.07	-0.21	0.06	0.99	-0.37	-0.21	-0.04	-0.21	0.07	0.98
54492	NEURL1B	-0.35	-0.16	0.05	-0.17	0.07	0.92	-0.33	-0.18	-0.03	-0.18	0.07	0.95	-0.36	-0.18	0.05	-0.18	0.08	0.94
54536	EXOC6	-0.38	-0.19	0.11	-0.19	0.07	0.96	-0.35	-0.18	-0.02	-0.19	0.06	0.98	-0.36	-0.21	-0.05	-0.20	0.07	0.98
54550	NECAB2	-0.47	-0.21	0.16	-0.21	0.09	0.94	-0.38	-0.20	-0.02	-0.21	0.08	0.98	-0.41	-0.22	0.00	-0.23	0.09	0.96

54551	MAGEL2	-0.10	0.17	0.37	0.17	0.07	0.94	0.04	0.17	0.32	0.18	0.06	0.96	-0.01	0.17	0.35	0.18	0.07	0.95
54566	EPB41L4B	-0.32	-0.15	0.09	-0.15	0.06	0.93	-0.29	-0.16	-0.01	-0.16	0.05	0.97	-0.31	-0.17	0.01	-0.17	0.06	0.95
54793	KCTD9	-0.11	0.19	0.42	0.19	0.08	0.95	0.04	0.19	0.40	0.19	0.07	0.97	-0.02	0.20	0.39	0.20	0.08	0.96
54843	SYTL2	-0.33	-0.17	0.08	-0.17	0.06	0.95	-0.30	-0.17	-0.03	-0.18	0.05	0.99	-0.33	-0.19	0.01	-0.18	0.06	0.98
54847	SIDT1	-0.33	-0.17	0.08	-0.17	0.06	0.95	-0.28	-0.16	-0.05	-0.17	0.05	0.97	-0.32	-0.18	-0.03	-0.18	0.06	0.96
54874	FNBP1L	-0.42	-0.19	0.11	-0.18	0.08	0.93	-0.35	-0.20	-0.04	-0.20	0.07	0.98	-0.38	-0.21	-0.02	-0.21	0.08	0.98
55022	PID1	-0.46	-0.17	0.11	-0.17	0.09	0.90	-0.37	-0.17	0.01	-0.18	0.08	0.93	-0.40	-0.20	0.04	-0.20	0.08	0.96
55040	EPN3	-0.10	0.19	0.43	0.20	0.08	0.96	0.06	0.21	0.41	0.21	0.07	0.99	-0.01	0.21	0.40	0.21	0.08	0.96
55086	CXorf57	-0.48	-0.20	0.15	-0.20	0.09	0.94	-0.38	-0.20	-0.04	-0.20	0.08	0.97	-0.41	-0.21	0.02	-0.22	0.09	0.95
55118	CRTAC1	-0.10	0.16	0.34	0.16	0.06	0.94	0.08	0.17	0.30	0.17	0.05	1.00	0.03	0.18	0.33	0.17	0.06	0.96
55122	AKIRIN2	-0.35	-0.16	0.08	-0.16	0.07	0.91	-0.31	-0.17	0.00	-0.17	0.06	0.98	-0.32	-0.18	-0.01	-0.18	0.07	0.96
55160	ARHGEF10L	-0.10	0.17	0.38	0.17	0.07	0.95	0.00	0.17	0.32	0.17	0.06	0.95	0.01	0.18	0.33	0.18	0.07	0.94
55170	PRMT6	-0.29	-0.15	0.06	-0.15	0.05	0.95	-0.28	-0.18	-0.06	-0.17	0.05	0.99	-0.27	-0.17	-0.04	-0.17	0.05	0.98
55190	NUDT11	-0.45	-0.17	0.10	-0.17	0.08	0.91	-0.35	-0.17	-0.01	-0.17	0.08	0.92	-0.39	-0.19	0.01	-0.19	0.08	0.94
55208	DCUN1D2	-0.11	0.17	0.41	0.17	0.07	0.93	0.04	0.17	0.35	0.18	0.06	0.95	0.00	0.17	0.34	0.18	0.08	0.92
55225	RAVER2	-0.35	-0.15	0.11	-0.15	0.07	0.91	-0.28	-0.15	-0.02	-0.15	0.06	0.94	-0.31	-0.15	-0.01	-0.16	0.07	0.90
55244	SLC47A1	-0.13	0.25	0.46	0.25	0.08	0.98	0.11	0.26	0.45	0.26	0.07	1.00	0.05	0.25	0.44	0.26	0.08	1.00
55282	LRRC36	-0.42	-0.17	0.07	-0.16	0.08	0.90	-0.34	-0.19	-0.04	-0.18	0.07	0.92	-0.35	-0.19	0.02	-0.19	0.08	0.94
55315	SLC29A3	-0.33	-0.18	0.08	-0.18	0.06	0.97	-0.29	-0.18	-0.04	-0.18	0.05	0.99	-0.31	-0.19	-0.03	-0.19	0.06	0.97
55353	LAPTM4B	-0.10	0.18	0.42	0.18	0.08	0.94	0.07	0.18	0.36	0.19	0.07	0.99	0.02	0.20	0.37	0.20	0.07	0.96
55359	STYK1	-0.32	-0.14	0.05	-0.14	0.06	0.91	-0.25	-0.14	0.03	-0.14	0.05	0.95	-0.30	-0.16	-0.03	-0.15	0.06	0.92
55502	HES6	-0.09	0.16	0.38	0.16	0.07	0.91	0.02	0.16	0.34	0.17	0.06	0.96	0.00	0.18	0.34	0.18	0.07	0.95
55509	BATF3	-0.44	-0.20	0.11	-0.19	0.08	0.95	-0.39	-0.20	-0.03	-0.21	0.07	0.97	-0.39	-0.21	0.00	-0.21	0.08	0.98
55591	VEZT	-0.34	-0.17	0.09	-0.17	0.06	0.96	-0.30	-0.17	-0.05	-0.17	0.05	0.96	-0.32	-0.18	0.00	-0.18	0.07	0.94
55686	MREG	-0.08	0.17	0.34	0.17	0.06	0.95	0.07	0.17	0.33	0.17	0.05	0.99	0.02	0.17	0.34	0.17	0.06	0.95
55714	TENM3	-0.37	-0.15	0.04	-0.15	0.06	0.93	-0.31	-0.15	-0.04	-0.15	0.06	0.94	-0.33	-0.17	0.00	-0.17	0.06	0.95
55800	SCN3B	-0.49	-0.22	0.13	-0.22	0.09	0.98	-0.42	-0.22	-0.05	-0.23	0.08	0.98	-0.42	-0.24	-0.04	-0.24	0.09	0.98
55853	IDI2-AS1	-0.13	0.19	0.40	0.19	0.07	0.97	0.09	0.18	0.37	0.19	0.06	1.00	-0.03	0.20	0.37	0.20	0.07	0.98
55884	WSB2	-0.29	-0.14	0.07	-0.15	0.06	0.93	-0.26	-0.15	-0.02	-0.14	0.05	0.95	-0.29	-0.16	0.01	-0.16	0.06	0.95
55897	MESP1	-0.45	-0.20	0.11	-0.20	0.07	0.97	-0.37	-0.20	-0.03	-0.21	0.07	0.99	-0.37	-0.21	-0.04	-0.22	0.08	0.98
56172	ANKH	-0.12	0.20	0.47	0.20	0.08	0.94	0.05	0.21	0.39	0.21	0.07	0.98	0.01	0.20	0.37	0.21	0.08	0.97
56477	CCL28	-0.28	-0.14	0.04	-0.14	0.05	0.93	-0.25	-0.14	-0.07	-0.15	0.04	0.97	-0.26	-0.16	-0.03	-0.16	0.05	0.97
56648	EIF5A2	-0.12	0.19	0.46	0.19	0.09	0.93	0.03	0.19	0.38	0.20	0.07	0.98	-0.02	0.20	0.38	0.21	0.08	0.96
56666	PANX2	-0.16	0.17	0.42	0.17	0.08	0.91	0.00	0.17	0.35	0.17	0.07	0.93	-0.03	0.18	0.36	0.18	0.08	0.91
56848	SPHK2	-0.13	0.14	0.36	0.15	0.07	0.90	-0.01	0.14	0.33	0.15	0.06	0.91	-0.04	0.16	0.32	0.16	0.07	0.92
56884	FSTL5	-0.37	-0.15	0.09	-0.15	0.06	0.93	-0.28	-0.16	-0.03	-0.17	0.06	0.94	-0.30	-0.16	0.04	-0.16	0.07	0.90
56906	THAP10	-0.13	0.18	0.46	0.18	0.08	0.93	0.03	0.18	0.38	0.18	0.07	0.97	-0.01	0.19	0.37	0.19	0.08	0.92
56927	GPR108	-0.13	0.14	0.33	0.14	0.06	0.91	0.02	0.13	0.25	0.14	0.05	0.90	-0.03	0.15	0.29	0.15	0.06	0.90
56934	CA10	-0.39	-0.19	0.13	-0.20	0.08	0.96	-0.34	-0.20	-0.03	-0.20	0.07	0.97	-0.42	-0.21	0.05	-0.20	0.08	0.94
56937	PMEPA1	-0.11	0.19	0.40	0.20	0.07	0.96	0.05	0.21	0.36	0.22	0.06	0.99	0.06	0.21	0.37	0.21	0.07	0.99
56967	C14orf132	-0.32	-0.16	0.07	-0.16	0.06	0.94	-0.28	-0.15	-0.07	-0.16	0.04	1.00	-0.29	-0.16	0.01	-0.16	0.05	0.96
56971	CEACAM19	-0.09	0.15	0.34	0.15	0.06	0.93	0.03	0.16	0.32	0.17	0.06	0.96	0.00	0.16	0.34	0.16	0.06	0.93
57110	HRASLS	-0.35	-0.16	0.10	-0.15	0.07	0.91	-0.33	-0.18	-0.05	-0.18	0.06	0.98	-0.32	-0.19	-0.01	-0.18	0.07	0.95
57194	ATP10A	-0.08	0.16	0.32	0.16	0.06	0.95	0.05	0.18	0.30	0.18	0.05	0.99	0.03	0.18	0.30	0.18	0.05	0.98
57406	ABHD6	-0.08	0.15	0.31	0.15	0.06	0.92	0.04	0.16	0.27	0.16	0.05	0.96	0.03	0.16	0.32	0.17	0.06	0.95
57453	DSCAML1	-0.09	0.16	0.30	0.16	0.06	0.94	0.05	0.18	0.30	0.18	0.05	0.98	0.04	0.17	0.30	0.17	0.06	0.97
57465	TBC1D24	-0.39	-0.17	0.09	-0.17	0.07	0.94	-0.28	-0.17	-0.04	-0.17	0.06	0.97	-0.34	-0.19	-0.02	-0.19	0.07	0.98
57484	RNF150	-0.42	-0.17	0.09	-0.17	0.08	0.92	-0.33	-0.18	-0.01	-0.18	0.07	0.98	-0.38	-0.19	0.00	-0.19	0.08	0.94
57495	NWD2	-0.38	-0.21	0.11	-0.21	0.07	0.99	-0.36	-0.20	-0.09	-0.21	0.06	1.00	-0.37	-0.23	-0.03	-0.22	0.07	0.99
57496	MKL2	-0.38	-0.20	0.12	-0.20	0.07	0.98	-0.33	-0.18	-0.05	-0.19	0.06	0.98	-0.38	-0.21	-0.01	-0.20	0.07	0.97
57519	STARD9	-0.06	0.17	0.35	0.16	0.06	0.92	0.05	0.19	0.35	0.19	0.06	0.96	0.03	0.18	0.33	0.18	0.07	0.96
57526	PCDH19	-0.43	-0.22	0.12	-0.22	0.07	0.98	-0.37	-0.22	-0.05	-0.23	0.06	0.99	-0.40	-0.23	-0.07	-0.23	0.07	1.00
57596	BEGAIN	-0.28	-0.14	0.06	-0.14	0.05	0.91	-0.24	-0.14	-0.04	-0.14	0.04	0.97	-0.28	-0.15	-0.01	-0.15	0.05	0.94
57630	SH3RF1	-0.39	-0.17	0.09	-0.16	0.07	0.91	-0.34	-0.17	-0.05	-0.18	0.07	0.96	-0.36	-0.18	-0.02	-0.19	0.07	0.97
57631	LRCH2	-0.29	-0.13	0.10	-0.13	0.05	0.91	-0.26	-0.14	-0.04	-0.14	0.04	0.94	-0.28	-0.14	0.01	-0.14	0.05	0.93
57644	MYH7B	-0.08	0.17	0.42	0.17	0.08	0.92	0.02	0.17	0.34	0.18	0.07	0.97	0.00	0.19	0.36	0.19	0.08	0.95
57699	CPNE5	-0.35	-0.16	0.05	-0.16	0.06	0.93	-0.28	-0.16	-0.04	-0.16	0.05	0.96	-0.31	-0.18	-0.03	-0.17	0.06	0.97
57718	PPP4R4	-0.47	-0.20	0.12	-0.20	0.09	0.94	-0.40	-0.20	-0.04	-0.21	0.08	0.98	-0.41	-0.22	0.00	-0.22	0.09	0.97
57761	TRIB3	-0.26	-0.14	0.05	-0.14	0.05	0.95	-0.23	-0.14	-0.06	-0.14	0.05	0.98	-0.28	-0.16	-0.03	-0.16	0.05	0.97
60626	RIC8A	-0.37	-0.15	0.09	-0.15	0.07	0.91	-0.30	-0.16	-0.05	-0.16	0.06	0.96	-0.35	-0.18	-0.01	-0.18	0.07	0.95
63941	NECAB3	-0.10	0.16	0.39	0.16	0.07	0.93	0.02	0.16	0.29	0.16	0.06	0.95	-0.03	0.16	0.37	0.17	0.08	0.91
63974	NEUROD6	-0.36	-0.18	0.06	-0.18	0.07	0.95	-0.33	-0.19	-0.05	-0.20	0.06	0.99	-0.38	-0.21	0.00	-0.20	0.07	0.97
63982	ANO3	-0.44	-0.19	0.12	-0.19	0.08	0.94	-0.38	-0.18	-0.03	-0.20	0.07	0.97	-0.42	-0.20	0.02	-0.20	0.09	0.94
64131	XYLT1	-0.45	-0.19	0.12	-0.19	0.08	0.94	-0.35	-0.20	-0.05	-0.20	0.07	0.98	-0.40	-0.21	0.01	-0.21	0.08	0.96
64132	XYLT2	-0.12	0.18	0.39	0.18	0.07	0.96	0.00	0.18	0.37	0.19	0.07	0.97	0.01	0.20	0.35	0.20	0.07	0.97
64135	IFIH1	-0.06	0.15	0.29	0.15	0.05	0.94	0.06	0.16	0.26	0.16	0.05	0.97	0.01	0.17	0.30	0.17	0.05	0.97
64137	ABCG4	-0.13	0.16	0.38	0.16	0.07	0.92	0.03	0.16	0.31	0.16	0.06	0.98	0.01	0.17	0.34	0.17	0.07	0.94
64149	C17orf75	-0.14	0.16	0.34	0.16	0.06	0.95	0.03	0.16	0.33	0.16	0.06	0.98	0.00	0.17	0.31	0.17	0.07	0.91
64150	DIO3OS	-0.06	0.13	0.27	0.13	0.05	0.94	0.04	0.14	0.31	0.15	0.05	0.96	0.02	0.16	0.27	0.15	0.05	0.96
64333	ARHGAP9	-0.13	0.18	0.45	0.18	0.08	0.93	0.05	0.18	0.37	0.19	0.07	0.95	-0.02	0.20	0.40	0.20	0.08	0.94
64792	IFT22	-0.41	-0.18	0.09	-0.17	0.08	0.92	-0.38	-0.20	-0.05	-0.20	0.07	0.97	-0.38	-0.20	0.00	-0.20	0.08	0.96
64881	PCDH20	-0.44	-0.18	0.14	-0.18	0.08	0.93	-0.35	-0.17	-0.03	-0.18	0.08	0.93	-0.38	-0.20	0.02	-0.20	0.09	0.93
65078	RTN4R	-0.29	-0.14	0.03	-0.14	0.04	0.96	-0.22	-0.13	-0.05	-0.14	0.04	0.99	-0.24	-0.14	0.00	-0.14	0.04	0.95
65110	UPF3A	-0.06	0.15	0.31	0.15	0.06	0.91	0.05	0.16	0.32	0.17	0.06	0.96	-0.01	0.17	0.33	0.17	0.06	0.95
65263	PYCR3	-0.33	-0.15	0.08	-0.15	0.06	0.91	-0.29	-0.15	-0.02	-0.15	0.06	0.95	-0.29	-0.16	-0.01	-0.16	0.06	0.93
65982	ZSCAN18	-0.43	-0.16	0.11	-0.16	0.07	0.90	-0.32	-0.15	0.01	-0.16	0.07	0.92	-0.37	-0.18	0.02	-0.18	0.07	0.95
65997	RASL11B	-0.39	-0.20	0.11	-0.20	0.07	0.97	-0.33	-0.20	-0.03	-0.20	0.06	0.99	-0.38	-0.22	-0.04	-0.22	0.07	0.98
66000	TMEM108	-0.39	-0.17	0.14	-0.18	0.07	0.96	-0.32	-0.17	-0.06	-0.18	0.06	0.98	-0.34	-0.19	-0.02	-0.19	0.07	0.96
66008	TRAK2	-0.12	0.17	0.36	0.17	0.06	0.97	0.07	0.17	0.29	0.17	0.05	0.98	0.02	0.17	0.32	0.18	0.07	0.95
78990	OTUB2	-0.27	-0.13	0.07	-0.13	0.05	0.93	-0.23	-0.13	-0.02	-0.13	0.04	0.95	-0.25	-0.14	-0.01	-0.14	0.05	0.92
79012	CAMKV	-0.37	-0.18	0.09	-0.18	0.07	0.96	-0.34	-0.17	0.00	-0.18	0.06	0.97	-0.36	-0.19	-0.02	-0.20	0.07	0.97
79017	GGCT	-0.42	-0.19	0.09	-0.19	0.08	0.95	-0.37	-0.19	-0.06	-0.20	0.07	0.96	-0.38	-0.20	-0.02	-0.20	0.08	0.96
79085	SLC25A23	-0.34	-0.15	0.07	-0.15	0.06	0.93	-0.25	-0.14	-0.02	-0.14	0.05	0.96	-0.32	-0.15	-0.01	-0.15	0.06	0.91
79183	TTPAL	-0.30	-0.15	0.11	-0.15	0.06	0.93	-0.30	-0.16	-0.01	-0.17	0.05	0.95	-0.31	-0.17	0.01	-0.17	0.06	0.96
79442	LRRC2	-0.42	-0.18	0.09	-0.18	0.08	0.94	-0.32	-0.17	-0.01	-0.18	0.07	0.94	-0.40	-0.19	0.05	-0.19	0.08	0.93
79585	CORO7	-0.08	0.16	0.37	0.16	0.07	0.92	0.05	0.16	0.31	0.17	0.06	0.96	0.02	0.17	0.34	0.18	0.07	0.96
79645	EFCAB1	-0.50	-0.21	0.13	-0.21	0.09	0.95	-0.41	-0.21	-0.05	-0.22	0.08	0.97	-0.42	-0.23	0.00	-0.23	0.09	0.97
79660	PPP1R3B	-0.02	0.16	0.32	0.17	0.05	0.97	0.07	0.17	0.28	0.17	0.04	0.99	0.04	0.18	0.29	0.17	0.05	0.99
79720	VPS37B	-0.27	-0.14	0.06	-0.14	0.05	0.94	-0.29	-0.13	-0.04	-0.14	0.05	0.98	-0.25	-0.14	0.02	-0.14	0.05	0.92
79745	CLIP4	-0.10	0.16	0.31	0.16	0.06	0.96	0.04	0.16	0.28	0.16	0.05	0.98	0.03	0.17	0.28	0.16	0.06	0.94
79750	ZNF385D	-0.13	0.17	0.34	0.17	0.06	0.95	0.04	0.16	0.33	0.17	0.05	0.98	-0.01	0.17	0.31	0.17	0.06	0.95
79754	ASB13	-0.11	0.19	0.47	0.20	0.09	0.94	0.07	0.21	0.40	0.21	0.07	0.99	0.03	0.21	0.40	0.22	0.08	0.97
79822	ARHGAP28	-0.39	-0.19	0.12	-0.19	0.07	0.96	-0.34	-0.19	-0.06	-0.19	0.06	0.98	-0.36	-0.19	-0.01	-0.20	0.08	0.95
79874	RABEP2	-0.11	0.17	0.35	0.17	0.06	0.96	0.07	0.17	0.30	0.18	0.05	0.99	0.04	0.18	0.34	0.18	0.06	0.97
79884	MAP9	-0.12	0.15	0.40	0.16	0.07	0.90	0.03	0.16	0.33	0.16	0.06	0.96	-0.01	0.17	0.35	0.17	0.07	0.93
79930	DOK3	-0.10	0.16	0.37	0.17	0.07	0.92	0.01	0.16	0.32	0.16	0.06	0.96	-0.02	0.18	0.35	0.18	0.07	0.96
79957	PAQR6	-0.01	0.12	0.20	0.12	0.04	0.93	0.03	0.12	0.21	0.12	0.04	0.90	0.00	0.12	0.23	0.12	0.04	0.91
79958	DENND1C	-0.26	-0.13	0.07	-0.13	0.05	0.91	-0.25	-0.14	0.01	-0.14	0.04	0.95	-0.29	-0.14	0.00	-0.13	0.05	0.91
79993	ELOVL7	-0.07	0.15	0.30	0.15	0.05	0.94	0.03	0.16	0.28	0.16	0.04	0.98	0.05	0.16	0.28	0.16	0.05	0.96
80020	FOXRED2	-0.38	-0.21	0.11	-0.21	0.07	0.98	-0.35	-0.21	-0.05	-0.22	0.06	0.99	-0.38	-0.23	-0.05	-0.22	0.07	0.99
80036	TRPM3	-0.14	0.19	0.43	0.20	0.08	0.95	0.04	0.20	0.37	0.20	0.07	0.98	0.01	0.21	0.39	0.21	0.08	0.98
80119	PIF1	-0.12	0.22	0.49	0.22	0.08	0.96	0.07	0.23	0.42	0.23	0.07	0.99	0.04	0.24	0.41	0.24	0.08	0.99
80176	SPSB1	-0.06	0.15	0.28	0.15	0.05	0.94	0.05	0.16	0.28	0.17	0.05	0.99	0.04	0.17	0.32	0.17	0.06	0.97
80179	MYO19	-0.11	0.18	0.42	0.18	0.08	0.92	0.05	0.19	0.39	0.20	0.07	0.96	-0.01	0.19	0.39	0.20	0.08	0.95
80307	FER1L4	-0.15	0.23	0.47	0.23	0.08	0.98	0.06	0.25	0.42	0.25	0.07	0.99	0.05	0.24	0.42	0.25	0.08	0.99
80323	CCDC68	-0.36	-0.17	0.10	-0.18	0.07	0.96	-0.32	-0.19	-0.04	-0.19	0.06	0.98	-0.35	-0.20	-0.01	-0.20	0.07	0.97
80774	LIMD2	-0.26	-0.14	0.00	-0.13	0.04	0.95	-0.24	-0.15	-0.02	-0.15	0.04	0.98	-0.26	-0.14	-0.01	-0.14	0.05	0.93
80816	ASXL3	-0.31	-0.14	0.09	-0.14	0.06	0.92	-0.25	-0.13	0.00	-0.13	0.05	0.92	-0.28	-0.15	0.04	-0.15	0.06	0.90
80818	ZNF436	-0.33	-0.15	0.07	-0.15	0.06	0.92	-0.31	-0.15	0.00	-0.16	0.06	0.95	-0.32	-0.17	0.00	-0.17	0.06	0.94
80820	EEPD1	-0.13	0.22	0.49	0.21	0.09	0.96	0.06	0.22	0.44	0.23	0.08	0.99	0.01	0.23	0.44	0.23	0.09	0.97
80854	SETD7	-0.12	0.18	0.39	0.19	0.07	0.96	0.06	0.18	0.33	0.18	0.06	0.98	0.03	0.19	0.35	0.19	0.07	0.95
81033	KCNH6	-0.15	0.19	0.42	0.19	0.08	0.96	0.06	0.19	0.37	0.20	0.07	0.99	0.01	0.20	0.39	0.20	0.08	0.96
81539	SLC38A1	-0.12	0.15	0.36	0.15	0.07	0.92	0.00	0.16	0.31	0.16	0.06	0.93	0.00	0.15	0.29	0.16	0.06	0.93
81552	VOPP1	-0.32	-0.15	0.09	-0.15	0.06	0.92	-0.29	-0.16	-0.02	-0.16	0.05	0.95	-0.31	-0.16	0.00	-0.16	0.06	0.94
81602	CDADC1	-0.10	0.15	0.38	0.16	0.07	0.92	0.03	0.15	0.34	0.16	0.06	0.95	0.00	0.16	0.31	0.17	0.07	0.94
81605	URM1	-0.10	0.18	0.45	0.18	0.09	0.90	0.00	0.19	0.40	0.19	0.08	0.97	-0.02	0.20	0.40	0.20	0.08	0.95
81849	ST6GALNAC5	-0.46	-0.23	0.12	-0.23	0.08	0.98	-0.38	-0.24	-0.09	-0.24	0.07	1.00	-0.43	-0.25	-0.03	-0.25	0.08	0.99
83445	GSG1	-0.44	-0.19	0.12	-0.19	0.08	0.96	-0.36	-0.20	-0.06	-0.20	0.07	0.99	-0.39	-0.21	0.00	-0.21	0.09	0.95
83468	GLT8D2	-0.34	-0.18	0.05	-0.18	0.06	0.96	-0.34	-0.18	-0.08	-0.19	0.05	1.00	-0.34	-0.20	-0.03	-0.20	0.06	0.99
83482	SCRT1	-0.13	0.19	0.48	0.20	0.09	0.94	0.03	0.20	0.40	0.20	0.08	0.97	-0.01	0.21	0.40	0.22	0.08	0.96
83546	RTBDN	-0.29	-0.13	0.09	-0.13	0.05	0.91	-0.25	-0.14	-0.03	-0.14	0.05	0.95	-0.26	-0.15	-0.02	-0.15	0.05	0.94
83547	RILP	-0.11	0.17	0.38	0.17	0.07	0.92	0.05	0.16	0.36	0.18	0.06	0.97	0.00	0.19	0.37	0.19	0.07	0.96
83660	TLN2	-0.39	-0.20	0.08	-0.20	0.07	0.97	-0.33	-0.19	-0.07	-0.20	0.05	0.99	-0.35	-0.22	-0.06	-0.21	0.06	0.99
83690	CRISPLD1	-0.13	0.15	0.31	0.15	0.06	0.92	-0.05	0.14	0.26	0.15	0.05	0.94	-0.01	0.16	0.29	0.16	0.06	0.93
83692	CD99L2	-0.12	0.16	0.41	0.17	0.08	0.92	0.04	0.19	0.35	0.18	0.07	0.96	-0.01	0.18	0.36	0.18	0.08	0.94
83707	TRPT1	-0.12	0.14	0.33	0.15	0.06	0.91	0.04	0.13	0.28	0.14	0.05	0.91	0.00	0.16	0.31	0.16	0.06	0.94
83714	NRIP2	-0.11	0.15	0.29	0.15	0.06	0.95	0.06	0.15	0.25	0.15	0.05	0.97	0.02	0.16	0.29	0.16	0.06	0.93
83723	FAM57B	-0.14	0.15	0.34	0.15	0.06	0.94	0.05	0.15	0.31	0.15	0.05	0.96	-0.01	0.16	0.32	0.16	0.06	0.93
83787	ARMC10	-0.35	-0.15	0.03	-0.15	0.06	0.90	-0.30	-0.16	-0.01	-0.16	0.06	0.96	-0.32	-0.17	-0.02	-0.17	0.06	0.96
83875	BCO2	-0.14	0.16	0.34	0.16	0.07	0.93	0.02	0.15	0.29	0.15	0.06	0.93	-0.04	0.16	0.33	0.16	0.07	0.93
83937	RASSF4	-0.12	0.17	0.43	0.18	0.08	0.93	0.03	0.18	0.37	0.18	0.07	0.95	-0.01	0.19	0.37	0.19	0.08	0.95
83992	CTTNBP2	-0.10	0.15	0.35	0.15	0.06	0.91	0.01	0.15	0.29	0.16	0.06	0.95	-0.01	0.16	0.30	0.16	0.06	0.94
84034	EMILIN2	-0.09	0.21	0.34	0.20	0.06	0.98	0.11	0.22	0.32	0.22	0.05	1.00	0.05	0.22	0.36	0.22	0.06	1.00
84063	KIRREL2	-0.47	-0.19	0.11	-0.19	0.09	0.93	-0.40	-0.20	0.01	-0.20	0.08	0.97	-0.41	-0.21	0.02	-0.21	0.09	0.95
84083	ZRANB3	-0.30	-0.13	0.06	-0.14	0.06	0.91	-0.26	-0.13	-0.01	-0.14	0.05	0.91	-0.28	-0.15	0.01	-0.15	0.05	0.93
84109	QRFPR	-0.08	0.19	0.40	0.20	0.07	0.97	0.04	0.21	0.37	0.21	0.06	0.98	-0.03	0.20	0.41	0.20	0.08	0.95
84221	SPATC1L	-0.36	-0.17	0.08	-0.17	0.06	0.94	-0.30	-0.17	-0.05	-0.18	0.05	0.98	-0.30	-0.18	-0.04	-0.18	0.06	0.98
84314	TMEM107	-0.28	-0.14	0.08	-0.14	0.05	0.93	-0.25	-0.14	-0.04	-0.15	0.04	0.99	-0.26	-0.15	-0.01	-0.15	0.05	0.95
84332	DYDC2	-0.46	-0.18	0.08	-0.18	0.09	0.91	-0.40	-0.20	-0.03	-0.20	0.08	0.97	-0.41	-0.21	0.00	-0.21	0.08	0.96
84439	HHIPL1	-0.38	-0.18	0.12	-0.18	0.07	0.96	-0.34	-0.20	-0.06	-0.19	0.06	0.98	-0.35	-0.18	-0.02	-0.18	0.08	0.92
84524	ZC3H8	-0.20	-0.12	0.00	-0.12	0.04	0.93	-0.23	-0.13	-0.03	-0.13	0.03	0.97	-0.22	-0.12	-0.02	-0.12	0.04	0.94
84525	HOPX	-0.30	-0.16	0.05	-0.16	0.05	0.95	-0.26	-0.16	-0.04	-0.16	0.04	0.98	-0.30	-0.17	-0.05	-0.17	0.05	0.98
84542	KIAA1841	-0.11	0.15	0.34	0.15	0.07	0.91	-0.01	0.16	0.32	0.16	0.06	0.94	-0.02	0.17	0.32	0.17	0.06	0.96
84623	KIRREL3	-0.36	-0.18	0.12	-0.18	0.07	0.95	-0.29	-0.17	-0.04	-0.18	0.05	0.98	-0.35	-0.19	-0.01	-0.19	0.06	0.97
84691	FAM71F1	-0.44	-0.17	0.10	-0.17	0.08	0.91	-0.34	-0.17	-0.01	-0.17	0.07	0.96	-0.38	-0.19	0.01	-0.19	0.08	0.95

84709	MGARP	-0.11	0.14	0.34	0.14	0.06	0.91	0.02	0.14	0.29	0.14	0.06	0.91	-0.02	0.15	0.32	0.16	0.07	0.91
84769	MPV17L2	-0.34	-0.17	0.08	-0.16	0.06	0.95	-0.32	-0.16	-0.03	-0.17	0.06	0.98	-0.31	-0.18	-0.02	-0.17	0.06	0.93
84803	GPAT3	-0.13	0.19	0.49	0.20	0.09	0.94	0.06	0.21	0.38	0.21	0.08	0.98	-0.01	0.21	0.42	0.22	0.09	0.95
84812	PLCD4	-0.11	0.19	0.44	0.20	0.08	0.96	0.07	0.20	0.36	0.20	0.07	0.96	0.03	0.21	0.40	0.21	0.08	0.98
84864	RIOX2	-0.42	-0.17	0.06	-0.17	0.07	0.92	-0.30	-0.16	-0.03	-0.17	0.06	0.96	-0.33	-0.17	0.00	-0.17	0.07	0.94
84870	RSPO3	-0.36	-0.14	0.08	-0.14	0.06	0.90	-0.29	-0.15	-0.03	-0.16	0.06	0.92	-0.33	-0.17	-0.05	-0.18	0.06	0.98
84936	ZFYVE19	-0.10	0.18	0.40	0.18	0.07	0.94	0.02	0.17	0.34	0.18	0.06	0.98	0.03	0.20	0.36	0.19	0.07	0.95
84937	ZNRF1	-0.32	-0.17	0.02	-0.16	0.05	0.94	-0.30	-0.18	-0.08	-0.18	0.05	1.00	-0.30	-0.19	-0.06	-0.19	0.05	0.99
84957	RELT	-0.10	0.15	0.34	0.15	0.06	0.90	0.03	0.15	0.27	0.15	0.05	0.95	0.00	0.16	0.30	0.16	0.07	0.91
85015	USP45	-0.13	0.16	0.36	0.16	0.07	0.91	-0.03	0.16	0.35	0.16	0.07	0.94	-0.02	0.18	0.34	0.17	0.07	0.94
85352	SHISAL1	-0.37	-0.17	0.08	-0.17	0.07	0.94	-0.33	-0.18	-0.07	-0.19	0.06	0.99	-0.33	-0.19	-0.03	-0.19	0.07	0.97
85461	TANC1	-0.07	0.15	0.29	0.15	0.05	0.95	0.05	0.15	0.25	0.15	0.04	0.98	0.03	0.16	0.27	0.16	0.05	0.96
89782	LMLN	-0.11	0.16	0.38	0.16	0.07	0.92	0.03	0.16	0.33	0.16	0.06	0.96	0.00	0.17	0.34	0.17	0.07	0.95
89846	FGD3	-0.32	-0.15	0.10	-0.15	0.06	0.91	-0.32	-0.17	-0.04	-0.17	0.06	0.96	-0.31	-0.17	-0.01	-0.17	0.06	0.95
90102	PHLDB2	-0.04	0.15	0.29	0.15	0.05	0.93	0.01	0.16	0.29	0.17	0.05	0.99	0.02	0.16	0.29	0.16	0.05	0.95
90488	TMEM263	-0.43	-0.17	0.10	-0.18	0.09	0.91	-0.39	-0.19	-0.02	-0.19	0.08	0.95	-0.41	-0.20	0.01	-0.20	0.08	0.97
90523	MLIP	-0.31	-0.18	0.07	-0.17	0.05	0.98	-0.27	-0.17	-0.09	-0.18	0.04	1.00	-0.30	-0.19	0.00	-0.19	0.05	0.99
90850	ZNF598	-0.05	0.14	0.28	0.14	0.05	0.91	0.04	0.16	0.28	0.15	0.05	0.96	0.03	0.15	0.26	0.15	0.05	0.94
90861	JPT2	0.00	0.14	0.23	0.14	0.04	0.97	0.06	0.16	0.23	0.16	0.04	1.00	0.01	0.15	0.25	0.15	0.04	0.98
90865	IL33	-0.39	-0.16	0.10	-0.16	0.08	0.90	-0.34	-0.17	-0.02	-0.17	0.06	0.97	-0.35	-0.17	0.02	-0.17	0.08	0.92
91133	L3MBTL4	-0.31	-0.16	0.09	-0.16	0.06	0.95	-0.28	-0.18	-0.02	-0.18	0.05	0.98	-0.30	-0.17	-0.03	-0.17	0.06	0.95
91252	SLC39A13	-0.13	0.20	0.45	0.21	0.08	0.96	0.05	0.21	0.40	0.22	0.07	0.99	0.04	0.22	0.40	0.23	0.08	0.99
91624	NEXN	-0.12	0.19	0.40	0.19	0.08	0.95	0.06	0.19	0.33	0.19	0.07	0.96	-0.01	0.21	0.36	0.20	0.07	0.97
91683	SYT12	-0.13	0.17	0.39	0.17	0.08	0.92	0.04	0.18	0.35	0.18	0.07	0.97	-0.01	0.17	0.34	0.18	0.07	0.94
92017	SNX29	-0.02	0.11	0.19	0.11	0.04	0.91	0.02	0.12	0.19	0.11	0.04	0.92	0.02	0.12	0.22	0.12	0.04	0.92
92293	TMEM132C	-0.10	0.13	0.31	0.14	0.06	0.92	0.04	0.13	0.30	0.13	0.05	0.91	-0.03	0.14	0.28	0.15	0.06	0.92
92335	STRADA	-0.07	0.15	0.33	0.15	0.06	0.92	0.01	0.16	0.29	0.16	0.05	0.96	0.02	0.16	0.30	0.16	0.06	0.95
92399	MRRF	-0.13	0.16	0.36	0.16	0.07	0.93	0.06	0.16	0.30	0.16	0.06	0.96	-0.01	0.16	0.35	0.17	0.07	0.91
92597	MOB1B	-0.39	-0.18	0.12	-0.17	0.07	0.93	-0.35	-0.19	-0.03	-0.19	0.06	0.98	-0.36	-0.20	-0.03	-0.20	0.07	0.96
92610	TIFA	-0.15	0.18	0.42	0.18	0.08	0.93	0.04	0.17	0.33	0.18	0.07	0.95	-0.02	0.18	0.36	0.18	0.08	0.90
94160	ABCC12	-0.29	-0.15	0.08	-0.15	0.06	0.92	-0.27	-0.16	-0.02	-0.16	0.05	0.96	-0.30	-0.16	0.04	-0.16	0.06	0.94
113263	GLCCI1	-0.10	0.19	0.45	0.19	0.09	0.93	0.06	0.20	0.40	0.20	0.08	0.97	-0.01	0.20	0.41	0.20	0.09	0.95
113452	TMEM54	-0.37	-0.17	0.09	-0.17	0.06	0.95	-0.30	-0.18	-0.01	-0.18	0.05	0.99	-0.34	-0.17	-0.01	-0.18	0.07	0.95
113675	SDSL	-0.11	0.18	0.37	0.18	0.07	0.95	0.06	0.17	0.33	0.18	0.06	0.97	-0.02	0.19	0.35	0.19	0.07	0.97
114571	SLC22A9	-0.32	-0.15	0.04	-0.15	0.06	0.93	-0.27	-0.15	-0.02	-0.15	0.05	0.97	-0.33	-0.15	0.08	-0.15	0.06	0.96
114787	GPRIN1	-0.37	-0.17	0.07	-0.18	0.06	0.97	-0.32	-0.18	-0.07	-0.18	0.06	0.99	-0.33	-0.20	-0.02	-0.20	0.06	0.97
114804	RNF157	-0.12	0.15	0.38	0.15	0.07	0.92	0.00	0.16	0.33	0.16	0.07	0.94	-0.04	0.17	0.31	0.17	0.07	0.92
114990	VASN	-0.29	-0.15	0.06	-0.14	0.05	0.94	-0.27	-0.15	-0.05	-0.15	0.04	0.99	-0.27	-0.15	-0.02	-0.15	0.05	0.96
116028	RMI2	-0.29	-0.14	0.08	-0.15	0.06	0.92	-0.27	-0.15	0.00	-0.16	0.05	0.98	-0.28	-0.17	-0.03	-0.16	0.05	0.95
116135	LRRC3B	-0.47	-0.20	0.11	-0.20	0.09	0.95	-0.38	-0.21	-0.05	-0.21	0.07	0.97	-0.40	-0.22	0.00	-0.22	0.09	0.97
116150	NUS1	-0.31	-0.14	0.02	-0.14	0.05	0.91	-0.26	-0.14	-0.05	-0.15	0.05	0.96	-0.29	-0.15	-0.02	-0.16	0.06	0.95
116535	MRGPRF	-0.11	0.15	0.38	0.16	0.07	0.92	0.01	0.16	0.31	0.16	0.06	0.96	0.00	0.18	0.31	0.18	0.06	0.97
116966	WDR17	-0.28	-0.13	0.04	-0.13	0.05	0.91	-0.23	-0.14	-0.04	-0.14	0.04	0.97	-0.28	-0.14	0.00	-0.14	0.05	0.93
117154	DACH2	-0.44	-0.19	0.11	-0.19	0.08	0.93	-0.39	-0.19	-0.03	-0.20	0.07	0.97	-0.41	-0.22	0.01	-0.21	0.08	0.96
117245	HRASLS5	-0.29	-0.14	0.05	-0.14	0.05	0.92	-0.23	-0.14	-0.04	-0.14	0.04	0.96	-0.27	-0.16	-0.04	-0.15	0.05	0.96
118427	OLFM3	-0.32	-0.18	0.10	-0.18	0.07	0.95	-0.30	-0.18	-0.06	-0.18	0.06	0.98	-0.35	-0.19	0.04	-0.19	0.07	0.95
118429	ANTXR2	-0.08	0.16	0.32	0.16	0.06	0.95	0.06	0.17	0.30	0.17	0.05	0.98	0.03	0.17	0.30	0.17	0.05	0.96
119587	CPXM2	-0.01	0.14	0.29	0.14	0.05	0.93	0.07	0.16	0.26	0.16	0.04	0.99	-0.03	0.16	0.30	0.16	0.05	0.96
122622	ADSSL1	-0.08	0.15	0.31	0.15	0.05	0.93	0.05	0.16	0.27	0.16	0.05	0.96	0.04	0.17	0.30	0.17	0.06	0.97
122953	JDP2	-0.15	0.19	0.48	0.20	0.09	0.94	0.02	0.21	0.41	0.21	0.08	0.98	-0.03	0.21	0.39	0.21	0.09	0.96
126755	LRRC38	-0.13	0.17	0.38	0.17	0.07	0.94	0.05	0.17	0.32	0.17	0.05	0.99	0.01	0.19	0.35	0.18	0.07	0.96
127833	SYT2	-0.14	0.24	0.50	0.24	0.09	0.98	0.09	0.25	0.46	0.25	0.08	1.00	0.01	0.26	0.46	0.26	0.09	0.99
128434	VSTM2L	-0.37	-0.18	0.13	-0.18	0.08	0.95	-0.34	-0.19	-0.04	-0.18	0.07	0.96	-0.38	-0.18	0.05	-0.18	0.08	0.92
128611	ZNF831	-0.45	-0.16	0.10	-0.16	0.08	0.91	-0.31	-0.15	0.01	-0.16	0.07	0.91	-0.37	-0.18	0.02	-0.18	0.08	0.94
130399	ACVR1C	-0.07	0.20	0.41	0.20	0.07	0.98	0.03	0.21	0.41	0.22	0.06	0.99	0.05	0.22	0.37	0.22	0.07	0.99
132160	PPM1M	-0.48	-0.19	0.07	-0.19	0.08	0.94	-0.36	-0.20	-0.07	-0.20	0.07	0.98	-0.40	-0.21	-0.03	-0.22	0.08	0.97
132321	C4orf33	-0.12	0.19	0.42	0.18	0.08	0.94	0.03	0.18	0.38	0.20	0.07	0.96	0.00	0.20	0.38	0.21	0.08	0.97
133418	EMB	-0.12	0.16	0.36	0.16	0.07	0.94	0.01	0.16	0.29	0.16	0.06	0.97	-0.02	0.17	0.32	0.17	0.07	0.94
134548	SOWAHA	-0.39	-0.18	0.09	-0.18	0.07	0.95	-0.33	-0.18	-0.02	-0.18	0.06	0.97	-0.39	-0.19	-0.01	-0.19	0.07	0.96
138428	PTRH1	-0.27	-0.13	0.04	-0.14	0.05	0.94	-0.25	-0.15	-0.05	-0.15	0.05	0.99	-0.26	-0.15	-0.02	-0.15	0.05	0.95
139221	MUM1L1	-0.42	-0.20	0.14	-0.21	0.08	0.96	-0.39	-0.21	0.00	-0.21	0.07	0.97	-0.44	-0.22	0.02	-0.22	0.09	0.95
139411	PTCHD1	-0.45	-0.18	0.08	-0.18	0.08	0.95	-0.34	-0.17	-0.03	-0.18	0.07	0.96	-0.38	-0.20	-0.02	-0.20	0.07	0.97
139728	PNCK	-0.41	-0.17	0.11	-0.16	0.07	0.91	-0.34	-0.16	-0.03	-0.17	0.07	0.96	-0.33	-0.18	-0.01	-0.18	0.07	0.93
143279	HECTD2	-0.31	-0.15	0.06	-0.15	0.06	0.93	-0.25	-0.16	-0.06	-0.16	0.05	0.99	-0.29	-0.18	-0.01	-0.18	0.06	0.98
144348	ZNF664	-0.05	0.13	0.23	0.13	0.04	0.94	0.05	0.12	0.22	0.13	0.04	0.94	0.01	0.13	0.22	0.13	0.04	0.91
144402	CPNE8	-0.32	-0.18	0.07	-0.18	0.06	0.97	-0.32	-0.18	-0.07	-0.19	0.05	0.99	-0.31	-0.18	0.02	-0.18	0.06	0.97
146760	RTN4RL1	-0.25	-0.13	0.09	-0.13	0.05	0.92	-0.22	-0.12	0.01	-0.13	0.04	0.96	-0.22	-0.14	-0.02	-0.13	0.05	0.92
147463	ANKRD29	-0.12	0.19	0.43	0.19	0.08	0.97	0.03	0.19	0.36	0.20	0.07	0.96	0.00	0.20	0.39	0.20	0.07	0.96
147650	SPACA6	-0.10	0.14	0.33	0.14	0.06	0.90	0.00	0.15	0.29	0.15	0.06	0.96	0.01	0.16	0.29	0.16	0.06	0.93
147968	CAPN12	-0.31	-0.17	0.08	-0.17	0.06	0.96	-0.28	-0.18	-0.06	-0.18	0.05	0.98	-0.30	-0.18	-0.01	-0.18	0.06	0.96
148281	SYT6	-0.12	0.19	0.40	0.19	0.08	0.95	0.02	0.19	0.31	0.19	0.06	0.97	-0.04	0.19	0.37	0.19	0.08	0.94
149473	CCDC24	-0.26	-0.14	0.05	-0.14	0.05	0.93	-0.24	-0.14	-0.04	-0.14	0.04	0.99	-0.27	-0.14	0.03	-0.14	0.05	0.92
150209	AIFM3	-0.10	0.15	0.38	0.15	0.07	0.90	0.02	0.15	0.31	0.15	0.06	0.92	-0.01	0.17	0.32	0.17	0.07	0.94
151516	ASPRV1	0.00	0.12	0.23	0.12	0.04	0.92	0.03	0.13	0.22	0.13	0.04	0.95	0.01	0.13	0.21	0.13	0.04	0.96
152189	CMTM8	-0.33	-0.15	0.10	-0.16	0.06	0.94	-0.30	-0.17	-0.08	-0.17	0.05	1.00	-0.34	-0.19	0.02	-0.18	0.06	0.97
152273	FGD5	-0.08	0.16	0.35	0.16	0.06	0.96	0.05	0.17	0.32	0.17	0.05	0.97	0.02	0.17	0.30	0.17	0.06	0.98
152940	C4orf45	-0.33	-0.17	0.10	-0.17	0.06	0.96	-0.28	-0.18	-0.06	-0.18	0.05	0.99	-0.33	-0.19	-0.04	-0.19	0.06	0.98
155382	VPS37D	-0.36	-0.14	0.08	-0.14	0.06	0.91	-0.29	-0.16	-0.04	-0.16	0.06	0.95	-0.30	-0.16	-0.01	-0.16	0.06	0.93
160760	PPTC7	-0.12	0.17	0.40	0.18	0.07	0.96	0.06	0.18	0.37	0.18	0.06	0.98	-0.03	0.19	0.37	0.19	0.08	0.95
162494	RHBDL3	-0.15	0.21	0.49	0.21	0.09	0.95	0.04	0.21	0.42	0.21	0.08	0.98	0.00	0.22	0.41	0.23	0.09	0.96
163183	SYNE4	-0.12	0.20	0.48	0.20	0.08	0.96	0.03	0.20	0.40	0.21	0.07	0.97	0.01	0.21	0.43	0.21	0.09	0.97
163782	KANK4	-0.09	0.20	0.37	0.20	0.06	0.98	0.10	0.21	0.32	0.22	0.05	1.00	0.03	0.21	0.38	0.21	0.06	0.99
165215	FAM171B	-0.39	-0.17	0.08	-0.17	0.07	0.91	-0.32	-0.17	-0.02	-0.17	0.06	0.98	-0.35	-0.18	0.00	-0.19	0.07	0.95
167681	PRSS35	-0.43	-0.18	0.08	-0.18	0.08	0.91	-0.36	-0.19	-0.02	-0.19	0.07	0.96	-0.37	-0.19	0.02	-0.19	0.08	0.93
167691	LCA5	-0.09	0.16	0.38	0.16	0.07	0.92	0.01	0.17	0.37	0.17	0.07	0.94	-0.01	0.18	0.35	0.18	0.07	0.95
170850	KCNG3	-0.40	-0.17	0.11	-0.17	0.07	0.92	-0.28	-0.16	-0.02	-0.16	0.06	0.95	-0.35	-0.18	0.02	-0.18	0.07	0.94
192668	CYS1	-0.29	-0.14	0.03	-0.14	0.05	0.94	-0.24	-0.15	-0.04	-0.15	0.04	0.99	-0.27	-0.16	0.01	-0.16	0.05	0.97
196383	RILPL2	-0.45	-0.20	0.13	-0.20	0.09	0.95	-0.39	-0.20	-0.04	-0.21	0.07	0.99	-0.39	-0.21	-0.02	-0.22	0.08	0.96
199800	ADM5	-0.29	-0.13	0.07	-0.13	0.05	0.92	-0.26	-0.14	-0.03	-0.14	0.05	0.96	-0.26	-0.15	0.00	-0.15	0.05	0.94
200058	FLJ23867	-0.10	0.17	0.39	0.17	0.07	0.93	0.02	0.17	0.35	0.18	0.07	0.96	0.00	0.18	0.37	0.19	0.07	0.96
200942	KLHDC8B	-0.43	-0.18	0.11	-0.18	0.08	0.94	-0.37	-0.19	-0.06	-0.19	0.07	0.98	-0.38	-0.20	-0.02	-0.20	0.08	0.95
201191	SAMD14	-0.29	-0.15	0.07	-0.15	0.06	0.93	-0.26	-0.17	-0.02	-0.17	0.04	0.98	-0.30	-0.16	0.00	-0.16	0.06	0.94
202333	CMYA5	-0.12	0.19	0.46	0.19	0.08	0.94	0.03	0.20	0.38	0.20	0.07	0.97	0.02	0.22	0.40	0.22	0.08	0.98
203286	ANKS6	-0.06	0.16	0.35	0.16	0.07	0.92	0.05	0.17	0.33	0.17	0.06	0.97	0.03	0.17	0.33	0.18	0.07	0.95
220202	ATOH7	-0.47	-0.20	0.10	-0.19	0.08	0.95	-0.40	-0.20	-0.03	-0.21	0.07	0.98	-0.41	-0.22	-0.02	-0.22	0.08	0.98
221294	NT5DC1	-0.05	0.18	0.34	0.18	0.06	0.96	0.09	0.20	0.31	0.20	0.05	1.00	0.06	0.20	0.34	0.20	0.06	0.99
221336	BEND6	-0.14	0.17	0.42	0.17	0.08	0.93	0.02	0.17	0.36	0.18	0.07	0.95	-0.02	0.18	0.36	0.19	0.08	0.92
221421	RSPH9	-0.44	-0.18	0.10	-0.18	0.08	0.93	-0.36	-0.18	-0.03	-0.19	0.07	0.98	-0.36	-0.20	0.00	-0.20	0.08	0.95
222537	HS3ST5	-0.10	0.19	0.40	0.20	0.07	0.96	0.10	0.21	0.37	0.22	0.06	1.00	0.05	0.21	0.37	0.22	0.07	0.99
253832	ZDHHC20	-0.03	0.14	0.25	0.14	0.05	0.93	0.06	0.16	0.26	0.15	0.04	0.99	0.04	0.15	0.28	0.16	0.05	0.96
254102	EHBP1L1	-0.33	-0.20	0.06	-0.20	0.06	0.97	-0.34	-0.20	-0.08	-0.21	0.05	1.00	-0.36	-0.21	-0.07	-0.21	0.06	1.00
254170	FBXO33	-0.11	0.15	0.37	0.16	0.07	0.91	0.01	0.16	0.36	0.17	0.07	0.97	0.00	0.18	0.35	0.18	0.07	0.95
254263	CNIH2	-0.38	-0.15	0.05	-0.16	0.07	0.91	-0.33	-0.18	-0.06	-0.18	0.06	0.99	-0.34	-0.18	-0.02	-0.18	0.07	0.95
254552	NUDT8	-0.07	0.12	0.29	0.13	0.05	0.91	0.01	0.12	0.26	0.12	0.05	0.92	0.01	0.14	0.25	0.14	0.05	0.90
256281	NUDT14	-0.42	-0.17	0.11	-0.18	0.08	0.92	-0.37	-0.19	-0.04	-0.19	0.07	0.94	-0.38	-0.19	0.03	-0.19	0.09	0.93
260434	PYDC1	-0.40	-0.18	0.09	-0.18	0.07	0.94	-0.35	-0.19	-0.05	-0.20	0.06	0.99	-0.36	-0.20	-0.04	-0.20	0.07	0.97
282969	FUOM	-0.29	-0.16	0.06	-0.16	0.06	0.94	-0.29	-0.17	-0.05	-0.17	0.05	0.98	-0.29	-0.17	-0.01	-0.16	0.06	0.95
283143	LINC00900	-0.30	-0.14	0.08	-0.14	0.06	0.91	-0.26	-0.14	-0.02	-0.15	0.05	0.95	-0.29	-0.16	-0.02	-0.16	0.06	0.92
283209	PGM2L1	-0.40	-0.17	0.07	-0.17	0.07	0.93	-0.34	-0.17	-0.01	-0.17	0.07	0.93	-0.34	-0.18	-0.02	-0.19	0.07	0.96
283284	IGSF22	-0.35	-0.16	0.04	-0.17	0.06	0.95	-0.31	-0.18	-0.07	-0.18	0.05	0.99	-0.34	-0.19	-0.06	-0.19	0.06	0.98
283316	CD163L1	-0.10	0.15	0.30	0.15	0.05	0.95	0.06	0.14	0.26	0.15	0.05	0.99	-0.04	0.15	0.28	0.15	0.06	0.91
284339	TMEM145	-0.12	0.17	0.39	0.17	0.08	0.91	0.03	0.18	0.38	0.19	0.07	0.97	-0.02	0.19	0.40	0.19	0.08	0.94
284348	LYPD5	-0.13	0.19	0.46	0.19	0.09	0.93	0.02	0.20	0.40	0.20	0.08	0.96	-0.02	0.21	0.38	0.21	0.08	0.97
284415	VSTM1	-0.10	0.16	0.35	0.16	0.06	0.94	-0.01	0.15	0.27	0.15	0.05	0.94	-0.05	0.16	0.33	0.16	0.06	0.93
284454	LOC284454	-0.08	0.15	0.30	0.15	0.06	0.94	0.03	0.16	0.31	0.17	0.05	0.99	0.05	0.18	0.29	0.17	0.05	0.98
284485	RIIAD1	-0.38	-0.16	0.05	-0.16	0.07	0.90	-0.33	-0.18	-0.02	-0.18	0.06	0.98	-0.37	-0.18	-0.03	-0.19	0.07	0.95
284611	FAM102B	-0.37	-0.17	0.06	-0.17	0.07	0.93	-0.31	-0.18	-0.05	-0.18	0.06	0.97	-0.35	-0.19	-0.01	-0.19	0.07	0.96
284716	RIMKLA	-0.14	0.17	0.42	0.18	0.08	0.95	0.05	0.18	0.37	0.19	0.07	0.98	-0.01	0.20	0.37	0.20	0.08	0.96
285598	ARL10	-0.32	-0.14	0.04	-0.14	0.05	0.91	-0.27	-0.13	-0.03	-0.14	0.05	0.95	-0.26	-0.14	0.02	-0.14	0.05	0.93
285755	PPIL6	-0.33	-0.15	0.04	-0.15	0.05	0.93	-0.25	-0.16	-0.05	-0.16	0.04	0.97	-0.30	-0.16	-0.02	-0.17	0.05	0.97
285780	LY86-AS1	-0.37	-0.19	0.08	-0.19	0.07	0.96	-0.33	-0.19	-0.02	-0.19	0.06	0.97	-0.37	-0.20	-0.01	-0.20	0.07	0.98
286133	SCARA5	-0.48	-0.22	0.13	-0.22	0.09	0.97	-0.42	-0.24	-0.07	-0.24	0.08	1.00	-0.46	-0.24	0.02	-0.25	0.09	0.98
326624	RAB37	-0.13	0.21	0.46	0.21	0.08	0.95	0.05	0.22	0.42	0.22	0.07	0.98	0.02	0.22	0.41	0.23	0.08	0.98
339983	NAT8L	-0.11	0.16	0.43	0.17	0.08	0.91	0.03	0.18	0.35	0.18	0.07	0.96	-0.03	0.19	0.37	0.19	0.08	0.96
340348	TSPAN33	-0.41	-0.17	0.12	-0.18	0.08	0.94	-0.32	-0.17	0.01	-0.18	0.07	0.96	-0.38	-0.19	0.03	-0.19	0.08	0.93
340719	NANOS1	-0.43	-0.17	0.09	-0.17	0.08	0.91	-0.34	-0.18	-0.04	-0.18	0.07	0.96	-0.36	-0.19	0.01	-0.20	0.08	0.96
342667	STAC2	-0.14	0.21	0.48	0.21	0.09	0.95	0.02	0.22	0.44	0.23	0.08	0.98	0.02	0.23	0.42	0.23	0.08	0.98
347902	AMIGO2	-0.49	-0.19	0.15	-0.19	0.10	0.92	-0.40	-0.19	-0.03	-0.19	0.08	0.93	-0.43	-0.20	0.04	-0.21	0.10	0.92
348013	TMEM255B	-0.13	0.19	0.37	0.19	0.07	0.97	0.05	0.20	0.33	0.20	0.06	0.99	0.02	0.19	0.36	0.20	0.07	0.96
349136	WDR86	-0.50	-0.24	0.13	-0.24	0.09	0.96	-0.46	-0.25	-0.08	-0.25	0.08	1.00	-0.47	-0.25	-0.03	-0.25	0.10	0.98
373156	GSTK1	-0.07	0.14	0.27	0.14	0.05	0.94	0.04	0.15	0.27	0.15	0.05	0.97	0.03	0.16	0.27	0.15	0.05	0.96
373863	DND1	-0.15	0.17	0.38	0.17	0.07	0.93	0.02	0.17	0.36	0.18	0.07	0.96	-0.02	0.18	0.34	0.18	0.07	0.93
374378	GALNT18	-0.26	-0.14	0.07	-0.14	0.05	0.92	-0.24	-0.13	-0.04	-0.13	0.04	0.96	-0.26	-0.14	0.04	-0.14	0.05	0.93
374875	HSD11B1L	-0.35	-0.15	0.08	-0.15	0.07	0.92	-0.31	-0.17	-0.04	-0.17	0.06	0.96	-0.33	-0.17	0.00	-0.17	0.07	0.92
375057	STUM	-0.47	-0.19	0.11	-0.20	0.09	0.93	-0.39	-0.20	0.00	-0.21	0.08	0.97	-0.40	-0.21	0.01	-0.21	0.09	0.96
375449	MAST4	-0.09	0.16	0.39	0.16	0.07	0.90	-0.01	0.16	0.34	0.17	0.07	0.97	-0.01	0.18	0.35	0.19	0.07	0.95
386618	KCTD4	-0.46	-0.20	0.13	-0.20	0.08	0.96	-0.42	-0.20	0.00	-0.21	0.07	0.98	-0.40	-0.22	0.00	-0.22	0.08	0.98
387357	THEMIS	-0.09	0.14	0.29	0.14	0.06	0.90	0.05	0.16	0.30	0.16	0.05	0.95	0.00	0.15	0.29	0.15	0.06	0.91
389073	C2orf80	-0.39	-0.18	0.08	-0.18	0.07	0.96	-0.31	-0.19	-0.07	-0.19	0.06	0.99	-0.37	-0.20	-0.04	-0.20	0.07	0.98
389941	C1QL3	-0.48	-0.18	0.10	-0.18	0.08	0.92	-0.35	-0.18	-0.02	-0.18	0.08	0.95	-0.39	-0.20	0.01	-0.20	0.08	0.95
400120	SERTM1	-0.32	-0.14	0.08	-0.15	0.06	0.93	-0.30	-0.15	-0.05	-0.15	0.06	0.93	-0.32	-0.17	0.03	-0.17	0.06	0.96
400569	MED11	-0.12	0.15	0.35	0.15	0.07	0.91	0.02	0.15	0.31	0.16	0.06	0.95	0.00	0.17	0.32	0.17	0.07	0.95
400745	SH2D5	-0.36	-0.18	0.09	-0.19	0.07	0.96	-0.32	-0.20	-0.02	-0.19	0.06	0.99	-0.40	-0.21	0.01	-0.20	0.08	0.95
401994	OR14I1	-0.32	-0.16	0.08	-0.16	0.07	0.95	-0.30	-0.16	-0.05	-0.16	0.05	0.94	-0.34	-0.17	0.06	-0.16	0.07	0.93
404217	CTXN1	-0.47	-0.21	0.10	-0.21	0.08	0.96	-0.41	-0.22	-0.03	-0.22	0.07	0.98	-0.40	-0.23	-0.04	-0.23	0.08	0.99

407738	FAM19A1	-0.27	-0.13	0.07	-0.14	0.05	0.92	-0.25	-0.14	-0.04	-0.14	0.05	0.94	-0.26	-0.15	0.00	-0.15	0.05	0.93
415116	PIM3	-0.12	0.16	0.40	0.17	0.08	0.91	0.01	0.17	0.35	0.17	0.07	0.94	-0.02	0.18	0.36	0.18	0.08	0.93
441027	TMEM150C	-0.34	-0.15	0.13	-0.15	0.07	0.91	-0.28	-0.16	0.00	-0.16	0.06	0.92	-0.35	-0.17	0.10	-0.16	0.08	0.90
441108	C5orf56	-0.07	0.14	0.27	0.14	0.05	0.93	0.03	0.15	0.26	0.15	0.04	0.97	0.01	0.14	0.27	0.15	0.05	0.95
494470	RNF165	-0.26	-0.13	0.01	-0.13	0.04	0.93	-0.23	-0.14	-0.05	-0.14	0.04	0.98	-0.27	-0.15	-0.02	-0.15	0.05	0.95
503542	SPRN	-0.37	-0.19	0.08	-0.20	0.07	0.97	-0.33	-0.20	-0.05	-0.20	0.06	0.98	-0.37	-0.21	-0.02	-0.21	0.08	0.97
574029	DUSP5P1	-0.29	-0.14	0.08	-0.14	0.06	0.90	-0.27	-0.14	-0.04	-0.14	0.05	0.97	-0.30	-0.15	-0.01	-0.15	0.05	0.94
574036	SERTAD4-AS1	-0.11	0.20	0.48	0.20	0.08	0.94	0.06	0.21	0.41	0.21	0.08	0.98	0.03	0.21	0.40	0.22	0.09	0.97
642273	FAM110C	-0.52	-0.24	0.15	-0.24	0.09	0.98	-0.43	-0.24	-0.10	-0.25	0.08	1.00	-0.45	-0.26	-0.04	-0.26	0.09	0.99
642852	LOC642852	-0.36	-0.16	0.08	-0.16	0.06	0.93	-0.30	-0.17	-0.07	-0.16	0.05	0.99	-0.32	-0.17	-0.03	-0.17	0.07	0.93
643037	C11orf97	-0.42	-0.18	0.09	-0.17	0.08	0.91	-0.39	-0.20	-0.06	-0.20	0.07	0.97	-0.40	-0.20	-0.02	-0.21	0.08	0.96
646627	LYPD8	-0.42	-0.20	0.12	-0.20	0.08	0.95	-0.40	-0.21	-0.05	-0.21	0.07	0.97	-0.44	-0.21	0.01	-0.21	0.09	0.93
654502	IQCJ	-0.34	-0.16	0.08	-0.16	0.06	0.94	-0.26	-0.14	-0.04	-0.14	0.04	0.98	-0.31	-0.17	0.00	-0.16	0.06	0.97
654790	PCP4L1	-0.10	0.17	0.40	0.17	0.07	0.93	0.07	0.17	0.32	0.18	0.06	0.98	0.03	0.18	0.34	0.18	0.06	0.97
100170841	EPOP	-0.37	-0.17	0.09	-0.17	0.07	0.94	-0.31	-0.16	-0.03	-0.17	0.06	0.94	-0.36	-0.19	0.00	-0.19	0.07	0.95
100507436	MICA	-0.09	0.17	0.32	0.17	0.06	0.94	0.03	0.17	0.29	0.17	0.05	0.98	0.03	0.18	0.31	0.18	0.06	0.96

Abbreviations: rsFC, resting-state functional connectivity; L, left; A1/2/3ulhf, upper limb, head and face region of area 1/2/3; CNP, the Consortium for Neuropsychiatric Phenomics; SALD, the Southwest University Adult Lifespan Dataset; Min, minimum; Med, median; Max, maximum; SD, standard deviation.

Genes related to rsFC of the R-A1/2/3ulhf in both the discovery and validation experiments

GeneID	Genesymbol	Discovery dataset						CNP dataset						SALD dataset					
		r					Percentage	*r*					Percentage	*r*					Percentage
		Min	Med	Max	Mean	SD		Min	Med	Max	Mean	SD		Min	Med	Max	Mean	SD	
176	ACAN	-0.16	0.21	0.48	0.21	0.10	0.93	0.02	0.22	0.43	0.22	0.10	0.94	-0.02	0.25	0.47	0.25	0.11	0.94
817	CAMK2D	-0.43	-0.19	0.11	-0.19	0.09	0.92	-0.40	-0.20	0.00	-0.20	0.09	0.94	-0.42	-0.23	0.01	-0.22	0.09	0.95
1006	CDH8	-0.40	-0.18	0.08	-0.18	0.08	0.92	-0.38	-0.21	-0.01	-0.20	0.08	0.94	-0.39	-0.21	0.01	-0.21	0.08	0.95
2104	ESRRG	-0.18	0.20	0.49	0.20	0.10	0.91	-0.02	0.21	0.44	0.21	0.10	0.93	-0.01	0.23	0.45	0.23	0.11	0.92
2161	F12	-0.47	-0.18	0.07	-0.18	0.09	0.90	-0.39	-0.20	-0.03	-0.20	0.08	0.91	-0.41	-0.21	-0.02	-0.22	0.09	0.94
2830	GPR6	-0.33	-0.17	0.02	-0.16	0.06	0.92	-0.33	-0.17	-0.01	-0.17	0.06	0.92	-0.36	-0.19	0.00	-0.19	0.07	0.93
2952	GSTT1	-0.16	0.18	0.44	0.19	0.09	0.90	0.00	0.20	0.40	0.20	0.08	0.91	-0.04	0.22	0.41	0.21	0.10	0.93
3755	KCNG1	-0.46	-0.18	0.16	-0.19	0.10	0.91	-0.44	-0.20	-0.02	-0.21	0.09	0.95	-0.44	-0.22	0.04	-0.22	0.10	0.91
4258	MGST2	-0.13	0.19	0.43	0.19	0.09	0.90	-0.01	0.20	0.38	0.19	0.09	0.90	0.00	0.22	0.42	0.21	0.09	0.93
4286	MITF	-0.05	0.16	0.34	0.16	0.07	0.90	0.02	0.16	0.29	0.17	0.06	0.91	0.01	0.18	0.34	0.18	0.07	0.93
4609	MYC	-0.09	0.17	0.37	0.17	0.08	0.92	0.03	0.17	0.37	0.17	0.07	0.90	-0.06	0.19	0.35	0.19	0.08	0.92
4703	NEB	-0.14	0.19	0.42	0.19	0.09	0.92	-0.01	0.21	0.39	0.21	0.09	0.95	-0.05	0.21	0.44	0.21	0.10	0.93
5176	SERPINF1	-0.31	-0.17	0.08	-0.16	0.07	0.91	-0.31	-0.19	0.05	-0.17	0.07	0.93	-0.35	-0.19	0.01	-0.19	0.07	0.93
5580	PRKCD	-0.49	-0.19	0.14	-0.19	0.10	0.91	-0.46	-0.20	0.01	-0.21	0.10	0.93	-0.45	-0.22	0.00	-0.23	0.10	0.92
6330	SCN4B	-0.16	0.20	0.45	0.21	0.09	0.94	0.03	0.21	0.43	0.21	0.09	0.93	-0.05	0.24	0.45	0.24	0.10	0.93
8938	BAIAP3	-0.55	-0.20	0.18	-0.20	0.12	0.92	-0.48	-0.21	-0.01	-0.22	0.10	0.91	-0.51	-0.23	0.04	-0.24	0.12	0.91
9033	PKD2L1	-0.37	-0.17	0.06	-0.17	0.08	0.92	-0.30	-0.18	0.01	-0.17	0.07	0.91	-0.40	-0.20	0.05	-0.19	0.08	0.93
9607	CARTPT	-0.42	-0.21	0.12	-0.20	0.09	0.95	-0.38	-0.23	0.02	-0.22	0.09	0.92	-0.42	-0.22	0.06	-0.22	0.09	0.91
10154	PLXNC1	-0.40	-0.16	0.10	-0.17	0.08	0.91	-0.37	-0.17	-0.05	-0.18	0.07	0.90	-0.36	-0.19	-0.02	-0.19	0.08	0.91
10231	RCAN2	-0.11	0.16	0.40	0.17	0.08	0.90	0.04	0.18	0.38	0.18	0.08	0.94	-0.01	0.20	0.37	0.19	0.08	0.91
22801	ITGA11	-0.09	0.18	0.38	0.18	0.08	0.92	-0.01	0.20	0.35	0.20	0.08	0.92	-0.01	0.20	0.42	0.20	0.08	0.91
22987	SV2C	-0.17	0.18	0.42	0.18	0.09	0.91	0.01	0.19	0.42	0.19	0.09	0.92	-0.10	0.21	0.42	0.21	0.10	0.90
25841	ABTB2	-0.09	0.18	0.39	0.18	0.08	0.90	0.03	0.20	0.37	0.20	0.08	0.93	0.00	0.22	0.42	0.22	0.08	0.94
25989	ULK3	-0.13	0.17	0.42	0.17	0.08	0.91	0.00	0.19	0.38	0.19	0.08	0.92	0.00	0.19	0.39	0.20	0.08	0.91
55244	SLC47A1	-0.13	0.22	0.44	0.22	0.09	0.96	0.04	0.23	0.43	0.23	0.09	0.93	0.02	0.24	0.44	0.24	0.09	0.94
57495	NWD2	-0.36	-0.18	0.09	-0.18	0.07	0.93	-0.34	-0.18	-0.02	-0.18	0.08	0.91	-0.36	-0.20	0.03	-0.20	0.08	0.93
57526	PCDH19	-0.42	-0.19	0.10	-0.18	0.08	0.92	-0.37	-0.20	0.01	-0.19	0.08	0.91	-0.41	-0.22	0.00	-0.21	0.09	0.93
79660	PPP1R3B	-0.03	0.14	0.31	0.14	0.05	0.90	0.03	0.14	0.27	0.14	0.05	0.91	0.00	0.16	0.29	0.16	0.06	0.92
80020	FOXRED2	-0.36	-0.18	0.09	-0.18	0.08	0.93	-0.34	-0.18	0.00	-0.18	0.07	0.93	-0.37	-0.20	0.01	-0.20	0.08	0.93
80307	FER1L4	-0.16	0.19	0.45	0.20	0.09	0.91	0.01	0.22	0.42	0.21	0.09	0.92	-0.01	0.23	0.43	0.23	0.10	0.94
81849	ST6GALNAC5	-0.45	-0.20	0.10	-0.19	0.09	0.91	-0.39	-0.22	-0.02	-0.21	0.08	0.94	-0.45	-0.23	0.03	-0.23	0.09	0.94
84034	EMILIN2	-0.07	0.18	0.31	0.18	0.06	0.95	0.04	0.19	0.31	0.19	0.06	0.96	0.00	0.21	0.36	0.20	0.07	0.95
91252	SLC39A13	-0.13	0.17	0.45	0.18	0.09	0.91	0.01	0.18	0.43	0.19	0.09	0.90	-0.01	0.21	0.40	0.21	0.09	0.92
127833	SYT2	-0.16	0.20	0.46	0.20	0.10	0.91	0.00	0.22	0.44	0.22	0.09	0.94	-0.04	0.24	0.45	0.24	0.10	0.94
130399	ACVR1C	-0.14	0.17	0.38	0.17	0.08	0.92	-0.01	0.19	0.39	0.19	0.08	0.91	0.01	0.20	0.37	0.20	0.08	0.93
163782	KANK4	-0.06	0.17	0.34	0.17	0.07	0.92	0.04	0.18	0.31	0.19	0.07	0.94	-0.02	0.20	0.37	0.20	0.07	0.93
221294	NT5DC1	-0.06	0.15	0.31	0.15	0.06	0.91	0.03	0.17	0.31	0.17	0.06	0.93	0.01	0.19	0.33	0.18	0.07	0.93
254102	EHBP1L1	-0.33	-0.17	0.04	-0.17	0.07	0.93	-0.33	-0.18	-0.03	-0.18	0.06	0.94	-0.34	-0.19	-0.01	-0.19	0.07	0.95
286133	SCARA5	-0.46	-0.18	0.17	-0.18	0.10	0.90	-0.41	-0.21	-0.01	-0.21	0.09	0.91	-0.46	-0.22	0.06	-0.22	0.10	0.92
349136	WDR86	-0.49	-0.20	0.14	-0.20	0.10	0.91	-0.46	-0.23	0.01	-0.22	0.10	0.93	-0.47	-0.23	0.03	-0.23	0.11	0.91
642273	FAM110C	-0.51	-0.20	0.17	-0.20	0.10	0.91	-0.43	-0.21	-0.03	-0.22	0.10	0.93	-0.47	-0.23	0.03	-0.24	0.11	0.94

Abbreviations: rsFC, resting-state functional connectivity; R, right; A1/2/3ulhf, upper limb, head and face region of area 1/2/3; CNP, the Consortium for Neuropsychiatric Phenomics; SALD, the Southwest University Adult Lifespan Dataset; Min, minimum; Med, median; Max, maximum; SD, standard deviation.

Genes related to rsFC of the L-A1/2/3tonIa in both the discovery and validation experiments

GeneID	Genesymbol	Discovery dataset						CNP dataset						SALD dataset					
		r					Percentage	*r*					Percentage	*r*					Percentage
		Min	Med	Max	Mean	SD		Min	Med	Max	Mean	SD		Min	Med	Max	Mean	SD	
1300	COL10A1	-0.27	-0.16	-0.01	-0.16	0.05	0.93	-0.26	-0.17	-0.03	-0.17	0.05	0.96	-0.28	-0.16	0.01	-0.15	0.05	0.94
2634	GBP2	0.02	0.15	0.25	0.15	0.04	0.92	0.04	0.15	0.23	0.15	0.04	0.98	0.01	0.15	0.25	0.14	0.04	0.94
2888	GRB14	-0.34	-0.19	-0.02	-0.18	0.06	0.94	-0.33	-0.19	-0.05	-0.19	0.06	0.95	-0.39	-0.19	0.00	-0.19	0.07	0.95
5587	PRKD1	-0.24	-0.15	-0.02	-0.14	0.04	0.95	-0.24	-0.13	0.00	-0.13	0.04	0.94	-0.22	-0.12	0.00	-0.12	0.04	0.90
9033	PKD2L1	-0.32	-0.17	0.03	-0.17	0.06	0.91	-0.30	-0.18	-0.01	-0.18	0.07	0.92	-0.34	-0.17	0.02	-0.17	0.07	0.91
9312	KCNB2	-0.32	-0.16	0.00	-0.16	0.06	0.92	-0.30	-0.17	0.01	-0.17	0.06	0.92	-0.34	-0.18	0.00	-0.17	0.06	0.94
9607	CARTPT	-0.43	-0.25	-0.04	-0.25	0.08	0.98	-0.41	-0.25	-0.03	-0.25	0.08	0.98	-0.43	-0.25	0.00	-0.24	0.08	0.98
22801	ITGA11	-0.02	0.19	0.38	0.18	0.07	0.93	0.05	0.20	0.38	0.20	0.06	0.97	0.00	0.19	0.38	0.18	0.07	0.92
51454	GULP1	-0.36	-0.18	0.07	-0.18	0.07	0.93	-0.34	-0.20	-0.02	-0.19	0.07	0.98	-0.42	-0.19	0.01	-0.20	0.07	0.95
81849	ST6GALNAC5	-0.40	-0.18	0.00	-0.18	0.07	0.90	-0.36	-0.21	-0.07	-0.21	0.07	0.96	-0.41	-0.20	0.00	-0.20	0.08	0.92
84034	EMILIN2	-0.03	0.16	0.33	0.16	0.06	0.93	0.02	0.18	0.30	0.18	0.06	0.94	-0.02	0.16	0.30	0.16	0.06	0.91
94160	ABCC12	-0.28	-0.15	0.03	-0.15	0.06	0.90	-0.28	-0.16	0.01	-0.16	0.06	0.92	-0.30	-0.16	0.02	-0.16	0.06	0.93
118427	OLFM3	-0.31	-0.15	0.03	-0.15	0.06	0.90	-0.29	-0.15	0.03	-0.16	0.06	0.91	-0.32	-0.16	0.00	-0.16	0.06	0.90
118429	ANTXR2	0.01	0.18	0.32	0.18	0.05	0.95	0.08	0.18	0.32	0.18	0.05	0.99	0.03	0.18	0.30	0.18	0.05	0.95
147968	CAPN12	-0.31	-0.16	0.01	-0.16	0.05	0.91	-0.31	-0.16	0.01	-0.16	0.06	0.93	-0.29	-0.16	0.03	-0.16	0.06	0.92

Abbreviations: rsFC, resting-state functional connectivity; L, left; A1/2/3tonIa, tongue and larynx region of area 1/2/3; CNP, the Consortium for Neuropsychiatric Phenomics; SALD, the Southwest University Adult Lifespan Dataset;
Min, minimum; Med, median; Max, maximum; SD, standard deviation.

Genes related to rsFC of the R-A1/2/3tonIa in both the discovery and validation experiments

GeneID	Genesymbol	Discovery dataset						CNP dataset						SALD dataset					
		r					Percentage	*r*					Percentage	*r*					Percentage
		Min	Med	Max	Mean	SD		Min	Med	Max	Mean	SD		Min	Med	Max	Mean	SD	
9607	CARTPT	-0.40	-0.23	0.16	-0.22	0.09	0.93	-0.43	-0.25	-0.01	-0.24	0.08	0.97	-0.43	-0.25	0.06	-0.24	0.08	0.96

Abbreviations: rsFC, resting-state functional connectivity; R, right; A1/2/3tonIa, tongue and larynx region of area 1/2/3; CNP, the Consortium for Neuropsychiatric Phenomics; SALD, the Southwest University Adult Lifespan Dataset;
Min, minimum; Med, median; Max, maximum; SD, standard deviation.

Genes related to rsFC of the L-A2 in both the discovery and validation experiments

GeneID	Genesymbol	Discovery dataset						CNP dataset						SALD dataset					
		r					Percentage	*r*					Percentage	*r*					Percentage
		Min	Med	Max	Mean	SD		Min	Med	Max	Mean	SD		Min	Med	Max	Mean	SD	
92	ACVR2A	-0.30	-0.14	0.00	-0.14	0.05	0.91	-0.30	-0.17	-0.06	-0.17	0.05	0.98	-0.30	-0.18	-0.03	-0.17	0.06	0.96
176	ACAN	-0.16	0.20	0.48	0.21	0.10	0.92	0.02	0.23	0.45	0.23	0.09	0.96	0.02	0.25	0.47	0.25	0.09	0.97
793	CALB1	-0.42	-0.18	0.12	-0.18	0.08	0.91	-0.36	-0.20	-0.01	-0.19	0.08	0.93	-0.41	-0.21	-0.01	-0.21	0.08	0.97
817	CAMK2D	-0.44	-0.18	0.14	-0.19	0.08	0.91	-0.42	-0.21	-0.02	-0.21	0.08	0.95	-0.42	-0.22	0.00	-0.22	0.08	0.97
2044	EPHA5	-0.28	-0.15	0.01	-0.15	0.05	0.96	-0.31	-0.17	-0.06	-0.17	0.05	0.96	-0.30	-0.18	-0.03	-0.18	0.05	0.97
2823	GPM6A	-0.34	-0.15	0.08	-0.15	0.06	0.90	-0.30	-0.16	-0.01	-0.15	0.06	0.92	-0.32	-0.18	-0.02	-0.18	0.06	0.96
2830	GPR6	-0.31	-0.16	0.03	-0.16	0.06	0.94	-0.34	-0.18	-0.01	-0.18	0.06	0.94	-0.34	-0.19	-0.01	-0.18	0.06	0.93
2952	GSTT1	-0.15	0.19	0.44	0.19	0.09	0.91	0.02	0.21	0.42	0.21	0.08	0.96	0.00	0.22	0.40	0.22	0.08	0.96
3067	HDC	-0.41	-0.17	0.06	-0.17	0.07	0.92	-0.32	-0.19	-0.02	-0.18	0.07	0.91	-0.35	-0.20	-0.06	-0.20	0.06	0.98
3755	KCNG1	-0.47	-0.20	0.11	-0.20	0.09	0.91	-0.44	-0.22	-0.01	-0.22	0.08	0.98	-0.43	-0.23	-0.02	-0.23	0.09	0.97
4703	NEB	-0.19	0.16	0.39	0.17	0.08	0.90	-0.01	0.20	0.40	0.20	0.09	0.92	-0.02	0.19	0.41	0.20	0.08	0.94
6330	SCN4B	-0.11	0.20	0.46	0.21	0.09	0.93	0.01	0.23	0.46	0.23	0.08	0.97	0.03	0.24	0.43	0.25	0.08	0.98
6543	SLC8A2	-0.34	-0.14	0.07	-0.14	0.06	0.91	-0.29	-0.15	-0.01	-0.14	0.06	0.90	-0.29	-0.17	0.00	-0.17	0.06	0.95
6804	STX1A	-0.37	-0.17	0.09	-0.18	0.07	0.95	-0.36	-0.18	-0.01	-0.18	0.07	0.90	-0.38	-0.21	-0.03	-0.21	0.07	0.98
7534	YWHAZ	-0.31	-0.15	0.03	-0.15	0.06	0.92	-0.30	-0.16	-0.02	-0.16	0.06	0.94	-0.31	-0.17	-0.01	-0.17	0.06	0.95
8209	C21orf33	-0.03	0.14	0.29	0.14	0.05	0.91	0.04	0.16	0.26	0.16	0.05	0.94	0.02	0.17	0.28	0.17	0.05	0.97
8704	B4GALT2	-0.41	-0.18	0.13	-0.18	0.08	0.90	-0.35	-0.19	-0.02	-0.18	0.07	0.93	-0.40	-0.20	-0.03	-0.20	0.08	0.96
9651	PLCH2	-0.27	-0.14	0.01	-0.14	0.05	0.91	-0.26	-0.15	-0.03	-0.14	0.05	0.91	-0.30	-0.16	0.01	-0.16	0.05	0.94
10160	FARP1	-0.45	-0.18	0.07	-0.18	0.09	0.90	-0.40	-0.19	-0.02	-0.20	0.08	0.94	-0.42	-0.21	-0.02	-0.22	0.08	0.96
10231	RCAN2	-0.06	0.16	0.40	0.17	0.08	0.91	0.05	0.18	0.40	0.20	0.07	0.97	0.00	0.20	0.38	0.20	0.07	0.95
22987	SV2C	-0.07	0.20	0.50	0.20	0.08	0.94	0.05	0.22	0.45	0.22	0.08	0.97	0.03	0.23	0.44	0.23	0.08	0.97
22996	TTC39A	-0.08	0.15	0.33	0.15	0.07	0.91	0.04	0.17	0.32	0.16	0.06	0.95	0.00	0.18	0.35	0.18	0.07	0.93
23406	COTL1	-0.02	0.13	0.27	0.13	0.05	0.90	0.03	0.14	0.28	0.14	0.05	0.91	-0.03	0.15	0.29	0.15	0.06	0.91
23484	LEPROTL1	-0.10	0.16	0.38	0.17	0.08	0.91	0.02	0.19	0.38	0.19	0.07	0.96	-0.01	0.20	0.37	0.19	0.07	0.96
25953	PNKD	-0.07	0.16	0.40	0.16	0.07	0.91	0.05	0.18	0.37	0.17	0.06	0.93	0.01	0.19	0.36	0.19	0.07	0.95
27132	CPNE7	-0.47	-0.18	0.13	-0.18	0.09	0.90	-0.37	-0.20	0.00	-0.19	0.08	0.95	-0.41	-0.21	-0.01	-0.21	0.08	0.98
29803	REPIN1	-0.11	0.16	0.38	0.16	0.07	0.90	0.02	0.18	0.34	0.18	0.07	0.95	0.02	0.19	0.35	0.19	0.07	0.97
50486	G0S2	-0.07	0.14	0.28	0.14	0.06	0.90	0.04	0.16	0.34	0.16	0.05	0.95	-0.02	0.18	0.33	0.18	0.06	0.95
54492	NEURL1B	-0.41	-0.18	0.00	-0.18	0.07	0.94	-0.34	-0.19	-0.04	-0.20	0.06	0.96	-0.37	-0.22	-0.03	-0.21	0.06	0.99
54793	KCTD9	-0.09	0.17	0.43	0.18	0.08	0.90	0.04	0.20	0.41	0.19	0.07	0.97	-0.03	0.21	0.39	0.20	0.08	0.94
55040	EPN3	-0.09	0.17	0.43	0.18	0.08	0.91	0.05	0.20	0.42	0.20	0.07	0.97	0.00	0.21	0.41	0.21	0.08	0.95
55244	SLC47A1	-0.15	0.20	0.43	0.21	0.08	0.96	0.05	0.23	0.45	0.23	0.08	0.97	-0.02	0.23	0.43	0.23	0.08	0.97
55686	MREG	-0.06	0.15	0.34	0.15	0.06	0.91	0.06	0.17	0.34	0.17	0.05	0.96	-0.01	0.18	0.32	0.18	0.06	0.94
55853	IDI2-AS1	-0.10	0.17	0.40	0.18	0.07	0.92	0.04	0.19	0.37	0.18	0.07	0.94	0.02	0.20	0.37	0.20	0.07	0.96
57495	NWD2	-0.36	-0.17	0.11	-0.18	0.07	0.94	-0.36	-0.19	0.00	-0.19	0.07	0.93	-0.36	-0.20	-0.03	-0.20	0.07	0.98
57761	TRIB3	-0.28	-0.13	0.03	-0.13	0.05	0.90	-0.23	-0.15	-0.02	-0.14	0.05	0.91	-0.27	-0.16	-0.04	-0.16	0.05	0.96
80020	FOXRED2	-0.36	-0.17	0.11	-0.17	0.07	0.93	-0.36	-0.18	-0.02	-0.18	0.07	0.91	-0.36	-0.20	-0.02	-0.20	0.07	0.97
80774	LIMD2	-0.23	-0.13	0.04	-0.13	0.04	0.91	-0.26	-0.15	-0.02	-0.15	0.04	0.93	-0.25	-0.15	-0.02	-0.15	0.05	0.95
83445	GSG1	-0.45	-0.17	0.12	-0.18	0.09	0.90	-0.37	-0.19	-0.02	-0.18	0.08	0.91	-0.40	-0.20	-0.02	-0.21	0.08	0.96
84937	ZNRF1	-0.32	-0.15	0.04	-0.15	0.06	0.91	-0.31	-0.18	-0.02	-0.17	0.06	0.96	-0.31	-0.18	-0.02	-0.18	0.06	0.96
85301	COL27A1	-0.04	0.14	0.37	0.14	0.06	0.91	0.02	0.15	0.28	0.15	0.06	0.90	0.00	0.17	0.29	0.17	0.05	0.97
91624	NEXN	-0.13	0.17	0.39	0.17	0.08	0.90	0.01	0.18	0.33	0.18	0.07	0.92	0.00	0.20	0.38	0.20	0.07	0.97
113675	SDSL	-0.08	0.16	0.35	0.16	0.07	0.91	0.03	0.17	0.35	0.17	0.06	0.93	0.01	0.19	0.35	0.19	0.07	0.95
114787	GPRIN1	-0.35	-0.16	0.04	-0.16	0.06	0.93	-0.32	-0.19	-0.02	-0.18	0.06	0.94	-0.34	-0.20	-0.01	-0.20	0.06	0.98
127833	SYT2	-0.16	0.19	0.47	0.20	0.09	0.93	0.02	0.23	0.47	0.22	0.09	0.96	0.02	0.24	0.45	0.24	0.09	0.97
144348	ZNF664	-0.02	0.13	0.23	0.13	0.04	0.91	0.01	0.13	0.22	0.13	0.04	0.92	0.03	0.13	0.24	0.13	0.04	0.93
160760	PPTC7	-0.08	0.17	0.43	0.18	0.08	0.91	0.05	0.19	0.39	0.19	0.07	0.95	0.02	0.21	0.38	0.21	0.07	0.96
254102	EHBP1L1	-0.31	-0.15	0.10	-0.15	0.07	0.90	-0.30	-0.18	-0.01	-0.17	0.07	0.91	-0.32	-0.17	0.06	-0.17	0.07	0.95
347730	LRRTM1	-0.28	-0.14	-0.01	-0.14	0.05	0.92	-0.27	-0.17	-0.07	-0.16	0.05	0.99	-0.29	-0.17	0.02	-0.16	0.06	0.95
642273	FAM110C	-0.52	-0.21	0.16	-0.21	0.10	0.92	-0.43	-0.23	-0.01	-0.22	0.09	0.93	-0.45	-0.23	-0.03	-0.24	0.09	0.98

Abbreviations: rsFC, resting-state functional connectivity; L, left; A2, area 2; CNP, the Consortium for Neuropsychiatric Phenomics; SALD, the Southwest University Adult Lifespan Dataset; Min, minimum; Med, median; Max, maximum; SD, standard deviation.

Genes related to rsFC of the L-A1/2/3tru in both the discovery and validation experiments

GeneID	Genesymbol	Discovery dataset						CNP dataset						SALD dataset					
		r					Percentage	*r*					Percentage	*r*					Percentage
		Min	Med	Max	Mean	SD		Min	Med	Max	Mean	SD		Min	Med	Max	Mean	SD	
92	ACVR2A	-0.31	-0.15	0.06	-0.14	0.06	0.91	-0.30	-0.15	-0.04	-0.15	0.05	0.97	-0.32	-0.16	0.05	-0.16	0.06	0.94
98	ACYP2	-0.03	0.15	0.41	0.15	0.07	0.91	-0.01	0.14	0.32	0.15	0.06	0.90	-0.10	0.15	0.33	0.15	0.07	0.91
161	AP2A2	-0.33	-0.14	0.01	-0.14	0.06	0.90	-0.26	-0.13	0.01	-0.13	0.05	0.91	-0.29	-0.14	0.04	-0.15	0.06	0.90
176	ACAN	-0.02	0.22	0.52	0.23	0.09	0.96	0.01	0.23	0.43	0.23	0.08	0.99	-0.03	0.24	0.50	0.25	0.09	0.97
286	ANK1	-0.05	0.19	0.53	0.20	0.09	0.92	0.00	0.22	0.41	0.21	0.08	0.96	-0.03	0.21	0.46	0.22	0.09	0.94
308	ANXA5	0.01	0.13	0.22	0.13	0.04	0.94	0.00	0.14	0.23	0.14	0.04	0.95	0.02	0.15	0.27	0.15	0.04	0.96
320	APBA1	-0.24	-0.13	-0.01	-0.13	0.04	0.90	-0.27	-0.14	-0.05	-0.14	0.04	0.98	-0.24	-0.14	-0.01	-0.14	0.04	0.97
341	APOC1	-0.33	-0.15	0.01	-0.16	0.06	0.92	-0.30	-0.16	-0.04	-0.16	0.05	0.97	-0.33	-0.17	0.09	-0.17	0.06	0.96
367	AR	-0.06	0.18	0.44	0.19	0.08	0.94	0.02	0.19	0.38	0.19	0.07	0.95	-0.04	0.19	0.39	0.20	0.08	0.95
430	ASCL2	-0.53	-0.19	0.04	-0.20	0.09	0.93	-0.40	-0.21	-0.02	-0.21	0.08	0.98	-0.43	-0.22	0.05	-0.22	0.09	0.95
445	ASS1	-0.38	-0.18	0.06	-0.18	0.07	0.92	-0.34	-0.19	-0.06	-0.19	0.06	0.97	-0.41	-0.21	-0.01	-0.21	0.07	0.96
460	ASTN1	-0.36	-0.16	0.02	-0.16	0.06	0.94	-0.32	-0.17	-0.05	-0.17	0.06	0.96	-0.32	-0.18	0.06	-0.18	0.06	0.96
493	ATP2B4	-0.51	-0.17	0.05	-0.18	0.09	0.90	-0.37	-0.18	0.02	-0.18	0.08	0.95	-0.43	-0.19	0.07	-0.19	0.10	0.91
586	BCAT1	-0.07	0.16	0.36	0.16	0.06	0.93	0.00	0.15	0.34	0.15	0.06	0.92	-0.05	0.16	0.33	0.16	0.06	0.93
784	CACNB3	-0.34	-0.17	-0.02	-0.17	0.05	0.97	-0.30	-0.16	-0.05	-0.16	0.05	0.98	-0.33	-0.18	0.06	-0.18	0.05	0.98
793	CALB1	-0.43	-0.20	0.00	-0.20	0.07	0.96	-0.35	-0.21	-0.04	-0.21	0.07	0.97	-0.41	-0.23	0.05	-0.22	0.08	0.97
814	CAMK4	-0.33	-0.19	-0.04	-0.19	0.06	0.98	-0.32	-0.18	-0.03	-0.18	0.05	0.97	-0.34	-0.20	0.01	-0.20	0.05	0.99
817	CAMK2D	-0.47	-0.21	0.00	-0.21	0.08	0.96	-0.40	-0.21	-0.05	-0.22	0.07	0.98	-0.42	-0.24	0.02	-0.24	0.08	0.97
818	CAMK2G	-0.07	0.17	0.45	0.17	0.08	0.91	-0.02	0.18	0.35	0.18	0.07	0.94	-0.05	0.18	0.42	0.19	0.08	0.92
1002	CDH4	-0.37	-0.15	0.01	-0.16	0.07	0.92	-0.30	-0.16	-0.01	-0.16	0.06	0.95	-0.33	-0.17	0.04	-0.17	0.06	0.95
1006	CDH8	-0.39	-0.20	0.04	-0.20	0.07	0.95	-0.36	-0.21	-0.05	-0.21	0.06	0.97	-0.42	-0.22	-0.01	-0.22	0.07	0.97
1027	CDKN1B	-0.01	0.14	0.34	0.14	0.05	0.91	0.00	0.14	0.29	0.14	0.05	0.96	-0.02	0.15	0.28	0.15	0.06	0.93
1073	CFL2	0.00	0.14	0.32	0.14	0.05	0.92	0.03	0.15	0.27	0.15	0.05	0.94	-0.04	0.15	0.31	0.16	0.06	0.93
1272	CNTN1	-0.43	-0.16	0.05	-0.16	0.07	0.91	-0.34	-0.17	0.00	-0.17	0.07	0.96	-0.37	-0.17	0.03	-0.18	0.08	0.91
1294	COL7A1	-0.01	0.15	0.39	0.15	0.06	0.91	0.02	0.17	0.29	0.16	0.06	0.96	-0.03	0.16	0.34	0.17	0.07	0.91
1300	COL10A1	-0.33	-0.17	-0.02	-0.17	0.05	0.96	-0.30	-0.19	-0.06	-0.18	0.05	0.99	-0.30	-0.19	-0.04	-0.19	0.05	0.99
1501	CTNND2	-0.37	-0.15	0.03	-0.16	0.07	0.90	-0.30	-0.16	-0.01	-0.16	0.06	0.96	-0.35	-0.17	-0.02	-0.18	0.07	0.93
1522	CTSZ	-0.02	0.17	0.37	0.17	0.07	0.93	0.00	0.15	0.36	0.16	0.06	0.96	-0.09	0.18	0.38	0.18	0.07	0.95
1607	DGKB	-0.36	-0.16	0.01	-0.16	0.07	0.90	-0.31	-0.16	-0.02	-0.16	0.06	0.92	-0.40	-0.18	0.03	-0.18	0.07	0.92
1755	DMBT1	0.02	0.12	0.20	0.12	0.04	0.93	0.04	0.13	0.21	0.13	0.04	0.96	-0.02	0.14	0.23	0.14	0.04	0.97
1893	ECM1	-0.04	0.19	0.44	0.19	0.08	0.93	0.03	0.19	0.38	0.19	0.06	0.99	-0.06	0.19	0.38	0.20	0.08	0.94
2027	ENO3	-0.01	0.12	0.22	0.12	0.04	0.91	0.01	0.13	0.20	0.13	0.04	0.93	-0.06	0.13	0.24	0.13	0.04	0.90
2044	EPHA5	-0.31	-0.16	-0.05	-0.16	0.05	0.98	-0.33	-0.16	-0.06	-0.16	0.05	0.99	-0.32	-0.18	0.03	-0.18	0.05	0.98
2070	EYA4	-0.02	0.18	0.42	0.18	0.07	0.93	-0.02	0.18	0.38	0.19	0.07	0.96	-0.01	0.20	0.42	0.20	0.08	0.95
2101	ESRRA	-0.05	0.19	0.51	0.19	0.09	0.92	-0.01	0.21	0.39	0.20	0.08	0.95	-0.04	0.21	0.43	0.21	0.09	0.95
2104	ESRRG	-0.04	0.21	0.56	0.21	0.09	0.94	-0.03	0.22	0.43	0.22	0.08	0.96	-0.04	0.22	0.48	0.23	0.10	0.95
2161	F12	-0.45	-0.17	0.05	-0.18	0.08	0.93	-0.38	-0.19	-0.01	-0.20	0.07	0.97	-0.41	-0.20	-0.01	-0.21	0.08	0.94
2322	FLT3	-0.03	0.16	0.37	0.16	0.07	0.93	0.02	0.16	0.35	0.16	0.06	0.95	-0.08	0.16	0.33	0.17	0.07	0.92
2560	GABRB1	-0.46	-0.17	0.04	-0.18	0.08	0.91	-0.35	-0.18	0.03	-0.18	0.07	0.93	-0.42	-0.19	0.04	-0.19	0.09	0.91
2562	GABRB3	-0.31	-0.14	0.01	-0.14	0.05	0.91	-0.26	-0.13	-0.01	-0.13	0.05	0.92	-0.32	-0.15	0.05	-0.15	0.06	0.93
2565	GABRG1	-0.33	-0.15	0.02	-0.15	0.06	0.91	-0.27	-0.15	-0.04	-0.15	0.05	0.93	-0.33	-0.16	0.06	-0.16	0.06	0.94
2634	GBP2	0.00	0.13	0.25	0.13	0.05	0.90	0.04	0.14	0.22	0.14	0.04	0.97	-0.01	0.15	0.24	0.14	0.04	0.97
2742	GLRA2	-0.45	-0.17	0.03	-0.18	0.08	0.91	-0.36	-0.19	0.00	-0.19	0.07	0.96	-0.42	-0.20	0.08	-0.20	0.09	0.95
2766	GMPR	-0.01	0.15	0.34	0.16	0.06	0.93	0.04	0.15	0.29	0.16	0.05	0.96	-0.03	0.16	0.32	0.16	0.06	0.95
2823	GPM6A	-0.36	-0.16	0.02	-0.16	0.06	0.94	-0.29	-0.15	-0.04	-0.16	0.05	0.96	-0.33	-0.18	0.03	-0.18	0.06	0.95
2830	GPR6	-0.35	-0.19	-0.02	-0.19	0.06	0.98	-0.35	-0.19	-0.08	-0.19	0.06	1.00	-0.36	-0.20	0.07	-0.20	0.06	0.98
2911	GRM1	-0.37	-0.16	0.03	-0.16	0.07	0.92	-0.32	-0.16	-0.01	-0.16	0.06	0.95	-0.36	-0.18	0.05	-0.18	0.07	0.93
2937	GSS	-0.36	-0.14	0.05	-0.14	0.06	0.91	-0.29	-0.14	0.01	-0.15	0.06	0.90	-0.31	-0.16	0.00	-0.17	0.06	0.95
2952	GSTT1	-0.05	0.20	0.47	0.20	0.08	0.94	0.03	0.21	0.40	0.21	0.07	0.99	-0.04	0.22	0.44	0.22	0.09	0.96
3067	HDC	-0.43	-0.17	0.04	-0.17	0.07	0.95	-0.33	-0.19	0.04	-0.19	0.07	0.97	-0.37	-0.20	0.02	-0.20	0.07	0.97
3290	HSD11B1	-0.01	0.16	0.35	0.16	0.07	0.94	0.04	0.19	0.30	0.18	0.06	0.94	-0.01	0.19	0.35	0.20	0.07	0.97
3316	HSPB2	-0.03	0.15	0.34	0.15	0.06	0.90	-0.01	0.14	0.29	0.14	0.06	0.92	-0.05	0.16	0.33	0.16	0.06	0.93
3350	HTR1A	-0.41	-0.16	0.04	-0.17	0.07	0.91	-0.34	-0.16	0.04	-0.16	0.07	0.94	-0.35	-0.17	0.08	-0.17	0.08	0.91
3624	INHBA	0.02	0.15	0.33	0.16	0.05	0.96	-0.03	0.16	0.29	0.16	0.05	0.97	0.04	0.18	0.31	0.18	0.05	0.98
3679	ITGA7	-0.04	0.14	0.24	0.14	0.05	0.94	0.02	0.15	0.28	0.16	0.04	0.98	0.01	0.17	0.27	0.17	0.04	0.98
3688	ITGB1	0.00	0.13	0.24	0.13	0.04	0.93	0.04	0.15	0.22	0.15	0.04	0.96	0.02	0.15	0.27	0.15	0.04	0.97
3736	KCNA1	-0.09	0.18	0.49	0.18	0.09	0.91	0.00	0.19	0.39	0.19	0.07	0.95	-0.06	0.20	0.42	0.20	0.09	0.94
3738	KCNA3	-0.45	-0.17	0.08	-0.18	0.09	0.91	-0.36	-0.18	0.04	-0.18	0.08	0.95	-0.49	-0.20	0.01	-0.20	0.09	0.92
3741	KCNA5	-0.45	-0.16	0.07	-0.17	0.09	0.90	-0.36	-0.18	0.03	-0.19	0.08	0.92	-0.44	-0.19	0.09	-0.19	0.09	0.93
3755	KCNG1	-0.52	-0.19	0.06	-0.20	0.09	0.92	-0.42	-0.21	-0.02	-0.21	0.08	0.96	-0.43	-0.21	0.07	-0.22	0.09	0.94
3783	KCNN4	-0.02	0.17	0.33	0.17	0.06	0.95	0.02	0.18	0.29	0.17	0.05	0.96	-0.02	0.17	0.33	0.17	0.06	0.95
3910	LAMA4	-0.04	0.17	0.38	0.18	0.07	0.93	0.04	0.19	0.32	0.19	0.06	0.96	0.01	0.19	0.41	0.19	0.07	0.95
4062	LY6H	-0.54	-0.19	0.03	-0.20	0.09	0.93	-0.43	-0.20	0.02	-0.21	0.08	0.97	-0.48	-0.22	0.02	-0.23	0.10	0.94
4082	MARCKS	-0.43	-0.18	0.02	-0.18	0.07	0.92	-0.35	-0.18	-0.01	-0.18	0.07	0.94	-0.39	-0.20	0.09	-0.20	0.08	0.95
4258	MGST2	-0.03	0.20	0.45	0.20	0.08	0.94	0.03	0.20	0.38	0.21	0.07	0.98	-0.01	0.21	0.43	0.22	0.08	0.97
4286	MITF	0.00	0.17	0.34	0.17	0.06	0.96	0.05	0.18	0.29	0.18	0.06	0.97	0.02	0.19	0.35	0.19	0.06	0.97
4324	MMP15	-0.05	0.15	0.33	0.15	0.06	0.90	0.00	0.16	0.28	0.15	0.05	0.94	-0.04	0.16	0.35	0.16	0.06	0.92
4345	CD200	-0.25	-0.13	-0.02	-0.13	0.04	0.92	-0.26	-0.13	-0.03	-0.13	0.04	0.96	-0.26	-0.14	0.07	-0.14	0.05	0.93
4354	MPP1	-0.08	0.17	0.44	0.17	0.08	0.90	0.00	0.18	0.36	0.18	0.07	0.97	-0.06	0.19	0.40	0.19	0.08	0.92
4599	MX1	-0.04	0.18	0.40	0.18	0.08	0.93	0.01	0.18	0.36	0.18	0.07	0.97	-0.06	0.21	0.38	0.21	0.07	0.96
4609	MYC	-0.05	0.19	0.38	0.19	0.07	0.96	0.05	0.19	0.37	0.19	0.06	0.97	0.00	0.20	0.36	0.20	0.07	0.97
4638	MYLK	0.02	0.15	0.26	0.15	0.04	0.97	0.04	0.15	0.25	0.15	0.04	0.97	0.01	0.16	0.27	0.16	0.05	0.97
4703	NEB	-0.02	0.21	0.44	0.20	0.08	0.96	0.01	0.22	0.40	0.22	0.07	0.99	0.02	0.22	0.45	0.23	0.08	0.97
4744	NEFH	-0.12	0.18	0.41	0.18	0.08	0.91	0.03	0.18	0.40	0.18	0.07	0.97	-0.10	0.20	0.39	0.19	0.08	0.94
4751	NEK2	-0.43	-0.19	0.05	-0.19	0.08	0.95	-0.38	-0.21	-0.03	-0.21	0.07	0.97	-0.45	-0.22	0.02	-0.22	0.08	0.96
4856	NOV	-0.45	-0.17	0.07	-0.18	0.09	0.90	-0.40	-0.19	0.02	-0.20	0.08	0.96	-0.48	-0.21	0.04	-0.21	0.09	0.93
4886	NPY1R	-0.49	-0.18	0.04	-0.18	0.08	0.94	-0.33	-0.17	0.06	-0.18	0.08	0.95	-0.40	-0.19	0.04	-0.19	0.08	0.93
4889	NPY5R	-0.27	-0.14	0.00	-0.14	0.05	0.93	-0.24	-0.15	-0.02	-0.14	0.05	0.95	-0.28	-0.16	-0.01	-0.15	0.05	0.96
5091	PC	-0.02	0.15	0.30	0.15	0.06	0.92	0.02	0.13	0.29	0.14	0.05	0.92	-0.07	0.15	0.31	0.15	0.06	0.93
5116	PCNT	-0.06	0.17	0.40	0.17	0.08	0.90	0.01	0.18	0.36	0.19	0.07	0.97	-0.04	0.18	0.43	0.19	0.08	0.93
5121	PCP4	-0.08	0.16	0.38	0.16	0.08	0.91	0.02	0.16	0.34	0.17	0.06	0.92	-0.11	0.18	0.36	0.18	0.07	0.95
5138	PDE2A	-0.39	-0.16	0.03	-0.16	0.07	0.90	-0.33	-0.15	-0.01	-0.15	0.06	0.91	-0.35	-0.17	0.10	-0.17	0.07	0.92
5141	PDE4A	-0.06	0.19	0.46	0.19	0.08	0.92	0.01	0.20	0.38	0.20	0.07	0.97	-0.08	0.20	0.42	0.20	0.09	0.94
5176	SERPINF1	-0.35	-0.19	-0.02	-0.19	0.06	0.97	-0.32	-0.20	0.01	-0.20	0.06	0.98	-0.35	-0.22	-0.04	-0.21	0.06	0.98
5409	PNMT	-0.51	-0.20	0.01	-0.21	0.08	0.94	-0.40	-0.20	-0.01	-0.20	0.08	0.96	-0.44	-0.22	0.10	-0.22	0.09	0.95
5412	UBL3	-0.01	0.16	0.35	0.16	0.06	0.93	0.00	0.17	0.31	0.16	0.06	0.95	-0.05	0.17	0.35	0.17	0.07	0.95
5547	PRCP	-0.02	0.13	0.29	0.13	0.05	0.90	0.02	0.13	0.28	0.14	0.05	0.93	-0.05	0.15	0.28	0.15	0.06	0.92
5569	PKIA	-0.51	-0.18	0.04	-0.19	0.09	0.91	-0.40	-0.19	0.00	-0.20	0.08	0.95	-0.46	-0.21	0.04	-0.21	0.10	0.93
5579	PRKCB	-0.34	-0.14	0.05	-0.15	0.06	0.90	-0.29	-0.13	-0.02	-0.14	0.06	0.91	-0.33	-0.16	0.02	-0.16	0.07	0.92
5580	PRKCD	-0.54	-0.19	0.06	-0.20	0.09	0.92	-0.44	-0.20	0.02	-0.21	0.08	0.96	-0.48	-0.22	0.02	-0.22	0.10	0.94
5582	PRKCG	-0.46	-0.18	0.03	-0.18	0.08	0.91	-0.39	-0.19	-0.01	-0.19	0.08	0.96	-0.43	-0.20	0.04	-0.20	0.09	0.92
5733	PTGER3	-0.56	-0.18	0.05	-0.19	0.09	0.91	-0.37	-0.18	0.02	-0.18	0.08	0.95	-0.40	-0.19	0.10	-0.20	0.09	0.91
5774	PTPN3	-0.29	-0.15	-0.04	-0.15	0.05	0.96	-0.26	-0.16	-0.02	-0.16	0.05	0.97	-0.28	-0.17	-0.05	-0.17	0.04	0.98
5794	PTPRH	0.02	0.14	0.24	0.14	0.04	0.96	0.06	0.16	0.24	0.15	0.04	0.98	-0.06	0.16	0.27	0.16	0.05	0.97
5860	QDPR	0.00	0.12	0.21	0.12	0.04	0.94	0.03	0.13	0.20	0.13	0.04	0.93	-0.01	0.15	0.24	0.15	0.04	0.98
5874	RAB27B	-0.46	-0.19	0.03	-0.19	0.08	0.93	-0.40	-0.21	-0.03	-0.21	0.07	0.97	-0.44	-0.22	-0.01	-0.22	0.09	0.94
5961	PRPH2	-0.34	-0.16	0.01	-0.16	0.06	0.95	-0.32	-0.15	0.06	-0.16	0.06	0.96	-0.32	-0.18	0.05	-0.18	0.06	0.96
5979	RET	-0.05	0.16	0.44	0.17	0.08	0.90	-0.02	0.17	0.35	0.17	0.07	0.93	-0.06	0.17	0.37	0.17	0.08	0.92
5993	RFX5	-0.04	0.16	0.47	0.17	0.07	0.91	-0.04	0.17	0.36	0.17	0.07	0.93	-0.04	0.18	0.38	0.18	0.08	0.93
6017	RLBP1	-0.30	-0.15	0.03	-0.15	0.06	0.91	-0.23	-0.15	-0.06	-0.15	0.04	0.96	-0.29	-0.15	0.04	-0.16	0.06	0.95
6272	SORT1	-0.01	0.15	0.30	0.15	0.06	0.92	0.05	0.17	0.27	0.16	0.05	0.95	-0.03	0.17	0.31	0.17	0.06	0.97
6330	SCN4B	-0.07	0.22	0.49	0.22	0.09	0.96	0.04	0.23	0.45	0.23	0.08	0.99	-0.04	0.25	0.46	0.25	0.09	0.98
6543	SLC8A2	-0.36	-0.16	-0.01	-0.16	0.06	0.95	-0.30	-0.14	-0.02	-0.15	0.05	0.95	-0.32	-0.17	0.02	-0.17	0.05	0.96
6646	SOAT1	-0.02	0.17	0.39	0.17	0.07	0.91	-0.01	0.16	0.34	0.17	0.06	0.95	-0.03	0.18	0.36	0.18	0.07	0.94
6804	STX1A	-0.38	-0.20	-0.01	-0.20	0.06	0.97	-0.36	-0.19	-0.05	-0.19	0.07	0.96	-0.38	-0.22	-0.05	-0.22	0.06	0.98
6843	VAMP1	-0.07	0.19	0.53	0.19	0.09	0.91	-0.02	0.20	0.40	0.20	0.08	0.95	-0.06	0.21	0.44	0.21	0.09	0.93
6890	TAP1	-0.01	0.17	0.32	0.16	0.06	0.94	0.04	0.18	0.32	0.18	0.06	0.98	0.04	0.20	0.36	0.19	0.06	0.97
6905	TBCE	-0.04	0.17	0.45	0.18	0.08	0.91	0.02	0.17	0.33	0.17	0.07	0.96	-0.04	0.17	0.39	0.18	0.08	0.91
7062	TCHH	-0.10	0.14	0.36	0.15	0.06	0.91	-0.01	0.15	0.30	0.15	0.06	0.93	-0.06	0.15	0.34	0.15	0.07	0.91
7068	THRB	-0.33	-0.15	0.00	-0.15	0.06	0.91	-0.28	-0.16	-0.04	-0.17	0.05	0.97	-0.33	-0.17	0.00	-0.17	0.06	0.94
7089	TLE2	-0.05	0.16	0.40	0.16	0.07	0.90	0.00	0.17	0.34	0.17	0.07	0.95	-0.01	0.17	0.41	0.18	0.07	0.92
7092	TLL1	-0.32	-0.16	-0.01	-0.16	0.06	0.93	-0.33	-0.15	0.07	-0.15	0.06	0.94	-0.33	-0.17	0.10	-0.18	0.06	0.95
7103	TSPAN8	0.01	0.15	0.27	0.14	0.05	0.94	0.04	0.15	0.23	0.15	0.04	0.97	-0.02	0.16	0.27	0.16	0.05	0.96
7138	TNNT1	-0.41	-0.17	0.05	-0.17	0.08	0.91	-0.36	-0.17	0.00	-0.17	0.06	0.96	-0.39	-0.19	0.07	-0.19	0.08	0.93
7145	TNS1	-0.01	0.13	0.24	0.13	0.04	0.91	0.02	0.13	0.23	0.13	0.04	0.91	0.01	0.15	0.28	0.15	0.05	0.95
7480	WNT10B	-0.47	-0.16	0.06	-0.17	0.08	0.91	-0.36	-0.18	0.03	-0.18	0.07	0.96	-0.42	-0.19	0.02	-0.19	0.09	0.93
7534	YWHAZ	-0.31	-0.15	-0.02	-0.15	0.05	0.93	-0.29	-0.15	-0.02	-0.15	0.05	0.94	-0.33	-0.17	0.04	-0.16	0.06	0.94
7781	SLC30A3	-0.33	-0.19	-0.01	-0.19	0.06	0.98	-0.32	-0.18	-0.04	-0.18	0.06	0.97	-0.34	-0.20	-0.02	-0.20	0.06	0.97
8001	GLRA3	-0.41	-0.17	0.04	-0.17	0.08	0.91	-0.33	-0.18	0.03	-0.18	0.07	0.93	-0.43	-0.19	0.05	-0.19	0.08	0.93
8209	C21orf33	0.01	0.14	0.29	0.14	0.05	0.93	0.02	0.15	0.26	0.15	0.05	0.95	0.00	0.16	0.28	0.16	0.05	0.97
8482	SEMA7A	-0.07	0.17	0.52	0.18	0.09	0.90	-0.04	0.19	0.40	0.19	0.08	0.95	-0.04	0.20	0.45	0.20	0.10	0.92
8611	PLPP1	0.02	0.14	0.29	0.15	0.05	0.93	0.07	0.15	0.25	0.15	0.04	1.00	-0.03	0.16	0.29	0.16	0.05	0.96
8704	B4GALT2	-0.44	-0.18	0.04	-0.19	0.08	0.93	-0.34	-0.19	0.00	-0.19	0.07	0.94	-0.41	-0.20	0.02	-0.21	0.08	0.97
8715	NOL4	-0.44	-0.17	0.03	-0.18	0.07	0.94	-0.34	-0.18	0.02	-0.18	0.07	0.96	-0.40	-0.19	0.06	-0.20	0.08	0.95
8717	TRADD	0.00	0.13	0.24	0.13	0.05	0.92	0.04	0.14	0.23	0.14	0.04	0.91	-0.04	0.14	0.27	0.14	0.05	0.93
8792	TNFRSF11A	-0.39	-0.17	0.03	-0.17	0.07	0.93	-0.34	-0.18	0.02	-0.18	0.07	0.95	-0.41	-0.20	0.03	-0.20	0.07	0.95
8871	SYNJ2	0.00	0.13	0.21	0.12	0.04	0.93	0.01	0.14	0.22	0.14	0.04	0.91	0.02	0.14	0.25	0.14	0.04	0.96
8884	SLC5A6	-0.04	0.16	0.42	0.16	0.07	0.90	0.02	0.18	0.34	0.18	0.06	0.97	-0.03	0.18	0.38	0.19	0.08	0.93
8938	BAIAP3	-0.59	-0.21	0.05	-0.22	0.10	0.93	-0.45	-0.22	0.04	-0.23	0.09	0.97	-0.55	-0.24	0.03	-0.24	0.11	0.95
9033	PKD2L1	-0.39	-0.19	-0.01	-0.18	0.07	0.95	-0.34	-0.19	-0.06	-0.19	0.06	0.96	-0.38	-0.20	0.00	-0.20	0.07	0.97
9127	P2RX6	-0.01	0.19	0.44	0.20	0.08	0.94	-0.02	0.20	0.39	0.20	0.07	0.97	-0.08	0.21	0.42	0.21	0.09	0.95
9196	KCNAB3	-0.06	0.18	0.50	0.18	0.09	0.91	-0.02	0.19	0.40	0.19	0.08	0.96	-0.04	0.19	0.44	0.20	0.09	0.91
9256	TSPOAP1	-0.35	-0.16	0.01	-0.17	0.07	0.93	-0.31	-0.17	-0.04	-0.16	0.05	0.97	-0.36	-0.18	0.07	-0.18	0.07	0.95
9312	KCNB2	-0.34	-0.16	0.00	-0.16	0.06	0.91	-0.31	-0.18	-0.03	-0.18	0.06	0.93	-0.37	-0.18	0.02	-0.18	0.06	0.97
9362	CPNE6	-0.53	-0.18	0.05	-0.18	0.09	0.90	-0.39	-0.19	-0.01	-0.18	0.08	0.95	-0.41	-0.19	0.08	-0.20	0.09	0.92
9454	HOMER3	-0.03	0.14	0.32	0.14	0.06	0.91	0.00	0.15	0.28	0.15	0.05	0.94	0.00	0.17	0.33	0.17	0.06	0.95
9473	THEMIS2	0.02	0.16	0.30	0.16	0.05	0.93	0.05	0.16	0.26	0.15	0.05	0.96	-0.10	0.17	0.32	0.17	0.06	0.96
9495	AKAP5	-0.29	-0.14	0.01	-0.13	0.05	0.90	-0.26	-0.14	-0.03	-0.14	0.05	0.95	-0.26	-0.15	0.06	-0.14	0.05	0.92

9607	CARTPT	-0.45	-0.22	0.00	-0.21	0.09	0.93	-0.42	-0.23	0.04	-0.23	0.08	0.98	-0.43	-0.24	0.02	-0.24	0.08	0.98
9609	RAB36	-0.47	-0.17	0.04	-0.17	0.08	0.93	-0.34	-0.18	0.00	-0.18	0.07	0.98	-0.37	-0.19	0.00	-0.20	0.08	0.95
9636	ISG15	-0.36	-0.16	0.03	-0.16	0.07	0.92	-0.36	-0.17	0.00	-0.17	0.05	0.98	-0.37	-0.19	0.06	-0.19	0.06	0.97
9653	HS2ST1	-0.24	-0.12	0.02	-0.12	0.04	0.93	-0.21	-0.13	-0.02	-0.13	0.04	0.90	-0.24	-0.14	0.04	-0.14	0.04	0.95
9731	CEP104	-0.03	0.12	0.23	0.12	0.04	0.90	0.01	0.13	0.22	0.12	0.04	0.94	0.01	0.13	0.24	0.13	0.04	0.92
9854	C2CD2L	-0.28	-0.14	-0.01	-0.14	0.05	0.94	-0.28	-0.13	-0.03	-0.13	0.05	0.95	-0.27	-0.15	0.04	-0.16	0.05	0.95
9911	TMCC2	-0.04	0.17	0.40	0.17	0.07	0.95	0.02	0.17	0.33	0.17	0.06	0.96	-0.06	0.19	0.35	0.19	0.07	0.95
10083	USH1C	0.02	0.14	0.25	0.14	0.05	0.94	0.02	0.15	0.25	0.14	0.05	0.96	0.00	0.16	0.27	0.15	0.05	0.96
10154	PLXNC1	-0.40	-0.16	0.03	-0.17	0.07	0.93	-0.35	-0.18	-0.03	-0.18	0.06	0.97	-0.35	-0.18	0.04	-0.19	0.07	0.95
10160	FARP1	-0.48	-0.18	0.04	-0.19	0.08	0.92	-0.36	-0.19	0.01	-0.19	0.08	0.96	-0.41	-0.20	0.06	-0.21	0.09	0.93
10231	RCAN2	-0.08	0.18	0.42	0.18	0.08	0.93	0.04	0.19	0.39	0.19	0.07	0.98	-0.06	0.20	0.38	0.19	0.08	0.94
10268	RAMP3	0.02	0.17	0.32	0.17	0.06	0.93	0.03	0.16	0.32	0.17	0.05	0.96	-0.09	0.18	0.34	0.18	0.06	0.97
10332	CLEC4M	-0.03	0.13	0.30	0.13	0.05	0.91	0.00	0.14	0.26	0.14	0.05	0.91	-0.02	0.14	0.29	0.14	0.05	0.93
10368	CACNG3	-0.34	-0.15	0.04	-0.15	0.07	0.90	-0.31	-0.15	-0.01	-0.16	0.06	0.94	-0.35	-0.17	0.02	-0.17	0.07	0.92
10384	BTN3A3	-0.01	0.14	0.28	0.14	0.05	0.92	0.01	0.14	0.26	0.14	0.05	0.94	-0.01	0.16	0.28	0.16	0.05	0.97
10395	DLC1	-0.01	0.14	0.27	0.14	0.05	0.91	0.02	0.14	0.26	0.14	0.05	0.93	-0.05	0.16	0.29	0.16	0.06	0.95
10451	VAV3	-0.05	0.19	0.51	0.20	0.09	0.92	-0.03	0.21	0.41	0.21	0.09	0.96	-0.06	0.20	0.51	0.21	0.10	0.94
10505	SEMA4F	-0.38	-0.16	0.02	-0.17	0.07	0.91	-0.31	-0.16	0.00	-0.17	0.07	0.95	-0.38	-0.17	0.03	-0.18	0.07	0.93
10669	CGREF1	-0.40	-0.16	0.00	-0.17	0.06	0.93	-0.33	-0.17	-0.02	-0.18	0.06	0.95	-0.33	-0.19	0.06	-0.19	0.06	0.97
10673	TNFSF13B	-0.28	-0.14	0.01	-0.14	0.05	0.93	-0.24	-0.14	-0.02	-0.14	0.05	0.93	-0.26	-0.15	-0.01	-0.15	0.05	0.96
10683	DLL3	-0.37	-0.15	0.02	-0.16	0.07	0.92	-0.29	-0.16	-0.02	-0.16	0.06	0.95	-0.37	-0.18	0.09	-0.18	0.07	0.95
10776	ARPP19	-0.39	-0.16	0.05	-0.16	0.06	0.93	-0.30	-0.15	-0.03	-0.15	0.06	0.93	-0.33	-0.17	0.02	-0.17	0.06	0.92
10783	NEK6	-0.25	-0.14	0.00	-0.14	0.04	0.93	-0.24	-0.14	-0.04	-0.14	0.04	0.96	-0.27	-0.15	-0.02	-0.15	0.04	0.96
10891	PPARGC1A	-0.06	0.18	0.47	0.19	0.08	0.92	0.01	0.19	0.41	0.19	0.07	0.97	-0.05	0.20	0.39	0.20	0.08	0.94
11069	RAPGEF4	-0.40	-0.16	0.03	-0.16	0.07	0.91	-0.33	-0.18	-0.04	-0.18	0.06	0.97	-0.36	-0.19	-0.01	-0.19	0.07	0.94
11138	TBC1D8	-0.03	0.16	0.35	0.16	0.07	0.92	0.03	0.16	0.30	0.16	0.05	0.96	-0.04	0.17	0.34	0.17	0.06	0.94
11151	CORO1A	-0.34	-0.14	0.03	-0.15	0.06	0.90	-0.29	-0.15	0.01	-0.15	0.06	0.94	-0.31	-0.16	0.00	-0.16	0.06	0.92
11164	NUDT5	-0.01	0.15	0.35	0.16	0.06	0.92	0.02	0.16	0.29	0.16	0.06	0.98	-0.07	0.18	0.34	0.18	0.07	0.95
11259	FILIP1L	-0.30	-0.16	0.00	-0.16	0.06	0.96	-0.30	-0.18	-0.02	-0.17	0.06	0.95	-0.31	-0.19	-0.05	-0.19	0.05	0.98
22801	ITGA11	-0.03	0.20	0.38	0.19	0.07	0.94	0.05	0.21	0.38	0.21	0.07	0.99	-0.01	0.21	0.41	0.21	0.07	0.97
22881	ANKRD6	-0.53	-0.18	0.05	-0.18	0.09	0.91	-0.39	-0.19	0.03	-0.19	0.08	0.95	-0.46	-0.20	0.06	-0.21	0.10	0.92
22987	SV2C	-0.07	0.20	0.53	0.21	0.08	0.95	0.01	0.21	0.44	0.21	0.08	0.96	-0.05	0.22	0.44	0.22	0.09	0.96
22996	TTC39A	-0.09	0.17	0.37	0.17	0.07	0.94	0.02	0.16	0.32	0.16	0.06	0.96	-0.05	0.18	0.35	0.18	0.07	0.95
23046	KIF21B	-0.37	-0.16	0.04	-0.16	0.06	0.93	-0.30	-0.16	0.00	-0.16	0.06	0.95	-0.33	-0.17	0.00	-0.17	0.06	0.96
23109	DDN	-0.33	-0.16	0.01	-0.16	0.06	0.93	-0.29	-0.15	-0.04	-0.15	0.05	0.96	-0.33	-0.17	0.02	-0.17	0.07	0.95
23180	RFTN1	-0.34	-0.16	0.00	-0.16	0.06	0.92	-0.29	-0.15	-0.01	-0.15	0.06	0.92	-0.32	-0.17	-0.01	-0.17	0.06	0.95
23274	CLEC16A	-0.07	0.16	0.44	0.16	0.08	0.90	-0.04	0.17	0.36	0.17	0.07	0.95	-0.01	0.17	0.39	0.18	0.08	0.92
23406	COTL1	-0.01	0.16	0.27	0.15	0.05	0.94	0.02	0.13	0.30	0.14	0.05	0.92	-0.06	0.16	0.31	0.16	0.05	0.96
23484	LEPROTL1	-0.04	0.17	0.41	0.18	0.07	0.93	0.01	0.18	0.38	0.18	0.07	0.97	-0.03	0.19	0.39	0.19	0.07	0.95
23504	RIMBP2	-0.33	-0.15	0.02	-0.15	0.06	0.90	-0.26	-0.14	-0.01	-0.15	0.05	0.93	-0.31	-0.17	0.02	-0.17	0.07	0.93
23642	SNHG1	-0.05	0.17	0.43	0.17	0.08	0.93	0.01	0.17	0.33	0.18	0.07	0.95	-0.03	0.18	0.42	0.18	0.08	0.91
25841	ABTB2	-0.01	0.19	0.41	0.20	0.07	0.96	0.04	0.21	0.37	0.21	0.07	0.98	0.04	0.23	0.44	0.23	0.07	0.98
25854	FAM149A	-0.41	-0.15	0.04	-0.16	0.07	0.90	-0.32	-0.17	-0.01	-0.17	0.06	0.97	-0.37	-0.18	0.01	-0.18	0.08	0.93
25871	NEPRO	-0.33	-0.14	0.01	-0.15	0.06	0.91	-0.30	-0.15	0.04	-0.15	0.06	0.94	-0.29	-0.16	0.03	-0.16	0.06	0.92
25924	MYRIP	-0.35	-0.15	0.00	-0.15	0.06	0.91	-0.31	-0.16	-0.05	-0.16	0.05	0.94	-0.33	-0.18	0.01	-0.18	0.06	0.95
25989	ULK3	-0.04	0.18	0.41	0.18	0.08	0.92	0.01	0.20	0.37	0.20	0.07	0.98	-0.01	0.20	0.42	0.20	0.08	0.95
26010	SPATS2L	-0.22	-0.13	-0.02	-0.13	0.04	0.93	-0.19	-0.12	-0.03	-0.12	0.04	0.91	-0.22	-0.13	0.01	-0.12	0.04	0.96
26059	ERC2	-0.36	-0.15	0.02	-0.15	0.06	0.90	-0.32	-0.16	-0.01	-0.16	0.06	0.90	-0.34	-0.17	0.09	-0.18	0.07	0.95
27077	B9D1	-0.51	-0.17	0.04	-0.18	0.08	0.91	-0.35	-0.18	0.00	-0.18	0.07	0.96	-0.40	-0.19	0.06	-0.20	0.08	0.93
27132	CPNE7	-0.49	-0.18	0.03	-0.18	0.08	0.93	-0.37	-0.19	-0.01	-0.19	0.07	0.96	-0.42	-0.21	0.02	-0.21	0.08	0.94
27163	NAAA	-0.04	0.17	0.40	0.17	0.07	0.93	0.00	0.19	0.33	0.18	0.06	0.97	0.00	0.19	0.38	0.19	0.07	0.95
27294	DHDH	-0.38	-0.16	0.02	-0.16	0.06	0.93	-0.32	-0.16	-0.02	-0.16	0.06	0.96	-0.35	-0.18	0.07	-0.18	0.07	0.95
28955	DEXI	-0.05	0.17	0.45	0.17	0.07	0.92	0.00	0.18	0.38	0.18	0.07	0.96	-0.04	0.19	0.37	0.19	0.08	0.93
29799	YPEL1	-0.41	-0.17	0.06	-0.17	0.08	0.91	-0.35	-0.18	-0.02	-0.17	0.07	0.94	-0.40	-0.19	0.08	-0.19	0.08	0.94
29803	REPIN1	-0.02	0.17	0.40	0.17	0.07	0.93	0.01	0.18	0.34	0.18	0.06	0.98	0.00	0.19	0.34	0.19	0.07	0.96
30850	CDR2L	-0.02	0.18	0.43	0.19	0.08	0.93	0.02	0.19	0.38	0.20	0.07	0.98	-0.02	0.21	0.44	0.21	0.08	0.96
50486	G0S2	-0.01	0.17	0.31	0.17	0.06	0.96	0.03	0.17	0.35	0.17	0.05	0.99	-0.05	0.19	0.36	0.19	0.06	0.97
50853	VILL	-0.05	0.15	0.31	0.15	0.06	0.91	0.02	0.15	0.34	0.15	0.06	0.97	-0.12	0.16	0.32	0.16	0.07	0.92
51059	FAM135B	-0.07	0.15	0.29	0.15	0.06	0.92	0.03	0.15	0.34	0.15	0.05	0.93	-0.08	0.15	0.32	0.15	0.06	0.94
51074	APIP	0.00	0.14	0.29	0.15	0.05	0.93	0.02	0.15	0.26	0.15	0.05	0.96	-0.01	0.16	0.30	0.16	0.06	0.95
51134	CEP83	-0.30	-0.14	0.06	-0.14	0.06	0.90	-0.27	-0.17	-0.03	-0.16	0.05	0.96	-0.30	-0.17	0.01	-0.16	0.06	0.95
51312	SLC25A37	-0.07	0.17	0.54	0.18	0.09	0.90	-0.05	0.19	0.41	0.19	0.08	0.95	-0.05	0.19	0.44	0.20	0.09	0.91
51375	SNX7	-0.50	-0.19	0.05	-0.19	0.09	0.93	-0.40	-0.20	-0.01	-0.21	0.08	0.98	-0.46	-0.22	0.04	-0.22	0.09	0.94
51393	TRPV2	-0.30	-0.14	0.01	-0.14	0.06	0.91	-0.26	-0.14	0.03	-0.14	0.05	0.93	-0.27	-0.15	0.00	-0.15	0.05	0.92
51440	HPCAL4	-0.42	-0.15	0.03	-0.16	0.08	0.90	-0.33	-0.17	-0.03	-0.17	0.07	0.94	-0.37	-0.18	0.06	-0.18	0.08	0.92
51454	GULP1	-0.40	-0.17	0.04	-0.17	0.08	0.90	-0.35	-0.18	0.00	-0.18	0.07	0.93	-0.41	-0.19	0.06	-0.19	0.08	0.95
51522	TMEM14C	-0.04	0.15	0.39	0.15	0.07	0.90	0.00	0.15	0.33	0.16	0.07	0.90	-0.04	0.17	0.37	0.17	0.07	0.92
51660	MPC1	0.00	0.18	0.41	0.18	0.07	0.94	0.02	0.18	0.35	0.18	0.07	0.95	-0.05	0.19	0.38	0.19	0.07	0.96
51667	NUB1	-0.05	0.15	0.40	0.15	0.07	0.91	0.03	0.15	0.30	0.16	0.06	0.92	-0.01	0.16	0.35	0.17	0.06	0.94
54112	GPR88	-0.42	-0.18	0.04	-0.18	0.07	0.94	-0.36	-0.19	-0.05	-0.19	0.06	0.98	-0.37	-0.20	0.06	-0.20	0.08	0.96
54492	NEURL1B	-0.42	-0.18	0.10	-0.18	0.07	0.93	-0.34	-0.18	0.02	-0.17	0.07	0.93	-0.38	-0.19	0.08	-0.19	0.08	0.95
54536	EXOC6	-0.42	-0.18	0.02	-0.18	0.07	0.95	-0.34	-0.18	-0.01	-0.18	0.07	0.96	-0.36	-0.20	0.04	-0.20	0.07	0.96
54550	NECAB2	-0.48	-0.19	0.03	-0.19	0.09	0.91	-0.37	-0.19	0.00	-0.19	0.08	0.95	-0.44	-0.21	0.09	-0.21	0.09	0.95
54551	MAGEL2	-0.05	0.16	0.41	0.16	0.07	0.91	-0.03	0.17	0.32	0.16	0.06	0.93	-0.07	0.17	0.35	0.17	0.07	0.92
54566	EPB41L4B	-0.30	-0.15	0.02	-0.15	0.06	0.90	-0.28	-0.15	0.01	-0.15	0.05	0.93	-0.31	-0.17	0.06	-0.16	0.06	0.94
54793	KCTD9	-0.07	0.18	0.46	0.19	0.08	0.93	0.02	0.18	0.40	0.18	0.07	0.96	-0.08	0.20	0.39	0.20	0.08	0.95
54843	SYTL2	-0.33	-0.16	0.02	-0.16	0.06	0.93	-0.29	-0.16	-0.02	-0.16	0.05	0.96	-0.32	-0.18	0.03	-0.18	0.06	0.95
54847	SIDT1	-0.33	-0.15	0.04	-0.15	0.06	0.92	-0.28	-0.15	0.02	-0.15	0.06	0.93	-0.34	-0.17	0.00	-0.17	0.06	0.95
54972	TMEM132A	-0.32	-0.14	0.00	-0.15	0.05	0.93	-0.27	-0.13	-0.01	-0.13	0.05	0.90	-0.27	-0.15	0.07	-0.15	0.06	0.94
55040	EPN3	-0.12	0.19	0.43	0.19	0.08	0.93	0.02	0.19	0.41	0.19	0.07	0.97	-0.05	0.20	0.41	0.20	0.08	0.94
55160	ARHGEF10L	-0.03	0.16	0.39	0.16	0.07	0.92	-0.01	0.16	0.32	0.16	0.06	0.95	-0.03	0.17	0.38	0.17	0.07	0.90
55244	SLC47A1	-0.02	0.24	0.46	0.24	0.08	0.99	0.09	0.24	0.46	0.25	0.07	1.00	-0.05	0.25	0.44	0.25	0.08	0.98
55315	SLC29A3	-0.35	-0.17	-0.01	-0.17	0.06	0.96	-0.31	-0.17	-0.02	-0.18	0.05	0.97	-0.33	-0.19	0.02	-0.19	0.06	0.98
55353	LAPTM4B	-0.09	0.16	0.43	0.16	0.08	0.90	0.01	0.17	0.36	0.17	0.07	0.95	-0.05	0.18	0.39	0.18	0.08	0.91
55509	BATF3	-0.43	-0.18	0.05	-0.18	0.08	0.92	-0.38	-0.19	-0.03	-0.19	0.07	0.97	-0.43	-0.20	0.01	-0.20	0.08	0.94
55591	VEZT	-0.37	-0.16	0.01	-0.16	0.06	0.94	-0.29	-0.16	-0.03	-0.16	0.06	0.97	-0.33	-0.17	0.03	-0.17	0.07	0.93
55686	MREG	-0.09	0.17	0.35	0.17	0.06	0.94	0.05	0.16	0.34	0.17	0.05	0.97	-0.05	0.18	0.33	0.17	0.06	0.98
55800	SCN3B	-0.52	-0.20	0.02	-0.21	0.08	0.94	-0.40	-0.21	-0.01	-0.21	0.08	0.97	-0.45	-0.22	0.03	-0.23	0.09	0.95
55853	IDI2-AS1	-0.04	0.16	0.42	0.17	0.07	0.93	0.02	0.17	0.35	0.18	0.07	0.94	-0.05	0.18	0.38	0.18	0.08	0.91
55897	MESP1	-0.46	-0.19	0.03	-0.19	0.08	0.95	-0.36	-0.19	-0.01	-0.20	0.07	0.98	-0.39	-0.21	0.03	-0.21	0.08	0.96
56477	CCL28	-0.26	-0.13	0.00	-0.13	0.05	0.92	-0.26	-0.14	-0.03	-0.14	0.05	0.91	-0.26	-0.15	0.01	-0.15	0.05	0.96
56934	CA10	-0.41	-0.18	0.07	-0.18	0.08	0.94	-0.36	-0.19	-0.01	-0.19	0.07	0.97	-0.42	-0.20	0.05	-0.19	0.08	0.94
56937	PMEPA1	-0.05	0.18	0.39	0.18	0.07	0.94	0.03	0.20	0.35	0.20	0.06	0.98	-0.03	0.20	0.38	0.20	0.07	0.96
56967	C14orf132	-0.32	-0.17	0.02	-0.17	0.06	0.95	-0.29	-0.15	-0.05	-0.15	0.05	0.97	-0.34	-0.17	0.04	-0.17	0.05	0.98
57110	HRASLS	-0.39	-0.16	0.06	-0.16	0.07	0.91	-0.33	-0.17	-0.05	-0.18	0.06	0.97	-0.34	-0.19	0.03	-0.19	0.06	0.96
57453	DSCAML1	-0.03	0.15	0.31	0.15	0.06	0.91	0.05	0.17	0.29	0.17	0.05	0.98	-0.02	0.18	0.35	0.17	0.06	0.96
57495	NWD2	-0.38	-0.20	-0.02	-0.20	0.06	0.97	-0.36	-0.19	-0.03	-0.20	0.06	0.99	-0.38	-0.21	0.02	-0.21	0.07	0.98
57496	MKL2	-0.37	-0.18	0.00	-0.18	0.07	0.96	-0.33	-0.18	-0.01	-0.18	0.07	0.96	-0.37	-0.19	0.03	-0.20	0.07	0.95
57526	PCDH19	-0.42	-0.20	0.01	-0.20	0.08	0.97	-0.37	-0.20	-0.04	-0.21	0.07	0.98	-0.42	-0.22	0.01	-0.22	0.08	0.97
57554	LRRC7	-0.28	-0.14	0.01	-0.14	0.05	0.91	-0.29	-0.13	-0.03	-0.13	0.05	0.91	-0.32	-0.14	0.08	-0.14	0.06	0.92
57718	PPP4R4	-0.49	-0.17	0.07	-0.18	0.09	0.91	-0.39	-0.19	0.01	-0.19	0.08	0.95	-0.45	-0.19	0.05	-0.20	0.09	0.92
57761	TRIB3	-0.30	-0.14	-0.01	-0.14	0.05	0.93	-0.24	-0.14	-0.01	-0.13	0.05	0.92	-0.27	-0.15	0.04	-0.15	0.05	0.95
64132	XYLT2	-0.05	0.16	0.40	0.16	0.07	0.93	-0.01	0.18	0.36	0.17	0.07	0.96	0.01	0.18	0.39	0.18	0.07	0.93
64135	IFIH1	0.02	0.14	0.28	0.14	0.05	0.91	0.01	0.15	0.26	0.15	0.05	0.96	0.02	0.16	0.30	0.16	0.06	0.95
64149	C17orf75	-0.04	0.16	0.40	0.16	0.06	0.93	-0.02	0.15	0.34	0.16	0.06	0.90	-0.04	0.16	0.33	0.17	0.07	0.93
65078	RTN4R	-0.29	-0.16	-0.05	-0.16	0.04	0.98	-0.26	-0.15	-0.03	-0.14	0.04	0.96	-0.25	-0.16	0.03	-0.16	0.04	0.98
65997	RASL11B	-0.41	-0.19	0.01	-0.19	0.07	0.96	-0.34	-0.18	-0.01	-0.19	0.07	0.97	-0.39	-0.21	-0.02	-0.21	0.07	0.98
66000	TMEM108	-0.40	-0.16	0.04	-0.16	0.07	0.91	-0.31	-0.16	0.00	-0.17	0.06	0.95	-0.38	-0.18	0.00	-0.18	0.07	0.94
66008	TRAK2	-0.02	0.16	0.35	0.16	0.06	0.94	0.01	0.16	0.30	0.17	0.06	0.96	0.00	0.18	0.34	0.18	0.06	0.95
79012	CAMKV	-0.38	-0.17	0.00	-0.17	0.07	0.93	-0.34	-0.16	0.01	-0.16	0.06	0.96	-0.35	-0.18	0.10	-0.18	0.07	0.95
79017	GGCT	-0.46	-0.16	0.06	-0.17	0.08	0.91	-0.36	-0.17	0.01	-0.18	0.07	0.96	-0.40	-0.19	0.03	-0.19	0.08	0.92
79183	TTPAL	-0.30	-0.15	0.04	-0.15	0.06	0.91	-0.29	-0.17	-0.06	-0.17	0.05	0.96	-0.31	-0.18	0.06	-0.18	0.06	0.96
79645	EFCAB1	-0.52	-0.17	0.05	-0.18	0.09	0.90	-0.39	-0.19	0.04	-0.19	0.08	0.96	-0.45	-0.19	0.06	-0.20	0.10	0.92
79660	PPP1R3B	0.00	0.16	0.32	0.16	0.05	0.96	0.07	0.16	0.27	0.16	0.04	1.00	-0.04	0.17	0.30	0.17	0.05	0.98
79720	VPS37B	-0.29	-0.15	0.02	-0.15	0.05	0.94	-0.31	-0.14	-0.04	-0.14	0.05	0.94	-0.30	-0.15	0.05	-0.15	0.05	0.95
79745	CLIP4	-0.01	0.15	0.31	0.15	0.06	0.93	0.02	0.16	0.29	0.15	0.05	0.94	0.00	0.16	0.30	0.17	0.06	0.96
79750	ZNF385D	-0.02	0.16	0.33	0.16	0.06	0.91	0.02	0.15	0.33	0.15	0.06	0.96	-0.06	0.15	0.30	0.15	0.06	0.94
79822	ARHGAP28	-0.40	-0.16	0.05	-0.17	0.07	0.93	-0.33	-0.18	0.00	-0.18	0.07	0.95	-0.40	-0.18	0.01	-0.19	0.07	0.94
79958	DENND1C	-0.27	-0.13	0.04	-0.13	0.05	0.92	-0.23	-0.15	-0.03	-0.14	0.04	0.95	-0.29	-0.14	-0.01	-0.14	0.05	0.95
79993	ELOVL7	-0.02	0.14	0.30	0.14	0.05	0.91	0.04	0.15	0.27	0.15	0.05	0.96	0.00	0.16	0.29	0.16	0.05	0.95
80020	FOXRED2	-0.38	-0.20	0.00	-0.20	0.07	0.96	-0.37	-0.20	-0.04	-0.20	0.06	0.98	-0.37	-0.22	-0.03	-0.22	0.06	0.99
80119	PIF1	-0.03	0.20	0.47	0.20	0.09	0.93	0.01	0.21	0.40	0.21	0.08	0.97	-0.01	0.22	0.43	0.22	0.09	0.95
80176	SPSB1	-0.02	0.15	0.29	0.15	0.05	0.94	0.02	0.16	0.26	0.16	0.05	0.98	-0.05	0.17	0.31	0.17	0.06	0.97
80307	FER1L4	-0.01	0.22	0.48	0.22	0.08	0.95	0.03	0.23	0.40	0.23	0.07	0.98	-0.02	0.24	0.45	0.24	0.09	0.96
80323	CCDC68	-0.39	-0.17	0.06	-0.17	0.07	0.96	-0.33	-0.18	-0.02	-0.19	0.07	0.95	-0.38	-0.20	0.01	-0.20	0.07	0.98
80774	LIMD2	-0.25	-0.14	0.00	-0.14	0.04	0.95	-0.29	-0.15	-0.03	-0.14	0.05	0.96	-0.29	-0.15	-0.02	-0.15	0.04	0.97
80820	EEPD1	-0.08	0.18	0.50	0.19	0.09	0.91	-0.02	0.20	0.42	0.21	0.08	0.97	-0.06	0.21	0.48	0.21	0.10	0.91
80854	SETD7	-0.01	0.17	0.39	0.17	0.07	0.94	-0.02	0.17	0.32	0.17	0.06	0.95	0.00	0.18	0.38	0.18	0.07	0.95
81033	KCNH6	-0.09	0.17	0.42	0.17	0.08	0.91	-0.01	0.17	0.35	0.17	0.07	0.95	-0.06	0.18	0.39	0.18	0.08	0.93
81849	ST6GALNAC5	-0.44	-0.19	0.05	-0.20	0.08	0.95	-0.36	-0.22	-0.02	-0.22	0.07	0.98	-0.46	-0.22	-0.01	-0.22	0.08	0.97
83445	GSG1	-0.48	-0.16	0.07	-0.17	0.08	0.91	-0.35	-0.19	0.03	-0.18	0.08	0.94	-0.44	-0.19	0.06	-0.20	0.09	0.94
83468	GLT8D2	-0.35	-0.16	-0.01	-0.16	0.06	0.94	-0.34	-0.17	-0.03	-0.17	0.06	0.96	-0.33	-0.19	0.06	-0.19	0.06	0.97
83660	TLN2	-0.38	-0.19	-0.04	-0.18	0.06	0.96	-0.32	-0.19	-0.04	-0.19	0.06	0.98	-0.36	-0.21	-0.01	-0.21	0.06	0.98
83690	CRISPLD1	-0.05	0.15	0.31	0.15	0.06	0.91	-0.06	0.14	0.27	0.14	0.05	0.94	-0.01	0.16	0.34	0.16	0.06	0.94
83714	NRIP2	-0.05	0.14	0.30	0.14	0.06	0.92	0.00	0.14	0.26	0.14	0.05	0.96	-0.01	0.16	0.31	0.16	0.06	0.95
83787	ARMC10	-0.33	-0.14	0.03	-0.15	0.06	0.92	-0.29	-0.16	0.00	-0.15	0.06	0.93	-0.32	-0.17	0.03	-0.17	0.07	0.91
83875	BCO2	-0.04	0.16	0.39	0.16	0.06	0.94	0.02	0.15	0.29	0.16	0.06	0.95	-0.03	0.17	0.36	0.17	0.07	0.93
84034	EMILIN2	0.04	0.19	0.34	0.19	0.06	0.98	0.09	0.21	0.34	0.21	0.05	1.00	0.01	0.21	0.35	0.22	0.06	0.98
84109	QRFPR	-0.05	0.16	0.40	0.16	0.08	0.92	0.00	0.17	0.36	0.17	0.07	0.95	-0.05	0.17	0.40	0.17	0.08	0.92
84187	TMEM164	-0.35	-0.15	0.04	-0.16	0.07	0.91	-0.29	-0.17	-0.05	-0.16	0.05	0.99	-0.35	-0.17	0.04	-0.17	0.07	0.93
84221	SPATC1L	-0.35	-0.15	0.04	-0.16	0.06	0.92	-0.28	-0.16	0.00	-0.16	0.06	0.95	-0.32	-0.18	0.00	-0.18	0.06	0.95
84439	HHIPL1	-0.39	-0.17	0.01	-0.18	0.07	0.92	-0.32	-0.18	-0.02	-0.18	0.06	0.96	-0.36	-0.18	0.06	-0.18	0.07	0.94
84525	HOPX	-0.29	-0.15	0.04	-0.15	0.05	0.93	-0.26	-0.15	-0.02	-0.15	0.05	0.94	-0.29	-0.16	0.02	-0.16	0.06	0.94
84623	KIRREL3	-0.33	-0.17	0.01	-0.17	0.07	0.92	-0.30	-0.16	0.03	-0.16	0.06	0.92	-0.35	-0.18	0.06	-0.18	0.06	0.97
84769	MPV17L2	-0.40	-0.17	0.01	-0.17	0.06	0.95	-0.31	-0.17	-0.03	-0.17	0.06	0.97	-0.31	-0.19	-0.01	-0.18	0.06	0.96

84812	PLCD4	-0.03	0.17	0.42	0.17	0.08	0.90	-0.01	0.18	0.35	0.18	0.07	0.95	-0.02	0.18	0.43	0.19	0.08	0.93
84937	ZNRF1	-0.32	-0.16	-0.01	-0.16	0.05	0.98	-0.29	-0.17	-0.07	-0.18	0.05	0.99	-0.30	-0.18	0.01	-0.18	0.05	0.98
85301	COL27A1	-0.06	0.13	0.38	0.13	0.06	0.90	-0.04	0.15	0.28	0.15	0.06	0.91	-0.05	0.15	0.29	0.15	0.06	0.94
85352	SHISAL1	-0.41	-0.16	-0.01	-0.17	0.07	0.93	-0.31	-0.17	-0.01	-0.18	0.06	0.98	-0.35	-0.18	0.01	-0.19	0.07	0.95
90102	PHLDB2	-0.03	0.14	0.29	0.14	0.05	0.91	-0.03	0.15	0.27	0.15	0.05	0.97	-0.05	0.16	0.28	0.15	0.06	0.94
90523	MLIP	-0.28	-0.16	-0.04	-0.16	0.05	0.97	-0.29	-0.16	-0.02	-0.16	0.05	0.94	-0.34	-0.19	-0.01	-0.19	0.05	0.99
90850	ZNF598	-0.02	0.14	0.29	0.14	0.05	0.93	0.03	0.15	0.26	0.16	0.05	0.97	0.01	0.16	0.28	0.16	0.05	0.95
90861	JPT2	0.02	0.14	0.23	0.14	0.04	0.97	0.03	0.15	0.23	0.15	0.04	0.97	0.00	0.16	0.26	0.15	0.04	0.98
91133	L3MBTL4	-0.30	-0.15	0.02	-0.15	0.06	0.90	-0.27	-0.16	-0.03	-0.16	0.05	0.98	-0.32	-0.17	0.01	-0.16	0.06	0.94
91252	SLC39A13	-0.04	0.18	0.48	0.18	0.08	0.92	0.00	0.20	0.38	0.19	0.07	0.96	-0.03	0.20	0.41	0.20	0.08	0.94
91624	NEXN	-0.01	0.18	0.42	0.18	0.07	0.94	0.03	0.18	0.34	0.19	0.06	0.96	-0.01	0.20	0.41	0.20	0.07	0.95
92335	STRADA	-0.05	0.15	0.34	0.15	0.06	0.91	0.05	0.15	0.28	0.15	0.05	0.94	-0.02	0.16	0.32	0.16	0.06	0.92
113675	SDSL	-0.03	0.16	0.37	0.16	0.07	0.91	0.00	0.16	0.35	0.16	0.06	0.94	-0.07	0.18	0.35	0.18	0.08	0.93
114571	SLC22A9	-0.34	-0.15	0.00	-0.15	0.06	0.94	-0.29	-0.14	0.00	-0.14	0.05	0.95	-0.36	-0.15	0.05	-0.16	0.06	0.93
114787	GPRIN1	-0.38	-0.17	0.00	-0.17	0.06	0.94	-0.33	-0.17	-0.03	-0.17	0.06	0.95	-0.34	-0.19	0.05	-0.19	0.07	0.96
114800	CCDC85A	-0.28	-0.12	0.01	-0.13	0.05	0.92	-0.21	-0.13	-0.02	-0.12	0.04	0.90	-0.25	-0.13	0.05	-0.12	0.04	0.91
114990	VASN	-0.28	-0.15	0.01	-0.15	0.05	0.95	-0.29	-0.15	-0.03	-0.15	0.05	0.97	-0.29	-0.17	0.04	-0.17	0.05	0.98
116028	RMI2	-0.31	-0.15	0.02	-0.15	0.06	0.93	-0.28	-0.14	0.00	-0.15	0.05	0.95	-0.29	-0.17	0.04	-0.17	0.06	0.95
116135	LRRC3B	-0.46	-0.18	0.04	-0.18	0.09	0.90	-0.37	-0.19	-0.01	-0.19	0.07	0.95	-0.44	-0.20	0.05	-0.20	0.09	0.92
118427	OLFM3	-0.34	-0.15	0.08	-0.15	0.07	0.91	-0.30	-0.17	-0.04	-0.17	0.06	0.93	-0.36	-0.18	0.04	-0.18	0.07	0.96
119587	CPXM2	0.00	0.14	0.29	0.14	0.05	0.93	0.04	0.14	0.27	0.15	0.04	0.94	-0.06	0.15	0.29	0.15	0.05	0.96
127833	SYT2	-0.05	0.22	0.50	0.22	0.09	0.94	0.02	0.22	0.45	0.23	0.08	0.98	-0.04	0.24	0.47	0.24	0.09	0.96
128434	VSTM2L	-0.39	-0.16	0.09	-0.17	0.08	0.92	-0.33	-0.18	-0.02	-0.18	0.07	0.96	-0.36	-0.19	0.02	-0.18	0.08	0.93
130399	ACVR1C	-0.03	0.18	0.42	0.18	0.07	0.94	0.02	0.20	0.40	0.19	0.06	0.97	-0.02	0.20	0.38	0.20	0.07	0.95
132160	PPM1M	-0.48	-0.17	0.03	-0.17	0.08	0.91	-0.35	-0.18	0.02	-0.18	0.07	0.97	-0.40	-0.20	0.05	-0.19	0.09	0.91
132321	C4orf33	-0.05	0.17	0.41	0.18	0.08	0.91	0.00	0.18	0.37	0.18	0.07	0.96	-0.03	0.20	0.43	0.20	0.08	0.93
134548	SOWAHA	-0.39	-0.18	0.01	-0.18	0.07	0.95	-0.34	-0.18	-0.01	-0.18	0.06	0.98	-0.37	-0.19	0.10	-0.19	0.08	0.95
139221	MUM1L1	-0.44	-0.17	0.08	-0.18	0.08	0.93	-0.37	-0.20	0.01	-0.19	0.08	0.95	-0.46	-0.20	0.06	-0.20	0.09	0.94
140733	MACROD2	-0.25	-0.13	0.02	-0.13	0.05	0.91	-0.26	-0.13	0.05	-0.13	0.05	0.95	-0.31	-0.16	0.01	-0.16	0.06	0.94
144348	ZNF664	0.02	0.14	0.24	0.14	0.04	0.95	-0.01	0.13	0.24	0.13	0.04	0.94	-0.02	0.14	0.23	0.14	0.04	0.95
144402	CPNE8	-0.33	-0.17	-0.02	-0.17	0.06	0.96	-0.33	-0.18	-0.05	-0.18	0.06	0.97	-0.33	-0.19	-0.01	-0.19	0.06	0.97
147463	ANKRD29	-0.06	0.17	0.44	0.17	0.08	0.91	-0.03	0.16	0.36	0.17	0.07	0.92	-0.06	0.18	0.38	0.18	0.08	0.93
147968	CAPN12	-0.31	-0.17	0.01	-0.16	0.06	0.94	-0.29	-0.18	-0.03	-0.17	0.06	0.95	-0.33	-0.19	-0.03	-0.19	0.05	0.98
148014	TTC9B	-0.25	-0.13	-0.01	-0.13	0.04	0.92	-0.25	-0.13	-0.02	-0.13	0.04	0.94	-0.26	-0.14	0.03	-0.14	0.05	0.95
149473	CCDC24	-0.26	-0.13	0.01	-0.13	0.05	0.91	-0.25	-0.13	0.02	-0.12	0.05	0.90	-0.26	-0.14	0.02	-0.14	0.05	0.92
151516	ASPRV1	0.01	0.12	0.22	0.12	0.04	0.91	0.03	0.13	0.21	0.13	0.04	0.92	0.01	0.13	0.23	0.13	0.04	0.93
152940	C4orf45	-0.33	-0.16	0.04	-0.16	0.06	0.93	-0.29	-0.17	-0.03	-0.17	0.05	0.96	-0.33	-0.18	0.00	-0.18	0.06	0.97
160760	PPTC7	-0.07	0.17	0.47	0.17	0.08	0.94	0.02	0.18	0.37	0.17	0.07	0.96	-0.08	0.19	0.38	0.19	0.08	0.94
162494	RHBDL3	-0.05	0.19	0.55	0.19	0.09	0.92	-0.03	0.19	0.40	0.20	0.08	0.95	-0.05	0.21	0.47	0.21	0.09	0.93
163183	SYNE4	-0.06	0.17	0.45	0.17	0.08	0.91	-0.03	0.18	0.39	0.18	0.07	0.96	-0.05	0.19	0.46	0.19	0.09	0.90
163732	CITED4	-0.29	-0.13	0.02	-0.13	0.05	0.92	-0.24	-0.13	0.00	-0.12	0.05	0.92	-0.26	-0.14	-0.02	-0.14	0.05	0.95
163782	KANK4	0.03	0.20	0.35	0.20	0.06	0.98	0.07	0.20	0.32	0.21	0.05	0.99	-0.03	0.22	0.37	0.22	0.06	0.99
165215	FAM171B	-0.38	-0.15	0.02	-0.16	0.07	0.90	-0.31	-0.15	-0.01	-0.16	0.06	0.95	-0.36	-0.17	0.08	-0.17	0.08	0.91
167691	LCA5	-0.05	0.16	0.42	0.16	0.07	0.90	0.01	0.16	0.35	0.16	0.07	0.93	-0.04	0.18	0.40	0.18	0.07	0.92
192668	CYS1	-0.26	-0.15	0.00	-0.15	0.05	0.94	-0.24	-0.14	-0.02	-0.14	0.04	0.94	-0.29	-0.17	0.10	-0.17	0.05	0.98
196383	RILPL2	-0.51	-0.18	0.03	-0.19	0.09	0.93	-0.37	-0.20	0.01	-0.19	0.07	0.96	-0.42	-0.20	0.06	-0.21	0.09	0.93
200058	FLJ23867	-0.04	0.17	0.42	0.17	0.07	0.91	0.00	0.16	0.34	0.17	0.07	0.94	-0.05	0.18	0.39	0.18	0.08	0.92
200942	KLHDC8B	-0.48	-0.16	0.05	-0.17	0.08	0.91	-0.35	-0.17	0.05	-0.17	0.07	0.94	-0.39	-0.18	0.05	-0.19	0.08	0.91
203286	ANKS6	-0.02	0.15	0.41	0.16	0.07	0.91	0.02	0.16	0.32	0.16	0.06	0.96	-0.04	0.17	0.33	0.17	0.07	0.91
221294	NT5DC1	0.01	0.17	0.35	0.17	0.06	0.96	0.07	0.18	0.29	0.18	0.05	1.00	-0.02	0.19	0.34	0.19	0.06	0.98
221421	RSPH9	-0.49	-0.16	0.03	-0.17	0.08	0.91	-0.34	-0.17	0.01	-0.17	0.07	0.95	-0.39	-0.18	0.10	-0.18	0.09	0.91
222537	HS3ST5	-0.03	0.18	0.40	0.19	0.07	0.94	0.04	0.20	0.35	0.20	0.06	0.98	-0.04	0.20	0.39	0.21	0.07	0.98
253832	ZDHHC20	0.01	0.14	0.26	0.14	0.05	0.93	0.03	0.15	0.25	0.15	0.04	0.95	-0.02	0.16	0.27	0.16	0.05	0.96
254102	EHBP1L1	-0.35	-0.20	0.01	-0.20	0.06	0.98	-0.33	-0.20	-0.07	-0.20	0.05	0.99	-0.37	-0.22	-0.02	-0.22	0.06	0.99
260434	PYDC1	-0.42	-0.16	0.03	-0.17	0.08	0.91	-0.34	-0.17	-0.04	-0.18	0.06	0.96	-0.36	-0.19	0.05	-0.19	0.08	0.94
282969	FUOM	-0.34	-0.16	0.00	-0.16	0.06	0.95	-0.28	-0.17	-0.07	-0.17	0.05	0.98	-0.30	-0.18	0.02	-0.18	0.06	0.95
283209	PGM2L1	-0.43	-0.16	0.03	-0.16	0.07	0.92	-0.33	-0.16	0.01	-0.17	0.07	0.94	-0.34	-0.18	0.01	-0.18	0.07	0.93
283284	IGSF22	-0.35	-0.15	0.03	-0.16	0.06	0.92	-0.30	-0.16	-0.03	-0.17	0.06	0.95	-0.35	-0.18	0.06	-0.18	0.06	0.96
284348	LYPD5	-0.05	0.18	0.51	0.18	0.09	0.91	-0.03	0.18	0.39	0.18	0.08	0.94	-0.06	0.19	0.41	0.19	0.09	0.92
285780	LY86-AS1	-0.36	-0.17	0.05	-0.17	0.07	0.92	-0.34	-0.18	0.01	-0.18	0.07	0.94	-0.38	-0.19	-0.01	-0.19	0.07	0.93
286133	SCARA5	-0.53	-0.20	0.06	-0.20	0.09	0.93	-0.39	-0.23	0.01	-0.22	0.08	0.96	-0.47	-0.23	0.04	-0.24	0.09	0.96
326624	RAB37	-0.05	0.19	0.45	0.19	0.09	0.92	0.01	0.20	0.40	0.20	0.07	0.96	-0.07	0.20	0.41	0.21	0.09	0.95
342667	STAC2	-0.03	0.20	0.49	0.20	0.09	0.94	0.03	0.21	0.42	0.21	0.08	0.97	-0.08	0.22	0.43	0.22	0.09	0.96
348013	TMEM255B	-0.03	0.18	0.38	0.18	0.07	0.94	0.05	0.19	0.33	0.19	0.06	0.98	-0.02	0.20	0.39	0.20	0.07	0.96
349136	WDR86	-0.53	-0.21	0.03	-0.22	0.09	0.94	-0.43	-0.23	-0.01	-0.24	0.08	0.99	-0.49	-0.24	0.03	-0.24	0.10	0.96
373156	GSTK1	-0.03	0.14	0.32	0.13	0.05	0.90	0.02	0.14	0.27	0.14	0.05	0.94	0.01	0.16	0.29	0.16	0.05	0.96
374378	GALNT18	-0.27	-0.14	0.02	-0.14	0.05	0.92	-0.24	-0.12	-0.03	-0.13	0.04	0.92	-0.29	-0.14	0.05	-0.14	0.05	0.95
374875	HSD11B1L	-0.36	-0.15	0.04	-0.15	0.07	0.91	-0.30	-0.16	-0.01	-0.16	0.06	0.95	-0.34	-0.17	0.02	-0.17	0.07	0.95
375057	STUM	-0.54	-0.18	0.04	-0.18	0.09	0.90	-0.38	-0.19	0.02	-0.19	0.08	0.94	-0.40	-0.19	0.06	-0.20	0.09	0.92
386618	KCTD4	-0.45	-0.19	0.02	-0.19	0.08	0.92	-0.41	-0.19	0.02	-0.19	0.07	0.97	-0.40	-0.20	0.09	-0.20	0.09	0.95
400745	SH2D5	-0.39	-0.15	0.06	-0.16	0.08	0.91	-0.32	-0.17	-0.01	-0.17	0.07	0.95	-0.40	-0.19	0.03	-0.19	0.07	0.95
404217	CTXN1	-0.45	-0.19	0.03	-0.19	0.08	0.93	-0.40	-0.20	-0.01	-0.20	0.07	0.98	-0.43	-0.22	-0.01	-0.22	0.08	0.95
407738	FAM19A1	-0.33	-0.13	-0.01	-0.14	0.05	0.90	-0.26	-0.14	-0.03	-0.14	0.05	0.91	-0.27	-0.15	0.05	-0.15	0.05	0.94
494470	RNF165	-0.26	-0.13	-0.02	-0.13	0.04	0.93	-0.24	-0.14	-0.02	-0.14	0.04	0.93	-0.28	-0.15	0.05	-0.15	0.05	0.95
503542	SPRN	-0.39	-0.18	0.04	-0.19	0.07	0.96	-0.35	-0.19	-0.02	-0.19	0.07	0.97	-0.38	-0.21	-0.01	-0.21	0.07	0.97
574036	SERTAD4-AS1	-0.04	0.18	0.49	0.19	0.09	0.92	0.00	0.19	0.39	0.19	0.07	0.97	-0.05	0.20	0.41	0.20	0.09	0.93
642273	FAM110C	-0.53	-0.21	0.02	-0.22	0.09	0.95	-0.42	-0.22	0.02	-0.23	0.08	0.98	-0.50	-0.24	0.00	-0.25	0.09	0.97
654502	IQCJ	-0.31	-0.15	0.02	-0.15	0.06	0.91	-0.26	-0.13	0.01	-0.13	0.05	0.93	-0.34	-0.16	0.06	-0.15	0.06	0.93
100507436	MICA	-0.03	0.16	0.32	0.16	0.06	0.94	0.01	0.17	0.30	0.16	0.05	0.96	0.02	0.18	0.35	0.18	0.06	0.97

Abbreviations: rsFC, resting-state functional connectivity; L, left; A1/2/3tru, trunk region of area 1/2/3; CNP, the Consortium for Neuropsychiatric Phenomics; SALD, the Southwest University Adult Lifespan Dataset; Min, minimum; Med, median; Max, maximum; SD, standard deviation.

Genes related to rsFC of the R-A1/2/3tru in both the discovery and validation experiments

GeneID	Genesymbol	Discovery dataset						CNP dataset						SALD dataset					
		r					Percentage	*r*					Percentage	*r*					Percentage
		Min	Med	Max	Mean	SD		Min	Med	Max	Mean	SD		Min	Med	Max	Mean	SD	
814	CAMK4	-0.31	-0.17	0.05	-0.16	0.06	0.91	-0.31	-0.16	0.01	-0.16	0.06	0.93	-0.34	-0.19	0.01	-0.18	0.06	0.96
7781	SLC30A3	-0.32	-0.17	0.07	-0.16	0.07	0.90	-0.33	-0.16	-0.01	-0.16	0.06	0.93	-0.35	-0.18	-0.01	-0.18	0.06	0.95
55244	SLC47A1	-0.09	0.21	0.46	0.21	0.09	0.95	-0.01	0.23	0.45	0.22	0.09	0.96	-0.01	0.23	0.44	0.23	0.09	0.96
84034	EMILIN2	-0.07	0.17	0.34	0.17	0.07	0.93	-0.03	0.19	0.33	0.18	0.07	0.92	0.04	0.20	0.36	0.20	0.06	0.96
163782	KANK4	-0.06	0.17	0.35	0.17	0.07	0.93	-0.08	0.19	0.31	0.18	0.07	0.92	0.03	0.21	0.39	0.21	0.07	0.96
254102	EHBP1L1	-0.34	-0.18	0.05	-0.18	0.07	0.93	-0.31	-0.18	-0.03	-0.18	0.06	0.94	-0.35	-0.21	-0.04	-0.21	0.06	0.98

Abbreviations: rsFC, resting-state functional connectivity; R, right; A1/2/3tru, trunk region of area 1/2/3; CNP, the Consortium for Neuropsychiatric Phenomics; SALD, the Southwest University Adult Lifespan Dataset; Min, minimum; Med, median; Max, maximum; SD, standard deviation.
