# Supplementary material for: Transcriptional substrates underlying functional connectivity profiles of subregions within the human sensorimotor cortex
Source: Hum Brain Mapp. 2022 Jul 27;43(18):5562–78. doi: 10.1002/hbm.26031 (PMC9704778; doi:10.1002/hbm.26031)
Supplement: Supplementary file 1 — Appendix S1 Supplementary Information [file HBM-43-5562-s001.zip › HBM_26031_Supplementary file 3.pdf]

| Tissue-specific expression for the genes related to rsFC of sensorimotor subregions | | | | | | | | |
|---|---|---|---|---|---|---|---|---|
| **Tissue** | **A4hf** | **A6cdl** | **A4ul** | **A4t** | **A4ll** | **A1/2/3ulhf** | **A1/2/3tonIa** | **A1/2/3tru** |
| Adipose Tissue | 0.003 | 0.307 | 0.003 | 0.359 | $9.857\times10^{-4}$ | 0.010 | 0.776 | 0.012 |
| Adrenal Gland | 0.044 | 0.153 | $1.776\times10^{-5}$ | 0.803 | 0.613 | $9.748\times10^{-5}$ | 0.614 | 0.028 |
| Blood | 0.085 | 0.849 | 0.775 | 0.881 | 0.988 | 0.383 | 0.705 | 0.453 |
| Blood Vessel | $3.888\times10^{-8}$ | 0.123 | $7.282\times10^{-6}$ | 0.359 | 0.005 | $5.017\times10^{-8}$ | 0.614 | $1.942\times10^{-5}$ |
| Brain | $2.979\times10^{-20}$ | $1.565\times10^{-5}$ | $2.843\times10^{-40}$ | 0.131 | $9.857\times10^{-4}$ | $4.176\times10^{-35}$ | 0.118 | $2.647\times10^{-22}$ |
| Breast | 0.073 | 0.813 | 0.216 | 1.000 | 1.000 | 0.052 | 1.000 | 0.535 |
| Colon | 0.237 | 0.849 | 0.393 | 1.000 | 0.856 | 0.510 | 1.000 | 0.453 |
| Esophagus | 0.779 | 0.475 | 0.998 | 1.000 | 1.000 | 0.907 | 0.776 | 0.905 |
| Fallopian Tube | 0.022 | 0.502 | $2.877\times10^{-4}$ | 0.359 | 0.662 | $9.748\times10^{-5}$ | 0.614 | 0.012 |
| Heart | 0.044 | 0.108 | 0.006 | 0.359 | 0.821 | 0.010 | 1.000 | 0.028 |
| Kidney | 0.030 | 0.108 | 0.065 | 0.803 | 0.662 | 0.026 | 0.614 | 0.122 |
| Liver | 0.044 | 0.249 | 0.071 | 0.803 | 0.275 | 0.197 | 0.614 | 0.122 |
| Lung | 0.457 | 0.944 | 0.932 | 1.000 | 0.925 | 0.759 | 0.616 | 0.641 |
| Muscle | $7.623\times10^{-5}$ | 0.309 | $8.427\times10^{-5}$ | 0.411 | 0.433 | $1.117\times10^{-4}$ | 1.000 | 0.019 |
| Nerve | 0.008 | 0.123 | 0.007 | 0.151 | 0.103 | 0.014 | 0.614 | 0.173 |
| Ovary | 0.004 | 0.603 | $2.292\times10^{-4}$ | 0.803 | 0.843 | $9.748\times10^{-5}$ | 0.826 | 0.041 |
| Pancreas | 0.498 | 0.475 | 0.496 | 0.803 | 1.000 | 0.844 | 0.826 | 0.905 |
| Pituitary | $1.717\times10^{-7}$ | 0.037 | $1.780\times10^{-13}$ | 0.359 | 0.356 | $1.169\times10^{-12}$ | 0.614 | $5.422\times10^{-8}$ |
| Prostate | 0.031 | 0.123 | 0.130 | 0.803 | 0.662 | 0.038 | 0.776 | 0.024 |
| Skin | 0.418 | 0.249 | 0.411 | 1.000 | 1.000 | 0.383 | 0.830 | 0.453 |
| Stomach | 0.409 | 0.475 | 0.985 | 1.000 | 0.768 | 0.876 | 0.776 | 0.453 |
| Testis | 0.996 | 0.944 | 0.998 | 1.000 | 1.000 | 0.996 | 0.614 | 0.983 |
| Thyroid | 0.136 | 0.126 | 0.100 | 0.803 | 0.595 | 0.197 | 1.000 | 0.122 |
| Uterus | $8.059\times10^{-4}$ | 0.249 | $5.433\times10^{-5}$ | 0.803 | 0.433 | $1.146\times10^{-5}$ | 0.614 | 0.012 |
| Vagina | 0.830 | 0.239 | 0.998 | 1.000 | 1.000 | 0.876 | 1.000 | 0.860 |

he Fisher's exact *P* values corrected by the FDR-BH.
ea 4; A6cdl, caudal dorsolateral area 6; A4ul, upper limb region of area 4; A4t, trunk region of Brodmann area 4;
1/2/3; A1/2/3tonIa, tongue and larynx region of Brodmann area 1/2/3; A1/2/3tru, trunk region of area 1/2/3.

| Cell type-specific expression for the genes related to rsFC of sensorimotor subregions | | | | | | |
|---|---|---|---|---|---|---|
| **Cell types** | **A4hf** | **A6cdl** | **A4ul** | **A4ll** | **A1/2/3ulhf** | **A1/2/3tru** |
| Oligodendrocyte progenitor cells | 0.744 | 0.762 | 0.786 | 1.000 | 0.693 | 0.786 |
| Immune cells | $3.307{\times}10^{-4}$ | 0.259 | 0.001 | 0.991 | $6.806{\times}10^{-4}$ | $1.005{\times}10^{-4}$ |
| Pnoc+ neurons | 0.082 | 1.000 | 0.053 | 1.000 | 0.050 | 0.571 |
| Ntsr+ neurons | $3.903{\times}10^{-4}$ | 1.000 | $1.278{\times}10^{-5}$ | 0.854 | $1.866{\times}10^{-6}$ | $3.801{\times}10^{-5}$ |
| Glt25d2 neurons | $8.280{\times}10^{-6}$ | 0.762 | $4.365{\times}10^{-6}$ | 1.000 | $7.723{\times}10^{-6}$ | $2.482{\times}10^{-5}$ |
| Astrocytes | 0.064 | 0.734 | 0.264 | 1.000 | 0.035 | 0.426 |
| Cort+ neurons | 0.026 | 0.134 | 0.010 | 0.818 | 0.018 | 0.002 |
| Myelinating oligodendrocytes | 0.628 | 0.762 | 0.211 | 0.818 | 0.624 | 0.803 |

The values in the tables are the Fisher's exact *P* values corrected by the FDR-BH.

Abbreviations: rsFC, resting-state functional connectivity; A4hf, head and face region of area 4; A6cdl, caudal dorsolateral area 6; A4ul, upper limb region of area 4; A4ll, lower limb region of area 4; A1/2/3ulhf, upper limb, head and face region of area 1/2/3; A1/2/3tru, trunk region of area 1/2/3.

| Temporal-specific expression for the genes related to rsFC of sensorimotor subregions | | | | | | |
|---|---|---|---|---|---|---|
| **Developmental stages** | **A4hf** | **A6cdl** | **A4ul** | **A4ll** | **A1/2/3ulhf** | **A1/2/3tru** |
| Cortex.Early.Fetal | 1.000 | 0.947 | 1.000 | 0.949 | 1.000 | 1.000 |
| Cortex.Early.Mid.Fetal | 0.259 | 0.534 | 0.828 | 0.513 | 0.255 | 0.719 |
| Cortex.Late.Mid.Fetal | 0.106 | 0.156 | 0.019 | 0.949 | 0.011 | 0.096 |
| Cortex.Late.Fetal | 0.045 | 0.113 | $3.462 \times 10^{-4}$ | 0.949 | 0.003 | 0.019 |
| Cortex.Neonatal.Early.Infancy | $5.799 \times 10^{-6}$ | 0.156 | $3.881 \times 10^{-11}$ | 0.406 | $8.233 \times 10^{-13}$ | $8.629 \times 10^{-12}$ |
| Cortex.Late.Infancy | 0.446 | 0.797 | 0.193 | 0.350 | 0.327 | 0.023 |
| Cortex.Early.Childhood | 0.019 | 0.708 | 0.019 | 0.513 | 0.014 | 0.019 |
| Cortex.Middle.Late.Childhood | $4.265 \times 10^{-4}$ | 0.534 | 0.001 | 0.895 | $3.103 \times 10^{-5}$ | $6.853 \times 10^{-6}$ |
| Cortex.Adolescence | $5.468 \times 10^{-6}$ | 0.094 | $1.645 \times 10^{-7}$ | 0.598 | $7.605 \times 10^{-9}$ | $6.707 \times 10^{-7}$ |
| Cortex.Young.Adulthood | $2.870 \times 10^{-8}$ | 0.002 | $1.543 \times 10^{-12}$ | 0.513 | $7.593 \times 10^{-12}$ | $2.438 \times 10^{-7}$ |

The values in the tables are the Fisher's exact *P* values corrected by the FDR-BH.
Abbreviations: rsFC, resting-state functional connectivity; A4hf, head and face region of area 4; A6cdl, caudal dorsolateral area 6; A4ul, upper limb region of area 4; A4ll, lower limb region of area 4; A1/2/3ulhf, upper limb, head and face region of area 1/2/3; A1/2/3tru, trunk region of area 1/2/3.
