# Supplementary material for: Transcriptional substrates underlying functional connectivity profiles of subregions within the human sensorimotor cortex
Source: Hum Brain Mapp. 2022 Jul 27;43(18):5562–78. doi: 10.1002/hbm.26031 (PMC9704778; doi:10.1002/hbm.26031)
Supplement: Supplementary file 1 — Appendix S1 Supplementary Information [file HBM-43-5562-s001.zip › HBM_26031_Supplementary file 4.pdf]

Enrichment results of the genes related to rsFC of the A4hf				
**Category**	**ID**	**Name**	***P* value**	***q* value (FDR-BH correction)**
GO: Molecular Function	GO:0046873	metal ion transmembrane transporter activity	1.37E-07	1.44E-04
GO: Molecular Function	GO:0022857	transmembrane transporter activity	6.06E-07	3.19E-04
GO: Molecular Function	GO:0022839	ion gated channel activity	1.32E-06	4.06E-04
GO: Molecular Function	GO:0005215	transporter activity	1.54E-06	4.06E-04
GO: Molecular Function	GO:0022836	gated channel activity	2.28E-06	4.38E-04
GO: Molecular Function	GO:0015318	inorganic molecular entity transmembrane transporter activity	2.50E-06	4.38E-04
GO: Molecular Function	GO:0005216	ion channel activity	1.06E-05	1.59E-03
GO: Molecular Function	GO:0015075	ion transmembrane transporter activity	2.36E-05	2.55E-03
GO: Molecular Function	GO:0005261	cation channel activity	2.53E-05	2.55E-03
GO: Molecular Function	GO:0008092	cytoskeletal protein binding	2.65E-05	2.55E-03
GO: Molecular Function	GO:0022890	inorganic cation transmembrane transporter activity	2.67E-05	2.55E-03
GO: Molecular Function	GO:0015267	channel activity	3.15E-05	2.62E-03
GO: Molecular Function	GO:0022803	passive transmembrane transporter activity	3.24E-05	2.62E-03
GO: Molecular Function	GO:0015081	sodium ion transmembrane transporter activity	5.26E-05	3.96E-03
GO: Molecular Function	GO:0008324	cation transmembrane transporter activity	9.60E-05	6.73E-03
GO: Molecular Function	GO:0086006	voltage-gated sodium channel activity involved in cardiac muscle cell action potential	1.27E-04	8.33E-03
GO: Molecular Function	GO:0005272	sodium channel activity	1.36E-04	8.38E-03
GO: Molecular Function	GO:0015077	monovalent inorganic cation transmembrane transporter activity	1.93E-04	1.13E-02
GO: Molecular Function	GO:0017080	sodium channel regulator activity	2.11E-04	1.17E-02
GO: Molecular Function	GO:0016247	channel regulator activity	3.05E-04	1.61E-02
GO: Molecular Function	GO:0005244	voltage-gated ion channel activity	3.55E-04	1.77E-02
GO: Molecular Function	GO:0022832	voltage-gated channel activity	3.70E-04	1.77E-02
GO: Molecular Function	GO:0098634	cell-matrix adhesion mediator activity	4.28E-04	1.96E-02
GO: Molecular Function	GO:0022852	glycine-gated chloride ion channel activity	5.71E-04	2.50E-02
GO: Molecular Function	GO:0019871	sodium channel inhibitor activity	6.73E-04	2.83E-02
GO: Molecular Function	GO:0098960	postsynaptic neurotransmitter receptor activity	8.05E-04	3.24E-02
GO: Molecular Function	GO:0030594	neurotransmitter receptor activity	8.55E-04	3.24E-02
GO: Molecular Function	GO:0043178	alcohol binding	8.63E-04	3.24E-02
GO: Molecular Function	GO:0005227	calcium activated cation channel activity	9.92E-04	3.60E-02
GO: Molecular Function	GO:0005267	potassium channel activity	1.22E-03	4.25E-02
GO: Molecular Function	GO:0015079	potassium ion transmembrane transporter activity	1.25E-03	4.25E-02
GO: Molecular Function	GO:0099095	ligand-gated anion channel activity	1.34E-03	4.41E-02
GO: Molecular Function	GO:0005509	calcium ion binding	1.38E-03	4.41E-02
GO: Molecular Function	GO:0004602	glutathione peroxidase activity	1.61E-03	4.97E-02
GO: Biological Process	GO:0099537	trans-synaptic signaling	7.64E-11	2.10E-07
GO: Biological Process	GO:0099536	synaptic signaling	1.30E-10	2.10E-07
GO: Biological Process	GO:0098916	anterograde trans-synaptic signaling	1.59E-10	2.10E-07
GO: Biological Process	GO:0007268	chemical synaptic transmission	1.59E-10	2.10E-07
GO: Biological Process	GO:0007267	cell-cell signaling	9.18E-09	9.67E-06
GO: Biological Process	GO:0044057	regulation of system process	1.14E-08	1.00E-05
GO: Biological Process	GO:0006811	ion transport	3.43E-08	2.58E-05
GO: Biological Process	GO:0050804	modulation of chemical synaptic transmission	9.24E-08	5.65E-05
GO: Biological Process	GO:0099177	regulation of trans-synaptic signaling	9.65E-08	5.65E-05
GO: Biological Process	GO:0055085	transmembrane transport	3.62E-07	1.79E-04
GO: Biological Process	GO:0034220	ion transmembrane transport	3.73E-07	1.79E-04
GO: Biological Process	GO:0043269	regulation of ion transport	1.67E-06	7.34E-04
GO: Biological Process	GO:0042391	regulation of membrane potential	2.98E-06	1.21E-03
GO: Biological Process	GO:0030001	metal ion transport	3.71E-06	1.40E-03
GO: Biological Process	GO:0098660	inorganic ion transmembrane transport	4.05E-06	1.42E-03
GO: Biological Process	GO:0006812	cation transport	5.35E-06	1.76E-03
GO: Biological Process	GO:0099565	chemical synaptic transmission, postsynaptic	1.27E-05	3.73E-03
GO: Biological Process	GO:0002028	regulation of sodium ion transport	1.27E-05	3.73E-03
GO: Biological Process	GO:0023061	signal release	1.52E-05	4.12E-03
GO: Biological Process	GO:0035637	multicellular organismal signaling	1.56E-05	4.12E-03
GO: Biological Process	GO:1902305	regulation of sodium ion transmembrane transport	1.76E-05	4.22E-03
GO: Biological Process	GO:0003013	circulatory system process	1.76E-05	4.22E-03
GO: Biological Process	GO:0032509	endosome transport via multivesicular body sorting pathway	2.09E-05	4.79E-03
GO: Biological Process	GO:0060078	regulation of postsynaptic membrane potential	2.74E-05	6.01E-03
GO: Biological Process	GO:0008015	blood circulation	3.31E-05	6.94E-03
GO: Biological Process	GO:0016477	cell migration	3.42E-05	6.94E-03
GO: Biological Process	GO:0098662	inorganic cation transmembrane transport	4.12E-05	8.03E-03
GO: Biological Process	GO:0035725	sodium ion transmembrane transport	4.40E-05	8.27E-03
GO: Biological Process	GO:0010765	positive regulation of sodium ion transport	4.76E-05	8.64E-03
GO: Biological Process	GO:0032594	protein transport within lipid bilayer	4.96E-05	8.72E-03
GO: Biological Process	GO:0006814	sodium ion transport	5.77E-05	9.65E-03
GO: Biological Process	GO:0051674	localization of cell	6.04E-05	9.65E-03
GO: Biological Process	GO:0048870	cell motility	6.04E-05	9.65E-03
GO: Biological Process	GO:0003012	muscle system process	7.14E-05	1.11E-02
GO: Biological Process	GO:0030029	actin filament-based process	7.99E-05	1.20E-02
GO: Biological Process	GO:0071248	cellular response to metal ion	1.03E-04	1.51E-02
GO: Biological Process	GO:0071985	multivesicular body sorting pathway	1.13E-04	1.61E-02
GO: Biological Process	GO:0001508	action potential	1.26E-04	1.73E-02
GO: Biological Process	GO:0140352	export from cell	1.28E-04	1.73E-02
GO: Biological Process	GO:0003015	heart process	1.45E-04	1.92E-02
GO: Biological Process	GO:0014888	striated muscle adaptation	1.50E-04	1.93E-02
GO: Biological Process	GO:0010038	response to metal ion	1.74E-04	2.18E-02
GO: Biological Process	GO:0010959	regulation of metal ion transport	1.79E-04	2.20E-02
GO: Biological Process	GO:0050877	nervous system process	2.00E-04	2.38E-02
GO: Biological Process	GO:0071277	cellular response to calcium ion	2.08E-04	2.38E-02
GO: Biological Process	GO:0040012	regulation of locomotion	2.10E-04	2.38E-02
GO: Biological Process	GO:0015672	monovalent inorganic cation transport	2.21E-04	2.38E-02
GO: Biological Process	GO:0098655	cation transmembrane transport	2.21E-04	2.38E-02
GO: Biological Process	GO:0007525	somatic muscle development	2.22E-04	2.38E-02
GO: Biological Process	GO:0031644	regulation of nervous system process	2.26E-04	2.38E-02
GO: Biological Process	GO:2000649	regulation of sodium ion transmembrane transporter activity	2.37E-04	2.45E-02
GO: Biological Process	GO:0006836	neurotransmitter transport	2.42E-04	2.45E-02
GO: Biological Process	GO:0060047	heart contraction	2.95E-04	2.77E-02
GO: Biological Process	GO:0042983	amyloid precursor protein biosynthetic process	2.95E-04	2.77E-02
GO: Biological Process	GO:0042984	regulation of amyloid precursor protein biosynthetic process	2.95E-04	2.77E-02
GO: Biological Process	GO:0030334	regulation of cell migration	2.95E-04	2.77E-02
GO: Biological Process	GO:2000145	regulation of cell motility	2.99E-04	2.77E-02
GO: Biological Process	GO:0120036	plasma membrane bounded cell projection organization	3.21E-04	2.92E-02
GO: Biological Process	GO:0030030	cell projection organization	3.30E-04	2.94E-02
GO: Biological Process	GO:0048666	neuron development	3.35E-04	2.94E-02
GO: Biological Process	GO:0022008	neurogenesis	3.44E-04	2.97E-02
GO: Biological Process	GO:0043270	positive regulation of ion transport	3.76E-04	3.18E-02
GO: Biological Process	GO:0106057	negative regulation of calcineurin-mediated signaling	3.86E-04	3.18E-02
GO: Biological Process	GO:0070885	negative regulation of calcineurin-NFAT signaling cascade	3.86E-04	3.18E-02
GO: Biological Process	GO:0099643	signal release from synapse	4.27E-04	3.41E-02
GO: Biological Process	GO:0007269	neurotransmitter secretion	4.27E-04	3.41E-02
GO: Biological Process	GO:0060079	excitatory postsynaptic potential	4.77E-04	3.73E-02
GO: Biological Process	GO:0046928	regulation of neurotransmitter secretion	4.90E-04	3.73E-02
GO: Biological Process	GO:0044272	sulfur compound biosynthetic process	4.93E-04	3.73E-02
GO: Biological Process	GO:0071241	cellular response to inorganic substance	4.95E-04	3.73E-02
GO: Biological Process	GO:0031175	neuron projection development	5.22E-04	3.78E-02
GO: Biological Process	GO:0051270	regulation of cellular component movement	5.24E-04	3.78E-02
GO: Biological Process	GO:2000184	positive regulation of progesterone biosynthetic process	5.28E-04	3.78E-02
GO: Biological Process	GO:0048699	generation of neurons	5.31E-04	3.78E-02
GO: Biological Process	GO:0061337	cardiac conduction	5.50E-04	3.79E-02
GO: Biological Process	GO:0050982	detection of mechanical stimulus	5.53E-04	3.79E-02
GO: Biological Process	GO:0032940	secretion by cell	5.57E-04	3.79E-02
GO: Biological Process	GO:0009914	hormone transport	5.61E-04	3.79E-02
GO: Biological Process	GO:0030182	neuron differentiation	5.88E-04	3.92E-02
GO: Biological Process	GO:0007155	cell adhesion	6.10E-04	4.02E-02
GO: Biological Process	GO:0006936	muscle contraction	6.18E-04	4.02E-02
GO: Biological Process	GO:0034765	regulation of ion transmembrane transport	6.51E-04	4.18E-02
GO: Biological Process	GO:0022610	biological adhesion	6.79E-04	4.31E-02
GO: Biological Process	GO:1903305	regulation of regulated secretory pathway	6.89E-04	4.32E-02

GO: Biological Process	GO:0001505	regulation of neurotransmitter levels	7.85E-04	4.87E-02
GO: Biological Process	GO:0034762	regulation of transmembrane transport	8.02E-04	4.91E-02
GO: Cellular Component	GO:0043005	neuron projection	2.25E-16	1.31E-13
GO: Cellular Component	GO:0045202	synapse	6.37E-15	1.85E-12
GO: Cellular Component	GO:0030424	axon	4.04E-12	7.83E-10
GO: Cellular Component	GO:0036477	somatodendritic compartment	5.27E-11	7.48E-09
GO: Cellular Component	GO:0097060	synaptic membrane	6.43E-11	7.48E-09
GO: Cellular Component	GO:0098794	postsynapse	5.82E-10	5.65E-08
GO: Cellular Component	GO:0030054	cell junction	1.93E-09	1.58E-07
GO: Cellular Component	GO:0030425	dendrite	2.44E-09	1.58E-07
GO: Cellular Component	GO:0097447	dendritic tree	2.44E-09	1.58E-07
GO: Cellular Component	GO:0098978	glutamatergic synapse	5.54E-09	3.22E-07
GO: Cellular Component	GO:0043025	neuronal cell body	9.77E-09	5.17E-07
GO: Cellular Component	GO:0045211	postsynaptic membrane	1.22E-08	5.92E-07
GO: Cellular Component	GO:0044297	cell body	3.33E-08	1.49E-06
GO: Cellular Component	GO:0005887	integral component of plasma membrane	1.46E-07	6.05E-06
GO: Cellular Component	GO:1902495	transmembrane transporter complex	1.99E-07	7.71E-06
GO: Cellular Component	GO:1990351	transporter complex	3.10E-07	1.13E-05
GO: Cellular Component	GO:0031226	intrinsic component of plasma membrane	4.04E-07	1.38E-05
GO: Cellular Component	GO:0034702	ion channel complex	7.23E-07	2.34E-05
GO: Cellular Component	GO:0034703	cation channel complex	1.36E-06	4.15E-05
GO: Cellular Component	GO:0099240	intrinsic component of synaptic membrane	2.87E-06	8.35E-05
GO: Cellular Component	GO:0098793	presynapse	3.60E-06	9.98E-05
GO: Cellular Component	GO:0099699	integral component of synaptic membrane	3.81E-06	1.01E-04
GO: Cellular Component	GO:0098797	plasma membrane protein complex	1.30E-05	3.30E-04
GO: Cellular Component	GO:0150034	distal axon	1.70E-05	4.02E-04
GO: Cellular Component	GO:0043204	perikaryon	1.73E-05	4.02E-04
GO: Cellular Component	GO:0098982	GABA-ergic synapse	2.64E-05	5.92E-04
GO: Cellular Component	GO:0014704	intercalated disc	1.11E-04	2.40E-03
GO: Cellular Component	GO:0044291	cell-cell contact zone	1.46E-04	3.03E-03
GO: Cellular Component	GO:0034706	sodium channel complex	1.67E-04	3.28E-03
GO: Cellular Component	GO:0042383	sarcolemma	1.69E-04	3.28E-03
GO: Cellular Component	GO:0001518	voltage-gated sodium channel complex	2.10E-04	3.84E-03
GO: Cellular Component	GO:0098936	intrinsic component of postsynaptic membrane	2.11E-04	3.84E-03
GO: Cellular Component	GO:0098984	neuron to neuron synapse	2.49E-04	4.40E-03
GO: Cellular Component	GO:0031594	neuromuscular junction	3.60E-04	6.16E-03
GO: Cellular Component	GO:0034705	potassium channel complex	3.88E-04	6.46E-03
GO: Cellular Component	GO:0044305	calyx of Held	4.24E-04	6.86E-03
GO: Cellular Component	GO:0099055	integral component of postsynaptic membrane	4.74E-04	7.46E-03
GO: Cellular Component	GO:0005911	cell-cell junction	5.11E-04	7.49E-03
GO: Cellular Component	GO:0034681	integrin alpha11-beta1 complex	5.15E-04	7.49E-03
GO: Cellular Component	GO:0034677	integrin alpha7-beta1 complex	5.15E-04	7.49E-03
GO: Cellular Component	GO:0099572	postsynaptic specialization	7.97E-04	1.13E-02
GO: Cellular Component	GO:0015629	actin cytoskeleton	8.25E-04	1.14E-02
GO: Cellular Component	GO:0008076	voltage-gated potassium channel complex	8.74E-04	1.18E-02
GO: Cellular Component	GO:0031902	late endosome membrane	9.55E-04	1.26E-02
GO: Cellular Component	GO:0098796	membrane protein complex	1.27E-03	1.64E-02
GO: Cellular Component	GO:0043197	dendritic spine	1.30E-03	1.65E-02
GO: Cellular Component	GO:0005938	cell cortex	1.39E-03	1.72E-02
GO: Cellular Component	GO:0044309	neuron spine	1.57E-03	1.88E-02
GO: Cellular Component	GO:0005884	actin filament	1.59E-03	1.88E-02
GO: Cellular Component	GO:0014069	postsynaptic density	1.65E-03	1.88E-02
GO: Cellular Component	GO:0032279	asymmetric synapse	1.65E-03	1.88E-02
GO: Cellular Component	GO:0005768	endosome	1.77E-03	1.98E-02
GO: Cellular Component	GO:0030426	growth cone	1.87E-03	2.01E-02
GO: Cellular Component	GO:0043679	axon terminus	1.87E-03	2.01E-02
GO: Cellular Component	GO:0030427	site of polarized growth	2.26E-03	2.40E-02
GO: Cellular Component	GO:0099501	exocytic vesicle membrane	2.42E-03	2.47E-02
GO: Cellular Component	GO:0030672	synaptic vesicle membrane	2.42E-03	2.47E-02
GO: Cellular Component	GO:0098889	intrinsic component of presynaptic membrane	2.69E-03	2.70E-02
GO: Cellular Component	GO:0042734	presynaptic membrane	3.39E-03	3.34E-02
GO: Cellular Component	GO:0098563	intrinsic component of synaptic vesicle membrane	3.89E-03	3.76E-02
GO: Cellular Component	GO:0048787	presynaptic active zone membrane	3.94E-03	3.76E-02
GO: Cellular Component	GO:0044306	neuron projection terminus	4.02E-03	3.77E-02
GO: Cellular Component	GO:0098685	Schaffer collateral - CA1 synapse	4.20E-03	3.88E-02
GO: Cellular Component	GO:0030285	integral component of synaptic vesicle membrane	4.32E-03	3.92E-02
GO: Cellular Component	GO:0032421	stereocilium bundle	4.83E-03	4.26E-02
GO: Cellular Component	GO:0032420	stereocilium	4.83E-03	4.26E-02
GO: Cellular Component	GO:0098686	hippocampal mossy fiber to CA3 synapse	5.15E-03	4.36E-02
GO: Cellular Component	GO:0097464	thorny excrescence	5.15E-03	4.36E-02
GO: Cellular Component	GO:0099056	integral component of presynaptic membrane	5.17E-03	4.36E-02
Pathway	1268763	Neuronal System	2.99E-08	5.24E-05
Pathway	777534	Insulin secretion	6.08E-06	5.23E-03
Pathway	M68	Regulation of RhoA activity	1.26E-05	5.23E-03
Pathway	138070	Regulation of RhoA activity	1.26E-05	5.23E-03
Pathway	1339117	Phase 0 - rapid depolarisation	1.49E-05	5.23E-03
Pathway	1269145	Neurotoxicity of clostridium toxins	6.31E-05	1.85E-02
Pathway	1268830	Voltage gated Potassium channels	8.10E-05	1.86E-02
Pathway	1269144	Uptake and actions of bacterial toxins	8.48E-05	1.86E-02
Pathway	1269868	Muscle contraction	1.33E-04	2.59E-02
Pathway	1339115	Cardiac conduction	1.72E-04	2.87E-02
Pathway	P05734	Synaptic vesicle trafficking	1.90E-04	2.87E-02
Pathway	908257	Adrenergic signaling in cardiomyocytes	1.96E-04	2.87E-02
Pathway	1427849	Protein-protein interactions at synapses	3.70E-04	4.68E-02
Pathway	M2890	Calcium signaling pathway	3.74E-04	4.68E-02
Disease	C4316903	Absence Seizures	1.28E-05	4.91E-02
Disease	C0023893	Liver Cirrhosis, Experimental	1.64E-05	4.91E-02

Abbreviations: rsFC, resting-state functional connectivity; A4hf, head and face region of area 4; GO, gene ontology.

Enrichment results of the genes related to rsFC of the A6cdl

Category	ID	Name	*P* value	*q* value (FDR-BH correction)
GO: Molecular Function	GO:0005244	voltage-gated ion channel activity	1.03E-05	1.59E-03
GO: Molecular Function	GO:0022832	voltage-gated channel activity	1.05E-05	1.59E-03
GO: Molecular Function	GO:0022843	voltage-gated cation channel activity	2.26E-05	2.27E-03
GO: Molecular Function	GO:0022839	ion gated channel activity	2.46E-04	1.56E-02
GO: Molecular Function	GO:0022836	gated channel activity	2.95E-04	1.56E-02
GO: Molecular Function	GO:0005245	voltage-gated calcium channel activity	3.10E-04	1.56E-02
GO: Molecular Function	GO:0005261	cation channel activity	4.79E-04	2.07E-02
GO: Molecular Function	GO:0005251	delayed rectifier potassium channel activity	6.62E-04	2.50E-02
GO: Molecular Function	GO:0008092	cytoskeletal protein binding	1.02E-03	3.19E-02
GO: Molecular Function	GO:0031432	titin binding	1.06E-03	3.19E-02
GO: Molecular Function	GO:0015643	toxic substance binding	1.22E-03	3.34E-02
GO: Molecular Function	GO:0046873	metal ion transmembrane transporter activity	1.50E-03	3.78E-02
GO: Molecular Function	GO:0005216	ion channel activity	1.67E-03	3.89E-02
GO: Molecular Function	GO:0016247	channel regulator activity	2.04E-03	4.41E-02
GO: Molecular Function	GO:0015267	channel activity	3.02E-03	4.59E-02
GO: Molecular Function	GO:0022803	passive transmembrane transporter activity	3.05E-03	4.59E-02
GO: Molecular Function	GO:0019120	hydrolase activity, acting on acid halide bonds, in C-halide compounds	3.49E-03	4.59E-02
GO: Molecular Function	GO:0070287	ferritin receptor activity	3.49E-03	4.59E-02
GO: Molecular Function	GO:0099567	calcium ion binding involved in regulation of postsynaptic cytosolic calcium ion concentration	3.49E-03	4.59E-02
GO: Molecular Function	GO:0070524	11-beta-hydroxysteroid dehydrogenase (NADP+) activity	3.49E-03	4.59E-02
GO: Molecular Function	GO:0004603	phenylethanolamine N-methyltransferase activity	3.49E-03	4.59E-02
GO: Molecular Function	GO:0016824	hydrolase activity, acting on acid halide bonds	3.49E-03	4.59E-02
GO: Molecular Function	GO:0047651	alkylhalidase activity	3.49E-03	4.59E-02
GO: Molecular Function	GO:0050431	transforming growth factor beta binding	3.96E-03	4.98E-02
GO: Biological Process	GO:0050976	detection of mechanical stimulus involved in sensory perception of touch	3.36E-05	2.02E-02
GO: Biological Process	GO:0033693	neurofilament bundle assembly	3.36E-05	2.02E-02
GO: Biological Process	GO:1904062	regulation of cation transmembrane transport	5.29E-05	2.02E-02
GO: Biological Process	GO:0032412	regulation of ion transmembrane transporter activity	6.20E-05	2.02E-02
GO: Biological Process	GO:2001257	regulation of cation channel activity	6.33E-05	2.02E-02
GO: Biological Process	GO:0022898	regulation of transmembrane transporter activity	7.33E-05	2.02E-02
GO: Biological Process	GO:0032409	regulation of transporter activity	1.01E-04	2.38E-02
GO: Biological Process	GO:0010959	regulation of metal ion transport	1.67E-04	3.06E-02
GO: Biological Process	GO:0050975	sensory perception of touch	1.67E-04	3.06E-02
GO: Biological Process	GO:0045110	intermediate filament bundle assembly	2.33E-04	3.60E-02
GO: Biological Process	GO:0006812	cation transport	2.40E-04	3.60E-02
GO: Biological Process	GO:0043269	regulation of ion transport	3.74E-04	4.82E-02
GO: Biological Process	GO:0030029	actin filament-based process	4.44E-04	4.82E-02
GO: Biological Process	GO:0034765	regulation of ion transmembrane transport	4.50E-04	4.82E-02
GO: Biological Process	GO:0098662	inorganic cation transmembrane transport	4.58E-04	4.82E-02
GO: Biological Process	GO:0051480	regulation of cytosolic calcium ion concentration	4.78E-04	4.82E-02
GO: Biological Process	GO:0060052	neurofilament cytoskeleton organization	4.96E-04	4.82E-02
GO: Cellular Component	GO:0005883	neurofilament	1.28E-05	1.98E-03
GO: Cellular Component	GO:0030424	axon	1.55E-05	1.98E-03
GO: Cellular Component	GO:0099160	postsynaptic intermediate filament cytoskeleton	6.62E-05	5.62E-03
GO: Cellular Component	GO:0097418	neurofibrillary tangle	1.10E-04	6.31E-03
GO: Cellular Component	GO:0044305	calyx of Held	1.24E-04	6.31E-03
GO: Cellular Component	GO:0043005	neuron projection	2.59E-04	1.10E-02
GO: Cellular Component	GO:0034703	cation channel complex	1.09E-03	3.66E-02
GO: Cellular Component	GO:0045202	synapse	1.15E-03	3.66E-02

Abbreviations: rsFC, resting-state functional connectivity; A6cdl, caudal dorsolateral area 6; GO, gene ontology.

Enrichment results of the genes related to rsFC of the A4ul

Category	ID	Name	*P* value	*q* value (FDR-BH correction)
GO: Molecular Function	GO:0022857	transmembrane transporter activity	1.33E-11	1.23E-08
GO: Molecular Function	GO:0005215	transporter activity	1.69E-11	1.23E-08
GO: Molecular Function	GO:0015075	ion transmembrane transporter activity	3.53E-10	1.70E-07
GO: Molecular Function	GO:0015318	inorganic molecular entity transmembrane transporter activity	5.19E-10	1.88E-07
GO: Molecular Function	GO:0046873	metal ion transmembrane transporter activity	1.65E-09	4.78E-07
GO: Molecular Function	GO:0015267	channel activity	7.70E-09	1.47E-06
GO: Molecular Function	GO:0005216	ion channel activity	8.05E-09	1.47E-06
GO: Molecular Function	GO:0022803	passive transmembrane transporter activity	8.13E-09	1.47E-06
GO: Molecular Function	GO:0005261	cation channel activity	1.81E-08	2.91E-06
GO: Molecular Function	GO:0008324	cation transmembrane transporter activity	2.63E-08	3.81E-06
GO: Molecular Function	GO:0022839	ion gated channel activity	1.02E-07	1.34E-05
GO: Molecular Function	GO:0022890	inorganic cation transmembrane transporter activity	1.30E-07	1.57E-05
GO: Molecular Function	GO:0022836	gated channel activity	2.23E-07	2.49E-05
GO: Molecular Function	GO:0016247	channel regulator activity	3.95E-07	4.08E-05
GO: Molecular Function	GO:0005244	voltage-gated ion channel activity	3.81E-06	3.68E-04
GO: Molecular Function	GO:0022832	voltage-gated channel activity	4.07E-06	3.69E-04
GO: Molecular Function	GO:0042165	neurotransmitter binding	1.65E-05	1.40E-03
GO: Molecular Function	GO:0030594	neurotransmitter receptor activity	2.38E-05	1.91E-03
GO: Molecular Function	GO:0005251	delayed rectifier potassium channel activity	2.65E-05	2.02E-03
GO: Molecular Function	GO:0004683	calmodulin-dependent protein kinase activity	2.94E-05	2.09E-03
GO: Molecular Function	GO:0005272	sodium channel activity	3.03E-05	2.09E-03
GO: Molecular Function	GO:0022843	voltage-gated cation channel activity	3.20E-05	2.10E-03
GO: Molecular Function	GO:0008092	cytoskeletal protein binding	4.37E-05	2.75E-03
GO: Molecular Function	GO:0099106	ion channel regulator activity	6.51E-05	3.90E-03
GO: Molecular Function	GO:0015085	calcium ion transmembrane transporter activity	6.74E-05	3.90E-03
GO: Molecular Function	GO:0015081	sodium ion transmembrane transporter activity	8.37E-05	4.66E-03
GO: Molecular Function	GO:0098960	postsynaptic neurotransmitter receptor activity	9.73E-05	5.22E-03
GO: Molecular Function	GO:0017080	sodium channel regulator activity	1.11E-04	5.72E-03
GO: Molecular Function	GO:0015077	monovalent inorganic cation transmembrane transporter activity	1.54E-04	7.59E-03
GO: Molecular Function	GO:0099095	ligand-gated anion channel activity	1.57E-04	7.59E-03
GO: Molecular Function	GO:0031681	G-protein beta-subunit binding	2.09E-04	9.75E-03
GO: Molecular Function	GO:0005509	calcium ion binding	2.18E-04	9.85E-03
GO: Molecular Function	GO:0008509	anion transmembrane transporter activity	4.47E-04	1.96E-02
GO: Molecular Function	GO:0005001	transmembrane receptor protein tyrosine phosphatase activity	4.91E-04	1.96E-02
GO: Molecular Function	GO:0019198	transmembrane receptor protein phosphatase activity	4.91E-04	1.96E-02
GO: Molecular Function	GO:0050811	GABA receptor binding	4.91E-04	1.96E-02
GO: Molecular Function	GO:0005267	potassium channel activity	5.02E-04	1.96E-02
GO: Molecular Function	GO:0015079	potassium ion transmembrane transporter activity	6.42E-04	2.45E-02
GO: Molecular Function	GO:0086006	voltage-gated sodium channel activity involved in cardiac muscle cell action potential	6.64E-04	2.47E-02
GO: Molecular Function	GO:0005249	voltage-gated potassium channel activity	8.75E-04	3.17E-02
GO: Molecular Function	GO:0043177	organic acid binding	1.07E-03	3.78E-02
GO: Molecular Function	GO:0022835	transmitter-gated channel activity	1.21E-03	4.07E-02
GO: Molecular Function	GO:0022824	transmitter-gated ion channel activity	1.21E-03	4.07E-02
GO: Molecular Function	GO:0005230	extracellular ligand-gated ion channel activity	1.24E-03	4.07E-02
GO: Molecular Function	GO:0015186	L-glutamine transmembrane transporter activity	1.29E-03	4.07E-02
GO: Molecular Function	GO:0031406	carboxylic acid binding	1.29E-03	4.07E-02
GO: Molecular Function	GO:0016597	amino acid binding	1.34E-03	4.12E-02
GO: Molecular Function	GO:0098631	cell adhesion mediator activity	1.37E-03	4.13E-02
GO: Molecular Function	GO:0005516	calmodulin binding	1.42E-03	4.21E-02
GO: Molecular Function	GO:0022851	GABA-gated chloride ion channel activity	1.55E-03	4.48E-02
GO: Molecular Function	GO:0005496	steroid binding	1.71E-03	4.85E-02
GO: Molecular Function	GO:0022852	glycine-gated chloride ion channel activity	1.79E-03	4.98E-02
GO: Biological Process	GO:0099536	synaptic signaling	2.19E-14	6.93E-11
GO: Biological Process	GO:0099537	trans-synaptic signaling	2.68E-14	6.93E-11
GO: Biological Process	GO:0098916	anterograde trans-synaptic signaling	4.21E-14	6.93E-11
GO: Biological Process	GO:0007268	chemical synaptic transmission	4.21E-14	6.93E-11
GO: Biological Process	GO:0006811	ion transport	1.02E-12	1.34E-09
GO: Biological Process	GO:0007267	cell-cell signaling	4.52E-12	4.96E-09
GO: Biological Process	GO:0055085	transmembrane transport	2.58E-11	2.43E-08
GO: Biological Process	GO:0034220	ion transmembrane transport	5.24E-11	4.31E-08
GO: Biological Process	GO:0030001	metal ion transport	3.58E-10	2.55E-07
GO: Biological Process	GO:0050804	modulation of chemical synaptic transmission	3.98E-10	2.55E-07
GO: Biological Process	GO:0099177	regulation of trans-synaptic signaling	4.26E-10	2.55E-07
GO: Biological Process	GO:0023061	signal release	6.48E-10	3.56E-07
GO: Biological Process	GO:0006812	cation transport	7.40E-10	3.75E-07
GO: Biological Process	GO:0043269	regulation of ion transport	1.08E-08	5.07E-06
GO: Biological Process	GO:0098660	inorganic ion transmembrane transport	1.46E-08	6.39E-06
GO: Biological Process	GO:0098655	cation transmembrane transport	2.45E-08	1.01E-05
GO: Biological Process	GO:0009914	hormone transport	2.40E-07	9.24E-05
GO: Biological Process	GO:0098662	inorganic cation transmembrane transport	2.53E-07	9.24E-05
GO: Biological Process	GO:0044057	regulation of system process	7.48E-07	2.59E-04
GO: Biological Process	GO:0010817	regulation of hormone levels	1.12E-06	3.66E-04
GO: Biological Process	GO:0046879	hormone secretion	1.17E-06	3.66E-04
GO: Biological Process	GO:0042391	regulation of membrane potential	1.71E-06	5.11E-04
GO: Biological Process	GO:0034762	regulation of transmembrane transport	1.90E-06	5.43E-04
GO: Biological Process	GO:0035637	multicellular organismal signaling	3.18E-06	8.71E-04
GO: Biological Process	GO:0140352	export from cell	3.77E-06	9.64E-04
GO: Biological Process	GO:0048699	generation of neurons	3.80E-06	9.64E-04
GO: Biological Process	GO:0015672	monovalent inorganic cation transport	4.30E-06	1.05E-03
GO: Biological Process	GO:0034765	regulation of ion transmembrane transport	5.04E-06	1.15E-03
GO: Biological Process	GO:0070838	divalent metal ion transport	5.04E-06	1.15E-03
GO: Biological Process	GO:0002028	regulation of sodium ion transport	6.09E-06	1.34E-03
GO: Biological Process	GO:0072511	divalent inorganic cation transport	7.00E-06	1.49E-03
GO: Biological Process	GO:0022008	neurogenesis	8.34E-06	1.72E-03
GO: Biological Process	GO:1902305	regulation of sodium ion transmembrane transport	9.44E-06	1.88E-03
GO: Biological Process	GO:0071248	cellular response to metal ion	1.15E-05	2.23E-03
GO: Biological Process	GO:0006814	sodium ion transport	1.26E-05	2.38E-03
GO: Biological Process	GO:0099565	chemical synaptic transmission, postsynaptic	1.31E-05	2.40E-03
GO: Biological Process	GO:0046883	regulation of hormone secretion	1.38E-05	2.45E-03
GO: Biological Process	GO:0006816	calcium ion transport	1.48E-05	2.51E-03
GO: Biological Process	GO:0032940	secretion by cell	1.53E-05	2.51E-03
GO: Biological Process	GO:0099643	signal release from synapse	1.56E-05	2.51E-03
GO: Biological Process	GO:0007269	neurotransmitter secretion	1.56E-05	2.51E-03
GO: Biological Process	GO:0015711	organic anion transport	1.63E-05	2.56E-03
GO: Biological Process	GO:0048666	neuron development	1.74E-05	2.66E-03
GO: Biological Process	GO:1904062	regulation of cation transmembrane transport	1.92E-05	2.87E-03
GO: Biological Process	GO:0050808	synapse organization	1.96E-05	2.87E-03
GO: Biological Process	GO:0032412	regulation of ion transmembrane transporter activity	2.00E-05	2.87E-03
GO: Biological Process	GO:0070588	calcium ion transmembrane transport	2.08E-05	2.89E-03
GO: Biological Process	GO:0035725	sodium ion transmembrane transport	2.11E-05	2.89E-03
GO: Biological Process	GO:0010959	regulation of metal ion transport	2.16E-05	2.90E-03
GO: Biological Process	GO:0007610	behavior	2.41E-05	3.17E-03
GO: Biological Process	GO:0006836	neurotransmitter transport	2.62E-05	3.38E-03
GO: Biological Process	GO:0006820	anion transport	3.06E-05	3.87E-03
GO: Biological Process	GO:0032409	regulation of transporter activity	3.18E-05	3.96E-03
GO: Biological Process	GO:0022898	regulation of transmembrane transporter activity	3.25E-05	3.96E-03
GO: Biological Process	GO:0098656	anion transmembrane transport	3.50E-05	4.19E-03
GO: Biological Process	GO:0010038	response to metal ion	3.72E-05	4.33E-03
GO: Biological Process	GO:0046942	carboxylic acid transport	3.75E-05	4.33E-03
GO: Biological Process	GO:0015849	organic acid transport	3.95E-05	4.41E-03
GO: Biological Process	GO:0006813	potassium ion transport	3.95E-05	4.41E-03
GO: Biological Process	GO:0071241	cellular response to inorganic substance	4.12E-05	4.53E-03
GO: Biological Process	GO:0030182	neuron differentiation	4.20E-05	4.54E-03
GO: Biological Process	GO:2000649	regulation of sodium ion transmembrane transporter activity	5.21E-05	5.54E-03
GO: Biological Process	GO:0046928	regulation of neurotransmitter secretion	6.26E-05	6.54E-03
GO: Biological Process	GO:0014888	striated muscle adaptation	6.84E-05	7.04E-03
GO: Biological Process	GO:0031175	neuron projection development	7.87E-05	7.98E-03
GO: Biological Process	GO:0051592	response to calcium ion	8.47E-05	8.46E-03

GO: Biological Process	GO:0030030	cell projection organization	9.73E-05	9.57E-03
GO: Biological Process	GO:0060078	regulation of postsynaptic membrane potential	1.07E-04	1.03E-02
GO: Biological Process	GO:0120036	plasma membrane bounded cell projection organization	1.08E-04	1.03E-02
GO: Biological Process	GO:0046903	secretion	1.13E-04	1.06E-02
GO: Biological Process	GO:0071805	potassium ion transmembrane transport	1.20E-04	1.12E-02
GO: Biological Process	GO:0043270	positive regulation of ion transport	1.22E-04	1.12E-02
GO: Biological Process	GO:0051668	localization within membrane	1.25E-04	1.12E-02
GO: Biological Process	GO:0051960	regulation of nervous system development	1.28E-04	1.14E-02
GO: Biological Process	GO:1903035	negative regulation of response to wounding	1.43E-04	1.25E-02
GO: Biological Process	GO:0003013	circulatory system process	1.66E-04	1.44E-02
GO: Biological Process	GO:1903530	regulation of secretion by cell	1.77E-04	1.52E-02
GO: Biological Process	GO:0051046	regulation of secretion	2.10E-04	1.75E-02
GO: Biological Process	GO:0050807	regulation of synapse organization	2.11E-04	1.75E-02
GO: Biological Process	GO:0140115	export across plasma membrane	2.12E-04	1.75E-02
GO: Biological Process	GO:0001508	action potential	2.19E-04	1.75E-02
GO: Biological Process	GO:0010975	regulation of neuron projection development	2.20E-04	1.75E-02
GO: Biological Process	GO:0071277	cellular response to calcium ion	2.20E-04	1.75E-02
GO: Biological Process	GO:0008015	blood circulation	2.35E-04	1.85E-02
GO: Biological Process	GO:0061337	cardiac conduction	2.44E-04	1.89E-02
GO: Biological Process	GO:0010765	positive regulation of sodium ion transport	2.48E-04	1.89E-02
GO: Biological Process	GO:0044272	sulfur compound biosynthetic process	2.50E-04	1.89E-02
GO: Biological Process	GO:0050767	regulation of neurogenesis	2.55E-04	1.90E-02
GO: Biological Process	GO:0051588	regulation of neurotransmitter transport	2.85E-04	2.11E-02
GO: Biological Process	GO:0098962	regulation of postsynaptic neurotransmitter receptor activity	2.92E-04	2.14E-02
GO: Biological Process	GO:0071407	cellular response to organic cyclic compound	2.96E-04	2.14E-02
GO: Biological Process	GO:1901655	cellular response to ketone	3.43E-04	2.46E-02
GO: Biological Process	GO:0050803	regulation of synapse structure or activity	3.80E-04	2.69E-02
GO: Biological Process	GO:0007155	cell adhesion	3.92E-04	2.69E-02
GO: Biological Process	GO:1903305	regulation of regulated secretory pathway	3.93E-04	2.69E-02
GO: Biological Process	GO:0090066	regulation of anatomical structure size	3.96E-04	2.69E-02
GO: Biological Process	GO:0010976	positive regulation of neuron projection development	3.97E-04	2.69E-02
GO: Biological Process	GO:0019228	neuronal action potential	4.01E-04	2.70E-02
GO: Biological Process	GO:0001505	regulation of neurotransmitter levels	4.13E-04	2.75E-02
GO: Biological Process	GO:0032594	protein transport within lipid bilayer	4.24E-04	2.79E-02
GO: Biological Process	GO:0032509	endosome transport via multivesicular body sorting pathway	4.42E-04	2.88E-02
GO: Biological Process	GO:0022610	biological adhesion	4.54E-04	2.93E-02
GO: Biological Process	GO:2001257	regulation of cation channel activity	4.73E-04	3.02E-02
GO: Biological Process	GO:0007626	locomotory behavior	4.90E-04	3.08E-02
GO: Biological Process	GO:0050890	cognition	4.90E-04	3.08E-02
GO: Biological Process	GO:0071242	cellular response to ammonium ion	5.19E-04	3.23E-02
GO: Biological Process	GO:1903034	regulation of response to wounding	5.34E-04	3.29E-02
GO: Biological Process	GO:1990778	protein localization to cell periphery	5.59E-04	3.41E-02
GO: Biological Process	GO:0050877	nervous system process	6.03E-04	3.64E-02
GO: Biological Process	GO:0048812	neuron projection morphogenesis	6.10E-04	3.64E-02
GO: Biological Process	GO:0032990	cell part morphogenesis	6.14E-04	3.64E-02
GO: Biological Process	GO:0071985	multivesicular body sorting pathway	6.24E-04	3.66E-02
GO: Biological Process	GO:1903825	organic acid transmembrane transport	6.38E-04	3.66E-02
GO: Biological Process	GO:1905039	carboxylic acid transmembrane transport	6.38E-04	3.66E-02
GO: Biological Process	GO:0060768	regulation of epithelial cell proliferation involved in prostate gland development	6.45E-04	3.66E-02
GO: Biological Process	GO:0007156	homophilic cell adhesion via plasma membrane adhesion molecules	6.45E-04	3.66E-02
GO: Biological Process	GO:0042493	response to drug	6.49E-04	3.66E-02
GO: Biological Process	GO:0031644	regulation of nervous system process	7.33E-04	4.09E-02
GO: Biological Process	GO:0045664	regulation of neuron differentiation	7.90E-04	4.37E-02
GO: Biological Process	GO:0050771	negative regulation of axonogenesis	8.05E-04	4.42E-02
GO: Biological Process	GO:0099601	regulation of neurotransmitter receptor activity	8.44E-04	4.60E-02
GO: Biological Process	GO:0086010	membrane depolarization during action potential	8.86E-04	4.76E-02
GO: Biological Process	GO:0007611	learning or memory	8.88E-04	4.76E-02
GO: Biological Process	GO:0097623	potassium ion export across plasma membrane	9.08E-04	4.82E-02
GO: Biological Process	GO:0120039	plasma membrane bounded cell projection morphogenesis	9.35E-04	4.83E-02
GO: Biological Process	GO:0010035	response to inorganic substance	9.35E-04	4.83E-02
GO: Biological Process	GO:0060767	epithelial cell proliferation involved in prostate gland development	9.37E-04	4.83E-02
GO: Biological Process	GO:0006869	lipid transport	9.40E-04	4.83E-02
GO: Biological Process	GO:0035690	cellular response to drug	9.45E-04	4.83E-02
GO: Cellular Component	GO:0043005	neuron projection	5.06E-23	3.74E-20
GO: Cellular Component	GO:0045202	synapse	6.41E-18	2.37E-15
GO: Cellular Component	GO:0030424	axon	1.17E-17	2.89E-15
GO: Cellular Component	GO:0036477	somatodendritic compartment	1.32E-15	2.45E-13
GO: Cellular Component	GO:0097060	synaptic membrane	1.43E-14	2.11E-12
GO: Cellular Component	GO:0030425	dendrite	5.53E-14	5.84E-12
GO: Cellular Component	GO:0097447	dendritic tree	5.53E-14	5.84E-12
GO: Cellular Component	GO:0098978	glutamatergic synapse	9.60E-13	8.88E-11
GO: Cellular Component	GO:0098794	postsynapse	7.72E-12	6.34E-10
GO: Cellular Component	GO:0005887	integral component of plasma membrane	3.54E-11	2.62E-09
GO: Cellular Component	GO:0099699	integral component of synaptic membrane	6.35E-11	4.27E-09
GO: Cellular Component	GO:0031226	intrinsic component of plasma membrane	7.90E-11	4.87E-09
GO: Cellular Component	GO:0099240	intrinsic component of synaptic membrane	1.19E-10	6.79E-09
GO: Cellular Component	GO:0043025	neuronal cell body	2.13E-10	1.13E-08
GO: Cellular Component	GO:1902495	transmembrane transporter complex	4.04E-10	1.99E-08
GO: Cellular Component	GO:0098793	presynapse	5.68E-10	2.53E-08
GO: Cellular Component	GO:0034702	ion channel complex	5.82E-10	2.53E-08
GO: Cellular Component	GO:0044297	cell body	6.40E-10	2.63E-08
GO: Cellular Component	GO:1990351	transporter complex	8.14E-10	3.17E-08
GO: Cellular Component	GO:0045211	postsynaptic membrane	9.25E-10	3.42E-08
GO: Cellular Component	GO:0034703	cation channel complex	8.79E-09	3.10E-07
GO: Cellular Component	GO:0098936	intrinsic component of postsynaptic membrane	3.26E-08	1.10E-06
GO: Cellular Component	GO:0099055	integral component of postsynaptic membrane	4.97E-08	1.60E-06
GO: Cellular Component	GO:0044309	neuron spine	1.57E-07	4.84E-06
GO: Cellular Component	GO:0150034	distal axon	4.25E-07	1.26E-05
GO: Cellular Component	GO:0099572	postsynaptic specialization	8.74E-07	2.49E-05
GO: Cellular Component	GO:0042734	presynaptic membrane	1.05E-06	2.88E-05
GO: Cellular Component	GO:0043197	dendritic spine	1.15E-06	3.05E-05
GO: Cellular Component	GO:0098984	neuron to neuron synapse	1.35E-06	3.43E-05
GO: Cellular Component	GO:0030054	cell junction	1.46E-06	3.59E-05
GO: Cellular Component	GO:0098797	plasma membrane protein complex	2.46E-06	5.86E-05
GO: Cellular Component	GO:0098889	intrinsic component of presynaptic membrane	3.06E-06	7.08E-05
GO: Cellular Component	GO:0099056	integral component of presynaptic membrane	3.30E-06	7.41E-05
GO: Cellular Component	GO:0098982	GABA-ergic synapse	3.44E-06	7.48E-05
GO: Cellular Component	GO:0034705	potassium channel complex	6.58E-06	1.39E-04
GO: Cellular Component	GO:0008076	voltage-gated potassium channel complex	8.52E-06	1.75E-04
GO: Cellular Component	GO:0009898	cytoplasmic side of plasma membrane	9.30E-06	1.86E-04
GO: Cellular Component	GO:0043204	perikaryon	9.97E-06	1.94E-04
GO: Cellular Component	GO:0044305	calyx of Held	1.07E-05	2.03E-04
GO: Cellular Component	GO:0014069	postsynaptic density	1.26E-05	2.27E-04
GO: Cellular Component	GO:0032279	asymmetric synapse	1.26E-05	2.27E-04
GO: Cellular Component	GO:0043679	axon terminus	2.76E-05	4.86E-04
GO: Cellular Component	GO:0014704	intercalated disc	4.50E-05	7.74E-04
GO: Cellular Component	GO:0098562	cytoplasmic side of membrane	7.53E-05	1.27E-03
GO: Cellular Component	GO:0032589	neuron projection membrane	8.98E-05	1.48E-03
GO: Cellular Component	GO:0044306	neuron projection terminus	1.09E-04	1.76E-03
GO: Cellular Component	GO:0001518	voltage-gated sodium channel complex	1.33E-04	2.10E-03
GO: Cellular Component	GO:0044291	cell-cell contact zone	1.38E-04	2.13E-03
GO: Cellular Component	GO:0097457	hippocampal mossy fiber	2.20E-04	3.32E-03
GO: Cellular Component	GO:0099060	integral component of postsynaptic specialization membrane	2.24E-04	3.32E-03
GO: Cellular Component	GO:0044302	dentate gyrus mossy fiber	2.54E-04	3.62E-03
GO: Cellular Component	GO:0034706	sodium channel complex	2.55E-04	3.62E-03
GO: Cellular Component	GO:0098948	intrinsic component of postsynaptic specialization membrane	3.89E-04	5.43E-03
GO: Cellular Component	GO:0019897	extrinsic component of plasma membrane	4.50E-04	6.16E-03
GO: Cellular Component	GO:0032590	dendrite membrane	4.90E-04	6.59E-03
GO: Cellular Component	GO:0042383	sarcolemma	5.11E-04	6.75E-03
GO: Cellular Component	GO:0098686	hippocampal mossy fiber to CA3 synapse	6.50E-04	8.30E-03
GO: Cellular Component	GO:0097464	thorny excrescence	6.50E-04	8.30E-03
GO: Cellular Component	GO:0098796	membrane protein complex	7.26E-04	9.11E-03

GO: Cellular Component	GO:1990026	hippocampal mossy fiber expansion	8.51E-04	1.05E-02
GO: Cellular Component	GO:0099634	postsynaptic specialization membrane	8.79E-04	1.07E-02
GO: Cellular Component	GO:0015629	actin cytoskeleton	1.06E-03	1.27E-02
GO: Cellular Component	GO:0031680	G-protein beta/gamma-subunit complex	1.24E-03	1.46E-02
GO: Cellular Component	GO:0030426	growth cone	1.44E-03	1.67E-02
GO: Cellular Component	GO:0019898	extrinsic component of membrane	1.50E-03	1.71E-02
GO: Cellular Component	GO:0005911	cell-cell junction	1.55E-03	1.72E-02
GO: Cellular Component	GO:0034681	integrin alpha11-beta1 complex	1.58E-03	1.72E-02
GO: Cellular Component	GO:0034677	integrin alpha7-beta1 complex	1.58E-03	1.72E-02
GO: Cellular Component	GO:0031234	extrinsic component of cytoplasmic side of plasma membrane	1.65E-03	1.77E-02
GO: Cellular Component	GO:0030427	site of polarized growth	1.85E-03	1.96E-02
GO: Cellular Component	GO:0043195	terminal bouton	1.98E-03	2.06E-02
GO: Cellular Component	GO:0032591	dendritic spine membrane	2.22E-03	2.29E-02
GO: Cellular Component	GO:0031252	cell leading edge	3.10E-03	3.10E-02
GO: Cellular Component	GO:0034707	chloride channel complex	3.10E-03	3.10E-02
GO: Cellular Component	GO:0031256	leading edge membrane	3.42E-03	3.37E-02
GO: Cellular Component	GO:0060076	excitatory synapse	3.59E-03	3.49E-02
GO: Cellular Component	GO:0045121	membrane raft	3.71E-03	3.56E-02
GO: Cellular Component	GO:0098857	membrane microdomain	3.84E-03	3.64E-02
GO: Cellular Component	GO:0005884	actin filament	4.14E-03	3.83E-02
GO: Cellular Component	GO:0043235	receptor complex	4.14E-03	3.83E-02
GO: Cellular Component	GO:0097478	leaflet of membrane bilayer	4.40E-03	3.97E-02
GO: Cellular Component	GO:0098552	side of membrane	4.40E-03	3.97E-02
GO: Cellular Component	GO:0031594	neuromuscular junction	4.90E-03	4.37E-02
GO: Cellular Component	GO:0098685	Schaffer collateral - CA1 synapse	4.98E-03	4.39E-02
GO: Cellular Component	GO:0030175	filopodium	5.32E-03	4.59E-02
GO: Cellular Component	GO:0008328	ionotropic glutamate receptor complex	5.33E-03	4.59E-02
GO: Cellular Component	GO:0009986	cell surface	5.55E-03	4.69E-02
GO: Cellular Component	GO:1902711	GABA-A receptor complex	5.58E-03	4.69E-02
GO: Cellular Component	GO:0032281	AMPA glutamate receptor complex	5.65E-03	4.70E-02
GO: Cellular Component	GO:0098589	membrane region	6.06E-03	4.98E-02
Pathway	1268763	Neuronal System	1.67E-16	3.91E-13
Pathway	1268766	Transmission across Chemical Synapses	1.79E-09	2.09E-06
Pathway	M2890	Calcium signaling pathway	7.81E-08	6.10E-05
Pathway	83050	Calcium signaling pathway	1.36E-07	7.95E-05
Pathway	1268786	Neurotransmitter Receptor Binding And Downstream Transmission In The Postsynaptic Cell	7.10E-07	2.98E-04
Pathway	P04385	Histamine H1 receptor mediated signaling pathway	7.63E-07	2.98E-04
Pathway	1268830	Voltage gated Potassium channels	1.27E-06	4.24E-04
Pathway	P04374	5HT2 type receptor mediated signaling pathway	1.48E-06	4.32E-04
Pathway	P04391	Oxytocin receptor mediated signaling pathway	2.01E-06	5.22E-04
Pathway	1339117	Phase 0 - rapid depolarisation	2.57E-06	6.02E-04
Pathway	1268821	Potassium Channels	3.34E-06	6.42E-04
Pathway	P04394	Thyrotropin-releasing hormone receptor signaling pathway	3.64E-06	6.42E-04
Pathway	537443	Retrograde endocannabinoid signaling	3.82E-06	6.42E-04
Pathway	1268795	Trafficking of AMPA receptors	4.12E-06	6.42E-04
Pathway	1268794	Glutamate Binding, Activation of AMPA Receptors and Synaptic Plasticity	4.12E-06	6.42E-04
Pathway	213818	Glutamatergic synapse	4.48E-06	6.56E-04
Pathway	908257	Adrenergic signaling in cardiomyocytes	7.91E-06	1.09E-03
Pathway	777534	Insulin secretion	9.67E-06	1.26E-03
Pathway	M68	Regulation of RhoA activity	1.49E-05	1.74E-03
Pathway	138070	Regulation of RhoA activity	1.49E-05	1.74E-03
Pathway	M7761	Melanogenesis	1.71E-05	1.82E-03
Pathway	83092	Melanogenesis	1.71E-05	1.82E-03
Pathway	213307	Endocrine and other factor-regulated calcium reabsorption	1.82E-05	1.86E-03
Pathway	552665	Morphine addiction	2.16E-05	2.11E-03
Pathway	1270102	Regulation of insulin secretion	2.33E-05	2.14E-03
Pathway	1339115	Cardiac conduction	2.38E-05	2.14E-03
Pathway	698773	Circadian entrainment	4.01E-05	3.47E-03
Pathway	948277	Inflammatory mediator regulation of TRP channels	4.51E-05	3.65E-03
Pathway	1269584	Opioid Signalling	4.52E-05	3.65E-03
Pathway	154409	Gastric acid secretion	5.60E-05	4.37E-03
Pathway	377263	GABAergic synapse	6.55E-05	4.95E-03
Pathway	153376	Salivary secretion	8.31E-05	6.08E-03
Pathway	83085	Long-term potentiation	8.94E-05	6.34E-03
Pathway	1269868	Muscle contraction	1.18E-04	8.11E-03
Pathway	M3115	Long-term potentiation	1.34E-04	8.87E-03
Pathway	1272485	Aldosterone synthesis and secretion	1.36E-04	8.87E-03
Pathway	P00057	Wnt signaling pathway	1.78E-04	1.13E-02
Pathway	1427849	Protein-protein interactions at synapses	1.98E-04	1.19E-02
Pathway	1270101	Integration of energy metabolism	1.99E-04	1.19E-02
Pathway	1269903	Transmembrane transport of small molecules	2.16E-04	1.23E-02
Pathway	1017634	cAMP signaling pathway	2.20E-04	1.23E-02
Pathway	137953	Role of Calcineurin-dependent NFAT signaling in lymphocytes	2.21E-04	1.23E-02
Pathway	M15181	Regulation of PGC-1a	2.58E-04	1.41E-02
Pathway	M9052	Phosphatidylinositol signaling system	2.84E-04	1.51E-02
Pathway	M113	Role of Calcineurin-dependent NFAT signaling in lymphocytes	2.99E-04	1.56E-02
Pathway	946598	Thyroid hormone signaling pathway	3.17E-04	1.61E-02
Pathway	1269341	Platelet homeostasis	3.70E-04	1.85E-02
Pathway	P00042	Muscarinic acetylcholine receptor 1 and 3 signaling pathway	3.98E-04	1.94E-02
Pathway	PW:0000232	phosphatidylinositol 3-kinase-Akt signaling	4.16E-04	1.99E-02
Pathway	1339121	Ion homeostasis	4.57E-04	2.14E-02
Pathway	1268796	Trafficking of GluR2-containing AMPA receptors	4.97E-04	2.28E-02
Pathway	1269145	Neurotoxicity of clostridium toxins	5.07E-04	2.28E-02
Pathway	M19118	Keratinocyte Differentiation	5.46E-04	2.41E-02
Pathway	1269951	Ion transport by P-type ATPases	5.97E-04	2.59E-02
Pathway	952859	Oxytocin signaling pathway	6.25E-04	2.65E-02
Pathway	1269587	PLC beta mediated events	6.34E-04	2.65E-02
Pathway	1427850	Interactions of neurexins and neuroligins at synapses	6.79E-04	2.77E-02
Pathway	P00027	Heterotrimeric G-protein signaling pathway-Gq alpha and Go alpha mediated pathway	6.87E-04	2.77E-02
Pathway	1269586	G-protein mediated events	7.34E-04	2.86E-02
Pathway	1269577	G alpha (z) signalling events	7.34E-04	2.86E-02
Pathway	525336	Serotonergic synapse	8.14E-04	3.13E-02
Pathway	1269989	Synthesis of IP3 and IP4 in the cytosol	8.63E-04	3.24E-02
Pathway	142435	glutathione-mediated detoxification	8.71E-04	3.24E-02
Pathway	1269950	Ion channel transport	1.15E-03	4.19E-02
Pathway	1270213	Glutathione conjugation	1.16E-03	4.19E-02
Pathway	1269588	Ca-dependent events	1.27E-03	4.43E-02
Pathway	1270325	Interaction between L1 and Ankyrins	1.27E-03	4.43E-02
Pathway	114228	Fc gamma R-mediated phagocytosis	1.34E-03	4.62E-02
Pathway	P00002	Alpha adrenergic receptor signaling pathway	1.42E-03	4.83E-02
Pathway	83091	GnRH signaling pathway	1.47E-03	4.87E-02
Pathway	1269916	Amino acid transport across the plasma membrane	1.52E-03	4.87E-02
Pathway	1269144	Uptake and actions of bacterial toxins	1.52E-03	4.87E-02
Pathway	1269345	Platelet calcium homeostasis	1.52E-03	4.87E-02
Disease	C0023893	Liver Cirrhosis, Experimental	2.82E-07	2.25E-03
Disease	C4316903	Absence Seizures	2.52E-06	1.01E-02
Disease	C0004352	Autistic Disorder	4.65E-06	1.24E-02

Abbreviations: rsFC, resting-state functional connectivity; A4ul, upper limb region of area 4; GO, gene ontology.

Enrichment results of the genes related to rsFC of the A4ll				
**Category**	**ID**	**Name**	***P* value**	***q* value (FDR-BH correction)**
GO: Cellular Component	GO:0031594	neuromuscular junction	6.13E-04	4.57E-02
GO: Cellular Component	GO:0045202	synapse	6.45E-04	4.57E-02
GO: Cellular Component	GO:0098982	GABA-ergic synapse	7.71E-04	4.57E-02
GO: Cellular Component	GO:0099501	exocytic vesicle membrane	1.33E-03	4.73E-02
GO: Cellular Component	GO:0030672	synaptic vesicle membrane	1.33E-03	4.73E-02
Pathway	1339117	Phase 0 - rapid depolarisation	9.52E-05	2.07E-02
Pathway	1269145	Neurotoxicity of clostridium toxins	1.57E-04	2.07E-02
Pathway	1269868	Muscle contraction	5.65E-04	4.95E-02
Disease	C0018799	Heart Diseases	1.19E-03	2.73E-02
Disease	C0345958	Large cell carcinoma of lung	1.36E-03	2.73E-02
Disease	cv:C3279564	Osteogenesis imperfecta, type VI	1.71E-03	2.73E-02
Disease	613105	CHOROIDAL DYSTROPHY, CENTRAL AREOLAR 2	1.71E-03	2.73E-02
Disease	C1842149	Spondyloepiphyseal Dysplasia, Kimberley Type	1.71E-03	2.73E-02
Disease	C1848137	EPILEPSY, FEMALE-RESTRICTED, WITH MENTAL RETARDATION (disorder)	1.71E-03	2.73E-02
Disease	cv:C1842149	Spondyloepiphyseal dysplasia, kimberley type	1.71E-03	2.73E-02
Disease	C3807697	Bilateral foot drop	1.71E-03	2.73E-02
Disease	C0423682	Low Back Pain, Mechanical	1.71E-03	2.73E-02
Disease	cv:C4015038	Myasthenic syndrome, congenital, 7, presynaptic	1.71E-03	2.73E-02
Disease	C2678484	Long Qt Syndrome 10	1.71E-03	2.73E-02
Disease	611819	LONG QT SYNDROME 10	1.71E-03	2.73E-02
Disease	616040	MYASTHENIC SYNDROME, CONGENITAL, 7, PRESYNAPTIC	1.71E-03	2.73E-02
Disease	cv:C4085590	Cone-rod dystrophy	1.71E-03	2.73E-02
Disease	C1850569	Nemaline Myopathy 2	1.71E-03	2.73E-02
Disease	612813	SPONDYLOEPIMETAPHYSEAL DYSPLASIA, AGGRECAN TYPE	1.71E-03	2.73E-02
Disease	cv:C1850569	Nemaline myopathy 2	1.71E-03	2.73E-02
Disease	cv:C2678484	Long QT syndrome 10	1.71E-03	2.73E-02
Disease	608361	SPONDYLOEPIPHYSEAL DYSPLASIA, KIMBERLEY TYPE	1.71E-03	2.73E-02
Disease	cv:C2750786	Muscular dystrophy, congenital, due to integrin alpha-7 deficiency	1.71E-03	2.73E-02
Disease	cv:C1842914	Macular dystrophy, vitelliform, adult-onset	1.71E-03	2.73E-02
Disease	613982	OSTEOGENESIS IMPERFECTA, TYPE VI	1.71E-03	2.73E-02
Disease	C3203523	Acute repetitive seizure	1.71E-03	2.73E-02
Disease	C1864445	Histiocytosis with joint contractures and sensorineural deafness	1.71E-03	2.73E-02
Disease	608161	MACULAR DYSTROPHY, VITELLIFORM, 3	1.71E-03	2.73E-02
Disease	256030	NEMALINE MYOPATHY 2	1.71E-03	2.73E-02
Disease	C4509881	Multifocal pattern dystrophy of retinal pigment epithelium simulating fundus flavimaculatus	1.71E-03	2.73E-02
Disease	C1850569	Nemaline Myopathy 2	1.71E-03	2.73E-02
Disease	C1141890	Congenital long QT syndrome	1.71E-03	2.73E-02
Disease	cv:C2751290	Choroidal dystrophy, central areolar 2	1.71E-03	2.73E-02
Disease	cv:C3665488	Osteochondritis dissecans	1.71E-03	2.73E-02
Disease	C2748544	Spondyloepimetaphyseal Dysplasia, Aggrecan Type	1.71E-03	2.73E-02
Disease	169150	MACULAR DYSTROPHY, PATTERNED, 1	1.71E-03	2.73E-02
Disease	C3539010	PEROXISOME BIOGENESIS DISORDER, COMPLEMENTATION GROUP F	1.71E-03	2.73E-02
Disease	165800	SHORT STATURE AND ADVANCED BONE AGE, WITH OR WITHOUT EARLY-ONSET OSTEOARTHRITIS AND/OR OS	1.71E-03	2.73E-02
Disease	300088	EPILEPTIC ENCEPHALOPATHY, EARLY INFANTILE, 9	1.71E-03	2.73E-02
Disease	C4551999	MACULAR DYSTROPHY, PATTERNED, 1	1.71E-03	2.73E-02
Disease	C1842149	Spondyloepiphyseal Dysplasia, Kimberley Type	1.71E-03	2.73E-02
Disease	C0019618	Histiocytosis	1.71E-03	2.73E-02
Disease	C2750786	Muscular Dystrophy, Congenital, Due To Integrin Alpha-7 Deficiency	1.71E-03	2.73E-02
Disease	C3665488	Familial Osteochondritis Dissecans	1.71E-03	2.73E-02
Disease	C0019625	Sinus histiocytosis	1.71E-03	2.73E-02
Disease	C0751884	Congenital Myasthenic Syndromes, Presynaptic	1.71E-03	2.73E-02
Disease	C4015038	MYASTHENIC SYNDROME, CONGENITAL, 7, PRESYNAPTIC	1.71E-03	2.73E-02
Disease	C2751290	CHOROIDAL DYSTROPHY, CENTRAL AREOLAR 2	1.71E-03	2.73E-02
Disease	cv:C1848137	Early infantile epileptic encephalopathy 9	1.71E-03	2.73E-02
Disease	cv:C4551999	Macular dystrophy, patterned, 1	1.71E-03	2.73E-02
Disease	cv:C2748544	Spondyloepimetaphyseal dysplasia, Aggrecan type	1.71E-03	2.73E-02
Disease	613204	MUSCULAR DYSTROPHY, CONGENITAL, DUE TO INTEGRIN ALPHA-7 DEFICIENCY	1.71E-03	2.73E-02
Disease	cv:C1864445	Histiocytosis-lymphadenopathy plus syndrome	1.71E-03	2.73E-02
Disease	C3279564	Osteogenesis Imperfecta, Type VI	1.71E-03	2.73E-02
Disease	602782	HISTIOCYTOSIS-LYMPHADENOPATHY PLUS SYNDROME	1.71E-03	2.73E-02
Disease	C0010043	Corneal Ulcer	3.41E-03	4.22E-02
Disease	C2676766	Osteopetrosis, Autosomal Recessive 7	3.41E-03	4.22E-02
Disease	C0020555	Hypertrichosis	3.41E-03	4.22E-02
Disease	608133	RETINITIS PIGMENTOSA 7	3.41E-03	4.22E-02
Disease	C1263858	Muscular dystrophy congenital, merosin negative	3.41E-03	4.22E-02
Disease	C1842475	Retinitis Pigmentosa 7	3.41E-03	4.22E-02
Disease	C0432262	Dysosteosclerosis	3.41E-03	4.22E-02
Disease	C0398620	Alpha-2-antiplasmin deficiency	3.41E-03	4.22E-02
Disease	cv:C1842475	Retinitis pigmentosa 7	3.41E-03	4.22E-02
Disease	C0730366	Rod dystrophy	3.41E-03	4.22E-02
Disease	C0085700	Chondromalacia	3.41E-03	4.22E-02
Disease	C0272274	Familial hemorrhagic diathesis	3.41E-03	4.22E-02
Disease	C1848137	EPILEPSY, FEMALE-RESTRICTED, WITH MENTAL RETARDATION (disorder)	3.41E-03	4.22E-02
Disease	C4288779	Major Congenital Anomaly	3.41E-03	4.22E-02
Disease	C2751855	Hypomyelination, Global Cerebral	3.41E-03	4.22E-02
Disease	C0270960	Congenital myopathy (disorder)	3.68E-03	4.49E-02

Abbreviations: rsFC, resting-state functional connectivity; A4ll, lower limb region of area 4; GO, gene ontology.

Enrichment results of the genes related to rsFC of the A1/2/3ulhf				
**Category**	**ID**	**Name**	***P* value**	***q* value (FDR-BH correction)**
GO: Molecular Function	GO:0022857	transmembrane transporter activity	4.75E-11	6.31E-08
GO: Molecular Function	GO:0005215	transporter activity	2.94E-10	1.95E-07
GO: Molecular Function	GO:0015267	channel activity	5.96E-10	2.10E-07
GO: Molecular Function	GO:0022803	passive transmembrane transporter activity	6.30E-10	2.10E-07
GO: Molecular Function	GO:0046873	metal ion transmembrane transporter activity	1.18E-09	3.12E-07
GO: Molecular Function	GO:0005216	ion channel activity	2.21E-09	4.89E-07
GO: Molecular Function	GO:0015075	ion transmembrane transporter activity	3.21E-09	6.10E-07
GO: Molecular Function	GO:0015318	inorganic molecular entity transmembrane transporter activity	6.39E-09	1.06E-06
GO: Molecular Function	GO:0005261	cation channel activity	9.24E-09	1.36E-06
GO: Molecular Function	GO:0016247	channel regulator activity	5.52E-08	7.13E-06
GO: Molecular Function	GO:0022836	gated channel activity	5.90E-08	7.13E-06
GO: Molecular Function	GO:0022839	ion gated channel activity	7.50E-08	8.31E-06
GO: Molecular Function	GO:0008324	cation transmembrane transporter activity	1.69E-07	1.72E-05
GO: Molecular Function	GO:0022890	inorganic cation transmembrane transporter activity	2.24E-07	2.13E-05
GO: Molecular Function	GO:0005272	sodium channel activity	1.66E-06	1.47E-04
GO: Molecular Function	GO:0017080	sodium channel regulator activity	5.98E-06	4.97E-04
GO: Molecular Function	GO:0042165	neurotransmitter binding	7.98E-06	6.24E-04
GO: Molecular Function	GO:0008092	cytoskeletal protein binding	1.24E-05	9.15E-04
GO: Molecular Function	GO:0030594	neurotransmitter receptor activity	1.36E-05	9.50E-04
GO: Molecular Function	GO:0099106	ion channel regulator activity	1.65E-05	1.10E-03
GO: Molecular Function	GO:0005244	voltage-gated ion channel activity	2.52E-05	1.59E-03
GO: Molecular Function	GO:0022832	voltage-gated channel activity	2.67E-05	1.61E-03
GO: Molecular Function	GO:0098960	postsynaptic neurotransmitter receptor activity	2.85E-05	1.65E-03
GO: Molecular Function	GO:0015077	monovalent inorganic cation transmembrane transporter activity	2.98E-05	1.65E-03
GO: Molecular Function	GO:0015085	calcium ion transmembrane transporter activity	3.24E-05	1.72E-03
GO: Molecular Function	GO:0015081	sodium ion transmembrane transporter activity	4.70E-05	2.40E-03
GO: Molecular Function	GO:0099095	ligand-gated anion channel activity	8.39E-05	4.13E-03
GO: Molecular Function	GO:0019871	sodium channel inhibitor activity	1.15E-04	5.41E-03
GO: Molecular Function	GO:0022843	voltage-gated cation channel activity	1.18E-04	5.41E-03
GO: Molecular Function	GO:0005267	potassium channel activity	1.26E-04	5.59E-03
GO: Molecular Function	GO:0005001	transmembrane receptor protein tyrosine phosphatase activity	2.90E-04	1.17E-02
GO: Molecular Function	GO:0019198	transmembrane receptor protein phosphatase activity	2.90E-04	1.17E-02
GO: Molecular Function	GO:0050811	GABA receptor binding	2.90E-04	1.17E-02
GO: Molecular Function	GO:0005089	Rho guanyl-nucleotide exchange factor activity	3.18E-04	1.24E-02
GO: Molecular Function	GO:0005509	calcium ion binding	3.46E-04	1.32E-02
GO: Molecular Function	GO:0015079	potassium ion transmembrane transporter activity	3.82E-04	1.41E-02
GO: Molecular Function	GO:0005251	delayed rectifier potassium channel activity	3.93E-04	1.41E-02
GO: Molecular Function	GO:0086006	voltage-gated sodium channel activity involved in cardiac muscle cell action potential	4.75E-04	1.66E-02
GO: Molecular Function	GO:0098631	cell adhesion mediator activity	6.11E-04	2.08E-02
GO: Molecular Function	GO:0008200	ion channel inhibitor activity	7.96E-04	2.64E-02
GO: Molecular Function	GO:0044325	ion channel binding	8.50E-04	2.76E-02
GO: Molecular Function	GO:0005516	calmodulin binding	9.72E-04	3.01E-02
GO: Molecular Function	GO:0043177	organic acid binding	9.74E-04	3.01E-02
GO: Molecular Function	GO:0022851	GABA-gated chloride ion channel activity	1.01E-03	3.05E-02
GO: Molecular Function	GO:0016248	channel inhibitor activity	1.06E-03	3.13E-02
GO: Molecular Function	GO:0005227	calcium activated cation channel activity	1.17E-03	3.39E-02
GO: Molecular Function	GO:0031406	carboxylic acid binding	1.25E-03	3.49E-02
GO: Molecular Function	GO:0016597	amino acid binding	1.27E-03	3.49E-02
GO: Molecular Function	GO:0005230	extracellular ligand-gated ion channel activity	1.30E-03	3.49E-02
GO: Molecular Function	GO:0005544	calcium-dependent phospholipid binding	1.40E-03	3.49E-02
GO: Molecular Function	GO:0022835	transmitter-gated channel activity	1.41E-03	3.49E-02
GO: Molecular Function	GO:0022824	transmitter-gated ion channel activity	1.41E-03	3.49E-02
GO: Molecular Function	GO:0022852	glycine-gated chloride ion channel activity	1.42E-03	3.49E-02
GO: Molecular Function	GO:0008481	sphinganine kinase activity	1.42E-03	3.49E-02
GO: Molecular Function	GO:0098634	cell-matrix adhesion mediator activity	1.57E-03	3.80E-02
GO: Molecular Function	GO:0005248	voltage-gated sodium channel activity	1.61E-03	3.80E-02
GO: Molecular Function	GO:0005262	calcium channel activity	1.63E-03	3.80E-02
GO: Molecular Function	GO:0042301	phosphate ion binding	1.82E-03	4.10E-02
GO: Molecular Function	GO:0016594	glycine binding	1.82E-03	4.10E-02
GO: Molecular Function	GO:0008289	lipid binding	2.25E-03	4.99E-02
GO: Biological Process	GO:0099537	trans-synaptic signaling	2.06E-14	5.49E-11
GO: Biological Process	GO:0007267	cell-cell signaling	3.06E-14	5.49E-11
GO: Biological Process	GO:0098916	anterograde trans-synaptic signaling	3.48E-14	5.49E-11
GO: Biological Process	GO:0007268	chemical synaptic transmission	3.48E-14	5.49E-11
GO: Biological Process	GO:0099536	synaptic signaling	4.47E-14	5.63E-11
GO: Biological Process	GO:0006811	ion transport	2.76E-13	2.90E-10
GO: Biological Process	GO:0023061	signal release	2.51E-11	2.26E-08
GO: Biological Process	GO:0034220	ion transmembrane transport	5.54E-11	4.35E-08
GO: Biological Process	GO:0055085	transmembrane transport	6.21E-11	4.35E-08
GO: Biological Process	GO:0050804	modulation of chemical synaptic transmission	1.59E-10	9.73E-08
GO: Biological Process	GO:0099177	regulation of trans-synaptic signaling	1.70E-10	9.73E-08
GO: Biological Process	GO:0006812	cation transport	4.91E-10	2.58E-07
GO: Biological Process	GO:0030001	metal ion transport	5.62E-10	2.73E-07
GO: Biological Process	GO:0043269	regulation of ion transport	1.49E-09	6.70E-07
GO: Biological Process	GO:0098660	inorganic ion transmembrane transport	3.96E-09	1.66E-06
GO: Biological Process	GO:0044057	regulation of system process	1.48E-08	5.84E-06
GO: Biological Process	GO:0009914	hormone transport	1.12E-07	4.06E-05
GO: Biological Process	GO:0098662	inorganic cation transmembrane transport	1.21E-07	4.06E-05
GO: Biological Process	GO:0098655	cation transmembrane transport	1.22E-07	4.06E-05
GO: Biological Process	GO:0046879	hormone secretion	2.12E-07	6.69E-05
GO: Biological Process	GO:0140352	export from cell	2.79E-07	8.39E-05
GO: Biological Process	GO:0034762	regulation of transmembrane transport	3.37E-07	9.65E-05
GO: Biological Process	GO:0042391	regulation of membrane potential	4.86E-07	1.29E-04
GO: Biological Process	GO:0048699	generation of neurons	4.92E-07	1.29E-04
GO: Biological Process	GO:0022008	neurogenesis	5.11E-07	1.29E-04
GO: Biological Process	GO:1903530	regulation of secretion by cell	7.35E-07	1.78E-04
GO: Biological Process	GO:0051046	regulation of secretion	8.54E-07	1.95E-04
GO: Biological Process	GO:0032940	secretion by cell	8.65E-07	1.95E-04
GO: Biological Process	GO:0007155	cell adhesion	1.03E-06	2.23E-04
GO: Biological Process	GO:0022610	biological adhesion	1.25E-06	2.62E-04
GO: Biological Process	GO:0048666	neuron development	1.31E-06	2.66E-04
GO: Biological Process	GO:0046883	regulation of hormone secretion	1.37E-06	2.70E-04
GO: Biological Process	GO:0030182	neuron differentiation	1.98E-06	3.77E-04
GO: Biological Process	GO:0099565	chemical synaptic transmission, postsynaptic	2.05E-06	3.80E-04
GO: Biological Process	GO:0046903	secretion	2.26E-06	3.97E-04
GO: Biological Process	GO:0099643	signal release from synapse	2.33E-06	3.97E-04
GO: Biological Process	GO:0007269	neurotransmitter secretion	2.33E-06	3.97E-04
GO: Biological Process	GO:0006836	neurotransmitter transport	2.73E-06	4.51E-04
GO: Biological Process	GO:2000649	regulation of sodium ion transmembrane transporter activity	2.80E-06	4.51E-04
GO: Biological Process	GO:1902305	regulation of sodium ion transmembrane transport	2.87E-06	4.51E-04
GO: Biological Process	GO:0031175	neuron projection development	2.93E-06	4.51E-04
GO: Biological Process	GO:0010038	response to metal ion	3.26E-06	4.90E-04
GO: Biological Process	GO:0046928	regulation of neurotransmitter secretion	3.60E-06	5.29E-04
GO: Biological Process	GO:0035637	multicellular organismal signaling	4.67E-06	6.70E-04
GO: Biological Process	GO:0006816	calcium ion transport	5.62E-06	7.87E-04
GO: Biological Process	GO:0010817	regulation of hormone levels	6.63E-06	9.08E-04
GO: Biological Process	GO:0015672	monovalent inorganic cation transport	7.12E-06	9.55E-04
GO: Biological Process	GO:0002028	regulation of sodium ion transport	7.49E-06	9.84E-04
GO: Biological Process	GO:0034765	regulation of ion transmembrane transport	8.31E-06	1.05E-03
GO: Biological Process	GO:0070838	divalent metal ion transport	8.31E-06	1.05E-03
GO: Biological Process	GO:1903035	negative regulation of response to wounding	9.54E-06	1.18E-03
GO: Biological Process	GO:0030030	cell projection organization	9.79E-06	1.19E-03
GO: Biological Process	GO:0003013	circulatory system process	1.08E-05	1.28E-03
GO: Biological Process	GO:0072511	divalent inorganic cation transport	1.12E-05	1.31E-03
GO: Biological Process	GO:0120036	plasma membrane bounded cell projection organization	1.19E-05	1.36E-03
GO: Biological Process	GO:0070588	calcium ion transmembrane transport	1.43E-05	1.60E-03
GO: Biological Process	GO:0001508	action potential	1.44E-05	1.60E-03
GO: Biological Process	GO:0007156	homophilic cell adhesion via plasma membrane adhesion molecules	1.52E-05	1.66E-03

GO: Biological Process	GO:0008015	blood circulation	1.66E-05	1.78E-03
GO: Biological Process	GO:0007610	behavior	1.83E-05	1.93E-03
GO: Biological Process	GO:0048812	neuron projection morphogenesis	1.97E-05	2.03E-03
GO: Biological Process	GO:0010959	regulation of metal ion transport	2.26E-05	2.30E-03
GO: Biological Process	GO:0060078	regulation of postsynaptic membrane potential	2.39E-05	2.39E-03
GO: Biological Process	GO:0050877	nervous system process	2.70E-05	2.66E-03
GO: Biological Process	GO:0120039	plasma membrane bounded cell projection morphogenesis	3.29E-05	3.17E-03
GO: Biological Process	GO:0010975	regulation of neuron projection development	3.34E-05	3.17E-03
GO: Biological Process	GO:0035725	sodium ion transmembrane transport	3.37E-05	3.17E-03
GO: Biological Process	GO:0032990	cell part morphogenesis	3.75E-05	3.48E-03
GO: Biological Process	GO:0048858	cell projection morphogenesis	3.88E-05	3.55E-03
GO: Biological Process	GO:0043270	positive regulation of ion transport	3.97E-05	3.58E-03
GO: Biological Process	GO:0051050	positive regulation of transport	4.34E-05	3.86E-03
GO: Biological Process	GO:0006813	potassium ion transport	4.46E-05	3.91E-03
GO: Biological Process	GO:0001505	regulation of neurotransmitter levels	4.99E-05	4.31E-03
GO: Biological Process	GO:0032412	regulation of ion transmembrane transporter activity	5.34E-05	4.55E-03
GO: Biological Process	GO:0071248	cellular response to metal ion	6.06E-05	5.09E-03
GO: Biological Process	GO:0051588	regulation of neurotransmitter transport	6.34E-05	5.26E-03
GO: Biological Process	GO:0051592	response to calcium ion	6.54E-05	5.36E-03
GO: Biological Process	GO:1904062	regulation of cation transmembrane transport	6.95E-05	5.62E-03
GO: Biological Process	GO:0032409	regulation of transporter activity	7.28E-05	5.81E-03
GO: Biological Process	GO:0022898	regulation of transmembrane transporter activity	8.22E-05	6.40E-03
GO: Biological Process	GO:0010035	response to inorganic substance	8.25E-05	6.40E-03
GO: Biological Process	GO:0032989	cellular component morphogenesis	8.33E-05	6.40E-03
GO: Biological Process	GO:0098656	anion transmembrane transport	1.07E-04	8.05E-03
GO: Biological Process	GO:0006820	anion transport	1.08E-04	8.05E-03
GO: Biological Process	GO:0006814	sodium ion transport	1.09E-04	8.05E-03
GO: Biological Process	GO:0003012	muscle system process	1.10E-04	8.05E-03
GO: Biological Process	GO:0015711	organic anion transport	1.26E-04	9.09E-03
GO: Biological Process	GO:0014888	striated muscle adaptation	1.27E-04	9.09E-03
GO: Biological Process	GO:0051960	regulation of nervous system development	1.29E-04	9.09E-03
GO: Biological Process	GO:0000902	cell morphogenesis	1.31E-04	9.09E-03
GO: Biological Process	GO:0015718	monocarboxylic acid transport	1.31E-04	9.09E-03
GO: Biological Process	GO:0010976	positive regulation of neuron projection development	1.33E-04	9.13E-03
GO: Biological Process	GO:0098609	cell-cell adhesion	1.36E-04	9.21E-03
GO: Biological Process	GO:0050796	regulation of insulin secretion	1.41E-04	9.43E-03
GO: Biological Process	GO:0030073	insulin secretion	1.50E-04	9.98E-03
GO: Biological Process	GO:0046942	carboxylic acid transport	1.52E-04	1.00E-02
GO: Biological Process	GO:0015849	organic acid transport	1.59E-04	1.04E-02
GO: Biological Process	GO:0030029	actin filament-based process	1.64E-04	1.05E-02
GO: Biological Process	GO:0031344	regulation of cell projection organization	1.68E-04	1.06E-02
GO: Biological Process	GO:0071805	potassium ion transmembrane transport	1.69E-04	1.06E-02
GO: Biological Process	GO:0098962	regulation of postsynaptic neurotransmitter receptor activity	1.69E-04	1.06E-02
GO: Biological Process	GO:0050890	cognition	1.76E-04	1.09E-02
GO: Biological Process	GO:0019228	neuronal action potential	1.82E-04	1.11E-02
GO: Biological Process	GO:0032102	negative regulation of response to external stimulus	1.84E-04	1.11E-02
GO: Biological Process	GO:0045664	regulation of neuron differentiation	1.86E-04	1.12E-02
GO: Biological Process	GO:0030072	peptide hormone secretion	1.91E-04	1.13E-02
GO: Biological Process	GO:0031644	regulation of nervous system process	1.93E-04	1.14E-02
GO: Biological Process	GO:0050767	regulation of neurogenesis	2.07E-04	1.21E-02
GO: Biological Process	GO:0035023	regulation of Rho protein signal transduction	2.20E-04	1.28E-02
GO: Biological Process	GO:0072507	divalent inorganic cation homeostasis	2.32E-04	1.32E-02
GO: Biological Process	GO:0120035	regulation of plasma membrane bounded cell projection organization	2.36E-04	1.32E-02
GO: Biological Process	GO:0032509	endosome transport via multivesicular body sorting pathway	2.36E-04	1.32E-02
GO: Biological Process	GO:0015837	amine transport	2.37E-04	1.32E-02
GO: Biological Process	GO:0090276	regulation of peptide hormone secretion	2.48E-04	1.37E-02
GO: Biological Process	GO:0007626	locomotory behavior	2.62E-04	1.44E-02
GO: Biological Process	GO:0032594	protein transport within lipid bilayer	2.71E-04	1.47E-02
GO: Biological Process	GO:0071985	multivesicular body sorting pathway	2.87E-04	1.55E-02
GO: Biological Process	GO:0071277	cellular response to calcium ion	3.19E-04	1.71E-02
GO: Biological Process	GO:0050771	negative regulation of axonogenesis	3.24E-04	1.72E-02
GO: Biological Process	GO:1903034	regulation of response to wounding	3.60E-04	1.89E-02
GO: Biological Process	GO:1903861	positive regulation of dendrite extension	3.64E-04	1.90E-02
GO: Biological Process	GO:0019226	transmission of nerve impulse	3.87E-04	2.00E-02
GO: Biological Process	GO:0060768	regulation of epithelial cell proliferation involved in prostate gland development	4.14E-04	2.11E-02
GO: Biological Process	GO:0050808	synapse organization	4.15E-04	2.11E-02
GO: Biological Process	GO:0071241	cellular response to inorganic substance	4.28E-04	2.15E-02
GO: Biological Process	GO:0031345	negative regulation of cell projection organization	4.31E-04	2.15E-02
GO: Biological Process	GO:0048589	developmental growth	4.32E-04	2.15E-02
GO: Biological Process	GO:0055074	calcium ion homeostasis	4.36E-04	2.15E-02
GO: Biological Process	GO:0086010	membrane depolarization during action potential	4.43E-04	2.16E-02
GO: Biological Process	GO:0003015	heart process	4.46E-04	2.16E-02
GO: Biological Process	GO:0048585	negative regulation of response to stimulus	4.78E-04	2.30E-02
GO: Biological Process	GO:0044272	sulfur compound biosynthetic process	5.00E-04	2.39E-02
GO: Biological Process	GO:0055065	metal ion homeostasis	5.40E-04	2.55E-02
GO: Biological Process	GO:0042493	response to drug	5.43E-04	2.55E-02
GO: Biological Process	GO:0061045	negative regulation of wound healing	5.45E-04	2.55E-02
GO: Biological Process	GO:0022604	regulation of cell morphogenesis	5.88E-04	2.72E-02
GO: Biological Process	GO:0007411	axon guidance	5.91E-04	2.72E-02
GO: Biological Process	GO:0097484	dendrite extension	6.03E-04	2.74E-02
GO: Biological Process	GO:0060767	epithelial cell proliferation involved in prostate gland development	6.04E-04	2.74E-02
GO: Biological Process	GO:0097485	neuron projection guidance	6.18E-04	2.78E-02
GO: Biological Process	GO:1903859	regulation of dendrite extension	6.50E-04	2.88E-02
GO: Biological Process	GO:0006936	muscle contraction	6.51E-04	2.88E-02
GO: Biological Process	GO:0045665	negative regulation of neuron differentiation	6.52E-04	2.88E-02
GO: Biological Process	GO:0010765	positive regulation of sodium ion transport	6.99E-04	3.04E-02
GO: Biological Process	GO:0060047	heart contraction	7.05E-04	3.04E-02
GO: Biological Process	GO:0061337	cardiac conduction	7.10E-04	3.04E-02
GO: Biological Process	GO:0019233	sensory perception of pain	7.10E-04	3.04E-02
GO: Biological Process	GO:0098742	cell-cell adhesion via plasma-membrane adhesion molecules	7.14E-04	3.04E-02
GO: Biological Process	GO:0050801	ion homeostasis	7.37E-04	3.12E-02
GO: Biological Process	GO:1903305	regulation of regulated secretory pathway	7.49E-04	3.15E-02
GO: Biological Process	GO:0048667	cell morphogenesis involved in neuron differentiation	7.71E-04	3.22E-02
GO: Biological Process	GO:0072503	cellular divalent inorganic cation homeostasis	7.84E-04	3.25E-02
GO: Biological Process	GO:0007525	somatic muscle development	8.05E-04	3.32E-02
GO: Biological Process	GO:0061564	axon development	8.50E-04	3.45E-02
GO: Biological Process	GO:1901685	glutathione derivative metabolic process	8.56E-04	3.45E-02
GO: Biological Process	GO:1901687	glutathione derivative biosynthetic process	8.56E-04	3.45E-02
GO: Biological Process	GO:0051899	membrane depolarization	8.60E-04	3.45E-02
GO: Biological Process	GO:2001257	regulation of cation channel activity	8.85E-04	3.53E-02
GO: Biological Process	GO:0031346	positive regulation of cell projection organization	9.03E-04	3.58E-02
GO: Biological Process	GO:0007611	learning or memory	9.14E-04	3.60E-02
GO: Biological Process	GO:0010977	negative regulation of neuron projection development	9.33E-04	3.65E-02
GO: Biological Process	GO:0016079	synaptic vesicle exocytosis	9.87E-04	3.84E-02
GO: Biological Process	GO:0006874	cellular calcium ion homeostasis	9.93E-04	3.84E-02
GO: Biological Process	GO:0040012	regulation of locomotion	1.08E-03	4.13E-02
GO: Biological Process	GO:0043501	skeletal muscle adaptation	1.09E-03	4.15E-02
GO: Biological Process	GO:0040007	growth	1.11E-03	4.21E-02
GO: Biological Process	GO:0090066	regulation of anatomical structure size	1.11E-03	4.21E-02
GO: Biological Process	GO:0009306	protein secretion	1.14E-03	4.26E-02
GO: Biological Process	GO:0099566	regulation of postsynaptic cytosolic calcium ion concentration	1.15E-03	4.30E-02
GO: Biological Process	GO:0048638	regulation of developmental growth	1.16E-03	4.30E-02
GO: Biological Process	GO:0035592	establishment of protein localization to extracellular region	1.17E-03	4.30E-02
GO: Biological Process	GO:0071407	cellular response to organic cyclic compound	1.20E-03	4.40E-02
GO: Biological Process	GO:1903522	regulation of blood circulation	1.23E-03	4.47E-02
GO: Biological Process	GO:0099601	regulation of neurotransmitter receptor activity	1.25E-03	4.53E-02
GO: Biological Process	GO:2000184	positive regulation of progesterone biosynthetic process	1.29E-03	4.59E-02
GO: Biological Process	GO:0006713	glucocorticoid catabolic process	1.29E-03	4.59E-02
GO: Biological Process	GO:0002790	peptide secretion	1.29E-03	4.59E-02
GO: Biological Process	GO:2001169	regulation of ATP biosynthetic process	1.30E-03	4.62E-02
GO: Biological Process	GO:0060770	negative regulation of epithelial cell proliferation involved in prostate gland development	1.37E-03	4.83E-02
GO: Biological Process	GO:0031099	regeneration	1.38E-03	4.83E-02

GO: Biological Process	GO:0071692	protein localization to extracellular region	1.41E-03	4.91E-02
GO: Biological Process	GO:0006869	lipid transport	1.42E-03	4.92E-02
GO: Biological Process	GO:0030048	actin filament-based movement	1.43E-03	4.94E-02
GO: Biological Process	GO:0040008	regulation of growth	1.45E-03	4.95E-02
GO: Biological Process	GO:0051952	regulation of amine transport	1.45E-03	4.95E-02
GO: Cellular Component	GO:0043005	neuron projection	1.67E-23	1.16E-20
GO: Cellular Component	GO:0030424	axon	2.81E-19	9.79E-17
GO: Cellular Component	GO:0045202	synapse	3.04E-18	7.05E-16
GO: Cellular Component	GO:0036477	somatodendritic compartment	6.79E-15	1.18E-12
GO: Cellular Component	GO:0097060	synaptic membrane	3.94E-14	5.49E-12
GO: Cellular Component	GO:0098978	glutamatergic synapse	3.31E-13	3.84E-11
GO: Cellular Component	GO:0005887	integral component of plasma membrane	1.38E-12	1.37E-10
GO: Cellular Component	GO:0030425	dendrite	2.49E-12	1.79E-10
GO: Cellular Component	GO:0097447	dendritic tree	2.49E-12	1.79E-10
GO: Cellular Component	GO:0031226	intrinsic component of plasma membrane	2.57E-12	1.79E-10
GO: Cellular Component	GO:0098793	presynapse	4.26E-12	2.70E-10
GO: Cellular Component	GO:0099699	integral component of synaptic membrane	1.38E-11	8.03E-10
GO: Cellular Component	GO:0099240	intrinsic component of synaptic membrane	2.35E-11	1.26E-09
GO: Cellular Component	GO:0043025	neuronal cell body	3.05E-11	1.52E-09
GO: Cellular Component	GO:0098794	postsynapse	3.29E-11	1.53E-09
GO: Cellular Component	GO:0044297	cell body	6.78E-11	2.95E-09
GO: Cellular Component	GO:0045211	postsynaptic membrane	2.73E-10	1.12E-08
GO: Cellular Component	GO:0034702	ion channel complex	1.18E-09	4.56E-08
GO: Cellular Component	GO:1902495	transmembrane transporter complex	2.37E-09	8.63E-08
GO: Cellular Component	GO:0034703	cation channel complex	2.48E-09	8.63E-08
GO: Cellular Component	GO:1990351	transporter complex	4.44E-09	1.47E-07
GO: Cellular Component	GO:0030054	cell junction	7.59E-09	2.40E-07
GO: Cellular Component	GO:0098936	intrinsic component of postsynaptic membrane	1.49E-08	4.51E-07
GO: Cellular Component	GO:0150034	distal axon	1.89E-08	5.47E-07
GO: Cellular Component	GO:0099055	integral component of postsynaptic membrane	2.50E-08	6.96E-07
GO: Cellular Component	GO:0043204	perikaryon	4.13E-07	1.11E-05
GO: Cellular Component	GO:0098889	intrinsic component of presynaptic membrane	5.77E-07	1.49E-05
GO: Cellular Component	GO:0099056	integral component of presynaptic membrane	6.70E-07	1.66E-05
GO: Cellular Component	GO:0043679	axon terminus	1.23E-06	2.94E-05
GO: Cellular Component	GO:0042734	presynaptic membrane	1.66E-06	3.84E-05
GO: Cellular Component	GO:0014704	intercalated disc	2.44E-06	5.49E-05
GO: Cellular Component	GO:0098982	GABA-ergic synapse	3.79E-06	8.24E-05
GO: Cellular Component	GO:0044305	calyx of Held	4.53E-06	9.55E-05
GO: Cellular Component	GO:0044306	neuron projection terminus	5.83E-06	1.19E-04
GO: Cellular Component	GO:0034705	potassium channel complex	8.00E-06	1.59E-04
GO: Cellular Component	GO:0044291	cell-cell contact zone	9.08E-06	1.76E-04
GO: Cellular Component	GO:0008076	voltage-gated potassium channel complex	1.17E-05	2.20E-04
GO: Cellular Component	GO:0098984	neuron to neuron synapse	2.18E-05	3.99E-04
GO: Cellular Component	GO:0099572	postsynaptic specialization	3.26E-05	5.81E-04
GO: Cellular Component	GO:0042383	sarcolemma	3.96E-05	6.78E-04
GO: Cellular Component	GO:0098797	plasma membrane protein complex	3.99E-05	6.78E-04
GO: Cellular Component	GO:0043197	dendritic spine	4.48E-05	7.43E-04
GO: Cellular Component	GO:0044309	neuron spine	6.08E-05	9.83E-04
GO: Cellular Component	GO:0099060	integral component of postsynaptic specialization membrane	7.00E-05	1.11E-03
GO: Cellular Component	GO:0001518	voltage-gated sodium channel complex	7.64E-05	1.18E-03
GO: Cellular Component	GO:0097457	hippocampal mossy fiber	9.08E-05	1.37E-03
GO: Cellular Component	GO:0044302	dentate gyrus mossy fiber	1.05E-04	1.56E-03
GO: Cellular Component	GO:0098948	intrinsic component of postsynaptic specialization membrane	1.25E-04	1.81E-03
GO: Cellular Component	GO:0034706	sodium channel complex	1.34E-04	1.91E-03
GO: Cellular Component	GO:0032589	neuron projection membrane	1.62E-04	2.25E-03
GO: Cellular Component	GO:0014069	postsynaptic density	2.10E-04	2.81E-03
GO: Cellular Component	GO:0032279	asymmetric synapse	2.10E-04	2.81E-03
GO: Cellular Component	GO:0099634	postsynaptic specialization membrane	2.76E-04	3.62E-03
GO: Cellular Component	GO:0098686	hippocampal mossy fiber to CA3 synapse	2.98E-04	3.77E-03
GO: Cellular Component	GO:0097464	thorny excrescence	2.98E-04	3.77E-03
GO: Cellular Component	GO:1990026	hippocampal mossy fiber expansion	3.93E-04	4.89E-03
GO: Cellular Component	GO:0098685	Schaffer collateral - CA1 synapse	6.03E-04	7.37E-03
GO: Cellular Component	GO:0043195	terminal bouton	6.88E-04	8.26E-03
GO: Cellular Component	GO:0070382	exocytic vesicle	7.85E-04	9.26E-03
GO: Cellular Component	GO:0015629	actin cytoskeleton	8.33E-04	9.53E-03
GO: Cellular Component	GO:0099061	integral component of postsynaptic density membrane	8.35E-04	9.53E-03
GO: Cellular Component	GO:0005911	cell-cell junction	8.52E-04	9.57E-03
GO: Cellular Component	GO:0030426	growth cone	9.48E-04	1.05E-02
GO: Cellular Component	GO:0099568	cytoplasmic region	1.03E-03	1.12E-02
GO: Cellular Component	GO:0009986	cell surface	1.13E-03	1.21E-02
GO: Cellular Component	GO:0030427	site of polarized growth	1.22E-03	1.25E-02
GO: Cellular Component	GO:0034681	integrin alpha11-beta1 complex	1.25E-03	1.25E-02
GO: Cellular Component	GO:0034677	integrin alpha7-beta1 complex	1.25E-03	1.25E-02
GO: Cellular Component	GO:0043235	receptor complex	1.25E-03	1.25E-02
GO: Cellular Component	GO:0032590	dendrite membrane	1.25E-03	1.25E-02
GO: Cellular Component	GO:0099146	intrinsic component of postsynaptic density membrane	1.49E-03	1.45E-02
GO: Cellular Component	GO:0045121	membrane raft	1.50E-03	1.45E-02
GO: Cellular Component	GO:0098857	membrane microdomain	1.55E-03	1.48E-02
GO: Cellular Component	GO:0060076	excitatory synapse	1.63E-03	1.53E-02
GO: Cellular Component	GO:0098796	membrane protein complex	1.90E-03	1.77E-02
GO: Cellular Component	GO:0008021	synaptic vesicle	2.04E-03	1.87E-02
GO: Cellular Component	GO:0031594	neuromuscular junction	2.13E-03	1.92E-02
GO: Cellular Component	GO:0098805	whole membrane	2.49E-03	2.20E-02
GO: Cellular Component	GO:0098589	membrane region	2.50E-03	2.20E-02
GO: Cellular Component	GO:0031256	leading edge membrane	2.79E-03	2.43E-02
GO: Cellular Component	GO:0016342	catenin complex	2.94E-03	2.53E-02
GO: Cellular Component	GO:0032281	AMPA glutamate receptor complex	3.43E-03	2.91E-02
GO: Cellular Component	GO:1902711	GABA-A receptor complex	3.67E-03	3.08E-02
GO: Cellular Component	GO:0005938	cell cortex	3.82E-03	3.17E-02
GO: Cellular Component	GO:0042470	melanosome	4.05E-03	3.28E-02
GO: Cellular Component	GO:0048770	pigment granule	4.05E-03	3.28E-02
GO: Cellular Component	GO:0031252	cell leading edge	4.17E-03	3.34E-02
GO: Cellular Component	GO:0005884	actin filament	4.50E-03	3.56E-02
GO: Cellular Component	GO:0030018	Z disc	4.69E-03	3.67E-02
GO: Cellular Component	GO:0099501	exocytic vesicle membrane	5.01E-03	3.83E-02
GO: Cellular Component	GO:0030672	synaptic vesicle membrane	5.01E-03	3.83E-02
GO: Cellular Component	GO:0043083	synaptic cleft	5.37E-03	4.02E-02
GO: Cellular Component	GO:1902710	GABA receptor complex	5.37E-03	4.02E-02
GO: Cellular Component	GO:0030016	myofibril	5.63E-03	4.17E-02
GO: Cellular Component	GO:0031253	cell projection membrane	5.84E-03	4.28E-02
GO: Cellular Component	GO:0030017	sarcomere	5.90E-03	4.28E-02
GO: Cellular Component	GO:0098839	postsynaptic density membrane	6.12E-03	4.39E-02
GO: Cellular Component	GO:0019897	extrinsic component of plasma membrane	6.34E-03	4.50E-02
GO: Cellular Component	GO:0030863	cortical cytoskeleton	6.66E-03	4.68E-02
GO: Cellular Component	GO:0030175	filopodium	6.72E-03	4.68E-02
GO: Cellular Component	GO:0070032	synaptobrevin 2-SNAP-25-syntaxin-1a-complexin I complex	6.88E-03	4.74E-02
GO: Cellular Component	GO:0030133	transport vesicle	7.02E-03	4.79E-02
GO: Cellular Component	GO:0034707	chloride channel complex	7.40E-03	4.97E-02
GO: Cellular Component	GO:0031902	late endosome membrane	7.43E-03	4.97E-02
Pathway	1268763	Neuronal System	2.44E-13	5.33E-10
Pathway	M68	Regulation of RhoA activity	7.26E-08	5.28E-05
Pathway	138070	Regulation of RhoA activity	7.26E-08	5.28E-05
Pathway	M2890	Calcium signaling pathway	1.58E-07	8.62E-05
Pathway	83050	Calcium signaling pathway	2.64E-07	1.15E-04
Pathway	1268766	Transmission across Chemical Synapses	5.22E-07	1.90E-04
Pathway	1339117	Phase 0 - rapid depolarisation	8.43E-07	2.63E-04
Pathway	777534	Insulin secretion	2.60E-06	7.10E-04
Pathway	1268830	Voltage gated Potassium channels	3.57E-06	8.64E-04
Pathway	908257	Adrenergic signaling in cardiomyocytes	6.18E-06	1.35E-03
Pathway	948277	Inflammatory mediator regulation of TRP channels	1.28E-05	2.53E-03
Pathway	1268795	Trafficking of AMPA receptors	1.62E-05	2.71E-03
Pathway	1268794	Glutamate Binding, Activation of AMPA Receptors and Synaptic Plasticity	1.62E-05	2.71E-03

Pathway	M7761	Melanogenesis	2.04E-05	2.97E-03
Pathway	83092	Melanogenesis	2.04E-05	2.97E-03
Pathway	1269868	Muscle contraction	7.13E-05	9.35E-03
Pathway	1339115	Cardiac conduction	7.29E-05	9.35E-03
Pathway	1268821	Potassium Channels	7.95E-05	9.63E-03
Pathway	537443	Retrograde endocannabinoid signaling	8.82E-05	1.01E-02
Pathway	137953	Role of Calcineurin-dependent NFAT signaling in lymphocytes	9.33E-05	1.02E-02
Pathway	1268786	Neurotransmitter Receptor Binding And Downstream Transmission In The Postsynaptic Cell	1.10E-04	1.11E-02
Pathway	1269144	Uptake and actions of bacterial toxins	1.12E-04	1.11E-02
Pathway	M113	Role of Calcineurin-dependent NFAT signaling in lymphocytes	1.27E-04	1.21E-02
Pathway	1017634	cAMP signaling pathway	1.43E-04	1.30E-02
Pathway	M15181	Regulation of PGC-1a	1.51E-04	1.32E-02
Pathway	83085	Long-term potentiation	1.66E-04	1.39E-02
Pathway	M13380	Neuroactive ligand-receptor interaction	2.02E-04	1.63E-02
Pathway	M3115	Long-term potentiation	2.41E-04	1.88E-02
Pathway	83053	Neuroactive ligand-receptor interaction	2.78E-04	2.09E-02
Pathway	1269950	Ion channel transport	2.88E-04	2.09E-02
Pathway	213307	Endocrine and other factor-regulated calcium reabsorption	2.96E-04	2.09E-02
Pathway	1269145	Neurotoxicity of clostridium toxins	3.28E-04	2.24E-02
Pathway	946598	Thyroid hormone signaling pathway	3.59E-04	2.38E-02
Pathway	154409	Gastric acid secretion	4.26E-04	2.74E-02
Pathway	153376	Salivary secretion	4.79E-04	2.99E-02
Pathway	142435	glutathione-mediated detoxification	5.19E-04	3.11E-02
Pathway	552665	Morphine addiction	5.28E-04	3.11E-02
Pathway	83091	GnRH signaling pathway	5.80E-04	3.32E-02
Pathway	1270213	Glutathione conjugation	5.94E-04	3.32E-02
Pathway	1269588	Ca-dependent events	7.01E-04	3.73E-02
Pathway	1270325	Interaction between L1 and Ankyrins	7.01E-04	3.73E-02
Pathway	83105	Pathways in cancer	7.93E-04	4.12E-02
Pathway	1269345	Platelet calcium homeostasis	8.42E-04	4.23E-02
Pathway	547607	Amphetamine addiction	8.66E-04	4.23E-02
Pathway	1272485	Aldosterone synthesis and secretion	8.74E-04	4.23E-02
Pathway	1339121	Ion homeostasis	1.00E-03	4.74E-02
Pathway	1269621	Signaling by Activin	1.02E-03	4.75E-02
Pathway	213818	Glutamatergic synapse	1.05E-03	4.78E-02
Disease	C4316903	Absence Seizures	6.95E-08	5.42E-04
Disease	C0004352	Autistic Disorder	2.44E-07	9.52E-04
Disease	C0270862	Hemiplegic migraine	9.58E-06	2.49E-02
Disease	C0014553	Absence Epilepsy	1.50E-05	2.65E-02
Disease	C4505072	Epileptic Syndromes	1.98E-05	2.65E-02
Disease	C0023893	Liver Cirrhosis, Experimental	2.04E-05	2.65E-02
Disease	C1720189	Episodic Ataxia	2.74E-05	3.05E-02
Disease	C0751057	Seizure, Febrile, Complex	4.20E-05	3.64E-02
Disease	C0149886	Seizure, Febrile, Simple	4.20E-05	3.64E-02

Abbreviations: rsFC, resting-state functional connectivity; A1/2/3ulhf, upper limb, head and face region of area 1/2/3; GO, gene ontology.

Enrichment results of the genes related to rsFC of the A1/2/3tru

Category	ID	Name	*P* value	*q* value (FDR-BH correction)
GO: Molecular Function	GO:0015267	channel activity	7.78E-07	2.71E-04
GO: Molecular Function	GO:0022803	passive transmembrane transporter activity	8.03E-07	2.71E-04
GO: Molecular Function	GO:0005216	ion channel activity	8.61E-07	2.71E-04
GO: Molecular Function	GO:0005261	cation channel activity	4.92E-06	8.12E-04
GO: Molecular Function	GO:0016247	channel regulator activity	5.12E-06	8.12E-04
GO: Molecular Function	GO:0046873	metal ion transmembrane transporter activity	5.16E-06	8.12E-04
GO: Molecular Function	GO:0022839	ion gated channel activity	8.30E-06	1.12E-03
GO: Molecular Function	GO:0008092	cytoskeletal protein binding	1.11E-05	1.31E-03
GO: Molecular Function	GO:0022836	gated channel activity	1.29E-05	1.36E-03
GO: Molecular Function	GO:0022857	transmembrane transporter activity	2.56E-05	2.23E-03
GO: Molecular Function	GO:0005215	transporter activity	2.61E-05	2.23E-03
GO: Molecular Function	GO:0099095	ligand-gated anion channel activity	3.14E-05	2.46E-03
GO: Molecular Function	GO:0015318	inorganic molecular entity transmembrane transporter activity	4.50E-05	3.27E-03
GO: Molecular Function	GO:0042165	neurotransmitter binding	5.79E-05	3.90E-03
GO: Molecular Function	GO:0022890	inorganic cation transmembrane transporter activity	8.47E-05	5.32E-03
GO: Molecular Function	GO:0016594	glycine binding	1.27E-04	7.46E-03
GO: Molecular Function	GO:0015075	ion transmembrane transporter activity	1.43E-04	7.70E-03
GO: Molecular Function	GO:0005516	calmodulin binding	1.54E-04	7.70E-03
GO: Molecular Function	GO:0005244	voltage-gated ion channel activity	1.57E-04	7.70E-03
GO: Molecular Function	GO:0022832	voltage-gated channel activity	1.63E-04	7.70E-03
GO: Molecular Function	GO:0022843	voltage-gated cation channel activity	1.73E-04	7.76E-03
GO: Molecular Function	GO:0050811	GABA receptor binding	2.14E-04	9.19E-03
GO: Molecular Function	GO:0008324	cation transmembrane transporter activity	2.44E-04	1.00E-02
GO: Molecular Function	GO:0030594	neurotransmitter receptor activity	2.79E-04	1.10E-02
GO: Molecular Function	GO:0022852	glycine-gated chloride ion channel activity	3.38E-04	1.27E-02
GO: Molecular Function	GO:0016597	amino acid binding	4.16E-04	1.51E-02
GO: Molecular Function	GO:0015085	calcium ion transmembrane transporter activity	5.08E-04	1.72E-02
GO: Molecular Function	GO:0099106	ion channel regulator activity	5.10E-04	1.72E-02
GO: Molecular Function	GO:0017080	sodium channel regulator activity	5.29E-04	1.72E-02
GO: Molecular Function	GO:0004983	neuropeptide Y receptor activity	6.56E-04	2.06E-02
GO: Molecular Function	GO:0017022	myosin binding	6.91E-04	2.10E-02
GO: Molecular Function	GO:0005230	extracellular ligand-gated ion channel activity	7.55E-04	2.18E-02
GO: Molecular Function	GO:0004683	calmodulin-dependent protein kinase activity	7.63E-04	2.18E-02
GO: Molecular Function	GO:0031406	carboxylic acid binding	1.11E-03	3.08E-02
GO: Molecular Function	GO:0098631	cell adhesion mediator activity	1.18E-03	3.17E-02
GO: Molecular Function	GO:0022835	transmitter-gated channel activity	1.45E-03	3.70E-02
GO: Molecular Function	GO:0022824	transmitter-gated ion channel activity	1.45E-03	3.70E-02
GO: Molecular Function	GO:0022851	GABA-gated chloride ion channel activity	1.50E-03	3.73E-02
GO: Molecular Function	GO:0043177	organic acid binding	1.63E-03	3.93E-02
GO: Molecular Function	GO:0005267	potassium channel activity	1.77E-03	4.18E-02
GO: Molecular Function	GO:0005251	delayed rectifier potassium channel activity	1.94E-03	4.26E-02
GO: Molecular Function	GO:0001601	peptide YY receptor activity	1.94E-03	4.26E-02
GO: Molecular Function	GO:0001602	pancreatic polypeptide receptor activity	1.94E-03	4.26E-02
GO: Molecular Function	GO:0005509	calcium ion binding	2.15E-03	4.61E-02
GO: Molecular Function	GO:0032036	myosin heavy chain binding	2.32E-03	4.87E-02
GO: Biological Process	GO:0099537	trans-synaptic signaling	2.31E-08	4.83E-05
GO: Biological Process	GO:0099536	synaptic signaling	3.43E-08	4.83E-05
GO: Biological Process	GO:0007267	cell-cell signaling	4.75E-08	4.83E-05
GO: Biological Process	GO:0098916	anterograde trans-synaptic signaling	5.16E-08	4.83E-05
GO: Biological Process	GO:0007268	chemical synaptic transmission	5.16E-08	4.83E-05
GO: Biological Process	GO:0006811	ion transport	3.16E-07	2.47E-04
GO: Biological Process	GO:0030001	metal ion transport	1.76E-06	1.12E-03
GO: Biological Process	GO:0006812	cation transport	1.92E-06	1.12E-03
GO: Biological Process	GO:0023061	signal release	2.45E-06	1.23E-03
GO: Biological Process	GO:0043269	regulation of ion transport	2.62E-06	1.23E-03
GO: Biological Process	GO:0042391	regulation of membrane potential	1.58E-05	6.74E-03
GO: Biological Process	GO:0098660	inorganic ion transmembrane transport	1.85E-05	7.21E-03
GO: Biological Process	GO:0070838	divalent metal ion transport	3.10E-05	1.12E-02
GO: Biological Process	GO:0072511	divalent inorganic cation transport	3.81E-05	1.27E-02
GO: Biological Process	GO:0055085	transmembrane transport	5.28E-05	1.59E-02
GO: Biological Process	GO:0009914	hormone transport	5.43E-05	1.59E-02
GO: Biological Process	GO:0006816	calcium ion transport	6.23E-05	1.72E-02
GO: Biological Process	GO:0034762	regulation of transmembrane transport	6.94E-05	1.81E-02
GO: Biological Process	GO:0140352	export from cell	8.65E-05	2.11E-02
GO: Biological Process	GO:0034220	ion transmembrane transport	9.04E-05	2.11E-02
GO: Biological Process	GO:0071248	cellular response to metal ion	9.46E-05	2.11E-02
GO: Biological Process	GO:0046879	hormone secretion	1.33E-04	2.84E-02
GO: Biological Process	GO:0098662	inorganic cation transmembrane transport	1.57E-04	3.07E-02
GO: Biological Process	GO:0032940	secretion by cell	1.57E-04	3.07E-02
GO: Biological Process	GO:0060078	regulation of postsynaptic membrane potential	1.72E-04	3.22E-02
GO: Biological Process	GO:0046883	regulation of hormone secretion	1.93E-04	3.34E-02
GO: Biological Process	GO:0099565	chemical synaptic transmission, postsynaptic	2.04E-04	3.34E-02
GO: Biological Process	GO:0044057	regulation of system process	2.14E-04	3.34E-02
GO: Biological Process	GO:0010817	regulation of hormone levels	2.18E-04	3.34E-02
GO: Biological Process	GO:0048699	generation of neurons	2.20E-04	3.34E-02
GO: Biological Process	GO:0098655	cation transmembrane transport	2.24E-04	3.34E-02
GO: Biological Process	GO:0050804	modulation of chemical synaptic transmission	2.30E-04	3.34E-02
GO: Biological Process	GO:0099177	regulation of trans-synaptic signaling	2.35E-04	3.34E-02
GO: Biological Process	GO:0010038	response to metal ion	3.28E-04	4.52E-02
GO: Biological Process	GO:1903035	negative regulation of response to wounding	3.52E-04	4.70E-02
GO: Biological Process	GO:0046903	secretion	3.63E-04	4.72E-02
GO: Biological Process	GO:0071241	cellular response to inorganic substance	3.93E-04	4.98E-02
GO: Cellular Component	GO:0043005	neuron projection	6.69E-21	3.72E-18
GO: Cellular Component	GO:0045202	synapse	2.25E-15	6.26E-13
GO: Cellular Component	GO:0030424	axon	6.10E-15	1.13E-12
GO: Cellular Component	GO:0036477	somatodendritic compartment	1.00E-10	1.39E-08
GO: Cellular Component	GO:0030425	dendrite	2.27E-10	2.10E-08
GO: Cellular Component	GO:0097447	dendritic tree	2.27E-10	2.10E-08
GO: Cellular Component	GO:0097060	synaptic membrane	2.04E-09	1.62E-07
GO: Cellular Component	GO:0098793	presynapse	1.83E-08	1.27E-06
GO: Cellular Component	GO:0098978	glutamatergic synapse	2.26E-08	1.39E-06
GO: Cellular Component	GO:0098794	postsynapse	3.97E-08	2.21E-06
GO: Cellular Component	GO:0043025	neuronal cell body	5.73E-08	2.90E-06
GO: Cellular Component	GO:0044297	cell body	1.02E-07	4.73E-06
GO: Cellular Component	GO:0005887	integral component of plasma membrane	4.87E-07	2.08E-05
GO: Cellular Component	GO:0030054	cell junction	6.58E-07	2.62E-05
GO: Cellular Component	GO:0031226	intrinsic component of plasma membrane	9.78E-07	3.62E-05
GO: Cellular Component	GO:0150034	distal axon	2.00E-06	6.94E-05
GO: Cellular Component	GO:0034702	ion channel complex	2.39E-06	7.82E-05
GO: Cellular Component	GO:0043204	perikaryon	3.75E-06	1.16E-04
GO: Cellular Component	GO:1902495	transmembrane transporter complex	6.97E-06	2.04E-04
GO: Cellular Component	GO:0045211	postsynaptic membrane	8.37E-06	2.27E-04
GO: Cellular Component	GO:0044305	calyx of Held	8.57E-06	2.27E-04
GO: Cellular Component	GO:0043679	axon terminus	9.64E-06	2.35E-04
GO: Cellular Component	GO:1990351	transporter complex	9.72E-06	2.35E-04
GO: Cellular Component	GO:0099240	intrinsic component of synaptic membrane	2.39E-05	5.53E-04
GO: Cellular Component	GO:0044306	neuron projection terminus	2.75E-05	6.11E-04
GO: Cellular Component	GO:0099699	integral component of synaptic membrane	4.09E-05	8.74E-04
GO: Cellular Component	GO:0098982	GABA-ergic synapse	9.21E-05	1.90E-03
GO: Cellular Component	GO:0034703	cation channel complex	2.08E-04	4.14E-03
GO: Cellular Component	GO:0098797	plasma membrane protein complex	2.80E-04	5.25E-03
GO: Cellular Component	GO:0098936	intrinsic component of postsynaptic membrane	2.89E-04	5.25E-03
GO: Cellular Component	GO:0034681	integrin alpha11-beta1 complex	3.02E-04	5.25E-03
GO: Cellular Component	GO:0034677	integrin alpha7-beta1 complex	3.02E-04	5.25E-03
GO: Cellular Component	GO:0099080	supramolecular complex	3.15E-04	5.31E-03
GO: Cellular Component	GO:0043197	dendritic spine	3.40E-04	5.55E-03
GO: Cellular Component	GO:0098984	neuron to neuron synapse	3.51E-04	5.57E-03
GO: Cellular Component	GO:0044309	neuron spine	4.12E-04	6.36E-03

GO: Cellular Component	GO:0015629	actin cytoskeleton	4.39E-04	6.54E-03
GO: Cellular Component	GO:0099572	postsynaptic specialization	4.47E-04	6.54E-03
GO: Cellular Component	GO:0099512	supramolecular fiber	5.23E-04	7.26E-03
GO: Cellular Component	GO:0042470	melanosome	5.35E-04	7.26E-03
GO: Cellular Component	GO:0048770	pigment granule	5.35E-04	7.26E-03
GO: Cellular Component	GO:0005938	cell cortex	5.59E-04	7.40E-03
GO: Cellular Component	GO:0099081	supramolecular polymer	5.75E-04	7.43E-03
GO: Cellular Component	GO:0043235	receptor complex	7.23E-04	9.14E-03
GO: Cellular Component	GO:0099055	integral component of postsynaptic membrane	7.61E-04	9.40E-03
GO: Cellular Component	GO:0008076	voltage-gated potassium channel complex	8.78E-04	1.06E-02
GO: Cellular Component	GO:0099568	cytoplasmic region	9.04E-04	1.07E-02
GO: Cellular Component	GO:0030018	Z disc	9.47E-04	1.09E-02
GO: Cellular Component	GO:0014704	intercalated disc	9.60E-04	1.09E-02
GO: Cellular Component	GO:0098563	intrinsic component of synaptic vesicle membrane	1.04E-03	1.16E-02
GO: Cellular Component	GO:0014069	postsynaptic density	1.25E-03	1.34E-02
GO: Cellular Component	GO:0032279	asymmetric synapse	1.25E-03	1.34E-02
GO: Cellular Component	GO:0042734	presynaptic membrane	1.28E-03	1.34E-02
GO: Cellular Component	GO:0030285	integral component of synaptic vesicle membrane	1.38E-03	1.42E-02
GO: Cellular Component	GO:0098858	actin-based cell projection	1.61E-03	1.59E-02
GO: Cellular Component	GO:0034705	potassium channel complex	1.64E-03	1.59E-02
GO: Cellular Component	GO:0031674	I band	1.65E-03	1.59E-02
GO: Cellular Component	GO:0034707	chloride channel complex	1.66E-03	1.59E-02
GO: Cellular Component	GO:0005884	actin filament	1.76E-03	1.66E-02
GO: Cellular Component	GO:0070382	exocytic vesicle	1.95E-03	1.81E-02
GO: Cellular Component	GO:0009986	cell surface	2.09E-03	1.90E-02
GO: Cellular Component	GO:0031253	cell projection membrane	2.21E-03	1.98E-02
GO: Cellular Component	GO:0097457	hippocampal mossy fiber	2.35E-03	2.07E-02
GO: Cellular Component	GO:0044302	dentate gyrus mossy fiber	2.55E-03	2.21E-02
GO: Cellular Component	GO:0008021	synaptic vesicle	3.34E-03	2.85E-02
GO: Cellular Component	GO:0042383	sarcolemma	3.44E-03	2.90E-02
GO: Cellular Component	GO:0098685	Schaffer collateral - CA1 synapse	3.64E-03	3.02E-02
GO: Cellular Component	GO:0045121	membrane raft	3.87E-03	3.14E-02
GO: Cellular Component	GO:0044291	cell-cell contact zone	3.95E-03	3.14E-02
GO: Cellular Component	GO:0098857	membrane microdomain	3.96E-03	3.14E-02
GO: Cellular Component	GO:1902711	GABA-A receptor complex	4.02E-03	3.14E-02
GO: Cellular Component	GO:0002141	stereocilia ankle link	4.25E-03	3.24E-02
GO: Cellular Component	GO:0002142	stereocilia ankle link complex	4.25E-03	3.24E-02
GO: Cellular Component	GO:0099522	region of cytosol	5.33E-03	3.91E-02
GO: Cellular Component	GO:0044295	axonal growth cone	5.33E-03	3.91E-02
GO: Cellular Component	GO:1902710	GABA receptor complex	5.37E-03	3.91E-02
GO: Cellular Component	GO:0098589	membrane region	5.42E-03	3.91E-02
GO: Cellular Component	GO:0002139	stereocilia coupling link	5.88E-03	4.09E-02
GO: Cellular Component	GO:0038037	G protein-coupled receptor dimeric complex	5.88E-03	4.09E-02
GO: Cellular Component	GO:0048179	activin receptor complex	5.88E-03	4.09E-02
GO: Cellular Component	GO:0030426	growth cone	6.06E-03	4.11E-02
GO: Cellular Component	GO:0031256	leading edge membrane	6.06E-03	4.11E-02
GO: Cellular Component	GO:0043194	axon initial segment	6.14E-03	4.11E-02
GO: Cellular Component	GO:0030017	sarcomere	6.24E-03	4.12E-02
GO: Cellular Component	GO:0031300	intrinsic component of organelle membrane	6.30E-03	4.12E-02
GO: Cellular Component	GO:0048786	presynaptic active zone	6.37E-03	4.12E-02
GO: Cellular Component	GO:0099501	exocytic vesicle membrane	6.83E-03	4.31E-02
GO: Cellular Component	GO:0030672	synaptic vesicle membrane	6.83E-03	4.31E-02
GO: Cellular Component	GO:0030427	site of polarized growth	7.02E-03	4.34E-02
GO: Cellular Component	GO:0031594	neuromuscular junction	7.03E-03	4.34E-02
GO: Cellular Component	GO:0032421	stereocilium bundle	7.19E-03	4.34E-02
GO: Cellular Component	GO:0032420	stereocilium	7.19E-03	4.34E-02
GO: Cellular Component	GO:0098889	intrinsic component of presynaptic membrane	7.41E-03	4.43E-02
GO: Cellular Component	GO:0032589	neuron projection membrane	7.62E-03	4.51E-02
GO: Cellular Component	GO:0098796	membrane protein complex	7.99E-03	4.61E-02
GO: Cellular Component	GO:0043195	terminal bouton	8.03E-03	4.61E-02
GO: Cellular Component	GO:0098862	cluster of actin-based cell projections	8.10E-03	4.61E-02
GO: Cellular Component	GO:0098805	whole membrane	8.12E-03	4.61E-02
GO: Cellular Component	GO:0030863	cortical cytoskeleton	8.36E-03	4.69E-02
GO: Cellular Component	GO:0048787	presynaptic active zone membrane	8.66E-03	4.81E-02
GO: Cellular Component	GO:0099523	presynaptic cytosol	8.83E-03	4.82E-02
GO: Cellular Component	GO:0005912	adherens junction	8.85E-03	4.82E-02
GO: Cellular Component	GO:0048471	perinuclear region of cytoplasm	8.97E-03	4.83E-02
GO: Cellular Component	GO:0031902	late endosome membrane	9.04E-03	4.83E-02
Pathway	1268763	Neuronal System	1.17E-09	1.75E-06
Pathway	1268795	Trafficking of AMPA receptors	1.39E-06	6.93E-04
Pathway	1268794	Glutamate Binding, Activation of AMPA Receptors and Synaptic Plasticity	1.39E-06	6.93E-04
Pathway	1268766	Transmission across Chemical Synapses	1.08E-05	3.93E-03
Pathway	1268830	Voltage gated Potassium channels	1.31E-05	3.93E-03
Pathway	1269145	Neurotoxicity of clostridium toxins	2.07E-05	5.16E-03
Pathway	M113	Role of Calcineurin-dependent NFAT signaling in lymphocytes	4.60E-05	9.83E-03
Pathway	M2890	Calcium signaling pathway	9.62E-05	1.80E-02
Pathway	83050	Calcium signaling pathway	1.25E-04	2.08E-02
Pathway	1339117	Phase 0 - rapid depolarisation	1.97E-04	2.95E-02
Pathway	137953	Role of Calcineurin-dependent NFAT signaling in lymphocytes	3.11E-04	4.11E-02
Pathway	PW:0000296	hypertrophic cardimyopathy	3.37E-04	4.11E-02
Pathway	1268786	Neurotransmitter Receptor Binding And Downstream Transmission In The Postsynaptic Cell	3.57E-04	4.11E-02
Pathway	P04391	Oxytocin receptor mediated signaling pathway	4.72E-04	4.73E-02
Pathway	1268821	Potassium Channels	4.75E-04	4.73E-02

Abbreviations: rsFC, resting-state functional connectivity; A1/2/3tru, trunk region of area 1/2/3; GO, gene ontology.
