# Supplementary material for: Transcriptional substrates underlying functional connectivity profiles of subregions within the human sensorimotor cortex
Source: Hum Brain Mapp. 2022 Jul 27;43(18):5562–78. doi: 10.1002/hbm.26031 (PMC9704778; doi:10.1002/hbm.26031)
Supplement: Supplementary file 1 — Appendix S1 Supplementary Information [file HBM-43-5562-s001.zip › HBM_26031_Supplementary file 5.pdf]

The genes related to rsFC of sensorimotor subregions at the DS cutoff threshold of 40%											
A4hf		A6cdl		A4ul		A4ll		A1/2/3ulhf		A1/2/3tru	
GeneID	Genesymbol	GeneID	Genesymbol	GeneID	Genesymbol	GeneID	Genesymbol	GeneID	Genesymbol	GeneID	Genesymbol
92	ACVR2A	92	ACVR2A	92	ACVR2A	176	ACAN	92	ACVR2A	92	ACVR2A
133	ADM	176	ACAN	98	ACYP2	341	APOC1	98	ACYP2	98	ACYP2
176	ACAN	326	AIRE	115	ADCY9	784	CACNB3	115	ADCY9	161	AP2A2
204	AK2	586	BCAT1	133	ADM	793	CALB1	133	ADM	176	ACAN
286	ANK1	784	CACNB3	161	AP2A2	814	CAMK4	161	AP2A2	204	AK2
308	ANXA5	793	CALB1	176	ACAN	817	CAMK2D	176	ACAN	249	ALPL
341	APOC1	817	CAMK2D	204	AK2	2044	EPHA5	204	AK2	286	ANK1
367	AR	1893	ECM1	220	ALDH1A3	2830	GPR6	249	ALPL	308	ANXA5
430	ASCL2	2044	EPHA5	249	ALPL	3679	ITGA7	286	ANK1	341	APOC1
445	ASS1	2322	FLT3	257	ALX3	4638	MYLK	292	SLC25A5	367	AR
460	ASTN1	2823	GPM6A	286	ANK1	4703	NEB	308	ANXA5	430	ASCL2
481	ATP1B1	2830	GPR6	292	SLC25A5	5176	SERPINF1	366	AQP9	445	ASS1
483	ATP1B3	2952	GSTT1	308	ANXA5	5794	PTPRH	367	AR	460	ASTN1
493	ATP2B4	3290	HSD11B1	341	APOC1	5961	PRPH2	396	ARHGDIA	493	ATP2B4
563	AZGP1	3736	KCNA1	367	AR	6330	SCN4B	430	ASCL2	586	BCAT1
586	BCAT1	3755	KCNG1	403	ARL3	7781	SLC30A3	445	ASS1	784	CACNB3
613	BCR	3787	KCNS1	430	ASCL2	9256	TSPOAP1	460	ASTN1	793	CALB1
784	CACNB3	4082	MARCKS	445	ASS1	9473	THEMIS2	481	ATP1B1	814	CAMK4
793	CALB1	4599	MX1	460	ASTN1	10268	RAMP3	492	ATP2B3	817	CAMK2D
814	CAMK4	4741	NEFM	481	ATP1B1	22987	SV2C	493	ATP2B4	818	CAMK2G
817	CAMK2D	4744	NEFH	483	ATP1B3	50486	G0S2	528	ATP6V1C1	1002	CDH4
818	CAMK2G	5121	PCP4	492	ATP2B3	55244	SLC47A1	563	AZGP1	1006	CDH8
869	CBLN1	5409	PNMT	493	ATP2B4	55315	SLC29A3	586	BCAT1	1027	CDKN1B
1002	CDH4	6330	SCN4B	563	AZGP1	57526	PCDH19	613	BCR	1073	CFL2
1006	CDH8	6482	ST3GAL1	586	BCAT1	79660	PPP1R3B	715	C1R	1272	CNTN1
1050	CEBPA	8557	TCAP	613	BCR	80307	FER1L4	784	CACNB3	1294	COL7A1
1272	CNTN1	9127	P2RX6	631	BFSP1	84034	EMILIN2	793	CALB1	1300	COL10A1
1300	COL10A1	9256	TSPOAP1	715	C1R	84937	ZNRF1	808	CALM3	1501	CTNND2
1381	CRABP1	9473	THEMIS2	716	C1S	114571	SLC22A9	814	CAMK4	1522	CTSZ
1501	CTNND2	9651	PLCH2	784	CACNB3	114990	VASN	817	CAMK2D	1607	DGKB
1522	CTSZ	10160	FARP1	793	CALB1	127833	SYT2	818	CAMK2G	1755	DMBT1
1607	DGKB	10231	RCAN2	808	CALM3	134548	SOWAHA	862	RUNX1T1	1893	ECM1
1730	DIAPH2	10268	RAMP3	814	CAMK4	148014	TTC9B	869	CBLN1	2027	ENO3
1809	DPYSL3	10683	DLL3	817	CAMK2D	163782	KANK4	1002	CDH4	2044	EPHA5
1893	ECM1	22987	SV2C	818	CAMK2G	221294	NT5DC1	1005	CDH7	2070	EYA4
1948	EFNB2	22996	TTC39A	831	CAST	222537	HS3ST5	1006	CDH8	2101	ESRRA
2027	ENO3	23406	COTL1	1002	CDH4	254102	EHBP1L1	1007	CDH9	2104	ESRRG
2044	EPHA5	25953	PNKD	1006	CDH8	286133	SCARA5	1027	CDKN1B	2161	F12
2070	EYA4	26010	SPATS2L	1007	CDH9			1031	CDKN2C	2322	FLT3
2101	ESRRA	27294	DHDH	1027	CDKN1B			1032	CDKN2D	2560	GABRB1
2104	ESRRG	29799	YPEL1	1031	CDKN2C			1040	CDS1	2562	GABRB3
2155	F7	50486	G0S2	1032	CDKN2D			1050	CEBPA	2565	GABRG1
2161	F12	50853	VILL	1040	CDS1			1073	CFL2	2620	GAS2
2173	FABP7	51059	FAM135B	1050	CEBPA			1176	AP3S1	2634	GBP2
2254	FGF9	54492	NEURL1B	1073	CFL2			1272	CNTN1	2742	GLRA2
2268	FGR	54551	MAGEL2	1176	AP3S1			1294	COL7A1	2766	GMPR
2289	FKBP5	54793	KCTD9	1272	CNTN1			1300	COL10A1	2823	GPM6A
2322	FLT3	55040	EPN3	1294	COL7A1			1381	CRABP1	2830	GPR6
2560	GABRB1	55244	SLC47A1	1300	COL10A1			1501	CTNND2	2911	GRM1
2565	GABRG1	55315	SLC29A3	1381	CRABP1			1522	CTSZ	2952	GSTT1
2620	GAS2	55686	MREG	1397	CRIP2			1523	CUX1	3067	HDC
2632	GBE1	55714	TENM3	1501	CTNND2			1607	DGKB	3290	HSD11B1
2634	GBP2	56967	C14orf132	1522	CTSZ			1730	DIAPH2	3316	HSPB2
2742	GLRA2	79660	PPP1R3B	1607	DGKB			1755	DMBT1	3350	HTR1A
2766	GMPR	79767	ELMO3	1675	CFD			1768	DNAH6	3624	INHBA
2778	GNAS	79930	DOK3	1730	DIAPH2			1809	DPYSL3	3632	INPP5A
2823	GPM6A	80020	FOXRED2	1755	DMBT1			1871	E2F3	3679	ITGA7
2830	GPR6	80307	FER1L4	1809	DPYSL3			1875	E2F5	3688	ITGB1
2888	GRB14	83547	RILP	1893	ECM1			1893	ECM1	3736	KCNA1
2898	GRIK2	84034	EMILIN2	1949	EFNB3			1909	EDNRA	3738	KCNA3
2911	GRM1	90523	MLIP	2027	ENO3			1948	EFNB2	3741	KCNA5
2918	GRM8	91624	NEXN	2044	EPHA5			1949	EFNB3	3755	KCNG1
2952	GSTT1	113675	SDSL	2070	EYA4			1960	EGR3	3783	KCNN4
3067	HDC	114787	GPRIN1	2101	ESRRA			2027	ENO3	3910	LAMA4
3231	HOXD1	114990	VASN	2104	ESRRG			2044	EPHA5	4062	LY6H
3269	HRH1	119587	CPXM2	2161	F12			2070	EYA4	4082	MARCKS
3290	HSD11B1	127833	SYT2	2170	FABP3			2101	ESRRA	4258	MGST2
3316	HSPB2	134548	SOWAHA	2173	FABP7			2104	ESRRG	4286	MITF
3350	HTR1A	147463	ANKRD29	2182	ACSL4			2161	F12	4324	MMP15
3396	MRPL58	160760	PPTC7	2254	FGF9			2170	FABP3	4345	CD200
3485	IGFBP2	163782	KANK4	2289	FKBP5			2173	FABP7	4354	MPP1
3595	IL12RB2	192668	CYS1	2322	FLT3			2182	ACSL4	4599	MX1
3624	INHBA	222537	HS3ST5	2558	GABRA5			2254	FGF9	4609	MYC
3632	INPP5A	286133	SCARA5	2560	GABRB1			2322	FLT3	4638	MYLK
3653	IPW	326624	RAB37	2562	GABRB3			2558	GABRA5	4703	NEB
3671	ISLR	342667	STAC2	2565	GABRG1			2560	GABRB1	4744	NEFH
3679	ITGA7	415116	PIM3	2620	GAS2			2562	GABRB3	4751	NEK2
3688	ITGB1	642273	FAM110C	2632	GBE1			2565	GABRG1	4753	NELL2
3736	KCNA1	654502	IQCJ	2634	GBP2			2620	GAS2	4856	NOV
3738	KCNA3			2690	GHR			2634	GBP2	4886	NPY1R
3741	KCNA5			2742	GLRA2			2690	GHR	4889	NPY5R
3755	KCNG1			2764	GMFB			2742	GLRA2	5091	PC
3782	KCNN3			2766	GMPR			2764	GMFB	5116	PCNT
3783	KCNN4			2778	GNAS			2766	GMPR	5121	PCP4
3827	KNG1			2786	GNG4			2778	GNAS	5138	PDE2A
3902	LAG3			2790	GNG10			2823	GPM6A	5141	PDE4A
3910	LAMA4			2820	GPD2			2830	GPR6	5176	SERPINF1
4062	LY6H			2823	GPM6A			2888	GRB14	5409	PNMT
4082	MARCKS			2830	GPR6			2890	GRIA1	5412	UBL3
4092	SMAD7			2888	GRB14			2898	GRIK2	5547	PRCP
4157	MC1R			2890	GRIA1			2911	GRM1	5569	PKIA
4257	MGST1			2898	GRIK2			2918	GRM8	5579	PRKCB
4258	MGST2			2911	GRM1			2947	GSTM3	5580	PRKCD
4286	MITF			2918	GRM8			2952	GSTT1	5582	PRKCG
4324	MMP15			2947	GSTM3			3067	HDC	5733	PTGER3
4354	MPP1			2952	GSTT1			3145	HMBS	5774	PTPN3
4599	MX1			3067	HDC			3156	HMGCR	5794	PTPRH
4609	MYC			3145	HMBS			3231	HOXD1	5874	RAB27B
4616	GADD45B			3156	HMGCR			3269	HRH1	5954	RCN1
4638	MYLK			3231	HOXD1			3290	HSD11B1	5961	PRPH2
4703	NEB			3269	HRH1			3316	HSPB2	5979	RET
4715	NDUFB9			3290	HSD11B1			3338	DNAJC4	5993	RFX5

4744	NEFH	3316	HSPB2	3350	HTR1A	6017	RLBP1
4751	NEK2	3338	DNAJC4	3358	HTR2C	6272	SORT1
4856	NOV	3350	HTR1A	3396	MRPL58	6330	SCN4B
4885	NPTX2	3358	HTR2C	3485	IGFBP2	6543	SLC8A2
4886	NPY1R	3396	MRPL58	3595	IL12RB2	6646	SOAT1
4889	NPY5R	3423	IDS	3598	IL13RA2	6804	STX1A
5090	PBX3	3598	IL13RA2	3624	INHBA	6843	VAMP1
5116	PCNT	3624	INHBA	3632	INPP5A	6890	TAP1
5121	PCP4	3632	INPP5A	3653	IPW	6905	TBCE
5141	PDE4A	3653	IPW	3671	ISLR	6920	TCEA3
5176	SERPINF1	3679	ITGA7	3679	ITGA7	7062	TCHH
5184	PEPD	3688	ITGB1	3688	ITGB1	7068	THRB
5272	SERPINB9	3706	ITPKA	3736	KCNA1	7089	TLE2
5292	PIM1	3736	KCNA1	3738	KCNA3	7092	TLL1
5325	PLAGL1	3738	KCNA3	3741	KCNA5	7103	TSPAN8
5332	PLCB4	3739	KCNA4	3746	KCNC1	7138	TNNT1
5409	PNMT	3741	KCNA5	3755	KCNG1	7145	TNS1
5412	UBL3	3746	KCNC1	3782	KCNN3	7378	UPP1
5467	PPARD	3755	KCNG1	3783	KCNN4	7480	WNT10B
5547	PRCP	3783	KCNN4	3787	KCNS1	7534	YWHAZ
5569	PKIA	3787	KCNS1	3827	KNG1	7781	SLC30A3
5580	PRKCD	3827	KNG1	3902	LAG3	8001	GLRA3
5582	PRKCG	3902	LAG3	3910	LAMA4	8209	C21orf33
5606	MAP2K3	3910	LAMA4	4062	LY6H	8482	SEMA7A
5774	PTPN3	4062	LY6H	4082	MARCKS	8611	PLPP1
5792	PTPRF	4082	MARCKS	4088	SMAD3	8704	B4GALT2
5794	PTPRH	4092	SMAD7	4092	SMAD7	8715	NOL4
5816	PVALB	4157	MC1R	4157	MC1R	8717	TRADD
5874	RAB27B	4168	MCF2	4168	MCF2	8792	TNFRSF11A
5912	RAP2B	4257	MGST1	4257	MGST1	8884	SLC5A6
5937	RBMS1	4258	MGST2	4258	MGST2	8938	BAIAP3
5950	RBP4	4286	MITF	4286	MITF	9033	PKD2L1
5954	RCN1	4324	MMP15	4324	MMP15	9127	P2RX6
5979	RET	4337	MOCS1	4345	CD200	9196	KCNAB3
5993	RFX5	4345	CD200	4354	MPP1	9253	NUMBL
6017	RLBP1	4354	MPP1	4599	MX1	9254	CACNA2D2
6122	RPL3	4599	MX1	4602	MYB	9256	TSPOAP1
6272	SORT1	4602	MYB	4609	MYC	9312	KCNB2
6323	SCN1A	4609	MYC	4616	GADD45B	9362	CPNE6
6324	SCN1B	4616	GADD45B	4625	MYH7	9454	HOMER3
6330	SCN4B	4625	MYH7	4638	MYLK	9473	THEMIS2
6451	SH3BGRL	4636	MYL5	4703	NEB	9495	AKAP5
6533	SLC6A6	4638	MYLK	4715	NDUFB9	9607	CARTPT
6543	SLC8A2	4703	NEB	4744	NEFH	9609	RAB36
6646	SOAT1	4715	NDUFB9	4751	NEK2	9636	ISG15
6804	STX1A	4744	NEFH	4753	NELL2	9651	PLCH2
6843	VAMP1	4751	NEK2	4782	NFIC	9911	TMCC2
6854	SYN2	4753	NELL2	4824	NKX3-1	10083	USH1C
6890	TAP1	4782	NFIC	4856	NOV	10154	PLXNC1
6905	TBCE	4824	NKX3-1	4885	NPTX2	10160	FARP1
6920	TCEA3	4826	NNAT	4886	NPY1R	10231	RCAN2
7062	TCHH	4856	NOV	4889	NPY5R	10268	RAMP3
7068	THRB	4878	NPPA	4968	OGG1	10332	CLEC4M
7089	TLE2	4885	NPTX2	4988	OPRM1	10368	CACNG3
7092	TLL1	4886	NPY1R	5064	PALM	10395	DLC1
7103	TSPAN8	4889	NPY5R	5090	PBX3	10451	VAV3
7138	TNNT1	4968	OGG1	5091	PC	10505	SEMA4F
7145	TNS1	4988	OPRM1	5116	PCNT	10669	CGREF1
7222	TRPC3	5064	PALM	5121	PCP4	10673	TNFSF13B
7301	TYRO3	5090	PBX3	5138	PDE2A	10683	DLL3
7378	UPP1	5091	PC	5141	PDE4A	10776	ARPP19
7402	UTRN	5111	PCNA	5176	SERPINF1	10783	NEK6
7409	VAV1	5116	PCNT	5184	PEPD	10891	PPARGC1A
7480	WNT10B	5119	CHMP1A	5272	SERPINB9	11069	RAPGEF4
7781	SLC30A3	5121	PCP4	5292	PIM1	11138	TBC1D8
8001	GLRA3	5138	PDE2A	5310	PKD1	11151	CORO1A
8174	MADCAM1	5141	PDE4A	5325	PLAGL1	11164	NUDT5
8209	C21orf33	5176	SERPINF1	5332	PLCB4	11259	FILIP1L
8321	FZD1	5184	PEPD	5361	PLXNA1	22801	ITGA11
8437	RASAL1	5256	PHKA2	5409	PNMT	22881	ANKRD6
8445	DYRK2	5272	SERPINB9	5412	UBL3	22987	SV2C
8482	SEMA7A	5292	PIM1	5467	PPARD	22996	TTC39A
8604	SLC25A12	5310	PKD1	5475	PPEF1	23046	KIF21B
8611	PLPP1	5330	PLCB2	5547	PRCP	23109	DDN
8704	B4GALT2	5332	PLCB4	5569	PKIA	23180	RFTN1
8715	NOL4	5409	PNMT	5579	PRKCB	23274	CLEC16A
8717	TRADD	5412	UBL3	5580	PRKCD	23406	COTL1
8792	TNFRSF11A	5453	POU3F1	5582	PRKCG	23484	LEPROTL1
8884	SLC5A6	5467	PPARD	5594	MAPK1	23504	RIMBP2
8938	BAIAP3	5547	PRCP	5606	MAP2K3	23642	SNHG1
9020	MAP3K14	5557	PRIM1	5655	KLK10	25841	ABTB2
9033	PKD2L1	5569	PKIA	5733	PTGER3	25854	FAM149A
9120	SLC16A6	5570	PKIB	5774	PTPN3	25871	NEPRO
9127	P2RX6	5579	PRKCB	5786	PTPRA	25924	MYRIP
9168	TMSB10	5580	PRKCD	5792	PTPRF	25989	ULK3
9196	KCNAB3	5582	PRKCG	5794	PTPRH	26010	SPATS2L
9254	CACNA2D2	5594	MAPK1	5801	PTPRR	26059	ERC2
9256	TSPOAP1	5606	MAP2K3	5816	PVALB	26996	GPR160
9312	KCNB2	5733	PTGER3	5874	RAB27B	27077	B9D1
9315	NREP	5774	PTPN3	5912	RAP2B	27132	CPNE7
9362	CPNE6	5786	PTPRA	5937	RBMS1	27163	NAAA
9452	ITM2A	5792	PTPRF	5950	RBP4	27294	DHDH
9454	HOMER3	5794	PTPRH	5954	RCN1	28955	DEXI
9592	IER2	5801	PTPRR	5961	PRPH2	29799	YPEL1
9607	CARTPT	5806	PTX3	5979	RET	29803	REPIN1
9609	RAB36	5816	PVALB	5993	RFX5	30850	CDR2L
9636	ISG15	5874	RAB27B	6017	RLBP1	50486	G0S2
9750	RIPOR2	5912	RAP2B	6272	SORT1	50853	VILL
9828	ARHGEF17	5937	RBMS1	6323	SCN1A	51059	FAM135B
9882	TBC1D4	5950	RBP4	6324	SCN1B	51074	APIP
9911	TMCC2	5954	RCN1	6326	SCN2A	51134	CEP83
10083	USH1C	5961	PRPH2	6330	SCN4B	51312	SLC25A37
10154	PLXNC1	5979	RET	6451	SH3BGRL	51375	SNX7
10160	FARP1	5993	RFX5	6509	SLC1A4	51393	TRPV2
10171	RCL1	6017	RLBP1	6513	SLC2A1	51440	HPCAL4
10231	RCAN2	6118	RPA2	6533	SLC6A6	51454	GULP1
10268	RAMP3	6122	RPL3	6543	SLC8A2	51522	TMEM14C

10332	CLEC4M	6272	SORT1	6567	SLC16A2	51660	MPC1
10395	DLC1	6320	CLEC11A	6585	SLIT1	51667	NUB1
10451	VAV3	6323	SCN1A	6623	SNCG	54112	GPR88
10505	SEMA4F	6324	SCN1B	6646	SOAT1	54492	NEURL1B
10613	ERLIN1	6326	SCN2A	6751	SSTR1	54536	EXOC6
10673	TNFSF13B	6330	SCN4B	6804	STX1A	54550	NECAB2
10891	PPARGC1A	6389	SDHA	6843	VAMP1	54551	MAGEL2
11069	RAPGEF4	6451	SH3BGRL	6854	SYN2	54566	EPB41L4B
11138	TBC1D8	6509	SLC1A4	6890	TAP1	54793	KCTD9
11151	CORO1A	6533	SLC6A6	6905	TBCE	54843	SYTL2
11164	NUDT5	6541	SLC7A1	6920	TCEA3	54847	SIDT1
11211	FZD10	6543	SLC8A2	7062	TCHH	54972	TMEM132A
11259	FILIP1L	6545	SLC7A4	7068	THRB	55040	EPN3
11279	KLF8	6567	SLC16A2	7087	ICAM5	55160	ARHGEF10L
22801	ITGA11	6585	SLIT1	7089	TLE2	55244	SLC47A1
22881	ANKRD6	6603	SMARCD2	7092	TLL1	55315	SLC29A3
22987	SV2C	6646	SOAT1	7103	TSPAN8	55353	LAPTM4B
22996	TTC39A	6676	SPAG4	7138	TNNT1	55509	BATF3
23046	KIF21B	6751	SSTR1	7145	TNS1	55591	VEZT
23109	DDN	6770	STAR	7222	TRPC3	55686	MREG
23180	RFTN1	6804	STX1A	7301	TYRO3	55800	SCN3B
23245	ASTN2	6843	VAMP1	7378	UPP1	55853	IDI2-AS1
23274	CLEC16A	6854	SYN2	7402	UTRN	55897	MESP1
23305	ACSL6	6890	TAP1	7480	WNT10B	56934	CA10
23406	COTL1	6905	TBCE	7533	YWHAH	56937	PMEPA1
23460	ABCA6	6920	TCEA3	7534	YWHAZ	56967	C14orf132
23484	LEPROTL1	7007	TECTA	7726	TRIM26	57110	HRASLS
23504	RIMBP2	7062	TCHH	7781	SLC30A3	57406	ABHD6
23642	SNHG1	7068	THRB	7791	ZYX	57453	DSCAML1
25825	BACE2	7087	ICAM5	8001	GLRA3	57495	NWD2
25841	ABTB2	7089	TLE2	8013	NR4A3	57496	MKL2
25900	IFFO1	7092	TLL1	8099	CDK2AP1	57526	PCDH19
25907	TMEM158	7103	TSPAN8	8174	MADCAM1	57554	LRRC7
25946	ZNF385A	7138	TNNT1	8209	C21orf33	57718	PPP4R4
25953	PNKD	7145	TNS1	8321	FZD1	64132	XYLT2
25989	ULK3	7222	TRPC3	8404	SPARCL1	64135	IFIH1
26031	OSBPL3	7301	TYRO3	8437	RASAL1	64149	C17orf75
26232	FBXO2	7347	UCHL3	8445	DYRK2	64420	SUSD1
27077	B9D1	7378	UPP1	8482	SEMA7A	65078	RTN4R
27132	CPNE7	7402	UTRN	8604	SLC25A12	65997	RASL11B
27163	NAAA	7409	VAV1	8611	PLPP1	66000	TMEM108
28955	DEXI	7480	WNT10B	8704	B4GALT2	66008	TRAK2
29115	SAP30BP	7533	YWHAH	8706	B3GALNT1	79012	CAMKV
29799	YPEL1	7534	YWHAZ	8715	NOL4	79017	GGCT
29803	REPIN1	7726	TRIM26	8717	TRADD	79183	TTPAL
29844	TFPT	7781	SLC30A3	8792	TNFRSF11A	79645	EFCAB1
29997	NOP53	7791	ZYX	8805	TRIM24	79660	PPP1R3B
30850	CDR2L	7798	LUZP1	8877	SPHK1	79720	VPS37B
50486	G0S2	8001	GLRA3	8884	SLC5A6	79745	CLIP4
50853	VILL	8099	CDK2AP1	8938	BAIAP3	79750	ZNF385D
51059	FAM135B	8174	MADCAM1	9020	MAP3K14	79822	ARHGAP28
51074	APIP	8193	DPF1	9033	PKD2L1	79958	DENND1C
51105	PHF20L1	8209	C21orf33	9120	SLC16A6	79993	ELOVL7
51123	ZNF706	8321	FZD1	9127	P2RX6	80020	FOXRED2
51134	CEP83	8404	SPARCL1	9168	TMSB10	80119	PIF1
51312	SLC25A37	8425	LTBP4	9196	KCNAB3	80176	SPSB1
51319	RSRC1	8437	RASAL1	9215	LARGE1	80307	FER1L4
51334	PRR16	8445	DYRK2	9253	NUMBL	80323	CCDC68
51375	SNX7	8470	SORBS2	9254	CACNA2D2	80820	EEPD1
51440	HPCAL4	8482	SEMA7A	9256	TSPOAP1	80854	SETD7
51454	GULP1	8507	ENC1	9312	KCNB2	81033	KCNH6
51522	TMEM14C	8573	CASK	9315	NREP	81849	ST6GALNAC5
51642	MRPL48	8604	SLC25A12	9362	CPNE6	83445	GSG1
51660	MPC1	8611	PLPP1	9382	COG1	83468	GLT8D2
54112	GPR88	8704	B4GALT2	9452	ITM2A	83660	TLN2
54536	EXOC6	8715	NOL4	9454	HOMER3	83690	CRISPLD1
54550	NECAB2	8717	TRADD	9473	THEMIS2	83714	NRIP2
54551	MAGEL2	8792	TNFRSF11A	9495	AKAP5	83787	ARMC10
54566	EPB41L4B	8805	TRIM24	9508	ADAMTS3	83875	BCO2
54793	KCTD9	8853	ASAP2	9570	GOSR2	84034	EMILIN2
54843	SYTL2	8877	SPHK1	9592	IER2	84109	QRFPR
54847	SIDT1	8884	SLC5A6	9607	CARTPT	84187	TMEM164
54874	FNBP1L	8938	BAIAP3	9609	RAB36	84221	SPATC1L
55040	EPN3	9020	MAP3K14	9636	ISG15	84439	HHIPL1
55086	CXorf57	9033	PKD2L1	9651	PLCH2	84525	HOPX
55118	CRTAC1	9120	SLC16A6	9750	RIPOR2	84542	KIAA1841
55122	AKIRIN2	9127	P2RX6	9823	ARMCX2	84623	KIRREL3
55160	ARHGEF10L	9168	TMSB10	9828	ARHGEF17	84769	MPV17L2
55170	PRMT6	9196	KCNAB3	9882	TBC1D4	84812	PLCD4
55208	DCUN1D2	9253	NUMBL	9903	KLHL21	84936	ZFYVE19
55244	SLC47A1	9254	CACNA2D2	9911	TMCC2	84937	ZNRF1
55315	SLC29A3	9256	TSPOAP1	9957	HS3ST1	85301	COL27A1
55353	LAPTM4B	9267	CYTH1	10023	FRAT1	85352	SHISAL1
55509	BATF3	9312	KCNB2	10025	MED16	90102	PHLDB2
55591	VEZT	9315	NREP	10026	PIGK	90523	MLIP
55686	MREG	9362	CPNE6	10039	PARP3	90850	ZNF598
55800	SCN3B	9379	NRXN2	10040	TOM1L1	90861	JPT2
55853	IDI2-AS1	9382	COG1	10083	USH1C	91133	L3MBTL4
55897	MESP1	9452	ITM2A	10154	PLXNC1	91252	SLC39A13
56172	ANKH	9454	HOMER3	10160	FARP1	91624	NEXN
56648	EIF5A2	9473	THEMIS2	10171	RCL1	92335	STRADA
56934	CA10	9495	AKAP5	10174	SORBS3	113675	SDSL
56937	PMEPA1	9508	ADAMTS3	10231	RCAN2	114571	SLC22A9
56967	C14orf132	9537	TP53I11	10268	RAMP3	114787	GPRIN1
56971	CEACAM19	9570	GOSR2	10318	TNIP1	114800	CCDC85A
57194	ATP10A	9592	IER2	10332	CLEC4M	114990	VASN
57406	ABHD6	9607	CARTPT	10368	CACNG3	116028	RMI2
57453	DSCAML1	9609	RAB36	10395	DLC1	116135	LRRC3B
57465	TBC1D24	9630	GNA14	10402	ST3GAL6	118427	OLFM3
57495	NWD2	9636	ISG15	10425	ARIH2	119587	CPXM2
57496	MKL2	9651	PLCH2	10451	VAV3	127833	SYT2
57519	STARD9	9717	SEC14L5	10478	SLC25A17	128434	VSTM2L
57526	PCDH19	9750	RIPOR2	10505	SEMA4F	130399	ACVR1C
57596	BEGAIN	9790	BMS1	10555	AGPAT2	132160	PPM1M
57718	PPP4R4	9823	ARMCX2	10613	ERLIN1	132321	C4orf33
63974	NEUROD6	9882	TBC1D4	10669	CGREF1	133418	EMB
63982	ANO3	9903	KLHL21	10673	TNFSF13B	134548	SOWAHA

64131	XYLT1	9909	DENND4B	10752	CHL1	139221	MUM1L1
64132	XYLT2	9911	TMCC2	10776	ARPP19	139411	PTCHD1
64135	IFIH1	10023	FRAT1	10783	NEK6	140733	MACROD2
64137	ABCG4	10025	MED16	10797	MTHFD2	144348	ZNF664
64149	C17orf75	10026	PIGK	10815	CPLX1	144402	CPNE8
64333	ARHGAP9	10039	PARP3	10857	PGRMC1	147463	ANKRD29
64850	ETNPPL	10040	TOM1L1	10867	TSPAN9	147968	CAPN12
65992	DDRGK1	10083	USH1C	10891	PPARGC1A	148014	TTC9B
65997	RASL11B	10154	PLXNC1	11069	RAPGEF4	149473	CCDC24
66000	TMEM108	10160	FARP1	11095	ADAMTS8	152273	FGD5
66008	TRAK2	10171	RCL1	11118	BTN3A2	152940	C4orf45
79012	CAMKV	10231	RCAN2	11138	TBC1D8	160760	PPTC7
79017	GGCT	10268	RAMP3	11151	CORO1A	162494	RHBDL3
79183	TTPAL	10318	TNIP1	11164	NUDT5	163183	SYNE4
79585	CORO7	10332	CLEC4M	11167	FSTL1	163732	CITED4
79645	EFCAB1	10368	CACNG3	11211	FZD10	163782	KANK4
79660	PPP1R3B	10395	DLC1	11228	RASSF8	165215	FAM171B
79720	VPS37B	10402	ST3GAL6	11259	FILIP1L	167691	LCA5
79745	CLIP4	10425	ARIH2	11279	KLF8	192668	CYS1
79750	ZNF385D	10451	VAV3	22801	ITGA11	196383	RILPL2
79754	ASB13	10478	SLC25A17	22881	ANKRD6	200058	FLJ23867
79822	ARHGAP28	10505	SEMA4F	22987	SV2C	200942	KLHDC8B
79874	RABEP2	10560	SLC19A2	22996	TTC39A	203286	ANKS6
79930	DOK3	10613	ERLIN1	23046	KIF21B	221294	NT5DC1
79958	DENND1C	10636	RGS14	23109	DDN	221421	RSPH9
79993	ELOVL7	10669	CGREF1	23180	RFTN1	222537	HS3ST5
80020	FOXRED2	10673	TNFSF13B	23212	RRS1	253832	ZDHHC20
80036	TRPM3	10683	DLL3	23245	ASTN2	254102	EHBP1L1
80119	PIF1	10776	ARPP19	23259	DDHD2	260434	PYDC1
80176	SPSB1	10783	NEK6	23263	MCF2L	282969	FUOM
80179	MYO19	10797	MTHFD2	23274	CLEC16A	282973	JAKMIP3
80307	FER1L4	10815	CPLX1	23305	ACSL6	283209	PGM2L1
80323	CCDC68	10857	PGRMC1	23327	NEDD4L	283284	IGSF22
80820	EEPD1	10858	CYP46A1	23370	ARHGEF18	284348	LYPD5
80854	SETD7	10867	TSPAN9	23406	COTL1	284415	VSTM1
81033	KCNH6	10891	PPARGC1A	23428	SLC7A8	285780	LY86-AS1
81539	SLC38A1	10988	METAP2	23460	ABCA6	286133	SCARA5
81552	VOPP1	11069	RAPGEF4	23467	NPTXR	326624	RAB37
81849	ST6GALNAC5	11095	ADAMTS8	23484	LEPROTL1	342667	STAC2
83445	GSG1	11118	BTN3A2	23504	RIMBP2	348013	TMEM255B
83468	GLT8D2	11138	TBC1D8	23642	SNHG1	349136	WDR86
83482	SCRT1	11151	CORO1A	23780	APOL2	373156	GSTK1
83547	RILP	11164	NUDT5	25759	SHC2	374378	GALNT18
83660	TLN2	11167	FSTL1	25769	SLC24A2	374875	HSD11B1L
83690	CRISPLD1	11211	FZD10	25818	KLK5	375057	STUM
83692	CD99L2	11228	RASSF8	25841	ABTB2	386618	KCTD4
83707	TRPT1	11259	FILIP1L	25854	FAM149A	400745	SH2D5
83714	NRIP2	11279	KLF8	25871	NEPRO	404217	CTXN1
83875	BCO2	22801	ITGA11	25900	IFFO1	407738	FAM19A1
84034	EMILIN2	22824	HSPA4L	25907	TMEM158	415116	PIM3
84063	KIRREL2	22881	ANKRD6	25924	MYRIP	494470	RNF165
84109	QRFPR	22987	SV2C	25946	ZNF385A	503542	SPRN
84187	TMEM164	22996	TTC39A	25953	PNKD	574036	SERTAD4-AS1
84221	SPATC1L	23046	KIF21B	25989	ULK3	642273	FAM110C
84439	HHIPL1	23094	SIPA1L3	26010	SPATS2L	654502	IQCJ
84524	ZC3H8	23109	DDN	26031	OSBPL3	100507436	MICA
84525	HOPX	23111	SPART	26059	ERC2		
84623	KIRREL3	23180	RFTN1	26232	FBXO2		
84803	GPAT3	23199	GSE1	26996	GPR160		
84812	PLCD4	23212	RRS1	27077	B9D1		
84936	ZFYVE19	23259	DDHD2	27132	CPNE7		
84937	ZNRF1	23263	MCF2L	27163	NAAA		
85352	SHISAL1	23274	CLEC16A	27254	CSDC2		
85461	TANC1	23305	ACSL6	27294	DHDH		
89846	FGD3	23325	WASHC4	27345	KCNMB4		
89958	SAPCD2	23406	COTL1	28955	DEXI		
90102	PHLDB2	23428	SLC7A8	29115	SAP30BP		
90523	MLIP	23460	ABCA6	29126	CD274		
90861	JPT2	23467	NPTXR	29799	YPEL1		
90865	IL33	23479	ISCU	29803	REPIN1		
91133	L3MBTL4	23484	LEPROTL1	29844	TFPT		
91252	SLC39A13	23504	RIMBP2	29902	FAM216A		
91624	NEXN	23516	SLC39A14	29997	NOP53		
91683	SYT12	23642	SNHG1	30850	CDR2L		
92335	STRADA	23780	APOL2	49855	SCAPER		
92399	MRRF	25759	SHC2	50486	G0S2		
92597	MOB1B	25769	SLC24A2	50853	VILL		
92610	TIFA	25818	KLK5	51059	FAM135B		
94160	ABCC12	25841	ABTB2	51074	APIP		
113452	TMEM54	25854	FAM149A	51105	PHF20L1		
113675	SDSL	25871	NEPRO	51123	ZNF706		
114571	SLC22A9	25900	IFFO1	51134	CEP83		
114787	GPRIN1	25907	TMEM158	51155	JPT1		
114990	VASN	25924	MYRIP	51161	C3orf18		
116135	LRRC3B	25946	ZNF385A	51312	SLC25A37		
116535	MRGPRF	25953	PNKD	51319	RSRC1		
117154	DACH2	25989	ULK3	51334	PRR16		
117245	HRASLS5	26010	SPATS2L	51375	SNX7		
118427	OLFM3	26031	OSBPL3	51393	TRPV2		
118429	ANTXR2	26059	ERC2	51440	HPCAL4		
119587	CPXM2	26232	FBXO2	51454	GULP1		
122622	ADSSL1	26472	PPP1R14B	51522	TMEM14C		
122953	JDP2	26996	GPR160	51538	ZCCHC17		
126755	LRRC38	27077	B9D1	51642	MRPL48		
127833	SYT2	27132	CPNE7	51660	MPC1		
130399	ACVR1C	27163	NAAA	51661	FKBP7		
132160	PPM1M	27252	KLHL20	51667	NUB1		
132321	C4orf33	27254	CSDC2	53616	ADAM22		
133418	EMB	27294	DHDH	53826	FXYD6		
134548	SOWAHA	27341	RRP7A	54112	GPR88		
139221	MUM1L1	27345	KCNMB4	54492	NEURL1B		
139411	PTCHD1	28231	SLCO4A1	54536	EXOC6		
143279	HECTD2	28955	DEXI	54550	NECAB2		
144402	CPNE8	28966	SNX24	54551	MAGEL2		
147463	ANKRD29	29115	SAP30BP	54566	EPB41L4B		
147968	CAPN12	29126	CD274	54793	KCTD9		
148281	SYT6	29799	YPEL1	54843	SYTL2		

149473	CCDC24
152189	CMTM8
152273	FGD5
152940	C4orf45
155382	VPS37D
160760	PPTC7
162494	RHBDL3
163183	SYNE4
163782	KANK4
196383	RILPL2
199720	GGN
200058	FLJ23867
200942	KLHDC8B
201191	SAMD14
202333	CMYA5
220202	ATOH7
221294	NT5DC1
221336	BEND6
221421	RSPH9
222537	HS3ST5
253832	ZDHHC20
254102	EHBP1L1
260434	PYDC1
283209	PGM2L1
283284	IGSF22
283316	CD163L1
284119	CAVIN1
284339	TMEM145
284348	LYPD5
284415	VSTM1
284454	LOC284454
284611	FAM102B
284716	RIMKLA
285755	PPIL6
285780	LY86-AS1
286133	SCARA5
326624	RAB37
339983	NAT8L
340348	TSPAN33
340719	NANOS1
342667	STAC2
348013	TMEM255B
349136	WDR86
373156	GSTK1
373863	DND1
374378	GALNT18
386618	KCTD4
387357	THEMIS
389073	C2orf80
400569	MED11
400745	SH2D5
404217	CTXN1
415116	PIM3
503542	SPRN
574036	SERTAD4-AS1
642273	FAM110C
646424	SPINK8
646627	LYPD8
654502	IQCJ
654790	PCP4L1
100507436	MICA
29803	REPIN1
29844	TFPT
29902	FAM216A
29904	EEF2K
30850	CDR2L
50486	G0S2
50853	VILL
51022	GLRX2
51043	ZBTB7B
51059	FAM135B
51074	APIP
51105	PHF20L1
51123	ZNF706
51134	CEP83
51155	JPT1
51312	SLC25A37
51319	RSRC1
51330	TNFRSF12A
51334	PRR16
51375	SNX7
51389	RWDD1
51393	TRPV2
51440	HPCAL4
51454	GULP1
51522	TMEM14C
51538	ZCCHC17
51635	DHRS7
51642	MRPL48
51660	MPC1
51667	NUB1
53826	FXYD6
54112	GPR88
54206	ERRFI1
54331	GNG2
54407	SLC38A2
54492	NEURL1B
54536	EXOC6
54550	NECAB2
54551	MAGEL2
54566	EPB41L4B
54793	KCTD9
54843	SYTL2
54847	SIDT1
54874	FNBP1L
54972	TMEM132A
55006	TRMT61B
55022	PID1
55040	EPN3
55062	WIPI1
55086	CXorf57
55118	CRTAC1
55122	AKIRIN2
55160	ARHGEF10L
55170	PRMT6
55203	LGI2
55208	DCUN1D2
55244	SLC47A1
55315	SLC29A3
55353	LAPTM4B
55359	STYK1
55502	HES6
55509	BATF3
55591	VEZT
55686	MREG
55714	TENM3
55790	CSGALNACT1
55800	SCN3B
55853	IDI2-AS1
55884	WSB2
55897	MESP1
55968	NSFL1C
56172	ANKH
56616	DIABLO
56648	EIF5A2
56666	PANX2
56848	SPHK2
56884	FSTL5
56888	KCMF1
56906	THAP10
56934	CA10
56937	PMEPA1
56967	C14orf132
57110	HRASLS
57185	NIPAL3
57194	ATP10A
57406	ABHD6
57453	DSCAML1
57465	TBC1D24
57484	RNF150
57495	NWD2
57496	MKL2
57519	STARD9
57526	PCDH19
57554	LRRC7
57596	BEGAIN
57631	LRCH2
57639	CCDC146
57644	MYH7B
57699	CPNE5
57718	PPP4R4
60625	DHX35
63974	NEUROD6
63982	ANO3
64131	XYLT1
64132	XYLT2
64135	IFIH1
64137	ABCG4
54847	SIDT1
54874	FNBP1L
54972	TMEM132A
55022	PID1
55040	EPN3
55086	CXorf57
55118	CRTAC1
55122	AKIRIN2
55160	ARHGEF10L
55170	PRMT6
55190	NUDT11
55208	DCUN1D2
55225	RAVER2
55244	SLC47A1
55282	LRRC36
55315	SLC29A3
55353	LAPTM4B
55359	STYK1
55502	HES6
55509	BATF3
55530	SVOP
55591	VEZT
55686	MREG
55714	TENM3
55800	SCN3B
55853	IDI2-AS1
55884	WSB2
55897	MESP1
56172	ANKH
56256	SERTAD4
56616	DIABLO
56648	EIF5A2
56666	PANX2
56848	SPHK2
56884	FSTL5
56906	THAP10
56927	GPR108
56934	CA10
56937	PMEPA1
56967	C14orf132
56971	CEACAM19
57110	HRASLS
57194	ATP10A
57406	ABHD6
57453	DSCAML1
57465	TBC1D24
57484	RNF150
57495	NWD2
57496	MKL2
57519	STARD9
57526	PCDH19
57554	LRRC7
57596	BEGAIN
57630	SH3RF1
57631	LRCH2
57644	MYH7B
57699	CPNE5
57718	PPP4R4
60626	RIC8A
63941	NECAB3
63974	NEUROD6
63982	ANO3
64131	XYLT1
64132	XYLT2
64135	IFIH1
64137	ABCG4
64149	C17orf75
64150	DIO3OS
64333	ARHGAP9
64792	IFT22
64881	PCDH20
65078	RTN4R
65110	UPF3A
65263	PYCR3
65982	ZSCAN18
65997	RASL11B
66000	TMEM108
66008	TRAK2
78990	OTUB2
79012	CAMKV
79017	GGCT
79085	SLC25A23
79183	TTPAL
79442	LRRC2
79585	CORO7
79645	EFCAB1
79660	PPP1R3B
79720	VPS37B
79745	CLIP4
79750	ZNF385D
79754	ASB13
79822	ARHGAP28
79874	RABEP2
79884	MAP9
79930	DOK3
79957	PAQR6
79958	DENND1C
79993	ELOVL7
80020	FOXRED2
80036	TRPM3
80119	PIF1
80176	SPSB1
80179	MYO19
80213	TM2D3
80307	FER1L4
80323	CCDC68
80816	ASXL3

64149	C17orf75	80818	ZNF436
64150	DIO3OS	80820	EEPD1
64332	NFKBIZ	80854	SETD7
64333	ARHGAP9	81033	KCNH6
64420	SUSD1	81539	SLC38A1
64781	CERK	81552	VOPP1
64792	IFT22	81602	CDADC1
64881	PCDH20	81605	URM1
65078	RTN4R	81849	ST6GALNAC5
65110	UPF3A	83445	GSG1
65263	PYCR3	83468	GLT8D2
65992	DDRGK1	83482	SCRT1
65997	RASL11B	83546	RTBDN
66000	TMEM108	83547	RILP
66008	TRAK2	83660	TLN2
78990	OTUB2	83690	CRISPLD1
79012	CAMKV	83692	CD99L2
79016	DDA1	83707	TRPT1
79017	GGCT	83714	NRIP2
79075	DSCC1	83723	FAM57B
79085	SLC25A23	83787	ARMC10
79158	GNPTAB	83875	BCO2
79183	TTPAL	83937	RASSF4
79442	LRRC2	83992	CTTNBP2
79585	CORO7	84034	EMILIN2
79645	EFCAB1	84063	KIRREL2
79660	PPP1R3B	84083	ZRANB3
79720	VPS37B	84109	QRFPR
79745	CLIP4	84187	TMEM164
79750	ZNF385D	84221	SPATC1L
79754	ASB13	84314	TMEM107
79762	C1orf115	84332	DYDC2
79767	ELMO3	84439	HHIPL1
79822	ARHGAP28	84524	ZC3H8
79874	RABEP2	84525	HOPX
79884	MAP9	84542	KIAA1841
79887	PLBD1	84623	KIRREL3
79930	DOK3	84691	FAM71F1
79956	ERMP1	84709	MGARP
79957	PAQR6	84769	MPV17L2
79962	DNAJC22	84803	GPAT3
79990	PLEKHH3	84812	PLCD4
79993	ELOVL7	84864	RIOX2
80020	FOXRED2	84870	RSPO3
80036	TRPM3	84936	ZFYVE19
80119	PIF1	84937	ZNRF1
80176	SPSB1	84957	RELT
80179	MYO19	85015	USP45
80307	FER1L4	85352	SHISAL1
80323	CCDC68	85461	TANC1
80818	ZNF436	89782	LMLN
80820	EEPD1	89846	FGD3
80853	KDM7A	90102	PHLDB2
80854	SETD7	90488	TMEM263
81033	KCNH6	90523	MLIP
81539	SLC38A1	90850	ZNF598
81552	VOPP1	90861	JPT2
81602	CDADC1	90865	IL33
81605	URM1	91133	L3MBTL4
81619	TSPAN14	91252	SLC39A13
81849	ST6GALNAC5	91624	NEXN
83445	GSG1	91683	SYT12
83468	GLT8D2	92293	TMEM132C
83482	SCRT1	92335	STRADA
83547	RILP	92399	MRRF
83660	TLN2	92597	MOB1B
83690	CRISPLD1	92610	TIFA
83692	CD99L2	94160	ABCC12
83707	TRPT1	113263	GLCCI1
83714	NRIP2	113452	TMEM54
83723	FAM57B	113675	SDSL
83787	ARMC10	114571	SLC22A9
83875	BCO2	114787	GPRIN1
83937	RASSF4	114804	RNF157
83992	CTTNBP2	114990	VASN
84034	EMILIN2	116028	RMI2
84063	KIRREL2	116135	LRRC3B
84109	QRFPR	116150	NUS1
84187	TMEM164	116535	MRGPRF
84221	SPATC1L	117154	DACH2
84314	TMEM107	117245	HRASLS5
84332	DYDC2	118427	OLFM3
84439	HHIPL1	118429	ANTXR2
84525	HOPX	119587	CPXM2
84542	KIAA1841	122622	ADSSL1
84623	KIRREL3	122953	JDP2
84691	FAM71F1	126755	LRRC38
84769	MPV17L2	127833	SYT2
84803	GPAT3	128414	NKAIN4
84812	PLCD4	128434	VSTM2L
84864	RIOX2	128611	ZNF831
84936	ZFYVE19	130399	ACVR1C
84937	ZNRF1	132160	PPM1M
84957	RELT	132321	C4orf33
85015	USP45	133418	EMB
85352	SHISAL1	134548	SOWAHA
85461	TANC1	139221	MUM1L1
87178	PNPT1	139411	PTCHD1
88455	ANKRD13A	139728	PNCK
89782	LMLN	143279	HECTD2
89846	FGD3	144348	ZNF664
90102	PHLDB2	144402	CPNE8
90488	TMEM263	146760	RTN4RL1
90523	MLIP	147463	ANKRD29
90850	ZNF598	147650	SPACA6
90861	JPT2	147968	CAPN12
91133	L3MBTL4	148281	SYT6

91252	SLC39A13
91624	NEXN
91683	SYT12
92293	TMEM132C
92335	STRADA
92399	MRRF
92597	MOB1B
92610	TIFA
93587	TRMT10A
113263	GLCCI1
113452	TMEM54
113675	SDSL
113829	SLC35A4
114571	SLC22A9
114787	GPRIN1
114800	CCDC85A
114801	TMEM200A
114804	RNF157
114880	OSBPL6
114990	VASN
115584	SLC5A11
116028	RMI2
116135	LRRC3B
116535	MRGPRF
116832	RPL39L
117154	DACH2
117245	HRASLS5
118427	OLFM3
119587	CPXM2
122622	ADSSL1
122953	JDP2
124976	SPNS2
126755	LRRC38
127003	C1orf194
127833	SYT2
128434	VSTM2L
130399	ACVR1C
132160	PPM1M
132321	C4orf33
133418	EMB
134548	SOWAHA
139221	MUM1L1
139411	PTCHD1
139728	PNCK
140733	MACROD2
143279	HECTD2
144348	ZNF664
144402	CPNE8
146760	RTN4RL1
147463	ANKRD29
147968	CAPN12
148014	TTC9B
148281	SYT6
149473	CCDC24
150209	AIFM3
152189	CMTM8
152273	FGD5
152940	C4orf45
154141	MBOAT1
154790	CLEC2L
160760	PPTC7
162494	RHBDL3
163183	SYNE4
163732	CITED4
163782	KANK4
165215	FAM171B
166752	FREM3
167681	PRSS35
167691	LCA5
170261	ZCCHC12
170850	KCNG3
192668	CYS1
196383	RILPL2
199800	ADM5
200058	FLJ23867
200942	KLHDC8B
201191	SAMD14
202333	CMYA5
203286	ANKS6
219348	PLAC9
220164	DOK6
220202	ATOH7
221294	NT5DC1
221336	BEND6
221421	RSPH9
222537	HS3ST5
253832	ZDHHC20
254102	EHBP1L1
254170	FBXO33
254263	CNIH2
254552	NUDT8
256281	NUDT14
260434	PYDC1
282969	FUOM
282973	JAKMIP3
283143	LINC00900
283209	PGM2L1
283284	IGSF22
283316	CD163L1
284119	CAVIN1
284339	TMEM145
284348	LYPD5
284415	VSTM1
284454	LOC284454
284485	RIIAD1
284611	FAM102B
284716	RIMKLA
149473	CCDC24
150209	AIFM3
152189	CMTM8
152273	FGD5
152940	C4orf45
154790	CLEC2L
155382	VPS37D
160760	PPTC7
162394	SLFN5
162494	RHBDL3
163183	SYNE4
163782	KANK4
165215	FAM171B
167681	PRSS35
167691	LCA5
170850	KCNG3
192668	CYS1
196383	RILPL2
199800	ADM5
200058	FLJ23867
200942	KLHDC8B
201191	SAMD14
202333	CMYA5
203286	ANKS6
220202	ATOH7
221294	NT5DC1
221336	BEND6
221421	RSPH9
222537	HS3ST5
253314	EIF4E1B
253832	ZDHHC20
254102	EHBP1L1
254170	FBXO33
254263	CNIH2
254552	NUDT8
256281	NUDT14
260434	PYDC1
282969	FUOM
283143	LINC00900
283209	PGM2L1
283284	IGSF22
283316	CD163L1
284339	TMEM145
284348	LYPD5
284415	VSTM1
284454	LOC284454
284485	RIIAD1
284611	FAM102B
284716	RIMKLA
285220	EPHA6
285464	CRIPAK
285598	ARL10
285755	PPIL6
285780	LY86-AS1
286133	SCARA5
326624	RAB37
339983	NAT8L
340348	TSPAN33
340719	NANOS1
342667	STAC2
347902	AMIGO2
348013	TMEM255B
349136	WDR86
373156	GSTK1
373863	DND1
374378	GALNT18
374875	HSD11B1L
375057	STUM
375449	MAST4
386618	KCTD4
387357	THEMIS
387775	SLC22A10
389073	C2orf80
389941	C1QL3
400120	SERTM1
400569	MED11
400745	SH2D5
401994	OR14I1
404217	CTXN1
407738	FAM19A1
415116	PIM3
441027	TMEM150C
441108	C5orf56
494470	RNF165
503542	SPRN
574029	DUSP5P1
574036	SERTAD4-AS1
642273	FAM110C
642852	LOC642852
643037	C11orf97
646627	LYPD8
654502	IQCJ
654790	PCP4L1
100170841	EPOP
100288911	LOC100288911
100507436	MICA

285220	EPHA6
285598	ARL10
285613	RELL2
285755	PPIL6
285780	LY86-AS1
286133	SCARA5
286336	FAM78A
326624	RAB37
339829	CCDC39
339983	NAT8L
340719	NANOS1
342667	STAC2
347730	LRRTM1
347902	AMIGO2
348013	TMEM255B
349136	WDR86
353149	TBC1D26
373156	GSTK1
373863	DND1
374378	GALNT18
374875	HSD11B1L
375057	STUM
375449	MAST4
386618	KCTD4
387775	SLC22A10
389073	C2orf80
389941	C1QL3
400120	SERTM1
400569	MED11
400745	SH2D5
401647	GOLGA7B
404037	HAPLN4
404217	CTXN1
407738	FAM19A1
408263	FNDC9
415116	PIM3
441108	C5orf56
442319	ZNF727
494470	RNF165
503542	SPRN
574036	SERTAD4-AS1
642273	FAM110C
642852	LOC642852
643037	C11orf97
646627	LYPD8
654502	IQCJ
654790	PCP4L1
728464	METTL24
100093630	SNHG8
100170841	EPOP
100288911	LOC100288911
100294145	LOC100294145
100507436	MICA

Abbreviations: rsFC, resting-state functional connectivity; DS, differential stability; A4hf, head and face region of area 4; A6cdl, caudal dorsolateral area 6; A4ul, upper limb region of area 4; A4ll, lower limb region of area 4; A1/2/3ulhf, upper limb, head and face region of area 1/2/3; A1/2/3tru, trunk region of area 1/2/3.

The genes related to rsFC of sensorimotor subregions at the DS cutoff threshold of 60%

A4hf		A6cdl		A4ul		A4ll		A1/2/3ulhf		A1/2/3tru	
GeneID	Genesymbol	GeneID	Genesymbol	GeneID	Genesymbol	GeneID	Genesymbol	GeneID	Genesymbol	GeneID	Genesymbol
92	ACVR2A	92	ACVR2A	92	ACVR2A	176	ACAN	92	ACVR2A	92	ACVR2A
133	ADM	176	ACAN	98	ACYP2	341	APOC1	98	ACYP2	98	ACYP2
176	ACAN	326	AIRE	115	ADCY9	784	CACNB3	115	ADCY9	176	ACAN
204	AK2	793	CALB1	133	ADM	793	CALB1	133	ADM	286	ANK1
286	ANK1	817	CAMK2D	161	AP2A2	814	CAMK4	161	AP2A2	308	ANXA5
320	APBA1	1893	ECM1	176	ACAN	817	CAMK2D	176	ACAN	320	APBA1
367	AR	2044	EPHA5	204	AK2	2044	EPHA5	204	AK2	341	APOC1
430	ASCL2	2830	GPR6	220	ALDH1A3	2830	GPR6	249	ALPL	367	AR
445	ASS1	2952	GSTT1	249	ALPL	3679	ITGA7	272	AMPD3	430	ASCL2
481	ATP1B1	3290	HSD11B1	272	AMPD3	4703	NEB	286	ANK1	445	ASS1
483	ATP1B3	3736	KCNA1	286	ANK1	5176	SERPINF1	292	SLC25A5	460	ASTN1
563	AZGP1	3755	KCNG1	292	SLC25A5	5794	PTPRH	308	ANXA5	493	ATP2B4
586	BCAT1	3787	KCNS1	308	ANXA5	5961	PRPH2	320	APBA1	586	BCAT1
613	BCR	4082	MARCKS	320	APBA1	6330	SCN4B	366	AQP9	784	CACNB3
784	CACNB3	4599	MX1	341	APOC1	9473	THEMIS2	367	AR	793	CALB1
793	CALB1	4741	NEFM	367	AR	10268	RAMP3	396	ARHGDIA	814	CAMK4
814	CAMK4	4744	NEFH	430	ASCL2	22987	SV2C	430	ASCL2	817	CAMK2D
817	CAMK2D	5121	PCP4	445	ASS1	50486	G0S2	445	ASS1	818	CAMK2G
818	CAMK2G	5409	PNMT	460	ASTN1	55244	SLC47A1	460	ASTN1	1002	CDH4
869	CBLN1	6330	SCN4B	481	ATP1B1	55315	SLC29A3	481	ATP1B1	1006	CDH8
1002	CDH4	6482	ST3GAL1	483	ATP1B3	79660	PPP1R3B	492	ATP2B3	1027	CDKN1B
1006	CDH8	8557	TCAP	492	ATP2B3	80307	FER1L4	493	ATP2B4	1073	CFL2
1050	CEBPA	9256	TSPOAP1	493	ATP2B4	84034	EMILIN2	528	ATP6V1C1	1272	CNTN1
1272	CNTN1	9473	THEMIS2	563	AZGP1	114571	SLC22A9	563	AZGP1	1294	COL7A1
1300	COL10A1	10160	FARP1	586	BCAT1	127833	SYT2	586	BCAT1	1300	COL10A1
1381	CRABP1	10231	RCAN2	627	BDNF	134548	SOWAHA	613	BCR	1501	CTNND2
1501	CTNND2	10268	RAMP3	631	BFSP1	148014	TTC9B	627	BDNF	1522	CTSZ
1522	CTSZ	10683	DLL3	715	C1R	163782	KANK4	715	C1R	1607	DGKB
1607	DGKB	22987	SV2C	716	C1S	221294	NT5DC1	784	CACNB3	1755	DMBT1
1730	DIAPH2	22996	TTC39A	784	CACNB3	254102	EHBP1L1	793	CALB1	1893	ECM1
1809	DPYSL3	23406	COTL1	793	CALB1	286133	SCARA5	808	CALM3	2044	EPHA5
1893	ECM1	25953	PNKD	808	CALM3			814	CAMK4	2070	EYA4
1948	EFNB2	29799	YPEL1	814	CAMK4			817	CAMK2D	2101	ESRRA
2027	ENO3	50486	G0S2	817	CAMK2D			818	CAMK2G	2104	ESRRG
2044	EPHA5	50853	VILL	818	CAMK2G			862	RUNX1T1	2161	F12
2070	EYA4	54492	NEURL1B	831	CAST			869	CBLN1	2322	FLT3
2101	ESRRA	54793	KCTD9	955	ENTPD6			955	ENTPD6	2560	GABRB1
2104	ESRRG	55040	EPN3	1002	CDH4			1002	CDH4	2562	GABRB3
2161	F12	55244	SLC47A1	1006	CDH8			1005	CDH7	2565	GABRG1
2173	FABP7	55686	MREG	1007	CDH9			1006	CDH8	2634	GBP2
2254	FGF9	56967	C14orf132	1027	CDKN1B			1007	CDH9	2742	GLRA2
2268	FGR	79660	PPP1R3B	1031	CDKN2C			1027	CDKN1B	2766	GMPR
2289	FKBP5	79767	ELMO3	1032	CDKN2D			1031	CDKN2C	2823	GPM6A
2322	FLT3	80212	CCDC92	1040	CDS1			1032	CDKN2D	2830	GPR6
2560	GABRB1	80307	FER1L4	1050	CEBPA			1040	CDS1	2904	GRIN2B
2565	GABRG1	83547	RILP	1073	CFL2			1050	CEBPA	2911	GRM1
2620	GAS2	84034	EMILIN2	1176	AP3S1			1073	CFL2	2937	GSS
2632	GBE1	90523	MLIP	1272	CNTN1			1272	CNTN1	2952	GSTT1
2634	GBP2	113675	SDSL	1294	COL7A1			1294	COL7A1	3067	HDC
2742	GLRA2	114787	GPRIN1	1299	COL9A3			1300	COL10A1	3290	HSD11B1
2766	GMPR	127833	SYT2	1300	COL10A1			1381	CRABP1	3350	HTR1A
2778	GNAS	134548	SOWAHA	1381	CRABP1			1501	CTNND2	3624	INHBA
2823	GPM6A	160760	PPTC7	1397	CRIP2			1522	CTSZ	3679	ITGA7
2830	GPR6	163782	KANK4	1501	CTNND2			1523	CUX1	3688	ITGB1
2888	GRB14	192668	CYS1	1522	CTSZ			1607	DGKB	3736	KCNA1
2898	GRIK2	222537	HS3ST5	1607	DGKB			1730	DIAPH2	3738	KCNA3
2911	GRM1	286133	SCARA5	1730	DIAPH2			1755	DMBT1	3741	KCNA5
2918	GRM8	326624	RAB37	1755	DMBT1			1768	DNAH6	3755	KCNG1
2952	GSTT1	342667	STAC2	1809	DPYSL3			1809	DPYSL3	3783	KCNN4
3067	HDC	415116	PIM3	1893	ECM1			1871	E2F3	3910	LAMA4
3231	HOXD1	642273	FAM110C	1949	EFNB3			1875	E2F5	4062	LY6H
3269	HRH1	654502	IQCJ	2027	ENO3			1893	ECM1	4082	MARCKS
3290	HSD11B1			2044	EPHA5			1909	EDNRA	4201	MEA1
3316	HSPB2			2070	EYA4			1948	EFNB2	4258	MGST2
3350	HTR1A			2101	ESRRA			1949	EFNB3	4286	MITF
3396	MRPL58			2104	ESRRG			1960	EGR3	4345	CD200
3624	INHBA			2161	F12			2027	ENO3	4354	MPP1
3632	INPP5A			2170	FABP3			2044	EPHA5	4599	MX1
3671	ISLR			2173	FABP7			2070	EYA4	4609	MYC
3679	ITGA7			2182	ACSL4			2101	ESRRA	4638	MYLK
3688	ITGB1			2230	FDX1			2104	ESRRG	4703	NEB
3736	KCNA1			2254	FGF9			2161	F12	4744	NEFH
3738	KCNA3			2322	FLT3			2170	FABP3	4751	NEK2
3741	KCNA5			2558	GABRA5			2173	FABP7	4886	NPY1R
3755	KCNG1			2560	GABRB1			2182	ACSL4	4889	NPY5R
3782	KCNN3			2562	GABRB3			2254	FGF9	5091	PC
3783	KCNN4			2565	GABRG1			2322	FLT3	5116	PCNT
3827	KNG1			2620	GAS2			2558	GABRA5	5121	PCP4
3902	LAG3			2632	GBE1			2560	GABRB1	5138	PDE2A
3910	LAMA4			2634	GBP2			2562	GABRB3	5141	PDE4A
4062	LY6H			2690	GHR			2565	GABRG1	5176	SERPINF1
4082	MARCKS			2742	GLRA2			2620	GAS2	5409	PNMT
4092	SMAD7			2764	GMFB			2634	GBP2	5412	UBL3
4157	MC1R			2766	GMPR			2690	GHR	5569	PKIA
4201	MEA1			2778	GNAS			2742	GLRA2	5580	PRKCD
4257	MGST1			2786	GNG4			2766	GMPR	5582	PRKCG
4258	MGST2			2790	GNG10			2778	GNAS	5733	PTGER3
4286	MITF			2820	GPD2			2823	GPM6A	5774	PTPN3
4324	MMP15			2823	GPM6A			2830	GPR6	5794	PTPRH
4354	MPP1			2830	GPR6			2888	GRB14	5860	QDPR
4599	MX1			2888	GRB14			2890	GRIA1	5874	RAB27B
4609	MYC			2890	GRIA1			2898	GRIK2	5961	PRPH2
4616	GADD45B			2898	GRIK2			2904	GRIN2B	5993	RFX5
4638	MYLK			2904	GRIN2B			2911	GRM1	6017	RLBP1
4703	NEB			2911	GRM1			2918	GRM8	6272	SORT1
4715	NDUFB9			2918	GRM8			2937	GSS	6330	SCN4B
4744	NEFH			2937	GSS			2947	GSTM3	6543	SLC8A2
4751	NEK2			2947	GSTM3			2952	GSTT1	6646	SOAT1
4856	NOV			2952	GSTT1			3067	HDC	6804	STX1A
4885	NPTX2			2958	GTF2A2			3145	HMBS	6843	VAMP1
4886	NPY1R			3067	HDC			3156	HMGCR	6890	TAP1
5116	PCNT			3145	HMBS			3231	HOXD1	6905	TBCE

5121	PCP4	3156	HMGCR	3269	HRH1	7062	TCHH
5141	PDE4A	3231	HOXD1	3290	HSD11B1	7068	THRB
5176	SERPINF1	3269	HRH1	3316	HSPB2	7092	TLL1
5184	PEPD	3290	HSD11B1	3338	DNAJC4	7103	TSPAN8
5272	SERPINB9	3295	HSD17B4	3350	HTR1A	7138	TNNT1
5292	PIM1	3316	HSPB2	3396	MRPL58	7145	TNS1
5332	PLCB4	3338	DNAJC4	3485	IGFBP2	7480	WNT10B
5409	PNMT	3350	HTR1A	3595	IL12RB2	7534	YWHAZ
5412	UBL3	3358	HTR2C	3598	IL13RA2	7781	SLC30A3
5467	PPARD	3396	MRPL58	3624	INHBA	8001	GLRA3
5547	PRCP	3423	IDS	3632	INPP5A	8209	C21orf33
5569	PKIA	3598	IL13RA2	3653	IPW	8482	SEMA7A
5580	PRKCD	3624	INHBA	3679	ITGA7	8611	PLPP1
5582	PRKCG	3632	INPP5A	3688	ITGB1	8704	B4GALT2
5606	MAP2K3	3653	IPW	3736	KCNA1	8715	NOL4
5774	PTPN3	3679	ITGA7	3738	KCNA3	8717	TRADD
5792	PTPRF	3688	ITGB1	3741	KCNA5	8792	TNFRSF11A
5794	PTPRH	3706	ITPKA	3746	KCNC1	8871	SYNJ2
5800	PTPRO	3736	KCNA1	3755	KCNG1	8884	SLC5A6
5816	PVALB	3738	KCNA3	3782	KCNN3	8938	BAIAP3
5860	QDPR	3739	KCNA4	3783	KCNN4	9033	PKD2L1
5874	RAB27B	3741	KCNA5	3787	KCNS1	9127	P2RX6
5937	RBMS1	3746	KCNC1	3827	KNG1	9196	KCNAB3
5950	RBP4	3755	KCNG1	3902	LAG3	9256	TSPOAP1
5954	RCN1	3783	KCNN4	3910	LAMA4	9312	KCNB2
5979	RET	3787	KCNS1	4062	LY6H	9454	HOMER3
5993	RFX5	3827	KNG1	4082	MARCKS	9473	THEMIS2
6017	RLBP1	3902	LAG3	4088	SMAD3	9607	CARTPT
6122	RPL3	3910	LAMA4	4092	SMAD7	9609	RAB36
6272	SORT1	4062	LY6H	4157	MC1R	9636	ISG15
6324	SCN1B	4082	MARCKS	4168	MCF2	9653	HS2ST1
6330	SCN4B	4092	SMAD7	4201	MEA1	9731	CEP104
6451	SH3BGRL	4157	MC1R	4257	MGST1	9854	C2CD2L
6533	SLC6A6	4168	MCF2	4258	MGST2	9911	TMCC2
6646	SOAT1	4201	MEA1	4286	MITF	10083	USH1C
6804	STX1A	4257	MGST1	4324	MMP15	10154	PLXNC1
6843	VAMP1	4258	MGST2	4345	CD200	10160	FARP1
6890	TAP1	4286	MITF	4354	MPP1	10231	RCAN2
6905	TBCE	4324	MMP15	4599	MX1	10268	RAMP3
7062	TCHH	4337	MOCS1	4602	MYB	10332	CLEC4M
7068	THRB	4345	CD200	4609	MYC	10368	CACNG3
7089	TLE2	4354	MPP1	4616	GADD45B	10384	BTN3A3
7092	TLL1	4599	MX1	4625	MYH7	10395	DLC1
7103	TSPAN8	4602	MYB	4638	MYLK	10451	VAV3
7138	TNNT1	4609	MYC	4703	NEB	10505	SEMA4F
7145	TNS1	4616	GADD45B	4715	NDUFB9	10669	CGREF1
7222	TRPC3	4625	MYH7	4744	NEFH	10673	TNFSF13B
7378	UPP1	4638	MYLK	4751	NEK2	10683	DLL3
7402	UTRN	4703	NEB	4753	NELL2	10776	ARPP19
7409	VAV1	4715	NDUFB9	4782	NFIC	10783	NEK6
7480	WNT10B	4744	NEFH	4824	NKX3-1	10891	PPARGC1A
7781	SLC30A3	4751	NEK2	4856	NOV	11069	RAPGEF4
8001	GLRA3	4753	NELL2	4885	NPTX2	11138	TBC1D8
8174	MADCAM1	4782	NFIC	4886	NPY1R	11151	CORO1A
8209	C21orf33	4856	NOV	4889	NPY5R	11164	NUDT5
8321	FZD1	4878	NPPA	4988	OPRM1	11259	FILIP1L
8437	RASAL1	4885	NPTX2	5090	PBX3	22801	ITGA11
8482	SEMA7A	4886	NPY1R	5091	PC	22881	ANKRD6
8604	SLC25A12	4889	NPY5R	5116	PCNT	22987	SV2C
8611	PLPP1	4968	OGG1	5121	PCP4	22996	TTC39A
8704	B4GALT2	4988	OPRM1	5138	PDE2A	23046	KIF21B
8715	NOL4	5064	PALM	5141	PDE4A	23109	DDN
8717	TRADD	5082	PDCL	5176	SERPINF1	23180	RFTN1
8792	TNFRSF11A	5090	PBX3	5184	PEPD	23406	COTL1
8884	SLC5A6	5091	PC	5272	SERPINB9	23484	LEPROTL1
8938	BAIAP3	5116	PCNT	5292	PIM1	23504	RIMBP2
9020	MAP3K14	5119	CHMP1A	5310	PKD1	23642	SNHG1
9033	PKD2L1	5121	PCP4	5325	PLAGL1	25841	ABTB2
9120	SLC16A6	5138	PDE2A	5332	PLCB4	25854	FAM149A
9127	P2RX6	5141	PDE4A	5361	PLXNA1	25871	NEPRO
9168	TMSB10	5176	SERPINF1	5409	PNMT	25924	MYRIP
9196	KCNAB3	5184	PEPD	5412	UBL3	25989	ULK3
9254	CACNA2D2	5272	SERPINB9	5467	PPARD	26010	SPATS2L
9256	TSPOAP1	5292	PIM1	5475	PPEF1	26059	ERC2
9312	KCNB2	5310	PKD1	5547	PRCP	27077	B9D1
9315	NREP	5330	PLCB2	5569	PKIA	27132	CPNE7
9452	ITM2A	5332	PLCB4	5579	PRKCB	27163	NAAA
9454	HOMER3	5409	PNMT	5580	PRKCD	27294	DHDH
9592	IER2	5412	UBL3	5582	PRKCG	28955	DEXI
9607	CARTPT	5453	POU3F1	5594	MAPK1	29799	YPEL1
9609	RAB36	5467	PPARD	5606	MAP2K3	29803	REPIN1
9636	ISG15	5480	PPIC	5733	PTGER3	30850	CDR2L
9828	ARHGEF17	5547	PRCP	5774	PTPN3	50486	G0S2
9911	TMCC2	5557	PRIM1	5786	PTPRA	50853	VILL
10083	USH1C	5569	PKIA	5792	PTPRF	51059	FAM135B
10154	PLXNC1	5570	PKIB	5794	PTPRH	51074	APIP
10160	FARP1	5579	PRKCB	5800	PTPRO	51312	SLC25A37
10171	RCL1	5580	PRKCD	5801	PTPRR	51375	SNX7
10231	RCAN2	5582	PRKCG	5816	PVALB	51393	TRPV2
10268	RAMP3	5594	MAPK1	5860	QDPR	51440	HPCAL4
10332	CLEC4M	5606	MAP2K3	5874	RAB27B	51454	GULP1
10384	BTN3A3	5733	PTGER3	5912	RAP2B	51660	MPC1
10395	DLC1	5774	PTPN3	5937	RBMS1	51667	NUB1
10451	VAV3	5786	PTPRA	5950	RBP4	54112	GPR88
10505	SEMA4F	5792	PTPRF	5954	RCN1	54492	NEURL1B
10613	ERLIN1	5794	PTPRH	5961	PRPH2	54536	EXOC6
10673	TNFSF13B	5800	PTPRO	5979	RET	54550	NECAB2
10891	PPARGC1A	5801	PTPRR	5991	RFX3	54551	MAGEL2
11069	RAPGEF4	5806	PTX3	5993	RFX5	54566	EPB41L4B
11138	TBC1D8	5816	PVALB	6017	RLBP1	54793	KCTD9
11164	NUDT5	5860	QDPR	6272	SORT1	54843	SYTL2
11259	FILIP1L	5874	RAB27B	6323	SCN1A	54847	SIDT1
11279	KLF8	5912	RAP2B	6324	SCN1B	55040	EPN3
22801	ITGA11	5937	RBMS1	6326	SCN2A	55160	ARHGEF10L
22881	ANKRD6	5950	RBP4	6330	SCN4B	55244	SLC47A1
22987	SV2C	5954	RCN1	6451	SH3BGRL	55315	SLC29A3
22996	TTC39A	5961	PRPH2	6509	SLC1A4	55509	BATF3

23046	KIF21B	5979	RET	6533	SLC6A6	55591	VEZT
23109	DDN	5991	RFX3	6543	SLC8A2	55686	MREG
23180	RFTN1	5993	RFX5	6567	SLC16A2	55800	SCN3B
23245	ASTN2	6017	RLBP1	6585	SLIT1	55853	IDI2-AS1
23274	CLEC16A	6122	RPL3	6623	SNCG	55897	MESP1
23305	ACSL6	6272	SORT1	6646	SOAT1	56477	CCL28
23406	COTL1	6320	CLEC11A	6696	SPP1	56934	CA10
23484	LEPROTL1	6323	SCN1A	6751	SSTR1	56937	PMEPA1
23504	RIMBP2	6324	SCN1B	6804	STX1A	56967	C14orf132
23642	SNHG1	6326	SCN2A	6843	VAMP1	57110	HRASLS
25841	ABTB2	6330	SCN4B	6854	SYN2	57453	DSCAML1
25900	IFFO1	6451	SH3BGRL	6890	TAP1	57495	NWD2
25907	TMEM158	6509	SLC1A4	6905	TBCE	57496	MKL2
25946	ZNF385A	6533	SLC6A6	6920	TCEA3	57526	PCDH19
25953	PNKD	6541	SLC7A1	7062	TCHH	57554	LRRC7
25989	ULK3	6543	SLC8A2	7068	THRB	57718	PPP4R4
26031	OSBPL3	6545	SLC7A4	7087	ICAM5	57761	TRIB3
26232	FBXO2	6567	SLC16A2	7089	TLE2	64132	XYLT2
27132	CPNE7	6585	SLIT1	7092	TLL1	64135	IFIH1
27163	NAAA	6646	SOAT1	7103	TSPAN8	64149	C17orf75
28955	DEXI	6676	SPAG4	7138	TNNT1	65078	RTN4R
29799	YPEL1	6696	SPP1	7145	TNS1	65997	RASL11B
29803	REPIN1	6751	SSTR1	7222	TRPC3	65999	LRRC61
29844	TFPT	6770	STAR	7301	TYRO3	66000	TMEM108
29997	NOP53	6804	STX1A	7378	UPP1	66008	TRAK2
30850	CDR2L	6843	VAMP1	7402	UTRN	79012	CAMKV
50486	G0S2	6854	SYN2	7480	WNT10B	79017	GGCT
51059	FAM135B	6890	TAP1	7533	YWHAH	79183	TTPAL
51074	APIP	6905	TBCE	7534	YWHAZ	79645	EFCAB1
51105	PHF20L1	6920	TCEA3	7726	TRIM26	79660	PPP1R3B
51134	CEP83	7062	TCHH	7781	SLC30A3	79720	VPS37B
51312	SLC25A37	7068	THRB	7791	ZYX	79745	CLIP4
51319	RSRC1	7087	ICAM5	8001	GLRA3	79750	ZNF385D
51334	PRR16	7089	TLE2	8013	NR4A3	79822	ARHGAP28
51375	SNX7	7092	TLL1	8174	MADCAM1	79958	DENND1C
51440	HPCAL4	7103	TSPAN8	8209	C21orf33	79993	ELOVL7
51454	GULP1	7138	TNNT1	8321	FZD1	80020	FOXRED2
51522	TMEM14C	7145	TNS1	8404	SPARCL1	80119	PIF1
51642	MRPL48	7222	TRPC3	8437	RASAL1	80176	SPSB1
51660	MPC1	7260	EIPR1	8445	DYRK2	80307	FER1L4
51705	EMCN	7301	TYRO3	8482	SEMA7A	80323	CCDC68
54112	GPR88	7347	UCHL3	8604	SLC25A12	80774	LIMD2
54536	EXOC6	7378	UPP1	8611	PLPP1	80820	EEPD1
54550	NECAB2	7402	UTRN	8704	B4GALT2	80854	SETD7
54551	MAGEL2	7409	VAV1	8706	B3GALNT1	81033	KCNH6
54566	EPB41L4B	7480	WNT10B	8715	NOL4	81553	FAM49A
54793	KCTD9	7533	YWHAH	8717	TRADD	81849	ST6GALNAC5
54843	SYTL2	7534	YWHAZ	8792	TNFRSF11A	83445	GSG1
54847	SIDT1	7726	TRIM26	8805	TRIM24	83468	GLT8D2
54874	FNBP1L	7781	SLC30A3	8871	SYNJ2	83660	TLN2
55040	EPN3	7791	ZYX	8877	SPHK1	83690	CRISPLD1
55086	CXorf57	7798	LUZP1	8884	SLC5A6	83714	NRIP2
55118	CRTAC1	8001	GLRA3	8938	BAIAP3	83787	ARMC10
55160	ARHGEF10L	8099	CDK2AP1	9020	MAP3K14	83875	BCO2
55170	PRMT6	8174	MADCAM1	9033	PKD2L1	84034	EMILIN2
55208	DCUN1D2	8193	DPF1	9120	SLC16A6	84109	QRFPR
55244	SLC47A1	8209	C21orf33	9127	P2RX6	84187	TMEM164
55315	SLC29A3	8321	FZD1	9168	TMSB10	84221	SPATC1L
55353	LAPTM4B	8404	SPARCL1	9196	KCNAB3	84439	HHIPL1
55509	BATF3	8425	LTBP4	9215	LARGE1	84525	HOPX
55591	VEZT	8437	RASAL1	9253	NUMBL	84623	KIRREL3
55686	MREG	8445	DYRK2	9254	CACNA2D2	84769	MPV17L2
55800	SCN3B	8470	SORBS2	9256	TSPOAP1	84937	ZNRF1
55853	IDI2-AS1	8482	SEMA7A	9312	KCNB2	85301	COL27A1
55897	MESP1	8507	ENC1	9315	NREP	85352	SHISAL1
56172	ANKH	8604	SLC25A12	9362	CPNE6	90102	PHLDB2
56648	EIF5A2	8611	PLPP1	9399	STOML1	90523	MLIP
56934	CA10	8675	STX16	9452	ITM2A	90850	ZNF598
56937	PMEPA1	8704	B4GALT2	9454	HOMER3	90861	JPT2
56967	C14orf132	8715	NOL4	9473	THEMIS2	91133	L3MBTL4
56971	CEACAM19	8717	TRADD	9495	AKAP5	91252	SLC39A13
57194	ATP10A	8792	TNFRSF11A	9592	IER2	91624	NEXN
57406	ABHD6	8805	TRIM24	9607	CARTPT	92335	STRADA
57453	DSCAML1	8853	ASAP2	9609	RAB36	113675	SDSL
57465	TBC1D24	8871	SYNJ2	9636	ISG15	114299	PALM2
57495	NWD2	8884	SLC5A6	9644	SH3PXD2A	114571	SLC22A9
57496	MKL2	8938	BAIAP3	9651	PLCH2	114787	GPRIN1
57526	PCDH19	9020	MAP3K14	9653	HS2ST1	114990	VASN
57596	BEGAIN	9033	PKD2L1	9750	RIPOR2	116028	RMI2
57718	PPP4R4	9120	SLC16A6	9770	RASSF2	116135	LRRC3B
63974	NEUROD6	9127	P2RX6	9823	ARMCX2	118427	OLFM3
63982	ANO3	9131	AIFM1	9828	ARHGEF17	119587	CPXM2
64131	XYLT1	9168	TMSB10	9854	C2CD2L	127833	SYT2
64132	XYLT2	9196	KCNAB3	9882	TBC1D4	128434	VSTM2L
64135	IFIH1	9253	NUMBL	9903	KLHL21	130399	ACVR1C
64149	C17orf75	9254	CACNA2D2	9911	TMCC2	132160	PPM1M
64333	ARHGAP9	9256	TSPOAP1	9957	HS3ST1	132321	C4orf33
64850	ETNPPL	9267	CYTH1	10023	FRAT1	134548	SOWAHA
65997	RASL11B	9312	KCNB2	10025	MED16	139221	MUM1L1
65998	C11orf95	9315	NREP	10026	PIGK	140733	MACROD2
66000	TMEM108	9362	CPNE6	10039	PARP3	144348	ZNF664
66008	TRAK2	9379	NRXN2	10040	TOM1L1	144402	CPNE8
79012	CAMKV	9382	COG1	10083	USH1C	147463	ANKRD29
79017	GGCT	9399	STOML1	10154	PLXNC1	147968	CAPN12
79183	TTPAL	9452	ITM2A	10160	FARP1	148014	TTC9B
79585	CORO7	9454	HOMER3	10171	RCL1	151516	ASPRV1
79645	EFCAB1	9473	THEMIS2	10231	RCAN2	152940	C4orf45
79660	PPP1R3B	9495	AKAP5	10268	RAMP3	160760	PPTC7
79720	VPS37B	9508	ADAMTS3	10318	TNIP1	162494	RHBDL3
79745	CLIP4	9537	TP53I11	10332	CLEC4M	163183	SYNE4
79750	ZNF385D	9570	GOSR2	10368	CACNG3	163732	CITED4
79754	ASB13	9592	IER2	10384	BTN3A3	163782	KANK4
79822	ARHGAP28	9607	CARTPT	10395	DLC1	192668	CYS1
79874	RABEP2	9609	RAB36	10402	ST3GAL6	196383	RILPL2
79930	DOK3	9630	GNA14	10425	ARIH2	200058	FLJ23867
79993	ELOVL7	9636	ISG15	10451	VAV3	200942	KLHDC8B
80020	FOXRED2	9651	PLCH2	10478	SLC25A17	203286	ANKS6

80036	TRPM3
80119	PIF1
80176	SPSB1
80179	MYO19
80307	FER1L4
80323	CCDC68
80774	LIMD2
80820	EEPD1
80854	SETD7
81033	KCNH6
81552	VOPP1
81553	FAM49A
81849	ST6GALNAC5
83445	GSG1
83468	GLT8D2
83482	SCRT1
83547	RILP
83660	TLN2
83690	CRISPLD1
83692	CD99L2
83707	TRPT1
83714	NRIP2
84034	EMILIN2
84109	QRFPR
84187	TMEM164
84221	SPATC1L
84439	HHIPL1
84524	ZC3H8
84525	HOPX
84623	KIRREL3
84803	GPAT3
84812	PLCD4
84936	ZFYVE19
84937	ZNRF1
85461	TANC1
89958	SAPCD2
90102	PHLDB2
90523	MLIP
90861	JPT2
90865	IL33
91133	L3MBTL4
91252	SLC39A13
91624	NEXN
92335	STRADA
92399	MRRF
92597	MOB1B
92610	TIFA
113452	TMEM54
113675	SDSL
114571	SLC22A9
114787	GPRIN1
114990	VASN
116135	LRRC3B
117154	DACH2
117245	HRASLS5
118427	OLFM3
118429	ANTXR2
119587	CPXM2
122622	ADSSL1
122953	JDP2
126755	LRRC38
127833	SYT2
130399	ACVR1C
132321	C4orf33
133418	EMB
134548	SOWAHA
139221	MUM1L1
139411	PTCHD1
143279	HECTD2
144402	CPNE8
147463	ANKRD29
147968	CAPN12
148281	SYT6
152189	CMTM8
152273	FGD5
152940	C4orf45
155382	VPS37D
160760	PPTC7
162494	RHBDL3
163183	SYNE4
163782	KANK4
196383	RILPL2
200058	FLJ23867
200942	KLHDC8B
201191	SAMD14
202333	CMYA5
219738	FAM241B
220202	ATOH7
221294	NT5DC1
221421	RSPH9
222537	HS3ST5
253832	ZDHHC20
254102	EHBP1L1
260434	PYDC1
283209	PGM2L1
283284	IGSF22
284119	CAVIN1
284339	TMEM145
284348	LYPD5
284415	VSTM1
284454	LOC284454
284716	RIMKLA
285755	PPIL6
285780	LY86-AS1
286133	SCARA5
326624	RAB37
340348	TSPAN33
9653	HS2ST1
9717	SEC14L5
9731	CEP104
9750	RIPOR2
9770	RASSF2
9790	BMS1
9823	ARMCX2
9854	C2CD2L
9882	TBC1D4
9903	KLHL21
9911	TMCC2
10023	FRAT1
10025	MED16
10026	PIGK
10039	PARP3
10040	TOM1L1
10083	USH1C
10152	ABI2
10154	PLXNC1
10160	FARP1
10171	RCL1
10231	RCAN2
10268	RAMP3
10318	TNIP1
10332	CLEC4M
10368	CACNG3
10384	BTN3A3
10395	DLC1
10402	ST3GAL6
10425	ARIH2
10451	VAV3
10478	SLC25A17
10505	SEMA4F
10613	ERLIN1
10636	RGS14
10669	CGREF1
10673	TNFSF13B
10683	DLL3
10776	ARPP19
10783	NEK6
10797	MTHFD2
10815	CPLX1
10857	PGRMC1
10867	TSPAN9
10891	PPARGC1A
10988	METAP2
11069	RAPGEF4
11118	BTN3A2
11138	TBC1D8
11151	CORO1A
11164	NUDT5
11167	FSTL1
11211	FZD10
11228	RASSF8
11252	PACSIN2
11259	FILIP1L
11279	KLF8
11342	RNF13
22801	ITGA11
22824	HSPA4L
22881	ANKRD6
22987	SV2C
22996	TTC39A
23046	KIF21B
23094	SIPA1L3
23109	DDN
23111	SPART
23180	RFTN1
23199	GSE1
23212	RRS1
23259	DDHD2
23263	MCF2L
23274	CLEC16A
23284	ADGRL3
23406	COTL1
23428	SLC7A8
23460	ABCA6
23467	NPTXR
23479	ISCU
23484	LEPROTL1
23504	RIMBP2
23516	SLC39A14
23642	SNHG1
23780	APOL2
24139	EML2
25759	SHC2
25769	SLC24A2
25818	KLK5
25841	ABTB2
25854	FAM149A
25871	NEPRO
25900	IFFO1
25907	TMEM158
25924	MYRIP
25946	ZNF385A
25953	PNKD
25989	ULK3
25999	CLIP3
26010	SPATS2L
26031	OSBPL3
26059	ERC2
26232	FBXO2
26996	GPR160
27077	B9D1
27132	CPNE7
27163	NAAA
27252	KLHL20
10505	SEMA4F
10555	AGPAT2
10655	DMRT2
10669	CGREF1
10673	TNFSF13B
10752	CHL1
10776	ARPP19
10783	NEK6
10797	MTHFD2
10815	CPLX1
10867	TSPAN9
10891	PPARGC1A
11069	RAPGEF4
11138	TBC1D8
11151	CORO1A
11164	NUDT5
11167	FSTL1
11211	FZD10
11228	RASSF8
11259	FILIP1L
11279	KLF8
22801	ITGA11
22881	ANKRD6
22987	SV2C
22996	TTC39A
23046	KIF21B
23109	DDN
23180	RFTN1
23212	RRS1
23245	ASTN2
23259	DDHD2
23263	MCF2L
23274	CLEC16A
23305	ACSL6
23370	ARHGEF18
23406	COTL1
23428	SLC7A8
23460	ABCA6
23467	NPTXR
23484	LEPROTL1
23504	RIMBP2
23642	SNHG1
23780	APOL2
24139	EML2
25769	SLC24A2
25818	KLK5
25841	ABTB2
25854	FAM149A
25871	NEPRO
25900	IFFO1
25907	TMEM158
25924	MYRIP
25946	ZNF385A
25953	PNKD
25989	ULK3
26031	OSBPL3
26059	ERC2
26232	FBXO2
26996	GPR160
27077	B9D1
27132	CPNE7
27163	NAAA
27254	CSDC2
27294	DHDH
27345	KCNMB4
28955	DEXI
29115	SAP30BP
29799	YPEL1
29803	REPIN1
29844	TFPT
29902	FAM216A
29997	NOP53
30850	CDR2L
49855	SCAPER
50486	G0S2
50853	VILL
51059	FAM135B
51074	APIP
51105	PHF20L1
51123	ZNF706
51134	CEP83
51155	JPT1
51161	C3orf18
51312	SLC25A37
51319	RSRC1
51334	PRR16
51375	SNX7
51393	TRPV2
51440	HPCAL4
51454	GULP1
51522	TMEM14C
51538	ZCCHC17
51642	MRPL48
51660	MPC1
51667	NUB1
51705	EMCN
53616	ADAM22
53826	FXYD6
54112	GPR88
54492	NEURL1B
54536	EXOC6
54550	NECAB2
54551	MAGEL2
54566	EPB41L4B
54793	KCTD9
54843	SYTL2
54847	SIDT1
219738	FAM241B
221294	NT5DC1
221421	RSPH9
222537	HS3ST5
253832	ZDHHC20
254102	EHBP1L1
260434	PYDC1
282969	FUOM
283209	PGM2L1
283284	IGSF22
284348	LYPD5
285780	LY86-AS1
286133	SCARA5
326624	RAB37
339263	C17orf51
342667	STAC2
348013	TMEM255B
349136	WDR86
374378	GALNT18
374875	HSD11B1L
386618	KCTD4
404217	CTXN1
407738	FAM19A1
494470	RNF165
503542	SPRN
574036	SERTAD4-AS1
642273	FAM110C
654502	IQCJ
768211	RELL1
100507436	MICA

340719	NANOS1
342667	STAC2
348013	TMEM255B
349136	WDR86
373156	GSTK1
373863	DND1
374378	GALNT18
386618	KCTD4
387357	THEMIS
389073	C2orf80
400745	SH2D5
404217	CTXN1
415116	PIM3
503542	SPRN
574036	SERTAD4-AS1
642273	FAM110C
646424	SPINK8
646627	LYPD8
654502	IQCJ
654790	PCP4L1
768211	RELL1
100507436	MICA

27254	CSDC2
27294	DHDH
27341	RRP7A
27345	KCNMB4
28231	SLCO4A1
28232	SLCO3A1
28955	DEXI
28966	SNX24
29126	CD274
29799	YPEL1
29803	REPIN1
29844	TFPT
29902	FAM216A
29904	EEF2K
30850	CDR2L
50486	G0S2
50853	VILL
51022	GLRX2
51059	FAM135B
51074	APIP
51123	ZNF706
51134	CEP83
51312	SLC25A37
51319	RSRC1
51330	TNFRSF12A
51334	PRR16
51375	SNX7
51393	TRPV2
51440	HPCAL4
51454	GULP1
51522	TMEM14C
51538	ZCCHC17
51635	DHRS7
51642	MRPL48
51660	MPC1
51667	NUB1
51705	EMCN
53826	FXYD6
54112	GPR88
54206	ERRFI1
54331	GNG2
54407	SLC38A2
54492	NEURL1B
54536	EXOC6
54550	NECAB2
54551	MAGEL2
54566	EPB41L4B
54680	ZNHIT6
54793	KCTD9
54843	SYTL2
54847	SIDT1
54874	FNBP1L
54972	TMEM132A
55006	TRMT61B
55022	PID1
55040	EPN3
55062	WIPI1
55086	CXorf57
55118	CRTAC1
55122	AKIRIN2
55160	ARHGEF10L
55170	PRMT6
55203	LGI2
55208	DCUN1D2
55244	SLC47A1
55315	SLC29A3
55353	LAPTM4B
55359	STYK1
55502	HES6
55509	BATF3
55591	VEZT
55686	MREG
55714	TENM3
55790	CSGALNACT1
55800	SCN3B
55853	IDI2-AS1
55884	WSB2
55897	MESP1
55902	ACSS2
56172	ANKH
56302	TRPV5
56477	CCL28
56616	DIABLO
56648	EIF5A2
56666	PANX2
56848	SPHK2
56888	KCMF1
56906	THAP10
56934	CA10
56937	PMEPA1
56967	C14orf132
57110	HRASLS
57180	ACTR3B
57185	NIPAL3
57194	ATP10A
57406	ABHD6
57453	DSCAML1
57465	TBC1D24
57484	RNF150
57493	HEG1
57495	NWD2
57496	MKL2
57519	STARD9
57526	PCDH19
57554	LRRC7
57596	BEGAIN
57644	MYH7B

54874	FNBP1L
55040	EPN3
55086	CXorf57
55118	CRTAC1
55122	AKIRIN2
55160	ARHGEF10L
55170	PRMT6
55190	NUDT11
55208	DCUN1D2
55244	SLC47A1
55315	SLC29A3
55353	LAPTM4B
55359	STYK1
55502	HES6
55509	BATF3
55591	VEZT
55686	MREG
55714	TENM3
55800	SCN3B
55853	IDI2-AS1
55884	WSB2
55897	MESP1
56172	ANKH
56477	CCL28
56648	EIF5A2
56666	PANX2
56884	FSTL5
56906	THAP10
56934	CA10
56937	PMEPA1
56967	C14orf132
56971	CEACAM19
56990	CDC42SE2
57110	HRASLS
57194	ATP10A
57406	ABHD6
57453	DSCAML1
57465	TBC1D24
57484	RNF150
57495	NWD2
57496	MKL2
57519	STARD9
57526	PCDH19
57596	BEGAIN
57630	SH3RF1
57631	LRCH2
57644	MYH7B
57699	CPNE5
57718	PPP4R4
57761	TRIB3
60626	RIC8A
63941	NECAB3
63974	NEUROD6
63982	ANO3
64131	XYLT1
64132	XYLT2
64135	IFIH1
64137	ABCG4
64149	C17orf75
64150	DIO3OS
64333	ARHGAP9
64792	IFT22
64881	PCDH20
65078	RTN4R
65110	UPF3A
65263	PYCR3
65982	ZSCAN18
65997	RASL11B
66000	TMEM108
66008	TRAK2
78990	OTUB2
79012	CAMKV
79017	GGCT
79085	SLC25A23
79183	TTPAL
79442	LRRC2
79585	CORO7
79645	EFCAB1
79660	PPP1R3B
79720	VPS37B
79745	CLIP4
79750	ZNF385D
79754	ASB13
79822	ARHGAP28
79874	RABEP2
79930	DOK3
79957	PAQR6
79958	DENND1C
79993	ELOVL7
80020	FOXRED2
80036	TRPM3
80119	PIF1
80176	SPSB1
80179	MYO19
80307	FER1L4
80323	CCDC68
80774	LIMD2
80818	ZNF436
80820	EEPD1
80854	SETD7
81033	KCNH6
81539	SLC38A1
81552	VOPP1
81553	FAM49A
81602	CDADC1
81605	URM1
81849	ST6GALNAC5

57699	CPNE5	83445	GSG1
57718	PPP4R4	83468	GLT8D2
57761	TRIB3	83482	SCRT1
60625	DHX35	83546	RTBDN
63974	NEUROD6	83547	RILP
63982	ANO3	83660	TLN2
64131	XYLT1	83690	CRISPLD1
64132	XYLT2	83692	CD99L2
64135	IFIH1	83707	TRPT1
64137	ABCG4	83714	NRIP2
64149	C17orf75	83723	FAM57B
64150	DIO3OS	83787	ARMC10
64332	NFKBIZ	83875	BCO2
64333	ARHGAP9	83937	RASSF4
64420	SUSD1	83992	CTTNBP2
64781	CERK	84034	EMILIN2
64792	IFT22	84063	KIRREL2
64881	PCDH20	84109	QRFPR
65078	RTN4R	84221	SPATC1L
65110	UPF3A	84314	TMEM107
65263	PYCR3	84332	DYDC2
65992	DDRGK1	84439	HHIPL1
65997	RASL11B	84524	ZC3H8
65999	LRRC61	84525	HOPX
66000	TMEM108	84542	KIAA1841
66008	TRAK2	84623	KIRREL3
78986	DUSP26	84691	FAM71F1
78990	OTUB2	84709	MGARP
79012	CAMKV	84769	MPV17L2
79017	GGCT	84803	GPAT3
79075	DSCC1	84812	PLCD4
79085	SLC25A23	84864	RIOX2
79158	GNPTAB	84870	RSPO3
79183	TTPAL	84936	ZFYVE19
79442	LRRC2	84937	ZNRF1
79585	CORO7	84957	RELT
79645	EFCAB1	85015	USP45
79660	PPP1R3B	85352	SHISAL1
79720	VPS37B	85461	TANC1
79745	CLIP4	89782	LMLN
79750	ZNF385D	89846	FGD3
79754	ASB13	90102	PHLDB2
79762	C1orf115	90488	TMEM263
79767	ELMO3	90523	MLIP
79822	ARHGAP28	90850	ZNF598
79874	RABEP2	90861	JPT2
79884	MAP9	90865	IL33
79887	PLBD1	91133	L3MBTL4
79930	DOK3	91252	SLC39A13
79956	ERMP1	91624	NEXN
79957	PAQR6	91683	SYT12
79962	DNAJC22	92017	SNX29
79990	PLEKHH3	92293	TMEM132C
79993	ELOVL7	92335	STRADA
80020	FOXRED2	92399	MRRF
80036	TRPM3	92597	MOB1B
80119	PIF1	92610	TIFA
80176	SPSB1	94160	ABCC12
80179	MYO19	113263	GLCCI1
80307	FER1L4	113452	TMEM54
80323	CCDC68	113675	SDSL
80774	LIMD2	114299	PALM2
80818	ZNF436	114571	SLC22A9
80820	EEPD1	114787	GPRIN1
80852	GRIP2	114804	RNF157
80853	KDM7A	114990	VASN
80854	SETD7	116028	RMI2
81033	KCNH6	116135	LRRC3B
81539	SLC38A1	116150	NUS1
81552	VOPP1	116535	MRGPRF
81553	FAM49A	116966	WDR17
81605	URM1	117154	DACH2
81619	TSPAN14	117245	HRASLS5
81849	ST6GALNAC5	118427	OLFM3
83445	GSG1	118429	ANTXR2
83468	GLT8D2	119587	CPXM2
83482	SCRT1	122622	ADSSL1
83547	RILP	122953	JDP2
83660	TLN2	126755	LRRC38
83690	CRISPLD1	127833	SYT2
83692	CD99L2	128434	VSTM2L
83707	TRPT1	128611	ZNF831
83714	NRIP2	130399	ACVR1C
83723	FAM57B	132160	PPM1M
83787	ARMC10	132321	C4orf33
83851	SYT16	133418	EMB
83875	BCO2	134548	SOWAHA
83937	RASSF4	138428	PTRH1
83986	FAM234A	139221	MUM1L1
83992	CTTNBP2	139411	PTCHD1
84034	EMILIN2	139728	PNCK
84063	KIRREL2	143279	HECTD2
84109	QRFPR	144348	ZNF664
84187	TMEM164	144402	CPNE8
84221	SPATC1L	146760	RTN4RL1
84314	TMEM107	147463	ANKRD29
84332	DYDC2	147650	SPACA6
84439	HHIPL1	147968	CAPN12
84525	HOPX	148281	SYT6
84542	KIAA1841	148479	PHF13
84572	GNPTG	149473	CCDC24
84623	KIRREL3	151516	ASPRV1
84691	FAM71F1	152189	CMTM8
84735	CNDP1	152273	FGD5
84769	MPV17L2	152940	C4orf45
84803	GPAT3	155382	VPS37D
84812	PLCD4	160760	PPTC7

84864	RIOX2
84936	ZFYVE19
84937	ZNRF1
84957	RELT
85352	SHISAL1
85461	TANC1
87178	PNPT1
89782	LMLN
89846	FGD3
90102	PHLDB2
90488	TMEM263
90523	MLIP
90850	ZNF598
90861	JPT2
91133	L3MBTL4
91252	SLC39A13
91624	NEXN
91683	SYT12
92293	TMEM132C
92335	STRADA
92399	MRRF
92597	MOB1B
92610	TIFA
93587	TRMT10A
113263	GLCCI1
113452	TMEM54
113675	SDSL
113829	SLC35A4
114299	PALM2
114571	SLC22A9
114787	GPRIN1
114800	CCDC85A
114804	RNF157
114880	OSBPL6
114926	SMIM19
114990	VASN
115584	SLC5A11
116028	RMI2
116135	LRRC3B
116535	MRGPRF
116832	RPL39L
116966	WDR17
117154	DACH2
117245	HRASLS5
118427	OLFM3
119587	CPXM2
122622	ADSSL1
122953	JDP2
124976	SPNS2
126755	LRRC38
127003	C1orf194
127833	SYT2
128434	VSTM2L
130399	ACVR1C
132160	PPM1M
132321	C4orf33
133418	EMB
134548	SOWAHA
138428	PTRH1
139221	MUM1L1
139411	PTCHD1
139728	PNCK
140733	MACROD2
143279	HECTD2
144348	ZNF664
144402	CPNE8
146760	RTN4RL1
147463	ANKRD29
147968	CAPN12
148014	TTC9B
148281	SYT6
149473	CCDC24
150209	AIFM3
151516	ASPRV1
152189	CMTM8
152273	FGD5
152940	C4orf45
154141	MBOAT1
154790	CLEC2L
160760	PPTC7
162494	RHBDL3
163183	SYNE4
163732	CITED4
163782	KANK4
165215	FAM171B
167681	PRSS35
167691	LCA5
170261	ZCCHC12
170850	KCNG3
192668	CYS1
196383	RILPL2
196527	ANO6
200058	FLJ23867
200942	KLHDC8B
201191	SAMD14
202333	CMYA5
203286	ANKS6
219348	PLAC9
219738	FAM241B
220164	DOK6
220202	ATOH7
221294	NT5DC1
221336	BEND6
221421	RSPH9
221662	RBM24
222537	HS3ST5
253832	ZDHHC20
162494	RHBDL3
163183	SYNE4
163782	KANK4
165215	FAM171B
167681	PRSS35
167691	LCA5
170850	KCNG3
192668	CYS1
196383	RILPL2
199800	ADM5
200058	FLJ23867
200942	KLHDC8B
201191	SAMD14
202333	CMYA5
203286	ANKS6
219738	FAM241B
220202	ATOH7
221294	NT5DC1
221336	BEND6
221421	RSPH9
222537	HS3ST5
253782	CERS6
253832	ZDHHC20
254102	EHBP1L1
254170	FBXO33
254263	CNIH2
254552	NUDT8
256281	NUDT14
260434	PYDC1
282969	FUOM
283143	LINC00900
283209	PGM2L1
283284	IGSF22
284339	TMEM145
284348	LYPD5
284415	VSTM1
284454	LOC284454
284485	RIIAD1
284611	FAM102B
284716	RIMKLA
285598	ARL10
285755	PPIL6
285780	LY86-AS1
286133	SCARA5
326624	RAB37
339263	C17orf51
339983	NAT8L
340348	TSPAN33
340719	NANOS1
342667	STAC2
347902	AMIGO2
348013	TMEM255B
349136	WDR86
373156	GSTK1
373863	DND1
374378	GALNT18
374875	HSD11B1L
375057	STUM
386618	KCTD4
387357	THEMIS
389073	C2orf80
389941	C1QL3
400120	SERTM1
400569	MED11
400745	SH2D5
401994	OR14I1
404217	CTXN1
407738	FAM19A1
415116	PIM3
441108	C5orf56
494470	RNF165
503542	SPRN
574029	DUSP5P1
574036	SERTAD4-AS1
642273	FAM110C
642852	LOC642852
643037	C11orf97
646627	LYPD8
654502	IQCJ
654790	PCP4L1
768211	RELL1
100170841	EPOP
100507436	MICA

254102	EHBP1L1
254170	FBXO33
254263	CNIH2
260434	PYDC1
282969	FUOM
282973	JAKMIP3
283131	NEAT1
283143	LINC00900
283209	PGM2L1
283284	IGSF22
283316	CD163L1
284069	FAM171A2
284119	CAVIN1
284339	TMEM145
284348	LYPD5
284415	VSTM1
284454	LOC284454
284485	RIIAD1
284611	FAM102B
284716	RIMKLA
285220	EPHA6
285598	ARL10
285613	RELL2
285755	PPIL6
285780	LY86-AS1
286133	SCARA5
286336	FAM78A
326624	RAB37
337876	CHSY3
339263	C17orf51
339983	NAT8L
340719	NANOS1
342667	STAC2
347902	AMIGO2
348013	TMEM255B
349136	WDR86
373156	GSTK1
373863	DND1
374378	GALNT18
374875	HSD11B1L
375057	STUM
375449	MAST4
386618	KCTD4
387775	SLC22A10
389073	C2orf80
389941	C1QL3
400569	MED11
400745	SH2D5
401647	GOLGA7B
404037	HAPLN4
404217	CTXN1
407738	FAM19A1
408263	FNDC9
415116	PIM3
441108	C5orf56
442319	ZNF727
494470	RNF165
503542	SPRN
574036	SERTAD4-AS1
642273	FAM110C
642852	LOC642852
643037	C11orf97
646627	LYPD8
654502	IQCJ
654790	PCP4L1
768211	RELL1
100093630	SNHG8
100170841	EPOP
100288911	LOC100288911
100507436	MICA

Abbreviations: rsFC, resting-state functional connectivity; DS, differential stability; A4hf, head and face region of area 4; A6cdl, caudal dorsolateral area 6; A4ul, upper limb region of area 4; A4ll, lower limb region of area 4; A1/2/3ulhf, upper limb, head and face region of area 1/2/3; A1/2/3tru, trunk region of area 1/2/3.
