# Supplementary material for: Transcriptional substrates underlying functional connectivity profiles of subregions within the human sensorimotor cortex
Source: Hum Brain Mapp. 2022 Jul 27;43(18):5562–78. doi: 10.1002/hbm.26031 (PMC9704778; doi:10.1002/hbm.26031)
Supplement: Supplementary file 1 — Appendix S1 Supplementary Information [file HBM-43-5562-s001.zip › HBM_26031_Supplementary file 6.pdf]

The genes related to rsFC of sensorimotor subregions based on BOLD data with GSR

A4hf		A6cdl		A4ul		A4ll		A1/2/3ulhf		A1/2/3tru	
GeneID	Genesymbol	GeneID	Genesymbol	GeneID	Genesymbol	GeneID	Genesymbol	GeneID	Genesymbol	GeneID	Genesymbol
176	ACAN	2632	GBE1	92	ACVR2A	-	-	92	ACVR2A	308	ANXA5
367	AR	2830	GPR6	98	ACYP2			133	ADM	445	ASS1
445	ASS1	4741	NEFM	133	ADM			176	ACAN	784	CACNB3
1006	CDH8	4744	NEFH	176	ACAN			292	SLC25A5	793	CALB1
1381	CRABP1	6330	SCN4B	204	AK2			308	ANXA5	814	CAMK4
1607	DGKB	6482	ST3GAL1	272	AMPD3			367	AR	1300	COL10A1
2634	GBP2	22987	SV2C	292	SLC25A5			445	ASS1	1755	DMBT1
2766	GMPR	51059	FAM135B	308	ANXA5			783	CACNB2	2044	EPHA5
2830	GPR6	54492	NEURL1B	320	APBA1			793	CALB1	2830	GPR6
2952	GSTT1	56950	SMYD2	367	AR			814	CAMK4	2952	GSTT1
3067	HDC	56967	C14orf132	445	ASS1			817	CAMK2D	3067	HDC
3290	HSD11B1	57616	TSHZ3	460	ASTN1			862	RUNX1T1	3706	ITPKA
3755	KCNG1	84250	SLF1	586	BCAT1			1006	CDH8	3783	KCNN4
3783	KCNN4	90523	MLIP	784	CACNB3			1031	CDKN2C	4286	MITF
4258	MGST2	163782	KANK4	793	CALB1			1381	CRABP1	4638	MYLK
4259	MGST3			814	CAMK4			1501	CTNND2	4703	NEB
4638	MYLK			817	CAMK2D			1522	CTSZ	5774	PTPN3
4703	NEB			862	RUNX1T1			1607	DGKB	5794	PTPRH
4715	NDUFB9			1006	CDH8			2101	ESRRA	5860	QDPR
4751	NEK2			1031	CDKN2C			2104	ESRRG	6326	SCN2A
4856	NOV			1073	CFL2			2161	F12	6330	SCN4B
5121	PCP4			1131	CHRM3			2173	FABP7	6696	SPP1
6017	RLBP1			1381	CRABP1			2560	GABRB1	6804	STX1A
6330	SCN4B			1522	CTSZ			2766	GMPR	7103	TSPAN8
6804	STX1A			1607	DGKB			2823	GPM6A	7781	SLC30A3
7103	TSPAN8			1755	DMBT1			2830	GPR6	8209	C21orf33
8001	GLRA3			2044	EPHA5			2888	GRB14	8704	B4GALT2
8704	B4GALT2			2070	EYA4			2898	GRIK2	8717	TRADD
8717	TRADD			2101	ESRRA			2918	GRM8	8871	SYNJ2
8938	BAIAP3			2104	ESRRG			2952	GSTT1	9033	PKD2L1
9033	PKD2L1			2161	F12			3067	HDC	9312	KCNB2
9312	KCNB2			2173	FABP7			3290	HSD11B1	9607	CARTPT
9454	HOMER3			2322	FLT3			3706	ITPKA	10083	USH1C
9607	CARTPT			2560	GABRB1			3736	KCNA1	10395	DLC1
10395	DLC1			2620	GAS2			3738	KCNA3	10451	VAV3
10451	VAV3			2632	GBE1			3741	KCNA5	10673	TNFSF13B
10505	SEMA4F			2766	GMPR			3755	KCNG1	10690	FUT9
10673	TNFSF13B			2823	GPM6A			3783	KCNN4	22801	ITGA11
22801	ITGA11			2830	GPR6			3902	LAG3	22941	SHANK2
22996	TTC39A			2898	GRIK2			4062	LY6H	22987	SV2C
23406	COTL1			2918	GRM8			4258	MGST2	23180	RFTN1
25841	ABTB2			2952	GSTT1			4286	MITF	23406	COTL1
51454	GULP1			3067	HDC			4609	MYC	25841	ABTB2
55244	SLC47A1			3208	HPCA			4638	MYLK	26010	SPATS2L
56937	PMEPA1			3231	HOXD1			4703	NEB	50486	G0S2
56967	C14orf132			3290	HSD11B1			4715	NDUFB9	51454	GULP1
57495	NWD2			3624	INHBA			4744	NEFH	54894	RNF43
57526	PCDH19			3679	ITGA7			4751	NEK2	55244	SLC47A1
63974	NEUROD6			3688	ITGB1			4856	NOV	56967	C14orf132
65997	RASL11B			3706	ITPKA			5090	PBX3	57495	NWD2
66000	TMEM108			3736	KCNA1			5121	PCP4	57526	PCDH19
66008	TRAK2			3738	KCNA3			5176	SERPINF1	57619	SHROOM3
79822	ARHGAP28			3741	KCNA5			5361	PLXNA1	65078	RTN4R
80020	FOXRED2			3755	KCNG1			5409	PNMT	66000	TMEM108
80307	FER1L4			3783	KCNN4			5412	UBL3	66008	TRAK2
81849	ST6GALNAC5			3902	LAG3			5547	PRCP	79762	C1orf115
83660	TLN2			4062	LY6H			5600	MAPK11	79957	PAQR6
83714	NRIP2			4082	MARCKS			5774	PTPN3	80020	FOXRED2
84034	EMILIN2			4258	MGST2			5991	RFX3	80323	CCDC68
84109	QRFPR			4259	MGST3			5993	RFX5	80774	LIMD2
90523	MLIP			4286	MITF			6326	SCN2A	81849	ST6GALNAC5
91522	COL23A1			4345	CD200			6330	SCN4B	83660	TLN2
94233	OPN4			4354	MPP1			6543	SLC8A2	83714	NRIP2
113452	TMEM54			4599	MX1			6696	SPP1	84034	EMILIN2
118427	OLFM3			4609	MYC			6804	STX1A	84539	MCHR2
118429	ANTXR2			4625	MYH7			7062	TCHH	90523	MLIP
127833	SYT2			4638	MYLK			7068	THRB	90861	JPT2
139221	MUM1L1			4703	NEB			7103	TSPAN8	91522	COL23A1
144402	CPNE8			4715	NDUFB9			7781	SLC30A3	114787	GPRIN1
147968	CAPN12			4744	NEFH			8001	GLRA3	114990	VASN
148281	SYT6			4751	NEK2			8209	C21orf33	118427	OLFM3
163782	KANK4			4856	NOV			8404	SPARCL1	144348	ZNF664
254102	EHBP1L1			4886	NPY1R			8704	B4GALT2	144402	CPNE8
283316	CD163L1			4889	NPY5R			8715	NOL4	147968	CAPN12
400745	SH2D5			5090	PBX3			8717	TRADD	154141	MBOAT1
503542	SPRN			5121	PCP4			8871	SYNJ2	163732	CITED4
642273	FAM110C			5176	SERPINF1			8938	BAIAP3	163782	KANK4
646424	SPINK8			5184	PEPD			9033	PKD2L1	221294	NT5DC1
				5409	PNMT			9312	KCNB2	254102	EHBP1L1
				5412	UBL3			9454	HOMER3	283316	CD163L1
				5547	PRCP			9607	CARTPT	348013	TMEM255B
				5774	PTPN3			9651	PLCH2	503542	SPRN
				5794	PTPRH			9770	RASSF2	642273	FAM110C
				5801	PTPRR			10083	USH1C	100507436	MICA
				5806	PTX3			10154	PLXNC1		
				5860	QDPR			10231	RCAN2		
				5874	RAB27B			10332	CLEC4M		
				5950	RBP4			10384	BTN3A3		
				5991	RFX3			10395	DLC1		
				5993	RFX5			10451	VAV3		
				6272	SORT1			10505	SEMA4F		
				6320	CLEC11A			10613	ERLIN1		
				6326	SCN2A			10655	DMRT2		
				6330	SCN4B			10673	TNFSF13B		
				6541	SLC7A1			10690	FUT9		
				6543	SLC8A2			10891	PPARGC1A		
				6659	SOX4			11259	FILIP1L		
				6696	SPP1			11279	KLF8		
				6804	STX1A			22801	ITGA11		
				6890	TAP1			22987	SV2C		
				7068	THRB			22996	TTC39A		
				7103	TSPAN8			23046	KIF21B		

7301	TYRO3
7781	SLC30A3
8001	GLRA3
8099	CDK2AP1
8209	C21orf33
8404	SPARCL1
8611	PLPP1
8704	B4GALT2
8715	NOL4
8717	TRADD
8792	TNFRSF11A
8871	SYNJ2
8938	BAIAP3
8996	NOL3
9033	PKD2L1
9120	SLC16A6
9256	TSPOAP1
9312	KCNB2
9379	NRXN2
9454	HOMER3
9473	THEMIS2
9607	CARTPT
9609	RAB36
9630	GNA14
9636	ISG15
9651	PLCH2
9770	RASSF2
9854	C2CD2L
9856	KIAA0319
9911	TMCC2
10083	USH1C
10154	PLXNC1
10268	RAMP3
10332	CLEC4M
10384	BTN3A3
10395	DLC1
10451	VAV3
10505	SEMA4F
10613	ERLIN1
10655	DMRT2
10673	TNFSF13B
10690	FUT9
10783	NEK6
10865	ARID5A
10891	PPARGC1A
11138	TBC1D8
11164	NUDT5
11259	FILIP1L
11279	KLF8
11342	RNF13
22801	ITGA11
22881	ANKRD6
22941	SHANK2
22987	SV2C
22996	TTC39A
23046	KIF21B
23406	COTL1
23467	NPTXR
23479	ISCU
23484	LEPROTL1
23630	KCNE5
23642	SNHG1
25759	SHC2
25841	ABTB2
25953	PNKD
25989	ULK3
27077	B9D1
27132	CPNE7
27294	DHDH
28955	DEXI
28966	SNX24
29799	YPEL1
29803	REPIN1
30850	CDR2L
50486	G0S2
50853	VILL
51022	GLRX2
51059	FAM135B
51226	COPZ2
51330	TNFRSF12A
51440	HPCAL4
51454	GULP1
51522	TMEM14C
51635	DHRS7
51642	MRPL48
51660	MPC1
54112	GPR88
54361	WNT4
54492	NEURL1B
54536	EXOC6
54550	NECAB2
54793	KCTD9
54847	SIDT1
55040	EPN3
55244	SLC47A1
55315	SLC29A3
55353	LAPTM4B
55509	BATF3
55591	VEZT
55686	MREG
55800	SCN3B
55853	IDI2-AS1
55884	WSB2
55897	MESP1
56477	CCL28
56937	PMEPA1
56950	SMYD2
23406	COTL1
23484	LEPROTL1
23504	RIMBP2
23630	KCNE5
25759	SHC2
25841	ABTB2
25989	ULK3
30850	CDR2L
50853	VILL
51059	FAM135B
51134	CEP83
51454	GULP1
51522	TMEM14C
51642	MRPL48
51660	MPC1
54112	GPR88
54536	EXOC6
54550	NECAB2
54847	SIDT1
55040	EPN3
55244	SLC47A1
55315	SLC29A3
55353	LAPTM4B
55800	SCN3B
55853	IDI2-AS1
55897	MESP1
56937	PMEPA1
56967	C14orf132
57465	TBC1D24
57495	NWD2
57496	MKL2
57526	PCDH19
57761	TRIB3
63974	NEUROD6
64131	XYLT1
64135	IFIH1
65997	RASL11B
66000	TMEM108
66008	TRAK2
79660	PPP1R3B
79745	CLIP4
79762	C1orf115
79822	ARHGAP28
79874	RABEP2
79957	PAQR6
80020	FOXRED2
80119	PIF1
80307	FER1L4
80774	LIMD2
80854	SETD7
81033	KCNH6
81849	ST6GALNAC5
83445	GSG1
83468	GLT8D2
83660	TLN2
83714	NRIP2
83723	FAM57B
83853	ROPN1L
84034	EMILIN2
84109	QRFPR
84314	TMEM107
84803	GPAT3
84812	PLCD4
85461	TANC1
90523	MLIP
90861	JPT2
91522	COL23A1
113675	SDSL
114787	GPRIN1
114990	VASN
118427	OLFM3
126755	LRRC38
127833	SYT2
130399	ACVR1C
139221	MUM1L1
144402	CPNE8
147463	ANKRD29
147968	CAPN12
148281	SYT6
149473	CCDC24
152189	CMTM8
152940	C4orf45
160760	PPTC7
163732	CITED4
163782	KANK4
221294	NT5DC1
222537	HS3ST5
254102	EHBP1L1
283316	CD163L1
284716	RIMKLA
286133	SCARA5
326624	RAB37
387357	THEMIS
400745	SH2D5
503542	SPRN
642273	FAM110C
100507436	MICA

56967	C14orf132
57185	NIPAL3
57453	DSCAML1
57465	TBC1D24
57495	NWD2
57496	MKL2
57526	PCDH19
57554	LRRC7
57761	TRIB3
60484	HAPLN2
63974	NEUROD6
64135	IFIH1
64149	C17orf75
65078	RTN4R
65997	RASL11B
66000	TMEM108
66008	TRAK2
79012	CAMKV
79645	EFCAB1
79660	PPP1R3B
79745	CLIP4
79762	C1orf115
79822	ARHGAP28
79957	PAQR6
79993	ELOVL7
80020	FOXRED2
80036	TRPM3
80119	PIF1
80176	SPSB1
80307	FER1L4
80323	CCDC68
80774	LIMD2
80854	SETD7
81849	ST6GALNAC5
83445	GSG1
83468	GLT8D2
83660	TLN2
83714	NRIP2
83723	FAM57B
83875	BCO2
84034	EMILIN2
84109	QRFPR
84314	TMEM107
84623	KIRREL3
84803	GPAT3
84937	ZNRF1
85461	TANC1
90423	ATP6V1E2
90523	MLIP
90861	JPT2
91522	COL23A1
91624	NEXN
92399	MRRF
92610	TIFA
113675	SDSL
114571	SLC22A9
114787	GPRIN1
114990	VASN
118427	OLFM3
119587	CPXM2
126755	LRRC38
127833	SYT2
130399	ACVR1C
132160	PPM1M
133418	EMB
134548	SOWAHA
143279	HECTD2
144348	ZNF664
144402	CPNE8
147463	ANKRD29
147968	CAPN12
148281	SYT6
149473	CCDC24
152940	C4orf45
154141	MBOAT1
160760	PPTC7
163183	SYNE4
163732	CITED4
163782	KANK4
192668	CYS1
221294	NT5DC1
221662	RBM24
222537	HS3ST5
253832	ZDHHC20
254102	EHBP1L1
283316	CD163L1
284415	VSTM1
284716	RIMKLA
286133	SCARA5
326624	RAB37
337876	CHSY3
348013	TMEM255B
349136	WDR86
374378	GALNT18
386618	KCTD4
387357	THEMIS
387775	SLC22A10
400745	SH2D5
404217	CTXN1
494470	RNF165
503542	SPRN
642273	FAM110C
654502	IQCJ
100507436	MICA

Abbreviations: rsFC, resting-state functional connectivity; BOLD, blood-oxygen-level-dependent; GSR, global signal regression; A4hf, head and face region of area 4; A6cdl, caudal dorsolateral area 6; A4ul, upper limb region of area 4; A4ll, lower limb region of area 4; A1/2/3ulhf, upper limb, head and face region of area 1/2/3; A1/2/3tru, trunk region of area 1/2/3.
