# Supplementary material for: Transcriptional substrates underlying functional connectivity profiles of subregions within the human sensorimotor cortex
Source: Hum Brain Mapp. 2022 Jul 27;43(18):5562–78. doi: 10.1002/hbm.26031 (PMC9704778; doi:10.1002/hbm.26031)
Supplement: Supplementary file 1 — Appendix S1 Supplementary Information [file HBM-43-5562-s001.zip › HBM_26031_Supplementary materials_HBM_R2.doc]

**­Supplementary materials**

**Table S1. Demographic information of the discovery and validation datasets**

| **Dataset** | **Sample size** | **Age (years)** | **Gender (F/M)** | **FD (mm)** |
| --- | --- | --- | --- | --- |
| Discovery | 361 | 28.84 ± 10.83 | 183/178 | 0.13 ± 0.07 |
| CNP | 103 | 30.87 ± 8.56 | 47/56 | 0.17 ± 0.08 |
| SALD | 329 | 37.81 ± 13.79 | 207/122 | 0.15 ± 0.08 |

Age and FD are expressed as mean ± standard deviation. Abbreviations: CNP, Consortium for Neuropsychiatric Phenomics; SALD, Southwest University Adult Lifespan Dataset; F, female; M, male; FD, frame-wise displacement.

**Table S2.** Resting-state fMRI parameters for three datasets

| **Parameters** | **Discovery** | **CNP** | **SALD** |
| --- | --- | --- | --- |
| Scanner | 3.0T General Electric Discovery MR750w | 3.0T Siemens Trio | 3.0T Siemens Trio |
| Sequence | GRE-SS-EPI | T2^*^-weighted EPI | GRE-EPI |
| TR (ms) | 2000 | 2000 | 2000 |
| TE (ms) | 30 | 30 | 30 |
| FA (°) | 90 | 90 | 90 |
| FOV (mm^2^) | 220 × 220 | 192 × 192 | 220 × 220 |
| Matrix size | 64 × 64 | 64 × 64 | 64 × 64 |
| Slice thickness (mm) | 3 | 4 | 3 |
| Slice gap (mm) | 1 | - | 1 |
| Slices | 35 | 34 | 32 |
| Time points | 185 | 152 | 242 |

Abbreviations: fMRI, functional magnetic resonance imaging; CNP, Consortium for Neuropsychiatric Phenomics; SALD, Southwest University Adult Lifespan Dataset; GRE, gradient echo; SS, single shot; EPI, echo planar imaging; TR, repetition time; TE, echo time; FA, flip angle; FOV, field of view.

**Table S3.** Demographic information of the six adult donors in the AHBA

| **Donor** | **Age (years)** | **Gender** | **Ethnicity** | **Hemisphere** | **Post-mortem interval (h)** |
| --- | --- | --- | --- | --- | --- |
| H0351.2001 | 24 | Male | African American | Both | 23 |
| H0351.2002 | 39 | Male | African American | Both | 10 |
| H0351.1009 | 57 | Male | Caucasian | Left | 25.5 |
| H0351.1012 | 31 | Male | Caucasian | Left | 17.5 |
| H0351.1015 | 49 | Female | Hispanic | Left | 30 |
| H0351.1016 | 55 | Male | Caucasian | Left | 18 |

Abbreviations: AHBA, Allen Human Brain Atlas.

**Table S4.** Results of spatially-constrained permutation tests

| **Sensorimotor subregion** | **rsFC-related genes** | ***P*_perm_ value** |
| --- | --- | --- |
| A4hf  A6cdl | 470  69 | < 0.001  < 0.001 |
| A4ul  A4t  A4tl  A1/2/3ll  A4ll | 821  11  5  2  35 | < 0.001  < 0.001  0.769  1  < 0.001 |
| A1/2/3ulhf  A1/2/3tonIa  A2 | 734  15  50 | < 0.001  0.009  0.711 |
| A1/2/3tru | 362 | < 0.001 |

Abbreviations: rsFC, resting-state functional connectivity; A4hf, head and face region of area 4; A6cdl, caudal dorsolateral area 6; A4ul, upper limb region of area 4; A4t, trunk region of area 4; A4tl, tongue and larynx region of area 4; A1/2/3ll, lower limb region of area 1/2/3; A4ll, lower limb region of area 4; A1/2/3ulhf, upper limb, head and face region of area 1/2/3; A1/2/3tonIa, tongue and larynx region of area 1/2/3; A2, area 2; A1/2/3tru, trunk region of area 1/2/3.

**Table S5.** Overlap between rsFC-related genes identified in the present study and 4­912 rsFC network-related genes reported by Anderson et al. (2018)

| **Sensorimotor subregion** | **rsFC-related genes** | **Overlap genes** | **OR** | ***P* value** |
| --- | --- | --- | --- | --- |
| A4hf  A6cdl | 470  69 | 381  53 | 13.81  10.69 | 2.20 × 10^-16^  2.20 × 10^-16^ |
| A4ul  A4ll | 821  35 | 631  29 | 10.71  15.60 | 2.20 × 10^-16^  5.06 × 10^-13^ |
| A1/2/3ulhf | 734 | 574 | 11.58 | 2.20 × 10^-16^ |
| A1/2/3tru | 362 | 292 | 13.46 | 2.20 × 10^-16^ |

Abbreviations: rsFC, resting-state functional connectivity; OR, odds ratio; A4hf, head and face region of area 4; A6cdl, caudal dorsolateral area 6; A4ul, upper limb region of area 4; A4ll, lower limb region of area 4; A1/2/3ulhf, upper limb, head and face region of area 1/2/3; A1/2/3tru, trunk region of area 1/2/3.

**Table S6.** Overlap between rsFC-related genes identified in the present study and 136 rsFC network-related genes reported by Richiardi et al. (2015)

| **Sensorimotor subregion** | **rsFC-related genes** | **Overlap genes** | **OR** | ***P* value** |
| --- | --- | --- | --- | --- |
| A4hf  A6cdl | 470  69 | 40  14 | 14.97  40.91 | 2.20 × 10^-16^  2.20 × 10^-16^ |
| A4ul  A4ll | 821  35 | 50  7 | 10.43  40.19 | 2.20 × 10^-16^  2.42 × 10^-9^ |
| A1/2/3ulhf | 734 | 47 | 11.01 | 2.20 × 10^-16^ |
| A1/2/3tru | 362 | 31 | 15.07 | 2.20 × 10^-16^ |

Abbreviations: rsFC, resting-state functional connectivity; OR, odds ratio; A4hf, head and face region of area 4; A6cdl, caudal dorsolateral area 6; A4ul, upper limb region of area 4; A4ll, lower limb region of area 4; A1/2/3ulhf, upper limb, head and face region of area 1/2/3; A1/2/3tru, trunk region of area 1/2/3.

**Table S7.** Numbers of genes related to rsFC of sensorimotor subregions at the DS cutoff threshold of 40% and overlap with those in the main analyses

| **Sensorimotor subregion** | **rsFC-related genes in the main analysis** | **rsFC-related genes at the threshold of 40%** | **Overlap genes** | **Overlap ratio** |
| --- | --- | --- | --- | --- |
| A4hf  A6cdl | 470  69 | 484  79 | 464  68 | 95.87%  86.08% |
| A4ul  A4ll | 821  35 | 797  38 | 777  35 | 97.49%  92.11% |
| A1/2/3ulhf | 734 | 733 | 707 | 96.45% |
| A1/2/3tru | 362 | 372 | 350 | 94.09% |

Abbreviations: rsFC, resting-state functional connectivity; DS, differential stability; A4hf, head and face region of area 4; A6cdl, caudal dorsolateral area 6; A4ul, upper limb region of area 4; A4ll, lower limb region of area 4; A1/2/3ulhf, upper limb, head and face region of area 1/2/3; A1/2/3tru, trunk region of area 1/2/3.

**Table S8.** Numbers of genes related to rsFC of sensorimotor subregions at the DS cutoff threshold of 60% and overlap with those in the main analyses

| **Sensorimotor subregion** | **rsFC-related genes in the main analysis** | **rsFC-related genes at the threshold of 60%** | **Overlap genes** | **Overlap ratio** |
| --- | --- | --- | --- | --- |
| A4hf  A6cdl | 470  69 | 445  62 | 440  62 | 98.88%  100.00% |
| A4ul  A4ll | 821  35 | 814  31 | 793  31 | 97.42%  100.00% |
| A1/2/3ulhf | 734 | 720 | 709 | 98.47% |
| A1/2/3tru | 362 | 346 | 338 | 97.69% |

Abbreviations: rsFC, resting-state functional connectivity; DS, differential stability; A4hf, head and face region of area 4; A6cdl, caudal dorsolateral area 6; A4ul, upper limb region of area 4; A4ll, lower limb region of area 4; A1/2/3ulhf, upper limb, head and face region of area 1/2/3; A1/2/3tru, trunk region of area 1/2/3.

**Table S9.** Numbers of genes related to rsFC of sensorimotor subregions based on BOLD data with GSR and overlap with those in the main analyses

| **Sensorimotor subregion** | **rsFC-related genes in the main analysis** | **rsFC-related genes based on BOLD data with GSR** | **Overlap genes** | **Overlap ratio** |
| --- | --- | --- | --- | --- |
| A4hf | 470 | 78 | 75 | 96.15% |
| A6cdl | 69 | 15 | 11 | 73.33% |
| A4ul | 821 | 313 | 294 | 93.93% |
| A4ll | 35 | 0 | 0 | - |
| A1/2/3ulhf | 734 | 199 | 189 | 94.97% |
| A1/2/3tru | 362 | 84 | 71 | 84.52% |

Abbreviations: rsFC, resting-state functional connectivity; BOLD, blood-oxygen-level-dependent; GSR, global signal regression; A4hf, head and face region of area 4; A6cdl, caudal dorsolateral area 6; A4ul, upper limb region of area 4; A4ll, lower limb region of area 4; A1/2/3ulhf, upper limb, head and face region of area 1/2/3; A1/2/3tru, trunk region of area 1/2/3.

**Figure S1.** The overlapping genes between different sensorimotor subregions. The numbers in brackets represent the numbers of genes related to rsFC of each sensorimotor subregion. Abbreviations: A4hf, head and face region of area 4; A6cdl, caudal dorsolateral area 6; A4ul, upper limb region of area 4; A4t, trunk region of area 4; A4ll, lower limb region of area 4; A1/2/3ulhf, upper limb, head and face region of area 1/2/3; A1/2/3tonIa, tongue and larynx region of area 1/2/3; A1/2/3tru, trunk region of area 1/2/3.
